# Quantifying Breslow intermediate reactivity in intermolecular Stetter reactions

**DOI:** 10.1039/d5sc05021a

**Published:** 2025-08-22

**Authors:** Zhuan Duan, Jiayun Zhu, Pankaj K. Majhi, Alister S. Goodfellow, AnnMarie C. O'Donoghue, Claire M. Young, Andrew D. Smith

**Affiliations:** a EaStCHEM, School of Chemistry, University of St Andrews Fife KY16 9ST UK alister.s.goodfellow@gmail.com claireyoungchemistry@gmail.com ads10@st-andrews.ac.uk; b Department of Chemistry, Durham University Durham DH1 3LE UK annmarie.odonoghue@durham.ac.uk

## Abstract

Quantification of the reactivity of the archetypal Breslow intermediate in NHC-mediated transformations has not been achievable to date and is regarded as a significant challenge due to multiple competitive pathways and their deconvolution. This manuscript describes the development of a kinetic approach to this challenge that avoids the influence of the competitive benzoin reaction and allows quantification of the reactivity of a Breslow intermediate derived from 2-pyridine carboxaldehyde and an *in situ* generated N-pentafluorophenyl substituted triazolinylidene NHC with a diverse range of Michael acceptors in the intermolecular Stetter reaction. Using this approach the pseudo first-order rate constants of >40 Michael acceptors, primarily derived from (*E*)-chalcones but also including a nitroolefin and malonic esters, were measured. Notably, incorporating electron-withdrawing substituents within the C(1)-aryl group of (*E*)-chalcones leads to a substantial enhancement in reactivity, with Hammett and Swain–Lupton analysis used to understand these observations. In addition, an unexpected additive substituent effect associated with the 4,4′-disubstitution of chalcones was observed, with DFT analysis offering insights into this intriguing phenomenon.

## Abstract 2

A kinetic method that allows quantification of Michael acceptor reactivity with a Breslow intermediate (BI) derived from pyridine-2-carboxaldehyde and an NHC is described.

## Graphical abstract

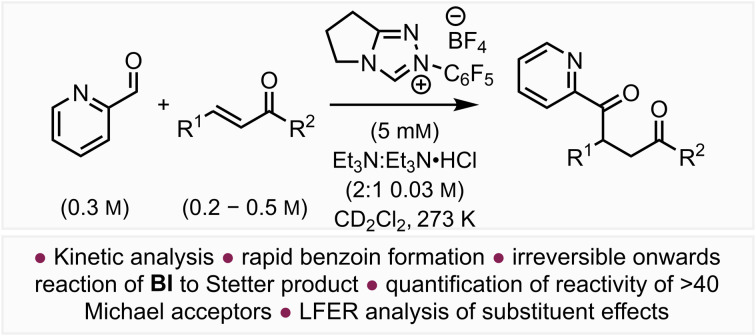

## 1 Introduction

Among the many synthetic transformations that are promoted by N-heterocyclic carbenes (NHCs) the archetypal benzoin and Stetter reactions are historically significant and synthetically versatile.^1,2^ Their demonstration and synthetic understanding have led to the diversity of NHC-promoted organocatalytic transformations that are available to the modern synthetic community. Central to both the benzoin and Stetter reactions is the catalytically competent “Breslow intermediate” (BI)^3^ that is regarded as the cornerstone of modern NHC-mediated catalysis. First characterised in 2012,^4^ its reactivity is key to a multitude of synthetic transformations. Despite its recognised importance, a quantitative understanding of the behaviour of this transiently formed species has yet to be defined and is regarded as a significant challenge.

**Fig. 1:**
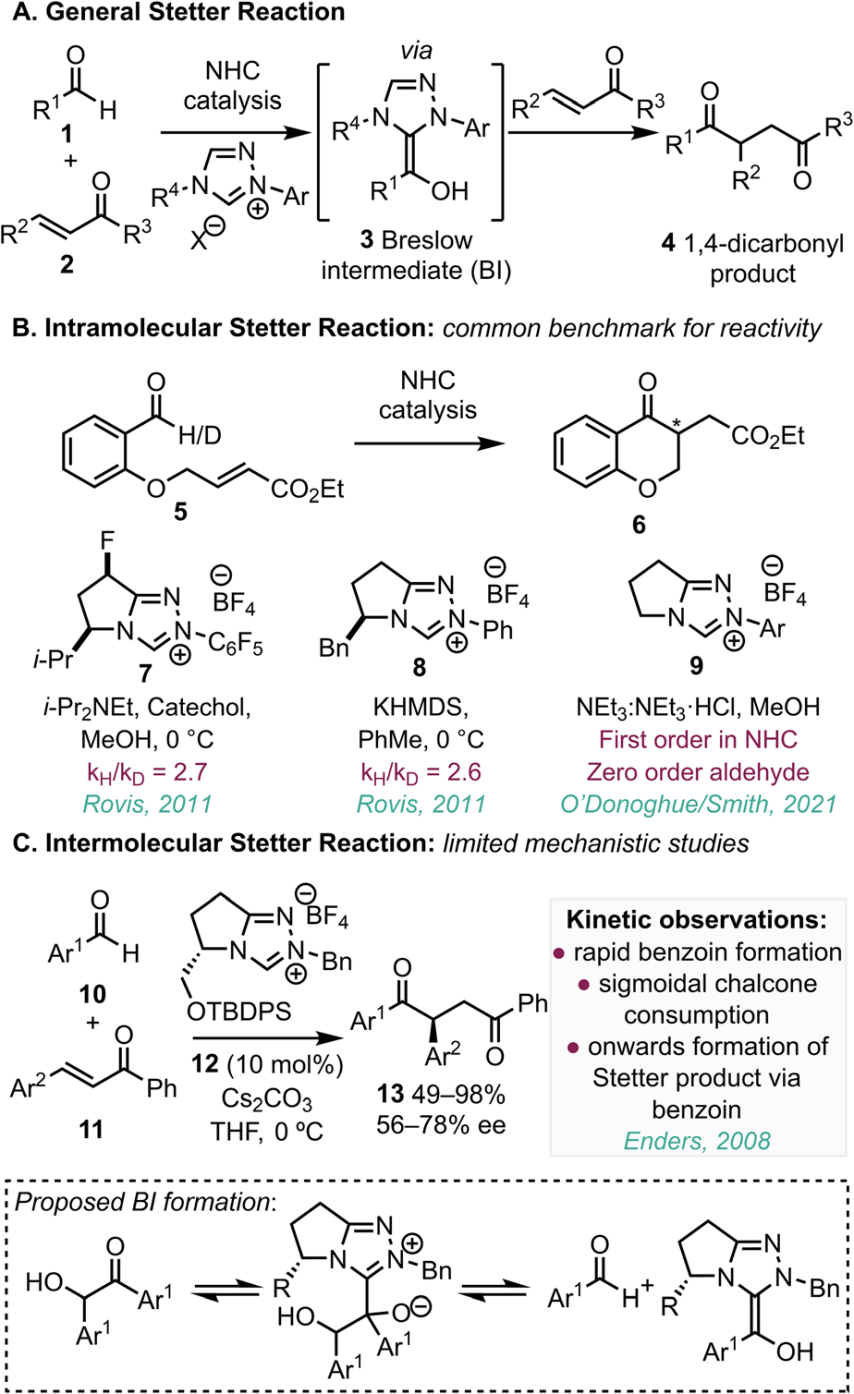
(a) The Stetter reaction; (b) mechanistic insight to the *intramolecular* Stetter reaction; (c) previous mechanistic insight to the *intermolecular* Stetter reaction.

Focusing upon the Stetter reaction, this process was first reported in 1973,^1*a*^ and allows the Umpolung coupling of an aldehyde with a Michael acceptor. This provides a potentially useful way to access 1,4-difunctionalised species of the general form 4 that are widely used as synthetic building blocks for heterocycle synthesis.^5^ This process is considered to proceed through *in situ* generation of a BI3 (derived from an NHC and an aldehyde 1), followed by addition to a Michael acceptor 2 and regeneration of the catalyst (Fig. 1a). Thiazolium, imidazolium and triazolium precursors to NHCs are most generally used, but metallophosphites^6^ and bisaminocyclopropenylidenes (BACs)^7^ have also been used as catalysts to access BI-type intermediates in Stetter-like reactions. In practice, the Stetter reaction remains a synthetic challenge due to competitive benzoin formation while factors that control chemoselectivity are not well understood. A deeper mechanistic understanding of the Stetter reaction could facilitate more selective synthetic methods while generating significant insight into quantification of the reactivity of the BI.

Previous mechanistic investigations into the Stetter reaction have largely focussed on the *intramolecular* reaction of substrate 5 to give product 6 (Fig. 1b), which is well-used both for the benchmarking of new reaction conditions, and as a model mechanistic probe.^8^ For example, in 2011, reports from Rovis demonstrated that deprotonation to form the Breslow intermediate is turnover-limiting under two mechanistically different scenarios.^9,10^ In the first,^9^ in the presence of catechol and using MeOH as solvent, a primary kinetic isotope effect (*k*_H_/*k*_D_ = 2.7) was observed using precatalyst 7 implicating the proton transfer step to the BI as rate-limiting. Further studies using a chiral NHC derived from 8 in toluene showed that the reaction process was first-order in both NHC catalyst and aldehyde substrate, with *k*_H_/*k*_D_ = 2.6.^10^ These results were further corroborated by a kinetic study from our groups for reactions performed in buffered methanol.^11^ This study indicated the reaction to be first-order in NHC catalyst and zero-order in aldehyde over a broad range of aldehyde concentrations. The reaction rate was also higher for NHC precatalysts 9 with N-aryl substituents bearing electron-withdrawing units within the triazolium skeleton. This is consistent with deprotonation to form the Breslow intermediate being turnover-limiting in this reaction.

The (enantioselective) intermolecular Stetter reaction has been widely studied, yet only limited mechanistic studies of this process have been demonstrated despite the significant synthetic utility. In this context, in 2008, Enders^12^ reported an asymmetric intermolecular Stetter reaction catalysed by a triazolium salt 12-derived NHC, using a variety of substituted (*E*)-chalcones 11 and benzaldehydes 10 as starting materials, giving the desired products 13 in good yields (49–98%) and enantioselectivities (56–78% ee) (Fig. 1c). Kinetic analysis of this reaction process indicated rapid benzoin formation and subsequent sigmoidal consumption of chalcone and benzoin that correlated to the formation of the Stetter product. This led to the proposal that the aldehyde is initially converted rapidly to benzoin by the NHC. Onwards formation of the Stetter product was postulated following NHC addition to benzoin, with subsequent fragmentation leading to BI formation. This property has been used in a synthetic setting by You, who exploited the reversibility of the benzoin reaction for the irreversible addition of benzaldehyde to imines.^13^

**Fig. 2:**
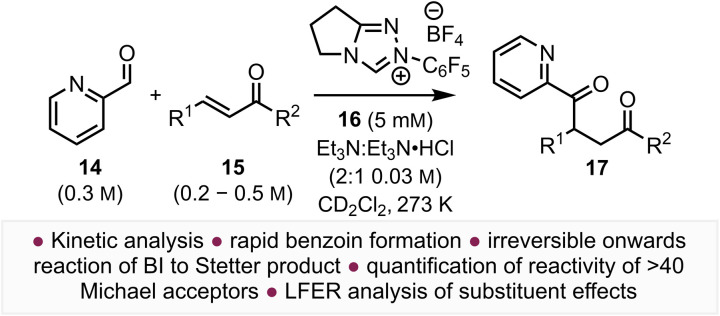
This work: quantifying the reactivity of the Breslow Intermediate with a range of Michael acceptors in the intermolecular Stetter reaction.

Significantly, despite widespread interest in the development of intermolecular Stetter reactions, the ability to quantify the reaction of presumed BIs with Michael acceptors remains a recognised untackled challenge, which is necessary to enable understanding and prediction of chemoselectivity. Herein we describe analysis of the reactivity of the BI derived from 2-pyridine carboxaldehyde 14 and an *in situ* generated N-pentafluorophenyl substituted triazolinylidene NHC derived from 16 that reacts with a wide range of Michael acceptors 15 to give products 17 (Fig. 2). This kinetic model enables quantification of Michael acceptor reactivity in the intermolecular Stetter reaction for the first time. This quantitative study (of BI reactivity with Michael acceptors) also revealed an additive substituent effect in chalcones through examining Hammett and Swain–Lupton linear free energy relationships, with DFT analysis offering insights into this intriguing phenomenon.

## 2 Results and discussion

### 2.1 Identification and validation of a kinetic model to quantify Breslow intermediate reactivity

**Fig. 3:**
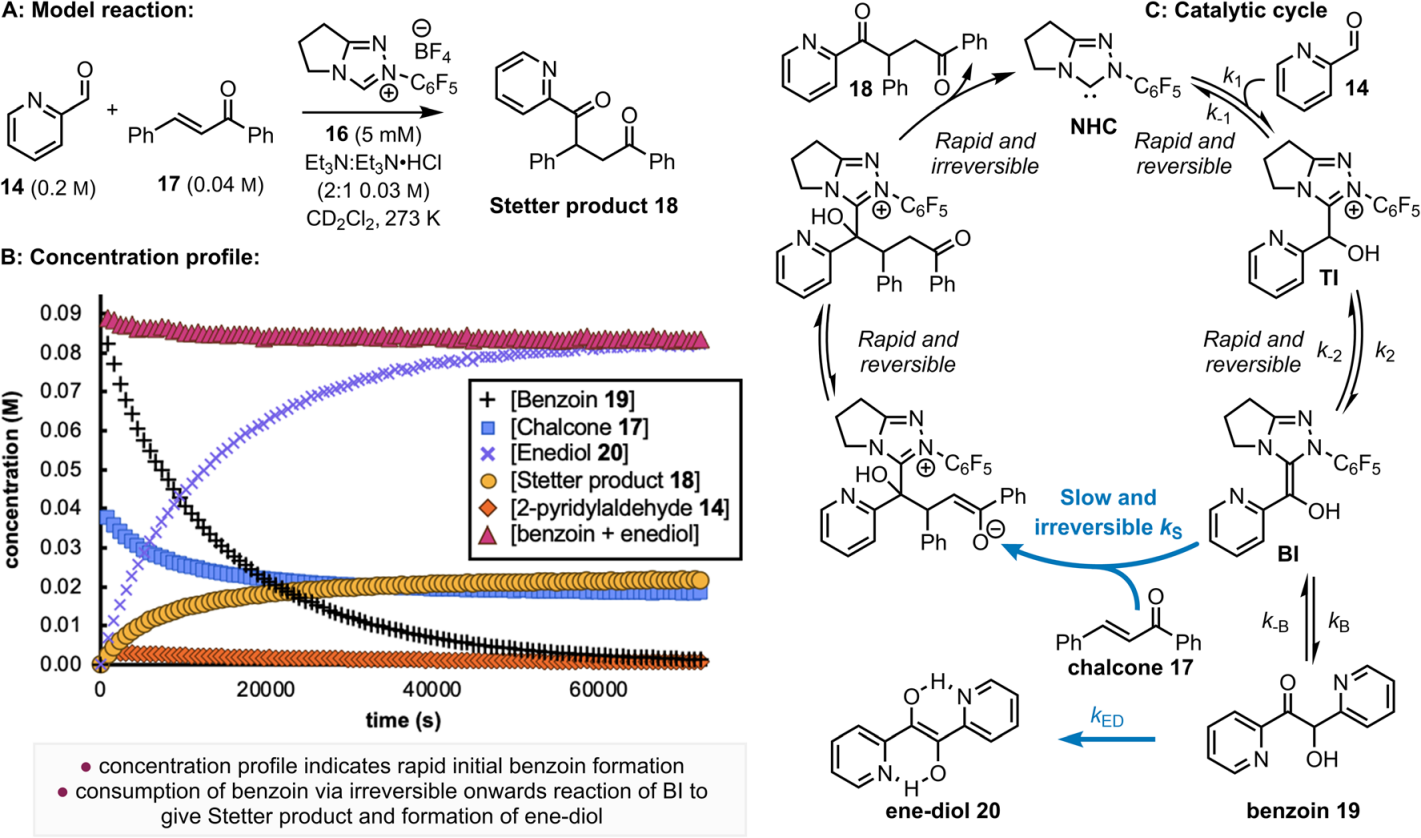
Concentration profile for the reaction between pyridine-2-carboxyaldehyde 14 (0.2 M) and (*E*)-chalcone 17 (0.04 M) catalysed by N–C_6_F_5_ triazolium derived catalyst 16 (5.0 m M) in NEt_3_ : NEt_3_·HCl (2 : 1, 0.03 M) in CD_2_Cl_2_ at 25 °C (A and B). For simplicity and improved visual clarity, data are shown for measurements at 720 s intervals however data were collected at 360 s intervals and are shown in full in the electronic supporting information. (C) The mechanism and catalytic cycle for the reaction of pyridine-2-carboxyaldehyde 14 and chalcone 17 catalysed by N–C_6_F_5_ triazolium derived catalyst 16.

An inherent problem in quantifying the reactivity of the BI intermediate within the intermolecular Stetter reaction is the difficulty in deconvoluting this process from the competitive benzoin reaction. In intramolecular Stetter processes, rapid intramolecular Michael addition of an assumed BI substantially outcompetes the benzoin reaction. However, in an intermolecular process, the reaction of the BI with the aldehyde starting material is normally competitive with 1,4-addition of the Stetter reaction. To mitigate the influence of the benzoin reaction, initial studies sought to increase the disparity of reaction rates between the Stetter and benzoin reactions, minimizing their interference. Building upon our previous work, heteroaromatic aldehydes exhibit exceptionally high reactivity with NHCs. Specifically, the equilibrium constant for TI formation increases with the introduction of 2,6-substitution on the N-aryl substituent of the triazolium salt, while N–C_6_F_5_ substitution leads to rapid benzoin product formation.^14^ With this concept in mind, highly reactive aldehydes and NHC catalysts were selected to expedite the reversible benzoin reaction, with the productive Stetter reaction postulated to proceed at a reduced rate that could be controlled using a suitable Michael acceptor. Pyridine-2-carboxyaldehyde 14 (0.2 M) and N–C_6_F_5_ triazolium salt 16 (5 mM) led to an exceptionally fast benzoin reaction^15^ and were chosen as the substrate and catalyst for these experiments, with (*E*)-chalcone 17 (0.04 M) used as the Michael acceptor (Fig. 3A). *In situ* reaction monitoring by ^1^H NMR spectroscopy allowed a concentration profile (Fig. 3B) of the reaction to be constructed. Consistent with our previous studies, the benzoin reaction exhibits a remarkably fast reaction rate, with 0.18 M of pyridine-2-carboxyaldehyde 14 rapidly and reversibly transformed into the corresponding benzoin product (∼0.09 M) 19 within seconds. Slow consumption of all benzoin product to zero then follows to give Stetter product 18 (∼0.02 M) that corresponds to the consumption of chalcone 17 (from 0.04 M to ∼0.02 M), and enediol 20 (∼0.08 M). The formation of enediol-type products from benzoin derivatives is well-documented under basic conditions,^15^ while the irreversibility of enediol and Stetter products were each confirmed experimentally by control reactions (see SI).^16^ Onwards and irreversible reaction of the BI with (*E*)-chalcone 17 is presumed to be the rate-determining step for the intermolecular Stetter reaction with the catalytic cycle illustrated in Fig. 3C.

Under these conditions, the initial concentration of pyridine-2-carboxyaldehyde 14 is much larger than the initial concentration ([Cat]_0_) of N–C_6_F_5_ triazolium precatalyst 16 ([aldehyde]_0_ >> [Cat]_0_). Although present at concentrations below the NMR detection limit, [BI] can be considered constant at the beginning of the reaction when [Ald]_0_ >> [NHC] as TI is in fast equilibrium with benzoin product 19, presumably *via*BI (see SI for details). The buffered conditions with NEt_3_·HCl/NEt_3_ in excess facilitates reversible deprotonation (*k*_2_) and reprotonation (*k*_−2_) steps between TI and BI. Given the intermolecular Michael addition of BI to the chalcone is expected to be the rate-determining step in the Stetter reaction, this allows the rate equation to be expressed using eqn (1) (where [BI] is the concentration of BI and [Ch] is the concentration of (*E*)-chalcone 17). As [BI] is constant, it can be incorporated into the pseudo first-order rate constant 
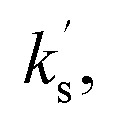
 shown in eqn (2). Using an initial rates method, the concentration of (*E*)-chalcone 17 ([Ch]) is approximated as the initial concentration ([Ch]_0_) and eqn (2) can be rewritten as eqn (3). A value of 
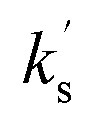
 can be calculated using the measured initial rate (*v*_max_) and the known [Ch]_0_.1
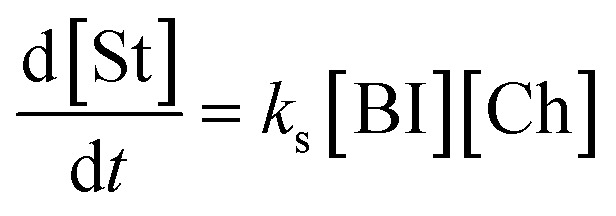
2
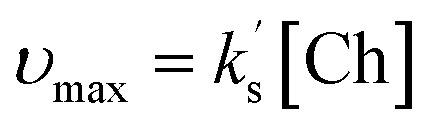
3
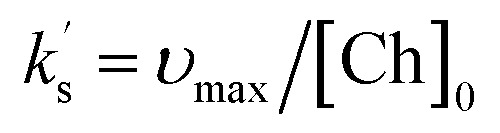


**Fig. 4:**
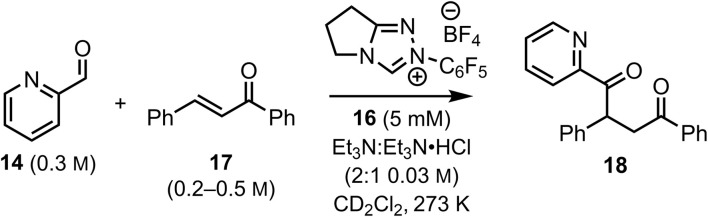
Individual rate constants (*k*_s_) and average rate constants 
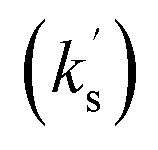
 for Stetter reactions of pyridine-2-carboxyaldehyde 14 (0.3 m) with different initial concentrations of (*E*)-chalcone 17 (0.2 m, 0.3 m, 0.4 m and 0.5 m).

To validate this hypothesis, a series of initial rate experiments were performed at different chalcone concentrations (from 0.2 to 0.5 M) to determine 
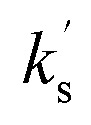
 using eqn (3). Four closely similar pseudo first-order rate constants 
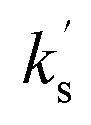
 (7.15, 7.10, 7.13 and 7.20 × 10^−5^ s^−1^) were obtained (Fig. 4), confirming that eqn (3) is a suitable description of the reaction and that BI addition to the chalcone 17 is turnover-limiting at low conversion. An average value of these rate constants, 
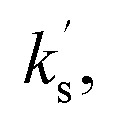
 was then calculated. Having validated this method, it was next applied to quantify the reactivity of a series of Michael acceptors reacting with the BI under these reaction conditions.

### 2.2 Application of kinetic analysis to quantifying BI reactivity with a range of Michael acceptors

**Fig. 5:**
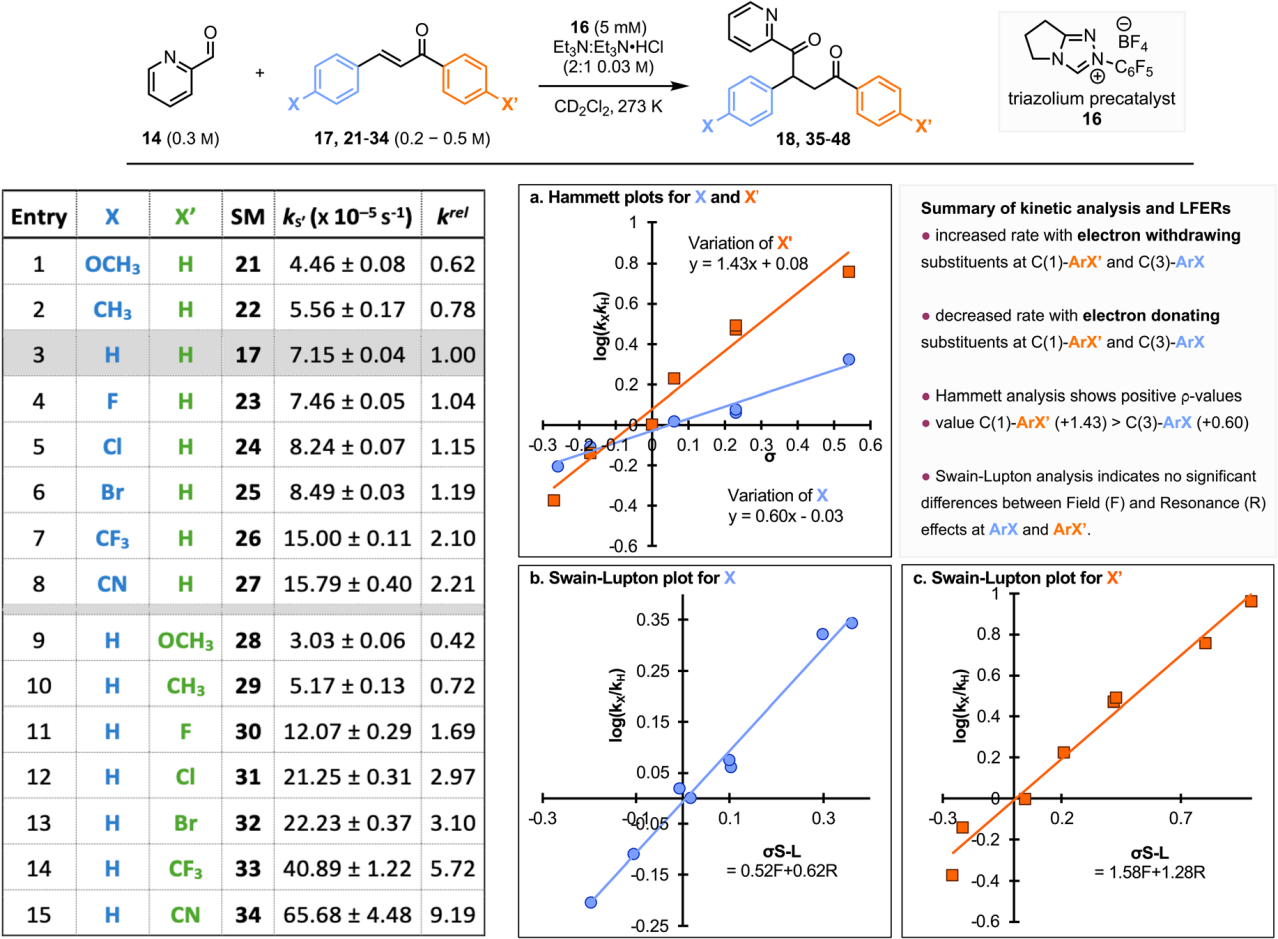
Rate constants 
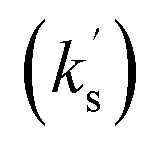
 and LFER analysis for the reaction of chalcone 17 and monosubstituted chalcones (21–34) with the BI derived from the addition of N–C_6_F_5_ triazolium catalyst 16 to pyridine-2-carboxaldehyde 14 at 25 °C. Entry 3 chalcone – highlighted in grey-was taken as a standard to calculate relative rates (*k*^rel^).

Further work aimed to quantify the electronic effect of substituent variation within the chalcone motif upon the initial rate of reaction, alongside assessing the reactivity of alternative classes of Michael acceptor (MA). First, a range of chalcone derivatives were prepared that incorporated electron donating and electron withdrawing substituents at the *para*-position within each aryl unit: C(1)–ArX′ and C(3)–ArX (Fig. 5). Authentic samples of each chalcone and its respective Stetter product were prepared (see SI for further information).^17^ Each chalcone was then subjected to the validated test conditions, and their initial rates were measured at 3 or 4 different initial concentrations ([MA]_0_). This ensures the assumption that [MA] is not changing during the initial stages of the reaction and can be approximated as ([MA]_0_) is maintained across the range of substituents tested. The average value of pseudo first-order rate constants, obtained from experiments conducted at various initial concentrations of the Michael acceptors ([MA]_0_), is given as the rate constant, 
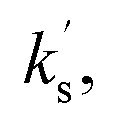
 of their reactions with the BI, allowing quantification of their reactivity.

#### 2.2.1 Effect of 4-substituent on aryl units (C(1)–ArX′ and C(3)–ArX) of (*E*)-chalcones

The pseudo first-order rate constants 
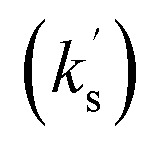
 for the reaction of mono-substituted chalcones with the BI derived from the addition of N–C_6_F_5_ triazolium precatalyst 16 to pyridine-2-carboxaldehyde 14 are summarized in Fig. 5. A clear trend in the rate constant with variation in substituent is observed. Notably, an increase in rate constant is observed for (*E*)-chalcones bearing electron-withdrawing substituents in the *para*-position of either substituent (C(1)–ArX′ and C(3)–ArX). Conversely, a decrease in the rate constant was observed when electron-donating substituents were present. Using the reactivity of the parent chalcone 17 as a standard, the relative rates (*k*^rel^) of each Michael acceptor can be quantified (for 17*k*^rel^ = 1.00). Notably, *k*^rel^ values of (*E*)-chalcones with electron-withdrawing substituents (Cl, Br, CF_3_ and CN) are larger than one (Table 1, entries 4–8 and 11–15), suggesting higher reactivity toward the BI than the parent (*E*)-chalcone. For (*E*)-chalcones with electron-donating substituents (Me and OMe), relative rates (*k*^rel^) below unity were obtained (Table 1, entries 1–2 and 9–10), consistent with the observations from both Ryu & Yang as well as Pacifico. ^18,19^ Comparing the relative rates (*k*^rel^) of (*E*)-chalcones with a given substituent upon either the C(3)-aromatic ring (ArX) or the C(1)-aromatic ring (ArX’) indicates their independent influence on the relative rate. For example, comparing 4-CN (*k*^rel^ = 2.21) and 4′-CN substituted chalcones (*k*^rel^ = 9.19) is indicative of the significant impact of C(1)Ar′ substitution (Table 1, entries 2 and 9). Similar trends are observed for other *para*-substituent chalcones, including those bearing electron-donating substituents (Me and OMe). Overall these trends indicate that the C(1)-aromatic group (ArX′) has a more significant electronic effect on the reactivity toward the presumed BI than the C(3)-aromatic group (ArX).

#### 2.2.2 Linear Free Energy Relationships^20^

In this study, the addition of BI to the chalcone is turnover-limiting allowing a linear free-energy relationship (LFER) to be established. Two standard Hammett plots were constructed to evaluate the effect of *para*-substitution on the C(1)-ArX′ and C(3)-ArX aromatic groups. Two positive reaction constants (*ρ*′ = 1.43 and *ρ* = 0.60, overlaid in Fig. 5a) were obtained. This is consistent with a build-up of negative charge in the chalcone component during the turnover-limiting step, aligning with the commonly accepted ionic Stetter reaction mechanism. Notably the significantly different magnitudes of the experimental *ρ*-values confirms that *para*-substitution on the C(1)–ArX′ has a more significant electronic effect than C(3)–ArX on the stability of the transition state associated with the turnover limiting step in the Stetter reaction. Swain–Lupton analysis (Fig. 5b and c) revealed that the relative contributions of Field (*F*) and Resonance (*R*) effects do not differ significantly between C(3)–ArX and C(1)–ArX′.

#### 2.2.3 An Additive Effect of 4,4′-Disubstitution of (*E*)-Chalcones

**Fig. 6:**
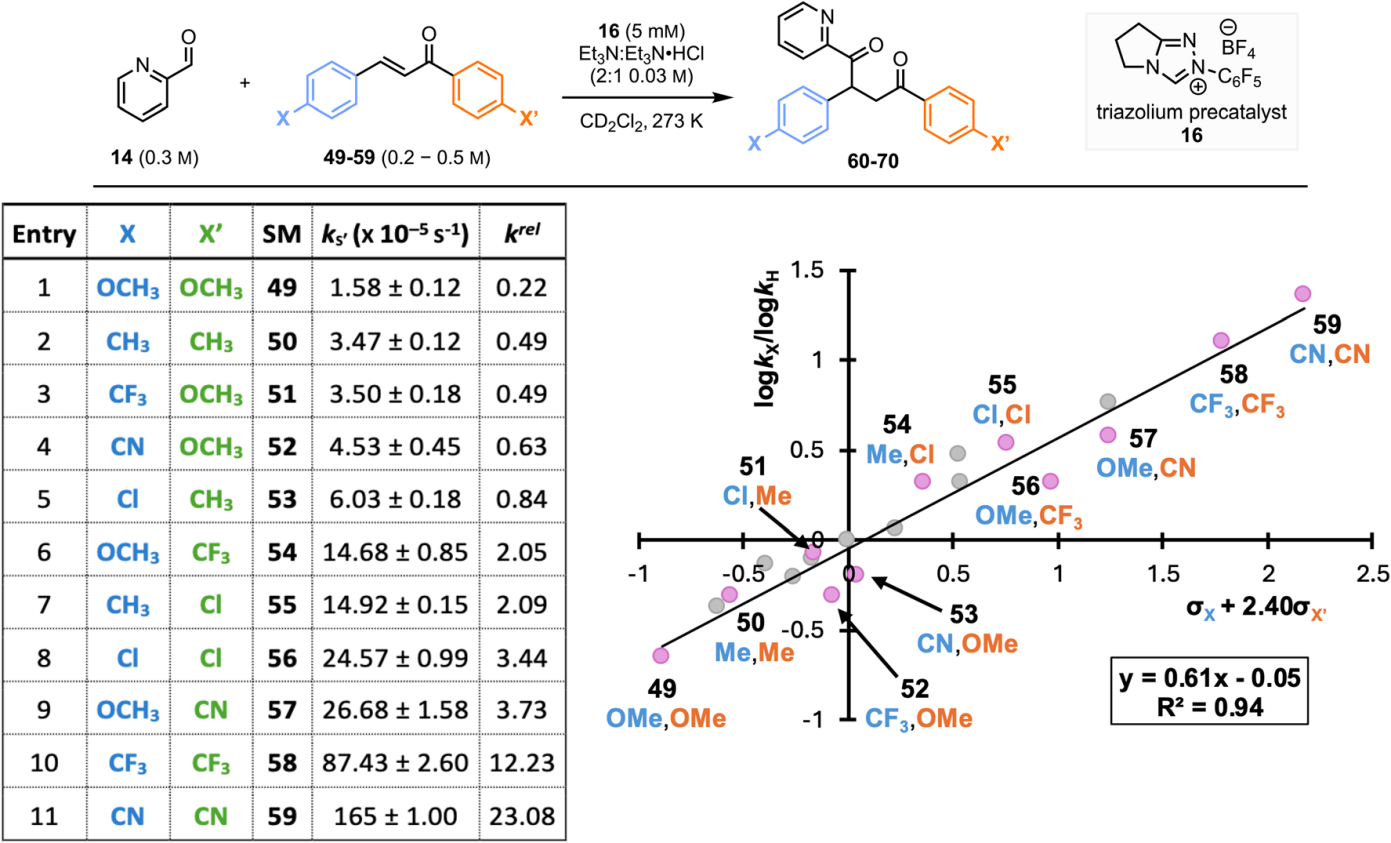
Rate constants 
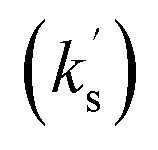
 and modified Hammett plot for the reaction of disubstituted chalcones (49–59) with the BI derived from the addition of N–C_6_F_5_ triazolium catalyst 16 to pyridine-2-carboxaldehyde 14 at 25 °C. Note: modified Hammett plot includes data for monosubstituted/unsubstituted chalcones (shown in grey) and disubstituted chalcones (shown in pink).

Building on this observation, the effect of including the same set of substituents upon both C(1)–ArX′ and C(3)–ArX within the chalcones was investigated (Fig. 6). Rate constants 
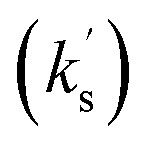
 for the reaction of 4,4′-disubstituted chalcones (49–59) with BI are summarised in Fig. 6 (ordered with increasing rate constants). The incorporation of substituents within both C(1)–ArX′ and C(3)–ArX has an additive effect upon the observed reaction rate constant, with the proportional contribution reflecting the relative *ρ* values obtained in the Hammett studies of ArX or ArX′. For example, 4,4′-dimethyl chalcone 50, exhibits a larger decrease in rate constant (
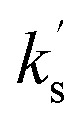
 = 3.47 × 10^−5^ s^−1^, entry 2) than 4-methyl or 4′-methyl chalcones (4-Me, 22, 
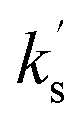
 = 5.56 × 10^−5^ s^−1^; 4′-Me, 29, 
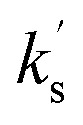
 = 5.17 × 10^−5^ s^−1^, Fig. 5, entries 2 and 10). Similarly 4,4′-bis(trifluoromethyl) chalcone (58, 
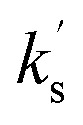
 = 87.43 × 10^−5^ s^−1^, entry 10) demonstrates a significant increase in rate constant compared with the mono-substituted chalcones (4-CF_3_, 26, 
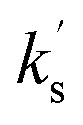
 = 15.00 × 10^−5^ s^−1^; 4′-CF_3_, 33, 
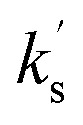
 = 40.89 × 10^−5^ s^−1^, Fig. 5 entries 7 and 14). Similar trends are also observed for 4,4′-dichloro, 4,4′-dimethoxy and 4,4′-dicyano chalcones (56, 58 and 59) when compared with the corresponding mono-substituted chalcones. When the C(1)–ArX′ and C(3)–ArX substituents are electronically distinct, the influence on rate constant is dominated by C(1)–ArX′. For example, comparing chalcones 51 and 54 bearing 4-OMe and 4-CF_3_ substituents, the chalcone 54 bearing an electron-withdrawing CF_3_ group on the 4′-position of C(1)–ArX′ undergoes reaction at a faster rate (Fig. 6 entries 3 and 6). This trend is maintained across all other tested disubstituted chalcones.

To probe the linear free-energy relationship for 4,4′-disubstituted chalcones, a method that allowed a single overall Hammett *σ*-value for disubstituted chalcones was developed. Using the ratio of reaction constants for the two series of mono-substituted chalcones (*ρ* = 0.60 and *ρ*′ = 1.43, Fig. 5a) as a correcting factor (2.40 = *ρ*′/ρ) a new Hammett plot with a modified *x*-axis (*σ*_*x*_ + 2.40*σ*_*x*′_) was prepared (Fig. 6B).^21^ This Hammett plot incorporates the electronic effect of disubstituted chalcones, giving a positive reaction constant (*ρ*_(4,4′)_ = 0.61, *R*^2^ = 0.94, Table 2) again aligning with the build-up of negative charge in the chalcone component during the turnover-limiting step of the Stetter reaction. The excellent linear correlation for all substituents (with no major outliers even when incorporating mono-substituted chalcones in grey) demonstrates that the aryl groups are independently influencing the observed reactivity, resulting in an additive substituent effect. Given the importance of nucleophilic addition to Michael acceptors across many synthetic transformations, the additive substituent effect observed for chalcones will likely have implications for understanding the reactivity of a range of alternative reaction processes.

**Fig. 7:**
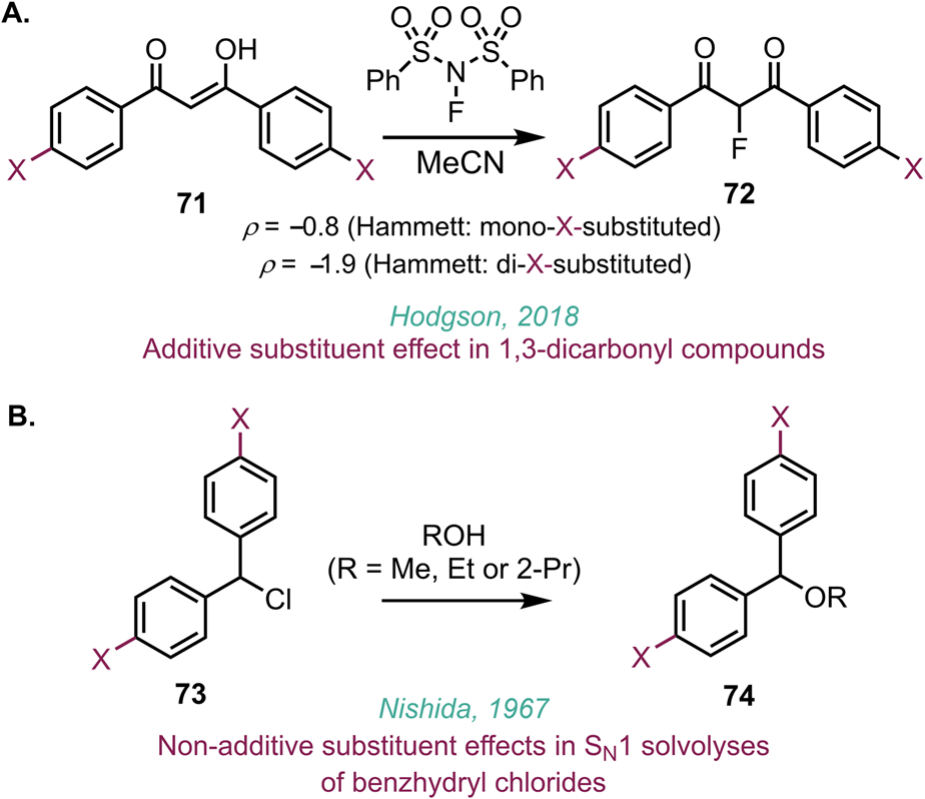
Selected literature examples considering additive Hammett effects.

To the best of our knowledge, there are few literature examples of reactions exhibiting this additive effect *across different aromatic substituents* although there are more examples of additive substituent effects within the same aryl ring. As an example, the fluorination of 1,3-diaryl-1,3-dicarbonyl derivatives 71 to give 72 was explored quantitatively by a range of electrophilic fluorinating agents (Fig. 7A).^22^ Although Swain–Lupton evaluation was not performed, the effects of mono- and di-aryl substitution were kinetically explored *via* Hammett analysis, with the *ρ*-value for di-substituted derivatives ∼2 times that observed for the mono-substituted analogues. Parallels may be drawn with *acceptor* behaviour of chalcones in the present study; the fluorination study explored a similar 1,3-unsaturated keto substrate but as *donor* nucleophiles rather than as acceptors. By contrast, the S_N_1 solvolysis of mono-and di-substituted diarylcarbinyl chlorides 73 in methanol, ethanol and 2-propanol solvents to give 74 did not show additivity for a broad range of substituents although additive effects were observed for a small subset of these substituents in a detailed Hammett analysis (Fig. 7B).^23^ By contrast with chalcone and dicarbonyl derivatives 72, the two aryl rings of diarylcarbinyl substrate 73 are attached to the *same carbon* thus preventing full conjugation of both rings simultaneously.

**Fig. 8:**
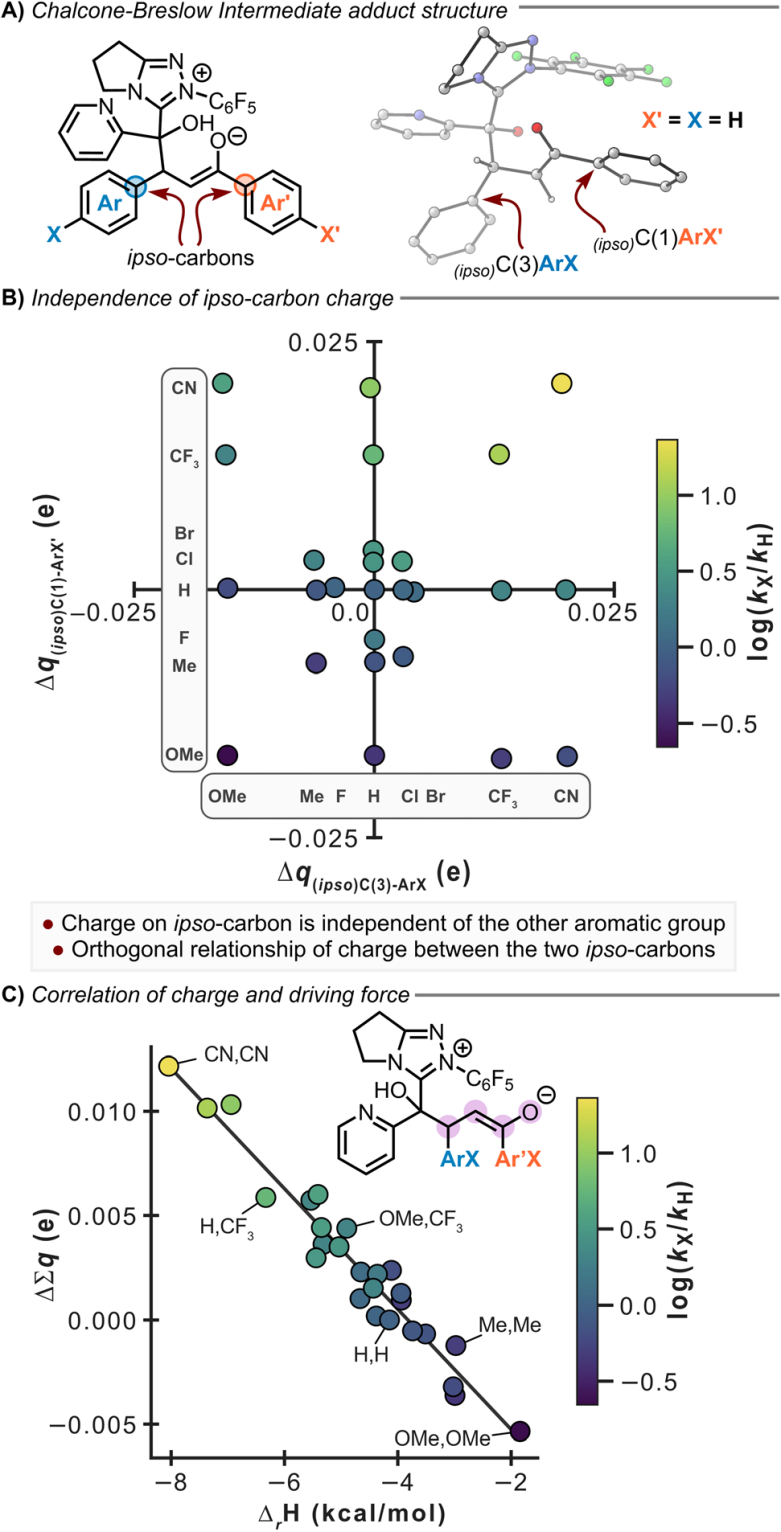
Computational analysis of Hirshfeld partial charges and correlation to the experimental log(*k*_X_/*k*_H_). (A) Structure of adduct formed from BI addition to chalcone. (B) Orthogonality of the effect of substitution relative to the parent unsubstituted system. (C) Correlations of the (relative) sum of partial charges to the computed driving force of the reaction.

Intrigued by the additive substituent effect, DFT calculations were performed to better understand these observations. Calculations were performed at the M06-2X_SMD(DCM)_/def2-TZVP//M06-2X_SMD(DCM)_/def2-SVP level of theory using Gaussian16 (ref. 24) on the proposed catalytic cycle with the parent chalcone 17 in Fig. 8. These calculations align well with the experimental concentration profile in Fig. 8A, with a facile formation of benzoin 19 as the kinetic product (Δ^‡^*G* = 13.5 kcal mol^−1^, Δ_*r*_G = −5.1 kcal mol^−1^) resulting from the addition of pyridine-2-carboxyaldehyde 14 to the BI (see full reaction profile in Fig. S86D). This is calculated to be favoured by Δ*Δ*^‡^*G* = 3.1 kcal mol^−1^ compared to the addition of the chalcone to the BI (Δ^‡^*G* = 16.6 kcal mol^−1^). From benzoin 19, the barrier to the chalcone addition is increased to Δ^‡^*G* = 20.6 kcal mol^−1^, leading to the thermodynamically favoured Stetter product (Δ_*r*_G = −12.6 kcal mol^−1^), which is 7.1 kcal mol^−1^ more stable than benzoin 19.

To further explore the effect of chalcone substitution, the barrier heights and driving forces for addition of the BI to the chalcone across the series of mono- and di-substituted chalcones were calculated. Chalcones containing EWGs exhibit stronger thermodynamic driving forces and generally lower kinetic barriers for the formation of the corresponding NHC-chalcone adduct derived from BI addition to the chalcone. The experimentally observed reaction rates correlate very well to the enthalpic driving force of the reaction (*R*^2^ = 0.93), with chalcones bearing electron-withdrawing substituents proceeding through a transition state with elongated C–C bond lengths in a more reactant-like transition state, consistent with the Hammond postulate (see Fig. S89). Attempts were then made to correlate charge distributions across the NHC–chalcone adduct derived from BI addition to the chalcone with Hammett parameters and to probe the experimentally observed additivity of Hammett parameters in the di-substituted chalcones.

Using an approach similar to Paton,^25^ the Hammett parameters correlated strongly to the Hirshfeld charge^26^ on the *ipso*-carbon of the mono-substituted chalcones (see Fig. S88). The charge on the *ipso*-carbon of either C(1)ArX′ or C(3)ArX is almost exclusively dependent on only the substituent on that specific ring. This is shown by the plot of Δ*q*_ipsoC(1)Ar*X*′_*vs.* Δ*q*_ipsoC(3)Ar*X*_ which demonstrates the orthogonality of the two charges, showing that the two properties are independent and exert separate effects on the reactivity (Fig. 8B). Considering the incorporation of OMe substitution for example, the introduction of this electron-donating group leads to a charge accumulation of −0.017 on each *ipso*-carbon compared to the unsubstituted chalcone, regardless of the substitution of the other ring. The effect of substitution can be combined into a single feature by considering the charges on the central portion of the chalcone (O, C(1), C(2) and C(3)). This feature shows strong correlation to the driving force, whereby positive charge accumulation (with EWGs) leads to a stronger driving force and faster rate (Fig. 8B). Substitution at C(1)-ArX′ has a larger influence on the charge (and hence the rate) than substitution of C(3)-ArX. For example, using enone 33 (C(3)ArX = PhH, C(1)ArX′ = 4-CF_3_C_6_H_4_) leads to a larger accumulation of positive charge (0.0059) than enone 26 (C(3)ArX = 4-CF_3_C_6_H_4_, C(1)ArX′ = PhH; 0.0036). The larger accumulation of positive charge is consistent with the build-up of negative charge being better stabilised in the reaction (Fig. 8C). This charge analysis is in excellent agreement with the experimental observations of the additive nature of Hammett parameters and is consistent across the mono- and di-substituted chalcones.

#### 2.2.4 Quantification of kinetic reactivity of further Michael acceptors

##### 2.2.4.1 C(3)-heterocyclic and C(3)-alkyl substituted enones

**Fig. 9:**
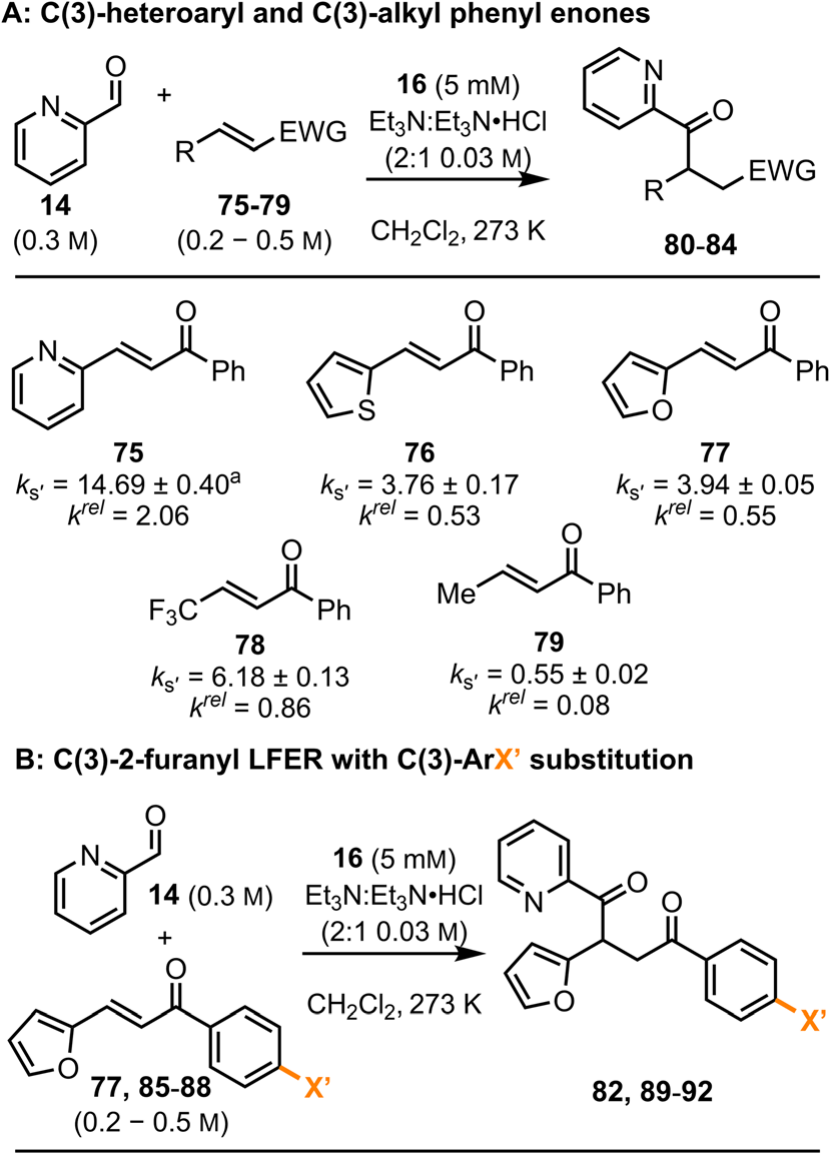
Rate constants 
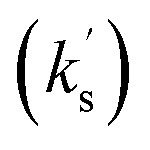
 for the reaction of other Michael acceptors (75–79, 85–88) with the BI derived from the addition of N–C_6_F_5_ triazolium catalyst 16 to pyridine-2-carboxaldehyde 14 at 25 °C. All rate constants shown in units ×10^−5^ s^−1^.

As a further extension of this model, it was applied to quantify the effect of heterocyclic substitution at C(3) *via* the reactivity C(3)-2-pyridyl, C(3)-2-furanyl and C(3)-2-thiophenyl enone acceptors 75–77 (Fig. 9). Among these substrates the observed rate constant 
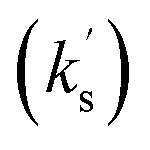
 of the 2-pyridyl enone 75 is the largest (14.69 × 10^−5^ s^−1^) with *k*^rel^ = 2.05 (to the parent chalcone). The 2-thiophenyl enone 76 and 2-furanyl enone 77 show similar rate constants (3.76 × 10^−5^ s^−1^ and 3.94 × 10^−5^ s^−1^, respectively), with *k*^rel^ = 0.52 and 0.55 respectively. The reactivity of C(3)-trifluoromethyl enone 78 and C(3)-methyl enone 79 were also evaluated, with the rate constant for 78 bearing the electron withdrawing trifluoromethyl substituent larger than that for the methyl enone 79 (6.18 × 10^−5^ s^−1^ and 0.55 × 10^−5^ s^−1^ respectively), giving *k*^rel^ = 0.86 and 0.08 respectively. To further explore if the previously observed electronic effect of C(1)–ArX′ substituents extended to heterocyclic systems, the effect of variation of the C(1)–ArX′ substituent within a series of C(3)-2-furanyl enones (77, 80–83) was evaluated. Consistent with previous observations, the inclusion of *p*-halogen substitution within C(1)-ArX′ led to increased rates of reaction with respect to the parent C(3)-2-furanyl enone 77, while the incorporation of a *p*-methyl substituent led to reduced reaction rates. Hammett analysis of this series indicated a positive *ρ*′ value of +1.65 consistent with the expected build-up of negative charge (see SI for further information).

##### 2.2.4.2 Alternative Michael acceptors

**Fig. 10:**
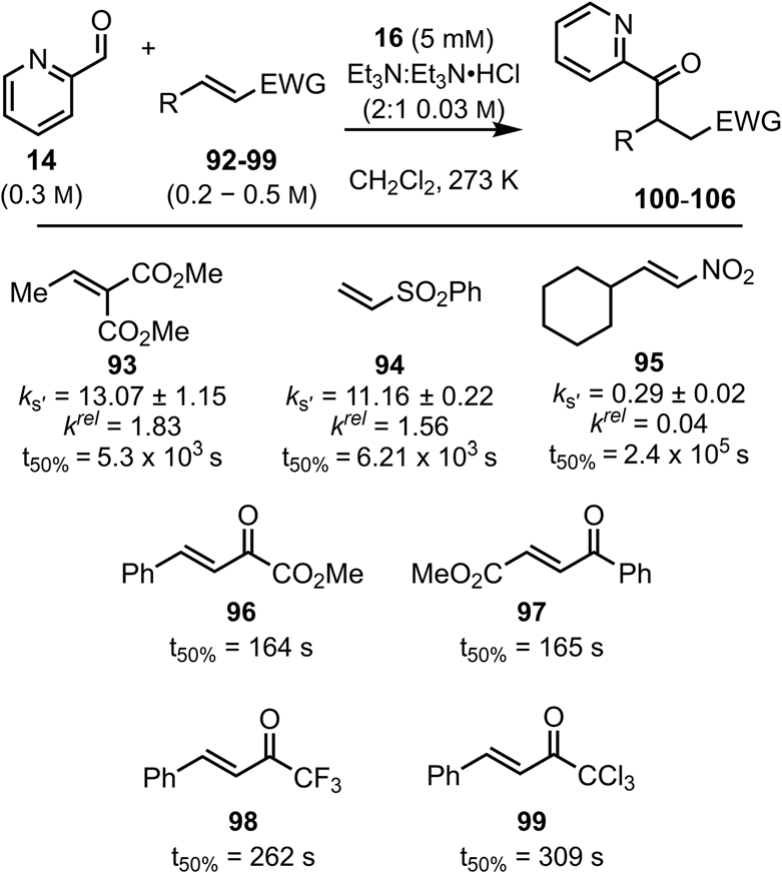
Rate constants 
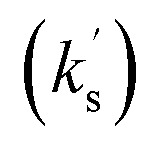
 for the reaction of other Michael acceptors (93–99) with the BI derived from the addition of N–C_6_F_5_ triazolium catalyst 16 to pyridine-2-carboxaldehyde 14 at 25 °C. All rate constants shown in units ×10^−5^ s^−1^. *t*_50%_ values shown in seconds.

The model was next applied to another distinct set of Michael acceptors incorporating a nitroolefin, diester, sulfone and enones containing strong electron withdrawing ester or perhalogenated substituents (Fig. 10). Dimethyl 2-ethylidenemalonate 93 and (vinylsulfonyl)benzene 94 showed high reactivity with relatively large rate constants observed (
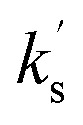
 = 13.07 × 10^−5^ s^−1^ and 
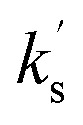
 = 11.16 × 10^−5^ s^−1^, respectively). However, (*E*)-(2-nitrovinyl)cyclohexane 95 exhibited a small rate constant (
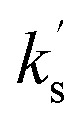
 = 0.29 × 10^−5^ s^−1^) implying low reactivity toward the BI. This model is not suitable for quantification of the reactivity of Michael acceptors (96–99) that bear strongly electron withdrawing substituents as they exhibit exceptionally high reactivity toward the BI. Using these substrates under the standardised conditions led to reactions that proceed to high conversion exceptionally quickly that did not allow initial rates to be calculated (see SI). However, the time taken to reach 50% conversion (*t*_50%_) was extracted from the concentration profile for these Michael acceptors, affording an approximation of their relative reactivities. This analysis reveals that methyl (*E*)-2-oxo-4-phenylbut-3-enoate 96 and methyl (*E*)-4-oxo-4-phenylbut-2-enoate 97 show similarly high reactivity, larger than the Michael acceptors 98 and 99. The result also gives a potential explanation why only an *α*-ketoester derived Stetter product was observed when competing with (*E*)-chalcones in Gravel's reported work^27^ as this is clearly significantly more reactive.

## 3 Conclusions

**Fig. 11:**
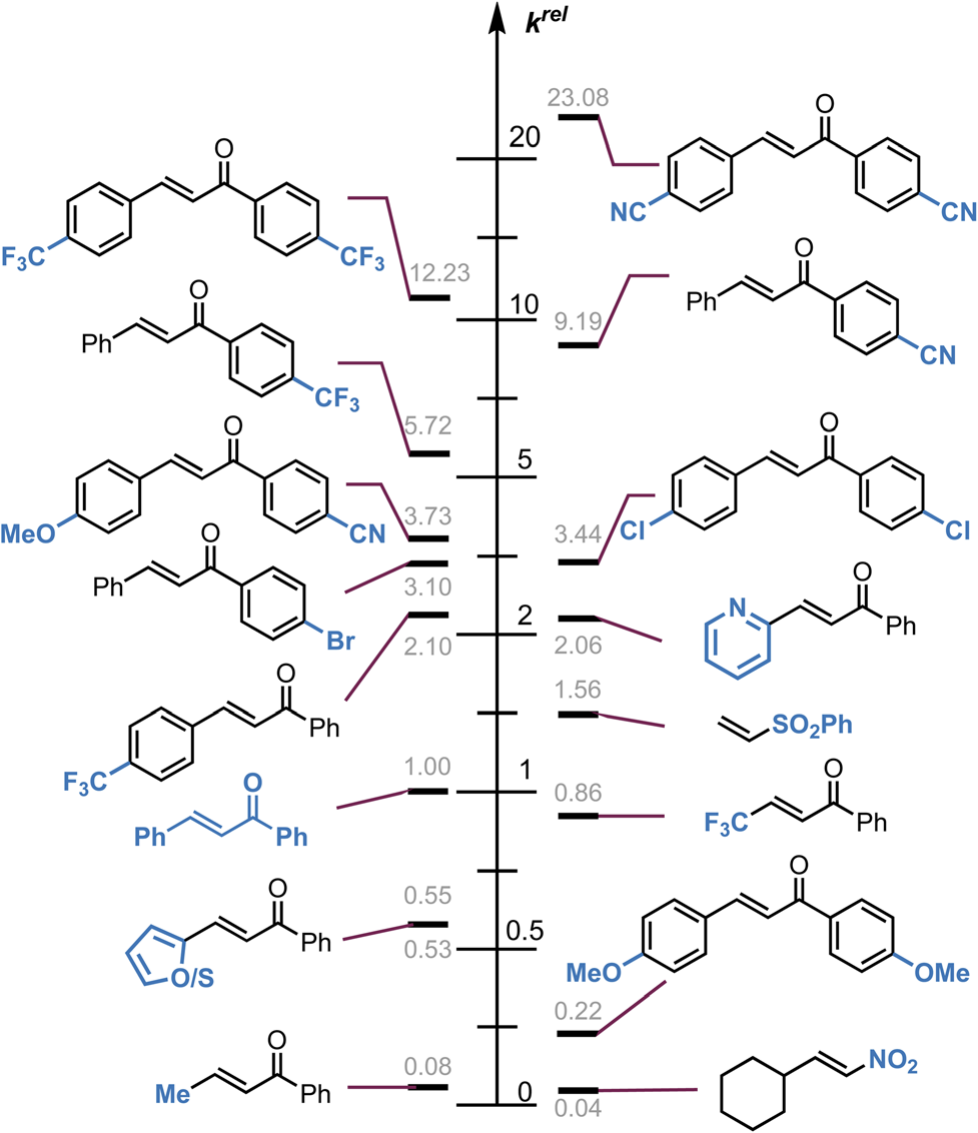
Pictorial representation of selected relative rates (*k*^rel^) for the reaction of selected Michael acceptors with the BI derived from the addition of N–C_6_F_5_ triazolium catalyst 16 to pyridine-2-carboxaldehyde 14 at 25 °C.

In this study, a kinetic method that allows for the precise quantification of the reactivity exhibited by a variety of Michael acceptors when they engage with a Breslow intermediate (BI) derived from pyridine-2-carboxaldehyde 14 and the NHC derived from an NC_6_F_5_ substituted triazolium precatalyst 16 is described. Measurement and analysis of pseudo first-order rate constants for a set of forty-three Michael acceptors is reported using this analysis allowing quantification of the reactivity of a common Breslow intermediate. Key findings demonstrate that the introduction of electron-withdrawing groups at the *para*-positions of C(1)–ArX′ and C(3)–ArX significantly augment the reactivity of chalcones in the Stetter reaction. Notably, C(1)–ArX′ substitution is exceptionally sensitive to electronic modification, resulting in a substantial enhancement of reactivity as pictorially represented in Fig. 11. Notably, the reactivity of the Michael acceptors observed in this process (across chalcones, alkylidene malonates, vinylsulfones and nitrostyrenes) correlate well with the established Mayr electrophilicity (*E*) reactivity scale.^28^ The positive reaction constants (*ρ*) obtained provide evidence for the buildup of negative charge within the chalcone component during the rate-limiting step of the studied process in agreement with the widely accepted ionic mechanism of the Stetter reaction. Furthermore, an additive effect associated with the 4,4′-disubstitution of C(1)–ArX′ and C(3)–ArX within chalcones has been reported. We present our preliminary investigations of this effect, which is generally relevant to the widespread examples of synthetic reactions involving chalcones as Michael acceptors.

## Author contributions

Zhuan Duan – conceptualization, investigation, writing – original draft. Claire M. Young – formal analysis, project administration, writing – original draft. Alister S. Goodfellow – formal analysis, investigation, writing – review and editing. Jiayun Zhu – formal analysis, conceptualization. Pankaj K. Majhi – supervision and analysis. AnnMarie C. O'Donoghue – conceptualization, funding acquisition, project administration, writing – review and editing. Andrew D. Smith – conceptualization, funding acquisition, project administration, writing – review and editing.

## Conflicts of interest

There are no conflicts to declare.

## Supplementary Material

SC-016-D5SC05021A-s001

## Data Availability

The research data supporting this publication can be accessed at https://doi.org/10.17630/966c2f26-72a5-4a42-9c4d-9eb42eadff04: data underpinning “Quantifying Breslow Intermediate Reactivity in Intermolecular Stetter Reactions”. University of St Andrews Research Portal; PURE ID: 320253920. Supplementary information is available. See DOI: https://doi.org/10.1039/d5sc05021a.
