# Supplementary material for: Quantifying Breslow intermediate reactivity in intermolecular Stetter reactions
Source: Chem Sci. 2025 Aug 22;16(45):21614–23. doi: 10.1039/d5sc05021a (PMC12530804; doi:10.1039/d5sc05021a)
Supplement: SC-016-D5SC05021A-s001 [file SC-016-D5SC05021A-s001.pdf]

Supplementary Information (SI) for Chemical Science.
This journal is © The Royal Society of Chemistry 2025

Supporting Information

Quantifying Breslow Intermediate Reactivity in Intermolecular Stetter Reactions

Zhuan Duan,[a] Jiayun Zhu,[b] Pankaj K. Majhi,[a] Alister S. Goodfellow,[a*] AnnMarie C. O'Donoghue,[b*] Claire M. Young,[a*] and Andrew D. Smith[a*]

[a] School of Chemistry, University of St Andrews, North Haugh, St Andrews KY16 9ST, UK
E-mail: ads10@st-andrews.ac.uk
[b] Department of Chemistry, University Science Laboratories, South Road, Durham, DH1 3LE, UK
E-mail: annmarie.odonoghue@durham.ac.uk

Table of Contents

1. General Instrumentation 2
2. Synthesis of Michael Acceptors 3
3. Determination of Rate Constants (ks') for Stetter Product Formation 21
4. Synthesis of Stetter Products 172
5. Computational Details 194
6. Conformational Approach 195
7. Computed Reactivity 196
8. Chalcone Reactivity Trends 197
9. References 199
10. ^{1}H, C^{13}\{^{1}H\}, F^{19}\{^{1}H\} NMR Spectra 202
11. Computational Raw Data 267

1. General Instrumentation

All reactions to synthesize triazolium precatalysts were performed in flame-dried glassware under an N_2 atmosphere. Anhydrous CH_2Cl_2 was obtained from an MBraun SPS-800 system. All other solvents were used without further purification unless otherwise stated. All heterocyclic aldehydes were purified before being used. Room temperature (rt) refers to 20–25°C. All kinetic experiments were conducted under N_2.

Analytical thin layer chromatography was performed on pre-coated aluminium plates (Kieselgel 60 F_{254} silica). Aluminium plates were visualized under UV light (254 nm). Flash column chromatography was performed on Kieselgel 60 silica in the solvent system stated under a positive pressure of compressed air. Melting points were measured using an Electrothermal 9100 melting point apparatus.

Infrared spectra (ν_{max}) were recorded on a Shimadzu IRAffinity-1 Fourier transform IR spectrophotometer fitted with a Specac Quest ATR accessory (diamond puck) using either thin film or solid, and only characteristic absorption wavenumbers (ν_{max}) were reported.

NMR spectra were recorded on either a Bruker AV400 with a BBFO probe (^{1}H 400 MHz; $^{13}C\{^{1}H\}$ 101 MHz; $^{19}F\{^{1}H\}$ 377 MHz), a Bruker AVII 400 with a BBFO probe (^{1}H 400 MHz; $^{13}C\{^{1}H\}$ 101 MHz; $^{19}F\{^{1}H\}$ 376 MHz), a Bruker AVIII-HD 500 with a SmartProbe BBFO + probe (^{1}H 500 MHz, $^{13}C\{^{1}H\}$ 126 MHz, $^{19}F\{^{1}H\}$ 470 MHz), or a Bruker AVIII 500 with a CryoProbe Prodigy BBO probe (^{1}H 500 MHz, $^{13}C\{^{1}H\}$ 126 MHz, ^{19}F 470 MHz) in the deuterated solvent stated. All kinetic data was measured using a Bruker AVIII-HD 500 with a SmartProbe BBFO + probe (^{1}H 500 MHz, $^{13}C\{^{1}H\}$ 126 MHz, $^{19}F\{^{1}H\}$ 470 MHz). All chemical shifts are quoted in parts per million (ppm) relative to the residual solvent peak. All coupling constants (*J*) are quoted in Hz. Multiplicities are indicated as s (singlet), d (doublet), t (triplet), q (quartet), m (multiplet), and multiples thereof. The abbreviation Ar denotes aromatic and br denotes broad singlet. NMR peak assignments were confirmed using 2D ^{1}H correlated spectroscopy (COSY), 2D ^{1}H-^{13}C heteronuclear single quantum coherence (HSQC), 2D ^{1}H-^{13}C heteronuclear multiple-bond correlation spectroscopy (HMBC).

Mass spectrometry (m/z) data were acquired by electrospray ionisation (ES), and electron impact (EI) at University of St Andrews.

2. Synthesis of Michael Acceptors

General procedure A

Benzaldehyde (1.0 equiv.) was dissolved in EtOH, and a 3 M $NaOH_{aq}$ solution (3.0 equiv.) and acetophenone (1.0 equiv.) were added and the resulting mixture was stirred at room temperature for 2 h to form a white precipitate. The white solid was separated by vacuum filtration and was washed three times with 10 mL ice water. The dry crude product was purified by recrystallization from MeOH or silica gel column to yield the product.

(*E*)-3-(4-methoxylphenyl)-1-phenylprop-2-en-1-one 21

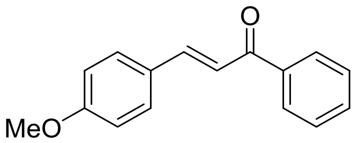

4-Methoxylbenzaldehyde (1.36 g, 10 mmol) was dissolved in EtOH (20 mL), and a 3 M $NaOH_{aq}$ solution (10 mL) and acetophenone (1.20 g, 10 mmol) were added and the resulting mixture was stirred at room temperature for overnight to form a white precipitate. The white solid was separated by vacuum filtration and was washed three times with 10 mL ice water. The dry crude product was purified by recrystallization from MeOH to yield the white titled product (1.95g, 82%). **mp**, 77-78 °C (literature: 77-78 °C[1]). **^{1}H NMR** (400 MHz, CD_2Cl_2) δ_H: 3.89 (3H, s, C*H₃*), 6.96-7.02 (2H, m, Ph*H*), 7.46-7.58 (3H, m, C*H* + Ph*H*), 7.61-7.69 (3H, m, Ph*H*), 7.82 (1H, d, *J* 15.6, C*H*), 8.04-8.07 (2H, m, Ph*H*). Spectra data in accordance with literature.[1]

(*E*)-3-(4-methylphenyl)-1-phenylprop-2-en-1-one 22

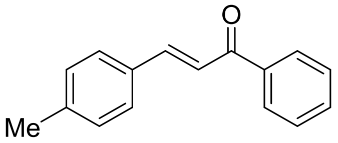

4-Methylbenzaldehyde (1.20 g, 10 mmol) was dissolved in EtOH (20 mL), and a 3 M $NaOH_{aq}$ solution (10 mL) and acetophenone (1.20 g, 10 mmol) were added and the resulting mixture was stirred at room temperature for overnight to form a white precipitate. The white solid was separated by vacuum filtration and was washed three times with 10 mL ice water. The dry crude product was purified by recrystallization from MeOH to yield the white titled product (1.69g, 76%). **mp**, 90-91 °C (literature: 94-95 °C[2]). **^{1}H NMR** (400 MHz, $CDCl_3$) δ_H: 2.42 (3H, s, C*H₃*), 7.25-7.28 (2.28H, m, Solvent + Ph*H*), 7.50-7.63 (6H, m, C*H* +

Ph*H*), 7.84 (1H, d, *J* 15.7, C*H*), 8.03-8.06 (2H, m, Ph*H*). Spectra data in accordance with literature.[2]

(*E*)-3-(4-fluorophenyl)-1-phenylprop-2-en-1-one 23

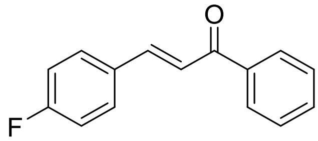

4-Fluorobenzaldehyde (1.24 g, 10 mmol) was dissolved in EtOH (20 mL), and a 3 M $NaOH_{aq}$ solution (10 mL) and acetophenone (1.20 g, 10 mmol) were added and the resulting mixture was stirred at room temperature for 2 h to form a white precipitate. The white solid was separated by vacuum filtration and was washed three times with 10 mL ice water. The dry crude product was purified by recrystallization from MeOH to yield the white titled product (1.51g, 67%). **mp**, 83-84 °C (literature: 83-85 °C[3]). **^{1}H NMR** (400 MHz, $CDCl_3$) δ_H: 7.11-7.18 (2H, m, Ph*H*), 7.51-7.56 (3H, m, C*H* + Ph*H*), 7.59-7.69 (3H, m, Ph*H*), 7.82 (1H, d, *J* 15.7, C*H*), 8.02-8.06 (2H, m, Ph*H*). Spectra data in accordance with literature.[3]

(*E*)-3-(4-chlorophenyl)-1-phenylprop-2-en-1-one 24

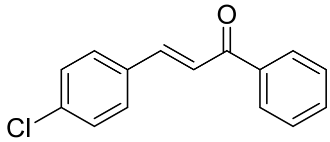

4-Chlorobenzaldehyde (1.40 g, 10 mmol) was dissolved in EtOH (20 mL), and a 3 M $NaOH_{aq}$ solution (10 mL) and acetophenone (1.20 g, 10 mmol) were added and the resulting mixture was stirred at room temperature for 2 h to form a white precipitate. The white solid was separated by vacuum filtration and was washed three times with 10 mL ice water. The dry crude product was purified by recrystallization from MeOH to yield the white titled product (1.91g, 79%). **mp**, 106-108 °C (literature: 106-108 °C[4]). **^{1}H NMR** (400 MHz, CD_2Cl_2) δ_H: 7.44-7.48 (2H, m, Ph*H*), 7.54-7.61 (3H, m, C*H* + Ph*H*), 7.63-7.69 (3H, m, Ph*H*), 7.80 (1H, d, *J* 15.7, C*H*), 8.04-8.07 (2H, m, Ph*H*). Spectra data in accordance with literature.[4]

(*E*)-3-(4-bromophenyl)-1-phenylprop-2-en-1-one 25

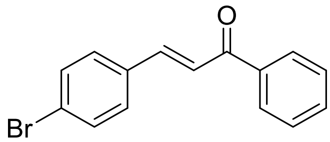

4-Bromobenzaldehyde (1.84 g, 10 mmol) was dissolved in EtOH (20 mL), and a 3 M $NaOH_{aq}$ solution (10 mL) and acetophenone (1.20 g, 10 mmol) were added and the resulting mixture was stirred at room

temperature for 2 h to form a white precipitate. The white solid was separated by vacuum filtration and was washed three times with 10 mL ice water. The dry crude product was purified by recrystallization from MeOH to yield the white titled product (2.32g, 81%). **mp**, 121-122 °C (literature: 121-123 °C[5]). **^{1}H NMR** (400 MHz, $CDCl_3$) δ_H: 7.51-7.65 (8H, m, C*H* + Ph*H*), 7.79 (1H, d, *J* 15.7, C*H*), 8.03-8.06 (2H, m, Ph*H*). Spectra data in accordance with literature.[5]

(*E*)-1-phenyl-3-(4-(trifluoromethyl)phenyl)prop-2-en-1-one 26

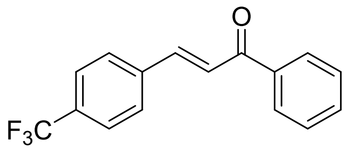

4-Trifluoromethylbenzaldehyde (1.74 g, 10 mmol) was dissolved in EtOH (20 mL), and a 3 M $NaOH_{aq}$ solution (10 mL) and acetophenone (1.20 g, 10 mmol) were added and the resulting mixture was stirred at room temperature for 1 h to form a white precipitate. The white solid was separated by vacuum filtration and was washed three times with 10 mL ice water. The dry crude product was purified by recrystallization from MeOH to yield the white titled product (2.04 g, 74%). **mp**, 119-120 °C (literature: 128-129 °C[6]). **^{1}H NMR** (400 MHz, $CDCl_3$) δ_H: 7.44-7.54 (4H, m, C*H* + Ph*H*), 7.66-7.70 (2H, m, Ph*H*), 7.79-7.81 (2H, m, Ph*H*), 7.84 (1H, d, *J* 15.8, C*H*), 8.12-8.14 (2H, m, Ph*H*). Spectra data in accordance with literature.[6]

(*E*)-4-(3-oxo-3-phenylprop-1-en-1-yl)benzonitrile 27

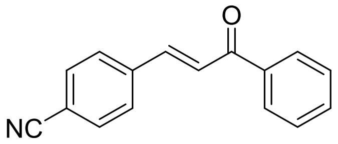

4-Formylbenzonitrile (1.31 g, 10 mmol) was dissolved in EtOH (20 mL), and a 3 M $NaOH_{aq}$ solution (10 mL) and acetophenone (1.20 g, 10 mmol) were added and the resulting mixture was stirred at room temperature for 1 h to form a white precipitate. The white solid was separated by vacuum filtration and was washed three times with 10 mL ice water. The dry crude product was purified by recrystallization from MeOH to yield the white titled product (2.07g, 89%). **mp**, 144-145 °C (literature: 146-149 °C[7]). **^{1}H NMR** (400 MHz, $CDCl_3$) δ_H: 7.53-7.57 (2H, m, C*H* + Ph*H*), 7.61-7.67 (2H, m, C*H* + 4-Ph*H*), 7.72-7.77 (4H, m, Ph*H*), 7.78 (1H, d, *J* 15.7, C*H*), 8.04-8.07 (2H, m, Ph*H*). Spectra data in accordance with literature.[7]

(*E*)-1-(4-methoxyphenyl)-3-phenylprop-2-en-1-one 28

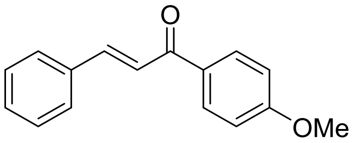

Benzaldehyde (1.06 g, 10 mmol) was dissolved in EtOH (20 mL), and a 3 M $NaOH_{aq}$ solution (10 mL) and 4′-methoxyacetophenone (1.50 g, 10 mmol) were added and the resulting mixture was stirred at room temperature for overnight to form a white precipitate. The white solid was separated by vacuum filtration and was washed three times with 10 mL ice water. The dry crude product was purified by recrystallization from MeOH to yield the white titled product (1.57g, 66%). **mp**, 95-96 °C (literature: 94-96 °C[8]). **^{1}H NMR** (400 MHz, $CDCl_3$) δ_H: 3.92 (3H, s, C*H*$_3$), 6.99-7.03 (2H, m, Ph*H*), 7.42-7.47 (3H, m, Ph*H*), 7.60 (1H, d, *J* 15.7, C*H*), 7.66-7.68 (2H, m, Ph*H*), 7.85 (1H, d, *J* 15.7, C*H*), 8.05-8.10 (2H, m, Ph*H*). Spectra data in accordance with literature.[8]

(*E*)-1-(4-methylphenyl)-3-phenylprop-2-en-1-one 29

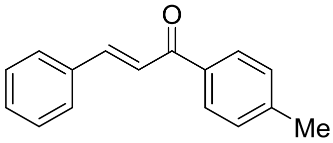

Benzaldehyde (1.06 g, 10 mmol) was dissolved in EtOH (20 mL), and a 3 M $NaOH_{aq}$ solution (10 mL) and 4′-methylacetophenone (1.34 g, 10 mmol) were added and the resulting mixture was stirred at room temperature for overnight to form a white precipitate. The white solid was separated by vacuum filtration and was washed three times with 10 mL ice water. The dry crude product was purified by recrystallization from MeOH to yield the white titled product (1.44g, 65%). **mp**, 63-65 °C (literature: 70 °C[9]). **^{1}H NMR** (400 MHz, $CDCl_3$) δ_H: 2.47 (3H, s, C*H*$_3$), 7.32-7.35 (2H, m, Ph*H*), 7.43-7.48 (3H, m, Ph*H*), 7.59 (1H, d, *J* 15.7, C*H*), 7.65-7.70 (2H, m, Ph*H*), 7.86 (1H, d, *J* 15.7, C*H*), 7.96-7.99 (2H, m, Ph*H*). Spectra data in accordance with literature.[4]

(*E*)-1-(4-fluorophenyl)-3-phenylprop-2-en-1-one 30

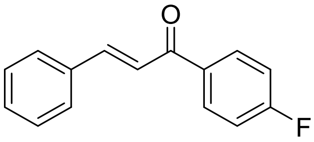

Benzaldehyde (1.06 g, 10 mmol) was dissolved in EtOH (20 mL), and a 3 M $NaOH_{aq}$ solution (10 mL) and 4′-fluoroacetophenone (1.38 g, 10 mmol) were added and the resulting mixture was stirred at room temperature for 2 h to form a white precipitate. The white solid was separated by vacuum filtration and was

washed three times with 10 mL ice water. The dry crude product was purified by recrystallization from MeOH to yield the white titled product (1.58g, 70%). **mp**, 78-80 °C (literature: 79-80 °C[10]). **^{1}H NMR** (400 MHz, $CDCl_3$) δ_H: 7.18-7.23 (2H, m, Ph*H*), 7.44-7.48 (3H, m, Ph*H*), 7.51 (1H, d, *J* 15.7, C*H*), 7.65-7.70 (2H, m, Ph*H*), 7.83 (1H, d, *J* 15.7, C*H*), 8.06-8.11 (2H, m, Ph*H*). Spectra data in accordance with literature.[10]

(*E*)-1-(4-chlorophenyl)-3-phenylprop-2-en-1-one 31

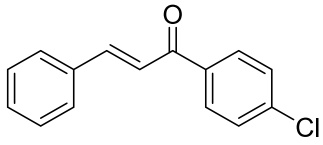

Benzaldehyde (1.06 g, 10 mmol) was dissolved in EtOH (20 mL), and a 3 M $NaOH_{aq}$ solution (10 mL) and 4′-chloroacetophenone (1.54 g, 10 mmol) were added and the resulting mixture was stirred at room temperature for 2 h to form a white precipitate. The white solid was separated by vacuum filtration and was washed three times with 10 mL ice water. The dry crude product was purified by recrystallization from MeOH to yield the white titled product (1.84g, 76%). **mp**, 112-113 °C (literature: 96-98 °C[11]). **^{1}H NMR** (400 MHz, CD_2Cl_2) δ_H: 7.46-7.50 (3H, m, Ph*H*), 7.53-7.59 (3H, m, C*H* + Ph*H*), 7.69-7.74 (2H, m, Ph*H*), 7.86 (1H, d, *J* 15.7, C*H*), 7.99-8.03 (2H, m, Ph*H*). Spectra data in accordance with literature.[11]

(*E*)-1-(4-bromophenyl)-3-phenylprop-2-en-1-one 32

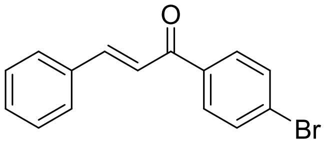

Benzaldehyde (1.06 g, 10 mmol) was dissolved in EtOH (20 mL), and a 3 M $NaOH_{aq}$ solution (10 mL) and 4′-bromoacetophenone (1.80 g, 10 mmol) were added and the resulting mixture was stirred at room temperature for 2 h to form a white precipitate. The white solid was separated by vacuum filtration and was washed three times with 10 mL ice water. The dry crude product was purified by recrystallization from MeOH to yield the white titled product (2.09g, 73%). **mp**, 93-95 °C (literature: 90-92 °C[8]). **^{1}H NMR** (400 MHz, $CDCl_3$) δ_H: 7.43-7.52 (4H, m, C*H* + Ph*H*), 7.64-7.70 (4H, m, Ph*H*), 7.82 (1H, d, *J* 15.7, C*H*), 7.89-7.94 (2H, m, Ph*H*). Spectra data in accordance with literature.[8]

(*E*)-1-(4-trifluoromethylphenyl)-3-phenylprop-2-en-1-one 33

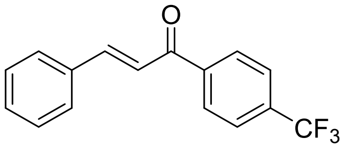

Benzaldehyde (1.06 g, 10 mmol) was dissolved in EtOH (20 mL), and a 3 M $NaOH_{aq}$ solution (10 mL) and 4′-trifluoromethylacetophenone (1.88 g, 10 mmol) were added and the resulting mixture was stirred at room temperature for 1 h to form a yellow precipitate. The yellow solid was separated by vacuum filtration and was washed three times with 10 mL ice water. The dry crude product was purified by recrystallization from MeOH to yield the yellow titled product (1.99g, 72%). **mp**, 116-117 °C (literature: 114-116 °C[8]). **^{1}H NMR** (400 MHz, $CDCl_3$) δ_H: 7.44-7.54 (4H, m, C*H* + Ph*H*), 7.66-7.71 (2H, m, Ph*H*), 7.79-7.81 (2H, m, Ph*H*), 7.88 (1H, d, *J* 15.8, C*H*), 8.10-8.17 (2H, m, Ph*H*). Spectra data in accordance with literature.[8]

4-Cinnamoylbenzonitrile 34

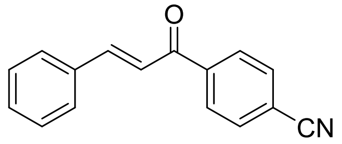

Benzaldehyde (1.06 g, 10 mmol) was dissolved in EtOH (20 mL), and a 3 M $NaOH_{aq}$ solution (10 mL) and 4-acetylbenzonitrile (1.45 g, 10 mmol) were added and the resulting mixture was stirred at room temperature for 1 h to form a yellow precipitate. The yellow solid was separated by vacuum filtration and was washed three times with 10 mL ice water. The dry crude product was purified by recrystallization from MeOH to yield the yellow titled product (1.68g, 72%). **mp**, 1108-109 °C (literature: 110-112 °C[12]). **^{1}H NMR** (400 MHz, $CDCl_3$) δ_H: 7.44-7.51 (4H, m, C*H* + Ph*H*), 7.65-7.71 (2H, m, Ph*H*), 7.82-7.88 (3H, m, C*H* + Ph*H*), 8.10-8.13 (2H, m, Ph*H*). Spectra data in accordance with literature.[12]

(*E*)-1,3-bis(4-methoxyphenyl)prop-2-en-1-one 49

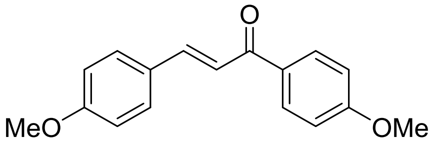

4-Methoxylbenzaldehyde (1.36 g, 10 mmol) was dissolved in EtOH (20 mL), and a 3 M $NaOH_{aq}$ solution (10 mL) and 4′-methoxyacetophenone (1.50 g, 10 mmol) were added and the resulting mixture was stirred at room temperature for 8 h to form a white precipitate. The white solid was separated by vacuum filtration and was washed three times with 10 mL ice water. The dry crude product was purified by recrystallization from MeOH to yield the white titled product (1.99 g, 65%). **mp**, 98-99 °C (literature: 102-105 °C[13]). **^{1}H**

NMR (400 MHz, $CDCl_3$) δ_H: 3.88 (3H, s, OC*H*$_3$), 3.91 (3H, s, OC*H*$_3$), 6.94-7.02 (4H, m, Ph*H*), 7.44 (1H, d, *J* 15.6, C*H*), 7.61-7.64 (2H, m, Ph*H*), 7.88 (1H, d, *J* 15.6, C*H*), 8.04-8.08 (2H, m, Ph*H*). Spectra data in accordance with literature.[14]

(*E*)-1,3-di-p-tolylprop-2-en-1-one 50

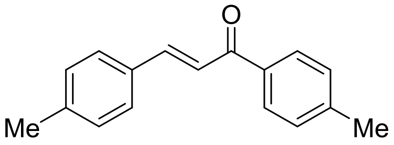

4-Methylbenzaldehyde (1.20 g, 10 mmol) was dissolved in EtOH (20 mL), and a 3 M $NaOH_{aq}$ solution (10 mL) and 4′-methylacetophenone (1.34 g, 10 mmol) were added and the resulting mixture was stirred at room temperature for 2 h to form a white precipitate. The white solid was separated by vacuum filtration and was washed three times with 10 mL ice water. The dry crude product was purified by recrystallization from MeOH to yield the white titled product (1.91g, 81%). **mp**, 123-125 °C (literature: 124-126 °C[15]). **^{1}H NMR** (400 MHz, $CDCl_3$) δ_H: 2.42 (3H, s, C*H*$_3$), 2.46 (3H, s, C*H*$_3$), 7.24-7.26 (2H, m, Ph*H*), 7.31-7.34 (2H, m, Ph*H*), 7.50-7.59 (3H, m, C*H* + Ph*H*), 7.83 (1H, d, *J* 15.7, C*H*), 7.94-7.98 (2H, m, Ph*H*). Spectra data in accordance with literature.[15]

(*E*)-1-(4-methoxyphenyl)-3-(4-(trifluoromethyl)phenyl)prop-2-en-1-one 51

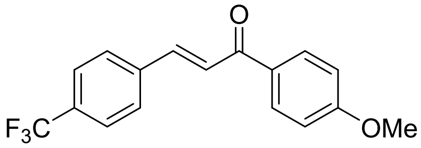

4-Trifluoromethylbenzaldehyde (1.74 g, 10 mmol) was dissolved in EtOH (20 mL), and a 3 M $NaOH_{aq}$ solution (10 mL) and 4′-methoxyacetophenone (1.50 g, 10 mmol) were added and the resulting mixture was stirred at room temperature for 2 h to form a light-yellow precipitate. The light yellow solid was separated by vacuum filtration and was washed three times with 10 mL ice water. The dry crude product was purified by recrystallization from MeOH to yield the light yellow titled product (2.23 g, 73%). **mp**, 127-128 °C (literature: 133.5-135 °C[16]). **^{1}H NMR** (400 MHz, $CDCl_3$) δ_H: 3.93 (3H, s, OC*H*$_3$), 7.01-7.04 (2H, m, Ph*H*), 7.61 (1H, d, *J* 15.7, C*H*), 7.69-7.71 (2H, m, Ph*H*), 7.75-7.78 (2H, m, Ph*H*), 7.80 (1H, d, *J* 15.7, C*H*), 8.06-8.10 (2H, m, Ph*H*). Spectra data in accordance with literature.[16]

(*E*)-4-(3-(4-methoxyphenyl)-3-oxoprop-1-en-1-yl)benzonitrile 52

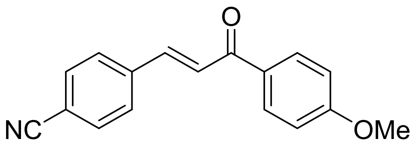

4-Formylbenzonitrile (1.31 g, 10 mmol) was dissolved in EtOH (20 mL), and a 3 M $NaOH_{aq}$ solution (10 mL) and 4′-methoxyacetophenone (1.50 g, 10 mmol) were added and the resulting mixture was stirred at room temperature for 1 h to form a yellow precipitate. The yellow solid was separated by vacuum filtration and was washed three times with 10 mL ice water. The dry crude product was purified by recrystallization from MeOH to yield the yellow titled product (1.55g, 59%). **mp**, 156-157 °C (literature: 167.8-172 °C[17]). **^{1}H NMR** (400 MHz, $CDCl_3$) δ_H: 3.92 (3H, s, OC*H*$_3$), 7.00-7.04 (2H, m, Ph*H*), 7.62 (1H, d, *J* 15.6, C*H*), 7.71-7.80 (5H, m, C*H* + Ph*H*), 8.04-8.09 (2H, m, Ph*H*). Spectra data in accordance with literature.[17]

(*E*)-3-(4-chlorophenyl)-1-(p-tolyl)prop-2-en-1-one 53

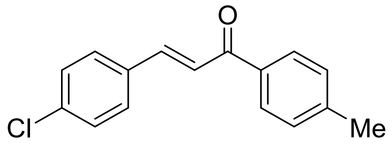

4-Chlorobenzaldehyde (1.40 g, 10 mmol) was dissolved in EtOH (20 mL), and a 3 M $NaOH_{aq}$ solution (10 mL) and 4′-methylacetophenone (1.34 g, 10 mmol) were added and the resulting mixture was stirred at room temperature for 2 h to form a white precipitate. The white solid was separated by vacuum filtration and was washed three times with 10 mL ice water. The dry crude product was purified by recrystallization from MeOH to yield the white titled product (2.20g, 86%). **mp**, 138-140 °C (literature: 138-139 °C[8]). **^{1}H NMR** (400 MHz, $CDCl_3$) δ_H: 2.47 (3H, s, C*H*$_3$), 7.32-7.34 (2H, m, Ph*H*), 7.40-7.43 (2H, m, Ph*H*), 7.55 (1H, d, *J* 15.7, C*H*), 7.58-7.62 (2H, m, Ph*H*), 7.79 (1H, d, *J* 15.7, C*H*), 7.94-7.97 (2H, m, Ph*H*). Spectra data in accordance with literature.[8]

(*E*)-3-(4-methoxyphenyl)-1-(4-(trifluoromethyl)phenyl)prop-2-en-1-one 54

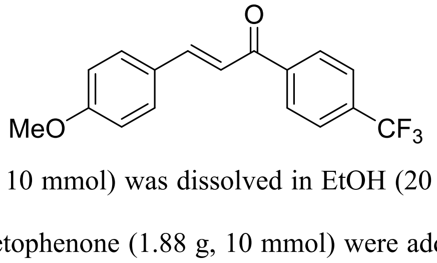

4-Methoxylbenzaldehyde (1.36 g, 10 mmol) was dissolved in EtOH (20 mL), and a 3 M $NaOH_{aq}$ solution (10 mL) and 4′-trifluoromethylacetophenone (1.88 g, 10 mmol) were added and the resulting mixture was stirred at room temperature for 2 h to form a light-yellow precipitate. The light yellow solid was separated by vacuum filtration and was washed three times with 10 mL ice water. The dry crude product was purified

by recrystallization from MeOH to yield the light yellow titled product (1.99 g, 65%). **mp**, 108-110 °C (literature: 116-118 °C[18]). **^{1}H NMR** (400 MHz, $CDCl_3$) δ_H: 3.89 (3H, s, OC*H$_3$*), 6.96-6.99 (2H, m, Ph*H*), 7.37 (1H, d, *J* 15.6, C*H*), 7.62-7.66 (2H, m, Ph*H*), 7.77-7.80 (2H, m, Ph*H*), 7.81 (1H, d, *J* 15.6, C*H*), 8.10-8.13 (2H, m, Ph*H*). Spectra data in accordance with literature.[18]

(*E*)-1-(4-chlorophenyl)-3-(p-tolyl)prop-2-en-1-one 55

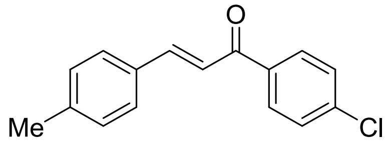

4-Methylbenzaldehyde (1.20 g, 10 mmol) was dissolved in EtOH (20 mL), and a 3 M $NaOH_{aq}$ solution (10 mL) and 4′-chloroacetophenone (1.54 g, 10 mmol) were added and the resulting mixture was stirred at room temperature for 2 h to form a white precipitate. The white solid was separated by vacuum filtration and was washed three times with 10 mL ice water. The dry crude product was purified by recrystallization from MeOH to yield the white titled product (2.10g, 81%). **mp**, 150-152 °C (literature: 153 °C[19]). **^{1}H NMR** (400 MHz, $CDCl_3$) δ_H: 2.42 (3H, s, C*H$_3$*), 7.25-7.27 (2H, m, Ph*H*), 7.45-7.52 (3H, m, C*H* + Ph*H*), 7.55-7.58 (2H, m, Ph*H*), 7.84 (1H, d, *J* 15.7, C*H*), 7.97-8.00 (2H, m, Ph*H*). Spectra data in accordance with literature.[19]

(*E*)-1,3-bis(4-chlorophenyl)prop-2-en-1-one 56

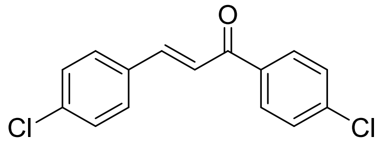

4-Chlorobenzaldehyde (1.40 g, 10 mmol) was dissolved in EtOH (20 mL), and a 3 M $NaOH_{aq}$ solution (10 mL) and 4′-chloroacetophenone (1.54 g, 10 mmol) were added and the resulting mixture was stirred at room temperature for 2 h to form a white precipitate. The white solid was separated by vacuum filtration and was washed three times with 10 mL ice water. The dry crude product was purified by recrystallization from MeOH to yield the white titled product (2.29g, 83%). **mp**, 145-146 °C (literature: 156-158 °C[20]). **^{1}H NMR** (400 MHz, $CDCl_3$) δ_H: 7.41-7.53 (5H, m, C*H* + Ph*H*), 7.58-7.62 (2H, m, Ph*H*), 7.81 (1H, d, *J* 15.8, C*H*), 7.96-8.00 (2H, m, Ph*H*). Spectra data in accordance with literature.[20]

(*E*)-4-(3-(4-methoxyphenyl)acryloyl)benzonitrile 57

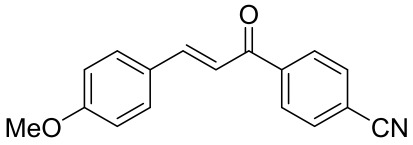

4-Methoxylbenzaldehyde (1.36 g, 10 mmol) was dissolved in EtOH (20 mL), and a 3 M $NaOH_{aq}$ solution (10 mL) and 4-acetylbenzonitrile (1.45 g, 10 mmol) were added and the resulting mixture was stirred at room temperature for 1 h to form a yellow precipitate. The yellow solid was separated by vacuum filtration and was washed three times with 10 mL ice water. The dry crude product was purified by recrystallization from MeOH to yield the yellow titled product (1.79g, 68%). **mp**, 136-137 °C (literature: 122-124 °C[18]). **^{1}H NMR** (400 MHz, $CDCl_3$) δ_H: 3.89 (3H, s, OC*H₃*), 3.95-6.99 (2H, m, Ph*H*), 7.34 (1H, d, *J* 15.6, C*H*), 7.62-7.65 (2H, m, Ph*H*), 7.81-7.85 (3H, m, C*H* + Ph*H*), 8.07-8.12 (2H, m, Ph*H*). Spectra data in accordance with literature.[18]

(*E*)-1,3-bis(4-(trifluoromethyl)phenyl)prop-2-en-1-one 58

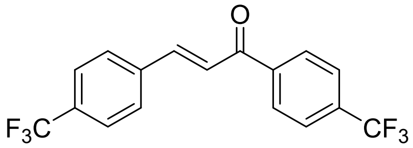

4-Trifluoromethylbenzaldehyde (1.74 g, 10 mmol) was dissolved in EtOH (20 mL), and a 3 M $NaOH_{aq}$ solution (10 mL) and 4′-trifluoromethylacetophenone (1.88 g, 10 mmol) were added and the resulting mixture was stirred at room temperature for 2 h to form a light-yellow precipitate. The yellow mixture was extracted using dichloromethane (x3), dried over Na_2SO_4, filtered, and concentrated to give a yellow crude product. The crude product was purified by silica gel column (Petrol: ethyl acetate = 10: 1) to yield the yellow soild product (1.24g, 36%). **mp**, 88-89 °C (literature: 98-100 °C[21]). **^{1}H NMR** (400 MHz, $CDCl_3$) δ_H: 7.56 (1H, d, *J* 15.8, C*H*), 7.71-7.73 (2H, m, Ph*H*), 7.77-7.83 (4H, m, Ph*H*), 7.84 (1H, d, *J* 15.8, C*H*), 8.13-8.16 (2H, m, Ph*H*). Spectra data in accordance with literature.[21]

(*E*)-4,4'-(3-oxoprop-1-ene-1,3-diyl)dibenzonitrile 59

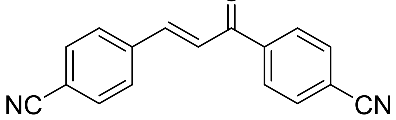

The procedure was based on the literature.[22] 4-Acetylbenzonitrile (1.45 g, 10 mmol) was dissolved in dry MeOH (14 mL) while stirring under nitrogen atmosphere. Upon complete dissolution of the reagent, solid 4-formylbenzonitrile (1.31 mg, 10 mmol) was added. The solution was heated at reflux, and stirred for 30

minutes. Then, 3M aq. NaOH (200 µL) was added until the formation of a yellow precipitate. After stirring at reflux for additional 15 minutes, the reaction was cooled at rt, a precipitate was filtered and washed with MeOH (3x30 mL). After drying, pure titled product was obtained as a yellow solid (1.45 g, 56% yield). **mp**, 179-180 °C. **^{1}H NMR** (400 MHz, $CDCl_3$) δ_H: 7.54 (1H, d, *J* 15.7, C*H*), 7.76 (4H, s, Ph*H*), 7.82-7.87 (3H, m, C*H* + Ph*H*), 8.11-8.14 (2H, m, Ph*H*). Spectra data in accordance with literature.[22]

(*E*)-1-phenyl-3-(pyridin-2-yl)prop-2-en-1-one 75 and 1,5-diphenyl-3-(pyridin-2-yl)pentane-1,5-dione 75a

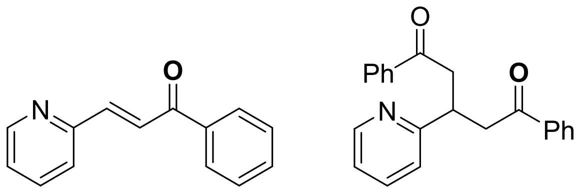

Pyridine-2-carbaldehyde (2.14 g, 1.90 mL, 20 mmol) was dissolved in MeOH (20 mL), and a 3 M $NaOH_{aq}$ solution (10 mL) and acetophenone (1.20 g, 10 mmol) were dropwise added and the resulting mixture was stirred at room temperature for overnight to form a yellow mixture. The yellow mixture was extracted using dichloromethane (x3), dried over Na_2SO_4, filtered, and concentrated to give a yellow crude product. The crude product was purified by silica gel column (Petrol: ethyl acetate = 10: 1) to give (E)-1-phenyl-3-(pyridin-2-yl)prop-2-en-1-one (1.23 g, 59%) as a light green solid and 1,5-diphenyl-3-(pyridin-2-yl)pentane-1,5-dione (0.51 g, 31%) as a white solid (The pentane-1,5-dione and en-1-one are very closed each other On TLC, but pentane-1,5-dione first come out from silica gel column).

(*E*)-1-phenyl-3-(pyridin-2-yl)prop-2-en-1-one 75: mp, 53-54 °C (literature: 61-62 °C[23]). **^{1}H NMR** (400 MHz, $CDCl_3$) δ_H: 7.32 (1H, ddd, *J* 7.6, 4.8, 1.2, ArC*H*), 7.50-7.56 (3H, m, Ph*H* + ArC*H*), 7.59-7.64 (1H, m, Ph*H*), 7.74-7.85 (2H, m, C*H* + Ar*H*), 8.11-8.17 (3H, m, Ph*H* + C*H*). 8.72 (1H, dq, *J* 4.8, 0.9, Ar*H*). Spectra data in accordance with literature.[23]

1,5-diphenyl-3-(pyridin-2-yl)pentane-1,5-dione 75a: mp, 109-110 °C (literature: 116 °C[24]). **^{1}H NMR** (400 MHz, $CDCl_3$) δ_H: 3.46 (2H, dd, *J* 17.3, 6.0, C*H$_2$*), 3.69 (2H, dd, *J* 17.4, 7.8, C*H$_2$*), 4.25 (1H, tt, *J* 7.7, 6.0, C*H*), 7.07 (1H, ddd, *J* 7.5, 4.8, 1.2, ArC*H*), 7.39-7.47 (5H, m, Ph*H* + ArC*H*), 7.53-7.62 (3H, m, ArC*H* + Ph*H*), 7.96-8.01 (4H, m, Ph*H*), 8.49 (1H, ddd, *J* 4.9, 1.8, 0.9, ArC*H*). Spectra data in accordance with literature.[24]

(*E*)-1-phenyl-3-(thiophen-2-yl)prop-2-en-1-one 76

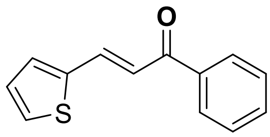

Thiophene-2-carbaldehyde (1.12 g, 0.93 mL, 10 mmol) was dissolved in MeOH (20 mL), and a 3 M $NaOH_{aq}$ solution (10 mL) and acetophenone (1.20 g, 10 mmol) were dropwise added and the resulting mixture was stirred at room temperature for 3 h to form a brown mixture. The brown mixture was extracted using dichloromethane (x3), dried over Na_2SO_4, filtered, and concentrated to give a brown crude product. The crude product was purified by silica gel column (Petrol: ethyl acetate = 10: 1) to yield the brown solid (1.90g, 89%). **mp**, 51-52 °C (literature: 56.7-58.0 °C[25]). **^{1}H NMR** (400 MHz, $CDCl_3$) δ_H: 7.12 (1H, dd, *J* 5.1, 3.6, ArC*H*), 7.33-7.40 (2H, m, C*H* + ArC*H*), 7.45 (1H, dt, *J* 5.0, 1.1, ArC*H*), 7.50-7.55 (2H, m, Ph*H*), 7.59-7.63 (1H, m, Ph*H*), 7.96 (1H, dt, *J* 15.3, 0.7, C*H*), 8.02-8.05 (2H, m, Ph*H*). Spectra data in accordance with literature.[25]

(*E*)-3-(furan-2-yl)-1-phenylprop-2-en-1-one 77

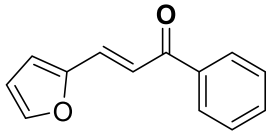

Furfural (0.96 g, 0.83 mL, 10 mmol) was dissolved in MeOH (20 mL), and a 3 M $NaOH_{aq}$ solution (10 mL) and acetophenone (1.20 g, 10 mmol) were dropwise added and the resulting mixture was stirred at room temperature for 3 h to form a brown mixture. The brown mixture was extracted using dichloromethane (x3), dried over Na_2SO_4, filtered, and concentrated to give a brown crude product. The crude product was purified by silica gel column (Petrol: ethyl acetate = 10: 1) to yield the brown solid (1.60g, 81%). **mp**, 34-35 °C (literature: 39-40 °C[26]). **^{1}H NMR** (400 MHz, $CDCl_3$) δ_H: 6.54 (1H, dd, *J* 3.4, 1.8, ArC*H*), 6.74 (1H, d, *J* 3.4, ArC*H*), 7.47-7.64 (6H, m, C*H* + ArC*H* + Ph*H*), 8.03-8.07 (2H, m, Ph*H*). Spectra data in accordance with literature.[25]

(*E*)-4,4,4-trifluoro-1-phenylbut-2-en-1-one 78

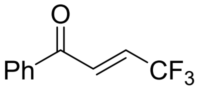

The preparation was adapted from the literature procedure.[27] *n*-BuLi (2.5 M in hexanes, 8.8 mL, 22 mmol) was slowly added into the solution of *i*-Pr_2NH (3.1 mL, 22 mmol) in THF (35 mL) at -78 °C, then the solution

was stirred for 20 mins. 2-Bromo-3,3,3-trifluoroprop-1-ene (1.1 mL, 10 mmol) was added dropwise at -78 °C followed by a further 5 mins of stirring. Benzaldehyde (1.0 mL, 10 mmol) was added dropwise followed by stirring at -78 °C for 30 minutes. The reaction mixture was quenched by adding HCl (1 M in H_2O, 20 mL) and then warm to rt, followed by extraction with EtOAc (×3), desiccation with $MgSO_4$, filtration, and concentration in vacuo to give the crude intermediate propargyl alcohol. Without further purification, the resulting oil was dissolved in THF (10 mL), then Et_3N (5.6 mL, 40 mmol) was added before the reaction mixture was heated at reflux overnight. The reaction mixture was quenched by adding HCl (1 M in H_2O, 30 mL), followed by extraction with EtOAc (×3), washing with water (×1), desiccation with $MgSO_4$, filtration, and concentration in vacuo to give the crude reaction mixture. The mixture was purified by column chromatography (petroleum ether/ethyl acetate = 10/1, and collected the first dot using TLC to detect the process) to give a light yellow solid (1.10 g, 55%).

Light yellow solid; R_f = 0.68 (petroleum ether:ethyl acetate, 5:1); mp 26-27 °C (26-28 °C[27]). **^{1}H NMR** (400 MHz, $CDCl_3$) δ_H: 6.84 (1H, dq, *J* 15.6, 6.6, =C*H*), 7.52-7.59 (3H, m, =C*H* + Ph*H*), 7.65-7.70 (1H, m, Ph*H*), 7.99-8.02 (2H, m, Ph*H*). Data in accordance with literature.[27]

(*E*)-1-phenylbut-2-en-1-one 79

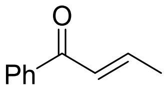

The preparation was adapted from the literature procedure.[28] $AlCl_3$ (1.34 g, 10.0 mmol) was suspended in benzene (10 mL) under an atmosphere of argon. Crotonic acid chloride (1.05 g, 10.0 mmol) was added dropwise under vigorous stirring. After 20 min the mixture was added to a mixture of water and 6 M HCl solution (20mL:2mL) and extracted with DCM (3 x 30 mL). The combined organic phases were washed with 1 M NaOH (20 mL), dried over Na_2SO_4, filtered and concentrated under reduced pressure. The product was further purified via column chromatography on silica (Petroleum ether/EtOAc 25:1) to give pure trans-β-crotonophenone (0.58 g, 40%) as a colorless liquid.

Colorless liquid; R_f = 0.78 (petroleum ether:ethyl acetate, 5:1); **^{1}H NMR** (400 MHz, $CDCl_3$) δ_H: 2.03 (3H, dd, *J* 6.8, 1.6, C*H*), 6.93 (1H, dq, *J* 15.3, 6.8, =C*H*), 7.09 (1H, dq, *J* 15.3, 1.6, =C*H*), 7.46-7.51 (2H, m, Ph*H*), 7.55-7.60 (1H, m, Ph*H*), 7.93-7.97 (2H, m, Ph*H*). Data in accordance with literature.[28]

(*E*)-1-(4-fluorophenyl)-3-(furan-2-yl)prop-2-en-1-one 85

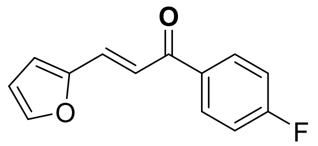

Furfural (0.96 g, 0.83 mL, 10 mmol) was dissolved in MeOH (20 mL), and a 3 M $NaOH_{aq}$ solution (10 mL) and 4′-fluoroacetophenone (1.38 g, 10 mmol) were dropwise added and the resulting mixture was stirred at room temperature for 3 h to form a brown mixture. The mixture was extracted using dichloromethane (x3), dried over Na_2SO_4, filtered, and concentrated to give a brown crude product. The crude product was purified by silica gel column (Petrol: ethyl acetate = 10: 1) to yield the brown solid (1.36g, 63%). **mp**, 63-64 °C (literature: 65-66 °C[29]). **^{1}H NMR** (500 MHz, $CDCl_3$) δ_H: 6.54 (1H, dd, *J* 3.4, 1.8, ArC*H*), 6.75 (1H, d, *J* 3.4, ArC*H*), 7.16-7.21 (2H, m, Ph*H*), 7.43 (1H, d, *J* 15.3, C*H*), 7.55 (1H, d, *J* 1.8, ArC*H*), 7.60 (1H, d, *J* 15.3, C*H*), 8.07-8.11 (2H, m, Ph*H*). Spectra data in accordance with literature.[30]

(E)-1-(4-chlorophenyl)-3-(furan-2-yl)prop-2-en-1-one 86

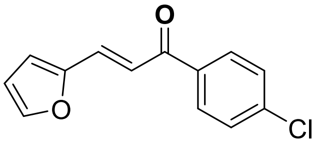

Furfural (0.96 g, 0.83 mL, 10 mmol) was dissolved in MeOH (20 mL), and a 3 M $NaOH_{aq}$ solution (10 mL) and 4′-chloroacetophenone (1.54 g, 10 mmol) were dropwise added and the resulting mixture was stirred at room temperature for 3 h to form a white mixture. The brown mixture was extracted using dichloromethane (x3), dried over Na_2SO_4, filtered, and concentrated to give a crude product. The crude product was purified by silica gel column (Petrol: ethyl acetate = 10: 1) to yield the white solid (1.83g, 79%). **mp**, 68-69 °C (literature: 88-90 °C[31]). **^{1}H NMR** (400 MHz, $CDCl_3$) δ_H: 6.55 (1H, dd, *J* 3.4, 1.8, ArC*H*), 6.76 (1H, dt, *J* 3.4, 0.6, ArC*H*), 7.42 (1H, d, *J* 15.3, C*H*), 7.47-7.51 (2H, m, Ph*H*), 7.56-7.57 (1H, m, ArC*H*), 7.60 (1H, d, *J* 15.3, C*H*), 7.98-8.02 (2H, m, Ph*H*). Spectra data in accordance with literature.[32]

(*E*)-1-(4-bromophenyl)-3-(furan-2-yl)prop-2-en-1-one 87

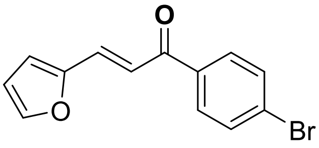

Furfural (0.96 g, 0.83 mL, 10 mmol) was dissolved in MeOH (20 mL), and a 3 M $NaOH_{aq}$ solution (10 mL) and 4′-bromoacetophenone (1.80 g, 10 mmol) were dropwise added and the resulting mixture was stirred at

room temperature for 3 h to form a white mixture. The brown mixture was extracted using dichloromethane (x3), dried over Na_2SO_4, filtered, and concentrated to give a crude product. The crude product was purified by silica gel column (Petrol: ethyl acetate = 10: 1) to yield the white solid (2.15g, 78%). **mp**, 69-70 °C (literature: 80-82 °C[33]). **^{1}H NMR** (400 MHz, $CDCl_3$) δ_H: 6.55 (1H, dd, *J* 3.4, 1.8, ArC*H*), 6.76 (1H, dq, *J* 3.5, 0.6, ArC*H*), 7.40 (1H, d, *J* 15.3, C*H*), 7.56-7.57 (1H, m, ArC*H*), 7.60-7.67 (3H, m, C*H* + Ph*H*), 7.91-7.94 (2H, m, Ph*H*). Spectra data in accordance with literature.[33]

(*E*)-3-(furan-2-yl)-1-(p-tolyl)prop-2-en-1-one 88

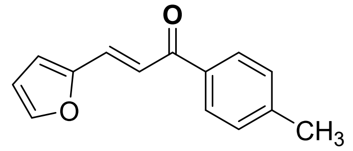

Furfural (0.96 g, 0.83 mL, 10 mmol) was dissolved in MeOH (20 mL), and a 3 M $NaOH_{aq}$ solution (10 mL) and 4′-methylacetophenone (1.34 g, 10 mmol) were dropwise added and the resulting mixture was stirred at room temperature for 3 h to form a light yellow mixture. The mixture was extracted using dichloromethane (x3), dried over Na_2SO_4, filtered, and concentrated to give a light yellow crude product. The crude product was purified by silica gel column (Petrol: ethyl acetate = 10: 1) to yield the light yellow solid (1.08g, 51%). **mp**, 63-65 °C (literature: 64-67 °C[34]). **^{1}H NMR** (400 MHz, $CDCl_3$) δ_H: 2.45 (1H, s, C*H$_3$*), 6.53 (1H, dd, *J* 3.4, 1.8, ArC*H*), 6.73 (1H, dd, *J* 3.4, 0.7, ArC*H*), 7.30-7.33 (2H, m, Ph*H*), 7.47 (1H, d, *J* 15.4, C*H*), 7.54-7.55 (1H, m, ArC*H*), 7.60 (1H, d, *J* 15.4, C*H*), 7.96-7.99 (2H, m, Ph*H*). Spectra data in accordance with literature.[35]

Dimethyl 2-ethylidenemalonate 93

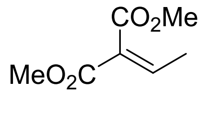

The preparation was adapted from a literature procedure.[36] Dimethyl malonate (1.2 mL, 10 mmol), acetic anhydride (1.9 mL, 20 mmol), and Lithium bromide (0.35 g, 4 mmol) were added into a 25 mL round bottom flask equipped with a reflux condenser. The reaction mixture was stirred at 80ºC for 4 h reflux under dry N_2. Then acetaldehyde (1.7 mL, 30 mmol) was swiftly added into the reaction through the reflux condenser, and the mixture was stirred at 80ºC for another 1.5 hours. After reflux, the reaction mixture was then poured into 15 mL of sat. Na_2CO_3 solution, followed by extraction with CH_2Cl_2 (3 x 15 mL). The residue was purified by silica gel chromatography (petroleum ether/ethyl acetate = 10/1, and collected the first dot using TLC to

detect the process) to yield the product as a colorless oil (632 mg, 40%).

Colorless oil; R_f = 0.45 (petroleum ether:ethyl acetate, 5:1); **^{1}H NMR** (400 MHz, $CDCl_3$) δ_H: 1.97 (3H, d, *J* 7.3, C*H$_3$*), 3.79 (3H, s, C*H$_3$*), 3.85 (3H, s, C*H$_3$*), 7.14 (1H, q, *J* 7.3, C*H*). Data in accordance with literature.[36]

(*E*)-(2-nitrovinyl)cyclohexane 95

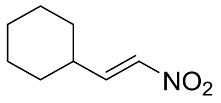

The preparation method is based on literature.[37] Cyclohexanecarboxaldehyde (16.5 mmol, 2.0 mL) and nitromethane (16.5 mmol, 900 μL) were dissolved in methanol (5.0 mL) and a cold solution of NaOH (2.0 Equiv., 33.0 mmol, 1.32 g) in water (5.0 mL) was added at room temperature. After the addition was complete, the mixture was stirred for 1 h at room temperature. To this suspension was added a cold mixture of conc. HCl/water (1/1, 20 mL) at room temperature and the mixture was then stirred for another hour. The product was isolated using CH_2Cl_2 (3 x 25 mL), the organic layer was dried over Na_2SO_4. The crude product was purified by silica gel chromatography (petroleum ether/ethyl acetate = 20/1, and collected the first dot using TLC to detect the process) to yield the titled product as a yellowish oil (1.30 g, 51%).

Yellowish oil; R_f = 0.64 (petroleum ether:ethyl acetate, 5:1); **^{1}H NMR** (400 MHz, CD_2Cl_2) δ_H: 1.16-1.39 (5H, m, C*H$_2$*), 1.69-1.86 (5H, m, C*H$_2$*), 2.23-2.32 (1H, m, C*H*), 6.93 (1H, dd, *J* 13.5, 1.4, =C*H*), 7.23 (1H, dd, *J* 13.5, 7.2, =C*H*). Data in accordance with literature.[37]

Methyl (*E*)-2-oxo-4-phenylbut-3-enoate 96

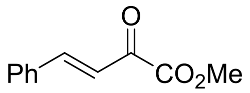

The preparation was adapted from the literature procedure.[38] A solution of pyruvic acid (0.92 g, 10.5 mmol) in 15 mL of methanol was stirring in room temperature then a freshly prepared solution of potassium hydroxide (0.84 g, 15 mmol) in methanol was added to the solution dropwise until pH 13. Benzaldehyde (10.0 mmol, 1.3 mL) was added, then another freshly prepared solution of potassium hydroxide (0.84 g, 15 mmol) in methanol was added to the mixture dropwise. During the addition, the solution turns a clear yellow and eventually yellow precipitates. After the complete addition of the base, the reaction was stirred for 4h. The reaction was quenched with 6M HCl until pH 2 and extracted with ethyl acetate (3 x 20 mL), dried with sodium sulfate anhydrous and the solvent was removed under reduced pressure to give the yellow oil,

followed by redissolving in methanol (20 mL) and toluene (10 mL), then concentrated hydrochloric acid (1 mL) was added. The mixture was heated to 95 °C for 4 h, cooled to ambient temperature and the solvent was removed in vacuo to give the residual oil, followed by a purification with flash column chromatography to give a light yellow product (0.93 g, 49%).

Light yellow solid; R_f = 0.64 (petroleum ether:ethyl acetate, 5:1); mp 67-69 °C (68-70 °C[39]). **^{1}H NMR** (400 MHz, CD_2Cl_2) δ_H: 3.95 (3H, s, C*H₃*), 7.36 (1H, d, *J* 16.1, =C*H*), 7.46-7.53 (3H, m, Ph*H*), 7.69-7.72 (2H, m, Ph*H*), 7.85 (1H, dd, *J* 16.2, 0.6, =C*H*). Data in accordance with literature.[39]

Methyl (*E*)-4-oxo-4-phenylbut-2-enoate 97

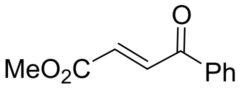

The preparation was adapted from the literature procedure.[27] Triphenylphosphine (2.62 g, 10 mmol) was added into a solution of 2-bromo-1-phenylethanone (1.98 g, 10 mmol) in CH_2Cl_2 (30 mL) at rt. The solution was allowed to stir for 4 h before being concentrated in vacuo, followed by removing the solvent under reduced pressure to give the phosphonium salt. Without further purification, it was kept under seal for the following reaction. To a solution of dimethyl tartrate (2.50 g, 14 mmol) in Et_2O (30 mL) at rt was added periodic acid (2.28 g, 14 mmol) and the solution was allowed to stir for 1h. The reaction mixture was filtered and the solid was washed with Et_2O to give the solution. The obtained solution was added into the above phosphonium salt, and K_2CO_3 (4.14 g, 40 mmol) was also added into the mixture, followed by a 5 h of stirring at rt, and the solvent was removed under reduced pressure to give the crude mixture, purified by column chromatography to yield the light yellow solid (1.20 g, 63%).

Light yellow solid; R_f = 0.59 (petroleum ether:ethyl acetate, 5:1); mp 33-34 °C (36.4-37.5 °C[40]). **^{1}H NMR** (400 MHz, $CDCl_3$) δ_H: 3.87 (3H, s, C*H₃*), 6.90 (1H, d, *J* 15.6, =C*H*), 7.52-7.56 (2H, m, Ph*H*), 7.62-7.67 (1H, m, Ph*H*), 7.93 (1H, d, *J* 15.6, =C*H*), 8.01-8.04 (2H, m, Ph*H*). Data in accordance with literature.[27]

(*E*)-4,4,4-trifluoro-1-phenylbut-2-en-1-one 98

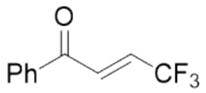

The preparation was adapted from the literature procedure.[27] *n*-BuLi (2.5 M in hexanes, 8.8 mL, 22 mmol) was slowly added into the solution of *i*-Pr_2NH (3.1 mL, 22 mmol) in THF (35 mL) at -78 °C, then the solution was stirred for 20 mins. 2-Bromo-3,3,3-trifluoroprop-1-ene (1.1 mL, 10 mmol) was added dropwise at -78

°C followed by a further 5 mins of stirring. Benzaldehyde (1.0 mL, 10 mmol) was added dropwise followed by stirring at -78 °C for 30 minutes. The reaction mixture was quenched by adding HCl (1 M in H_2O, 20 mL) and then warm to rt, followed by extraction with EtOAc (×3), desiccation with $MgSO_4$, filtration, and concentration in vacuo to give the crude intermediate propargyl alcohol. Without further purification, the resulting oil was dissolved in THF (10 mL), then Et_3N (5.6 mL, 40 mmol) was added before the reaction mixture was heated at reflux overnight. The reaction mixture was quenched by adding HCl (1 M in H_2O, 30 mL), followed by extraction with EtOAc (×3), washing with water (×1), desiccation with $MgSO_4$, filtration, and concentration in vacuo to give the crude reaction mixture. The mixture was purified by column chromatography (petroleum ether/ethyl acetate = 10/1, and collected the first dot using TLC to detect the process) to give a light yellow solid (1.10 g, 55%).

Light yellow solid; R_f = 0.68 (petroleum ether:ethyl acetate, 5:1); mp 26-27 °C (26-28 °C[42]). ^{1}H NMR (400 MHz, $CDCl_3$) δ_H: 6.84 (1H, dq, *J* 15.6, 6.6, =C*H*), 7.52-7.59 (3H, m, =C*H* + Ph*H*), 7.65-7.70 (1H, m, Ph*H*), 7.99-8.02 (2H, m, Ph*H*). Data in accordance with literature.[27]

(*E*)-1,1,1-trichloro-4-phenylbut-3-en-2-one 99

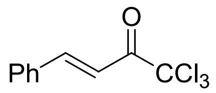

The preparation was adapted from the literature procedure.[41] A solution of KOH (20 mmol, 1.12 g) in EtOH (8 mL) was added dropwise to a stirred solution of cinnamyl aldehyde (20 mmol, 2.5 mL) and chloroform (44 mmol, 3.5mL) in DMF (30 mL) at 0 °C. The mixture turned deep red and was stirred at 0 °C for another 45 mins. The mixture was acidified with 2 M HCl to pH 5-7 and then extracted with EtOAc (×3), washed with water. The combined organics were dried over Na_2SO_4, filtered and concentrated under reduced pressure to give the crude product, followed by purification with flash silica column chromatography to give the brown oil alcohol product (3.35 g, 67%). A solution of DMSO (80 mmol, 5.7 mL) in anhydrous CH_2Cl_2 (5 mL) was added dropwise to a solution of oxalyl chloride (40 mmol, 3.4 mL) in CH_2Cl_2 (40 mL) at -78 °C. After 2 mins, a solution of the above alcohol product (10 mmol, 2.5 g) in CH_2Cl_2 (10 mL) was added dropwise. After a further 15 min, Et_3N (100 mmol, 27.8 mL) was added dropwise and a large amount of white solid was precipitated, followed by a vigorous stirring for 0.5 h, allowing it to warm to rt. The mixture was then quenched with 2 M HCl and extracted with CH_2Cl_2 (×3). The combined organics were dried over Na_2SO_4, filtered, concentrated under reduced pressure and purified by flash silica column chromatography

to give the yellow oil, and then crystallized in freezing methanol to give a light-yellow crystal product (1.80 g, 73%)

Light yellow solid; R_f = 0.72 (petroleum ether:ethyl acetate, 5:1); mp 56-57 °C (57-58 °C[41]). **^{1}H NMR** (400 MHz, $CDCl_3$) δ_H: 7.35 (1H, d, *J* 15.7, =C*H*), 7.44-7.53 (3H, m, Ph*H*), 7.66-7.70 (2H, m, Ph*H*), 8.02 (1H, d, *J* 15.7, =C*H*). Data in accordance with literature.[41]

3. Determination of Rate Constants (k_s') for Stetter Product Formation

As our knowledge, the competing benzoin reaction is the key obstacle for quantifying the reactivity of a variety of Michael acceptors. To mitigate the influence of the benzoin reaction, we proposed to enhance the disparity of reaction rates between these two reactions, thereby minimizing the interference with each other. With this concept in mind, highly reactive aldehydes and NHC catalysts are selected to expedite the benzoin reaction, while the Stetter reaction proceeds at a slower pace through use of suitable Michael acceptors. This strategic choice potentially allows the final step to become the rate-limiting step for the Stetter reaction as both reactions proceed through the same rapidly formed BI, starting from the aldehyde and catalyst (Scheme S1).

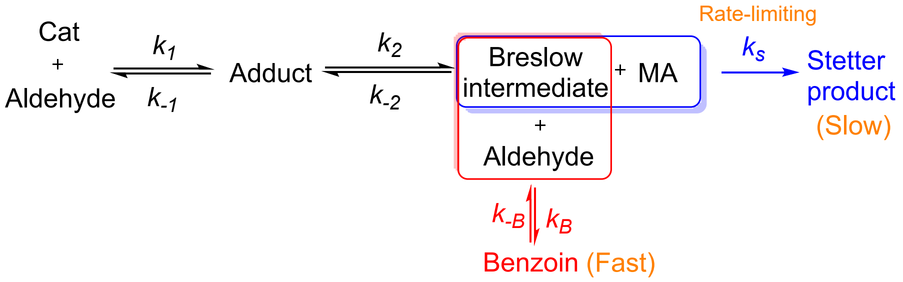

Scheme S1. The Stetter reaction and benzoin reaction.

In our previous research, heteroaromatic aldehydes exhibited exceptionally high reactivity for tetrahedral adduct formation compared with benzaldehydes. After extensive experimentation involving the screening of a range of heteroaromatic aldehydes and NHC precatalysts, pyridine-2-carboxyaldehyde **14** and *N*-C_6F_5 triazolium salt **16** were chosen as the substrate and catalyst for this experiment, with (*E*)-chalcone **17** used as the Michael acceptor. To gain detailed insight into the process, the reaction was monitored by temperature controlled (25°C) ^{1}H NMR spectroscopy on a Bruker Avance 500 MHz NMR spectrometer. Spectra were taken at 3-minute intervals over 20 hours. Representative NMR spectra over the course of the experiment are given in Figure S1

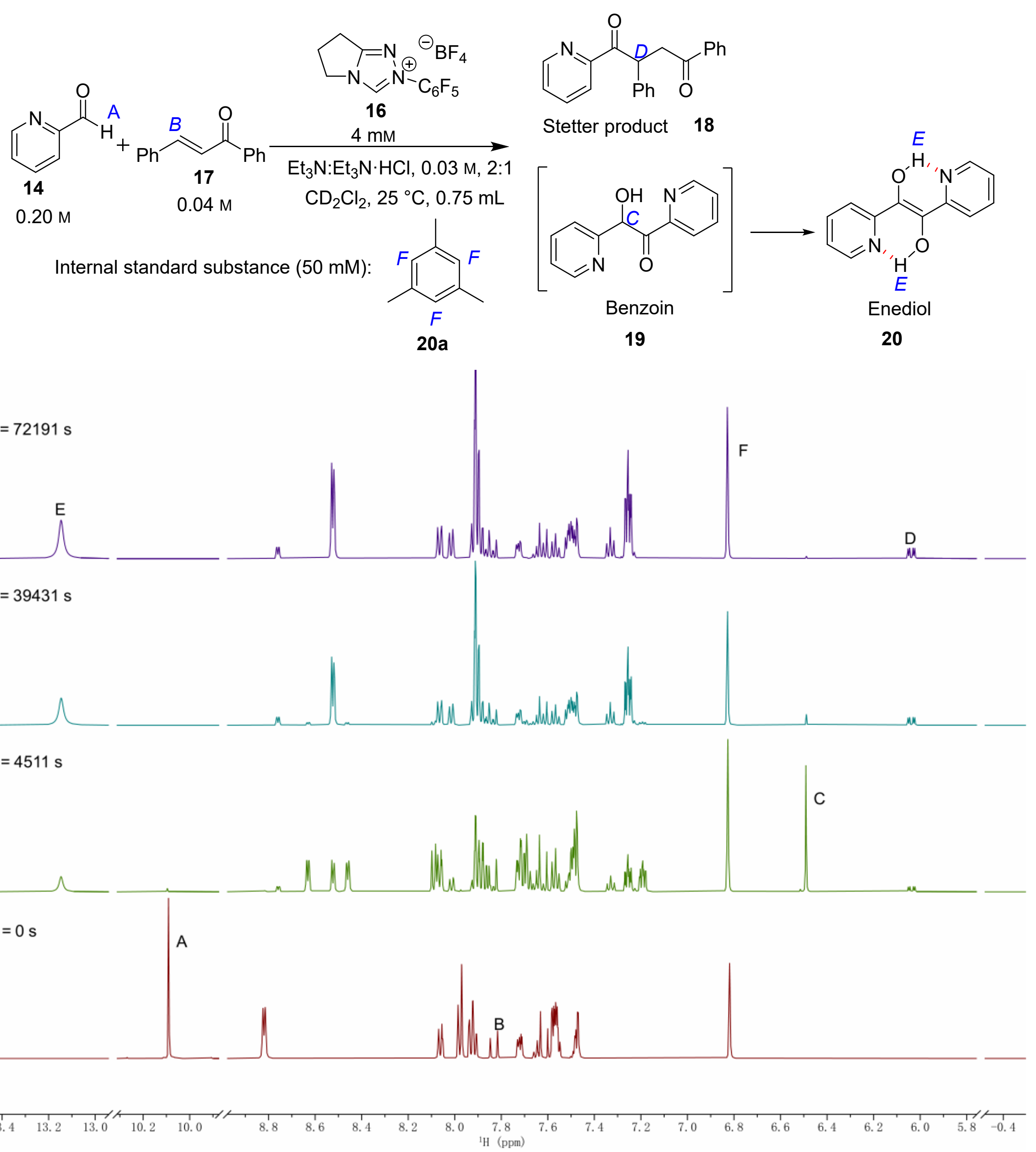

Figure S1. Representative ^{1}H NMR spectra (500 MHz) for reaction of 2-pyridinecarboxaldehyde **14** (0.20 M) with (*E*)-Chalcone **17** (0.04 M) catalysed by the NHC derived from *N*-C_6F_5 precatalyst **16** (4 mM) under a triethylamine buffered (NEt_3:NEt_3·HCl, 2:1, 0.03 M) CD_2Cl_2 at 25 °C. A = Aldehyde **14** C*H*O, B = (*E*)-Chalcone **17** C*H*, C = Benzoin **19** C*H*, D = Stetter product **18** C*H*, E = Enediol **20** O*H*, F = 2,4,6-trimethylbenzene **20a** Ph*H*.

Before initiation of the reaction by triethylamine buffer (t = 0 min), two signals at 10.09 ppm (**A**) and 7.82 ppm (**B**, doublet, the ratio of large peak relative to the doublet is 0.574) appear in the ^{1}H NMR spectrum, belonging to C*H*O of pyridine-2-carboxaldehyde **14** and C*H* of (*E*)-chalcone **17** respectively, and their concentrations at different times are determined by integrating relative to the internal standard (1,3,5-trimethylbenzene, 50 mM) signal **F** (Equations S4.1 and S4.2). After initiation by addition of triethylamine

buffer, three new species of interest are identifiable. A quartet (dd) at 6.05 ppm (**D**) appears, belonging to C*H* of Stetter product **18** and two new singlet signals at 6.49 ppm and 13.15 ppm (**E**) appear. The signal at 6.49 ppm is assigned to C*H* benzoin **19**, though it should be noted that this benzoin product could not be isolated as it is unstable to column chromatography but is assigned by analogy to other benzoin products. The signal at 13.15 ppm is assigned to O*H* of known enediol **20**, which is stabilized by two hydrogen bonds. The concentrations of benzoin **19**, enediol **20** and Stetter product **18** at different times are determined using Equations S3-5 and the total concentration of benzoin, [Benzoin(tot)], is calculated using Equation S6. The resulting concentration profile of this reaction is also displayed in Scheme S2.

$$[Aldehyde]_{(t=x)} = 3 * \frac{A_{A(t=x)}}{A_F} \times 0.05$$ **Equation S1**

$$[Chalcone]_{(t=x)} = 3 * \frac{A_{B(t=x)}}{0.574 * A_F} \times 0.05$$ **Equation S2**

$$[Benzoin]_{(t=x)} = 3 * \frac{A_{C(t=x)}}{A_F} \times 0.05$$ **Equation S3**

$$[Stetter]_{(t=x)} = 3 * \frac{A_{D(t=x)}}{A_F} \times 0.05$$ **Equation S4**

$$[Enediol]_{(t=x)} = 3 * \frac{A_{E(t=x)}}{2 * A_F} \times 0.05$$ **Equation S5**

$$[Benzoin(tot)] = [Benzoin] + [Enediol]$$ **Equation S6**

Analyzing the concentration profile, we observed that almost all pyridine-2-carboxyaldehyde **14** is rapidly transformed into the corresponding benzoin product **19** demonstrating that the resting state of the aldehyde is the benzoin. In our previous study, the *N*-C_6F_5 triazolium catalyst **16** showed a very high reactivity when reacting with pyridine-2-carboxyaldehyde **14** as the aldehyde **14** was totally transferred into tetrahedral adduct (TI) and benzoin product. Therefore, by using an initial concentration of pyridine-2-carboxyaldehyde **14** that is much larger than the initial concentration ($[Cat]_0$) of *N*-C_6F_5 triazolium precatalyst **16** in the kinetic experiment ($[aldehyde]_0 >> [Cat]_0$), the NHC is saturated and the rapid and reversible benzoin will not affect the rate of the Stetter reaction. The benzoin product **19** is slowly consumed, by i) reversal to the BI leading to irreversible Stetter reaction and ii) irreversible formation of the enediol. The irreversibility of both of these processes was confirmed by experiment.

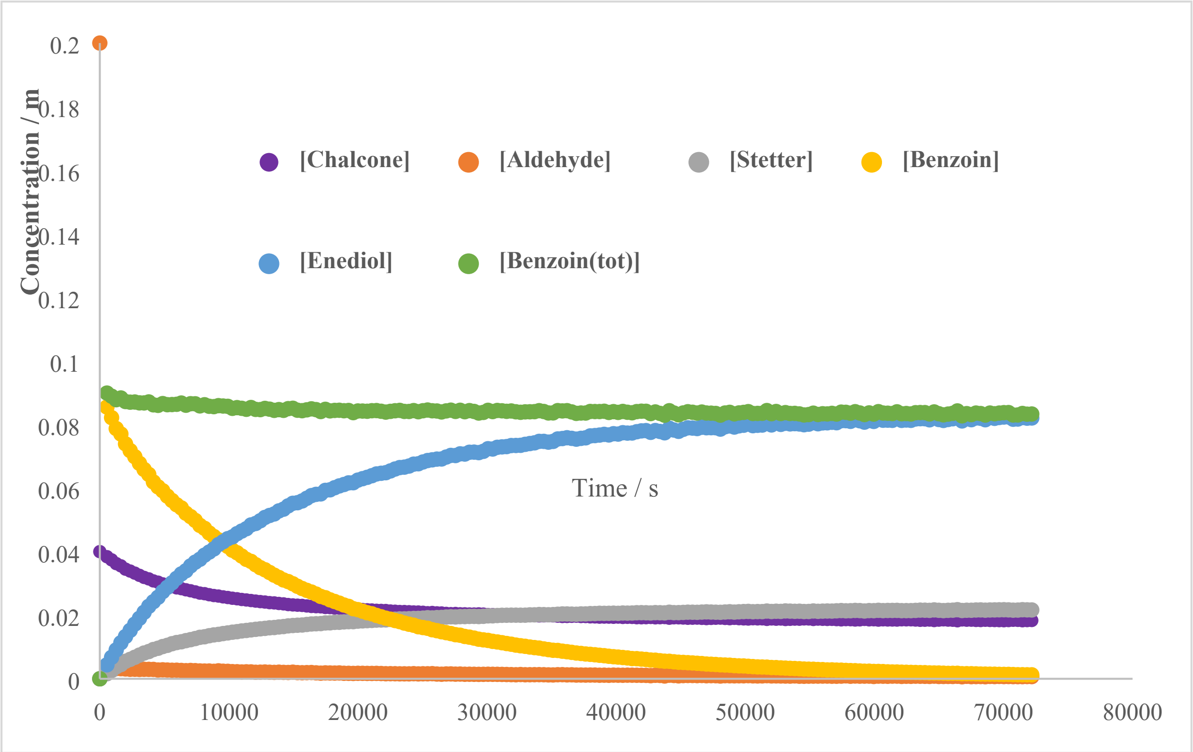

Scheme S2. Concentration profile for the reaction between pyridine-2-carboxyaldehyde **14** and (*E*)-chalcone **17** catalyzed by the NHC derived from *N*-C_6F_5 triazolium catalyst **16** under a triethylamine buffered (NEt_3:NEt_3·HCl, 2:1, 0.03 M) CD_2Cl_2 at 25 °C.

First, enediol **20** was subjected to catalytic reaction and no consumption enediol **20** was observed after 16 h (Figure S2b). (*E*)-Chalcone **17** was added to this reaction mixture, and the Stetter product **18** was not observed after a further 10 hours (Figure S2c). This gives strong evidence of the irreversibility of the slow enolization process.

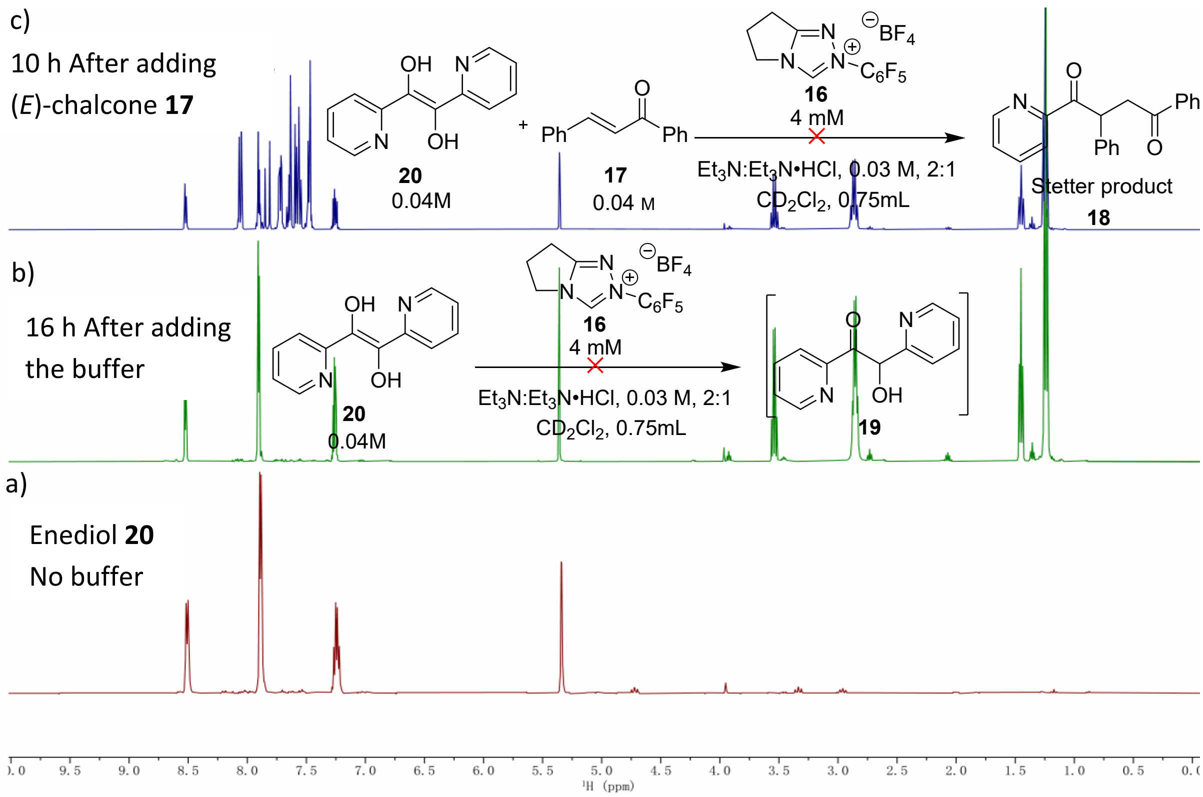

Figure S2. Representative ^{1}H NMR spectra for the experiments of enediol **20** (0.40 M) catalysed by the NHC derived from *N*-C_6F_5 precatalyst **16** (4 mM) under a triethylamine buffered (NEt_3:NEt_3·HCl, 2:1, 0.03 M) CD_2Cl_2 at 25 °C.

Then，1,4-diketone **18** was subjected to the same catalytic reaction conditions，and after 3 days，no pyridine-2-carboxyaldehyde **14** or (*E*)-chalcone **17** were observed by ^{1}H NMR spectrum (Figure S3).

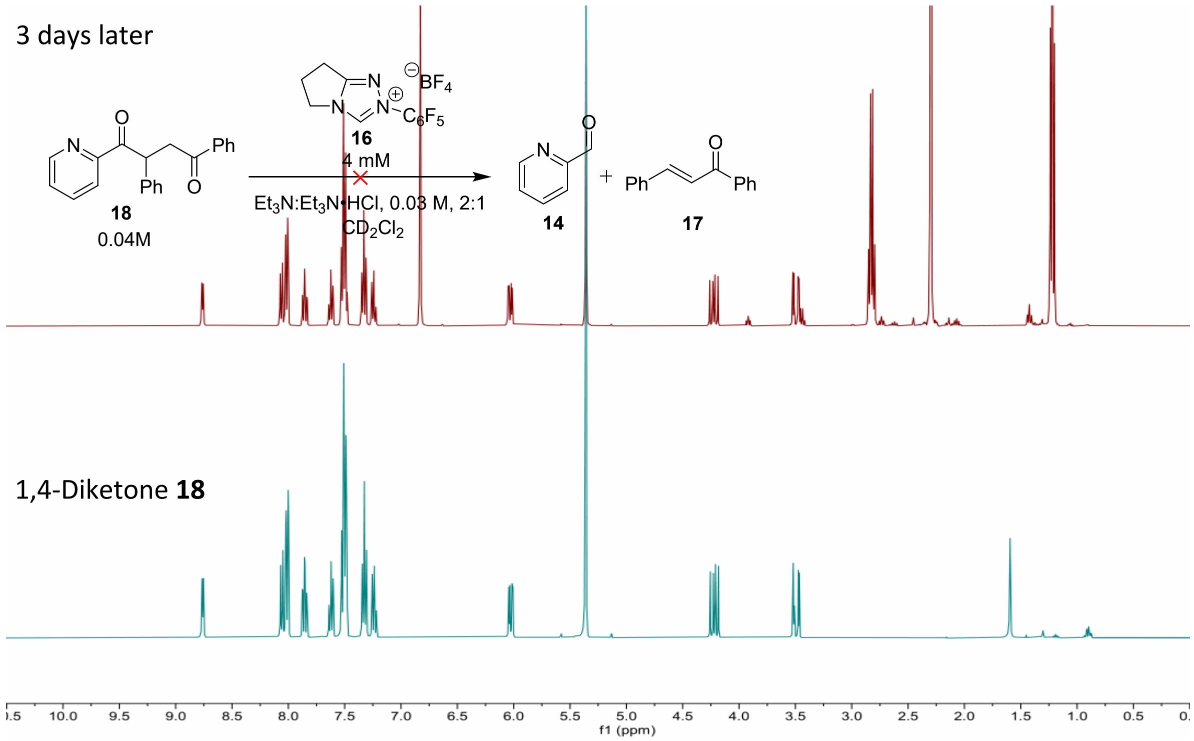

Figure S3. Representative ^{1}H NMR spectra for the experiment of 1,4-diketone **18** (0.40 M) catalysed by the

NHC derived from *N*-C_6F_5 precatalyst **16** (4 mM) under a triethylamine buffered (NEt_3:NEt_3·HCl, 2:1, 0.03 M) CD_2Cl_2 at 25 °C.

Considering these above observations, the reaction mechanism for this experiment is proposed in Scheme S3. Using catalyst **16** and pyridine-2-carboxyaldehyde **14**, the benzoin reaction exhibits a remarkably fast reaction rate compared with the Stetter reaction. This suggests that the formation of BI is rapid and that the irreversible reaction of the BI **18b** with (*E*)-chalcone **17** is the rate-determining step for Stetter reaction.

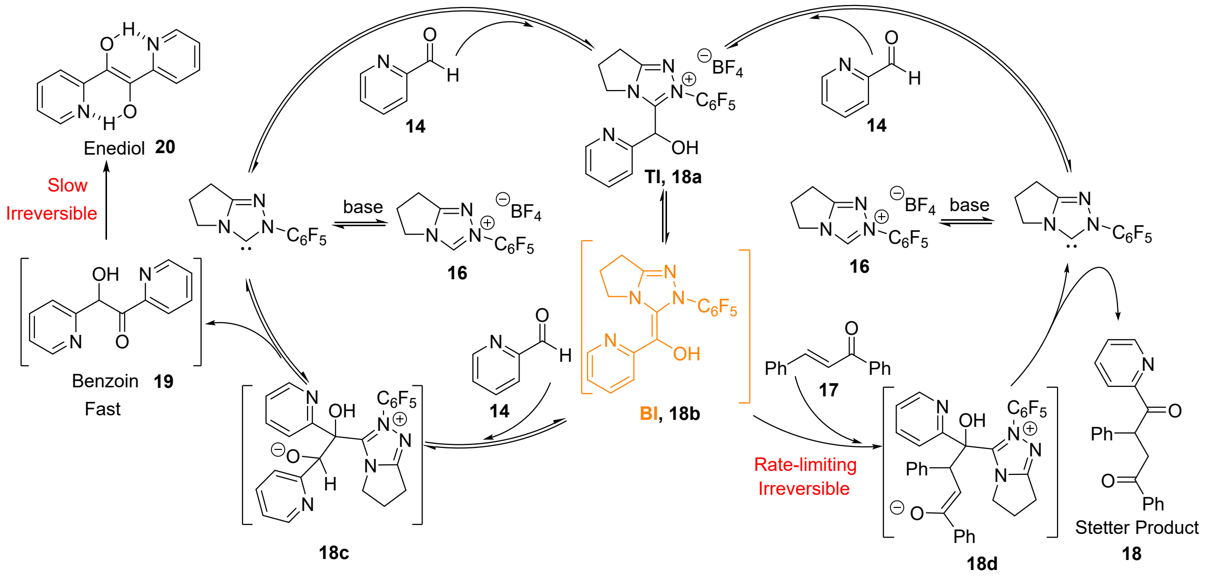

Scheme S3. The mechanism of the reaction of pyridine-2-carboxyaldehyde **14** and chalcone **17** catalysed by the *N*-C_6F_5 triazolium catalyst **16** derived N-heterocyclic carbene.

Since the final step is the rate-determining step in the Stetter reaction, the rate of generation of the Stetter product **18** can be expressed using Equation S4.7 (where [BI] is the concentration of BI **18b** and [MA] is the concentration of (*E*)-chalcone **17**). Due to catalyst saturation by aldehyde **14**, adduct **18a** concentration, [TI], is constant and equal to the initial concentration of triazolium catalyst **16** ($[Cat]_0$) (Scheme S4). Because TI is also in fast equilibrium with BI, [BI] can also be considered constant at the beginning of the reaction when [Ald] >> [NHC]. [BI] can therefore be incorporated into the pseudo first-order rate constant $k_s^{'}$ (Equation S8).

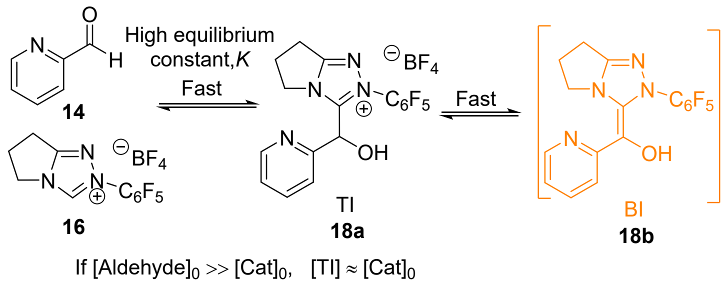

Scheme S4. The kinetic model of the reaction of pyridine-2-carboxyaldehyde **14** with *N*-C_6F_5 triazolium catalyst **16.**

Using an the initial rates method, the concentration of (*E*)-chalcone **17** ([MA]) is approximated to be equal to its initial concentration ($[MA]_0$) and Equation S8 can be rewritten as Equation S9, which implies that the value of $k_s^{'}$ can be calculated using Equation S10 as a result of the initial rate (v_{max}) measured and known $[MA]_0$.

$$\text{rate} = k_s[BI][MA] \qquad \textbf{Equation S7}$$

$$\text{rate} = k_s^{'}[MA] \qquad \textbf{Equation S8}$$

$$\text{Initial rate } (v_{max}) = k_s^{'}[MA]_0 \qquad \textbf{Equation S9}$$

$$k_s^{'} = v_{max}/[MA]_0 \qquad \textbf{Equation S10}$$

To validate this approach, four kinetic experiments between pyridine-2-carboxyaldehyde **14** and *trans*-chalcone **17** catalyzed by the NHC derived from *N*-C_6F_5 triazolium precatalyst **16** were designed and conducted under consistent reaction conditions. The initial concentrations of aldehyde (0.3 M) and catalyst (5 mM) were kept constant in all four experiments, with only the starting concentration of *trans*-chalcone **17** varying from 0.2 M to 0.5 M. If Equation S9 is valid, then these experiments are expected to give a consistent value for $k_s^{'}$, calculated using Equation S10. The reactions were monitored using ^1H NMR spectra, with representative NMR spectra over the course of the experiment with 0.2 M initial concentration of *trans*-chalcone **17** given in Figure S4.

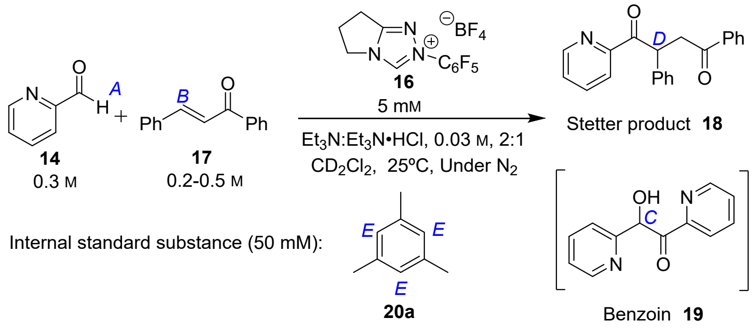

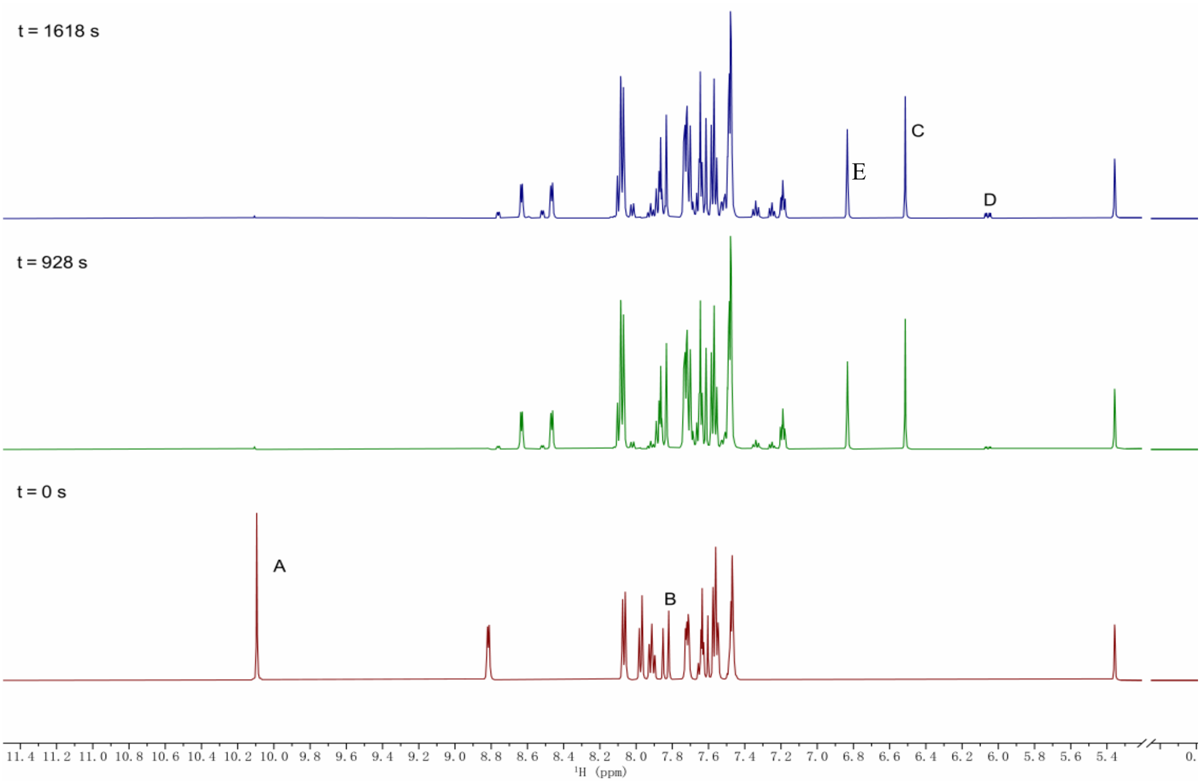

Figure S4. Representative ^{1}H NMR spectra (500 MHz) for reaction of pyridine-2-carboxaldehyde **14** (0.3 M) and (*E*)-chalcone **17** (0.2 M) catalysed by the NHC derived from *N*-C_6F_5 precatalyst **16** (5 mM) in NEt_3:NEt_3·HCl (2:1, 0.03 M) in CD_2Cl_2 at 25 °C. A = Aldehyde **14** C*H*O, B = (*E*)-Chalcone **17** C*H*, C = Benzoin **19** C*H*, D = Stetter product **18** C*H*, E = 1,3,5-trimethylbenzene **20a** Ph*H*.

Before initiation of the reaction by triethylamine buffer (t = 0 min), two signals at 10.09 ppm (**A**) and 7.82 ppm (**B**) appear in the ^{1}H NMR spectrum, which respectively belongs to C*H*O of pyridine-2-carboxaldehyde **14** and C*H* of *trans*-chalcone **17**. The two singlet signals at 6.51 ppm (**C**) and 6.81 ppm (**E**) respectively belong to C*H* of Benzoin **19** and aromatic proton of 1,3,5-trimethylbenzene. The ^{1}H NMR resonance of C*H* of Stetter product **18** appears at 6.06 ppm (**D**). Using these signals, the concentration of Benzoin **19** at different times was determined relative to the internal standard (1,3,5-trimethylbenzene, 50 mM) of signal **E** (Equation S11). Using the same method, the concentration of Stetter product **18** at different times can be also calculated according to Equation S12. The concentrations of Stetter product **18** and Benzoin **19** at different times for these four experiments are summarized in Table S1.

$$[Benzoin]_{(t=x)} = 3 * \frac{A_{C(t=x)}}{A_E} \times 0.05$$ **Equation S11**

$$[Stetter]_{(t=x)} = 3 * \frac{A_{D(t=x)}}{A_E} \times 0.05$$ **Equation S12**

Table S1. The concentrations of Stetter product **18** and benzoin **19** for the reaction of (*E*)-chalcone **17** to

pyridine-2-carboxaldehyde **14** (0.3 M), catalyzed by the NHC derived from C_6F_5 triazolium precatalyst **16** (5 mM) in 0.03 M triethylamine buffer (Et_3N:$Et_3N\cdot HCl$, 2:1) in CD_2Cl_2 at 25 °C.

**[Aldehyde]$_0$ / M**	**[MA]$_0$ / M**	**Time / s**	**[Benzoin] / M**	**[Stetter] / M**
0.3	0.2	0	0	0
		376	0.147935	0.004278
		514	0.148756	0.006822
		652	0.145405	0.008926
		790	0.142775	0.010948
		928	0.140709	0.012947
		1066	0.138485	0.014956
		1204	0.136006	0.016924
		1342	0.133589	0.018672
		1480	0.131634	0.020634
		1618	0.129926	0.022653
0.3	0.3	0	0	0
		354	0.145872	0.006911
		492	0.14543	0.01051
		630	0.142321	0.013688
		768	0.13869	0.016711
		906	0.135644	0.019519
		1044	0.132524	0.02236
		1182	0.129968	0.025034
		1320	0.127087	0.027841
		1458	0.124659	0.030722
		1596	0.121943	0.032216
0.3	0.4	0	0	0
		399	0.142585	0.011396
		537	0.141942	0.016337
		675	0.137663	0.020507
		813	0.13457	0.024626
		951	0.131276	0.028027
		1089	0.127828	0.031992
		1227	0.125078	0.035421
		1365	0.122123	0.038298
		1503	0.119984	0.042946
		1641	0.116771	0.044603
0.3	0.5	0	0	0
		393	0.141423	0.015731
		531	0.139559	0.01995
		669	0.133812	0.026328

		807	0.128851	0.030448
		945	0.123632	0.034797
		1083	0.122787	0.040144
		1221	0.119518	0.04478
		1359	0.11644	0.049178
		1497	0.113227	0.054368
		1635	0.110603	0.052514

Plotting the concentration of Stetter product **18** ([Stetter]) against time, allowed four different initial rates (1.16, 2.20, 2.85 and 3.60 × 10^{-5} M s^{-1}) to be obtained, with similar pseudo first-order rate constants $k_s^{'}$ (7.15, 7.10, 7.13 and 7.20 × 10^{-5} s^{-1}) calculated via Equation S10 (Scheme S5). The standard deviation (SD = sqrt[($\sum(x_i-\bar{x})$^2)/n)]) is also used for average value of rate constant. The observed rate constants are similar, validating the method proposed to quantify the reactivity of a series of Michael acceptors reacting with the BI.

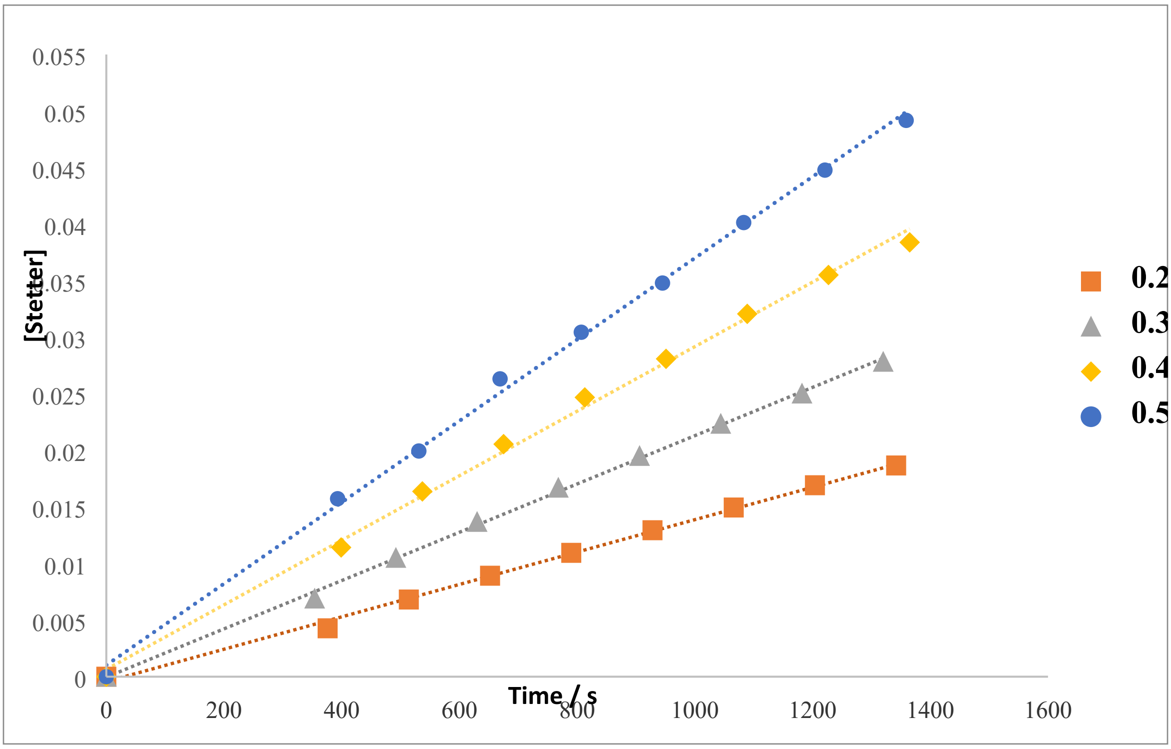

Entry	$[Ald]_0$/M	$[MA]_0$/M	[Cat]/mM	v_{max} (×10^{-5} M s^{-1})	$k_s^{'}$(×10^{-5} s^{-1})	Average
**1**	0.3	0.2	5	1.16	7.15	
**2**	0.3	0.3	5	2.20	7.10	7.15±0.04
**3**	0.3	0.4	5	2.85	7.13	

**4**	0.3	0.5	5	3.60	7.20

Scheme S5. Rate constant k_s' for the four Stetter reactions catalysed by the NHC derived from the *N*-C_6F_5 triazolium catalyst **16** (5 mM) at 25 °C, with different initial concentrations of *trans*-chalcone **17** (0.2 M, 0.3 M, 0.4 M and 0.5 M).

General experimental procedure B

In a nitrogen-protected NMR tube, the *N*-C_6F_5 triazolium salt **16** (1.35-1.37 mg, using 0.01 mg analytical balance) and 2-pyridinecarboxaldehyde **14** (21 μL) were added. And then the Michael acceptors was also added into the reaction system before adding 650 μL CD_2Cl_2. The reaction was initiated by adding 100 μL CD_2Cl_2 solution of NEt_3 (0.150 M), $Et_3N{\cdot}HCl$ (0.075 M) and the internal standard (1,3,5-trimethylbenzene **20a**, 0.375 M). This gave an overall 2-pyridinecarboxaldehyde **14** of 0.3 M, NHC precatalyst **16** concentration of 5 mM, 1,3,5-trimethylbenzene **20a** concentration of 50 mM and a total buffer concentration of 0.03 M. The reaction was monitored by temperature controlled (25 °C) ^1H NMR spectroscopy on a Bruker Avance 500 MHz NMR spectrometer. Spectra were taken at 1 or 2 minutes intervals over 0.5-1 hours. Over the course of this reaction, it was possible to observe the changing concentration of benzoin **19** and the corresponding Stetter product. Then, the same procedure was also conducted for other initial concentrations of Michael acceptors.

Entry 1

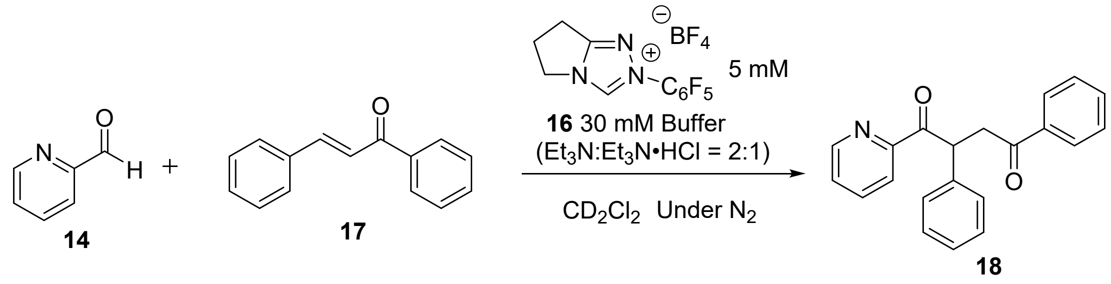

The reaction of pyridine-2-carboxaldehyde **14** and (*E*)-chalcone **17** catalysed by C_6F_5 triazolium salt **16** in triethylamine buffer was monitored using ^1H NMR spectra, with representative NMR spectra over the course of the experiment given in Figure S5.

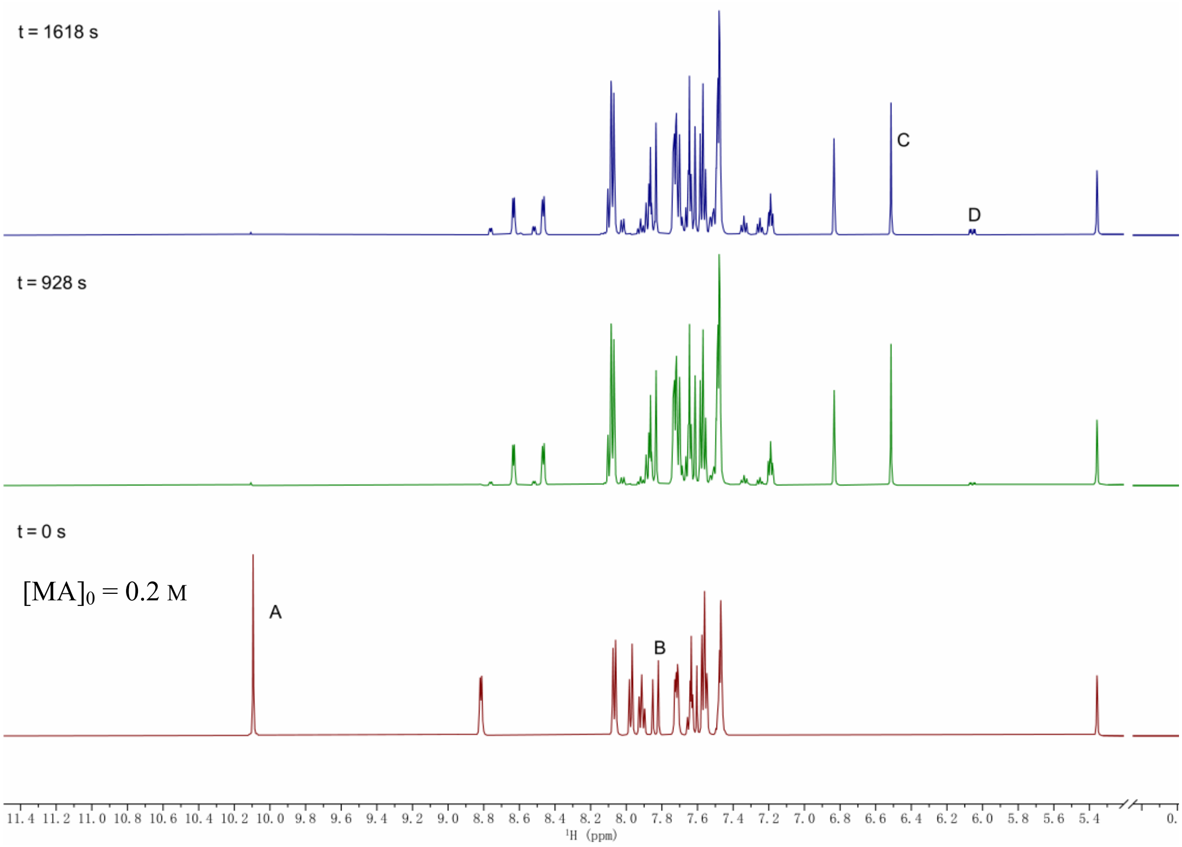
t = 1618 s
C
D
t = 928 s
t = 0 s
[MA]$_0$ = 0.2 M
A
B
^{1}H (ppm)

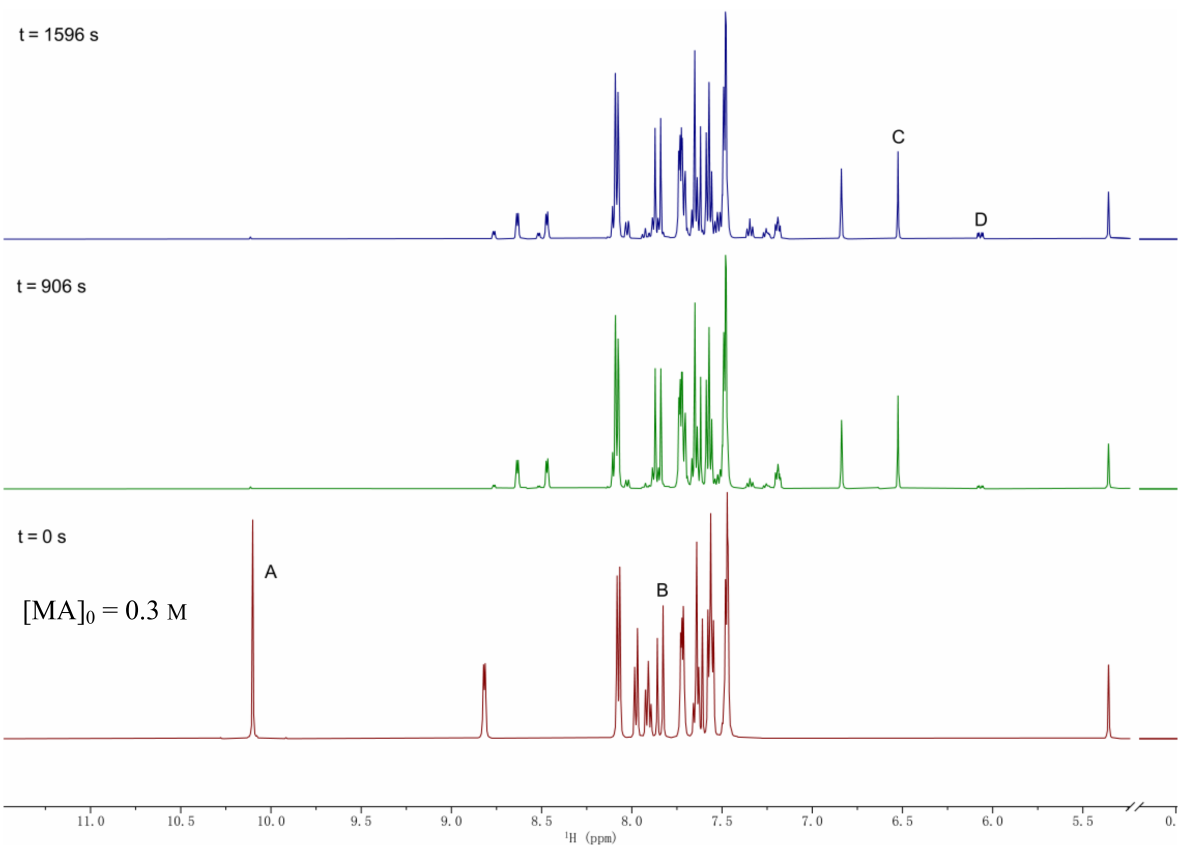
t = 1596 s
C
D
t = 906 s
t = 0 s
[MA]$_0$ = 0.3 M
A
B
^{1}H (ppm)

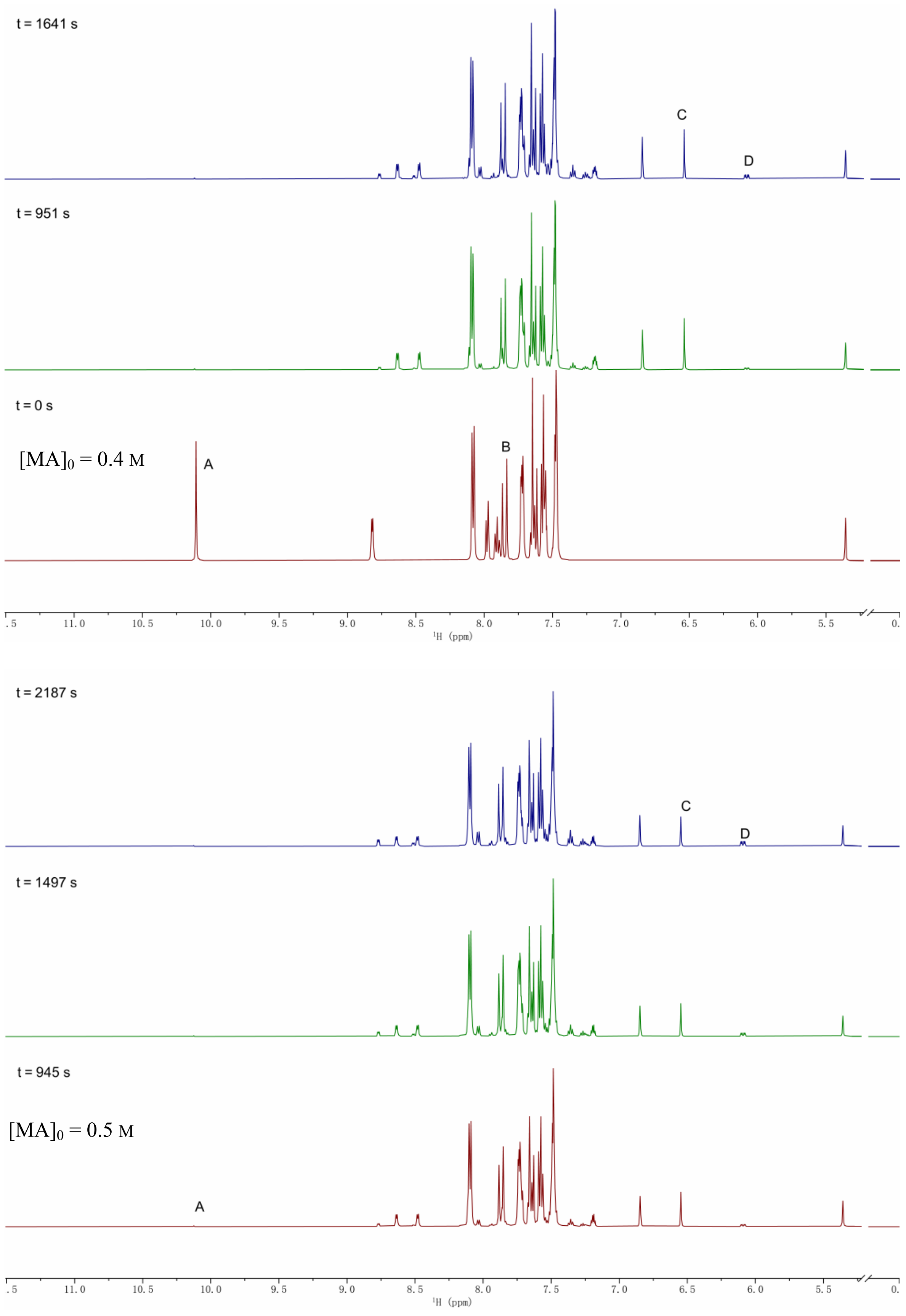

Figure S5. Representative ^{1}H NMR spectra (500 MHz) for reaction of pyridine-2-carboxaldehyde **14** (0.3 M) and (*E*)-chalcone **17** (0.2 M, 0.3 M, 0.4 M and 0.5 M) catalysed by *N*-C_6F_5 NHC precursor **16** (5 mM) in

NEt_3:NEt_3·HCl (2:1, 0.03 M) in CD_2Cl_2 at 25 °C. A = Aldehyde C*H*O, B = Chalcone C*H*, C = Benzoin C*H*, D = Stetter product C*H*.

Table S1. Reaction data and initial rates of Stetter product formation from (*E*)-chalcone **17**, catalyzed by C_6F_5 triazolium precatalyst **16** (5 mM) in 0.03 M triethylamine buffer (Et_3N:Et_3N·HCl, 2:1) in CD_2Cl_2 at 25 °C.

**[Aldehyde]$_0$ / M**	**[MA]$_0$ / M**	**Time / s**	**[Benzoin] / M**	**[Stetter] / M**	**v_{max} / 10^{-5} M s^{-1}**	**k_s' / 10^{-5} s^{-1}**	**Average / 10^{-5} s^{-1}**
0.3	0.2	0	0	0	1.16	7.15	7.15±0.04
		376	0.147935	0.004278			
		514	0.148756	0.006822			
		652	0.145405	0.008926			
		790	0.142775	0.010948			
		928	0.140709	0.012947			
		1066	0.138485	0.014956			
		1204	0.136006	0.016924			
		1342	0.133589	0.018672			
		1480	0.131634	0.020634			
		1618	0.129926	0.022653			
0.3	0.3	0	0	0	2.13	7.10	
		354	0.145872	0.006911			
		492	0.14543	0.01051			
		630	0.142321	0.013688			
		768	0.13869	0.016711			
		906	0.135644	0.019519			
		1044	0.132524	0.02236			
		1182	0.129968	0.025034			
		1320	0.127087	0.027841			
		1458	0.124659	0.030722			
		1596	0.121943	0.032216			
0.3	0.4	0	0	0	2.85	7.13	
		399	0.142585	0.011396			
		537	0.141942	0.016337			
		675	0.137663	0.020507			
		813	0.13457	0.024626			
		951	0.131276	0.028027			
		1089	0.127828	0.031992			
		1227	0.125078	0.035421			
		1365	0.122123	0.038298			
		1503	0.119984	0.042946			
		1641	0.116771	0.044603			
0.3	0.5	0	0	0	3.60	7.20	

		393	0.147423	0.015731			
		531	0.141559	0.01995			
		669	0.135812	0.026328			
		807	0.129851	0.030448			
		945	0.123632	0.034797			
		1083	0.122787	0.040144			
		1221	0.119518	0.04478			
		1359	0.11644	0.049178			
		1497	0.113227	0.054368			
		1635	0.110603	0.052514			

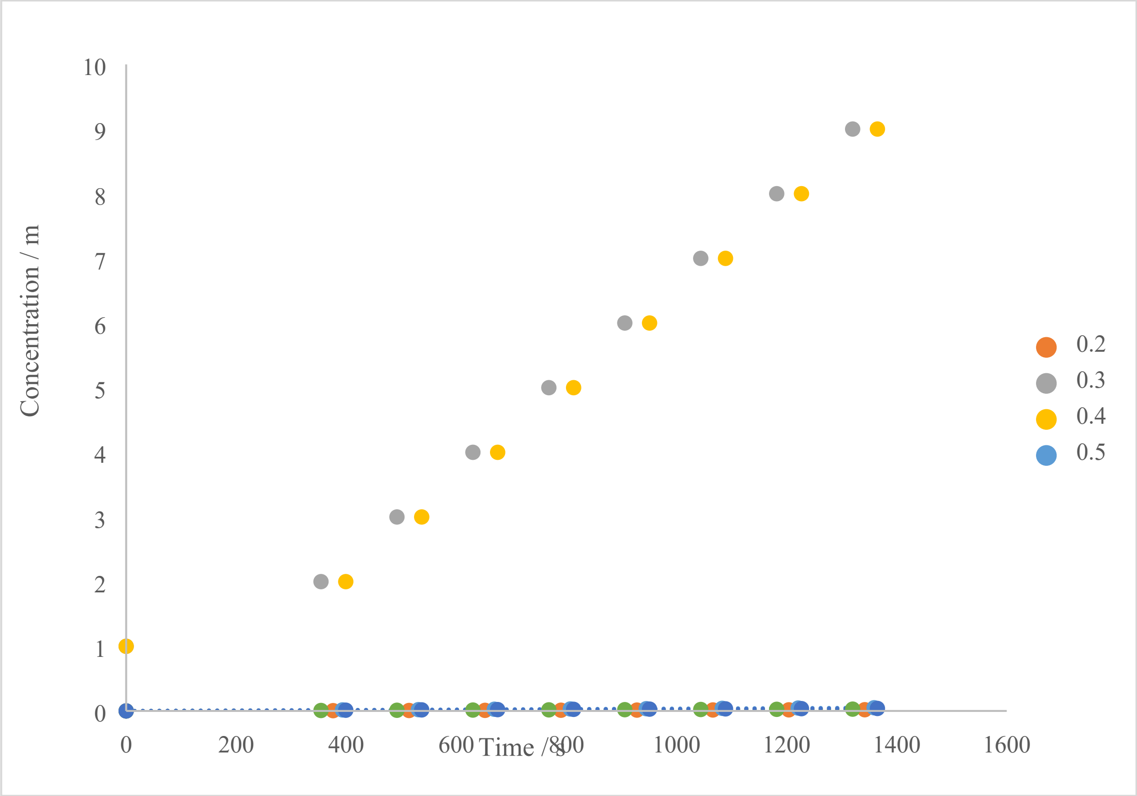

Figure S6. Plots of Stetter product concentration against time for the C_6F_5 triazolium precatalyst **16** (5 mM) catalysed Stetter reaction, at initial (*E*)-chalcone **17** concentrations 0.2 M, 0.3 M, 0.4 M, 0.5 M.

Entry 2

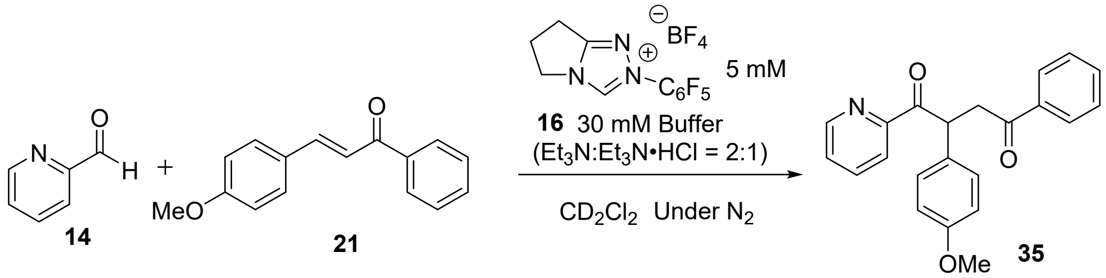

The reaction of pyridine-2-carboxaldehyde **14** and chalcone **21** catalysed by C_6F_5 triazolium salt **16** in

triethylamine buffer was monitored using ^{1}H NMR spectra, with representative NMR spectra over the course of the experiment given in Figure S7.

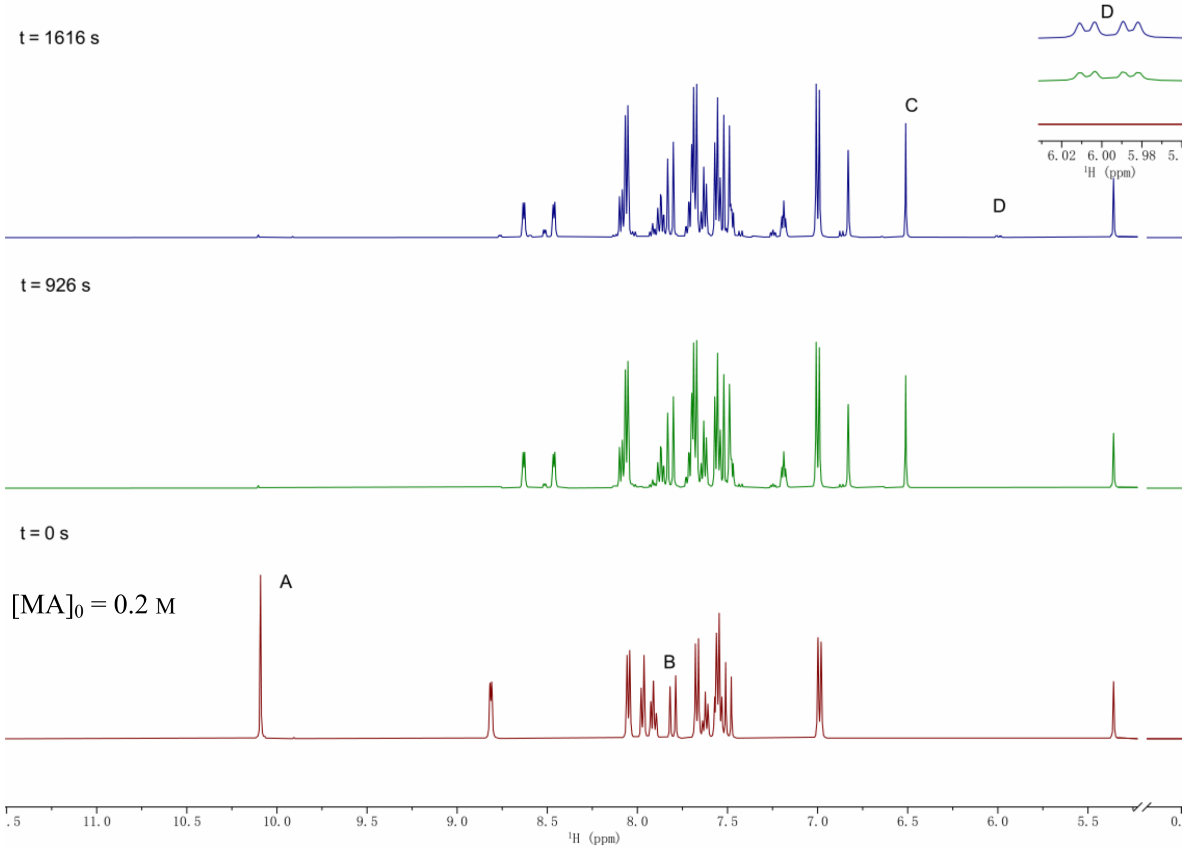

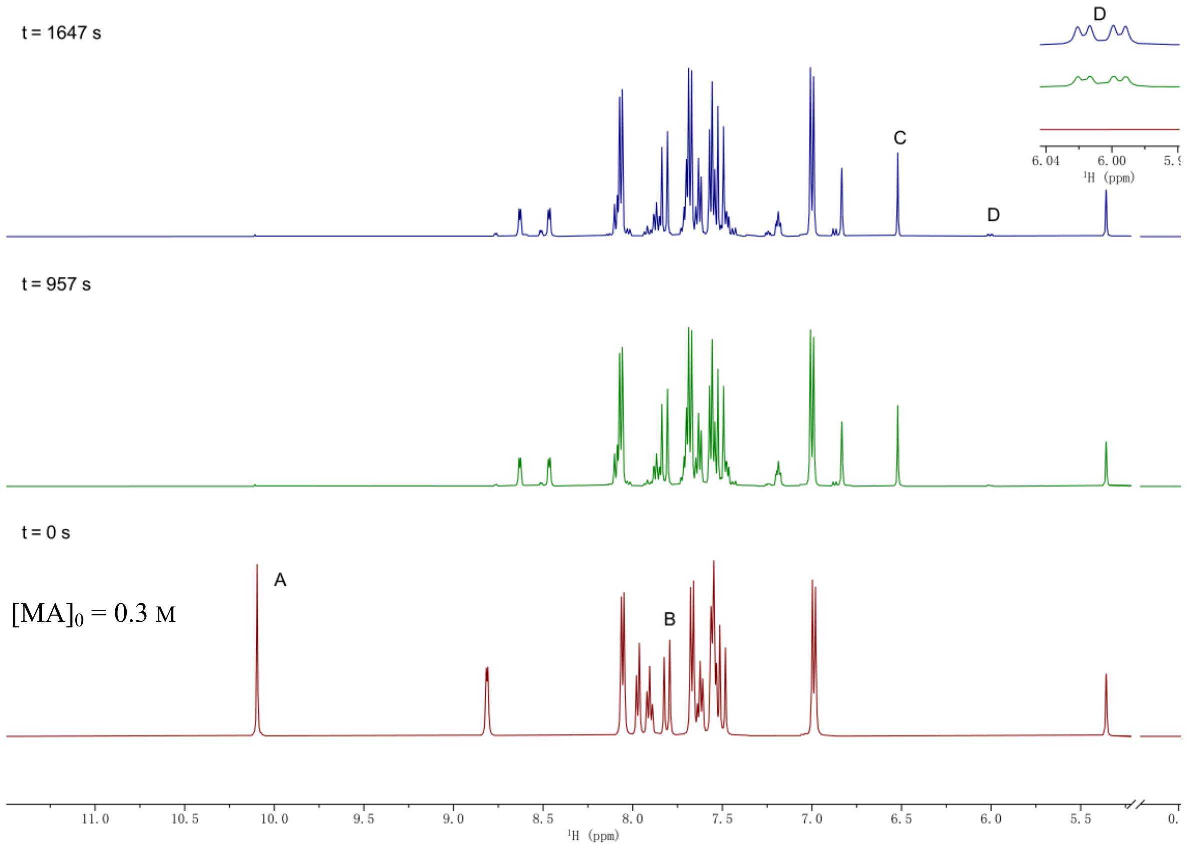

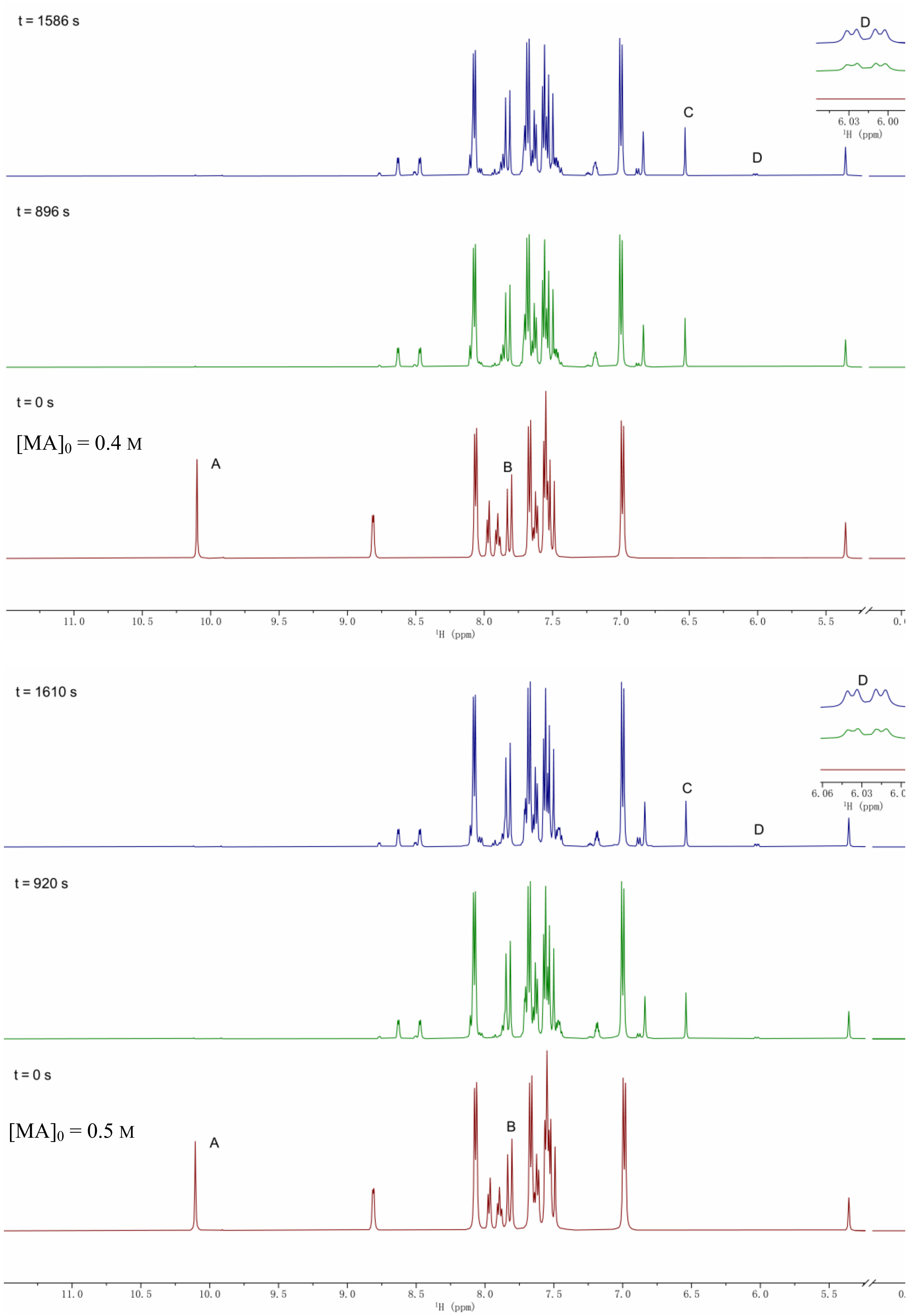

Figure S7. Representative ^{1}H NMR spectra (500 MHz) for reaction of pyridine-2-carboxaldehyde **14** (0.3 M) and chalcone **21** (0.2 M, 0.3 M, 0.4 M and 0.5 M) catalysed by *N*-C_6F_5 NHC precursor **16** (5 mM) in

NEt_3:NEt_3·HCl (2:1, 0.03 M) in CD_2Cl_2 at 25 °C. A = Aldehyde C*H*O, B = Chalcone C*H*, C = Benzoin C*H*, D = Stetter product C*H*.

Table S2. Reaction data and initial rates of Stetter product formation from (E)-3-(4-methoxylphenyl)-1-phenylprop-2-en-1-one **21**, catalyzed by C_6F_5 triazolium precatalyst **16** (5 mM) in 0.03 M triethylamine buffer (Et_3N:Et_3N·HCl, 2:1) in CD_2Cl_2 at 25 °C.

**[Aldehyde]$_0$ / M**	**[MA]$_0$ / M**	**Time / s**	**[Benzoin] / M**	**[Stetter] / M**	v_{max} **/ 10^{-5} M s^{-1}**	k_s' **/ 10^{-5} s^{-1}**	**Average / 10^{-5} s^{-1}**
0.3	0.2	0	0	0	0.896	4.48	4.46±0.08
		374	0.144052	0.002913			
		512	0.144674	0.004076			
		650	0.143196	0.005377			
		788	0.140054	0.006395			
		926	0.138933	0.008033			
		1064	0.13653	0.008999			
		1202	0.135414	0.010886			
		1340	0.131963	0.011688			
		1478	0.130151	0.012288			
		1616	0.128955	0.013710			
0.3	0.3	0	0	0	1.34	4.47	
		405	0.141013	0.004963			
		543	0.142441	0.006993			
		681	0.140262	0.008877			
		819	0.137979	0.011023			
		957	0.135463	0.013081			
		1095	0.132853	0.014775			
		1233	0.130794	0.016284			
		1371	0.129339	0.01796			
		1509	0.128066	0.020078			
		1647	0.125505	0.020862			
0.3	0.4	0	0	0	1.73	4.33	
		344	0.144552	0.005556			
		482	0.143905	0.00854			
		620	0.140791	0.011348			
		758	0.138236	0.013572			
		896	0.134649	0.01483			
		1034	0.132409	0.017427			
		1172	0.13073	0.019986			
		1310	0.127977	0.023256			
		1448	0.126542	0.025800			
		1586	0.123611	0.027546			
0.3	0.5	0	0	0	2.28	4.56	

		368	0.139338	0.008146			
		506	0.138887	0.012672			
		644	0.134858	0.013839			
		782	0.132268	0.018449			
		920	0.12972	0.022035			
		1058	0.126424	0.024117			
		1196	0.123591	0.026416			
		1334	0.122036	0.030977			
		1472	0.118336	0.030940			
		1610	0.11673	0.035461			

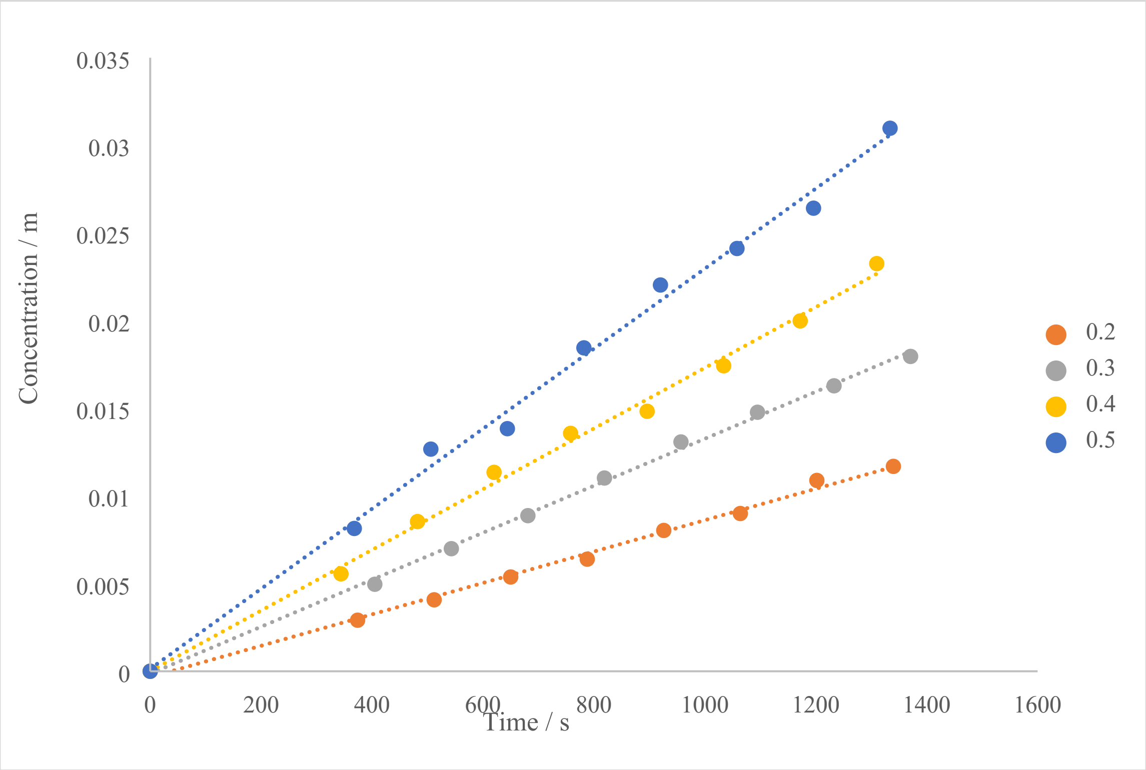

Figure S8. Plots of Stetter product concentration against time for the C_6F_5 triazolium precatalyst **16** (5 mM) catalysed Stetter reaction, at initial (E)-3-(4-methoxylphenyl)-1-phenylprop-2-en-1-one **21** concentrations 0.2 M, 0.3 M, 0.4 M, 0.5 M.

Entry 3

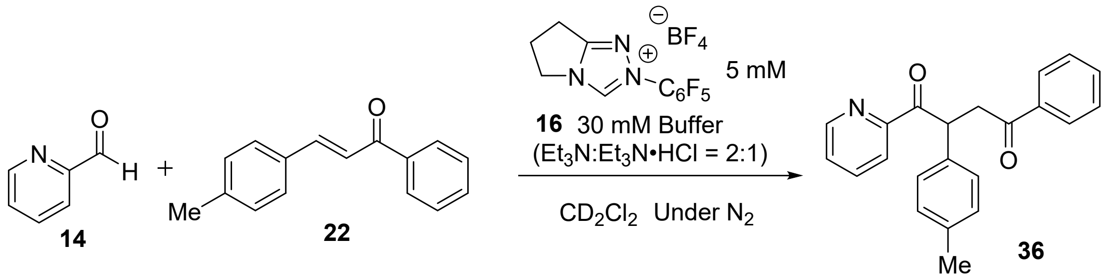

The reaction of pyridine-2-carboxaldehyde **14** and chalcone **22** catalysed by C_6F_5 triazolium salt **16** in

triethylamine buffer was monitored using ^{1}H NMR spectra, with representative NMR spectra over the course of the experiment given in Figure S9.

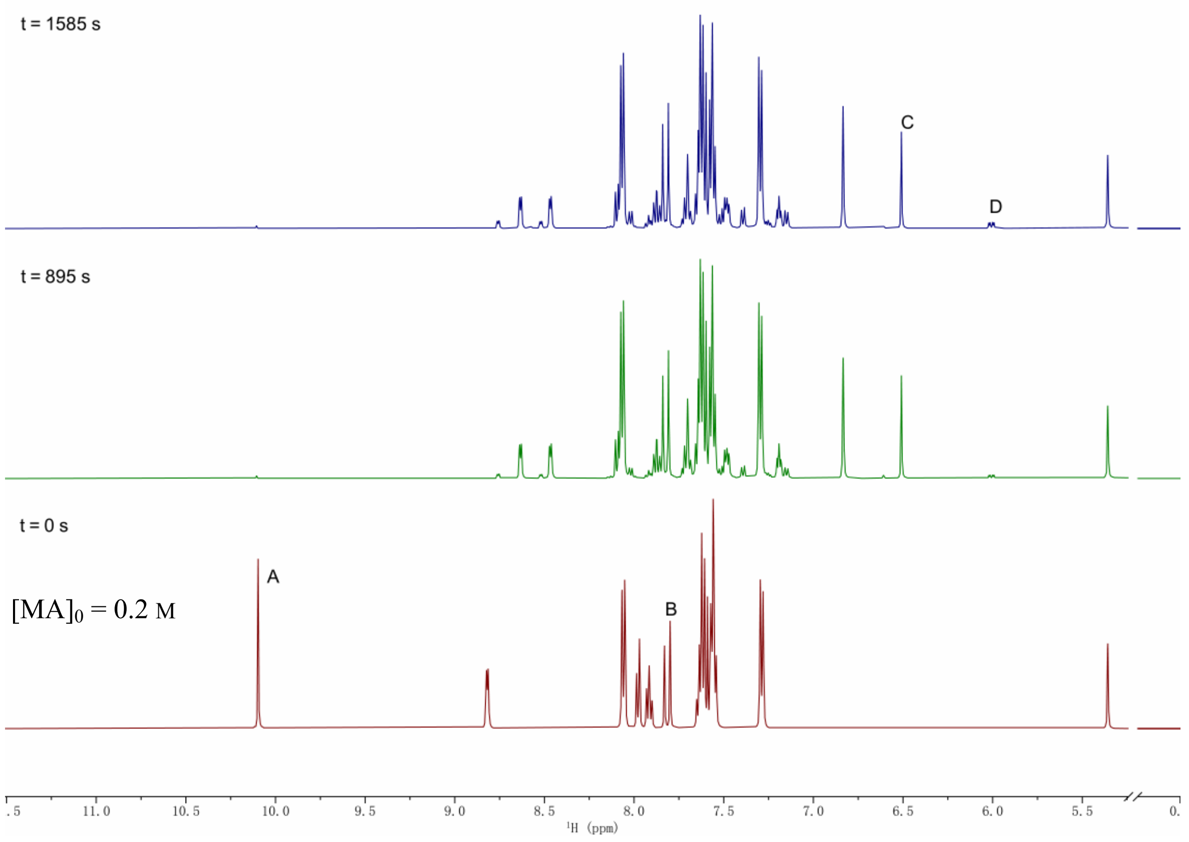

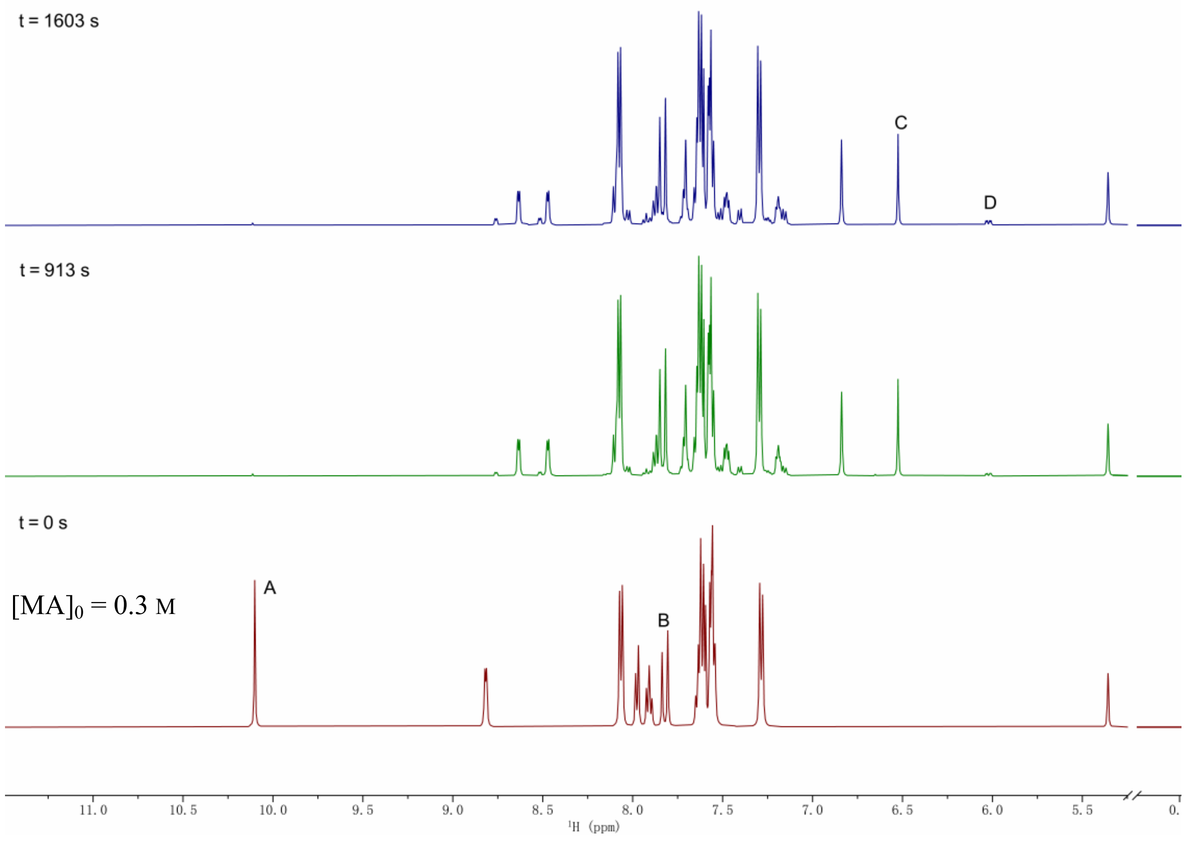

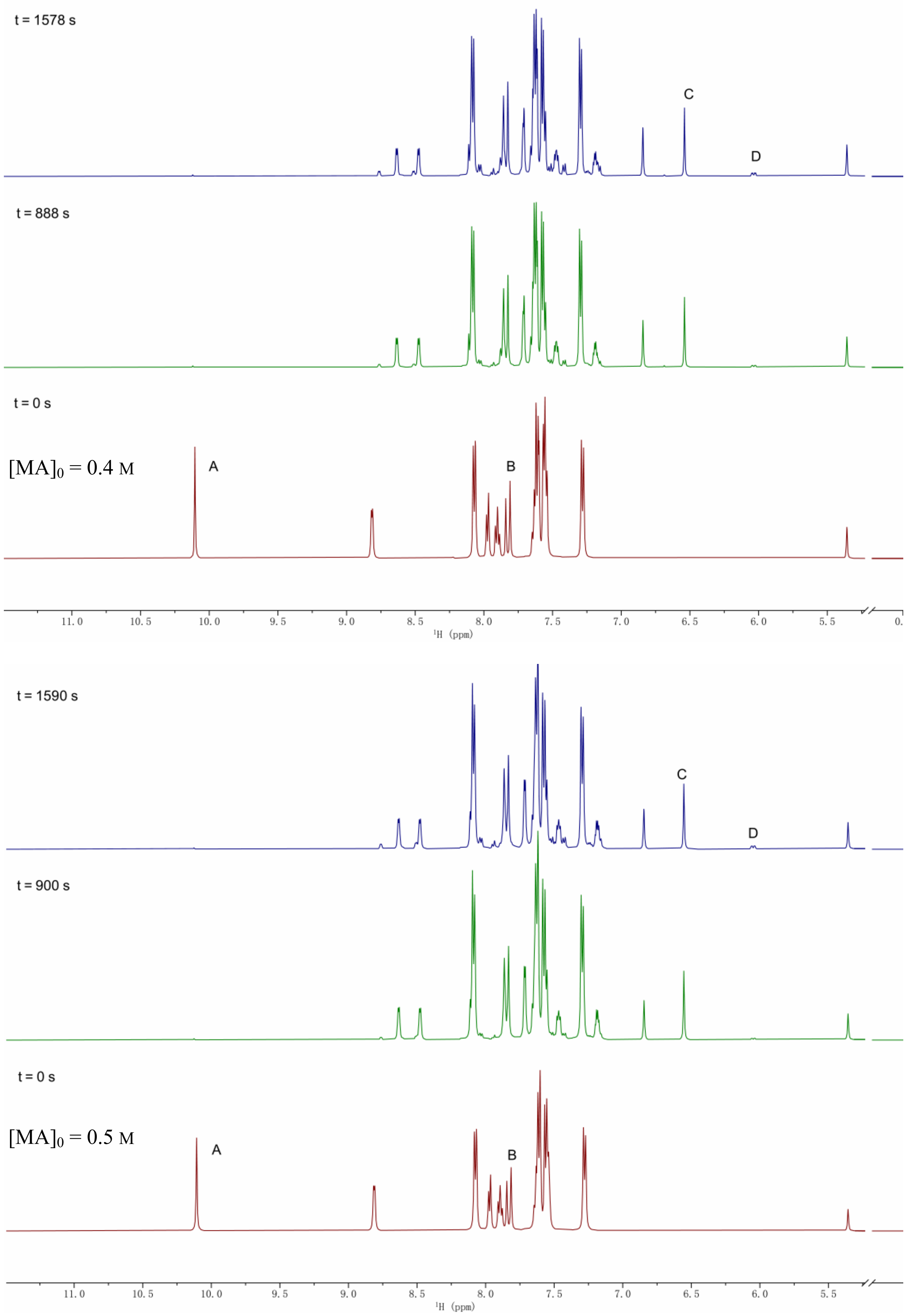

Figure S9. Representative ^{1}H NMR spectra (500 MHz) for reaction of pyridine-2-carboxaldehyde **14** (0.3 M) and chalcone **22** (0.2 M, 0.3 M, 0.4 M and 0.5 M) catalysed by *N*-C_6F_5 NHC precursor **16** (5 mM) in

NEt_3:NEt_3·HCl (2:1, 0.03 M) in CD_2Cl_2 at 25 °C. A = Aldehyde C*H*O, B = Chalcone C*H*, C = Benzoin C*H*, D = Stetter product C*H*.

Table S3. Reaction data and initial rates of Stetter product formation from (E)-3-(4-methylphenyl)-1-phenylprop-2-en-1-one **22**, catalyzed by C_6F_5 triazolium precatalyst **16** (5 mM) in 0.03 M triethylamine buffer (Et_3N:Et_3N·HCl, 2:1) in CD_2Cl_2 at 25 °C.

[Aldehyde]$_0$ / M	[MA]$_0$ / M	Time / s	[Benzoin] / M	[Stetter] / M	v_{max} / 10^{-5} M s^{-1}	k_s' / 10^{-5} s^{-1}	Average / 10^{-5} s^{-1}
0.3	0.2	0	0	0	1.11	5.55	5.56±0.17
		343	0.142656	0.002817			
		481	0.142887	0.004762			
		619	0.139959	0.006319			
		757	0.137149	0.007959			
		895	0.134173	0.009512			
		1033	0.131493	0.011049			
		1171	0.129299	0.012635			
		1309	0.126883	0.014223			
		1447	0.124633	0.015719			
		1585	0.122101	0.017030			
0.3	0.3	0	0	0	1.73	5.77	
		361	0.137574	0.004847			
		499	0.137979	0.008026			
		637	0.135437	0.010461			
		775	0.1329	0.012516			
		913	0.131026	0.015842			
		1051	0.128818	0.018122			
		1189	0.126708	0.01966			
		1327	0.124606	0.022296			
		1465	0.122820	0.024973			
		1603	0.120623	0.027133			
0.3	0.4	0	0	0	2.12	5.30	
		336	0.131438	0.004818			
		474	0.131894	0.008449			
		612	0.129543	0.011622			
		750	0.126288	0.015292			
		888	0.123759	0.018397			
		1026	0.123634	0.020476			
		1164	0.120126	0.024117			
		1302	0.119125	0.02635			
		1440	0.117947	0.029122			
		1578	0.115853	0.031596			
0.3	0.5	0	0	0	2.81	5.62	

		348	0.126734	0.008331			
		486	0.127413	0.01298			
		624	0.126832	0.015708			
		762	0.124003	0.021007			
		900	0.12381	0.02399			
		1038	0.120852	0.028617			
		1176	0.116699	0.033321			
		1314	0.118833	0.035773			
		1452	0.116889	0.039299			
		1590	0.115742	0.042881			

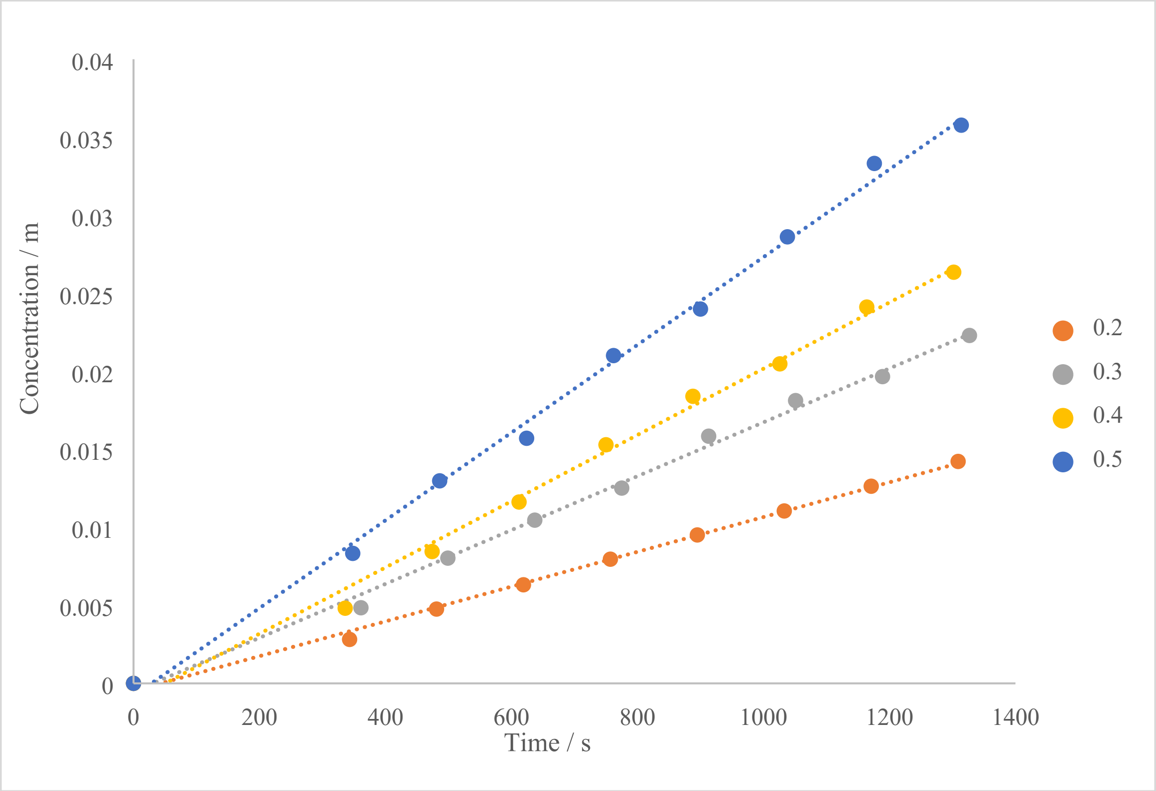

Figure S10. Plots of Stetter product concentration against time for the C_6F_5 triazolium precatalyst **16** (5 mM) catalysed Stetter reaction, at initial (E)-3-(4-methylphenyl)-1-phenylprop-2-en-1-one concentrations **22** 0.2 M, 0.3 M, 0.4 M, 0.5 M.

Entry 4

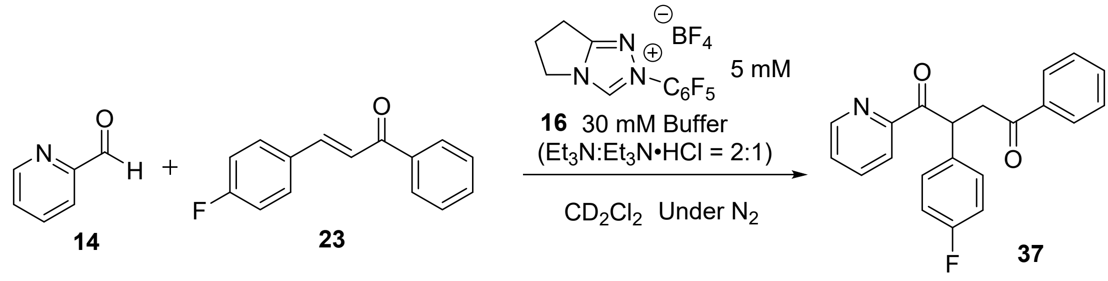

The reaction of pyridine-2-carboxaldehyde **14** and chalcone **23** catalysed by C_6F_5 triazolium salt **16** in triethylamine buffer was monitored using ^1H NMR spectra, with representative NMR spectra over the course of the experiment given in Figure S11.

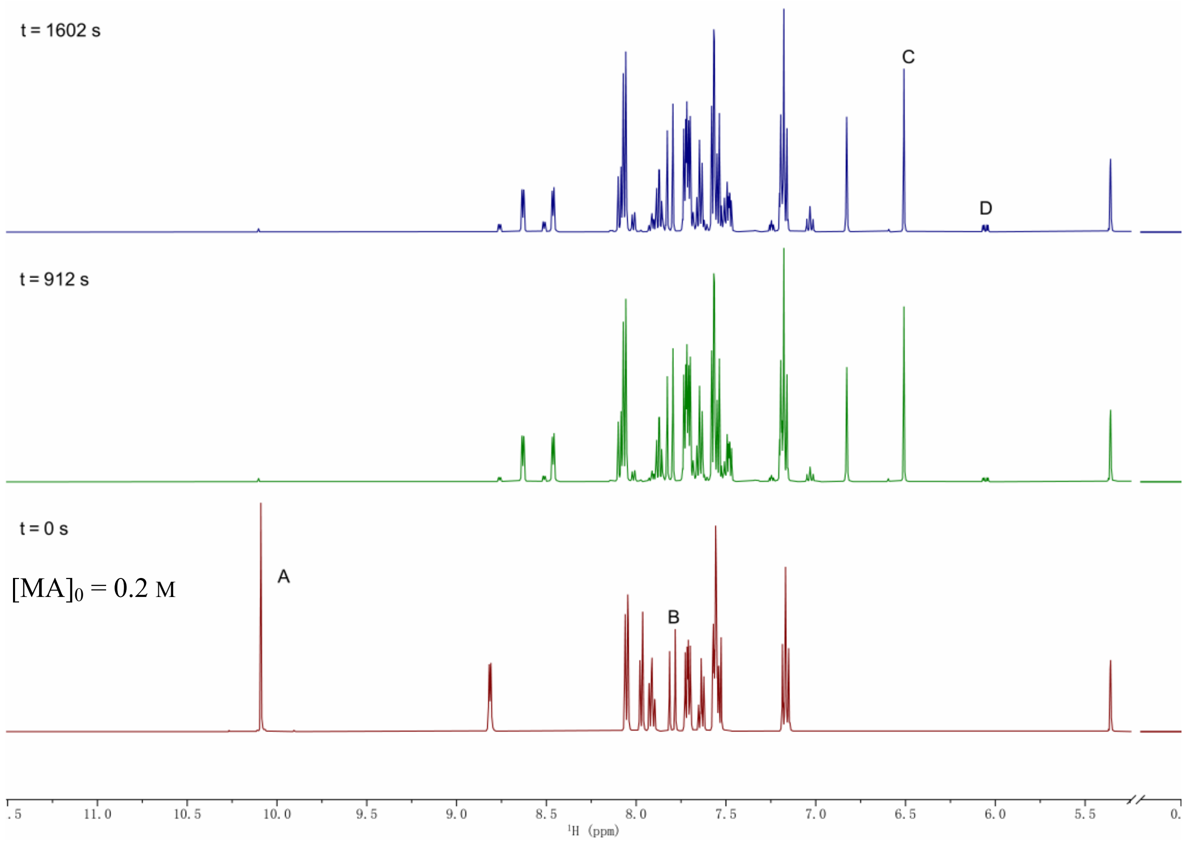

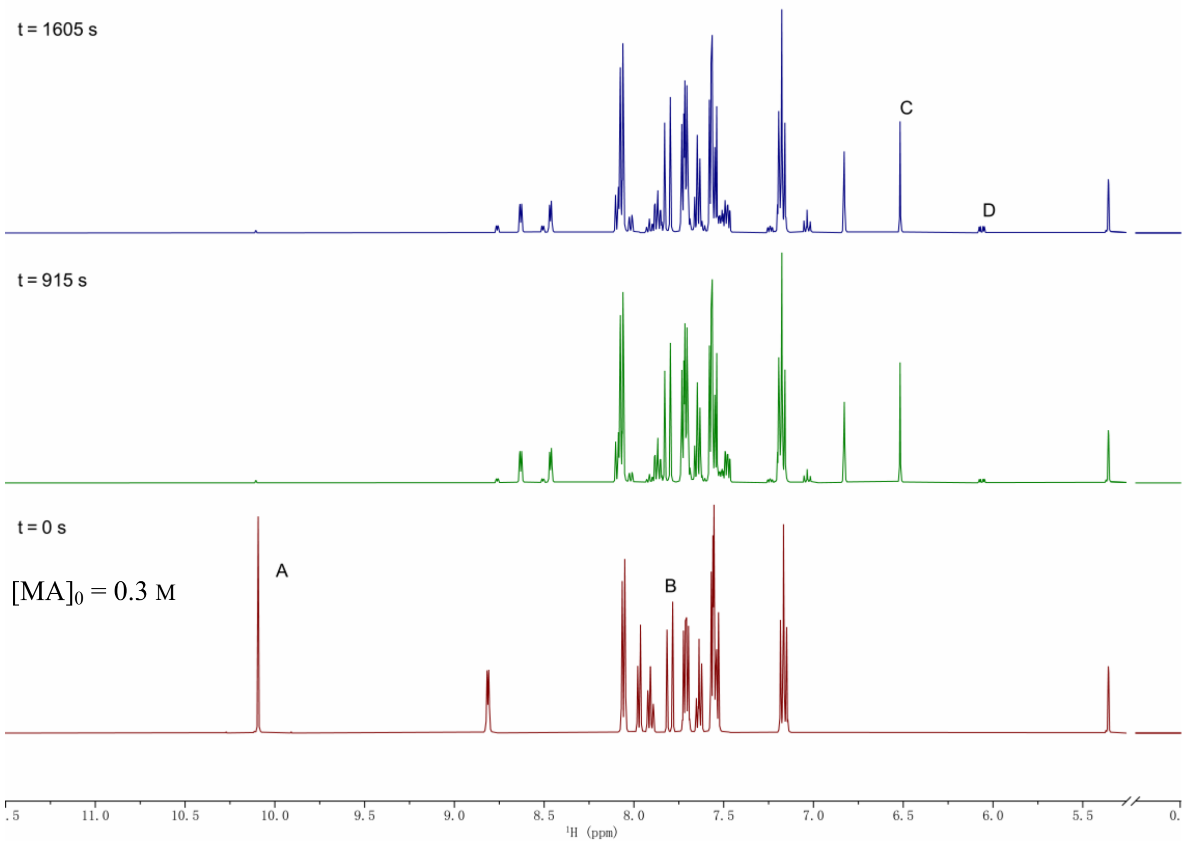

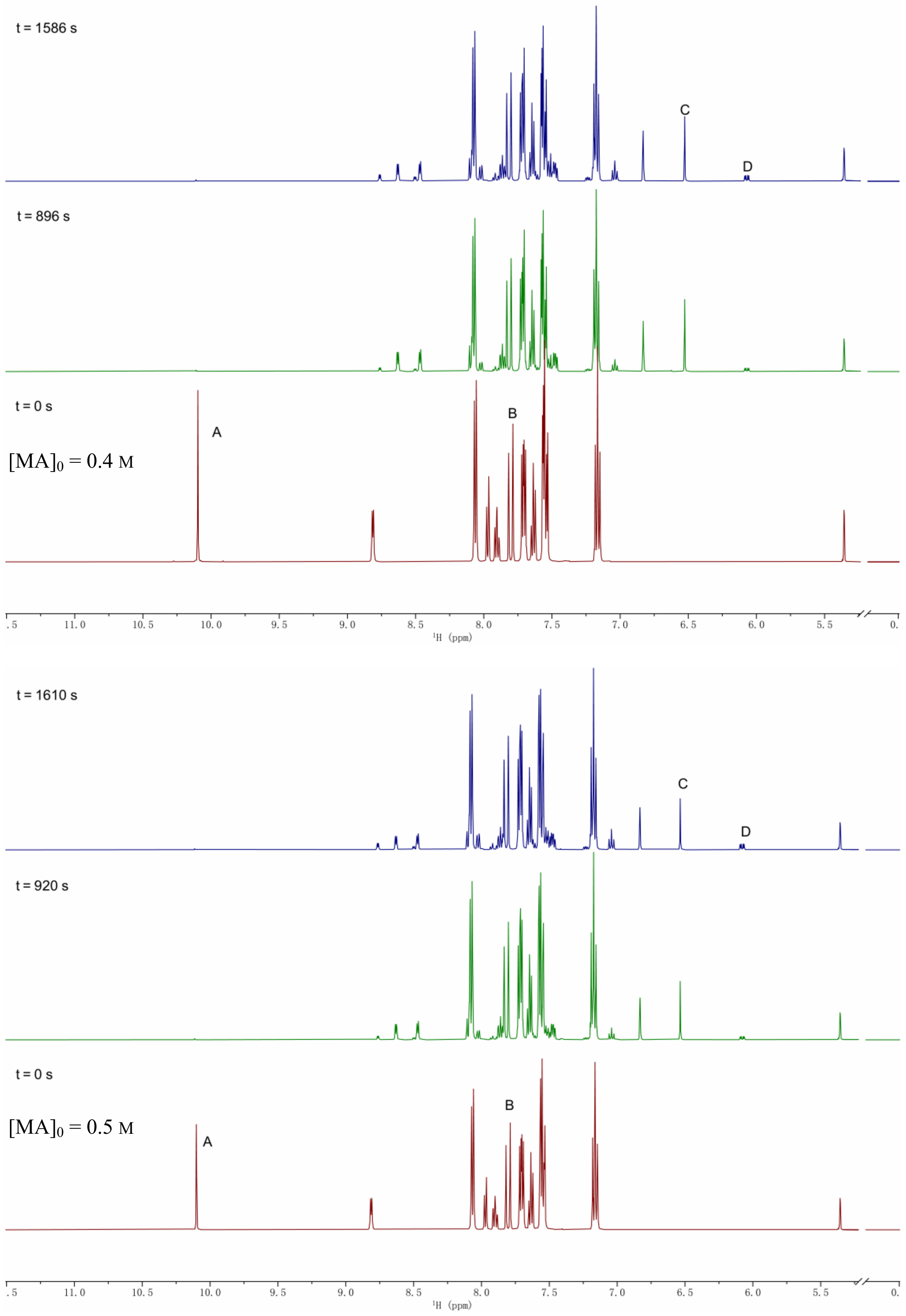

Figure S11. Representative ^{1}H NMR spectra (500 MHz) for reaction of pyridine-2-carboxaldehyde **14** (0.3 M) and chalcone **23** (0.2 M, 0.3 M, 0.4 M and 0.5 M) catalysed by *N*-C_6F_5 NHC precursor **16** (5 mM) in

NEt_3:NEt_3·HCl (2:1, 0.03 M) in CD_2Cl_2 at 25 °C. A = Aldehyde C*H*O, B = Chalcone C*H*, C = Benzoin C*H*, D = Stetter product C*H*.

Table S4. Reaction data and initial rates of Stetter product formation from (E)-3-(4-fluorophenyl)-1-phenylprop-2-en-1-one **23**, catalyzed by C_6F_5 triazolium precatalyst **16** (5 mM) in 0.03 M triethylamine buffer (Et_3N:Et_3N·HCl, 2:1) in CD_2Cl_2 at 25 °C.

[Aldehyde]$_0$ / M	[MA]$_0$ / M	Time / s	[Benzoin] / M	[Stetter] / M	v_{max} / 10^{-5} M s^{-1}	k_s' / 10^{-5} s^{-1}	Average / 10^{-5} s^{-1}
0.3	0.2	0	0	0	1.48	7.40	7.46±0.05
		360	0.143906	0.004092			
		498	0.144465	0.006562			
		636	0.141738	0.00877			
		774	0.139191	0.010817			
		912	0.136768	0.01309			
		1050	0.134383	0.015055			
		1188	0.132059	0.01704			
		1326	0.129726	0.019001			
		1464	0.127462	0.020569			
		1602	0.125285	0.022563			
0.3	0.3	0	0	0	2.26	7.53	
		363	0.140898	0.007114			
		501	0.140881	0.010863			
		639	0.137865	0.014197			
		777	0.135073	0.017236			
		915	0.132219	0.021038			
		1053	0.129545	0.023636			
		1191	0.126799	0.026408			
		1329	0.124622	0.029481			
		1467	0.122055	0.032128			
		1605	0.119735	0.035134			
0.3	0.4	0	0	0	2.99	7.48	
		344	0.138626	0.009637			
		482	0.138272	0.013999			
		620	0.134676	0.018674			
		758	0.131423	0.022717			
		896	0.127954	0.027041			
		1034	0.124822	0.031108			
		1172	0.121896	0.035026			
		1310	0.118704	0.03843			
		1448	0.116005	0.042301			
		1586	0.112819	0.04532			
0.3	0.5	0	0	0	3.71	7.42	

		368	0.137597	0.011043		
		506	0.13595	0.017973		
		644	0.132235	0.02265		
		782	0.128363	0.028217		
		920	0.124653	0.033833		
		1058	0.12114	0.03855		
		1196	0.117639	0.043438		
		1334	0.114487	0.048364		
		1472	0.111277	0.053356		
		1610	0.107759	0.057574		

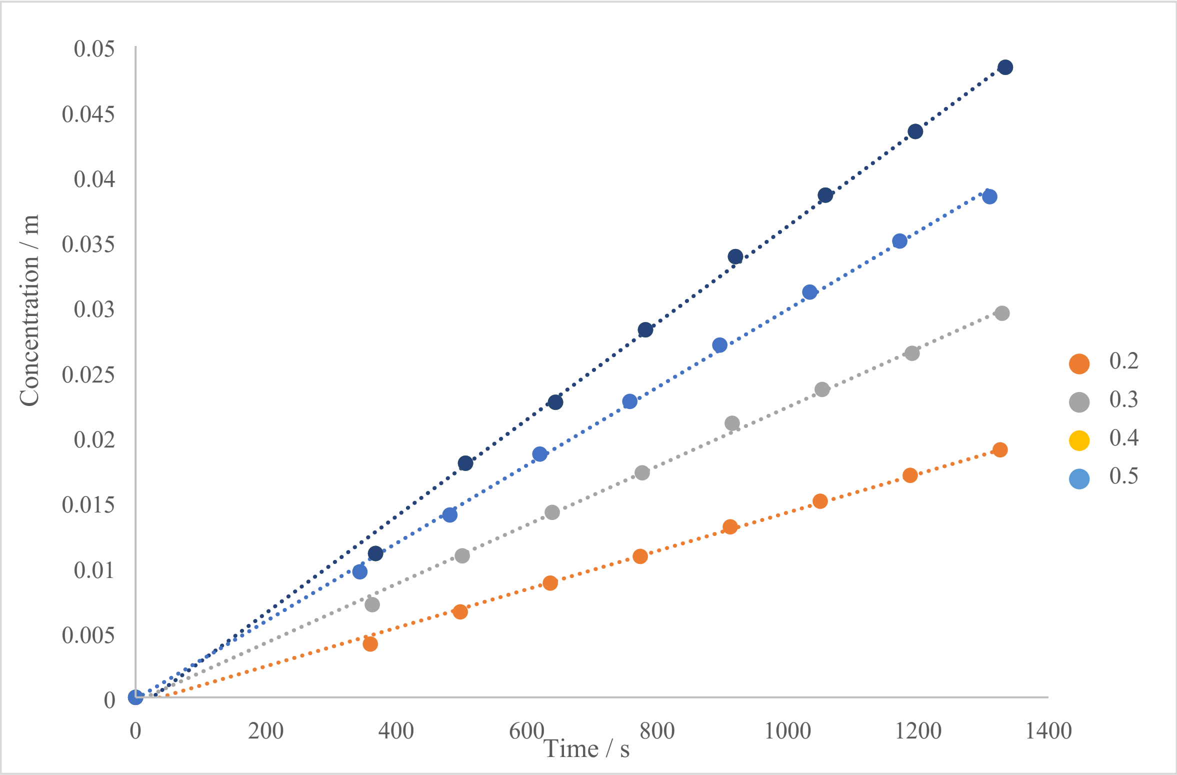

Figure S12. Plots of Stetter product concentration against time for the C_6F_5 triazolium precatalyst **16** (5 mM) catalysed Stetter reaction, at initial (E)-3-(4-fluorophenyl)-1-phenylprop-2-en-1-one **23** concentrations 0.2 M, 0.3 M, 0.4 M, 0.5 M.

Entry 5

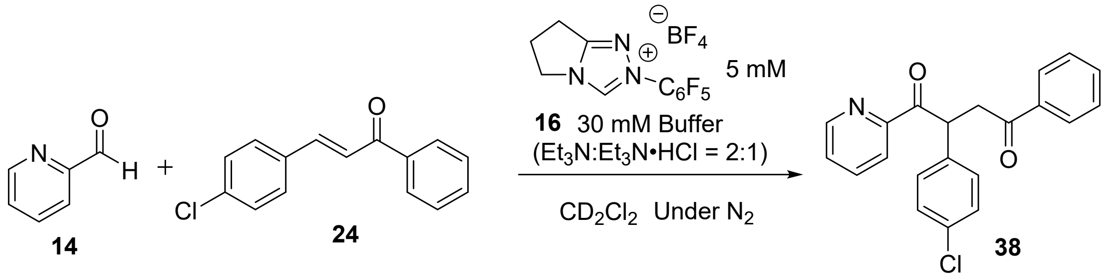

The reaction of pyridine-2-carboxaldehyde **14** and chalcone **24** catalysed by C_6F_5 triazolium salt **16** in

triethylamine buffer was monitored using ^{1}H NMR spectra, with representative NMR spectra over the course of the experiment given in Figure S13.

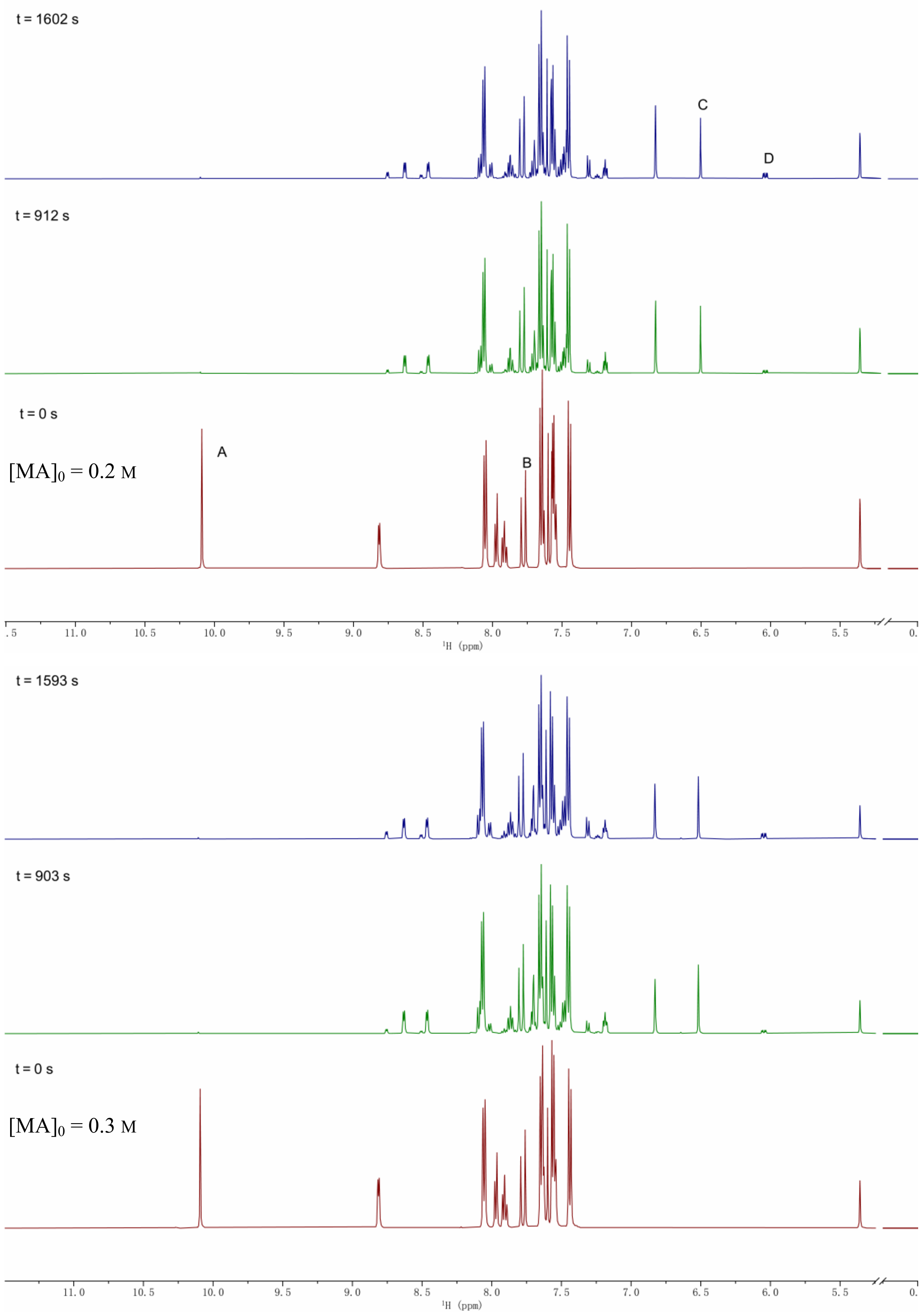

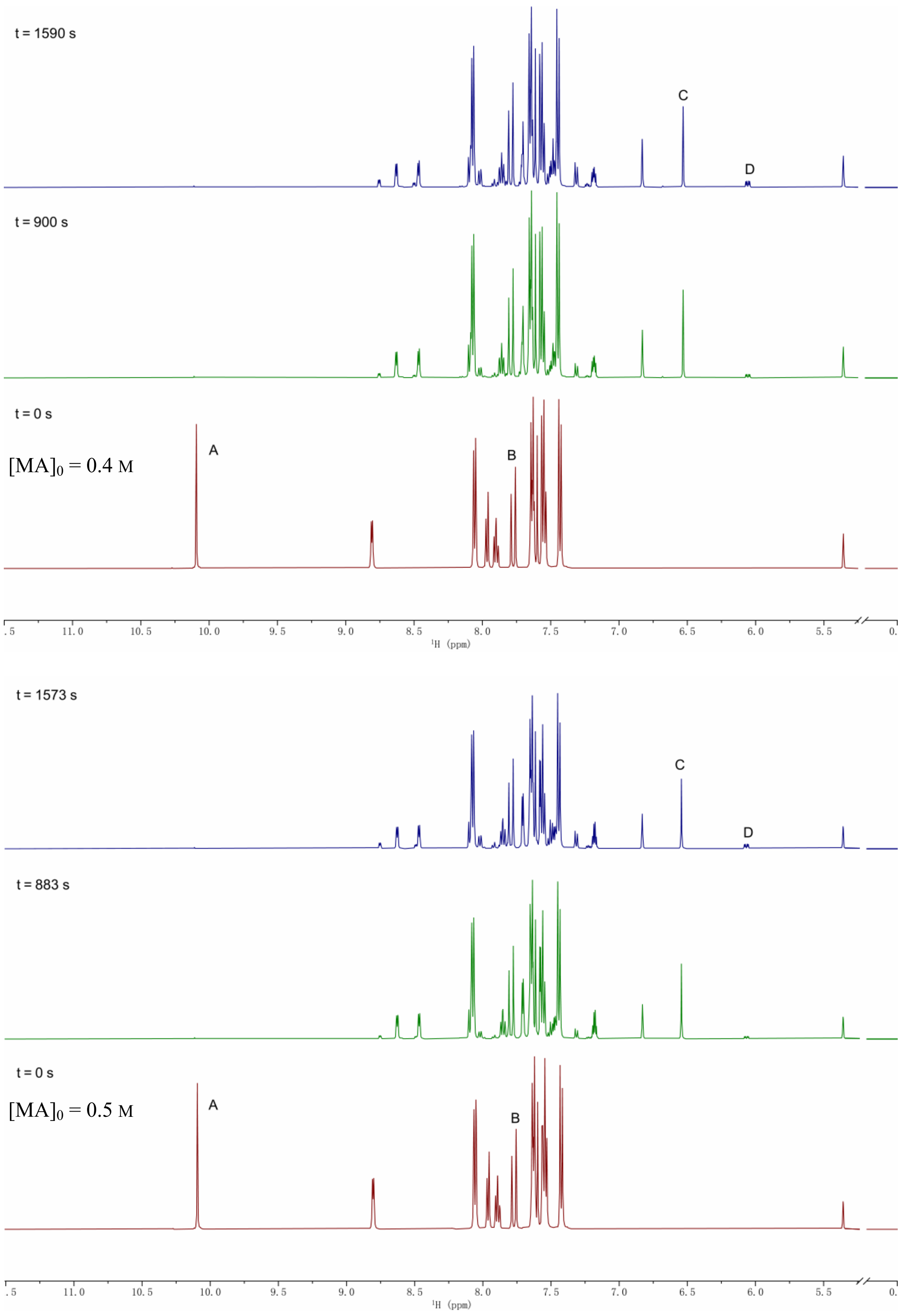

Figure S13. Representative ^{1}H NMR spectra (500 MHz) for reaction of pyridine-2-carboxaldehyde **14** (0.3 M) and chalcone **24** (0.2 M, 0.3 M, 0.4 M and 0.5 M) catalysed by *N*-C_6F_5 NHC precursor **16** (5 mM) in

NEt_3:NEt_3·HCl (2:1, 0.03 M) in CD_2Cl_2 at 25 °C. A = Aldehyde C*H*O, B = Chalcone C*H*, C = Benzoin C*H*, D = Stetter product C*H*.

Table S5. Reaction data and initial rates of Stetter product formation from (E)-3-(4-chlorophenyl)-1-phenylprop-2-en-1-one **24**, catalyzed by C_6F_5 triazolium precatalyst **16** (5 mM) in 0.03 M triethylamine buffer (Et_3N:Et_3N·HCl, 2:1) in CD_2Cl_2 at 25 °C.

[Aldehyde]$_0$ / M	[MA]$_0$ / M	Time / s	[Benzoin] / M	[Stetter] / M	v_{max} / 10^{-5} M s^{-1}	k_s' / 10^{-5} s^{-1}	Average / 10^{-5} s^{-1}
0.3	0.2	0	0	0	1.64	8.20	8.24±0.07
		360	0.144369	0.00504			
		498	0.144404	0.007863			
		636	0.140815	0.010411			
		774	0.137132	0.012623			
		912	0.133556	0.014939			
		1050	0.130236	0.017082			
		1188	0.127217	0.019187			
		1326	0.124315	0.021272			
		1464	0.121179	0.023142			
		1602	0.118022	0.024860			
0.3	0.3	0	0	0	2.50	8.33	
		351	0.140018	0.007236			
		489	0.140434	0.011225			
		627	0.136912	0.015246			
		765	0.134129	0.018545			
		903	0.13084	0.022282			
		1041	0.128287	0.025661			
		1179	0.125445	0.028855			
		1317	0.122456	0.032156			
		1455	0.119932	0.035202			
		1593	0.117319	0.038259			
0.3	0.4	0	0	0	3.26	8.15	
		348	0.134689	0.009455			
		486	0.134557	0.014547			
		624	0.131507	0.019526			
		762	0.128859	0.024198			
		900	0.126078	0.028976			
		1038	0.123421	0.032774			
		1176	0.120862	0.037209			
		1314	0.11860	0.042297			
		1452	0.116167	0.045587			
		1590	0.113895	0.050428			
0.3	0.5	0	0	0	4.14	8.28	

		331	0.129566	0.011059			
		469	0.129189	0.018051			
		607	0.127238	0.023515			
		745	0.124354	0.030338			
		883	0.12252	0.035585			
		1021	0.120292	0.041426			
		1159	0.118186	0.046714			
		1297	0.116032	0.052714			
		1435	0.113967	0.057190			
		1573	0.112090	0.062234			

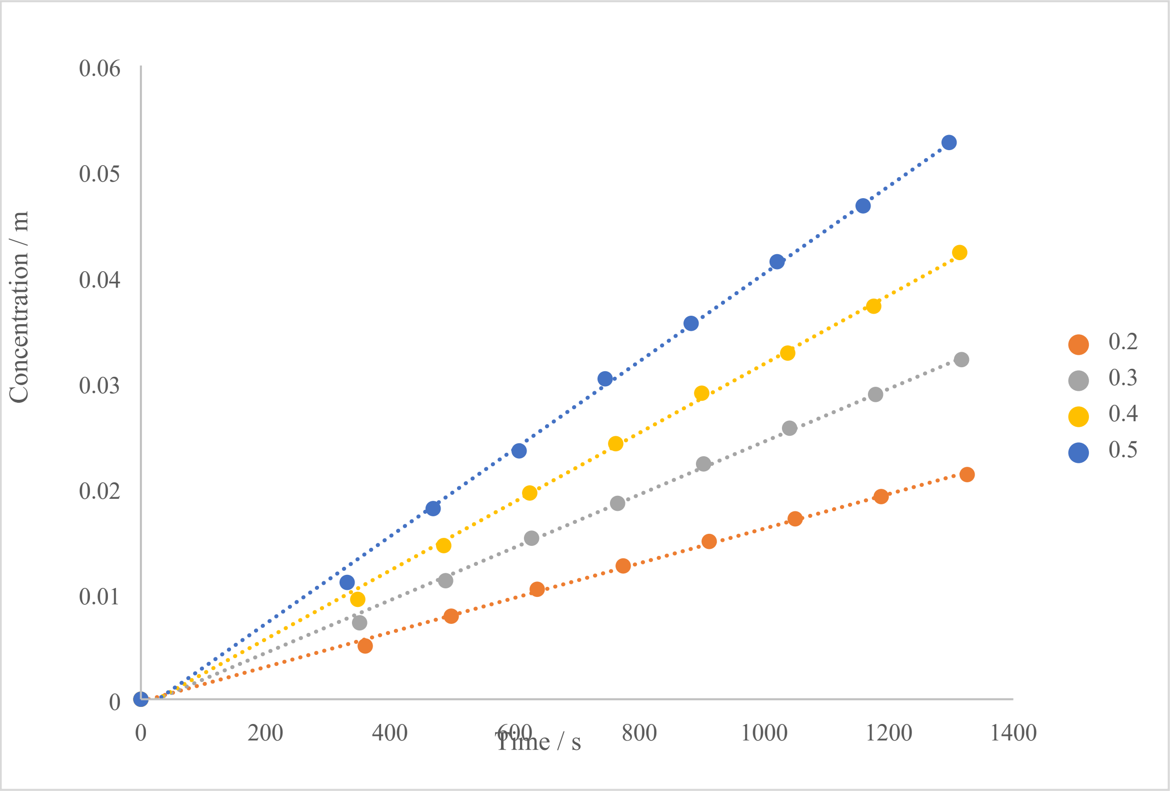

Figure S14. Plots of Stetter product concentration against time for the C_6F_5 triazolium precatalyst **16** (5 mM) catalysed Stetter reaction, at initial (E)-3-(4-chlorophenyl)-1-phenylprop-2-en-1-one **24** concentrations 0.2 M, 0.3 M, 0.4 M, 0.5 M.

Entry 6

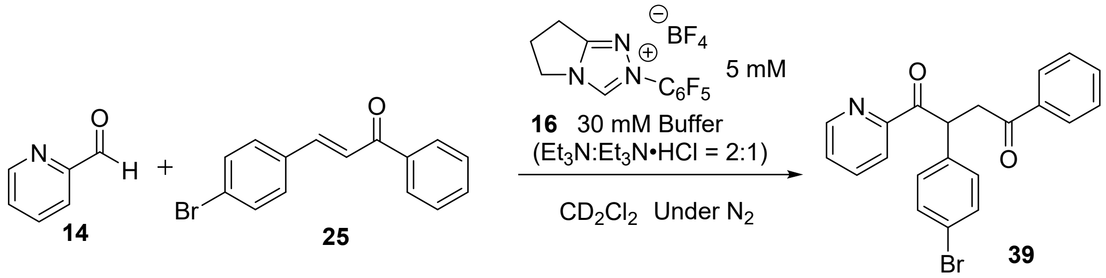

The reaction of pyridine-2-carboxaldehyde **14** and chalcone **25** catalysed by C_6F_5 triazolium salt **16** in

triethylamine buffer was monitored using ^{1}H NMR spectra, with representative NMR spectra over the course of the experiment given in Figure S15.

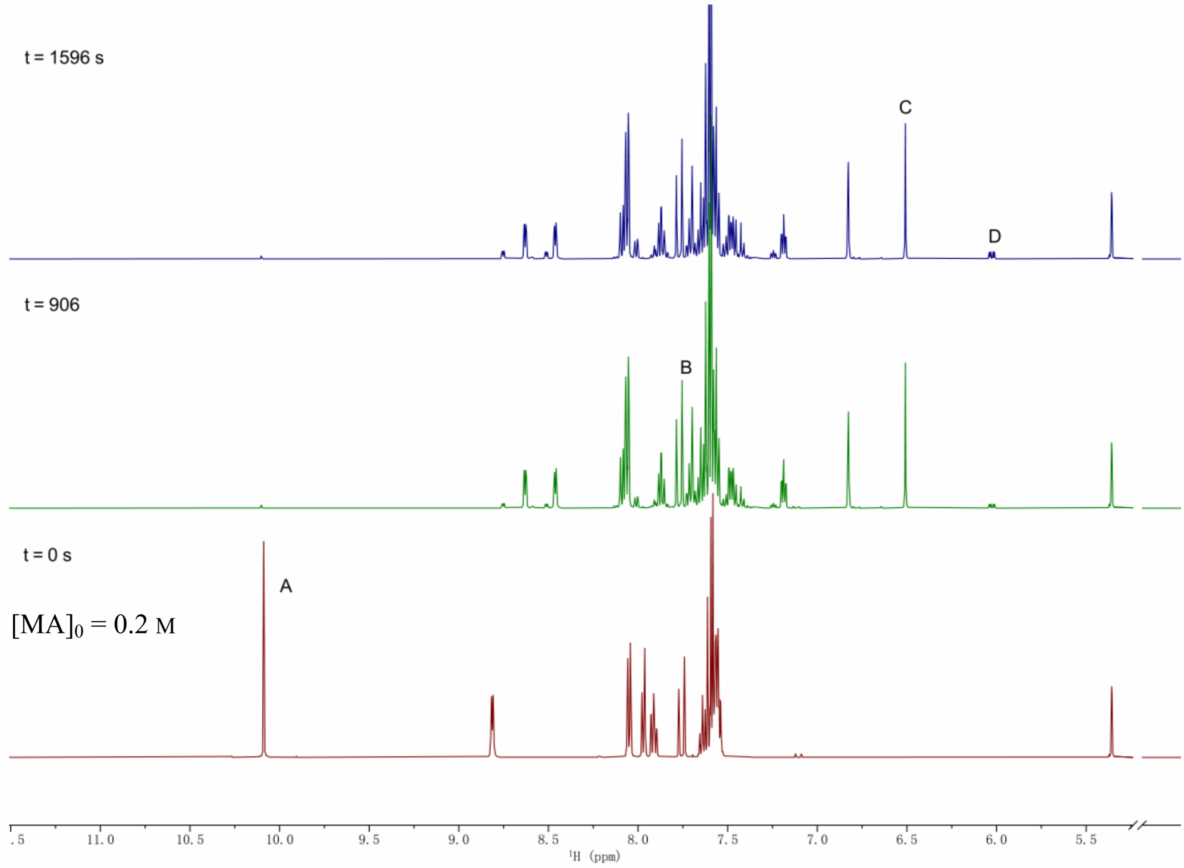

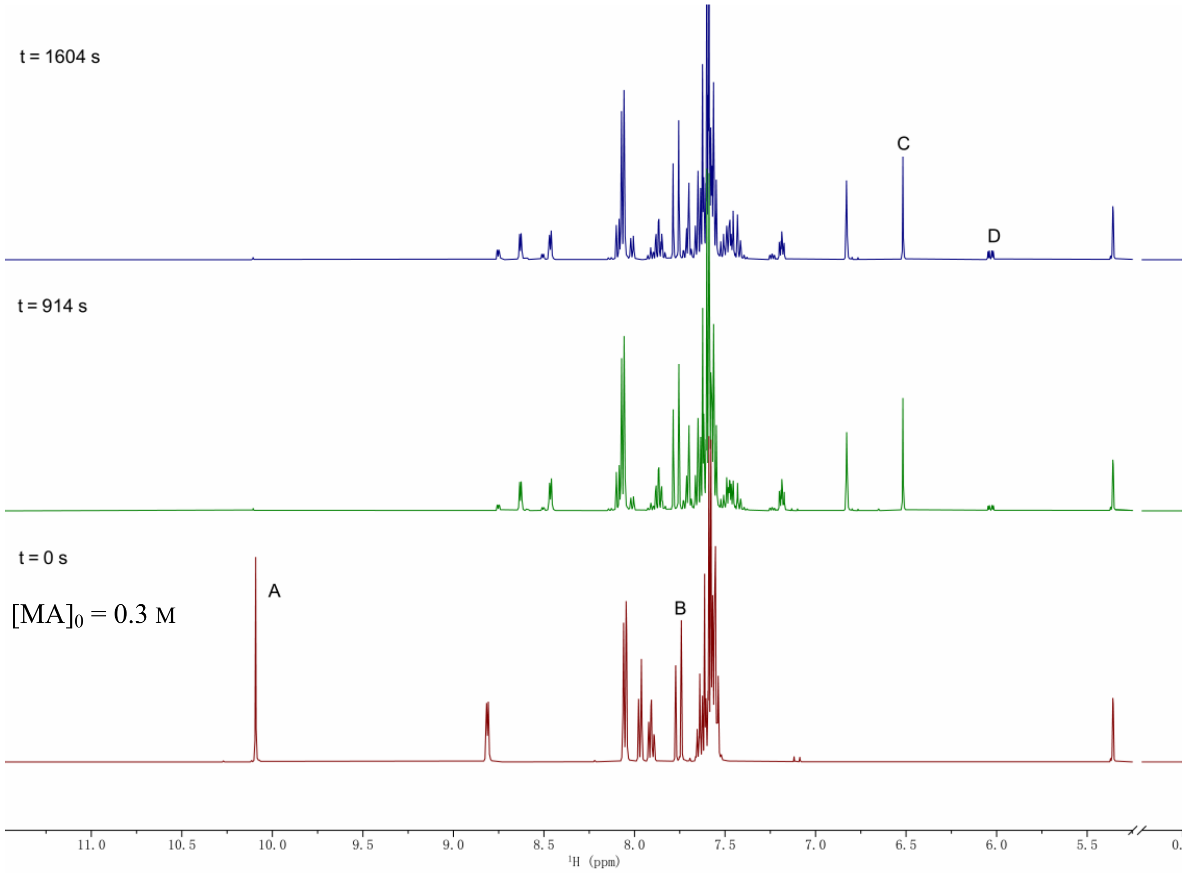

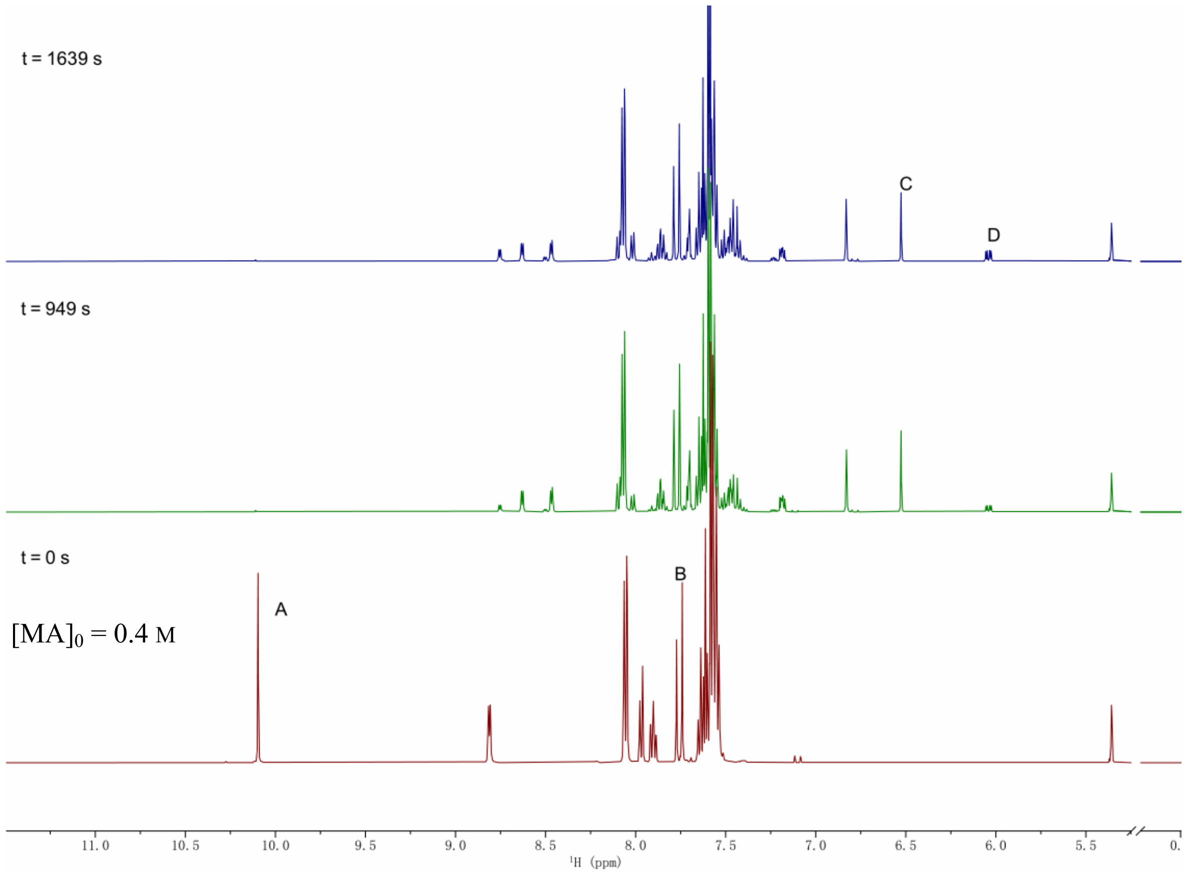

Figure S15. Representative ^{1}H NMR spectra (500 MHz) for reaction of pyridine-2-carboxaldehyde **14** (0.3 M) and chalcone **25** (0.2 M, 0.3 M, and 0.4 M) catalysed by *N*-C_6F_5 NHC precursor **16** (5 mM) in NEt_3:NEt_3·HCl (2:1, 0.03 M) in CD_2Cl_2 at 25 °C. A = Aldehyde C*H*O, B = Chalcone C*H*, C = Benzoin C*H*, D = Stetter product C*H*.

Table S6. Reaction data and initial rates of Stetter product formation from (E)-3-(4-bromophenyl)-1-phenylprop-2-en-1-one **25**, catalyzed by C_6F_5 triazolium precatalyst **16** (5 mM) in 0.03 M triethylamine buffer (Et_3N:Et_3N·HCl, 2:1) in CD_2Cl_2 at 25 °C.

**[Aldehyde]$_0$ / M**	**[MA]$_0$ / M**	**Time / s**	**[Benzoin] / M**	**[Stetter] / M**	v_{max} **/ 10^{-5} M s^{-1}**	k'_s **/ 10^{-5} s^{-1}**	**Average / 10^{-5} s^{-1}**
0.3	0.2	0	0	0	1.69	8.45	8.49±0.03
		354	0.139189	0.004784			
		492	0.138982	0.007623			
		630	0.136161	0.01028			
		768	0.133378	0.012794			
		906	0.130596	0.015130			
		1044	0.128055	0.017303			
		1182	0.125641	0.019610			
		1320	0.123311	0.021708			
		1458	0.121035	0.023832			
		1596	0.118776	0.025732			
0.3	0.3	0	0	0	2.55	8.50	

		362	0.133032	0.007492			
		500	0.131904	0.012002			
		638	0.128205	0.015576			
		776	0.124750	0.019378			
		914	0.121655	0.023154			
		1052	0.11858	0.026748			
		1190	0.115583	0.029759			
		1328	0.113054	0.032877			
		1466	0.110200	0.035749			
		1604	0.107603	0.038535			
0.3	0.4	0	0	0	3.41	8.53	
		397	0.127753	0.011937			
		535	0.125548	0.017710			
		673	0.120965	0.022944			
		811	0.116531	0.027928			
		949	0.112393	0.03239			
		1087	0.108358	0.036622			
		1225	0.10501	0.041371			
		1363	0.101583	0.045736			
		1501	0.098096	0.049797			
		1639	0.094651	0.053784			

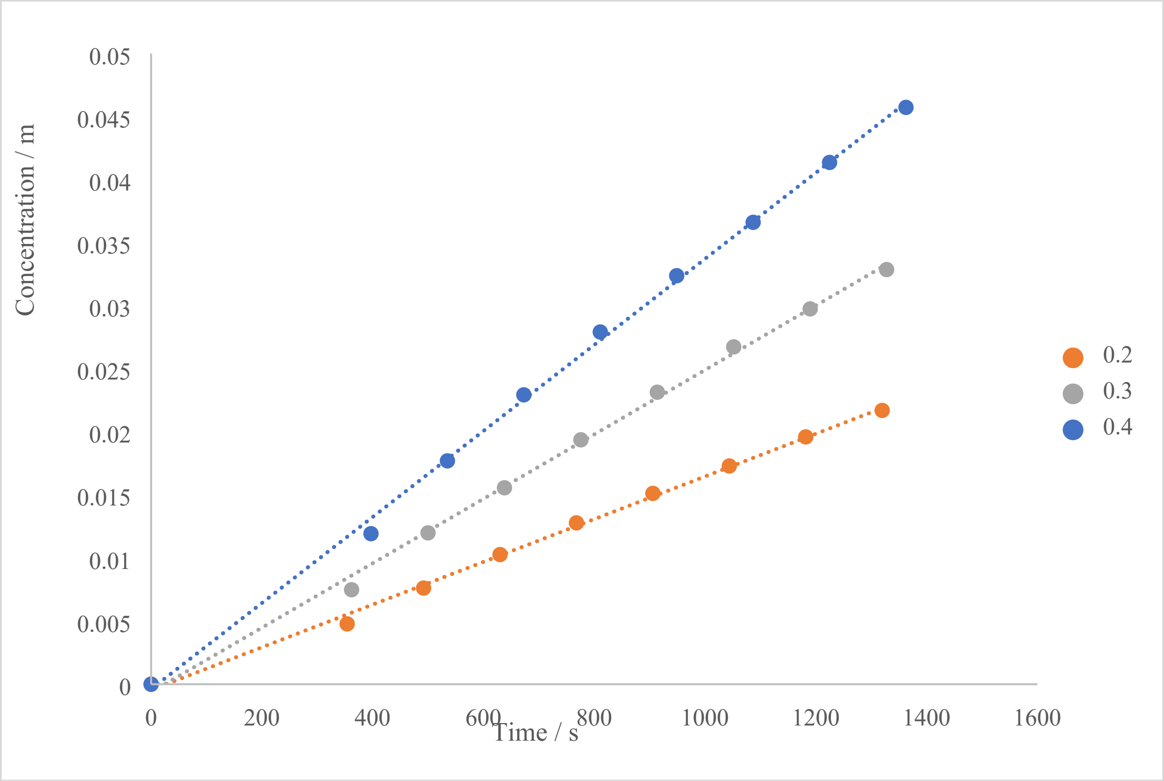

Figure S16. Plots of Stetter product concentration against time for the C_6F_5 triazolium precatalyst **16** (5 mM) catalysed Stetter reaction, at initial (E)-3-(4-bromophenyl)-1-phenylprop-2-en-1-one **25** concentrations 0.2

M, 0.3 M, 0.4 M.

Entry 7

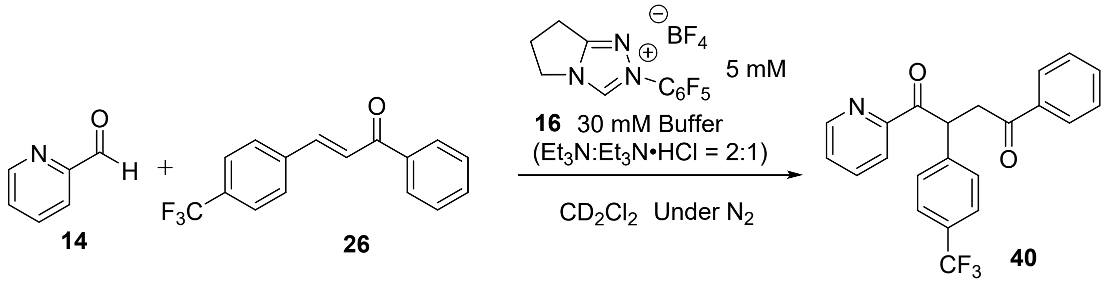

The reaction of pyridine-2-carboxaldehyde **14** and chalcone **26** catalysed by C_6F_5 triazolium salt **16** in triethylamine buffer was monitored using ^{1}H NMR spectra, with representative NMR spectra over the course of the experiment given in Figure S17.

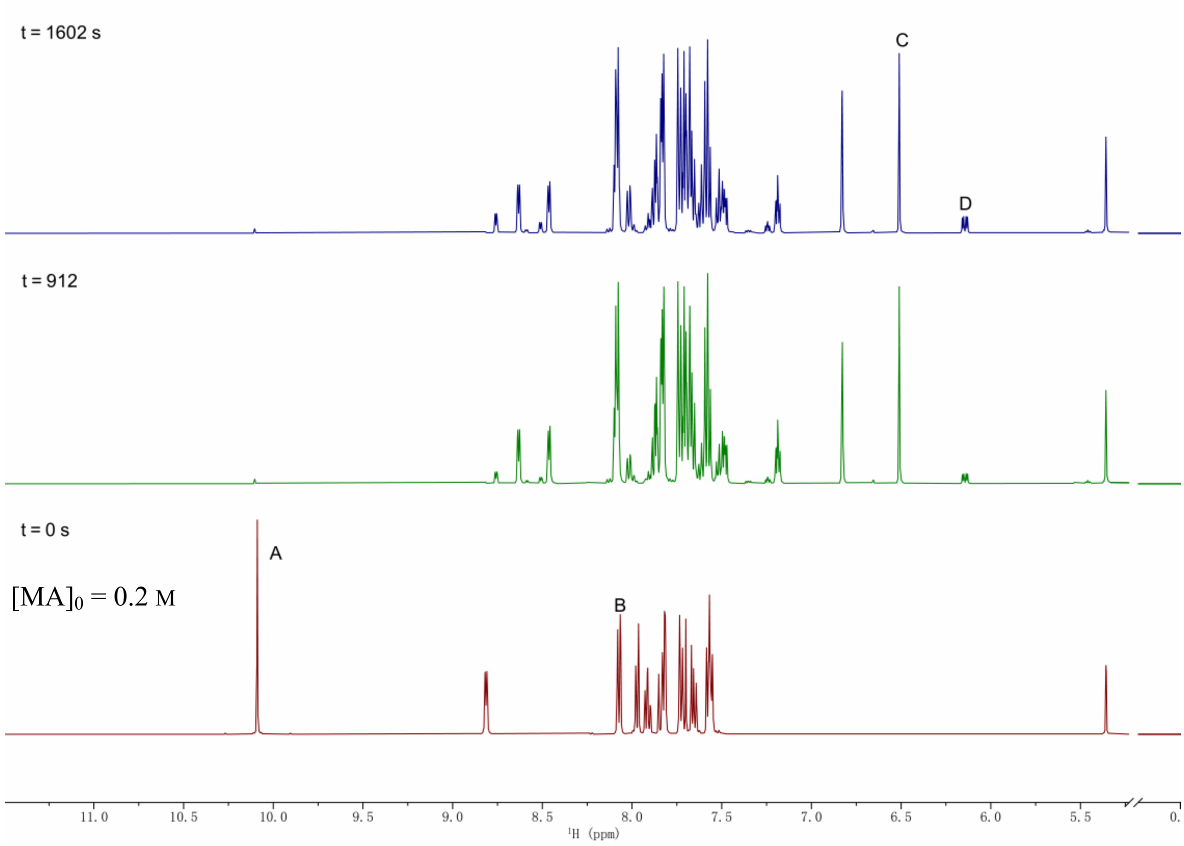

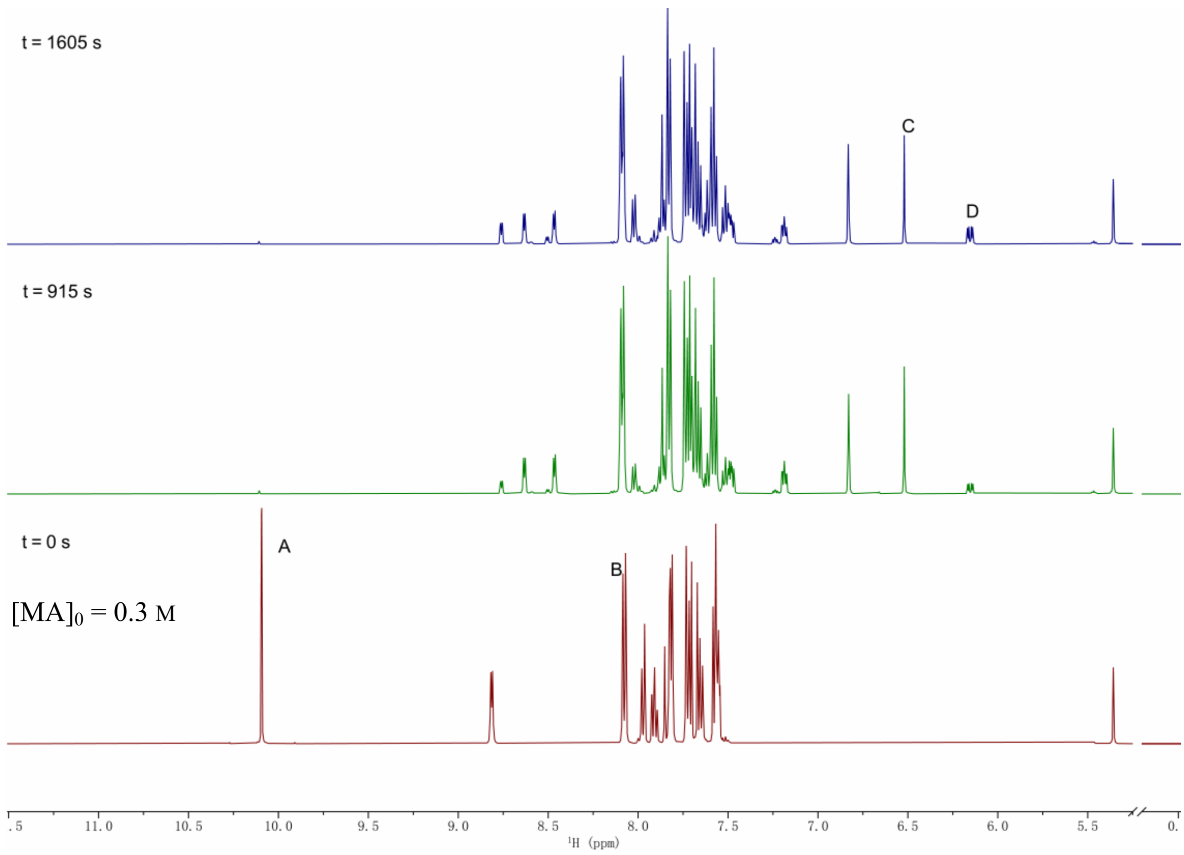
t = 1605 s
C
D
t = 915 s
t = 0 s
A
B
[MA]$_0$ = 0.3 M
11.0
10.5
10.0
9.5
9.0
8.5
8.0
7.5
7.0
6.5
6.0
5.5
^{1}H (ppm)

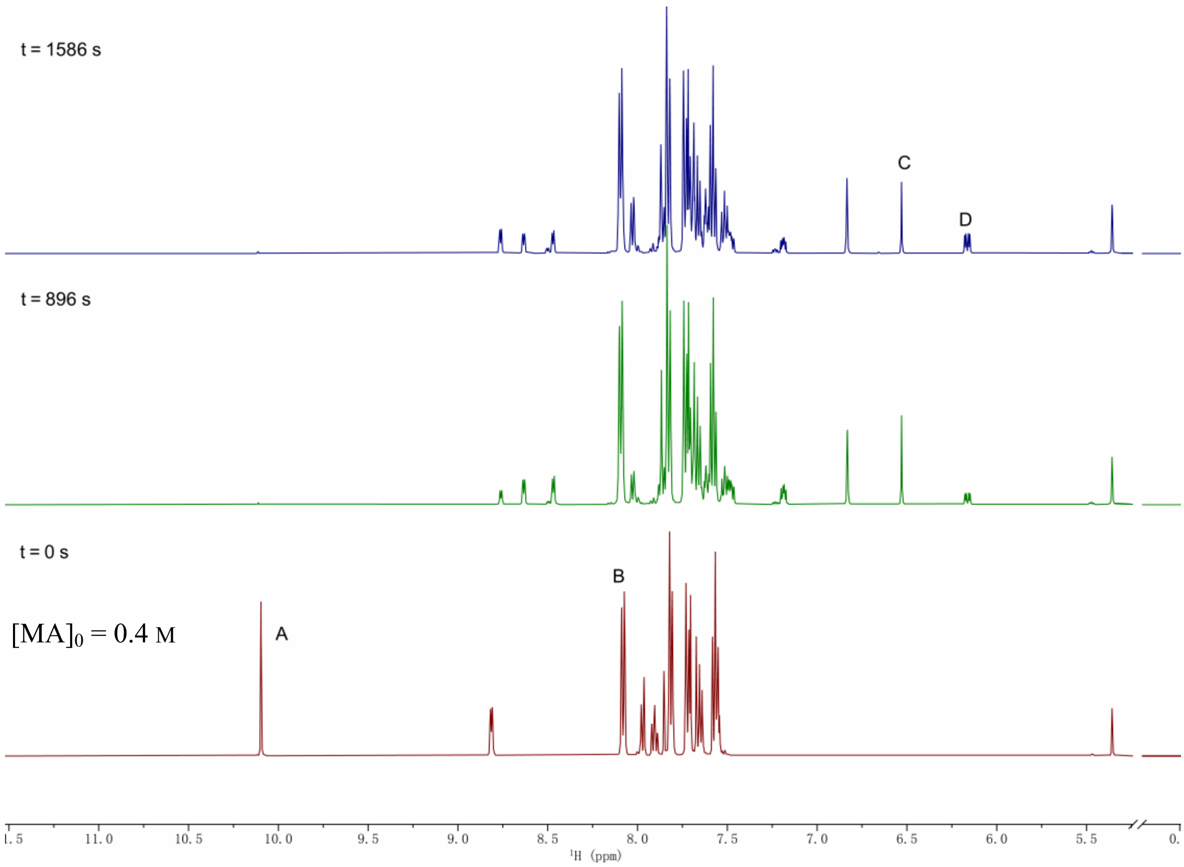
t = 1586 s
C
D
t = 896 s
t = 0 s
B
[MA]$_0$ = 0.4 M
A
11.0
10.5
10.0
9.5
9.0
8.5
8.0
7.5
7.0
6.5
6.0
5.5
^{1}H (ppm)

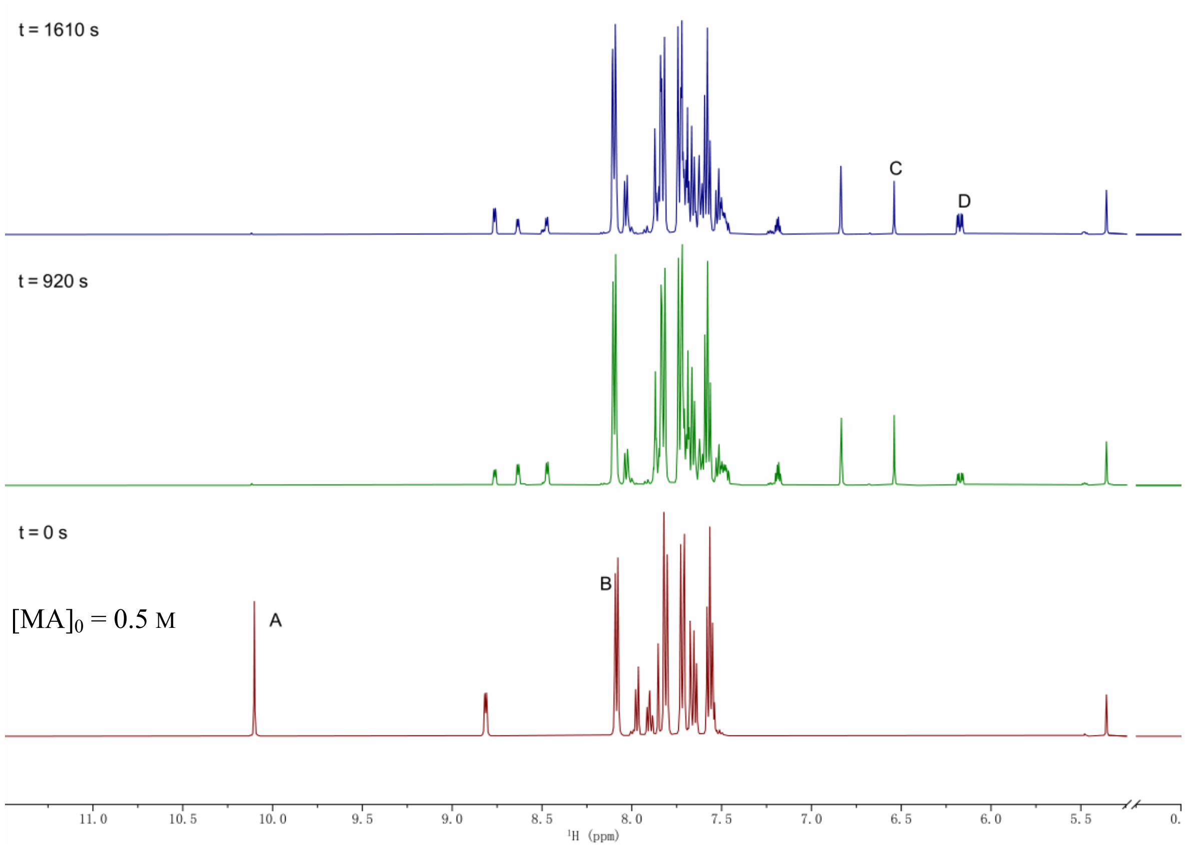

Figure S17. Representative ^{1}H NMR spectra (500 MHz) for reaction of pyridine-2-carboxaldehyde **14** (0.3 M) and chalcone **26** (0.2 M, 0.3 M, 0.4 M and 0.5 M) catalysed by *N*-C_6F_5 NHC precursor **16** (5 mM) in NEt_3:NEt_3·HCl (2:1, 0.03 M) in CD_2Cl_2 at 25 °C. A = Aldehyde C*H*O, B = Chalcone Ph*H*, C = Benzoin C*H*, D = Stetter product C*H*.

Table S7. Reaction data and initial rates of Stetter product formation from (E)-1-phenyl-3-(4-(trifluoromethyl)phenyl)prop-2-en-1-one **26**, catalyzed by C_6F_5 triazolium precatalyst **16** (5 mM) in 0.03 M triethylamine buffer (Et_3N:Et_3N·HCl, 2:1) in CD_2Cl_2 at 25 °C.

**[Aldehyde]$_0$ / M**	**[MA]$_0$ / M**	**Time / s**	**[Benzoin] / M**	**[Stetter] / M**	v_{max} **/ 10^{-5} M s^{-1}**	k_s' **/ 10^{-5} s^{-1}**	**Average / 10^{-5} s^{-1}**
0.3	0.2	0	0	0	2.96	14.80	15.00±0.11
		360	0.13991	0.008864			
		498	0.139051	0.014156			
		636	0.13554	0.018521			
		774	0.131997	0.022708			
		912	0.128752	0.026973			
		1050	0.125626	0.030816			
		1188	0.122581	0.03477			
		1326	0.119833	0.03817			
		1464	0.116967	0.041543			
		1602	0.114328	0.044816			
0.3	0.3	0	0	0	4.50	15.00	

		363	0.135699	0.013604			
		501	0.133263	0.021336			
		639	0.128565	0.028299			
		777	0.124027	0.034992			
		915	0.119859	0.041288			
		1053	0.1157	0.046876			
		1191	0.111937	0.052634			
		1329	0.108419	0.058236			
		1467	0.104775	0.063533			
		1605	0.101507	0.068872			
0.3	0.4	0	0	0	6.02	15.10	
		344	0.132575	0.018280			
		482	0.128669	0.028481			
		620	0.122457	0.037421			
		758	0.116606	0.045983			
		896	0.110950	0.054330			
		1034	0.105807	0.062490			
		1172	0.100586	0.069943			
		1310	0.095676	0.077202			
		1448	0.091021	0.084170			
		1586	0.086598	0.091163			
0.3	0.5	0	0	0	7.48	15.0	
		368	0.125421	0.025971			
		506	0.120477	0.038048			
		644	0.113904	0.049362			
		782	0.107355	0.059814			
		920	0.101101	0.070076			
		1058	0.095313	0.079892			
		1196	0.089716	0.089370			
		1334	0.084194	0.098332			
		1472	0.078995	0.107160			
		1610	0.074099	0.115850			

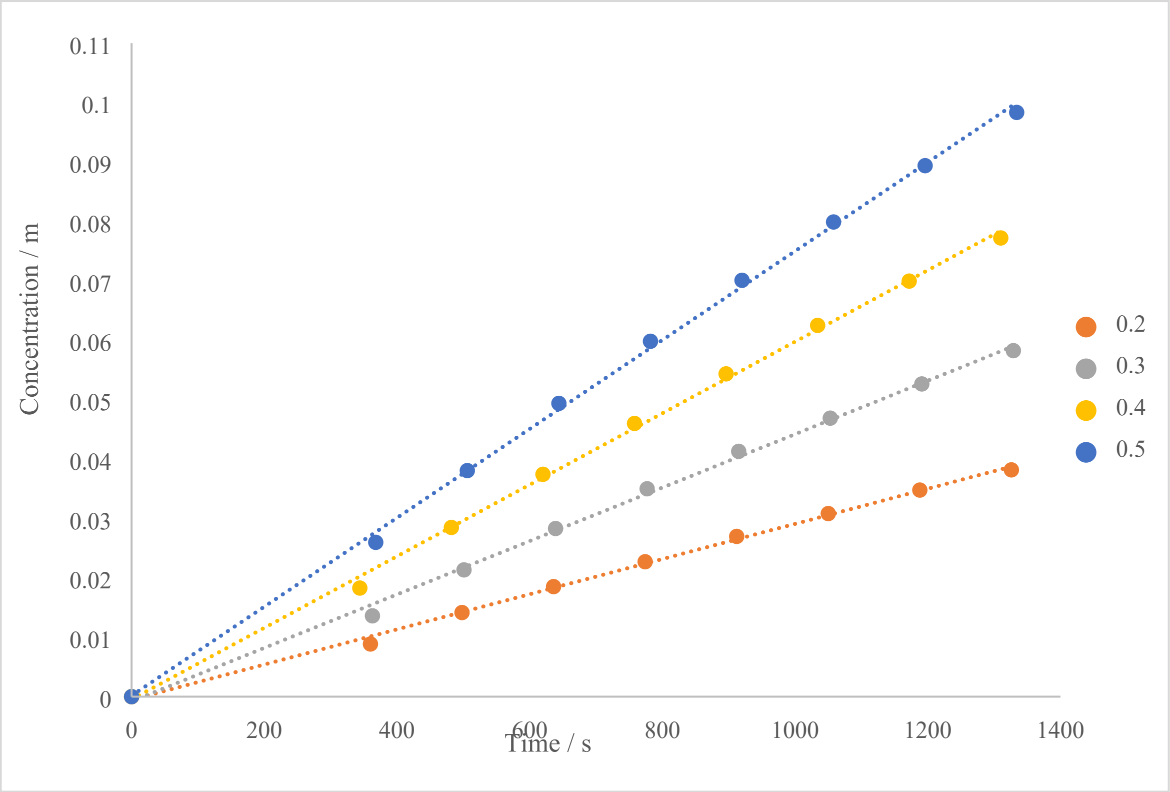

Figure S18. Plots of Stetter product concentration against time for the C_6F_5 triazolium precatalyst **16** (5 mM) catalysed Stetter reaction, at initial (E)-1-phenyl-3-(4-(trifluoromethyl)phenyl)prop-2-en-1-one **26** concentrations 0.2 M, 0.3 M, 0.4 M, 0.5 M.

Entry 8

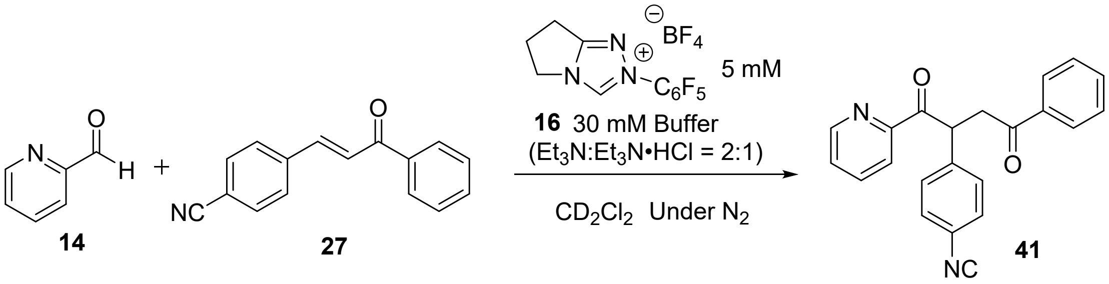

The reaction of pyridine-2-carboxaldehyde **14** and chalcone **27** catalysed by C_6F_5 triazolium salt **16** in triethylamine buffer was monitored using ^{1}H NMR spectra, with representative NMR spectra over the course of the experiment given in Figure S19.

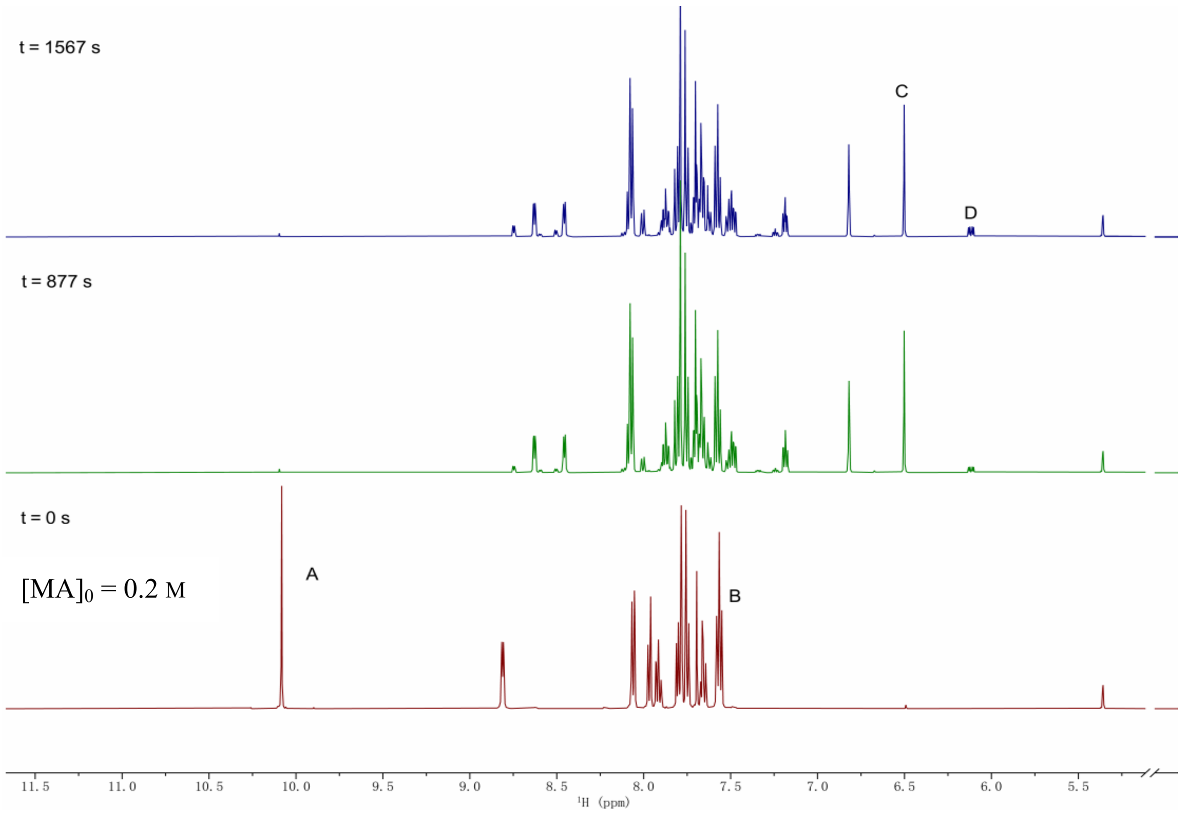
t = 1567 s
C
D
t = 877 s
t = 0 s
[MA]$_0$ = 0.2 M
A
B
11.5 11.0 10.5 10.0 9.5 9.0 8.5 8.0 7.5 7.0 6.5 6.0 5.5
^{1}H (ppm)

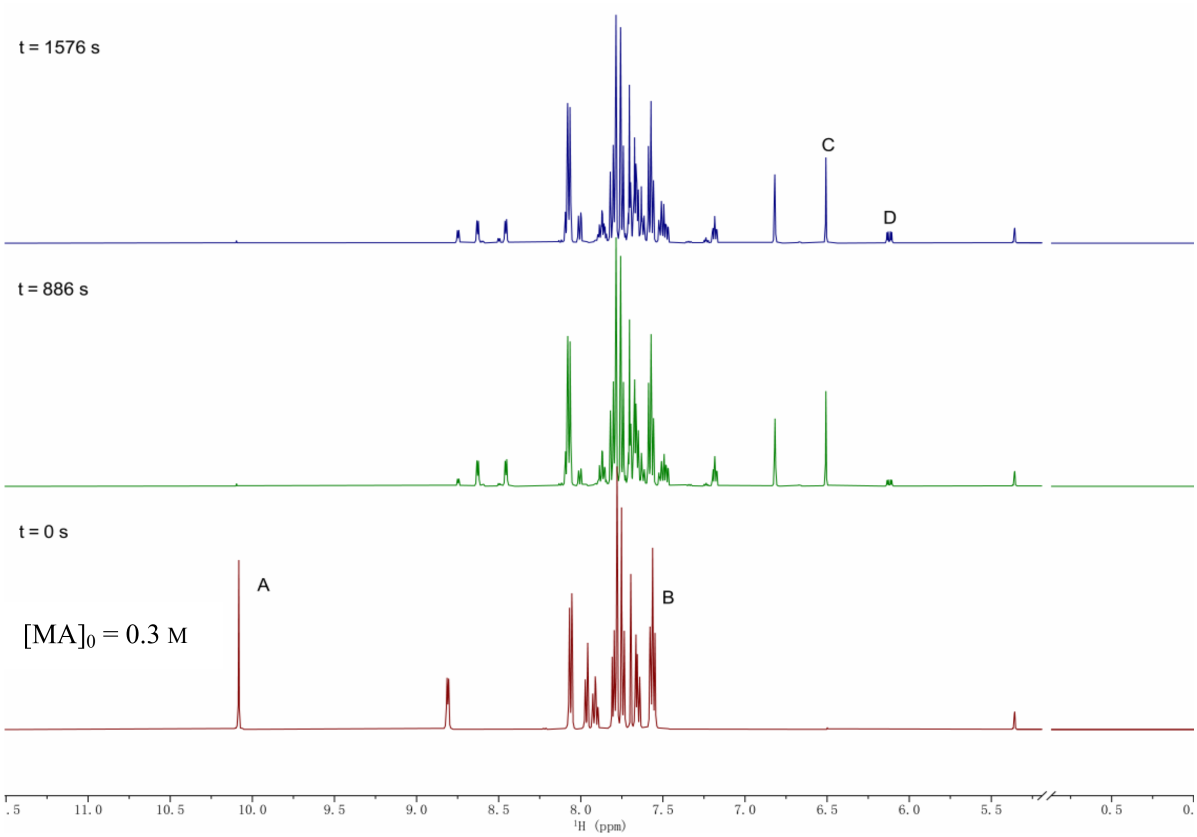
t = 1576 s
C
D
t = 886 s
t = 0 s
A
[MA]$_0$ = 0.3 M
B
.5 11.0 10.5 10.0 9.5 9.0 8.5 8.0 7.5 7.0 6.5 6.0 5.5 0.5 0.
^{1}H (ppm)

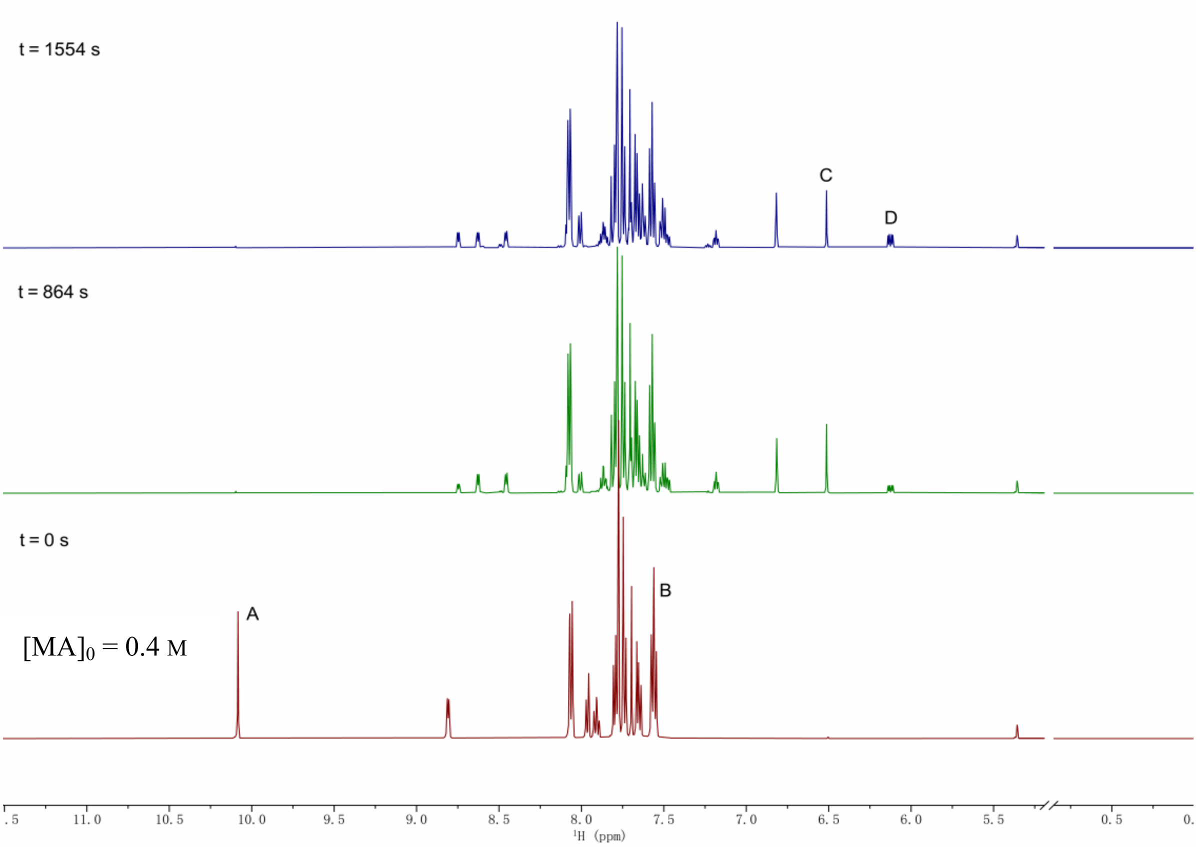

Figure S19. Representative ^{1}H NMR spectra (500 MHz) for reaction of pyridine-2-carboxaldehyde **14** (0.3 M) and chalcone **27** (0.2 M, 0.3 M and 0.4 M) catalysed by *N*-C_6F_5 NHC precursor **16** (5 mM) in NEt_3:NEt_3·HCl (2:1, 0.03 M) in CD_2Cl_2 at 25 °C. A = Aldehyde C*H*O, B = Chalcone Ph*H*, C = Benzoin C*H*, D = Stetter product C*H*.

Table S8. Reaction data and initial rates of Stetter product formation from chalcone **27** and pyridine-2-carboxaldehyde **14**, catalysed by C_6F_5 triazolium precatalyst **16** (5 mM) in 0.03 M triethylamine buffer (Et_3N:Et_3N·HCl, 2:1) in CD_2Cl_2 at 25 °C.

**[Aldehyde]$_0$ / M**	**[MA]$_0$ / M**	**Time / s**	**[Benzoin] / M**	**[Stetter] / M**	**v_{max} / 10^{-5} M s^{-1}**	**k_s' / 10^{-5} s^{-1}**	**Average / 10^{-5} s^{-1}**
0.3	0.2	0	0	0	3.21	16.05	15.79±0.40
		325	0.143816	0.010051			
		463	0.140805	0.015234			
		601	0.135913	0.020435			
		739	0.132298	0.024868			
		877	0.128589	0.029167			
		1015	0.125381	0.033203			
		1153	0.122357	0.036611			
		1291	0.119563	0.041169			
		1429	0.117292	0.044814			
		1567	0.11533	0.04857			
0.3	0.3	0	0	0	4.83	16.10	

		334	0.141881	0.014248			
		472	0.137208	0.021761			
		610	0.131881	0.029182			
		748	0.127325	0.036383			
		886	0.123209	0.042434			
		1024	0.119537	0.049292			
		1162	0.116388	0.055602			
		1300	0.113629	0.061633			
		1438	0.111136	0.06848			
		1576	0.10906	0.074408			
0.3	0.4	0	0	0	6.09	15.23	
		312	0.133595	0.020478			
		450	0.130385	0.029996			
		588	0.125329	0.038809			
		726	0.120426	0.047203			
		864	0.115918	0.055198			
		1002	0.111425	0.063098			
		1140	0.106819	0.070558			
		1278	0.102523	0.077826			
		1416	0.098172	0.084863			
		1554	0.094191	0.091699			

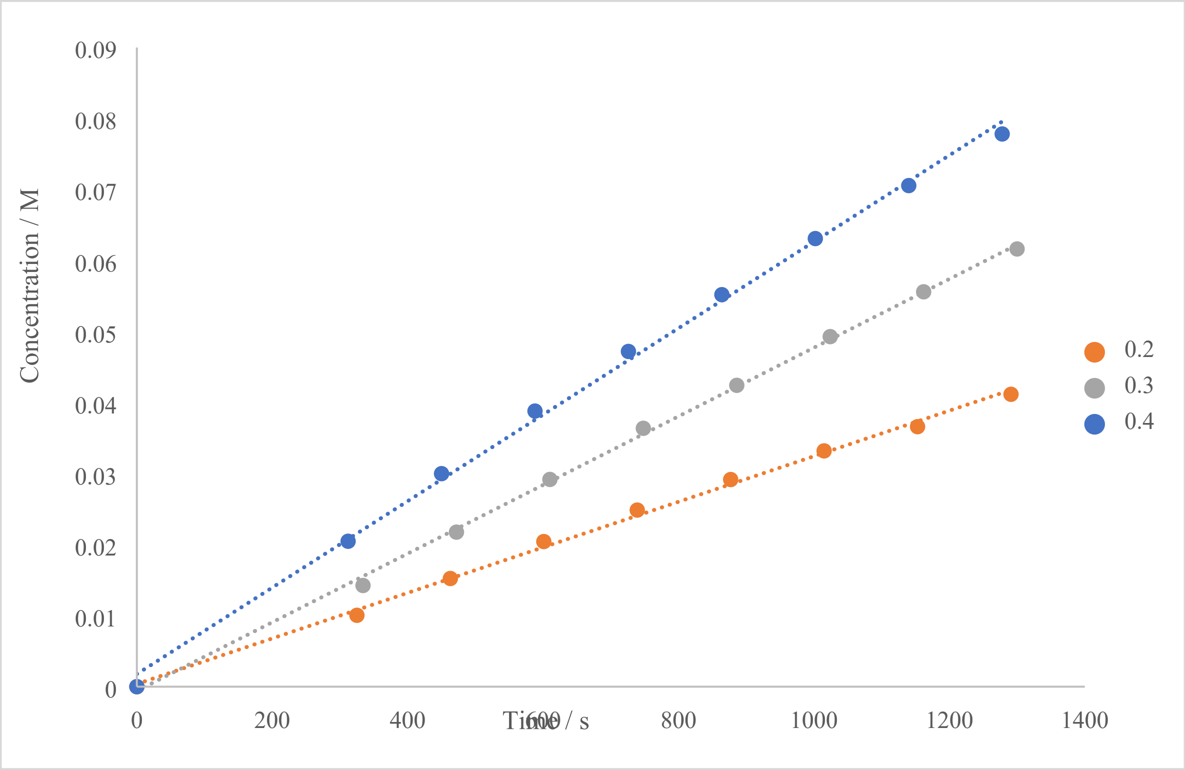

Figure S20. Plots of Stetter product concentration against time for the C_6F_5 triazolium precatalyst **16** (5 mM) catalysed Stetter reaction, at initial chalcone **27** concentrations 0.2 M, 0.3 M, 0.4 M.

Entry 9

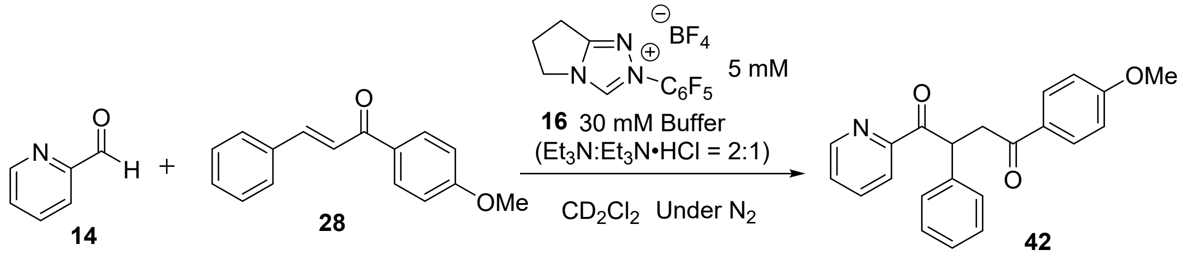

The reaction of pyridine-2-carboxaldehyde **14** and chalcone **28** catalysed by C_6F_5 triazolium salt **16** in triethylamine buffer was monitored using ^{1}H NMR spectra, with representative NMR spectra over the course of the experiment given in Figure S21.

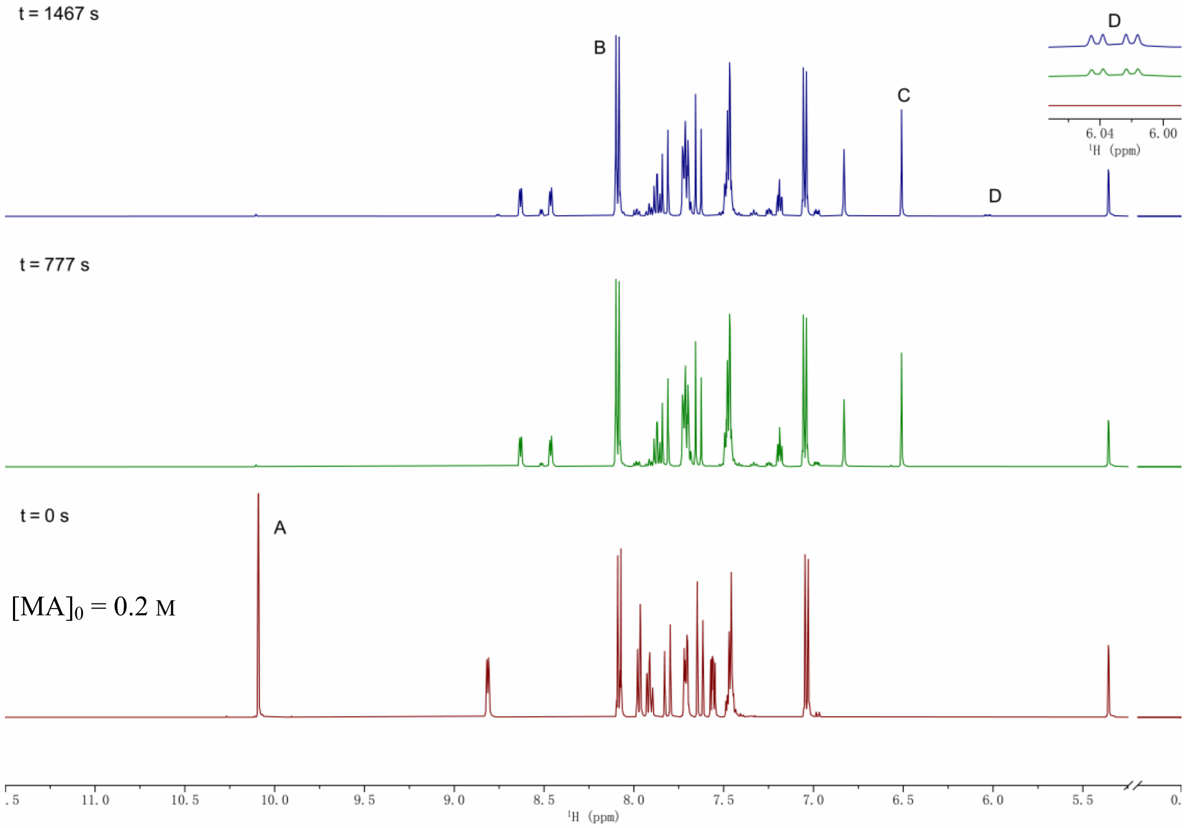

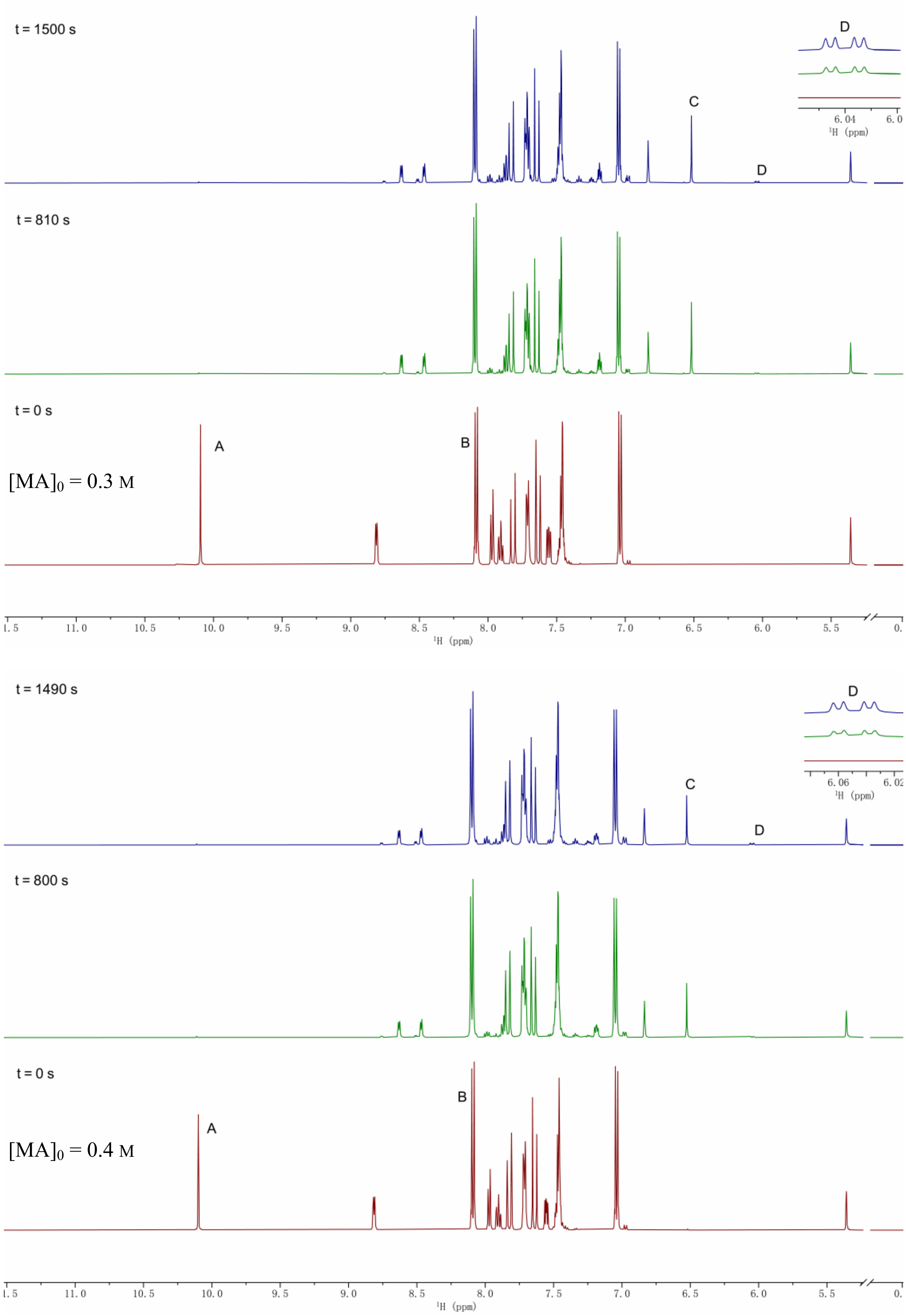

Figure S21. Representative ^{1}H NMR spectra (500 MHz) for reaction of pyridine-2-carboxaldehyde **14** (0.3 M) and chalcone **28** (0.2 M, 0.3 M and 0.4 M) catalysed by *N*-C_6F_5 NHC precursor **16** (5 mM) in NEt_3:NEt_3·HCl

(2:1, 0.03 M) in CD_2Cl_2 at 25 °C. A = Aldehyde C*H*O, B = Chalcone Ph*H*, C = Benzoin C*H*, D = Stetter product C*H*.

Table S9. Reaction data and initial rates of Stetter product formation from (E)-1-(4-methoxylphenyl)-3-phenylprop-2-en-1-one **28**, catalyzed by C_6F_5 triazolium precatalyst **16** (5 mM) in 0.03 M triethylamine buffer (Et_3N:Et_3N·HCl, 2:1) in CD_2Cl_2 at 25 °C.

**[Aldehyde]₀ / M**	**[MA]₀ / M**	**Time / s**	**[Benzoin] / M**	**[Stetter] / M**	v_{max} **/ 10⁻⁶ M s⁻¹**	k_s' **/ 10⁻⁵ s⁻¹**	**Average / 10⁻⁵ s⁻¹**
0.3	0.2	0	0	0	6.05	3.04	3.03±0.06
		225	0.146844	0.001121			
		363	0.14878	0.00175			
		501	0.146917	0.002866			
		639	0.1446	0.003768			
		777	0.142277	0.00442			
		915	0.140601	0.005291			
		1053	0.138628	0.006338			
		1191	0.136667	0.007009			
		1329	0.135077	0.007755			
		1467	0.133031	0.008396			
0.3	0.3	0	0	0	9.29	3.10	
		258	0.153217	0.001755			
		396	0.153706	0.00301			
		534	0.151054	0.004189			
		672	0.148474	0.005751			
		810	0.146051	0.006889			
		948	0.14407	0.008425			
		1086	0.141941	0.00966			
		1224	0.140287	0.011128			
		1362	0.137786	0.012384			
		1500	0.135781	0.013518			
0.3	0.4	0	0	0	11.80	2.95	
		248	0.145305	0.001836			
		386	0.145279	0.003813			
		524	0.142961	0.005778			
		662	0.140876	0.007423			
		800	0.13806	0.009108			
		938	0.136157	0.010492			
		1076	0.133544	0.012361			
		1214	0.131149	0.013704			
		1352	0.129487	0.015374			
		1490	0.127124	0.017077			

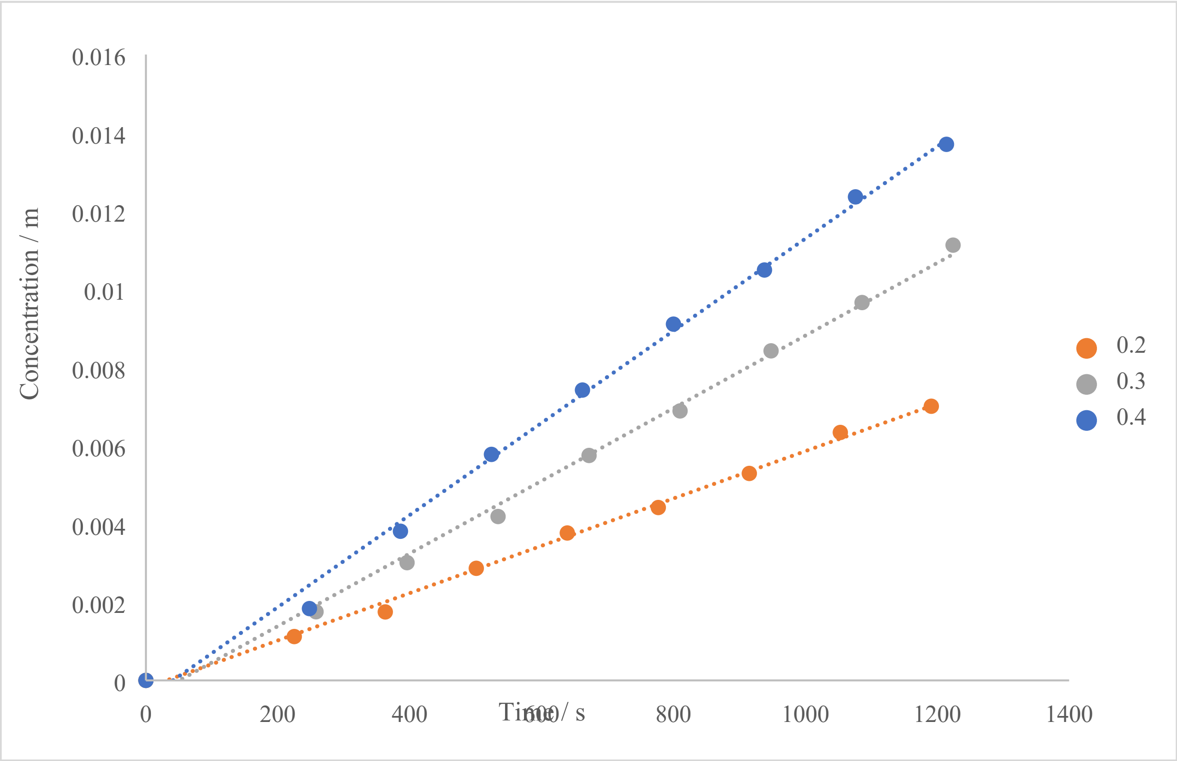

Figure S22. Plots of Stetter product concentration against time for the C_6F_5 triazolium precatalyst **16** (5 mM) catalysed Stetter reaction, at initial (E)-1-(4-methoxylphenyl)-3-phenylprop-2-en-1-one **28** concentrations 0.2 M, 0.3 M, 0.4 M.

Entry 10

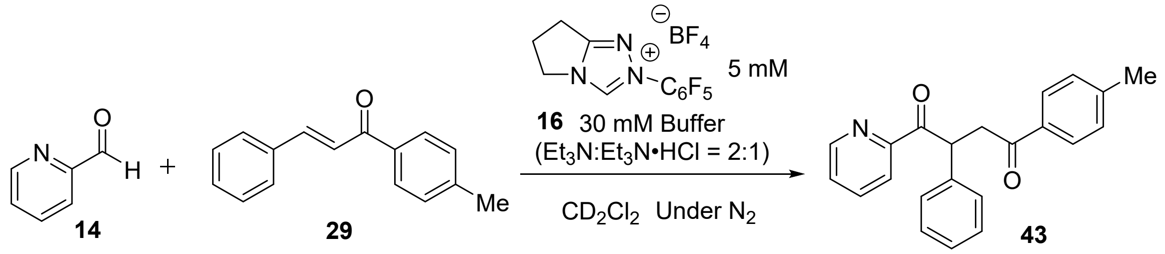

The reaction of pyridine-2-carboxaldehyde **14** and chalcone **29** catalysed by C_6F_5 triazolium salt **16** in triethylamine buffer was monitored using ^{1}H NMR spectra, with representative NMR spectra over the course of the experiment given in Figure S23.

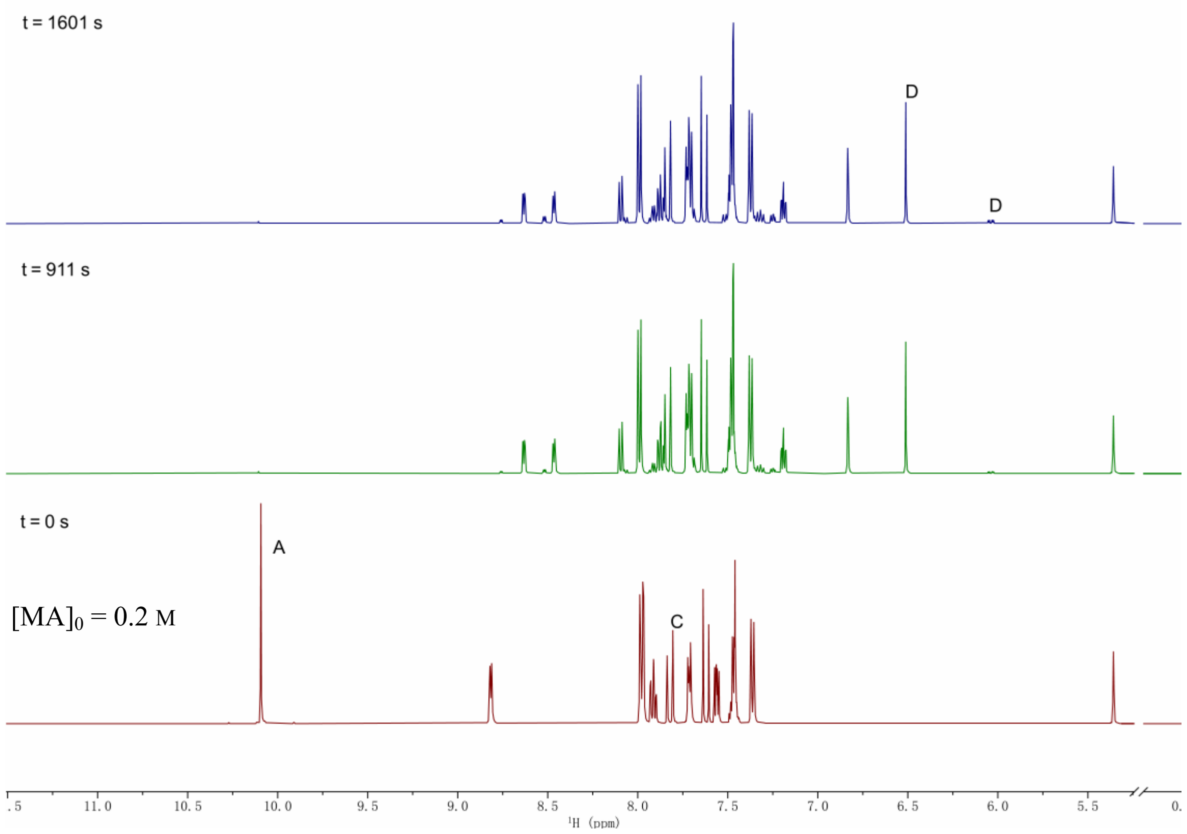
t = 1601 s
D
D
t = 911 s
t = 0 s
A
[MA]$_0$ = 0.2 M
C
.5
11.0
10.5
10.0
9.5
9.0
8.5
8.0
7.5
7.0
6.5
6.0
5.5
0.
^{1}H (ppm)

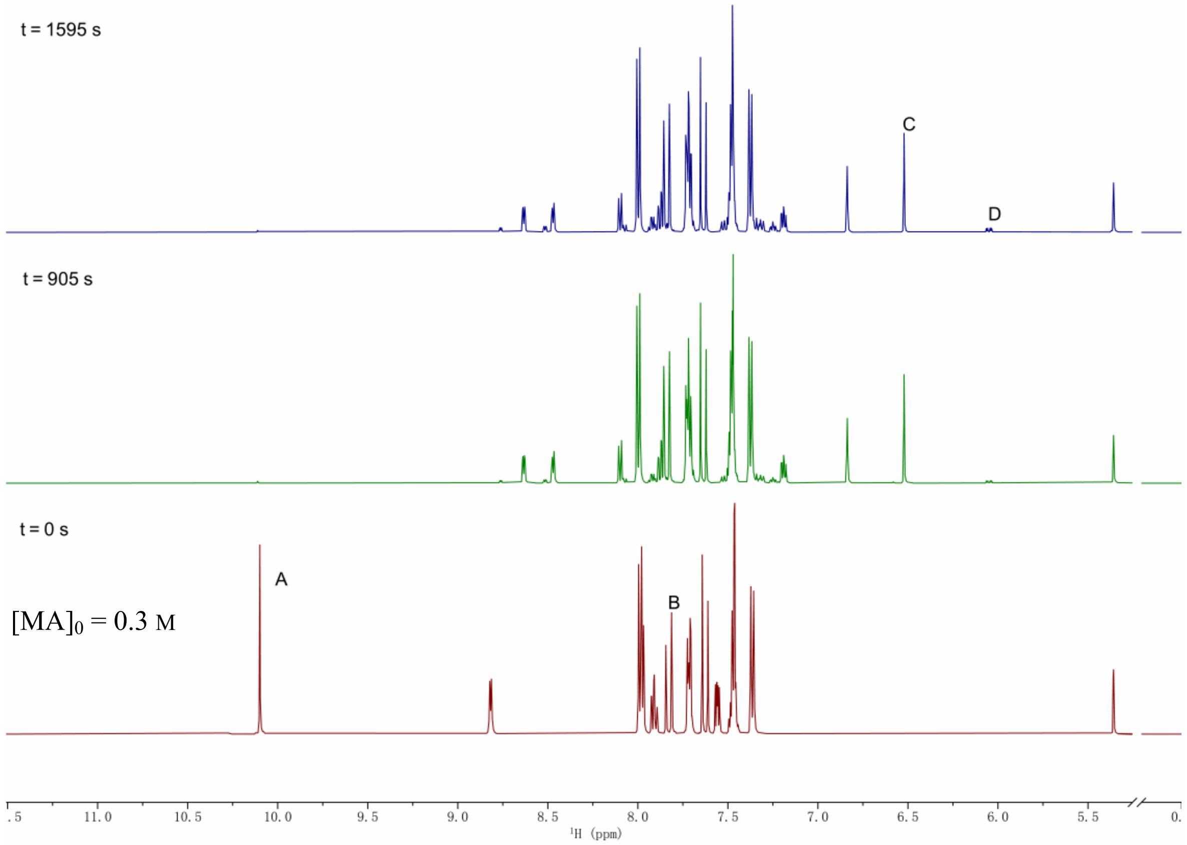
t = 1595 s
C
D
t = 905 s
t = 0 s
A
[MA]$_0$ = 0.3 M
B
.5
11.0
10.5
10.0
9.5
9.0
8.5
8.0
7.5
7.0
6.5
6.0
5.5
0.
^{1}H (ppm)

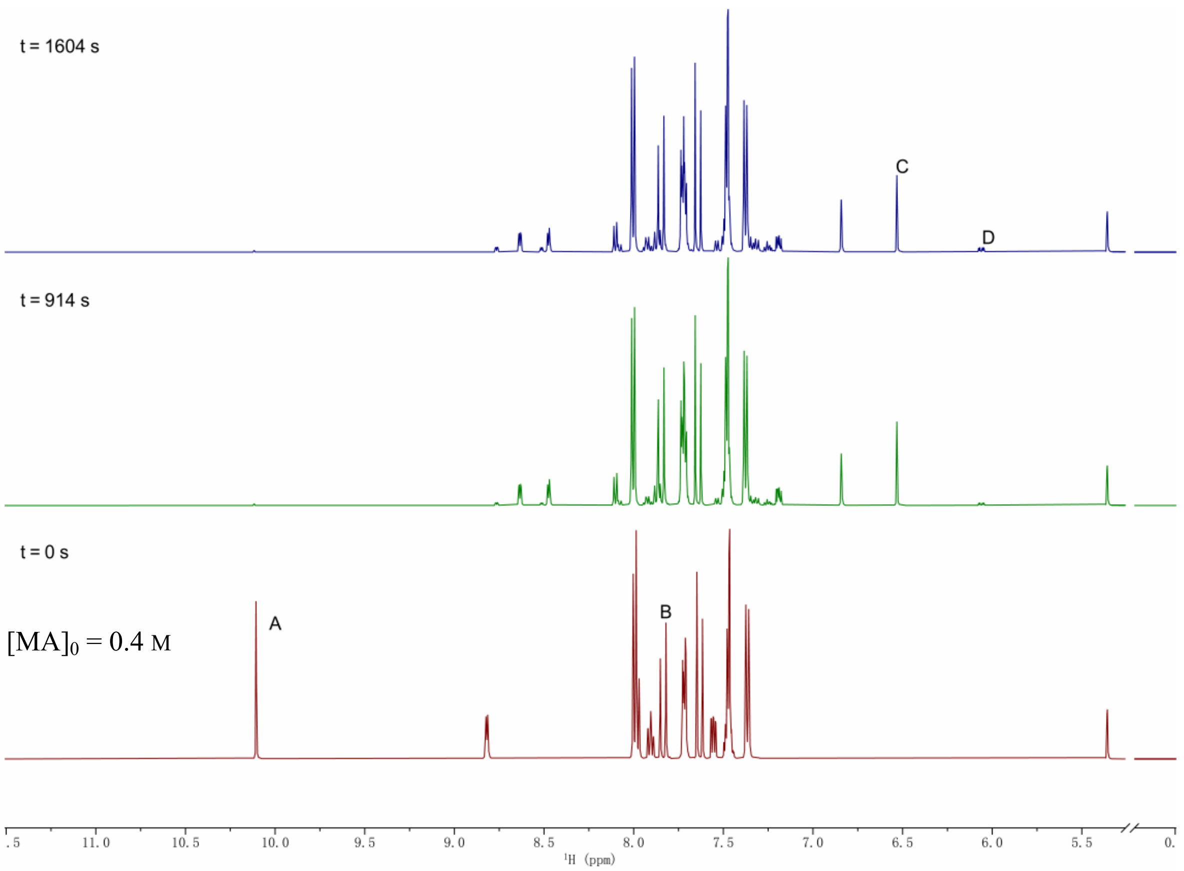

Figure S23. Representative ^{1}H NMR spectra (500 MHz) for reaction of pyridine-2-carboxaldehyde **14** (0.3 M) and chalcone **29** (0.2 M, 0.3 M and 0.4 M) catalysed by *N*-C_6F_5 NHC precursor **16** (5 mM) in NEt_3:NEt_3·HCl (2:1, 0.03 M) in CD_2Cl_2 at 25 °C. A = Aldehyde C*H*O, B = Chalcone C*H*, C = Benzoin C*H*, D = Stetter product C*H*.

Table S10. Reaction data and initial rates of Stetter product formation from (E)-1-(4-methylphenyl)-3-phenylprop-2-en-1-one **29**, catalyzed by C_6F_5 triazolium precatalyst **16** (5 mM) in 0.03 M triethylamine buffer (Et_3N:Et_3N·HCl, 2:1) in CD_2Cl_2 at 25 °C.

**[Aldehyde]$_0$ / M**	**[MA]$_0$ / M**	**Time / s**	**[Benzoin] / M**	**[Stetter] / M**	v_{max} **/ 10^{-5} M s^{-1}**	k'_s **/ 10^{-5} s^{-1}**	**Average / 10^{-5} s^{-1}**
0.3	0.2	0	0	0	1.07	5.35	5.17±0.13
		359	0.149072	0.003028			
		497	0.150121	0.004884			
		635	0.147664	0.006493			
		773	0.145426	0.008082			
		911	0.142658	0.009598			
		1049	0.140248	0.011026			
		1187	0.138136	0.012417			
		1325	0.136182	0.013775			
		1463	0.134183	0.015108			
		1601	0.13191	0.016468			
0.3	0.3	0	0	0	1.54	5.13	

		353	0.148711	0.004146			
		491	0.148809	0.006774			
		629	0.146145	0.009277			
		767	0.143223	0.011573			
		905	0.140294	0.013753			
		1043	0.13803	0.015796			
		1181	0.134817	0.017602			
		1319	0.132761	0.019585			
		1457	0.130296	0.021417			
		1595	0.128038	0.023263			
0.3	0.4	0	0	0	2.01	5.03	
		362	0.144279	0.005822			
		500	0.144337	0.009345			
		638	0.140812	0.012487			
		776	0.137817	0.015459			
		914	0.134328	0.018138			
		1052	0.131718	0.02081			
		1190	0.129041	0.023459			
		1328	0.126664	0.026055			
		1466	0.123949	0.02845			
		1604	0.121333	0.030719			

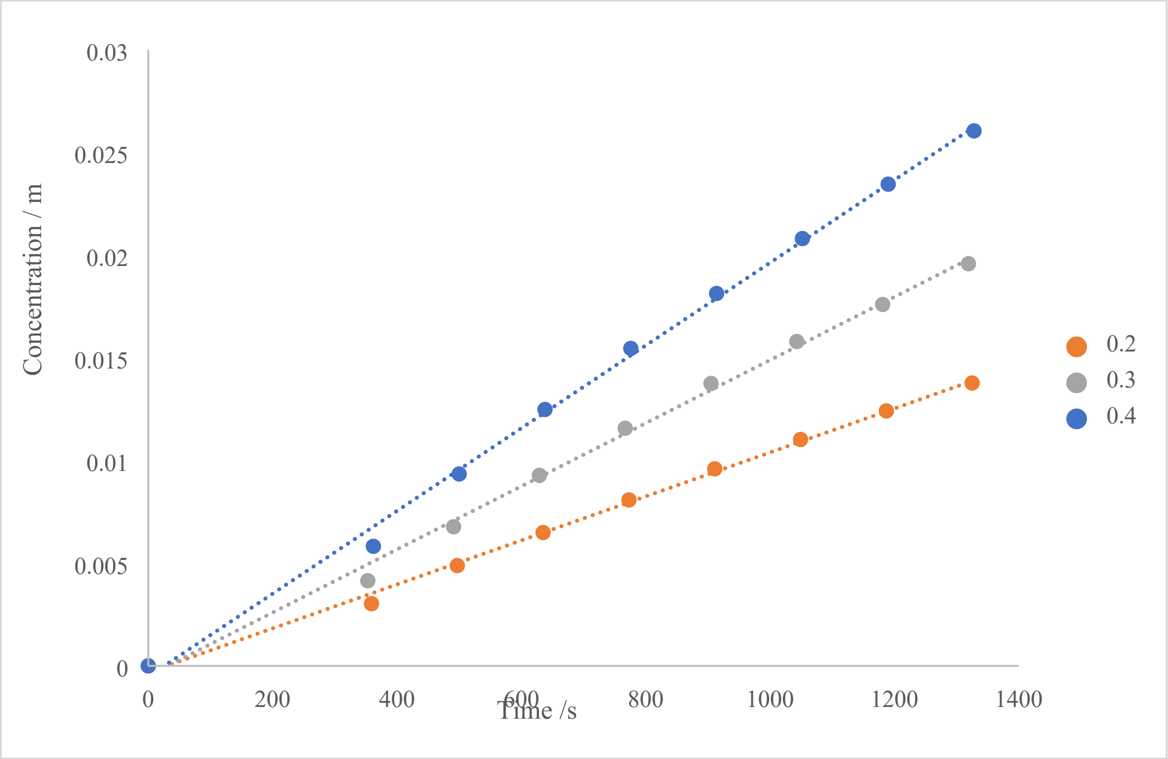

Figure S24. Plots of Stetter product concentration against time for the C_6F_5 triazolium precatalyst **16** (5 mM) catalysed Stetter reaction, at initial (E)-1-(4-methylphenyl)-3-phenylprop-2-en-1-one **29** concentrations 0.2

M, 0.3 M, 0.4 M.

Entry 11

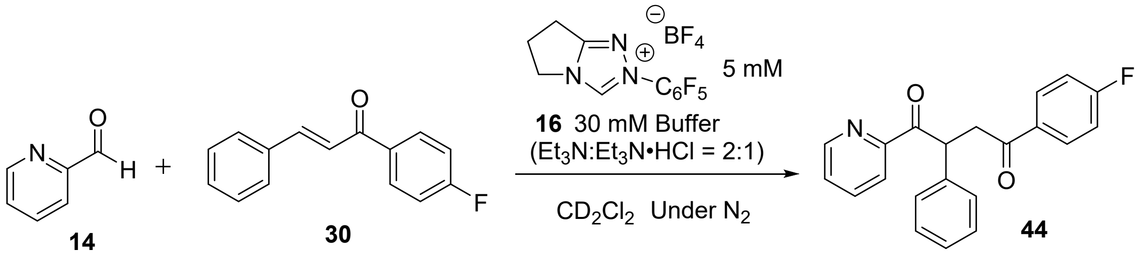

The reaction of pyridine-2-carboxaldehyde **14** and chalcone **30** catalysed by C_6F_5 triazolium salt **16** in triethylamine buffer was monitored using ^1H NMR spectra, with representative NMR spectra over the course of the experiment given in Figure S25.

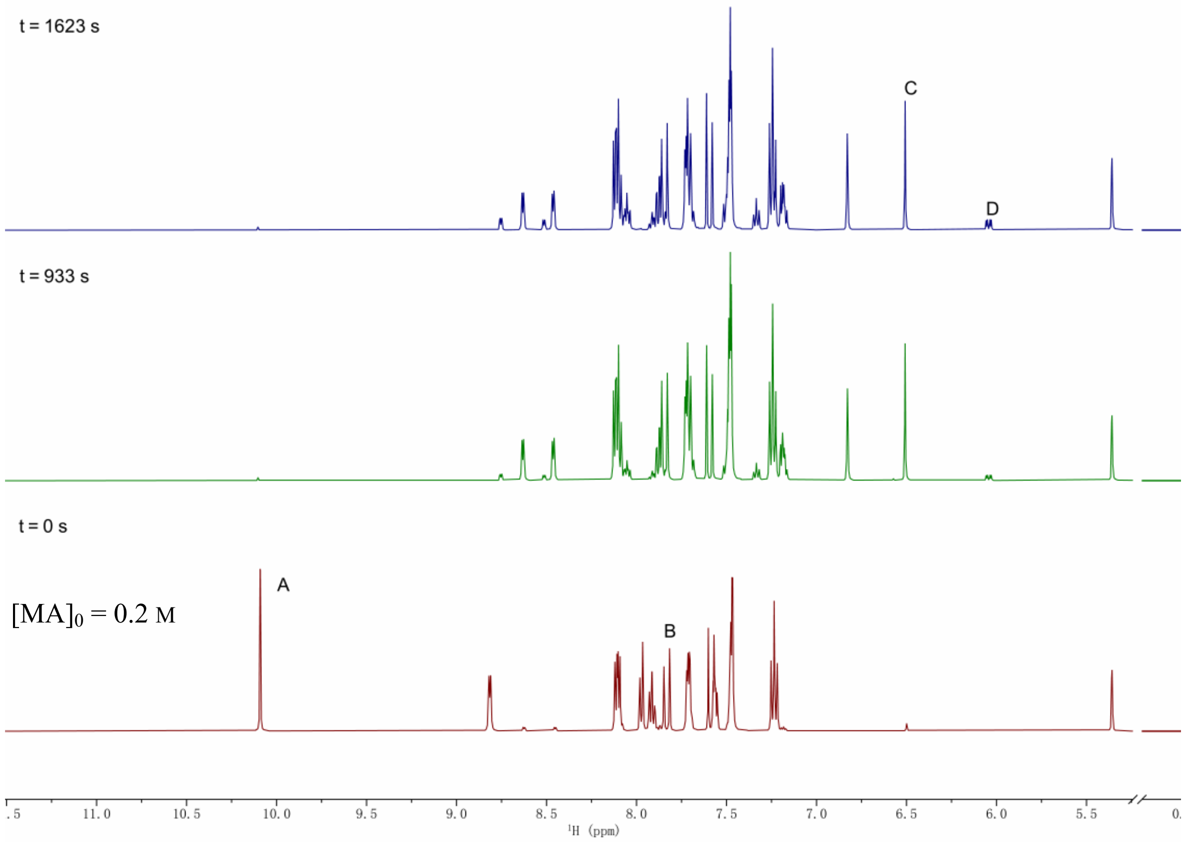

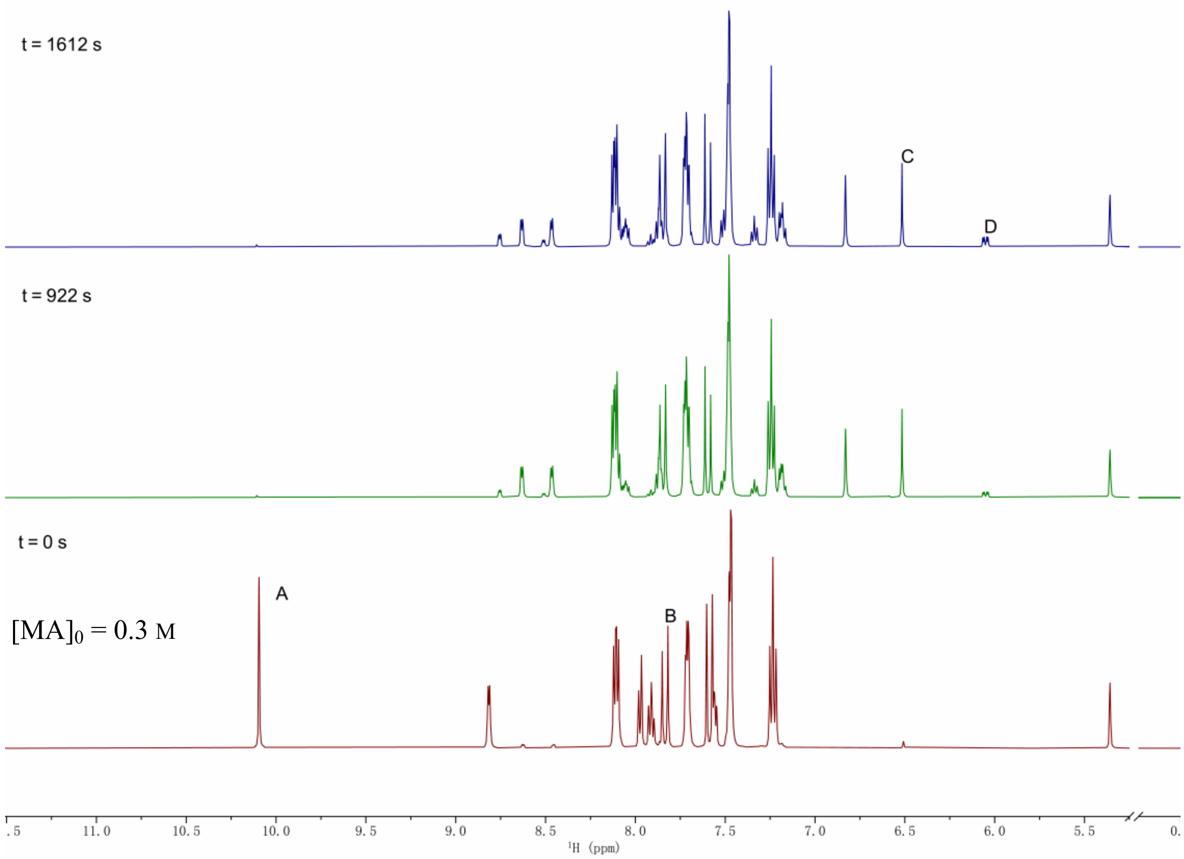

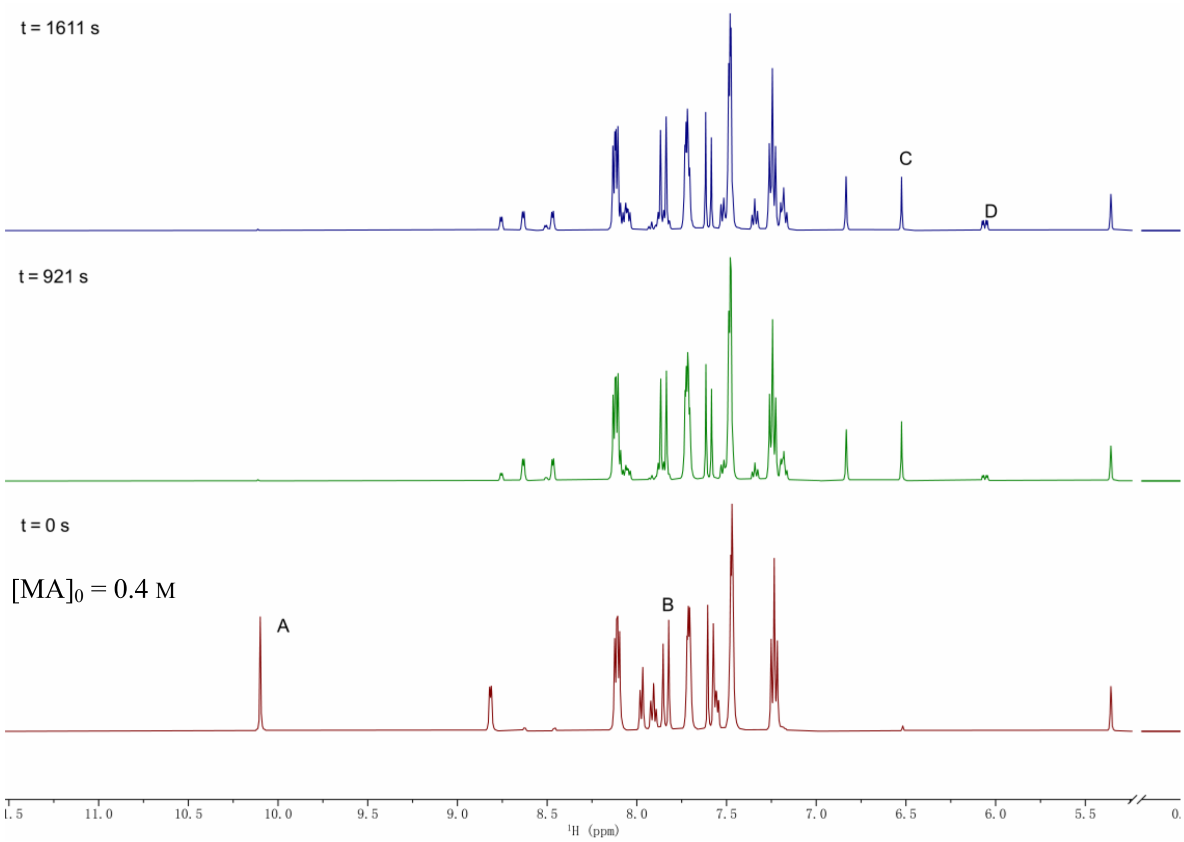

Figure S25. Representative ^{1}H NMR spectra (500 MHz) for reaction of pyridine-2-carboxaldehyde **14** (0.3 M) and chalcone **30** (0.2 M, 0.3 M and 0.4 M) catalysed by *N*-C_6F_5 NHC precursor **16** (5 mM) in NEt_3:NEt_3·HCl

(2:1, 0.03 M) in CD_2Cl_2 at 25 °C. A = Aldehyde C*H*O, B = Chalcone C*H*, C = Benzoin C*H*, D = Stetter product C*H*.

Table S11. Reaction data and initial rates of Stetter product formation from (E)-1-(4-fluorophenyl)-3-phenylprop-2-en-1-one **30**, catalyzed by C_6F_5 triazolium precatalyst **16** (5 mM) in 0.03 M triethylamine buffer (Et_3N:Et_3N·HCl, 2:1) in CD_2Cl_2 at 25 °C.

**[Aldehyde]$_0$ / M**	**[MA]$_0$ / M**	**Time / s**	**[Benzoin] / M**	**[Stetter] / M**	v_{max} **/ 10^{-5} M s^{-1}**	k_s' **/ 10^{-5} s^{-1}**	**Average / 10^{-5} s^{-1}**
0.3	0.2	0	0	0	2.42	12.10	12.07±0.29
		381	0.152946	0.007731			
		519	0.153205	0.011902			
		657	0.149786	0.015619			
		795	0.146101	0.019194			
		933	0.142997	0.022498			
		1071	0.140613	0.025787			
		1209	0.137389	0.028796			
		1347	0.133987	0.031629			
		1485	0.130937	0.034586			
		1623	0.128042	0.037064			
0.3	0.3	0	0	0	3.52	11.70	
		370	0.15	0.010392			
		508	0.148462	0.016715			
		646	0.143969	0.022105			
		784	0.141211	0.027218			
		922	0.137229	0.031958			
		1060	0.13374	0.036755			
		1198	0.129648	0.041262			
		1336	0.126346	0.04571			
		1474	0.122542	0.049495			
		1612	0.119216	0.053144			
0.3	0.4	0	0	0	4.95	12.40	
		369	0.147517	0.014714			
		507	0.145031	0.023091			
		645	0.139422	0.030764			
		783	0.135246	0.038103			
		921	0.129398	0.044987			
		1059	0.124806	0.051691			
		1197	0.121783	0.058583			
		1335	0.116729	0.064597			
		1473	0.113168	0.071032			
		1611	0.108146	0.076484			

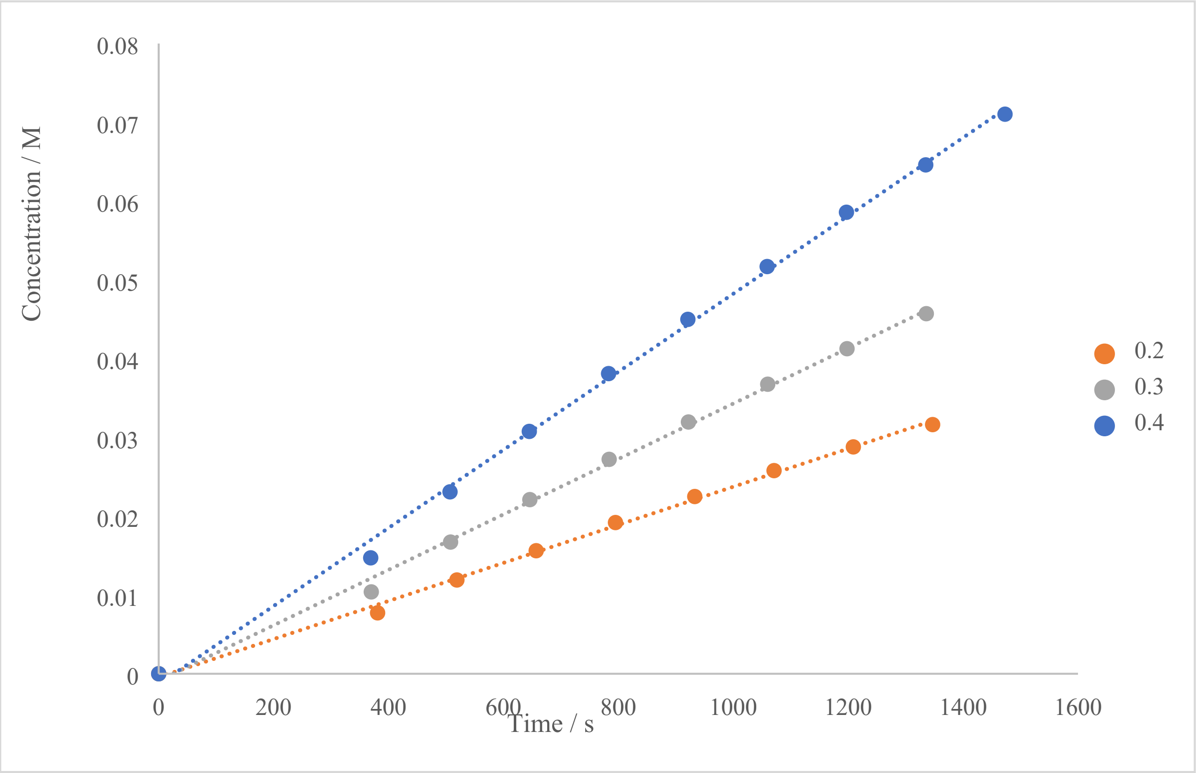

Figure S26. Plots of Stetter product concentration against time for the C_6F_5 triazolium precatalyst **16** (5 mM) catalysed Stetter reaction, at initial (E)-1-(4-fluorophenyl)-3-phenylprop-2-en-1-one **30** concentrations 0.2 M, 0.3 M, 0.4 M.

Entry 12

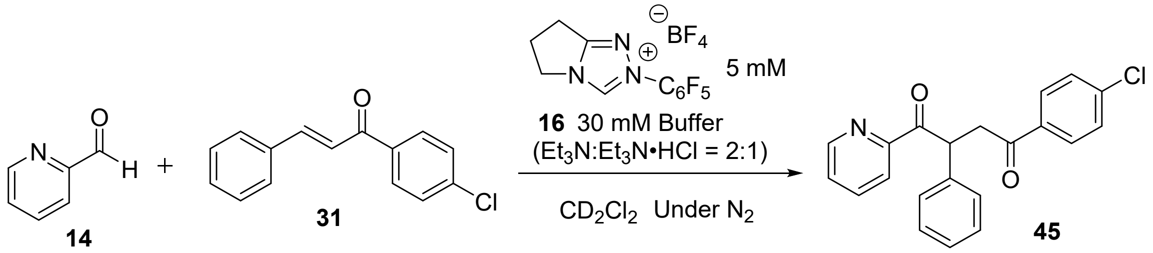

The reaction of pyridine-2-carboxaldehyde **14** and chalcone **31** catalysed by C_6F_5 triazolium salt **16** in triethylamine buffer was monitored using ^{1}H NMR spectra, with representative NMR spectra over the course of the experiment given in Figure S27.

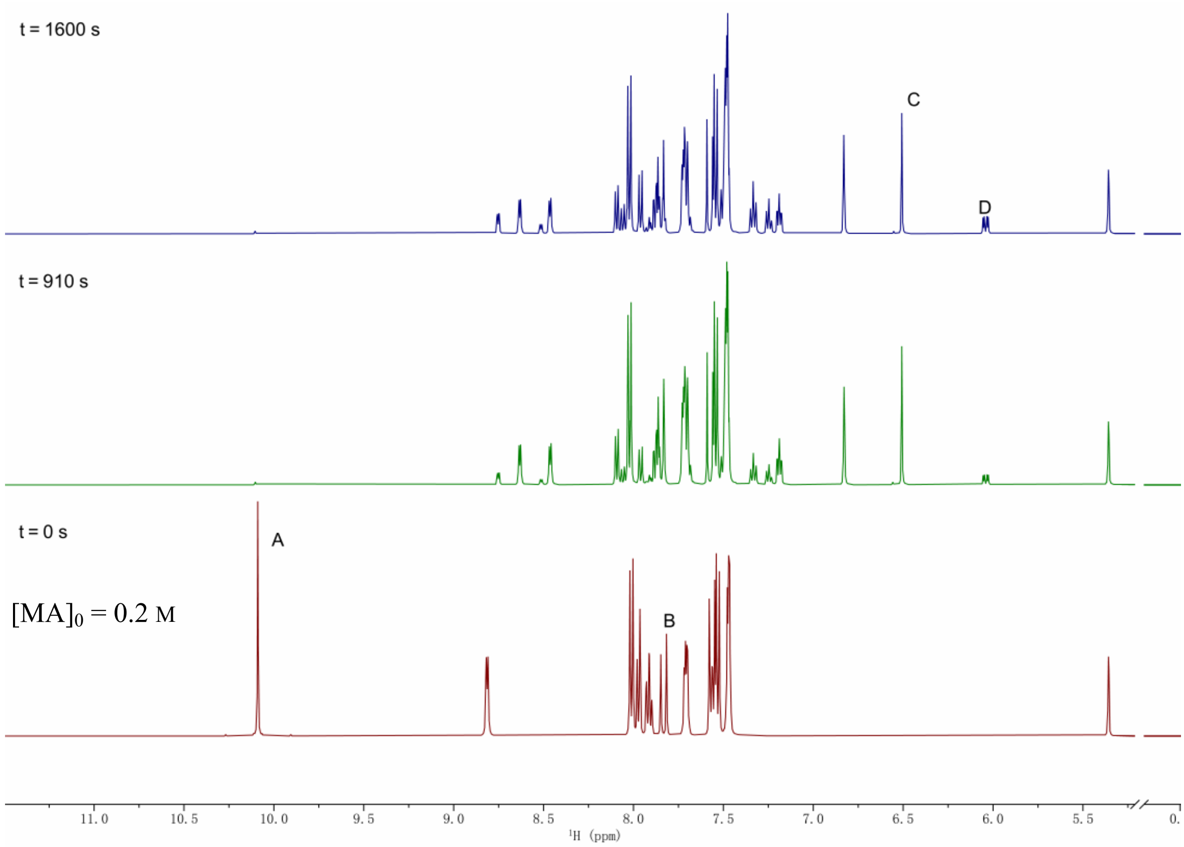
t = 1600 s
C
D
t = 910 s
t = 0 s
A
[MA]0 = 0.2 M
B
11.0 10.5 10.0 9.5 9.0 8.5 8.0 7.5 7.0 6.5 6.0 5.5 0.
1H (ppm)

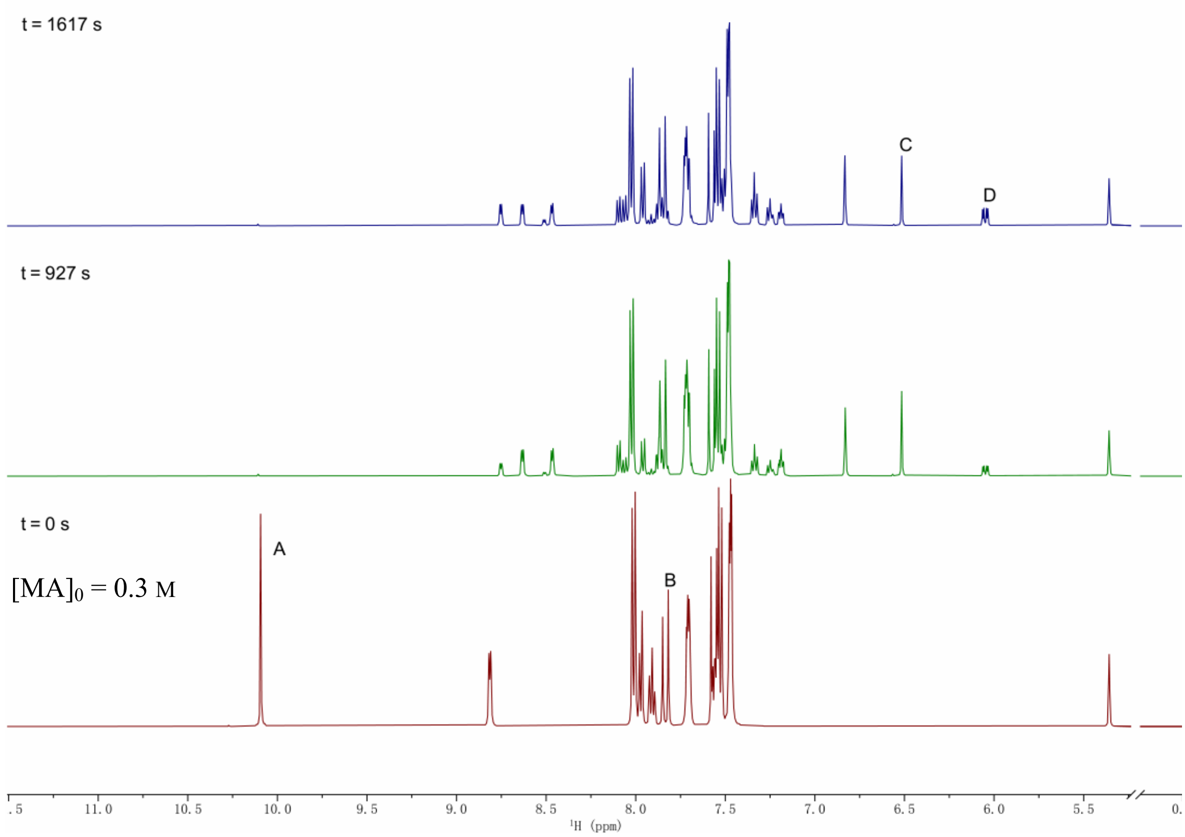
t = 1617 s
C
D
t = 927 s
t = 0 s
A
[MA]0 = 0.3 M
B
.5 11.0 10.5 10.0 9.5 9.0 8.5 8.0 7.5 7.0 6.5 6.0 5.5 0.
1H (ppm)

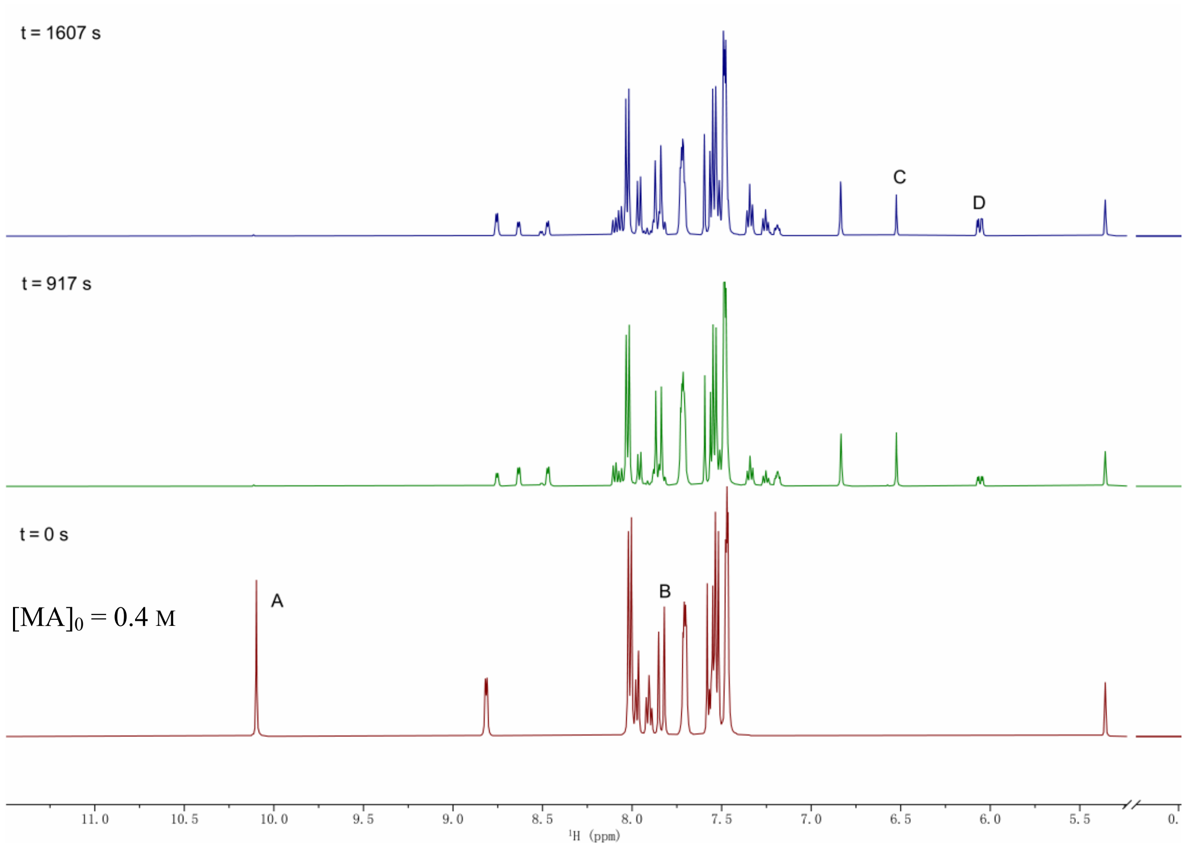

Figure S27. Representative ^{1}H NMR spectra (500 MHz) for reaction of pyridine-2-carboxaldehyde **14** (0.3 M) and chalcone **31** (0.2 M, 0.3 M and 0.4 M) catalysed by *N*-C_6F_5 NHC precursor **16** (5 mM) in NEt_3:NEt_3·HCl (2:1, 0.03 M) in CD_2Cl_2 at 25 °C. A = Aldehyde C*H*O, B = Chalcone C*H*, C = Benzoin C*H*, D = Stetter product C*H*.

Table S12. Reaction data and initial rates of Stetter product formation from (E)-1-(4-chlorophenyl)-3-phenylprop-2-en-1-one **31**, catalyzed by C_6F_5 triazolium precatalyst **16** (5 mM) in 0.03 M triethylamine buffer (Et_3N:Et_3N·HCl, 2:1) in CD_2Cl_2 at 25 °C.

**[Aldehyde]$_0$ / M**	**[MA]$_0$ / M**	**Time / s**	**[Benzoin] / M**	**[Stetter] / M**	**v_{max} / 10^{-5} M s^{-1}**	**k_s' / 10^{-5} s^{-1}**	**Average / 10^{-5} s^{-1}**
		0	0	0			
		358	0.148641	0.011655			
		496	0.147044	0.019257			
		634	0.142146	0.026146			
		772	0.137524	0.032712			
0.3	0.2	910	0.133241	0.038578	4.31	21.55	12.07±0.29
		1048	0.128793	0.044404			
		1186	0.124753	0.049756			
		1324	0.120747	0.054926			
		1462	0.117132	0.060096			
		1600	0.113514	0.064695			
0.3	0.3	0	0	0	6.41	21.37	

		375	0.150366	0.016448		
		513	0.147559	0.027916		
		651	0.140415	0.038457		
		789	0.134123	0.048141		
		927	0.127669	0.057335		
		1065	0.122185	0.065926		
		1203	0.116413	0.074314		
		1341	0.111186	0.08221		
		1479	0.106274	0.089876		
		1617	0.101391	0.096977		
0.3	0.4	0	0	0	8.33	20.83
		365	0.13769	0.023889		
		503	0.132439	0.038345		
		641	0.124783	0.051378		
		779	0.117485	0.063806		
		917	0.110517	0.075387		
		1055	0.104138	0.086500		
		1193	0.097951	0.097109		
		1331	0.092050	0.107232		
		1469	0.086360	0.116798		
		1607	0.081062	0.125781		

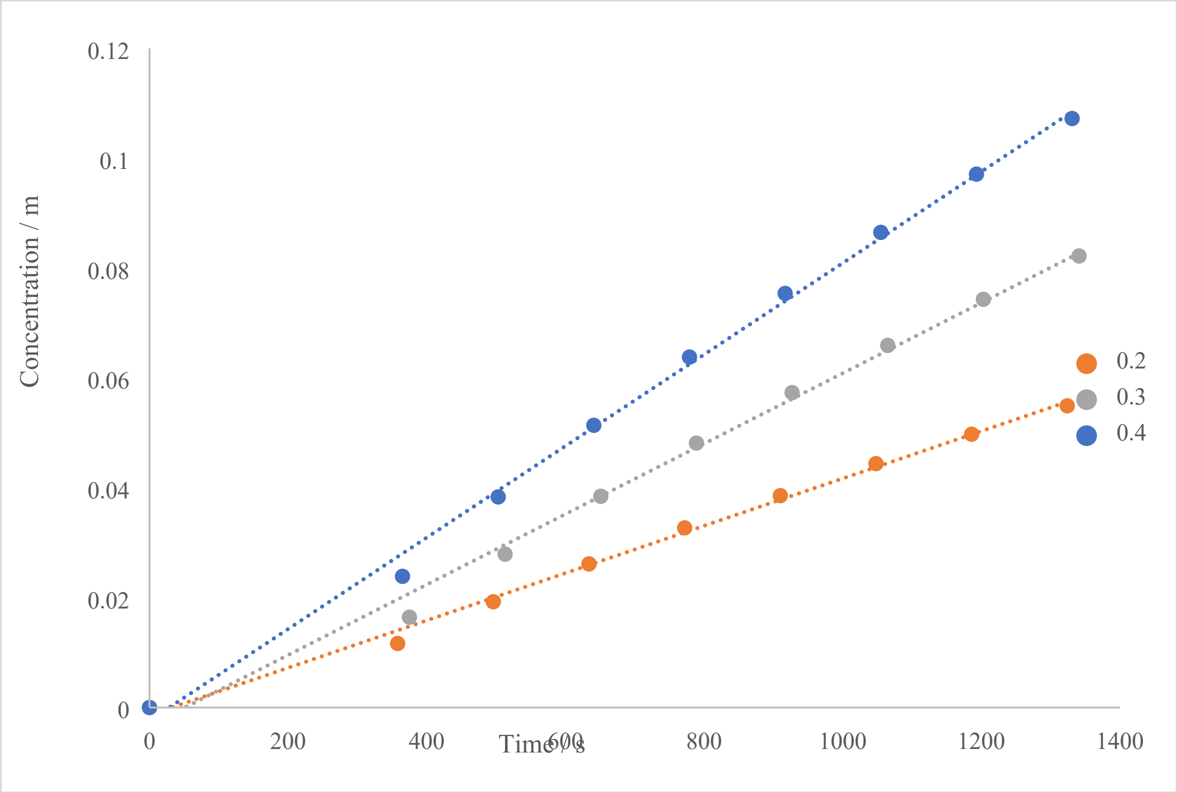

Figure S28. Plots of Stetter product concentration against time for the C_6F_5 triazolium precatalyst **16** (5 mM)

catalysed Stetter reaction, at initial (E)-1-(4-chlorophenyl)-3-phenylprop-2-en-1-one **31** concentrations 0.2 M, 0.3 M, 0.4 M.

Entry 13

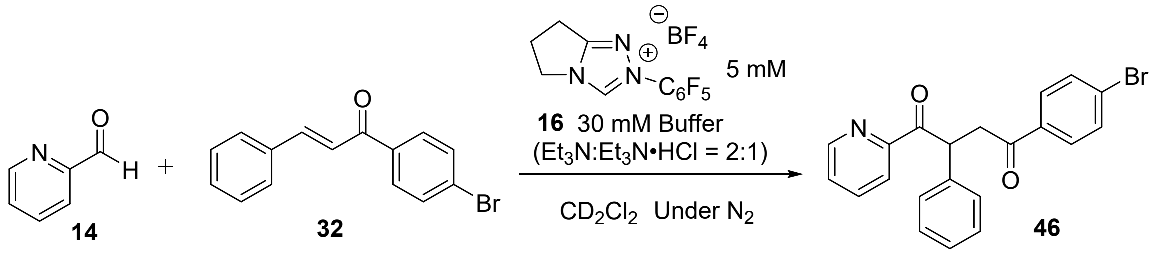

The reaction of pyridine-2-carboxaldehyde **14** and chalcone **32** catalysed by C_6F_5 triazolium salt **16** in triethylamine buffer was monitored using ^{1}H NMR spectra, with representative NMR spectra over the course of the experiment given in Figure S29.

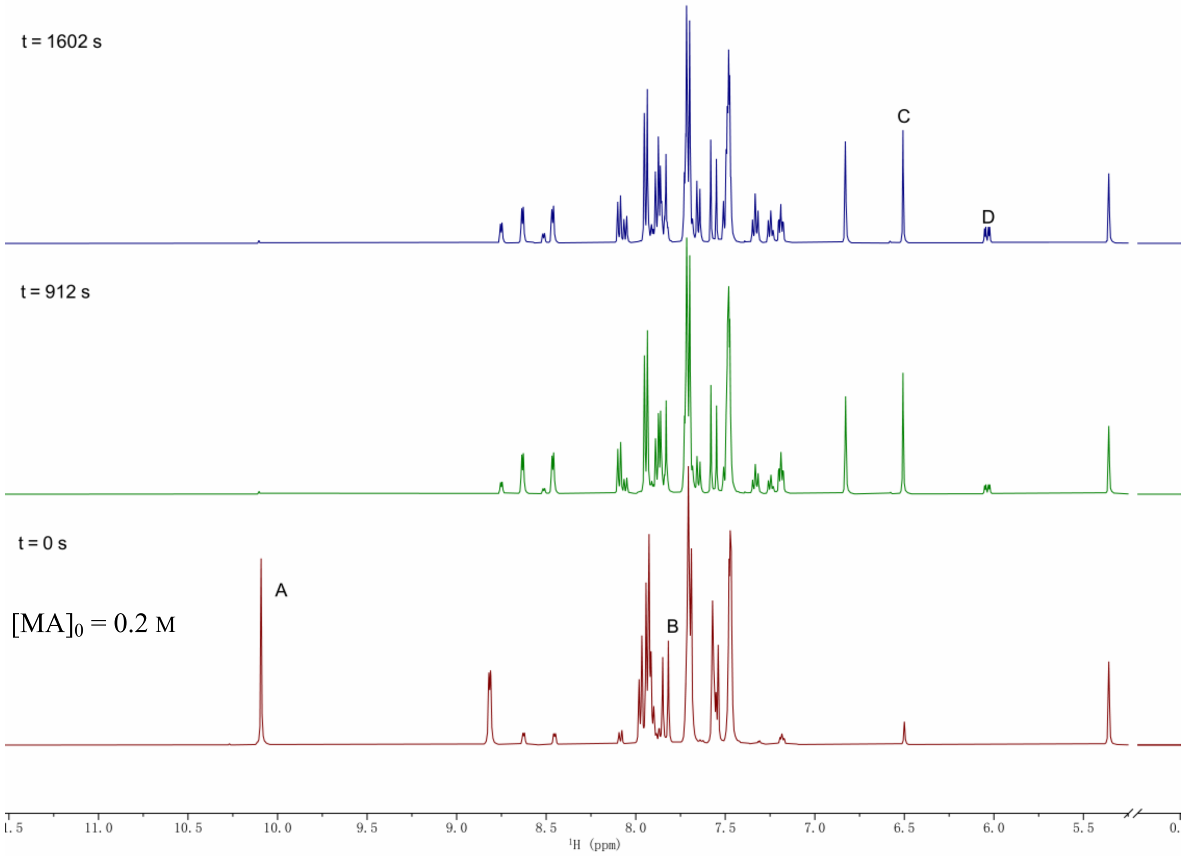

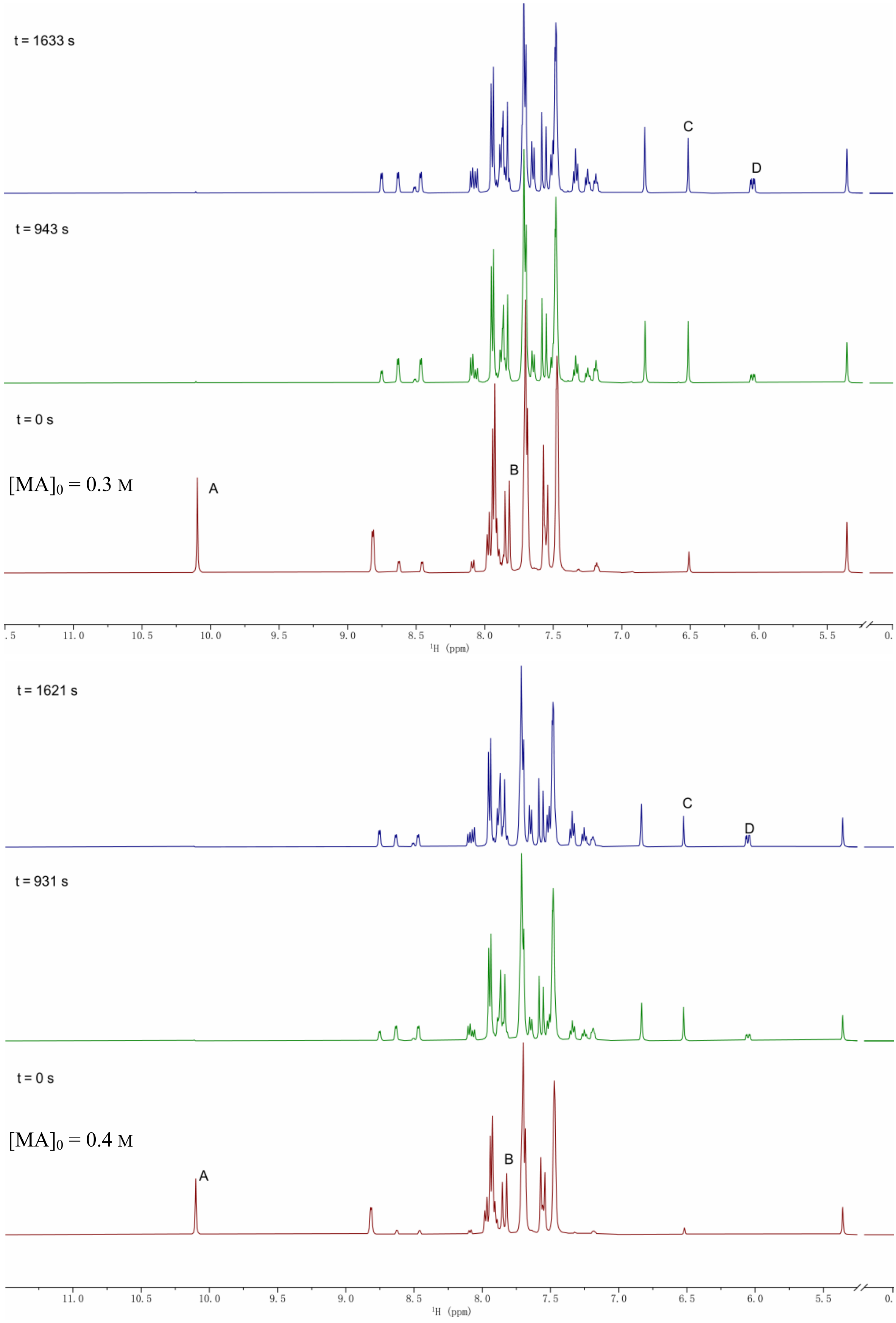

Figure S29. Representative ^{1}H NMR spectra (500 MHz) for reaction of pyridine-2-carboxaldehyde **14** (0.3 M) and chalcone **32** (0.2 M, 0.3 M and 0.4 M) catalysed by *N*-C_6F_5 NHC precursor **16** (5 mM) in NEt_3:NEt_3·HCl

(2:1, 0.03 M) in CD_2Cl_2 at 25 °C. A = Aldehyde C*H*O, B = Chalcone C*H*, C = Benzoin C*H*, D = Stetter product C*H*.

Table S13. Reaction data and initial rates of Stetter product formation from (E)-1-(4-bromophenyl)-3-phenylprop-2-en-1-one **32**, catalyzed by C_6F_5 triazolium precatalyst **16** (5 mM) in 0.03 M triethylamine buffer (Et_3N:$Et_3N{\cdot}HCl$, 2:1) in CD_2Cl_2 at 25 °C.

**[Aldehyde]$_0$ / M**	**[MA]$_0$ / M**	**Time / s**	**[Benzoin] / M**	**[Stetter] / M**	v_{max} **/ 10^{-5} M s^{-1}**	k_s' **/ 10^{-5} s^{-1}**	**Average / 10^{-5} s^{-1}**
		0	0	0			
		360	0.14366	0.013846			
		498	0.14188	0.021583			
		636	0.137195	0.028517			
		774	0.133011	0.034881			
0.3	0.2	912	0.128593	0.040674	4.47	22.35	
		1050	0.124744	0.046483			
		1188	0.121171	0.051777			
		1326	0.117714	0.056899			
		1464	0.114473	0.061776			
		1602	0.111278	0.06654			
		0	0	0			
		391	0.133372	0.023084			
		529	0.130574	0.03438			
		667	0.125279	0.044348			
		805	0.119491	0.053107			
0.3	0.3	943	0.114703	0.062066	6.52	21.73	22.23±0.37
		1081	0.109875	0.069953			
		1219	0.105594	0.07805			
		1357	0.101204	0.084523			
		1495	0.097248	0.09228			
		1633	0.093402	0.09868			
		0	0	0			
		379	0.132211	0.026507			
		517	0.12774	0.042547			
		655	0.121284	0.057276			
		793	0.114801	0.070496			
0.3	0.4	931	0.108629	0.082189	9.04	22.60	
		1069	0.102983	0.094849			
		1207	0.097617	0.105112			
		1345	0.092722	0.116931			
		1483	0.087887	0.12614			
		1621	0.083263	0.134702			

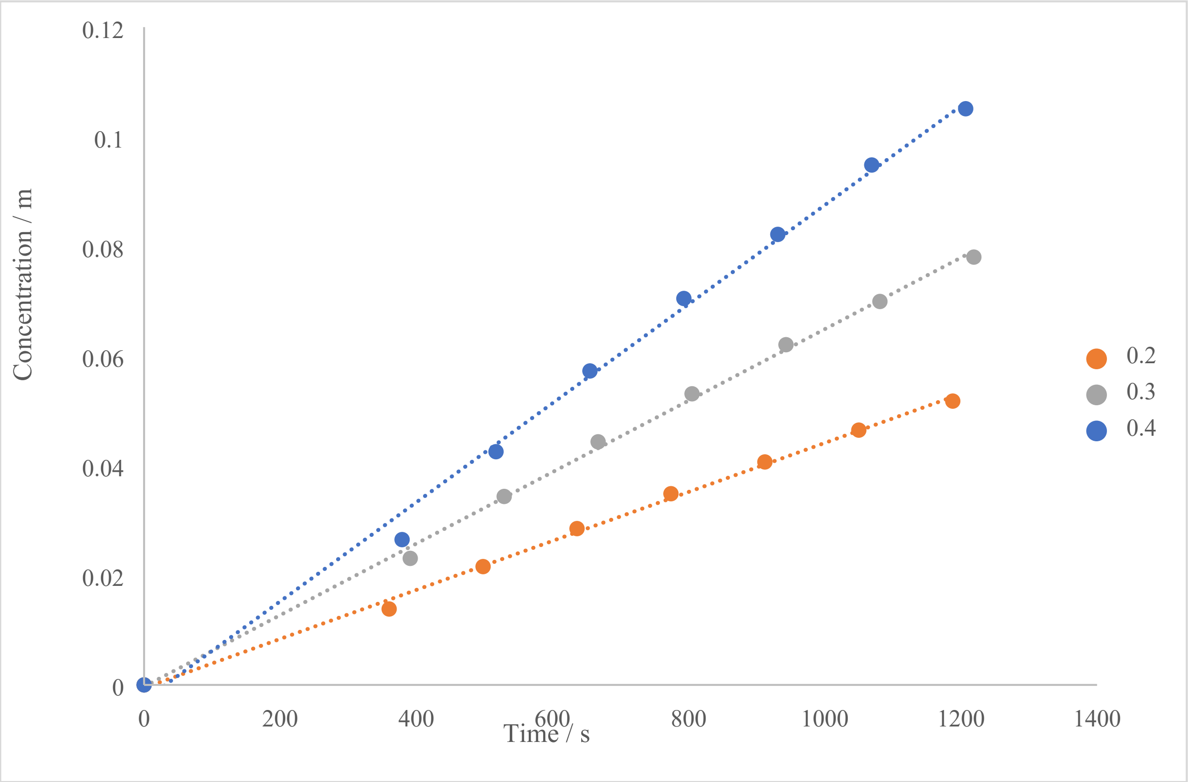

Figure S30. Plots of Stetter product concentration against time for the C_6F_5 triazolium precatalyst **16** (5 mM) catalysed Stetter reaction, at initial (E)-1-(4-bromophenyl)-3-phenylprop-2-en-1-one **32** concentrations 0.2 M, 0.3 M, 0.4 M.

Entry 14

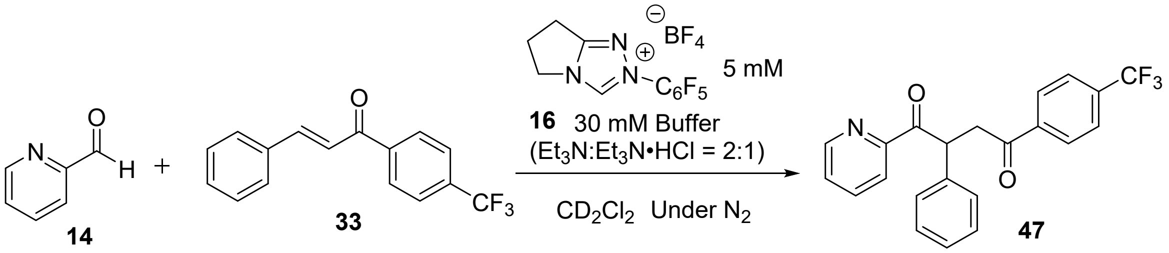

The reaction of pyridine-2-carboxaldehyde **14** and chalcone **33** catalysed by C_6F_5 triazolium salt **16** in triethylamine buffer was monitored using ^1H NMR spectra, with representative NMR spectra over the course of the experiment given in Figure S31.

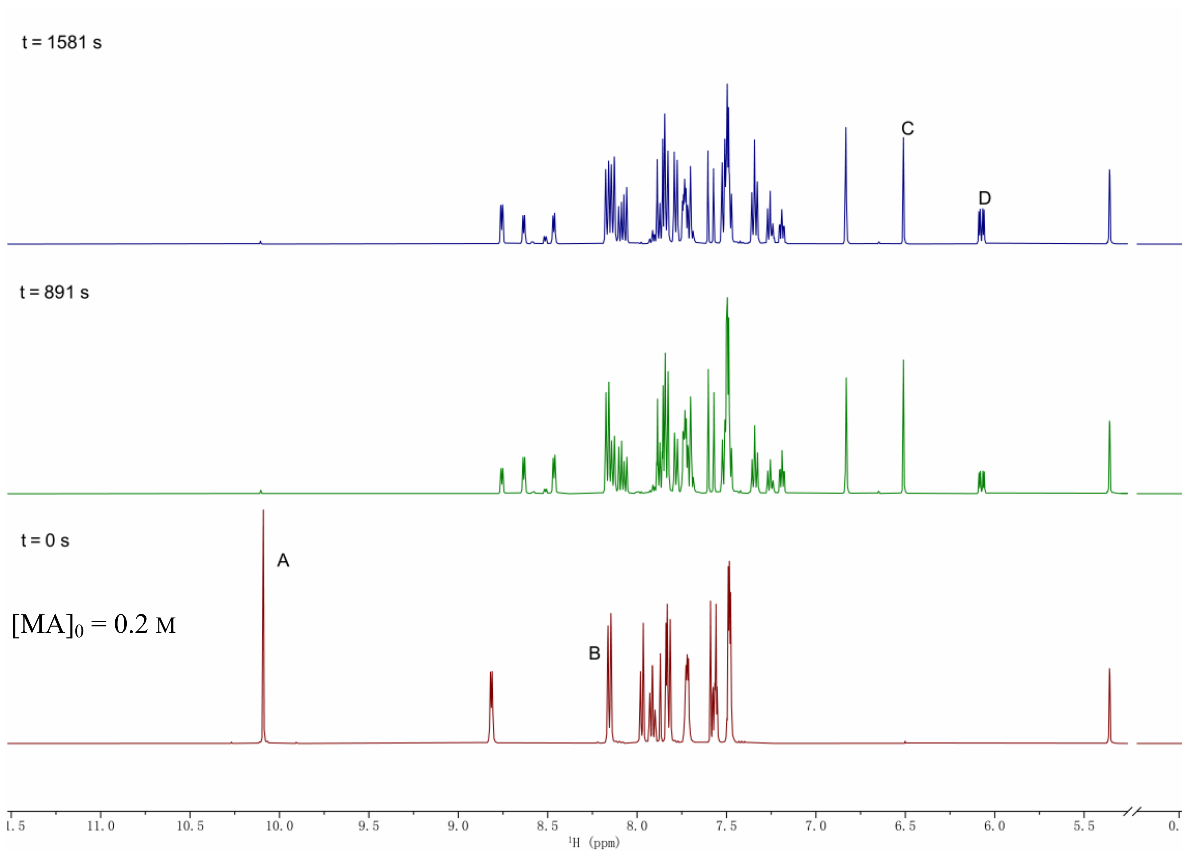
t = 1581 s
C
D
t = 891 s
t = 0 s
A
[MA]$_0$ = 0.2 M
B
11.0
10.5
10.0
9.5
9.0
8.5
8.0
7.5
7.0
6.5
6.0
5.5
^{1}H (ppm)

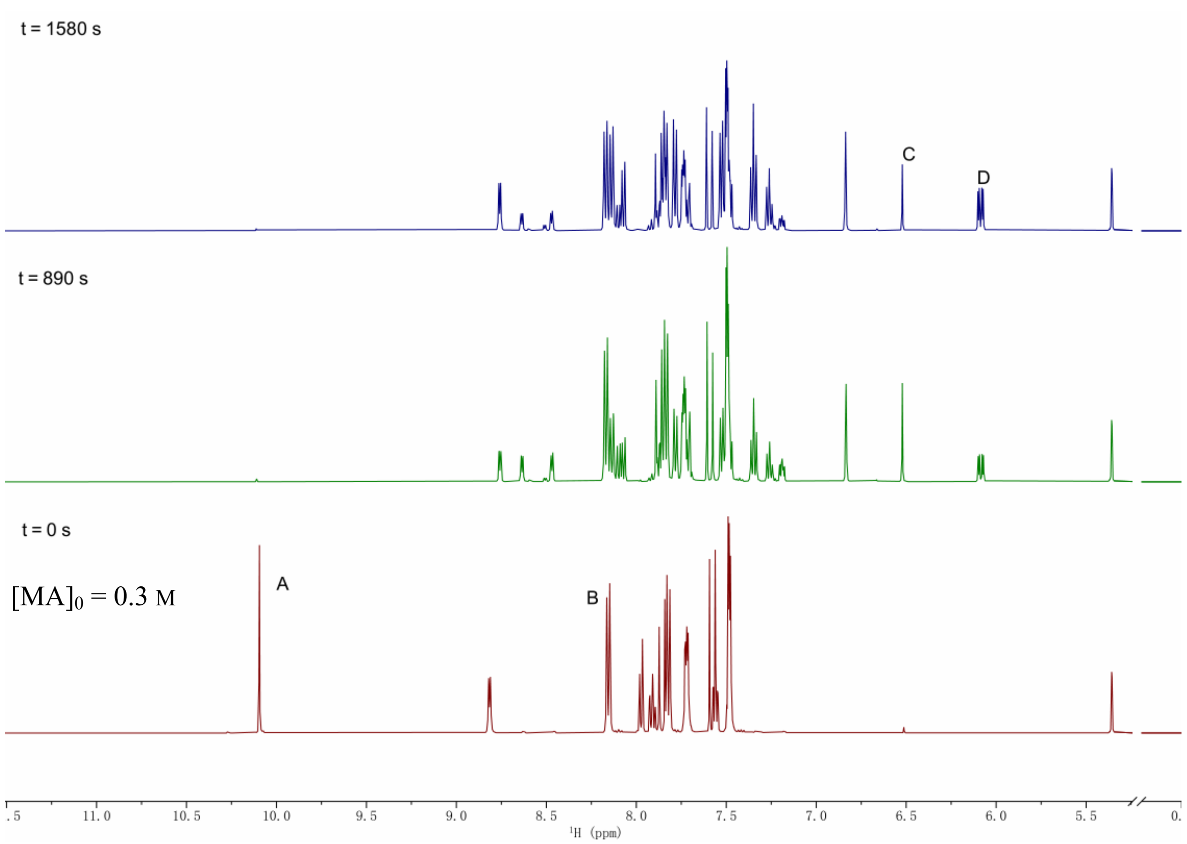
t = 1580 s
C
D
t = 890 s
t = 0 s
A
[MA]$_0$ = 0.3 M
B
11.0
10.5
10.0
9.5
9.0
8.5
8.0
7.5
7.0
6.5
6.0
5.5
^{1}H (ppm)

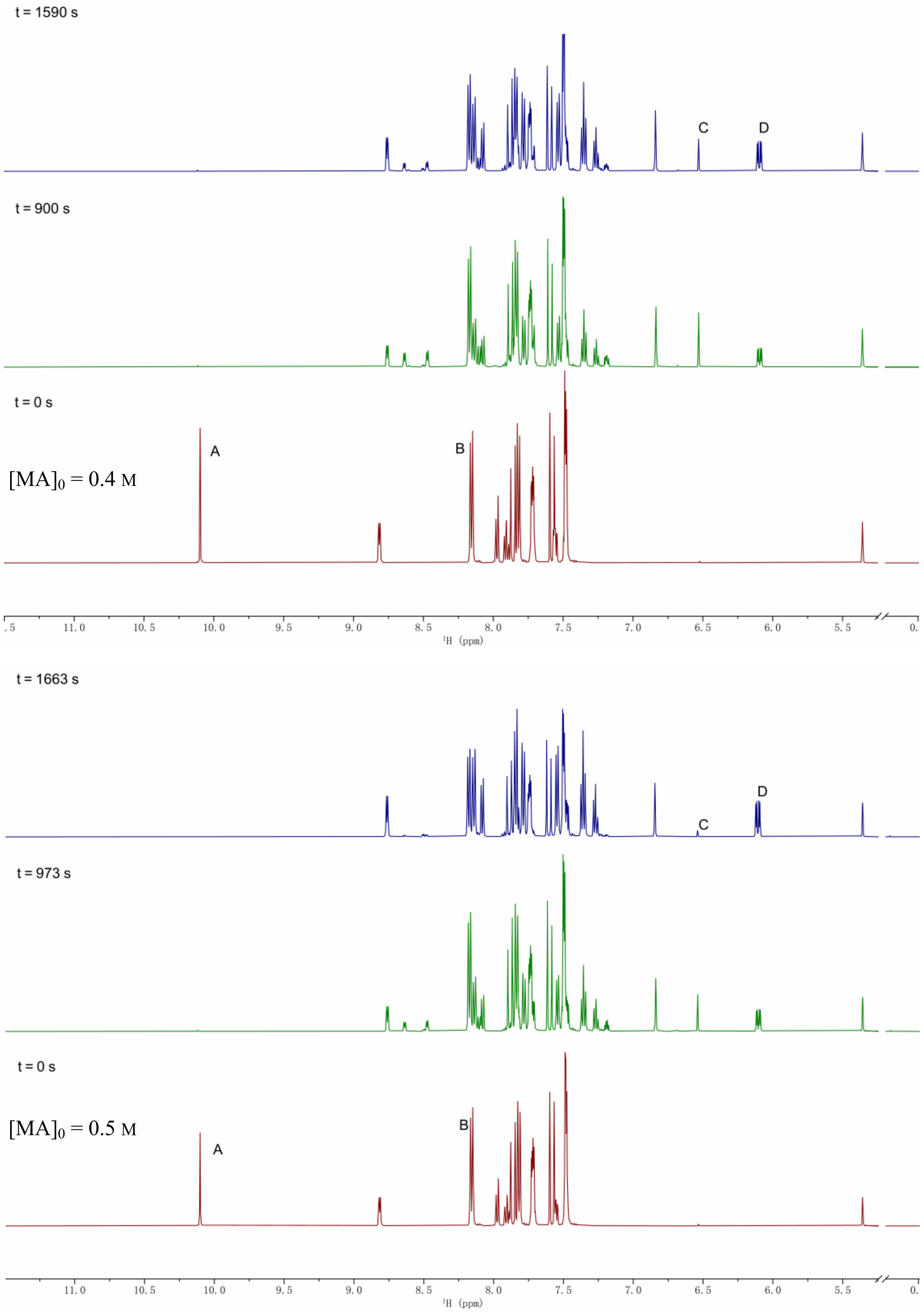

Figure S31. Representative ^{1}H NMR spectra (500 MHz) for reaction of pyridine-2-carboxaldehyde **14** (0.3 M) and chalcone **33** (0.2 M, 0.3 M, 0.4 M and 0.5 M) catalysed by *N*-C_6F_5 NHC precursor **16** (5 mM) in NEt_3:NEt_3·HCl (2:1, 0.03 M) in CD_2Cl_2 at 25 °C. A = Aldehyde C*H*O, B = Chalcone Ph*H*, C = Benzoin C*H*,

D = Stetter product C*H*.

Table S14. Reaction data and initial rates of Stetter product formation from (E)-1-(4-trifluoromethylphenyl)-3-phenylprop-2-en-1-one **33**, catalyzed by C_6F_5 triazolium precatalyst **16** (5 mM) in 0.03 M triethylamine buffer (Et_3N:$Et_3N \cdot HCl$, 2:1) in CD_2Cl_2 at 25 °C.

[Aldehyde]$_0$ / M	[MA]$_0$ / M	Time / s	[Benzoin] / M	[Stetter] / M	v_{max} / 10^{-5} M s^{-1}	k_s' / 10^{-5} s^{-1}	Average / 10^{-5} s^{-1}
0.3	0.2	0	0	0	8.23	41.15	40.89±1.22
		339	0.128786	0.025885			
		477	0.123726	0.039583			
		615	0.116907	0.051497			
		753	0.110725	0.062226			
		891	0.10507	0.072084			
		1029	0.099684	0.080933			
		1167	0.095019	0.089321			
		1305	0.090434	0.096884			
		1443	0.08621	0.10406			
		1581	0.082276	0.110524			
0.3	0.3	0	0	0	11.70	39.00	
		338	0.120511	0.036647			
		476	0.112521	0.0559			
		614	0.103464	0.07278			
		752	0.095025	0.087966			
		890	0.087555	0.102178			
		1028	0.080412	0.114728			
		1166	0.074037	0.126998			
		1304	0.068078	0.138152			
		1442	0.062496	0.148171			
		1580	0.057146	0.158139			
0.3	0.4	0	0	0	16.40	41.00	
		348	0.115959	0.052279			
		486	0.10658	0.079424			
		624	0.09651	0.10301			
		762	0.087058	0.124874			
		900	0.078704	0.144994			
		1038	0.070636	0.163282			
		1176	0.063348	0.179828			
		1314	0.056513	0.196017			
		1452	0.05045	0.210925			
		1590	0.044707	0.22443			
0.3	0.5	0	0	0	21.20	42.40	
		421	0.09796	0.088627			

		559	0.087591	0.122858			
		697	0.076512	0.151864			
		835	0.066458	0.178529			
		973	0.05716	0.203554			
		1111	0.04058	0.239846			
		1249	0.034153	0.265364			
		1387	0.022671	0.288893			
		1525	0.013815	0.314026			
		1663	0.009716	0.33308			

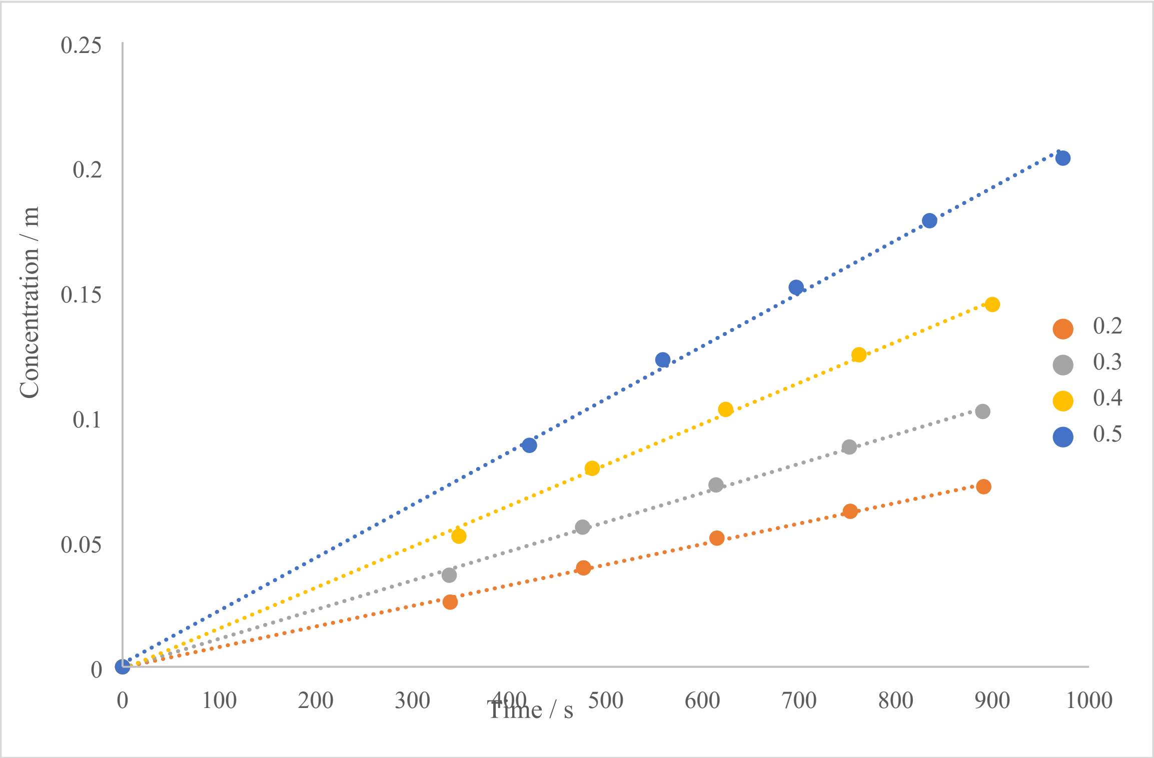

Figure S32. Plots of Stetter product concentration against time for the C_6F_5 triazolium precatalyst **16** (5 mM) catalysed Stetter reaction, at initial (E)-1-(4-trifluoromethylphenyl)-3-phenylprop-2-en-1-one **33** concentrations 0.2 M, 0.3 M, 0.4 M, 0.5 M.

Entry 15

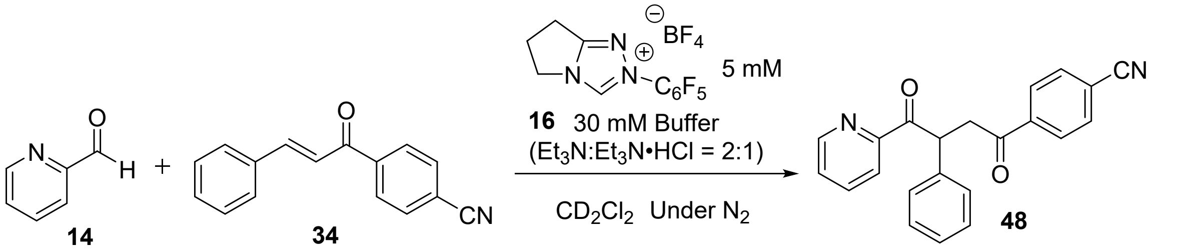

The reaction of pyridine-2-carboxaldehyde **14** and chalcone **34** catalysed by C_6F_5 triazolium salt **16** in triethylamine buffer was monitored using ^1H NMR spectra, with representative NMR spectra over the course

of the experiment given in Figure S33.

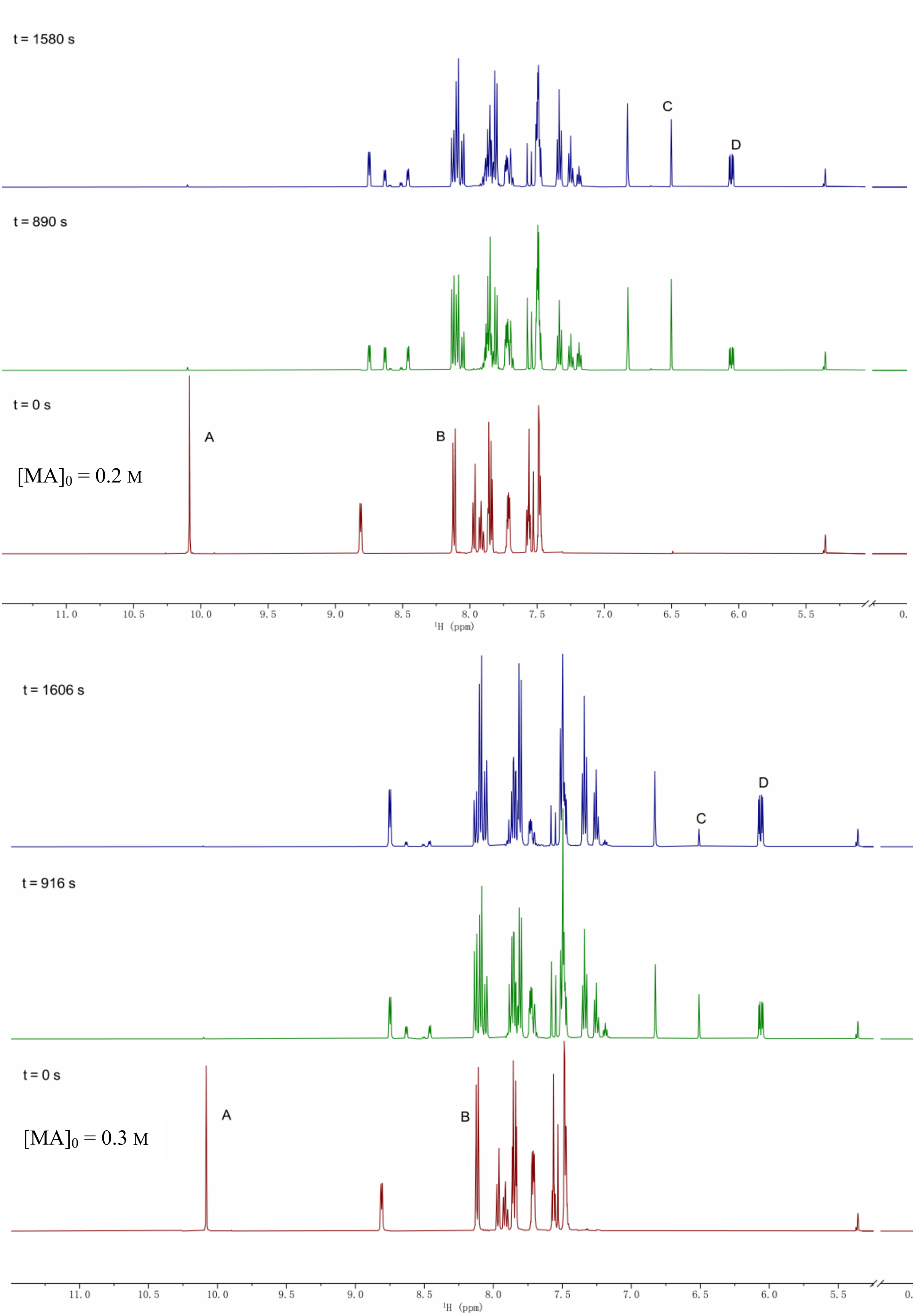

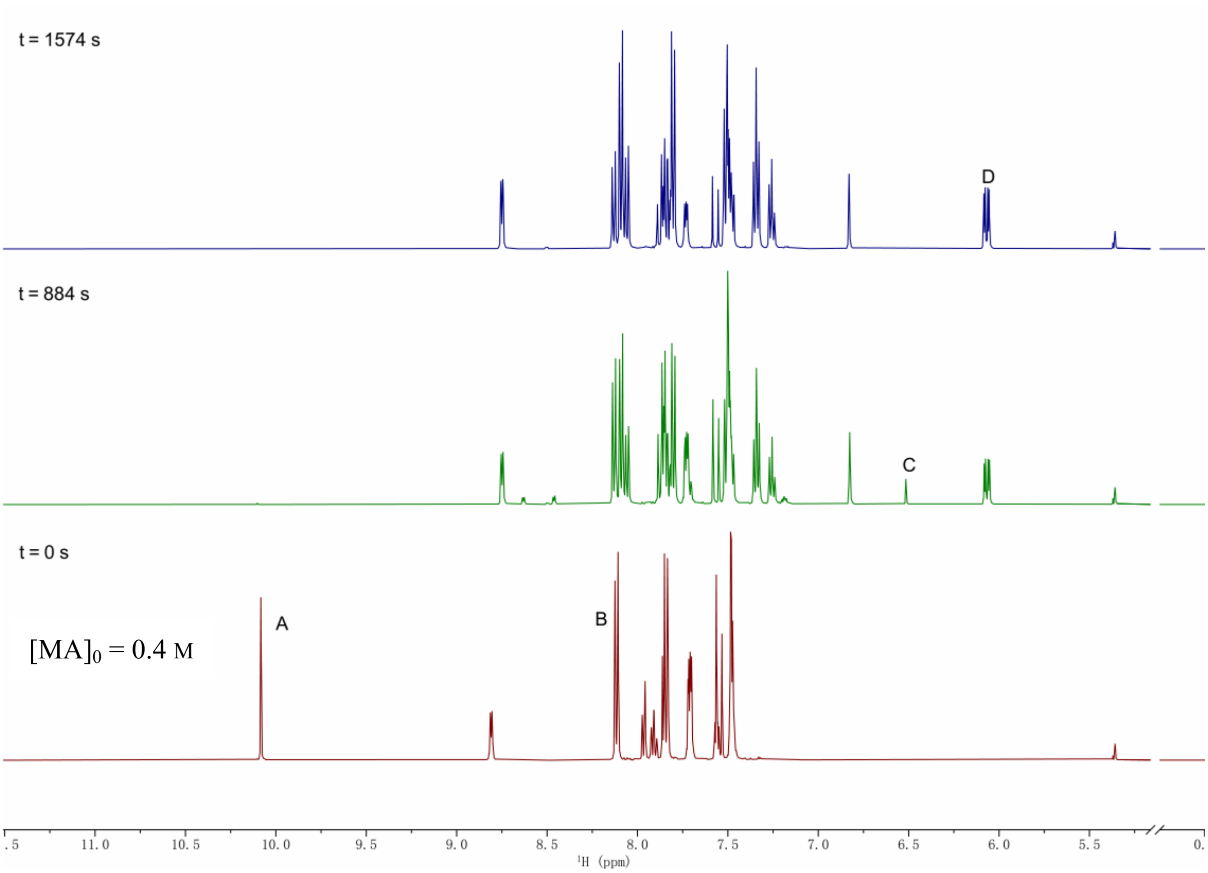

Figure S33. Representative ^{1}H NMR spectra (500 MHz) for reaction of pyridine-2-carboxaldehyde **14** (0.3 M) and chalcone **34** (0.2 M, 0.3 M and 0.4 M) catalysed by *N*-C_6F_5 NHC precursor **16** (5 mM) in NEt_3:NEt_3·HCl (2:1, 0.03 M) in CD_2Cl_2 at 25 °C. A = Aldehyde C*H*O, B = Chalcone Ph*H*, C = Benzoin C*H*, D = Stetter product C*H*.

Table S15. Reaction data and initial rates of Stetter product formation from chalcone **34** and pyridine-2-carboxaldehyde **14**, catalysed by C_6F_5 triazolium precatalyst **16** (5 mM) in 0.03 M triethylamine buffer (Et_3N:Et_3N·HCl, 2:1) in CD_2Cl_2 at 25 °C.

**[Aldehyde]$_0$ / M**	**[MA]$_0$ / M**	**Time / s**	**[Benzoin] / M**	**[Stetter] / M**	v_{max} / **10^{-5} M s^{-1}**	k'_S / **10^{-5} s^{-1}**	**Average / 10^{-5} s^{-1}**
0.3	0.2	0	0	0	11.80	59.00	65.68±4.84
		338	0.124263	0.038885			
		476	0.116256	0.057859			
		614	0.107808	0.074132			
		752	0.100393	0.087749			
		890	0.093705	0.099897			
		1028	0.087906	0.110462			
		1166	0.08269	0.120003			
		1304	0.077857	0.128066			
		1442	0.073758	0.135825			
		1580	0.069919	0.14257			
0.3	0.3	0	0	0	21.10	70.33	

		364	0.104629	0.075328			
		502	0.08981	0.109846			
		640	0.075983	0.13745			
		778	0.064135	0.161457			
		916	0.054278	0.181647			
		1054	0.045482	0.199429			
		1192	0.037976	0.214716			
		1330	0.031513	0.227811			
		1468	0.026051	0.238845			
		1606	0.021299	0.247914			
0.3	0.4	0	0	0	27.10	67.75	
		332	0.098686	0.083559			
		470	0.078084	0.129089			
		608	0.059896	0.16643			
		746	0.044451	0.198703			
		884	0.031232	0.22621			
		1022	0.019737	0.251085			
		1160	0.01013	0.271206			
		1298	0.002597	0.287862			
		1436	0.000126	0.296472			
		1574	-3.5E-05	0.2971			

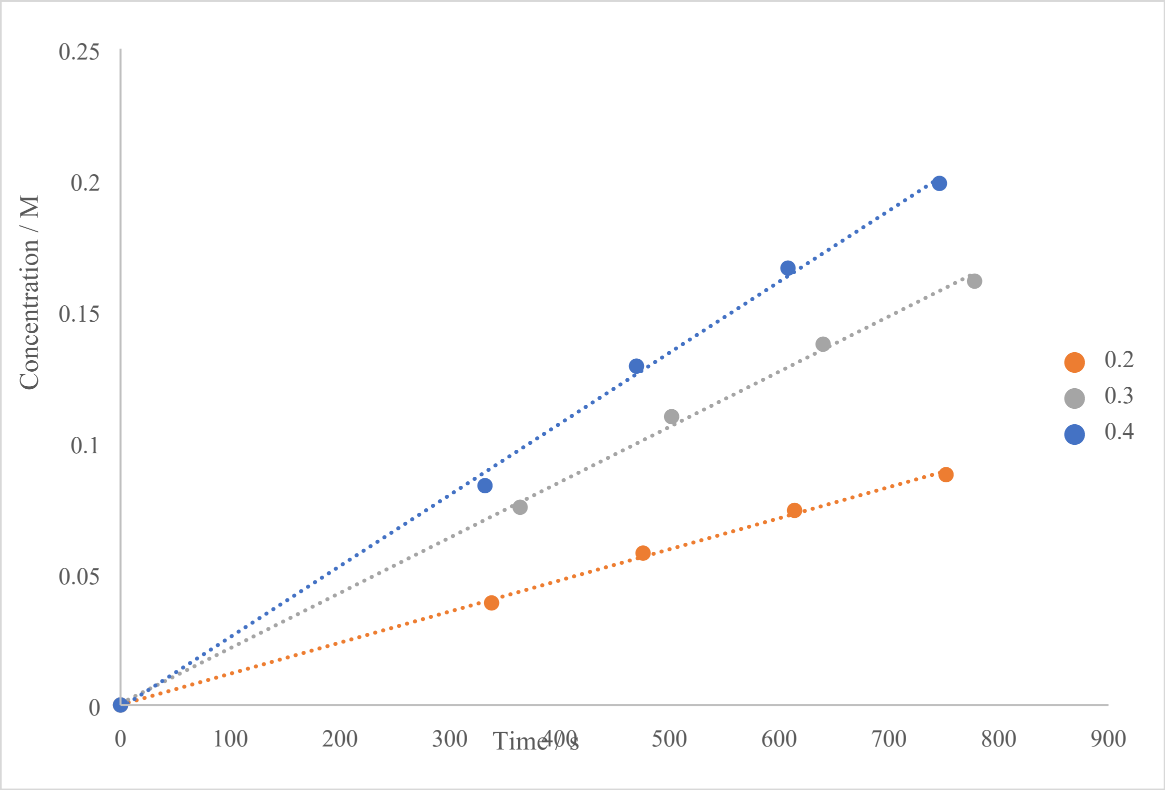

Figure S34. Plots of Stetter product concentration against time for the C_6F_5 triazolium precatalyst **16** (5 mM) catalysed Stetter reaction, at initial chalcone **34** concentrations 0.2 M, 0.3 M, 0.4 M.

Entry 16

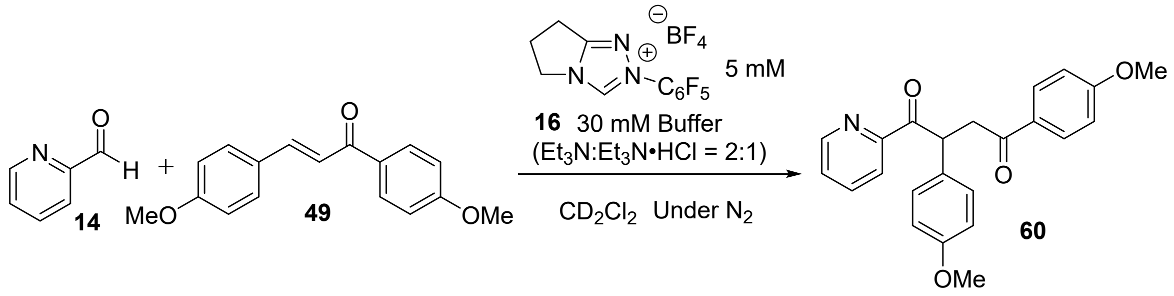

The reaction of pyridine-2-carboxaldehyde **14** and chalcone **49** catalysed by C_6F_5 triazolium salt **16** in triethylamine buffer was monitored using ^{1}H NMR spectra, with representative NMR spectra over the course of the experiment given in Figure S35.

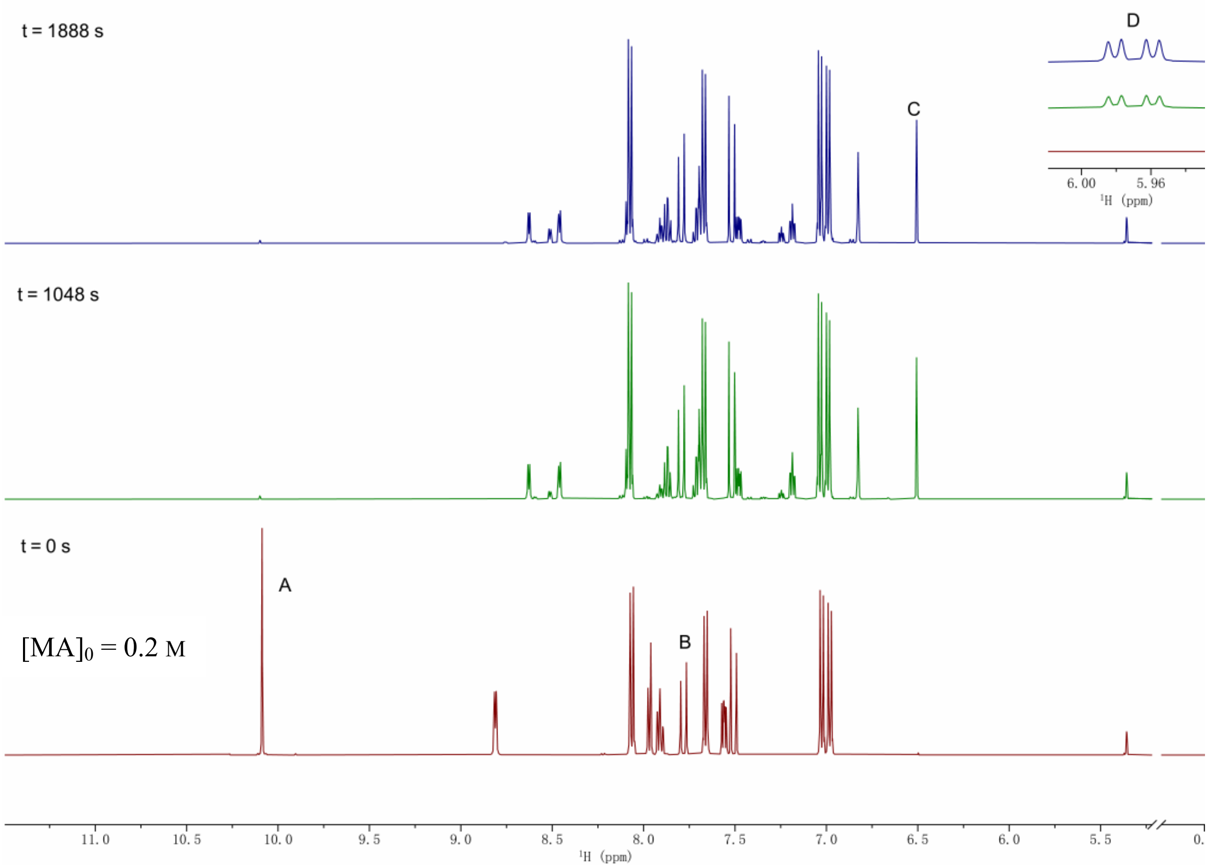

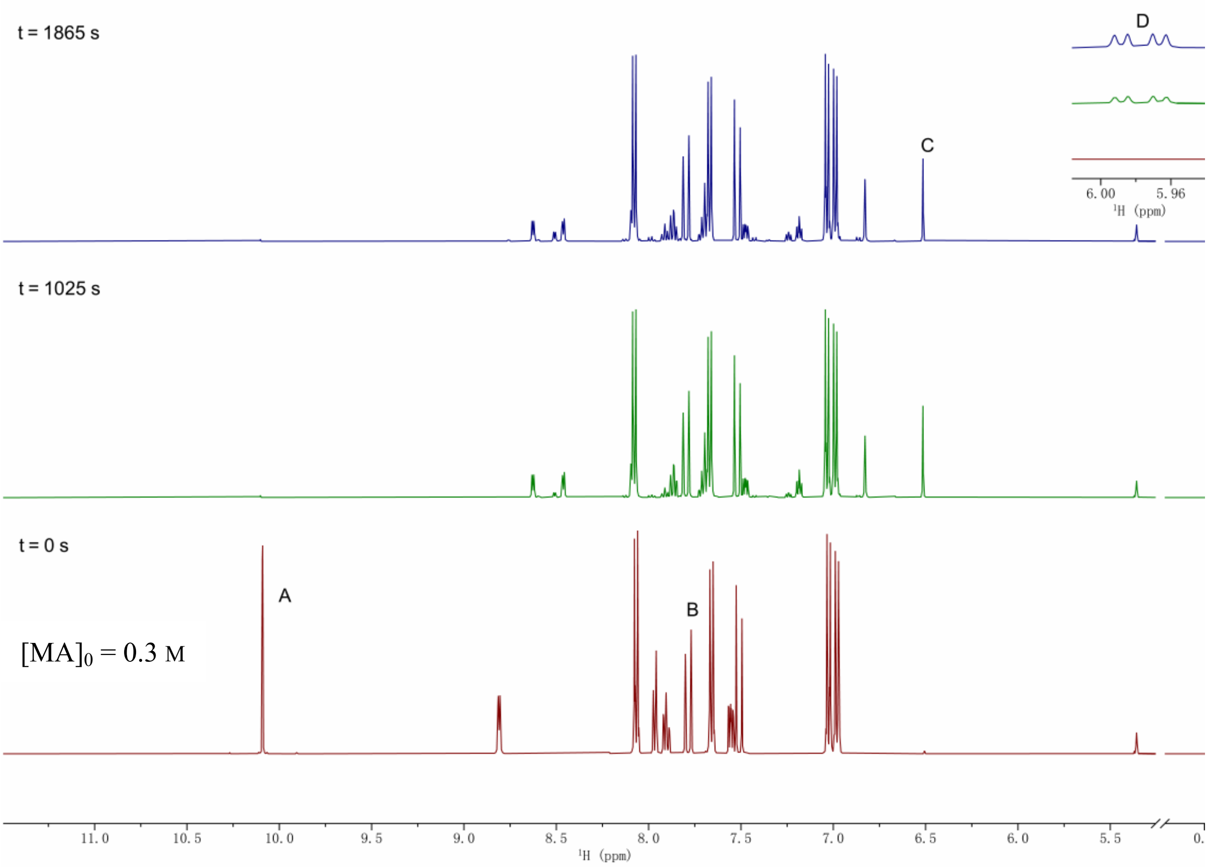

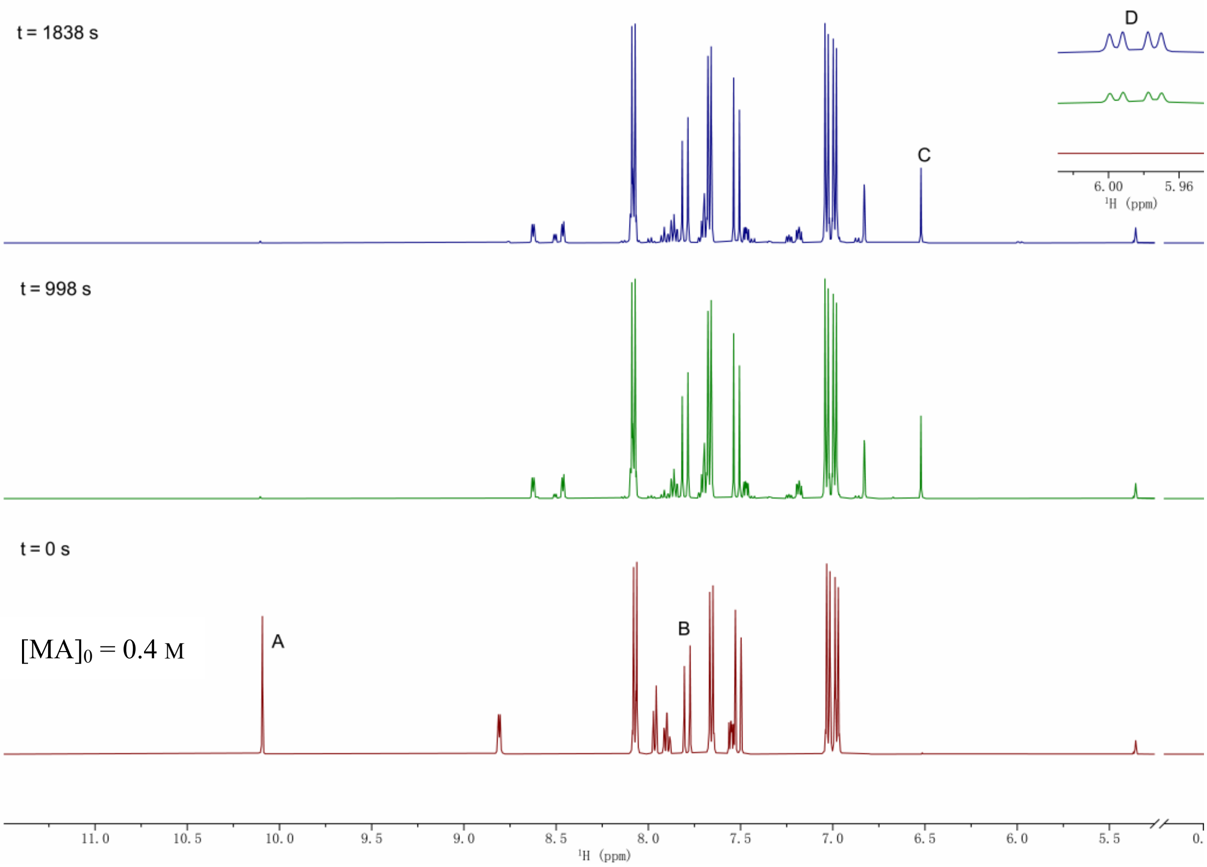

Figure S35. Representative ^{1}H NMR spectra (500 MHz) for reaction of pyridine-2-carboxaldehyde **14** (0.3 M) and chalcone **49** (0.2 M, 0.3 M and 0.4 M) catalysed by *N*-C_6F_5 NHC precursor **16** (5 mM) in NEt_3:NEt_3·HCl

(2:1, 0.03 M) in CD_2Cl_2 at 25 °C. A = Aldehyde C*H*O, B = Chalcone C*H*, C = Benzoin C*H*, D = Stetter product C*H*.

Table S16. Reaction data and initial rates of Stetter product formation from chalcone **49** and pyridine-2-carboxaldehyde **14**, catalysed by C_6F_5 triazolium precatalyst **16** (5 mM) in 0.03 M triethylamine buffer (Et_3N:Et_3N·HCl, 2:1) in CD_2Cl_2 at 25 °C.

**[Aldehyde]$_0$ / M**	**[MA]$_0$ / M**	**Time / s**	**[Benzoin] / M**	**[Stetter] / M**	**v_{max} / 10^{-5} M s^{-1}**	**k'_s / 10^{-5} s^{-1}**	**Average / 10^{-5} s^{-1}**
0.3	0.2	0	0	0	0.339	1.70	1.58±0.12
		376	0.144351	0.001428			
		544	0.139446	0.002213			
		712	0.136059	0.002879			
		880	0.132918	0.003508			
		1048	0.129943	0.003953			
		1216	0.126769	0.004282			
		1384	0.123915	0.004859			
		1552	0.121382	0.005306			
		1720	0.118572	0.005496			
		1888	0.116118	0.006225			
0.3	0.3	0	0	0	0.488	1.63	
		353	0.141935	0.001334			
		521	0.137238	0.002574			
		689	0.133655	0.003457			
		857	0.130544	0.00425			
		1025	0.127564	0.005277			
		1193	0.124481	0.005799			
		1361	0.121536	0.006452			
		1529	0.118745	0.007301			
		1697	0.116164	0.008007			
		1865	0.1134	0.008343			
0.3	0.4	0	0	0	0.567	1.42	
		326	0.140965	0.001678			
		494	0.135917	0.002913			
		662	0.13191	0.003838			
		830	0.129041	0.004724			
		998	0.126832	0.006066			
		1166	0.123553	0.006936			
		1334	0.11992	0.00744			
		1502	0.117117	0.008293			
		1670	0.115191	0.009338			
		1838	0.11102	0.009454			

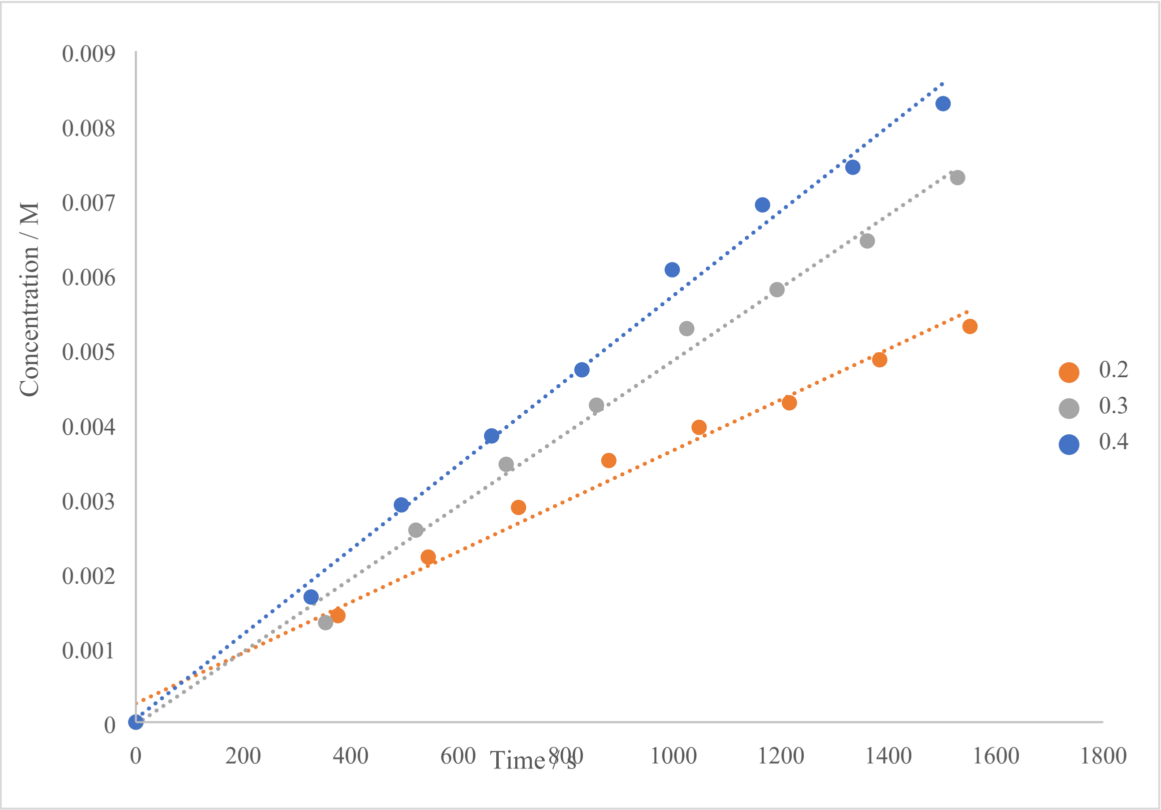

Figure S36. Plots of Stetter product concentration against time for the C_6F_5 triazolium precatalyst **16** (5 mM) catalysed Stetter reaction, at initial chalcone **49** concentrations 0.2 M, 0.3 M, 0.4 M.

Entry 17

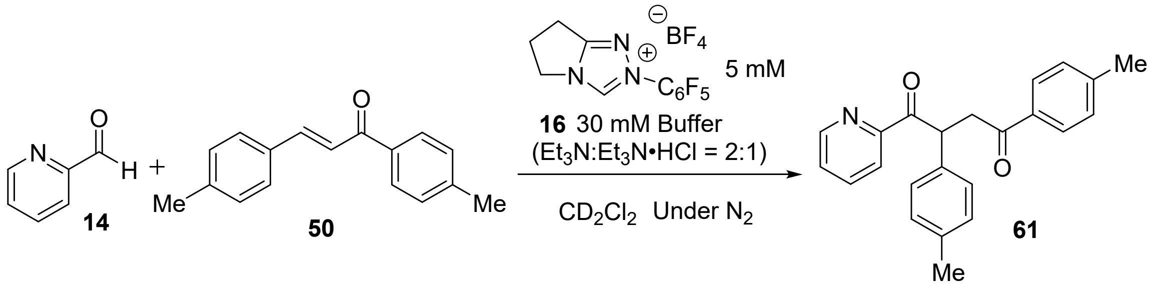

The reaction of pyridine-2-carboxaldehyde **14** and chalcone **50** catalysed by C_6F_5 triazolium salt **16** in triethylamine buffer was monitored using ^{1}H NMR spectra, with representative NMR spectra over the course of the experiment given in Figure S37.

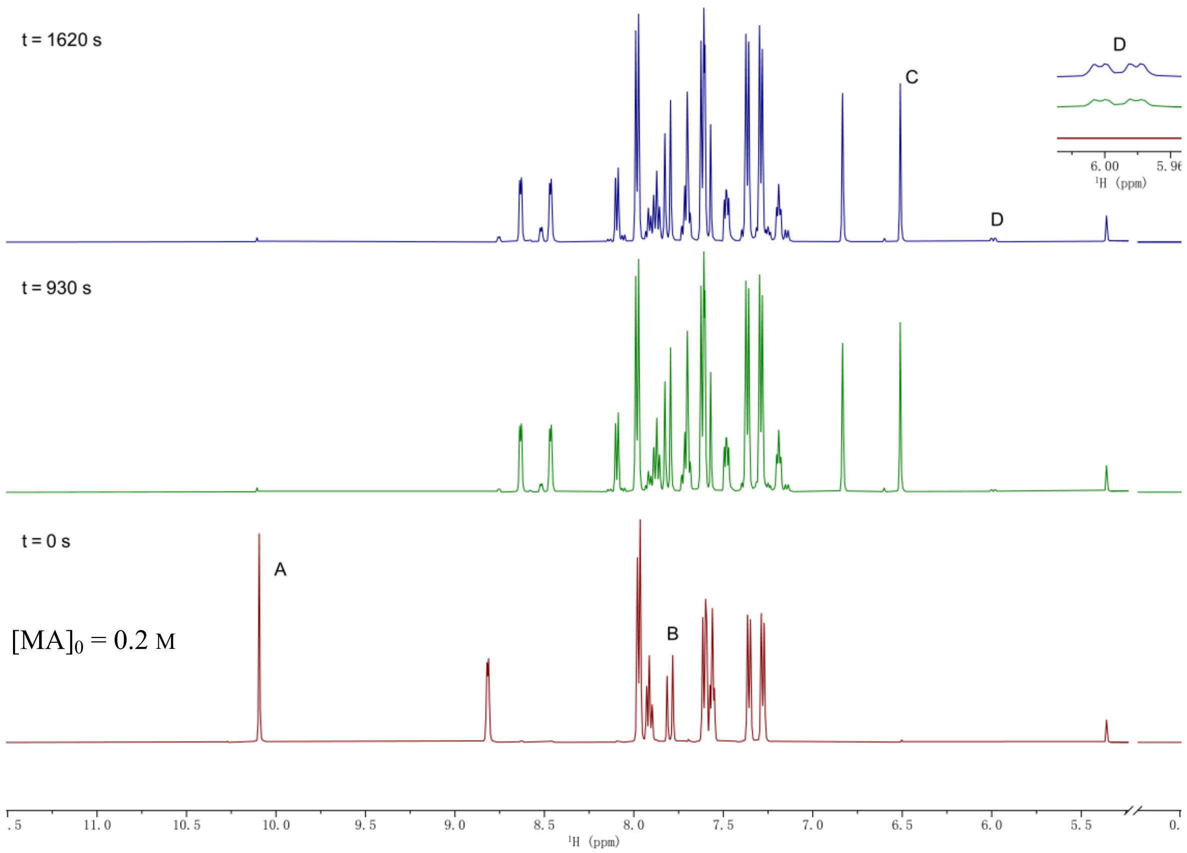
t = 1620 s
t = 930 s
t = 0 s
[MA]$_0$ = 0.2 M
A
B
C
D
11.0 10.5 10.0 9.5 9.0 8.5 8.0 7.5 7.0 6.5 6.0 5.5
^{1}H (ppm)

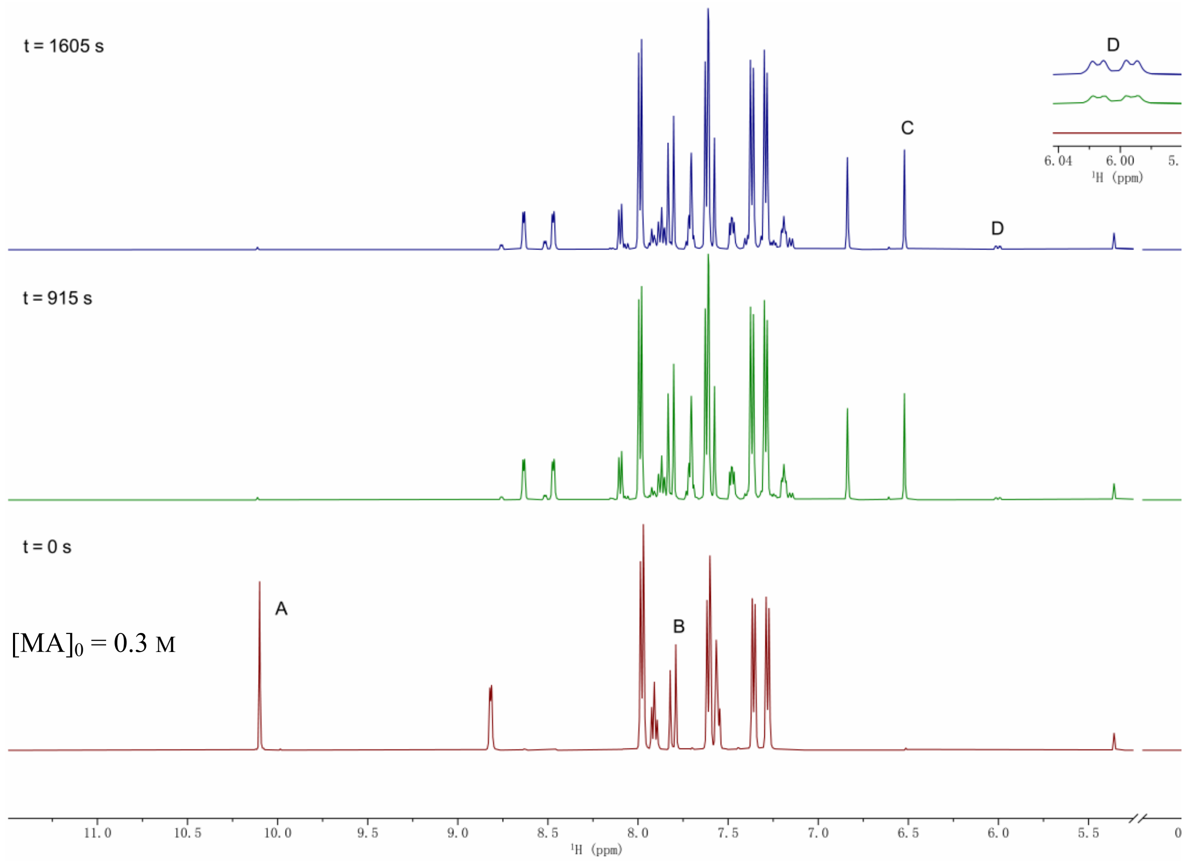
t = 1605 s
t = 915 s
t = 0 s
[MA]$_0$ = 0.3 M
A
B
C
D
6.04 6.00
11.0 10.5 10.0 9.5 9.0 8.5 8.0 7.5 7.0 6.5 6.0 5.5 0
^{1}H (ppm)

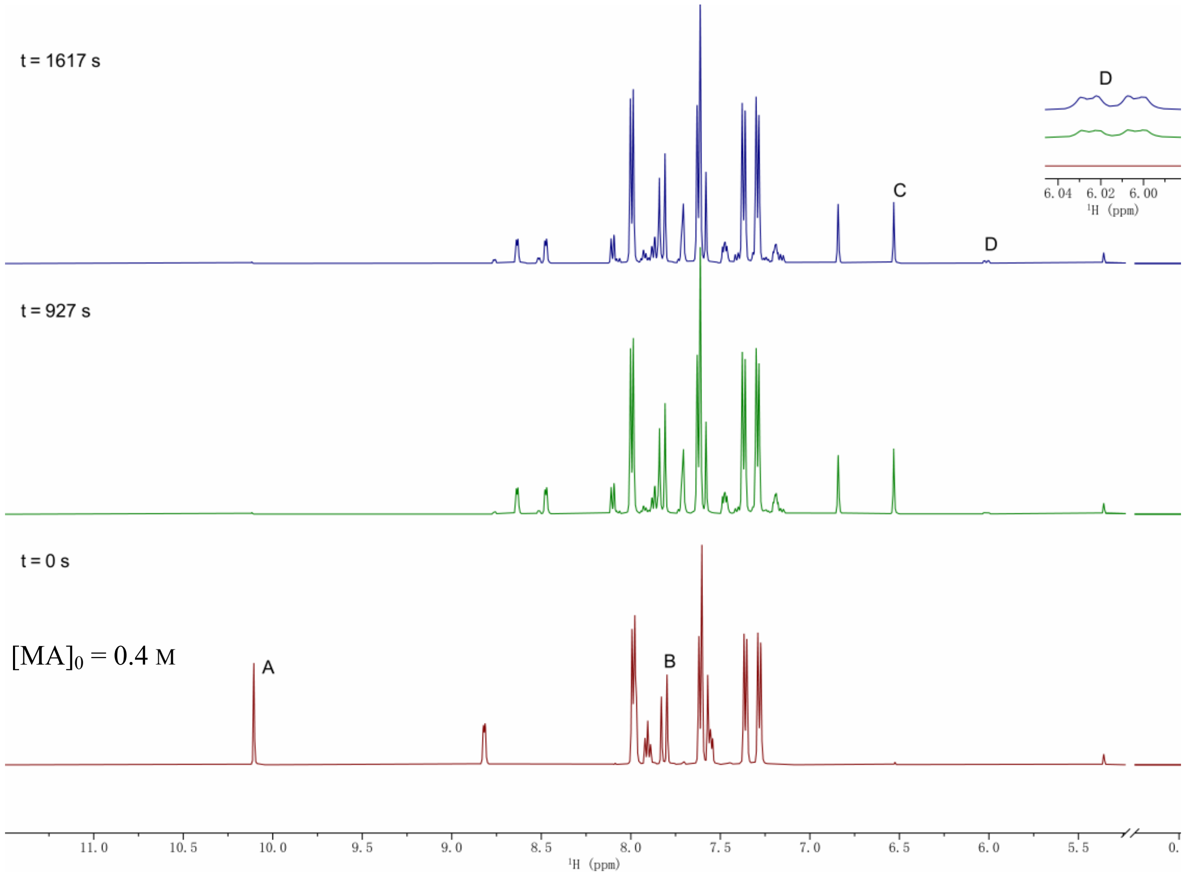

Figure S37. Representative ^{1}H NMR spectra (500 MHz) for reaction of pyridine-2-carboxaldehyde **14** (0.3 M) and chalcone **50** (0.2 M, 0.3 M and 0.4 M) catalysed by *N*-C_6F_5 NHC precursor **16** (5 mM) in NEt_3:NEt_3·HCl (2:1, 0.03 M) in CD_2Cl_2 at 25 °C. A = Aldehyde C*H*O, B = Chalcone C*H*, C = Benzoin C*H*, D = Stetter product C*H*.

Table S17. Reaction data and initial rates of Stetter product formation from (E)-1,3-di-p-tolylprop-2-en-1-one **50**, catalyzed by C_6F_5 triazolium precatalyst **16** (5 mM) in 0.03 M triethylamine buffer (Et_3N:Et_3N·HCl, 2:1) in CD_2Cl_2 at 25 °C.

**[Aldehyde]$_0$ / M**	**[MA]$_0$ / M**	**Time / s**	**[Benzoin] / M**	**[Stetter] / M**	**v_{max} / 10^{-6} M s^{-1}**	**k_s' / 10^{-5} s^{-1}**	**Average / 10^{-5} s^{-1}**
		0	0	0			
		378	0.142991	0.00245			
		516	0.144687	0.003262			
		654	0.142705	0.004591			
		792	0.140153	0.005605			
0.3	0.2	930	0.13875	0.006709	6.89	3.45	3.47±0.12
		1068	0.136891	0.00757			
		1206	0.134772	0.008211			
		1344	0.133278	0.008921			
		1482	0.13152	0.009743			
		1620	0.129904	0.010798			
0.3	0.3	0	0	0	10.90	3.63	

		363	0.143943	0.003347			
		501	0.144763	0.005193			
		639	0.14255	0.006732			
		777	0.14046	0.008156			
		915	0.13814	0.009817			
		1053	0.135958	0.011314			
		1191	0.133912	0.012773			
		1329	0.131968	0.014198			
		1467	0.12985	0.015358			
		1605	0.127969	0.01689			
		0	0	0			
		375	0.141266	0.004027			
		513	0.141875	0.006115			
		651	0.139293	0.008277			
		789	0.136651	0.010142			
0.3	0.4	927	0.134344	0.012035	13.30	3.33	
		1065	0.132112	0.013847			
		1203	0.129988	0.014934			
		1341	0.128225	0.017248			
		1479	0.125953	0.018495			
		1617	0.123907	0.020495			

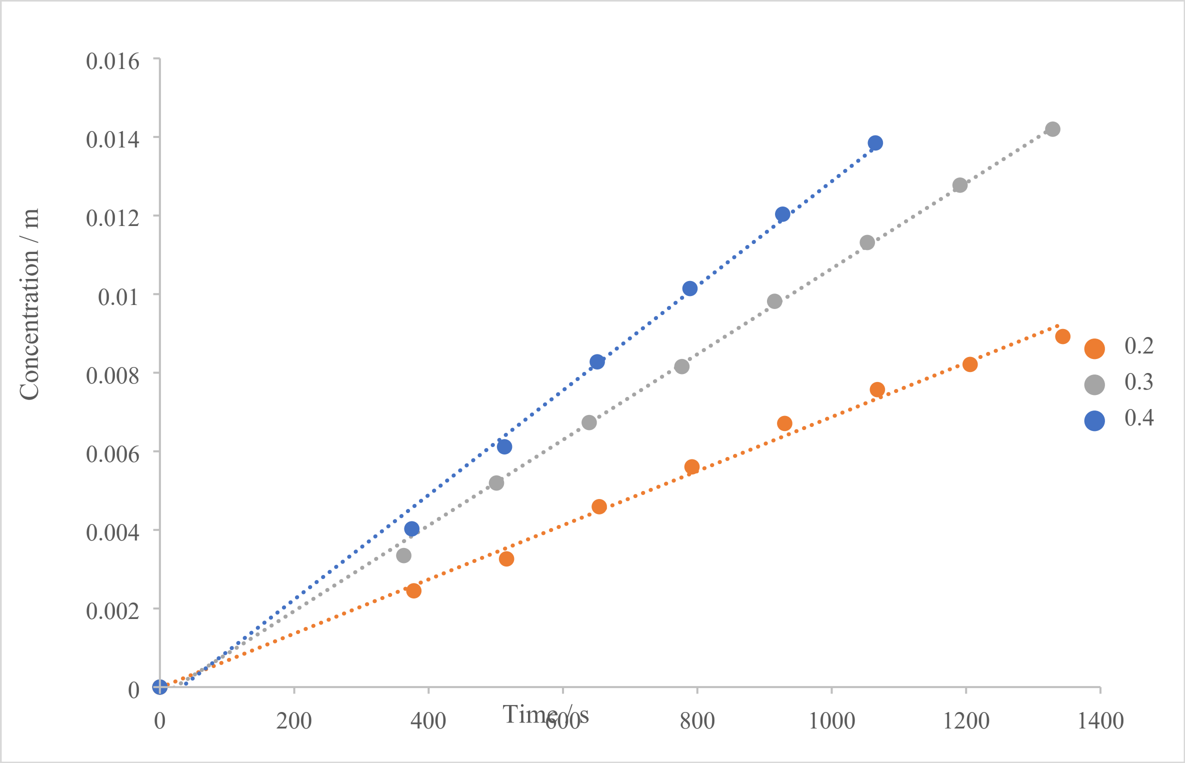

Figure S38. Plots of Stetter product concentration against time for the C_6F_5 triazolium precatalyst **16** (5 mM) catalysed Stetter reaction, at initial (E)-1,3-di-p-tolylprop-2-en-1-one **50** concentrations 0.2 M, 0.3 M, 0.4 M.

Entry 18

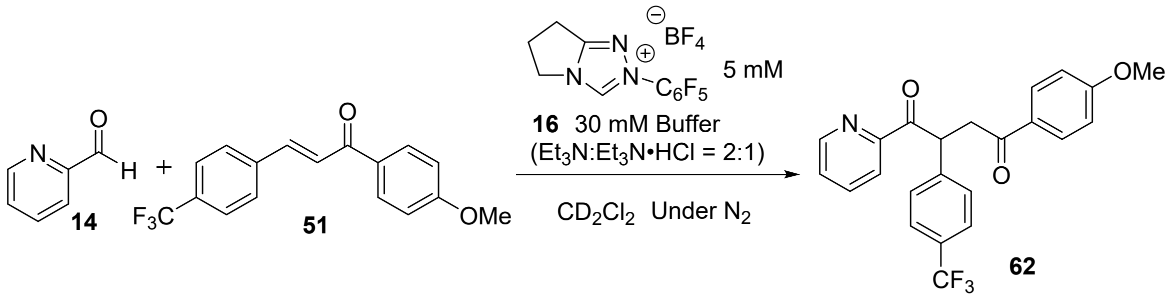

The reaction of pyridine-2-carboxaldehyde **14** and chalcone **51** catalysed by C_6F_5 triazolium salt **16** in triethylamine buffer was monitored using ^{1}H NMR spectra, with representative NMR spectra over the course of the experiment given in Figure S39.

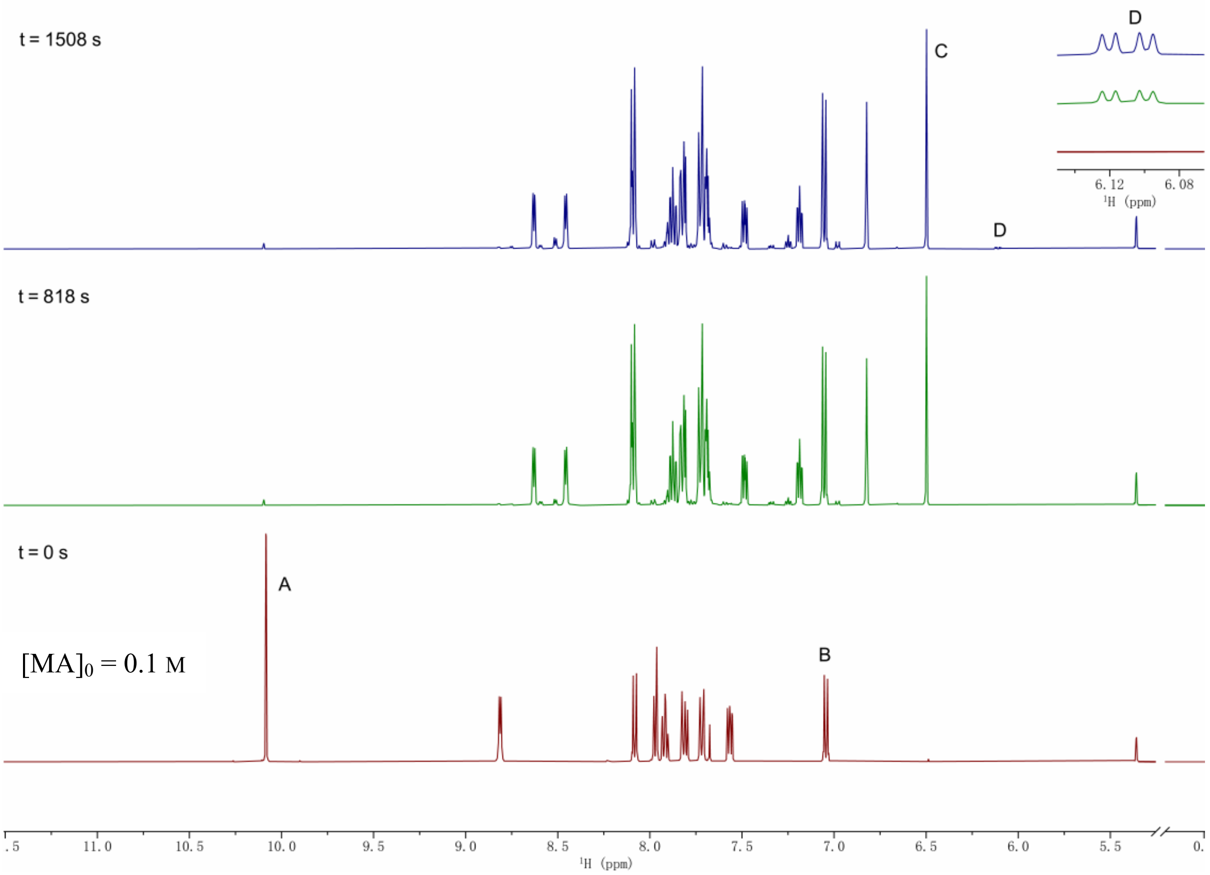

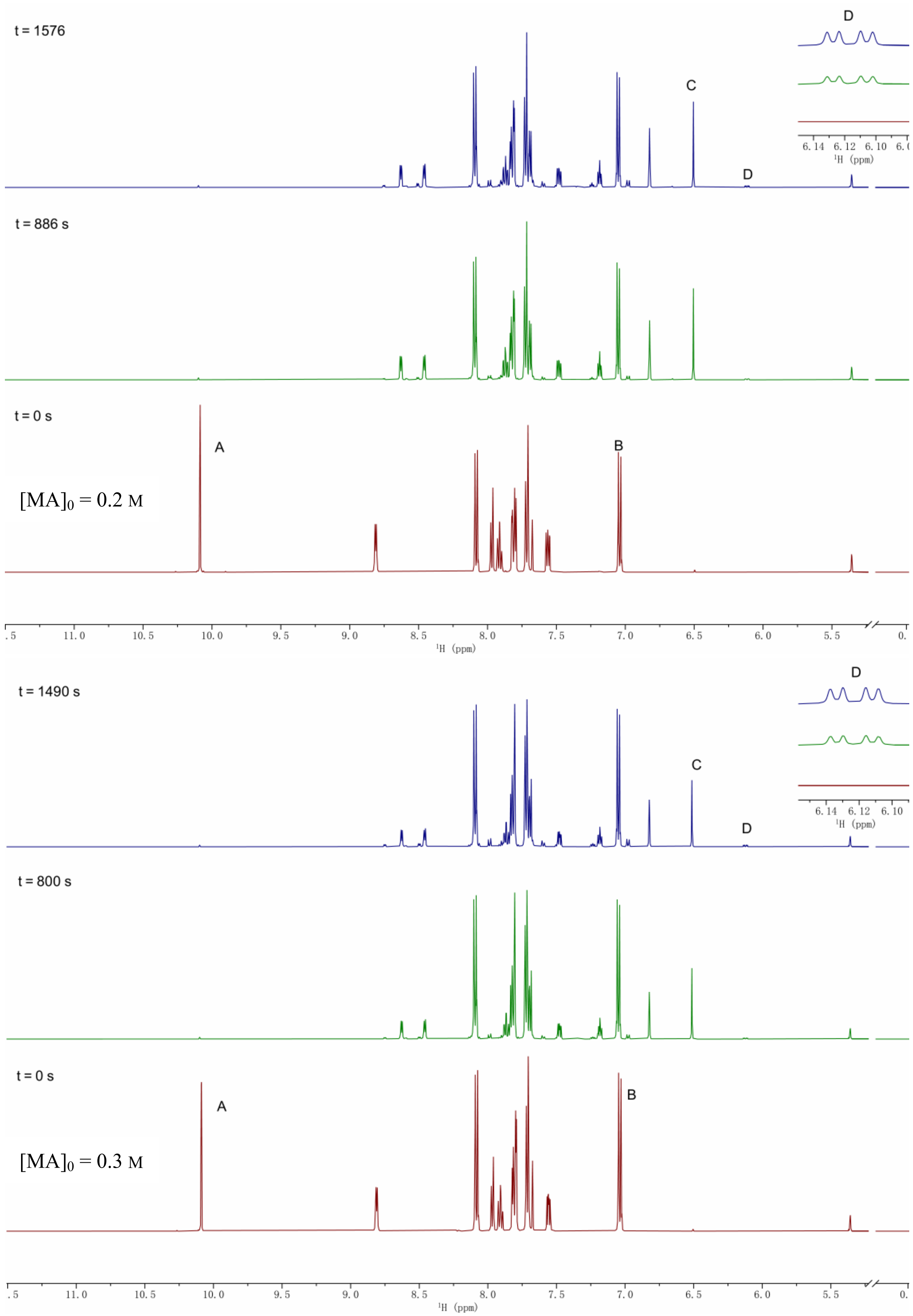

Figure S39. Representative ^{1}H NMR spectra (500 MHz) for reaction of pyridine-2-carboxaldehyde **14** (0.3 M) and chalcone **51** (0.1 M, 0.2 M and 0.3 M) catalysed by *N*-C_6F_5 NHC precursor **16** (5 mM) in NEt_3:$NEt_3 \cdot HCl$

(2:1, 0.03 M) in CD_2Cl_2 at 25 °C. A = Aldehyde C*H*O, B = Chalcone C*H*, C = Benzoin C*H*, D = Stetter product C*H*.

Table S18. Reaction data and initial rates of Stetter product formation from chalcone **51** and pyridine-2-carboxaldehyde **14**, catalysed by C_6F_5 triazolium precatalyst **16** (5 mM) in 0.03 M triethylamine buffer (Et_3N:Et_3N·HCl, 2:1) in CD_2Cl_2 at 25 °C.

**[Aldehyde]$_0$ / M**	**[MA]$_0$ / M**	**Time / s**	**[Benzoin] / M**	**[Stetter] / M**	**v_{max} / 10^{-5} M s^{-1}**	**k'_s / 10^{-5} s^{-1}**	**Average / 10^{-5} s^{-1}**
0.3	0.1	0	0	0	0.326	3.26	3.50±0.18
		266	0.13817	0.00039			
		404	0.138031	0.000978			
		542	0.136409	0.001479			
		680	0.135048	0.001913			
		818	0.134059	0.002489			
		956	0.133089	0.002967			
		1094	0.131293	0.003333			
		1232	0.130255	0.003769			
		1370	0.129053	0.004313			
		1508	0.127705	0.004653			
0.3	0.2	0	0	0	0.740	3.70	
		334	0.141447	0.001653			
		472	0.141543	0.002409			
		610	0.139409	0.003713			
		748	0.137816	0.005063			
		886	0.135694	0.006041			
		1024	0.13391	0.00711			
		1162	0.131945	0.008121			
		1300	0.130597	0.009187			
		1438	0.129255	0.010349			
		1576	0.127822	0.011429			
0.3	0.3	0	0	0	1.06	3.53	
		248	0.134782	0.002384			
		386	0.135387	0.004159			
		524	0.133511	0.005697			
		662	0.131866	0.007152			
		800	0.129911	0.008648			
		938	0.128263	0.009998			
		1076	0.126591	0.011272			
		1214	0.124971	0.01274			
		1352	0.123181	0.013856			
		1490	0.121781	0.015347			

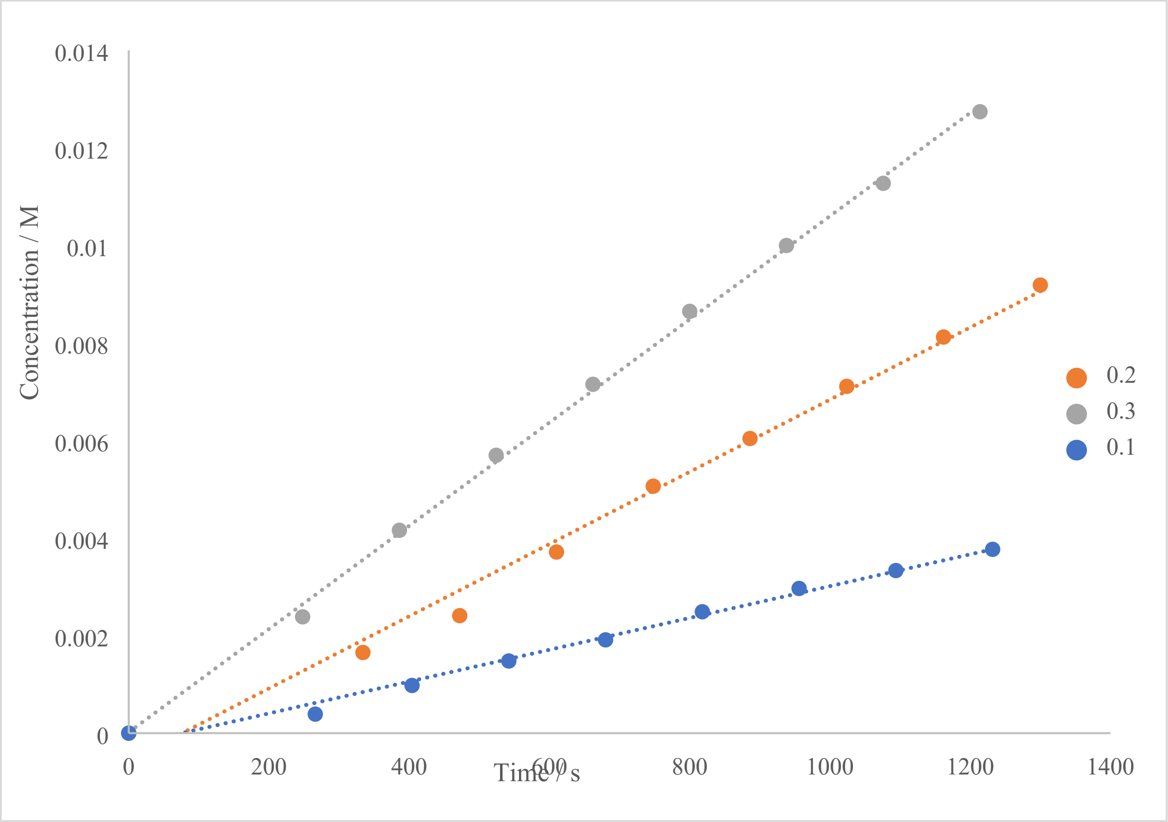

Figure S40. Plots of Stetter product concentration against time for the C_6F_5 triazolium precatalyst **16** (5 mM) catalysed Stetter reaction, at initial chalcone **51** concentrations 0.1 M, 0.2 M, 0.3 M.

Entry 19

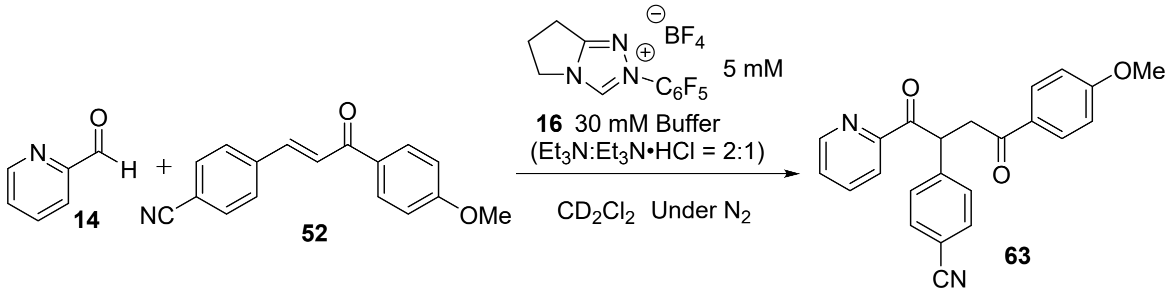

The reaction of pyridine-2-carboxaldehyde **14** and chalcone **52** catalysed by C_6F_5 triazolium salt **16** in triethylamine buffer was monitored using ^{1}H NMR spectra, with representative NMR spectra over the course of the experiment given in Figure S41.

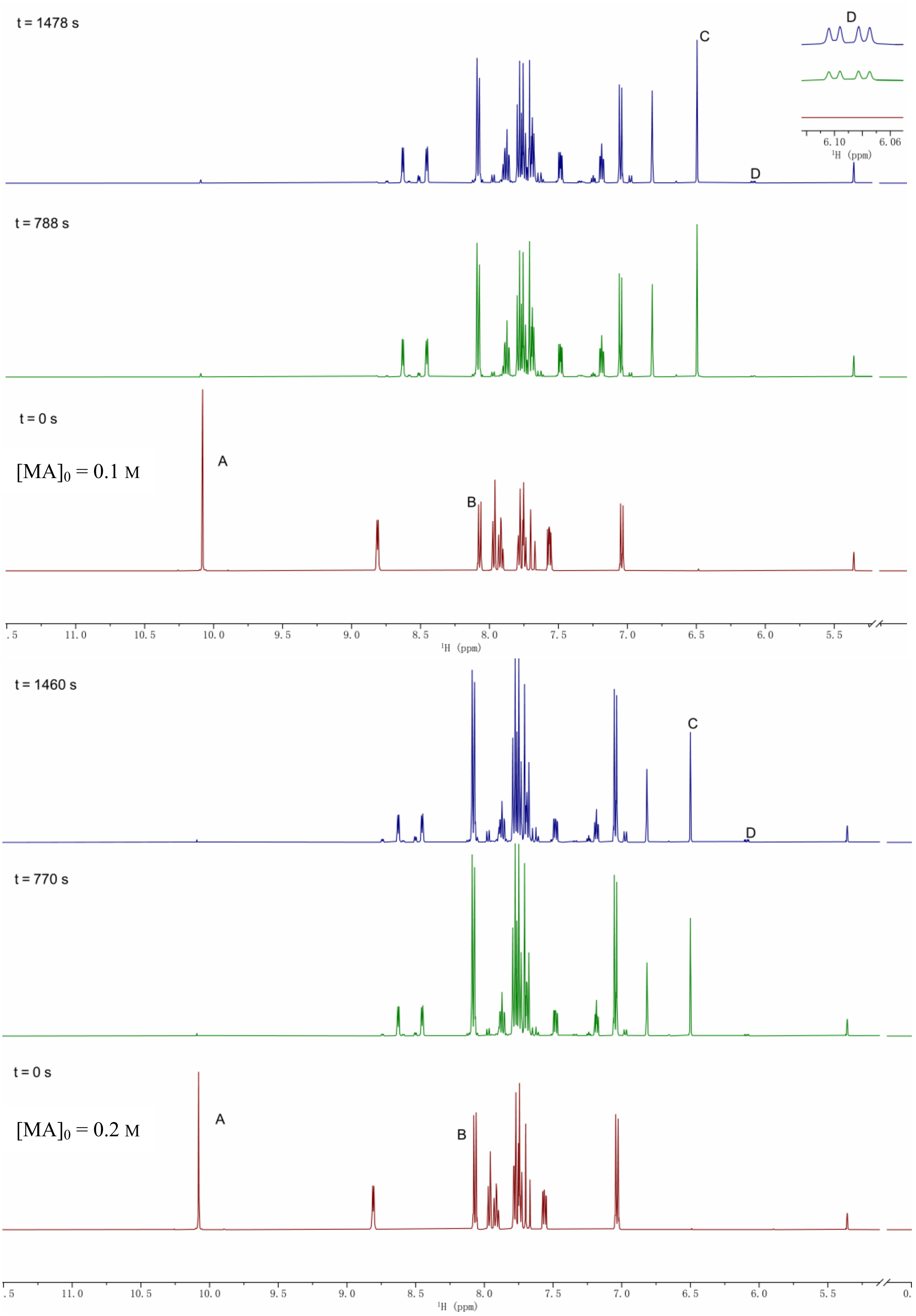
t = 1478 s
C
D
D
6.10
6.06
¹H (ppm)
t = 788 s
t = 0 s
[MA]₀ = 0.1 M
A
B
.5
11.0
10.5
10.0
9.5
9.0
8.5
8.0
7.5
7.0
6.5
6.0
5.5
¹H (ppm)
t = 1460 s
C
D
t = 770 s
t = 0 s
[MA]₀ = 0.2 M
A
B
.5
11.0
10.5
10.0
9.5
9.0
8.5
8.0
7.5
7.0
6.5
6.0
5.5
0.
¹H (ppm)

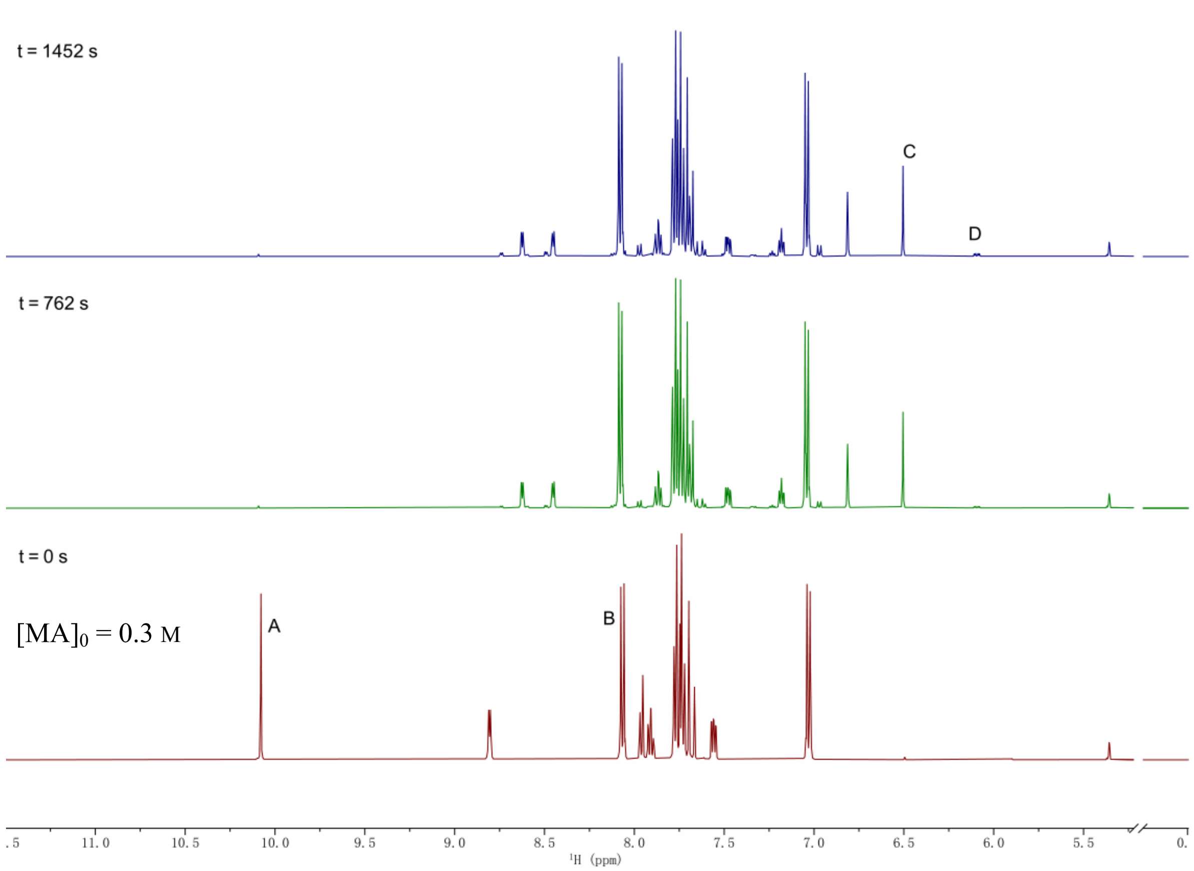

Figure S41. Representative ^{1}H NMR spectra (500 MHz) for reaction of pyridine-2-carboxaldehyde **14** (0.3 M) and chalcone **52** (0.1 M, 0.2 M and 0.3 M) catalysed by *N*-C_6F_5 NHC precursor **16** (5 mM) in NEt_3:NEt_3·HCl (2:1, 0.03 M) in CD_2Cl_2 at 25 °C. A = Aldehyde C*H*O, B = Chalcone Ph*H*, C = Benzoin C*H*, D = Stetter product C*H*.

Table S19. Reaction data and initial rates of Stetter product formation from chalcone **52** and pyridine-2-carboxaldehyde **14**, catalysed by C_6F_5 triazolium precatalyst **16** (5 mM) in 0.03 M triethylamine buffer (Et_3N:Et_3N·HCl, 2:1) in CD_2Cl_2 at 25 °C.

**[Aldehyde]$_0$ / M**	**[MA]$_0$ / M**	**Time / s**	**[Benzoin] / M**	**[Stetter] / M**	v_{max} / **10^{-5} M s^{-1}**	k_s' / **10^{-5} s^{-1}**	**Average / 10^{-5} s^{-1}**
		0	0	0			
		236	0.144563	0.000532			
		374	0.144535	0.001421			
		512	0.142686	0.002287			
		650	0.14102	0.002983			
0.3	0.1	788	0.139344	0.00376	0.502	5.02	
		926	0.137809	0.004313			4.53±0.45
		1064	0.136329	0.004989			
		1202	0.134724	0.005787			
		1340	0.13354	0.00622			
		1478	0.132017	0.007078			
0.3	0.2	0	0	0	0.927	4.64	

		218	0.14138	0.001393		
		356	0.141648	0.002686		
		494	0.139597	0.004047		
		632	0.138346	0.005335		
		770	0.136836	0.006672		
		908	0.135064	0.008129		
		1046	0.133429	0.009322		
		1184	0.131472	0.010669		
		1322	0.129886	0.012181		
		1460	0.128215	0.013596		
		0	0	0		
		210	0.141656	0.001036		
		348	0.141498	0.002837		
		486	0.139066	0.004468		
		624	0.137015	0.005802		
0.3	0.3	762	0.135105	0.008186	1.18	3.93
		900	0.133349	0.009823		
		1038	0.131555	0.011185		
		1176	0.129795	0.013441		
		1314	0.127975	0.0145		
		1452	0.126333	0.016821		

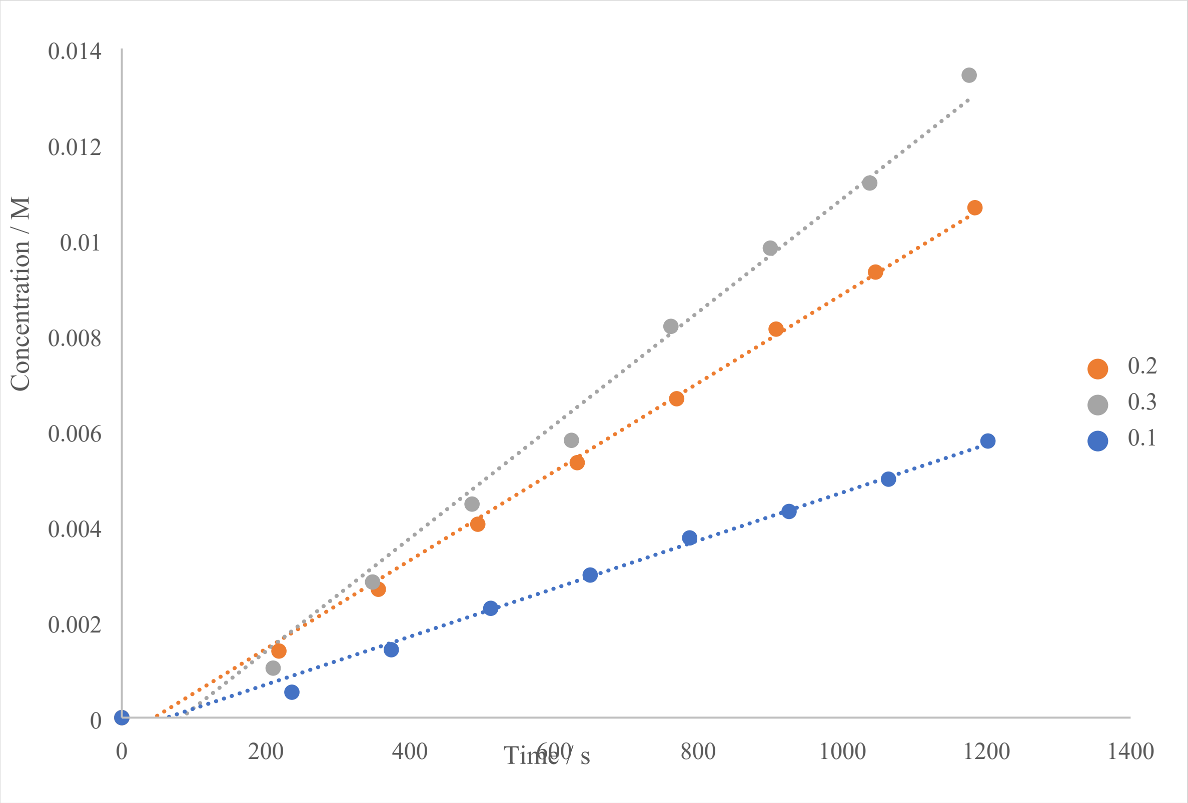

Figure S42. Plots of Stetter product concentration against time for the C_6F_5 triazolium precatalyst **16** (5 mM) catalysed Stetter reaction, at initial chalcone **52** concentrations 0.1 M, 0.2 M, 0.3 M.

Entry 20

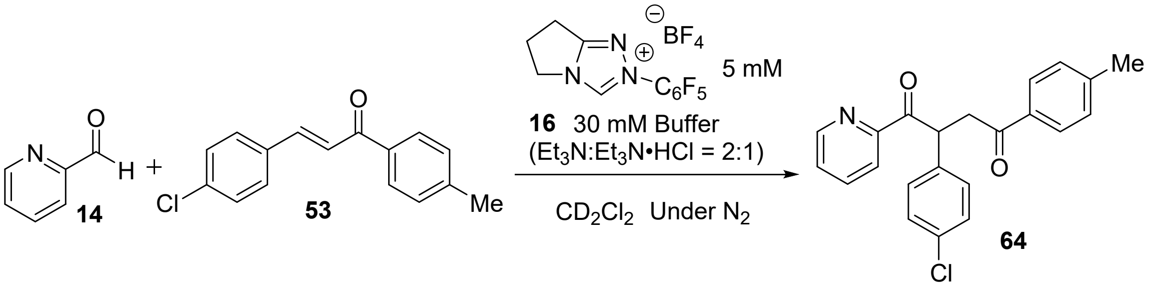

The reaction of pyridine-2-carboxaldehyde **14** and chalcone **53** catalysed by C_6F_5 triazolium salt **16** in triethylamine buffer was monitored using ^{1}H NMR spectra, with representative NMR spectra over the course of the experiment given in Figure S43.

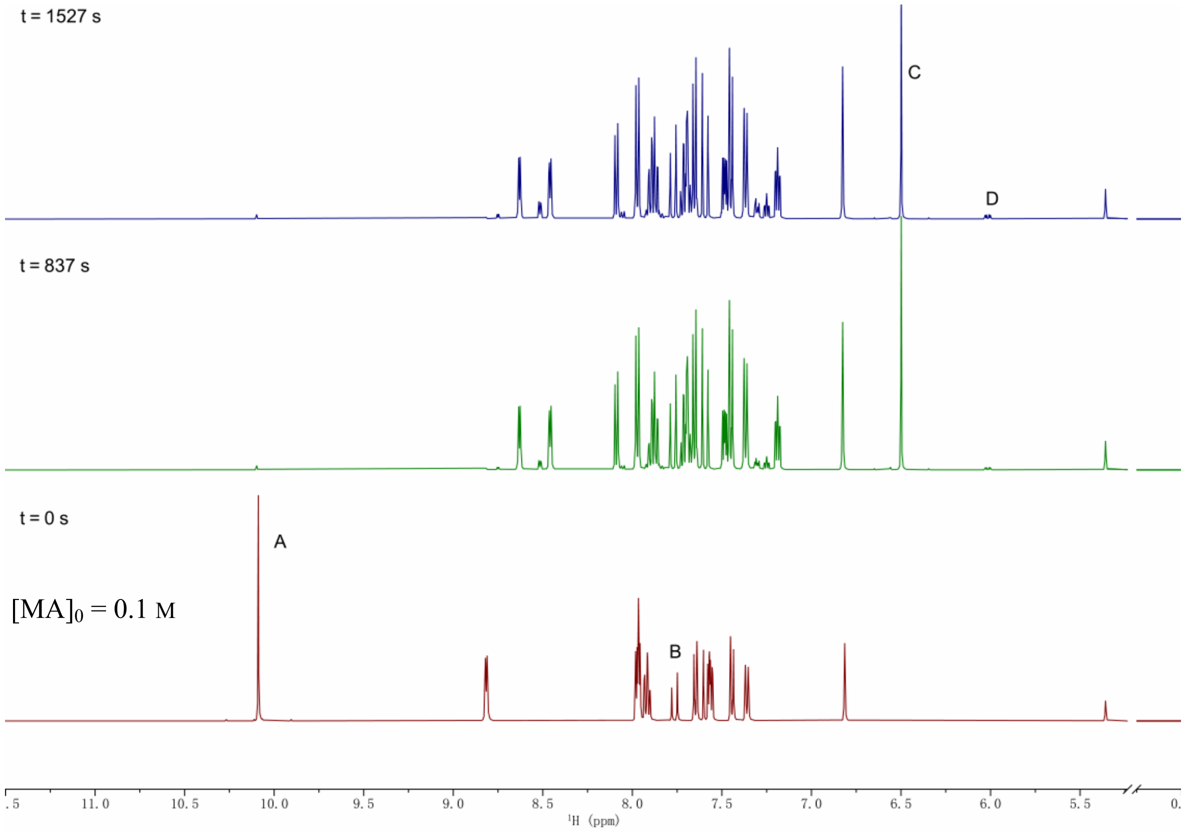

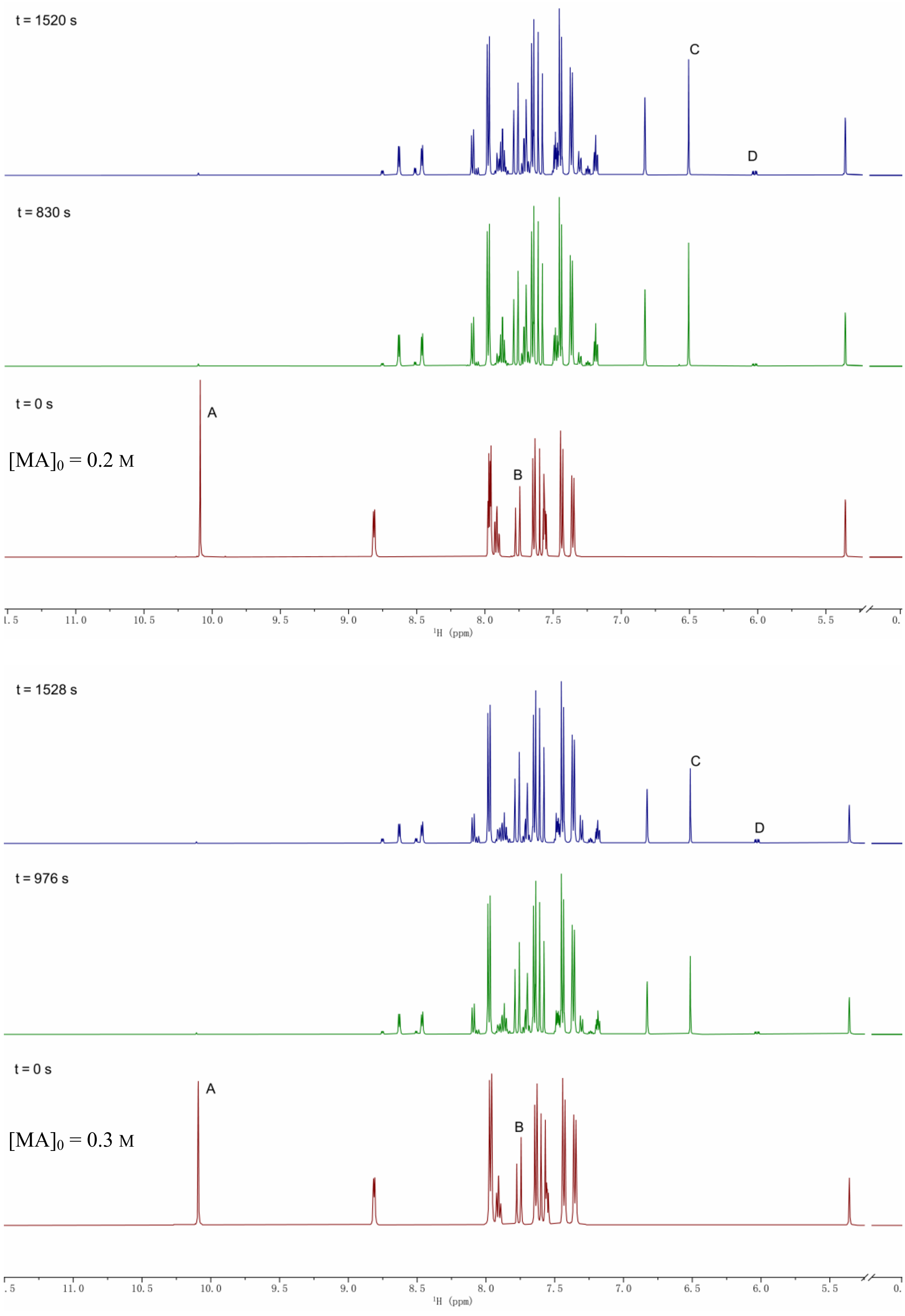

Figure S43. Representative ^{1}H NMR spectra (500 MHz) for reaction of pyridine-2-carboxaldehyde **14** (0.3 M) and chalcone **53** (0.1 M, 0.2 M and 0.3 M) catalysed by *N*-C_6F_5 NHC precursor **16** (5 mM) in NEt_3:NEt_3·HCl

(2:1, 0.03 M) in CD_2Cl_2 at 25 °C. A = Aldehyde C*H*O, B = Chalcone C*H*, C = Benzoin C*H*, D = Stetter product C*H*.

Table S20. Reaction data and initial rates of Stetter product formation from (E)-3-(4-chlorophenyl)-1-(p-tolyl)prop-2-en-1-one **53**, catalyzed by C_6F_5 triazolium precatalyst **16** (5 mM) in 0.03 M triethylamine buffer (Et_3N:Et_3N·HCl, 2:1) in CD_2Cl_2 at 25 °C.

**[Aldehyde]$_0$ / M**	**[MA]$_0$ / M**	**Time / s**	**[Benzoin] / M**	**[Stetter] / M**	**v_{max} / 10^{-5} M s^{-1}**	**k_s' / 10^{-5} s^{-1}**	**Average / 10^{-5} s^{-1}**
0.3	0.1	0	0	0	0.617	6.17	6.03±0.18
		285	0.149461	0.00164			
		423	0.150739	0.002407			
		561	0.148488	0.003393			
		699	0.146008	0.004233			
		837	0.143609	0.005116			
		975	0.141829	0.005886			
		1113	0.139810	0.006828			
		1251	0.137752	0.007634			
		1389	0.135849	0.008455			
		1527	0.133922	0.009232			
0.3	0.2	0	0	0	1.23	6.15	
		278	0.14183	0.002971			
		416	0.142566	0.004288			
		554	0.140081	0.006085			
		692	0.137533	0.008138			
		830	0.134958	0.009772			
		968	0.132409	0.011642			
		1106	0.130144	0.013293			
		1244	0.127911	0.01503			
		1382	0.12564	0.016562			
		1520	0.123256	0.018153			
0.3	0.3	0	0	0	1.73	5.77	
		424	0.137069	0.007144			
		562	0.135098	0.010041			
		700	0.132494	0.012481			
		838	0.129691	0.015022			
		976	0.12708	0.017197			
		1114	0.124592	0.019468			
		1252	0.122196	0.021628			
		1390	0.119768	0.0239			
		1528	0.117468	0.026071			

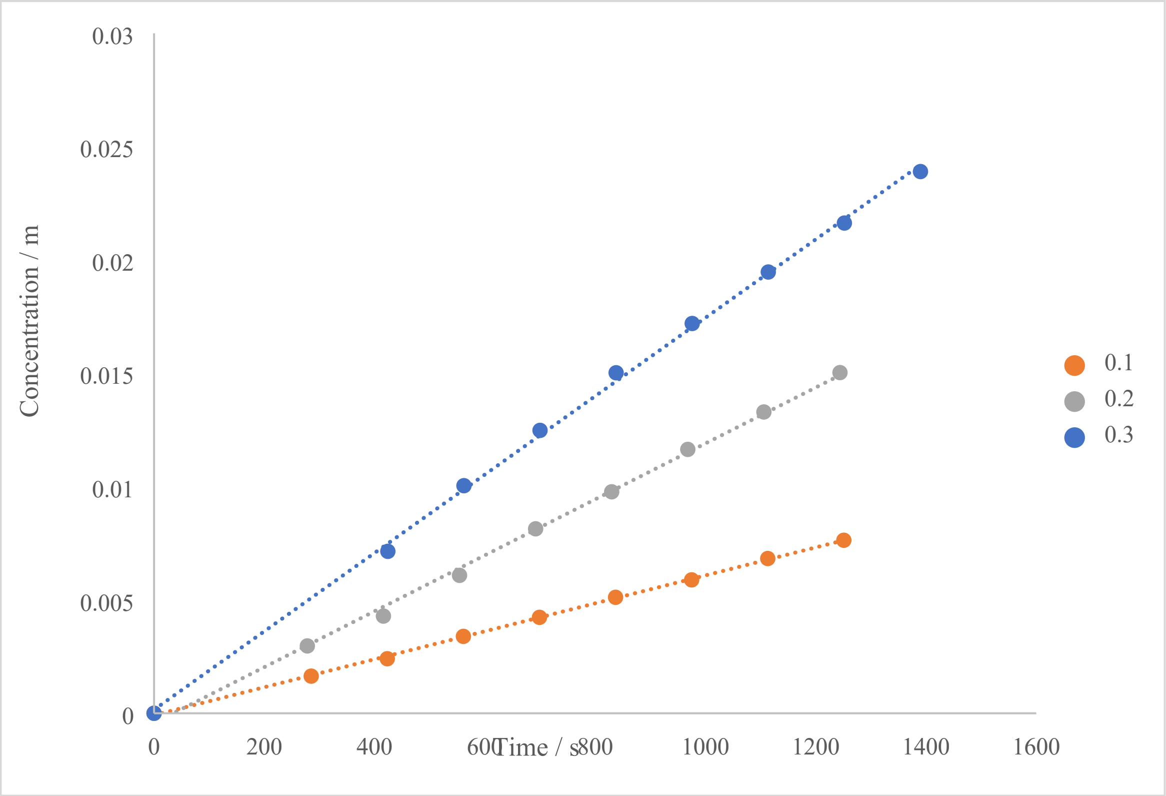

Figure S44. Plots of Stetter product concentration against time for the C_6F_5 triazolium precatalyst **16** (5 mM) catalysed Stetter reaction, at initial (E)-3-(4-chlorophenyl)-1-(p-tolyl)prop-2-en-1-one **53** concentrations 0.1 M, 0.2 M, 0.3 M.

Entry 21

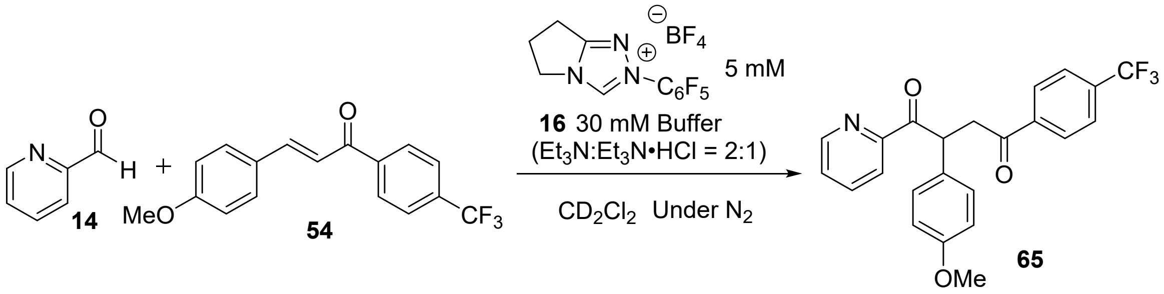

The reaction of pyridine-2-carboxaldehyde **14** and chalcone **54** catalysed by C_6F_5 triazolium salt **16** in triethylamine buffer was monitored using ^{1}H NMR spectra, with representative NMR spectra over the course of the experiment given in Figure S45.

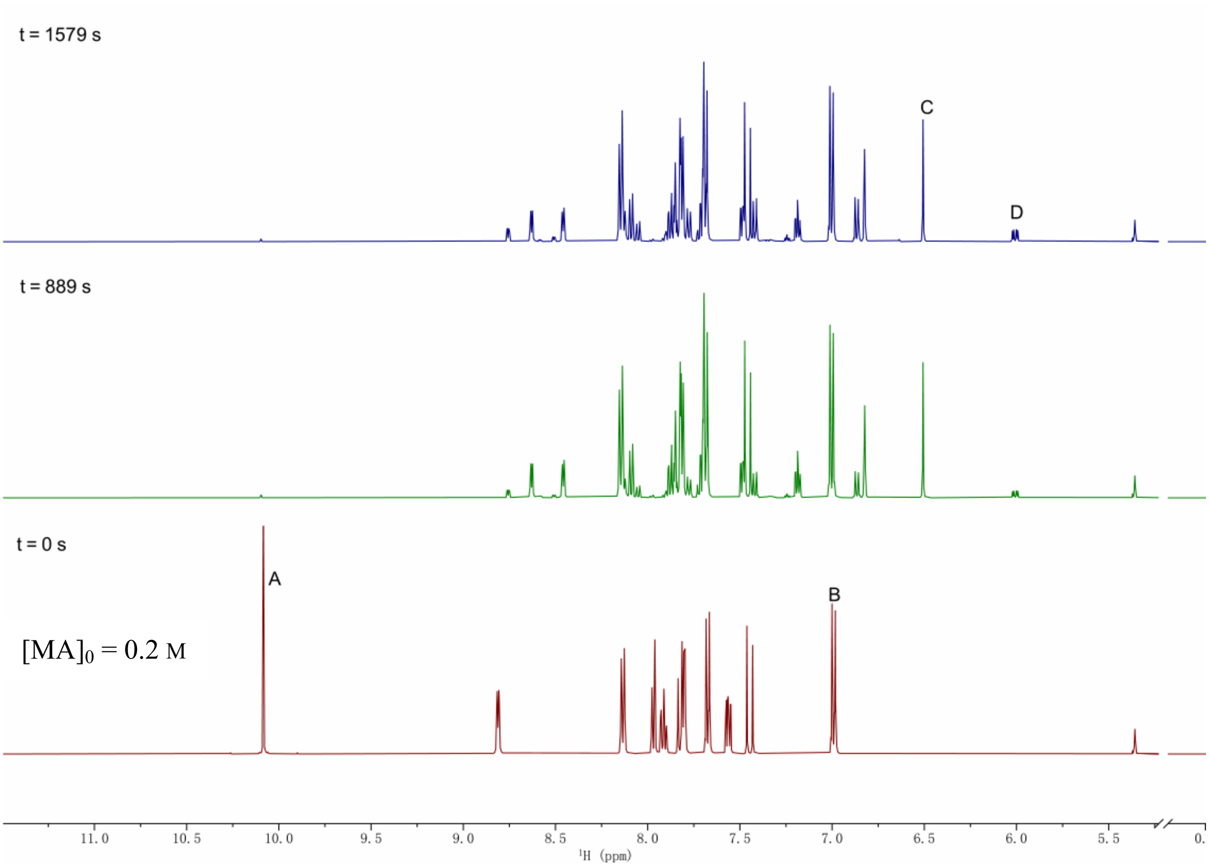
t = 1579 s
C
D
t = 889 s
t = 0 s
A
B
[MA]0 = 0.2 M
11.0
10.5
10.0
9.5
9.0
8.5
8.0
7.5
7.0
6.5
6.0
5.5
0.
1H (ppm)

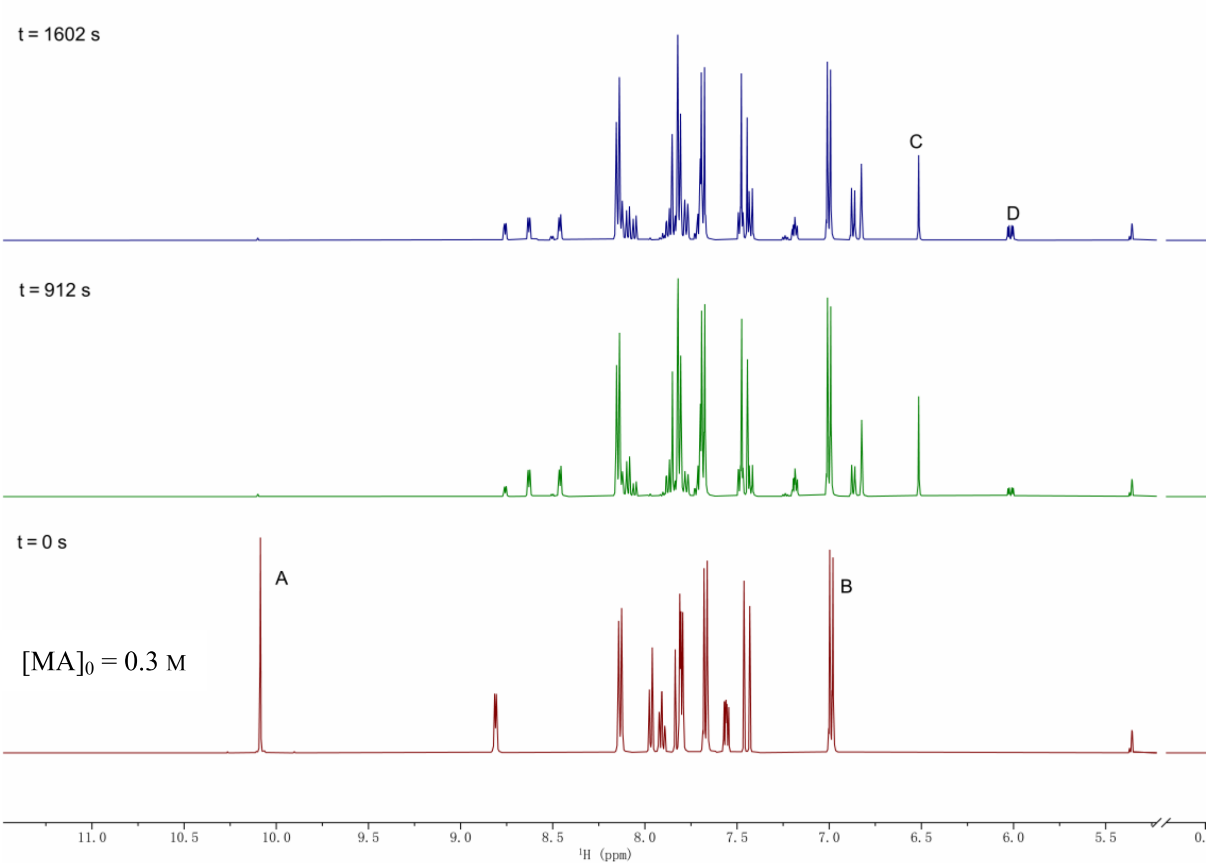
t = 1602 s
C
D
t = 912 s
t = 0 s
A
B
[MA]0 = 0.3 M
11.0
10.5
10.0
9.5
9.0
8.5
8.0
7.5
7.0
6.5
6.0
5.5
0.
1H (ppm)

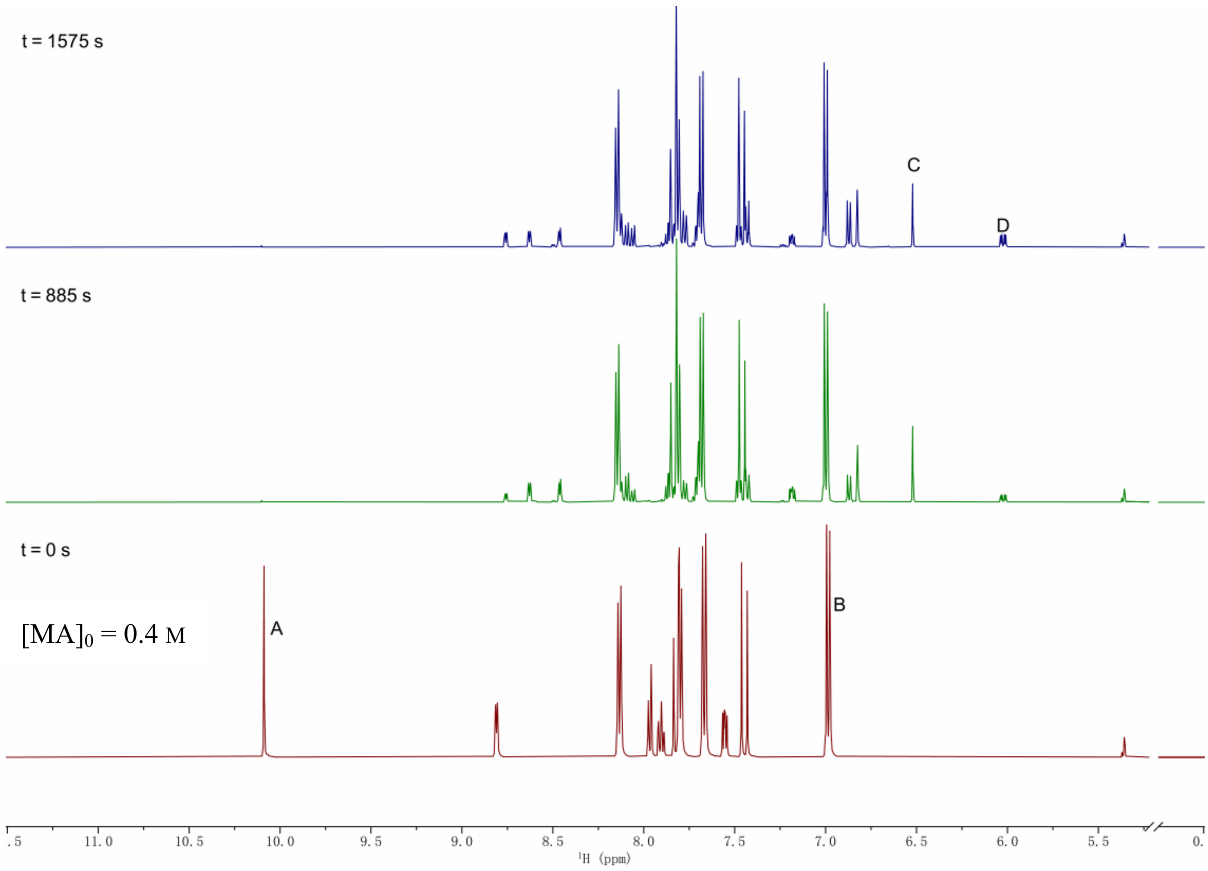

Figure S45. Representative ^{1}H NMR spectra (500 MHz) for reaction of pyridine-2-carboxaldehyde **14** (0.3 M) and chalcone **54** (0.2 M, 0.3 M and 0.4 M) catalysed by *N*-C_6F_5 NHC precursor **16** (5 mM) in NEt_3:NEt_3·HCl (2:1, 0.03 M) in CD_2Cl_2 at 25 °C. A = Aldehyde C*H*O, B = Chalcone Ph*H*, C = Benzoin C*H*, D = Stetter product C*H*.

Table S21. Reaction data and initial rates of Stetter product formation from chalcone **54** and pyridine-2-carboxaldehyde **14**, catalysed by C_6F_5 triazolium precatalyst **16** (5 mM) in 0.03 M triethylamine buffer (Et_3N:Et_3N·HCl, 2:1) in CD_2Cl_2 at 25 °C.

**[Aldehyde]$_0$ / M**	**[MA]$_0$ / M**	**Time / s**	**[Benzoin] / M**	**[Stetter] / M**	v_{max} / **10^{-5} M s^{-1}**	k_s' / **10^{-5} s^{-1}**	**Average / 10^{-5} s^{-1}**
0.3	0.2	0	0	0	3.06	15.30	14.68±0.85
		337	0.135058	0.008198			
		475	0.133514	0.013613			
		613	0.130066	0.018496			
		751	0.126654	0.02276			
		889	0.123817	0.026797			
		1027	0.121063	0.030872			
		1165	0.11861	0.034937			
		1303	0.115965	0.038808			
		1441	0.113423	0.042505			
		1579	0.110807	0.046003			
0.3	0.3	0	0	0	4.58	15.27	

		360	0.130968	0.013939		
		498	0.128549	0.022056		
		636	0.123673	0.028763		
		774	0.119384	0.035296		
		912	0.115509	0.041603		
		1050	0.112064	0.047867		
		1188	0.108388	0.053689		
		1326	0.104755	0.059422		
		1464	0.101467	0.064976		
		1602	0.098287	0.070454		
0.3	0.4	0	0	0	5.39	13.48
		333	0.129506	0.014514		
		471	0.125361	0.024179		
		609	0.120081	0.032768		
		747	0.115476	0.040407		
		885	0.111133	0.047754		
		1023	0.10704	0.054724		
		1161	0.10309	0.06149		
		1299	0.09908	0.06811		
		1437	0.095366	0.074691		
		1575	0.09127	0.080757		

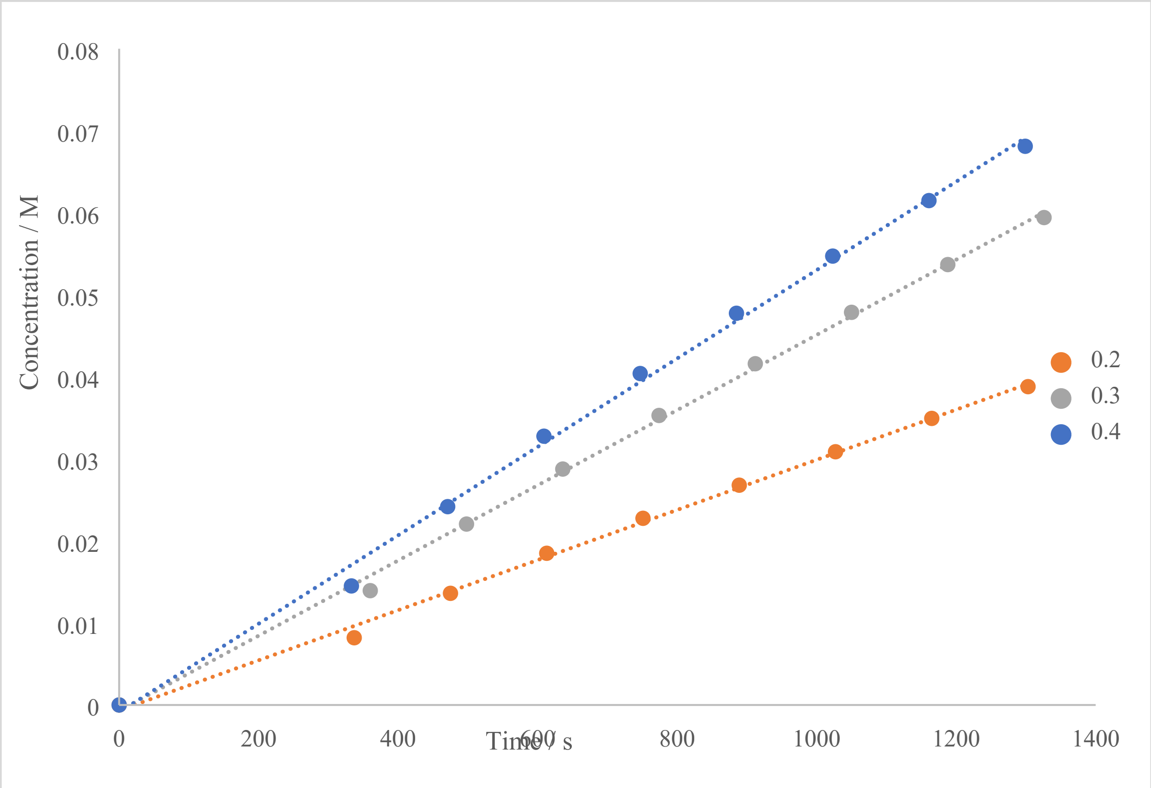

Figure S46. Plots of Stetter product concentration against time for the C_6F_5 triazolium precatalyst **16** (5 mM) catalysed Stetter reaction, at initial chalcone **54** concentrations 0.2 M, 0.3 M, 0.4 M.

Entry 22

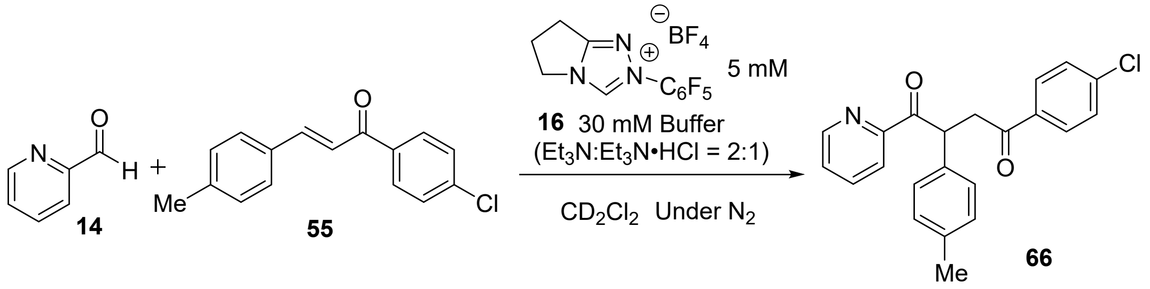

The reaction of pyridine-2-carboxaldehyde **14** and chalcone **55** catalysed by C_6F_5 triazolium salt **16** in triethylamine buffer was monitored using ^{1}H NMR spectra, with representative NMR spectra over the course of the experiment given in Figure S47.

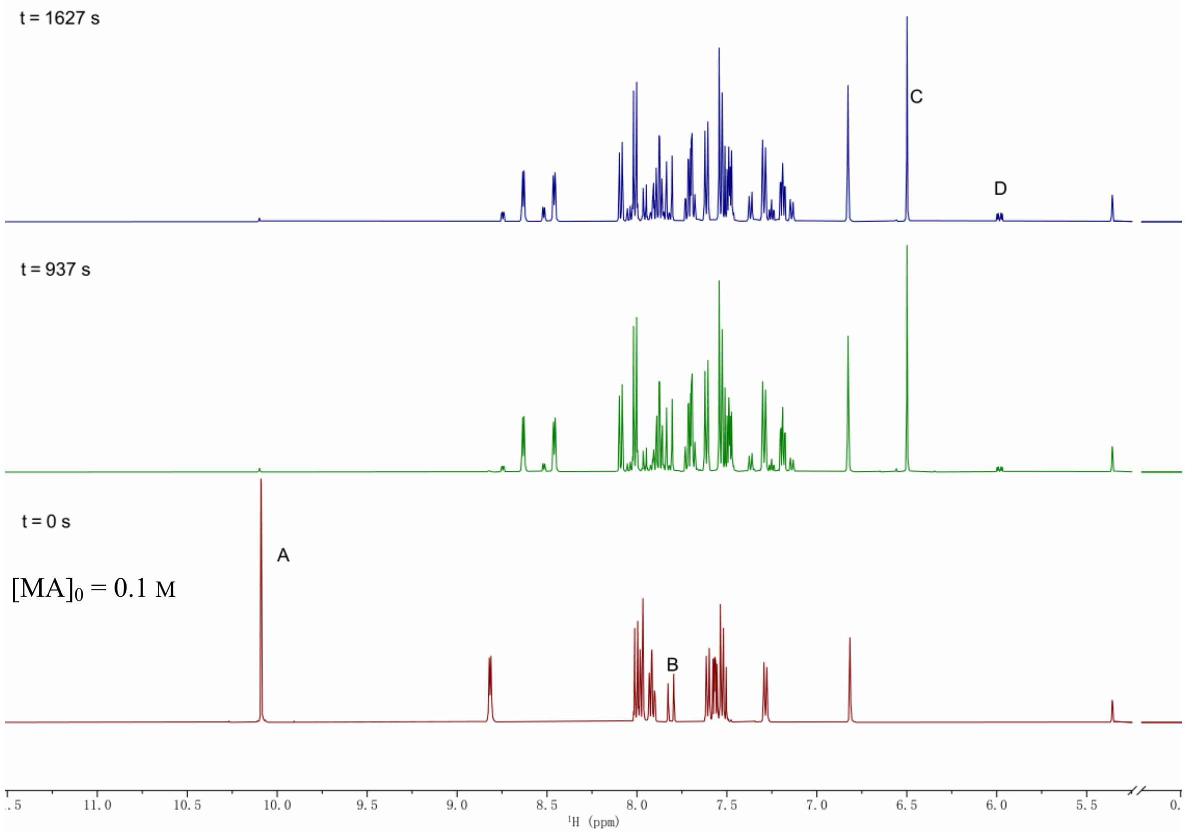

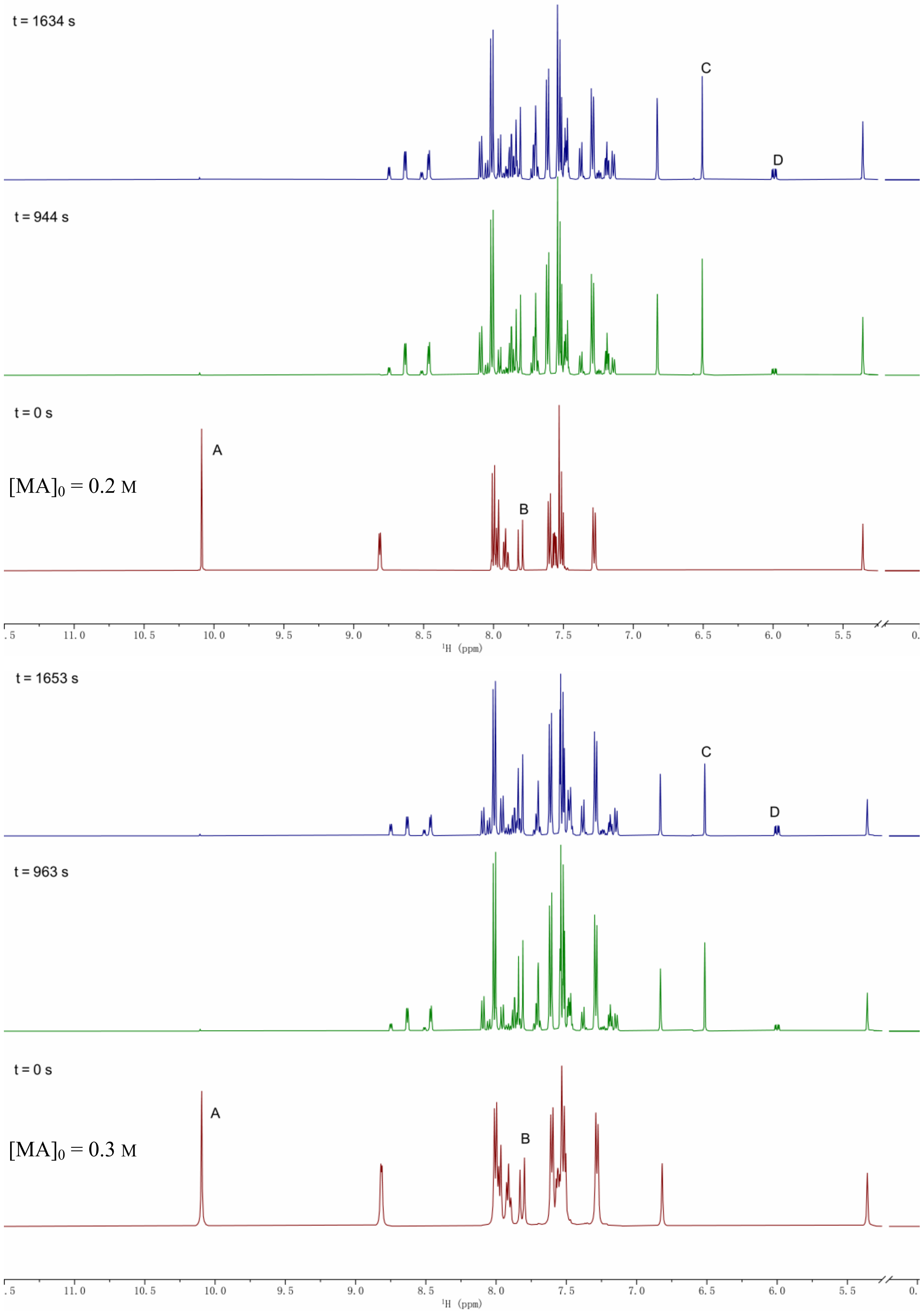

Figure S47. Representative ^{1}H NMR spectra (500 MHz) for reaction of pyridine-2-carboxaldehyde **14** (0.3 M) and chalcone **55** (0.1 M, 0.2 M and 0.3 M) catalysed by *N*-C_6F_5 NHC precursor **16** (5 mM) in NEt_3:NEt_3·HCl (2:1, 0.03 M) in CD_2Cl_2 at 25 °C. A = Aldehyde C*H*O, B = Chalcone C*H*, C = Benzoin C*H*, D = Stetter

product C*H*.

Table S22. Reaction data and initial rates of Stetter product formation from (E)-1-(4-chlorophenyl)-3-(p-tolyl)prop-2-en-1-one **55**, catalyzed by C_6F_5 triazolium precatalyst **16** (5 mM) in 0.03 M triethylamine buffer (Et_3N:Et_3N·HCl, 2:1) in CD_2Cl_2 at 25 °C.

[Aldehyde]$_0$ / M	[MA]$_0$ / M	Time / s	[Benzoin] / M	[Stetter] / M	v_{max} / 10^{-5} M s^{-1}	k_s' / 10^{-5} s^{-1}	Average / 10^{-5} s^{-1}
0.3	0.1	0	0	0	1.47	14.7	14.92±0.15
		385	0.143286	0.005041			
		523	0.143513	0.007549			
		661	0.141206	0.009768			
		799	0.138136	0.011925			
		937	0.135642	0.013998			
		1075	0.132629	0.01587			
		1213	0.130692	0.017758			
		1351	0.128485	0.019441			
		1489	0.126043	0.021186			
		1627	0.124159	0.022832			
0.3	0.2	0	0	0	3.01	15.05	
		392	0.138373	0.010022			
		530	0.138392	0.015218			
		668	0.134711	0.01981			
		806	0.131034	0.024229			
		944	0.127598	0.028432			
		1082	0.124397	0.032385			
		1220	0.121191	0.036172			
		1358	0.118204	0.039881			
		1496	0.115175	0.04348			
		1634	0.112351	0.046979			
0.3	0.3	0	0	0	4.50	15.00	
		411	0.134031	0.017595			
		549	0.133019	0.025154			
		687	0.129019	0.031753			
		825	0.124917	0.037995			
		963	0.120977	0.044148			
		1101	0.117331	0.050125			
		1239	0.113223	0.055559			
		1377	0.109859	0.061298			
		1515	0.106337	0.066616			
		1653	0.103009	0.072017			

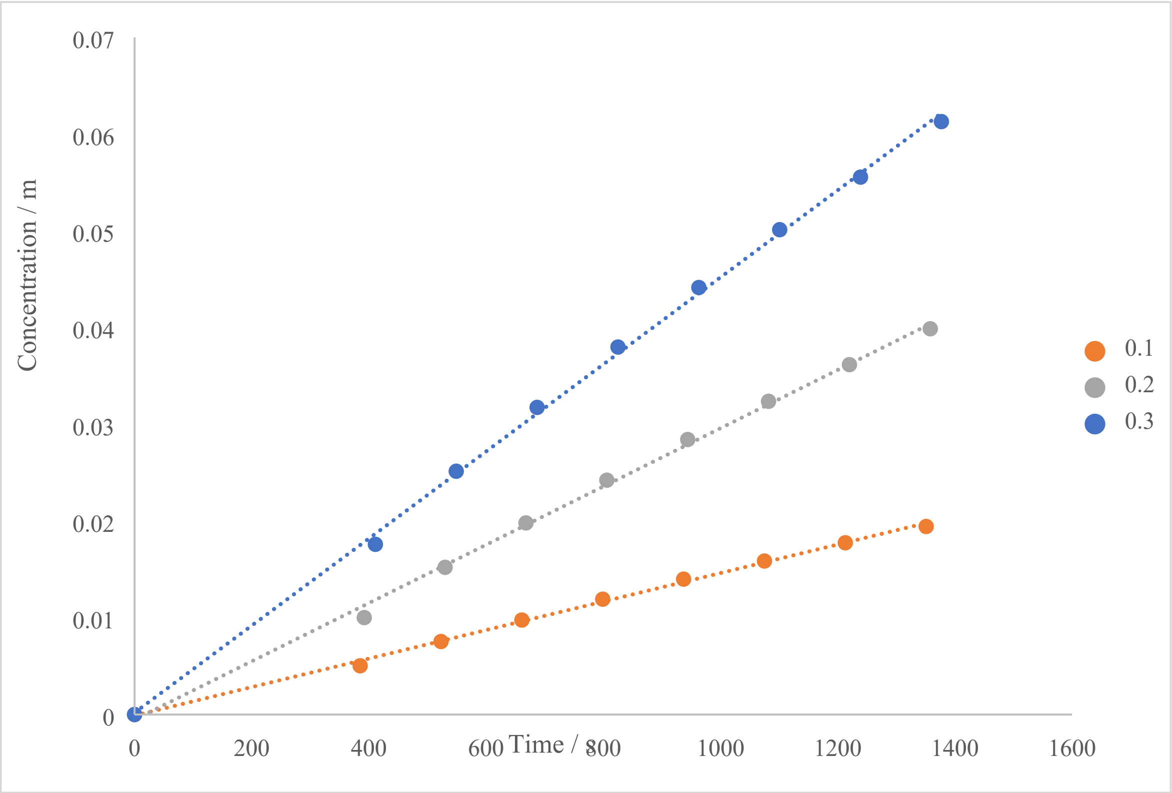

Figure S48. Plots of Stetter product concentration against time for the C_6F_5 triazolium precatalyst **16** (5 mM) catalysed Stetter reaction, at initial (E)-1-(4-chlorophenyl)-3-(p-tolyl)prop-2-en-1-one **55** concentrations 0.1 M, 0.2 M, 0.3 M.

Entry 23

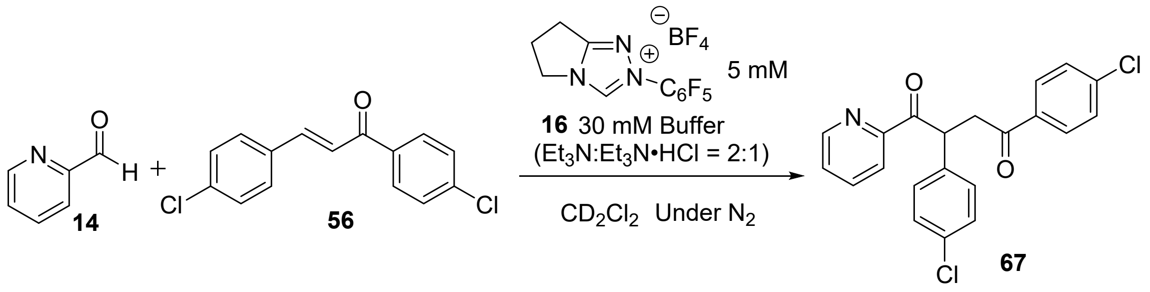

The reaction of pyridine-2-carboxaldehyde **14** and chalcone **56** catalysed by C_6F_5 triazolium salt **16** in triethylamine buffer was monitored using ^{1}H NMR spectra, with representative NMR spectra over the course of the experiment given in Figure S49.

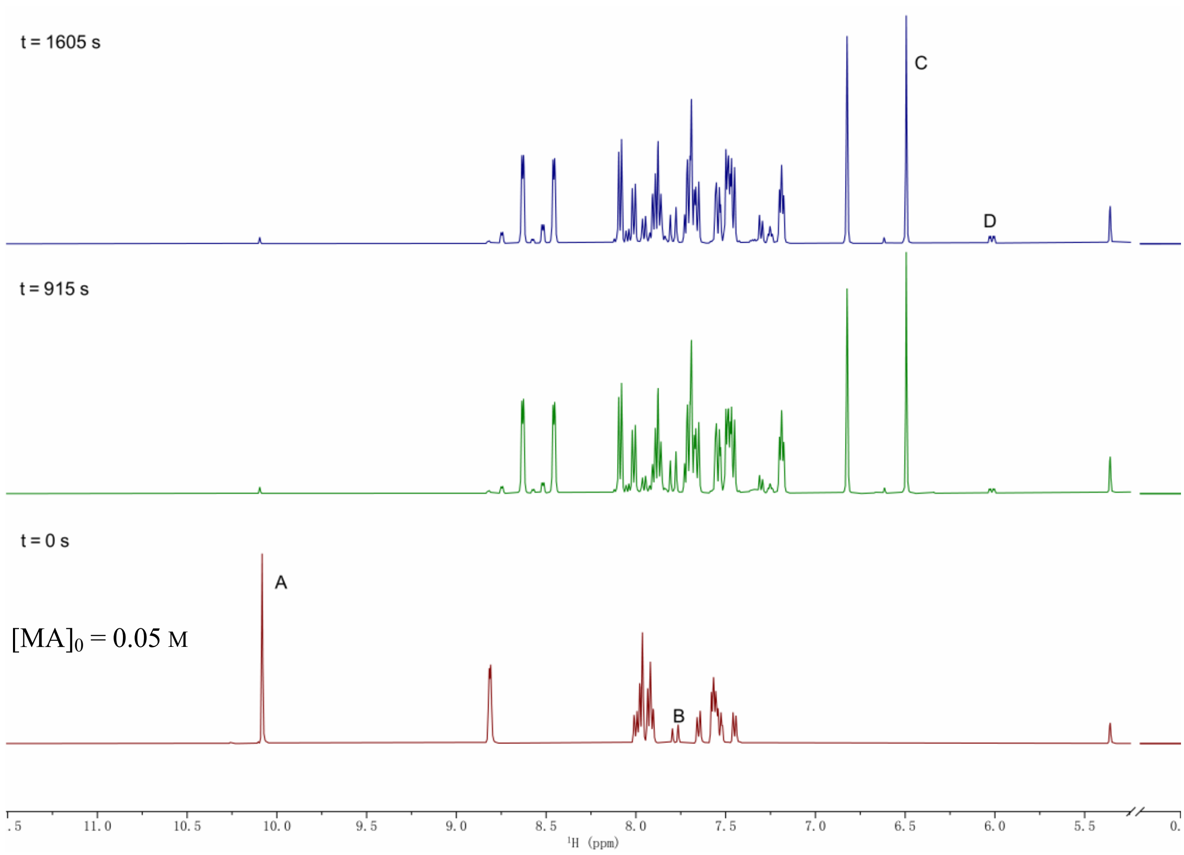
t = 1605 s
C
D
t = 915 s
t = 0 s
A
[MA]$_0$ = 0.05 M
B
.5
11.0
10.5
10.0
9.5
9.0
8.5
8.0
7.5
7.0
6.5
6.0
5.5
0.
^{1}H (ppm)

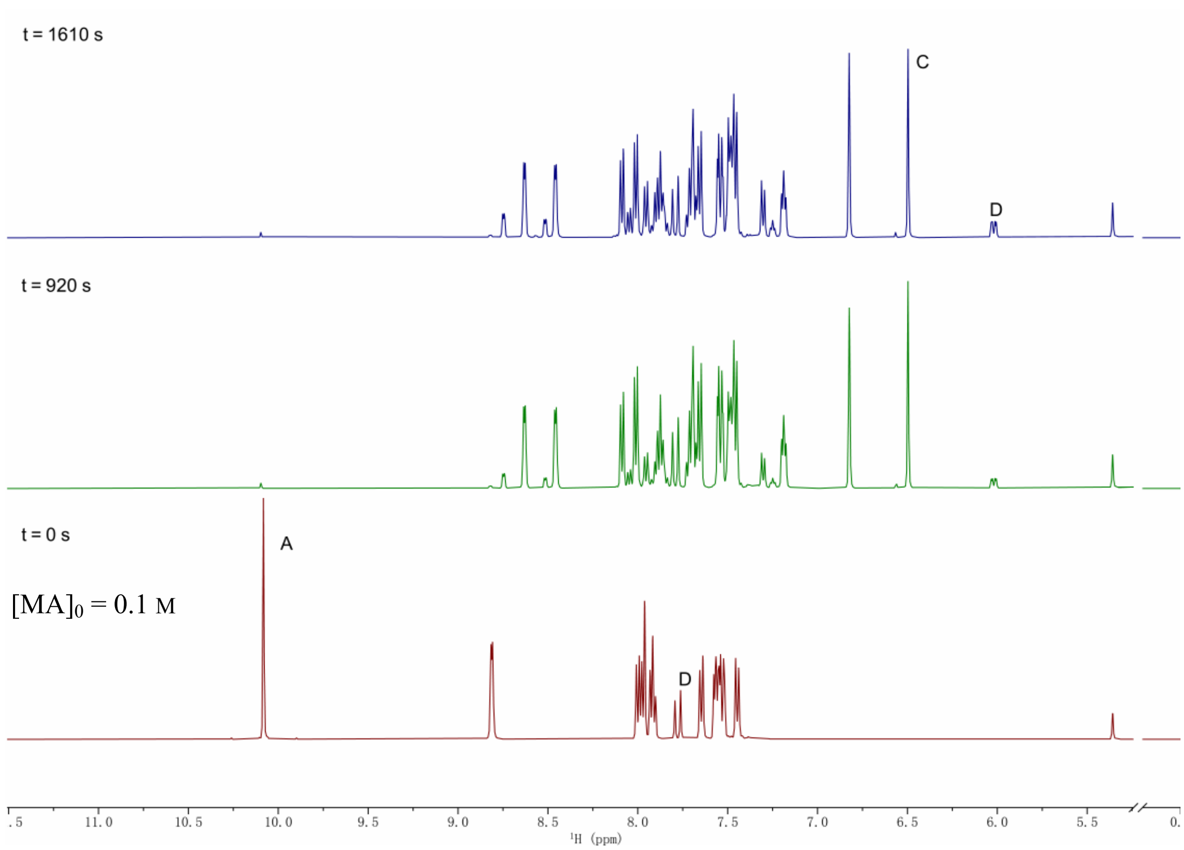
t = 1610 s
C
D
t = 920 s
t = 0 s
A
[MA]$_0$ = 0.1 M
D
.5
11.0
10.5
10.0
9.5
9.0
8.5
8.0
7.5
7.0
6.5
6.0
5.5
0.
^{1}H (ppm)

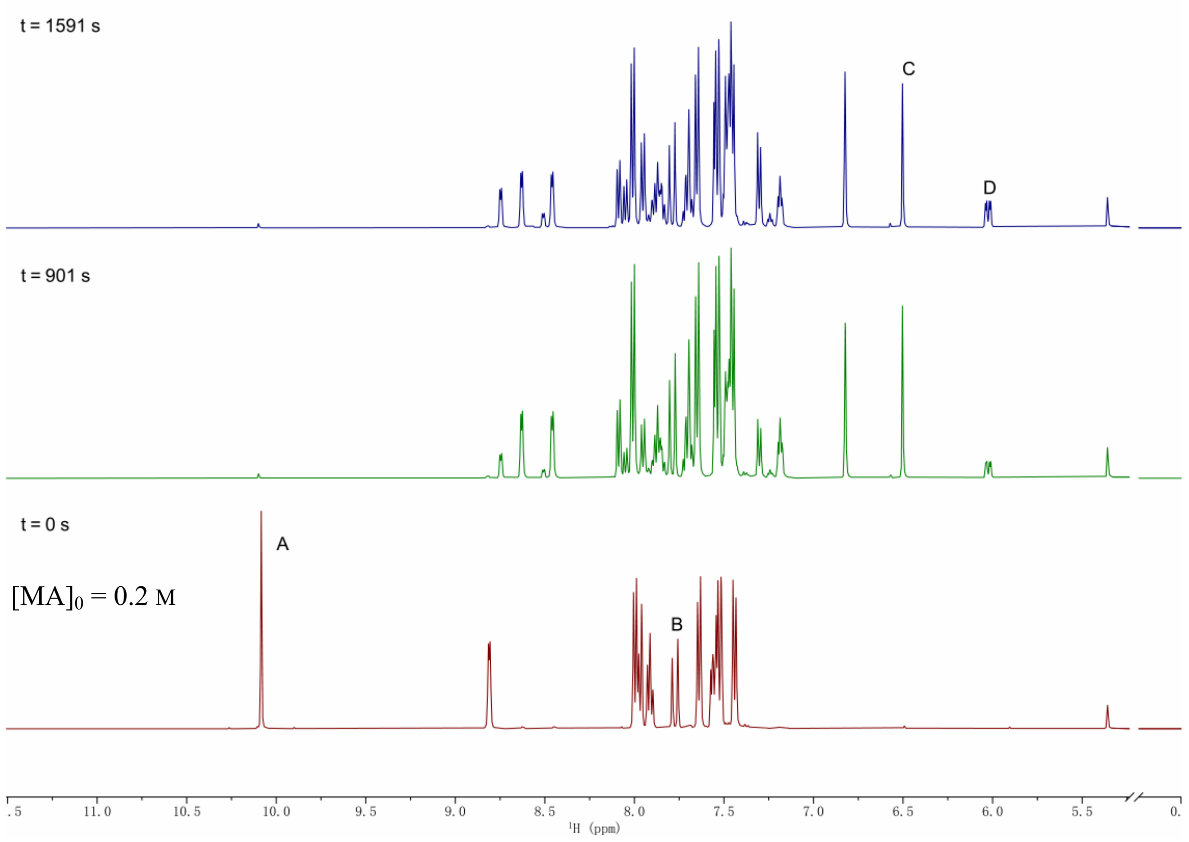

Figure S49. Representative ^{1}H NMR spectra (500 MHz) for reaction of pyridine-2-carboxaldehyde **14** (0.3 M) and chalcone **56** (0.05 M, 0.1 M and 0.2 M) catalysed by *N*-C_6F_5 NHC precursor **16** (5 mM) in NEt_3:NEt_3·HCl (2:1, 0.03 M) in CD_2Cl_2 at 25 °C. A = Aldehyde C*H*O, B = Chalcone C*H*, C = Benzoin C*H*, D = Stetter product C*H*.

Table S23. Reaction data and initial rates of Stetter product formation from (E)-1,3-bis(4-chlorophenyl)prop-2-en-1-one **56**, catalyzed by C_6F_5 triazolium precatalyst **16** (5 mM) in 0.03 M triethylamine buffer (Et_3N:Et_3N·HCl, 2:1) in CD_2Cl_2 at 25 °C.

**[Aldehyde]$_0$ / M**	**[MA]$_0$ / M**	**Time / s**	**[Benzoin] / M**	**[Stetter] / M**	**v_{max} / 10^{-5} M s^{-1}**	**k_s' / 10^{-5} s^{-1}**	**Average / 10^{-5} s^{-1}**
0.3	0.05	0	0	0	1.16	23.20	24.57±0.99
		363	0.148721	0.003786			
		501	0.149608	0.005539			
		639	0.147434	0.007491			
		777	0.145111	0.009005			
		915	0.143031	0.010379			
		1053	0.140626	0.011579			
		1191	0.13871	0.012839			
		1329	0.137128	0.0141			
		1467	0.135141	0.015107			
		1605	0.133315	0.016035			
0.3	0.1	0	0	0	2.55	25.50	

		368	0.148488	0.008563			
		506	0.14846	0.013069			
		644	0.145176	0.01727			
		782	0.141751	0.021011			
		920	0.138467	0.02469			
		1058	0.135312	0.027349			
		1196	0.132346	0.030468			
		1334	0.129462	0.03323			
		1472	0.126772	0.035922			
		1610	0.124403	0.038654			
0.3	0.2	0	0	0	5.00	25.00	
		349	0.147425	0.015768			
		487	0.145185	0.024693			
		625	0.139614	0.032198			
		763	0.134778	0.039536			
		901	0.129872	0.046171			
		1039	0.125327	0.052582			
		1177	0.121064	0.0587			
		1315	0.117101	0.06433			
		1453	0.113459	0.06999			
		1591	0.109783	0.075129			

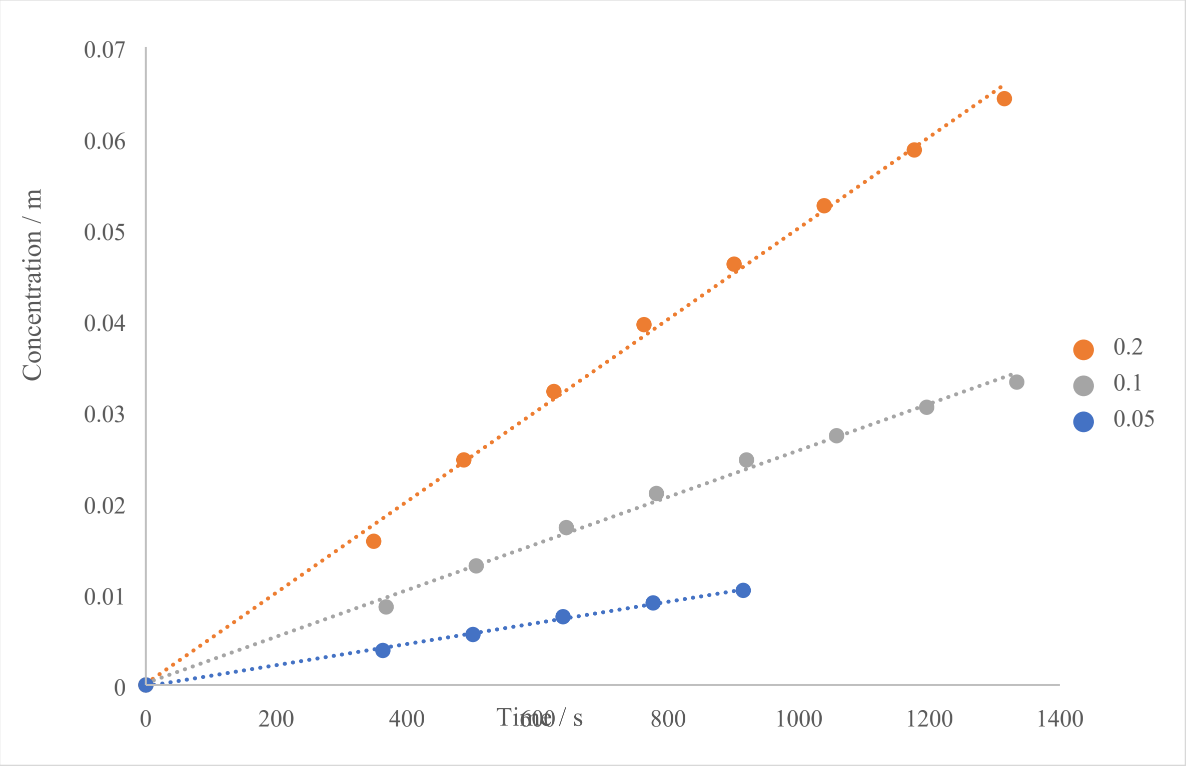

Figure S50. Plots of Stetter product concentration against time for the C_6F_5 triazolium precatalyst **16** (5 mM) catalysed Stetter reaction, at initial (E)-1,3-bis(4-chlorophenyl)prop-2-en-1-one **56** concentrations 0.05 M,

0.1 M, 0.2 M.

Entry 24

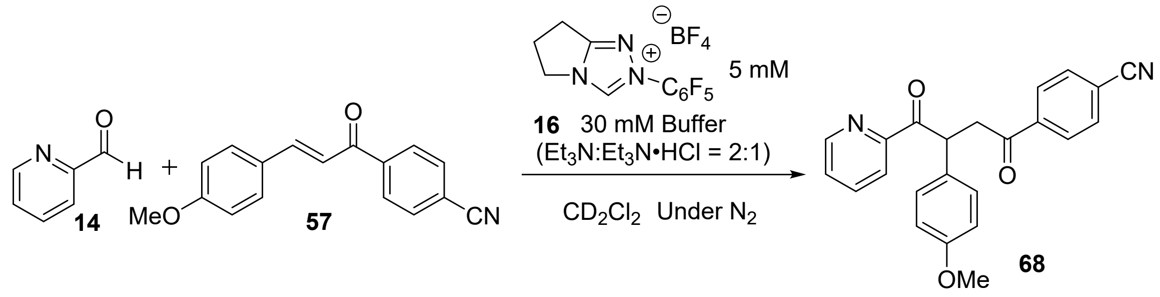

The reaction of pyridine-2-carboxaldehyde **14** and chalcone **57** catalysed by C_6F_5 triazolium salt **16** in triethylamine buffer was monitored using ^{1}H NMR spectra, with representative NMR spectra over the course of the experiment given in Figure S51.

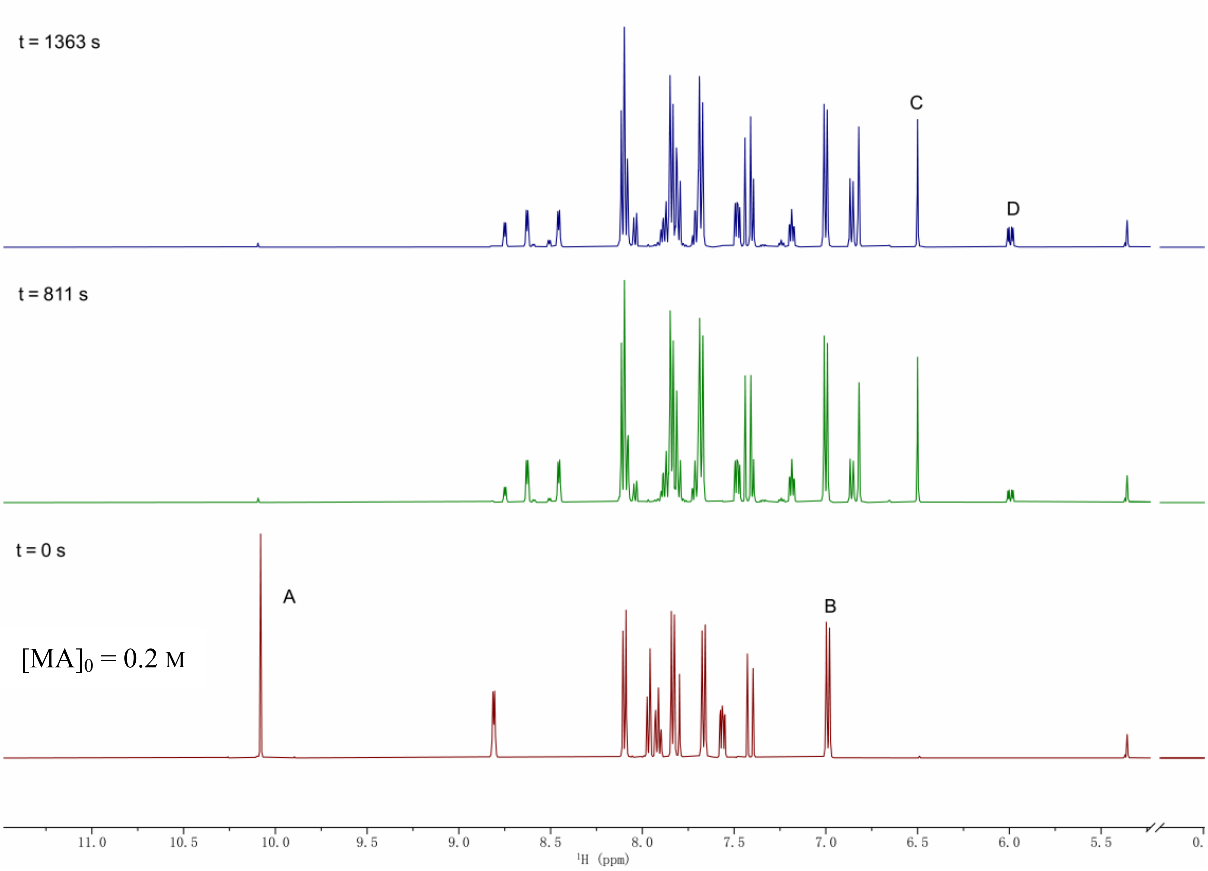

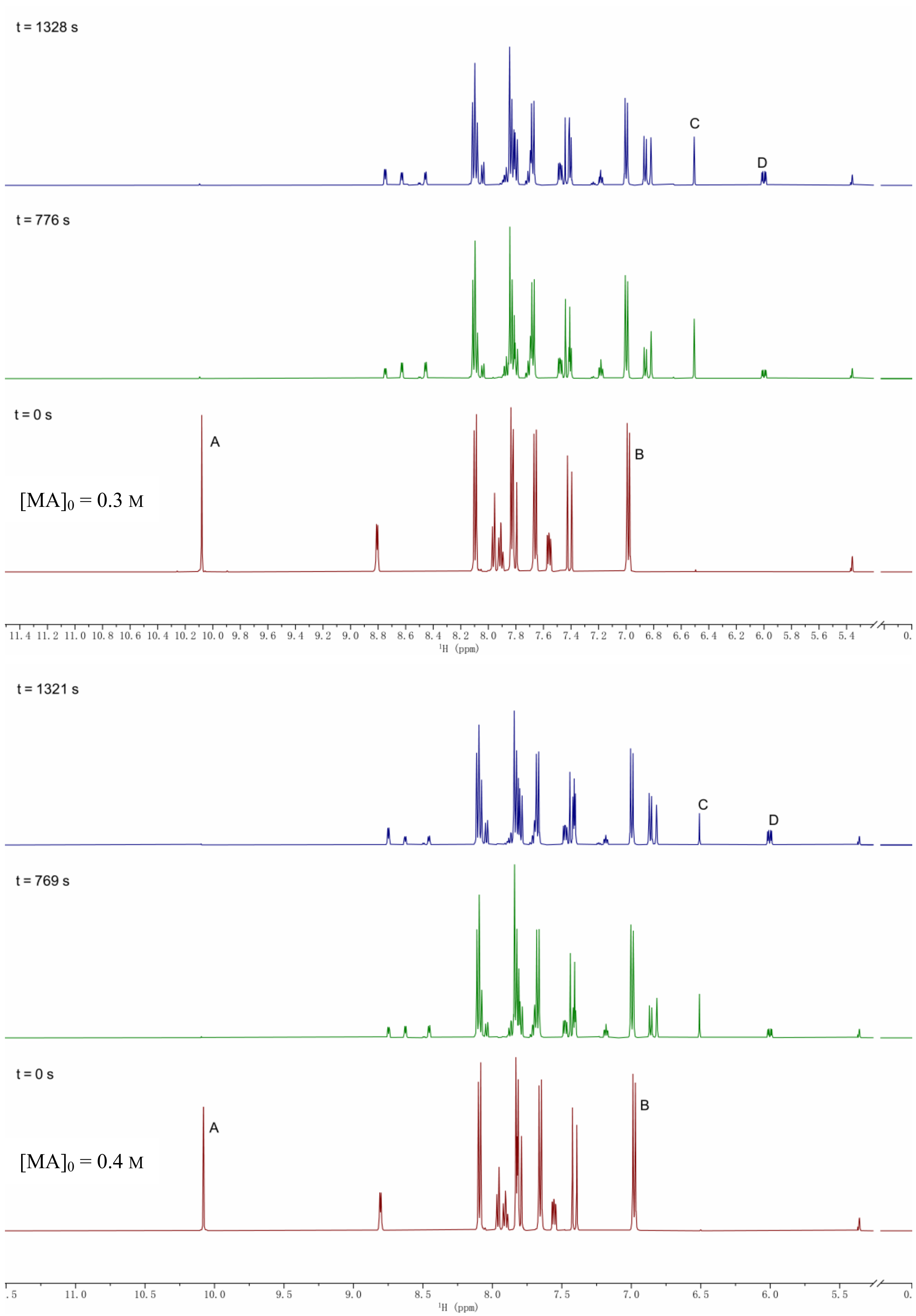

Figure S51. Representative ^{1}H NMR spectra (500 MHz) for reaction of pyridine-2-carboxaldehyde **14** (0.3 M) and chalcone **57** (0.2 M, 0.3 M and 0.4 M) catalysed by *N*-C_6F_5 NHC precursor **16** (5 mM) in NEt_3:NEt_3·HCl

(2:1, 0.03 M) in CD_2Cl_2 at 25 °C. A = Aldehyde C*H*O, B = Chalcone Ph*H*, C = Benzoin C*H*, D = Stetter product C*H*.

Table S24. Reaction data and initial rates of Stetter product formation from chalcone **57** and pyridine-2-carboxaldehyde **14**, catalysed by C_6F_5 triazolium precatalyst **16** (5 mM) in 0.03 M triethylamine buffer (Et_3N:Et_3N·HCl, 2:1) in CD_2Cl_2 at 25 °C.

[Aldehyde]$_0$ / M	[MA]$_0$ / M	Time / s	[Benzoin] / M	[Stetter] / M	v_{max} / 10^{-5} M s^{-1}	k_s' / 10^{-5} s^{-1}	Average / 10^{-5} s^{-1}
0.3	0.2	0	0	0	4.92	24.60	26.68±1.58
0.3	0.2	397	0.129346	0.019115	4.92	24.60	26.68±1.58
0.3	0.2	535	0.126762	0.027469	4.92	24.60	26.68±1.58
0.3	0.2	673	0.122173	0.034581	4.92	24.60	26.68±1.58
0.3	0.2	811	0.117871	0.041543	4.92	24.60	26.68±1.58
0.3	0.2	949	0.113547	0.04804	4.92	24.60	26.68±1.58
0.3	0.2	1087	0.109937	0.054754	4.92	24.60	26.68±1.58
0.3	0.2	1225	0.106158	0.060673	4.92	24.60	26.68±1.58
0.3	0.2	1363	0.102357	0.066161	4.92	24.60	26.68±1.58
0.3	0.3	0	0	0	8.53	28.16	26.68±1.58
0.3	0.3	362	0.129711	0.029752	8.53	28.16	26.68±1.58
0.3	0.3	500	0.124457	0.044472	8.53	28.16	26.68±1.58
0.3	0.3	638	0.117229	0.057848	8.53	28.16	26.68±1.58
0.3	0.3	776	0.110751	0.070059	8.53	28.16	26.68±1.58
0.3	0.3	914	0.104639	0.081442	8.53	28.16	26.68±1.58
0.3	0.3	1052	0.098954	0.092004	8.53	28.16	26.68±1.58
0.3	0.3	1190	0.093357	0.101639	8.53	28.16	26.68±1.58
0.3	0.3	1328	0.088773	0.111566	8.53	28.16	26.68±1.58
0.3	0.3	0	0	0	10.80	27.00	26.68±1.58
0.3	0.3	355	0.121243	0.036551	10.80	27.00	26.68±1.58
0.3	0.3	493	0.113565	0.055148	10.80	27.00	26.68±1.58
0.3	0.3	631	0.104955	0.071649	10.80	27.00	26.68±1.58
0.3	0.3	769	0.096851	0.087242	10.80	27.00	26.68±1.58
0.3	0.3	907	0.08921	0.101754	10.80	27.00	26.68±1.58
0.3	0.3	1045	0.082212	0.115143	10.80	27.00	26.68±1.58
0.3	0.3	1183	0.075512	0.128028	10.80	27.00	26.68±1.58
0.3	0.3	1321	0.06895	0.139211	10.80	27.00	26.68±1.58

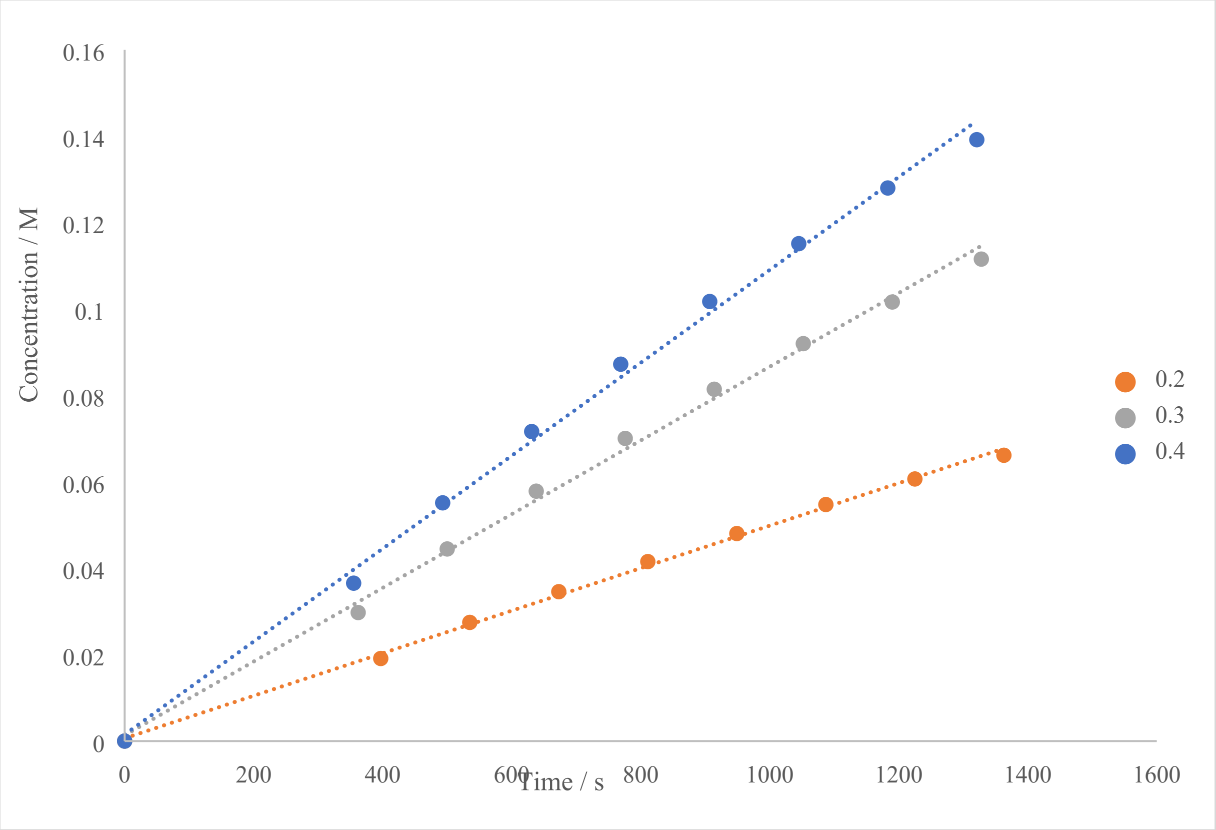

Figure S52. Plots of Stetter product concentration against time for the C_6F_5 triazolium precatalyst **16** (5 mM) catalysed Stetter reaction, at initial chalcone **57** concentrations 0.2 M, 0.3 M, 0.4 M.

Entry 25

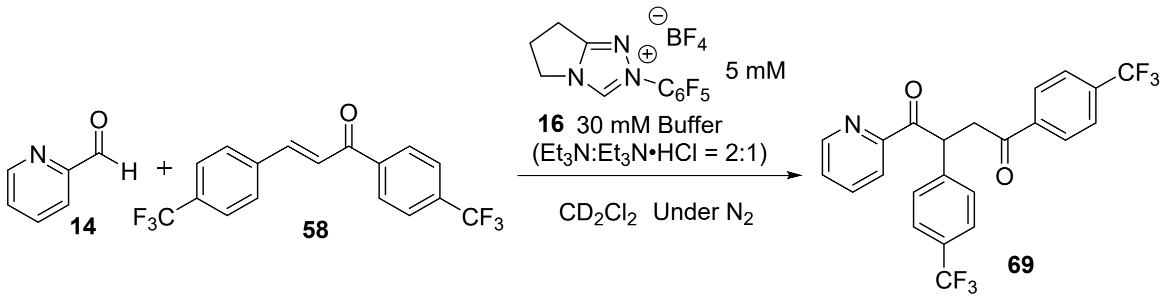

The reaction of pyridine-2-carboxaldehyde **14** and chalcone **58** catalysed by C_6F_5 triazolium salt **16** in triethylamine buffer was monitored using ^{1}H NMR spectra, with representative NMR spectra over the course of the experiment given in Figure S53.

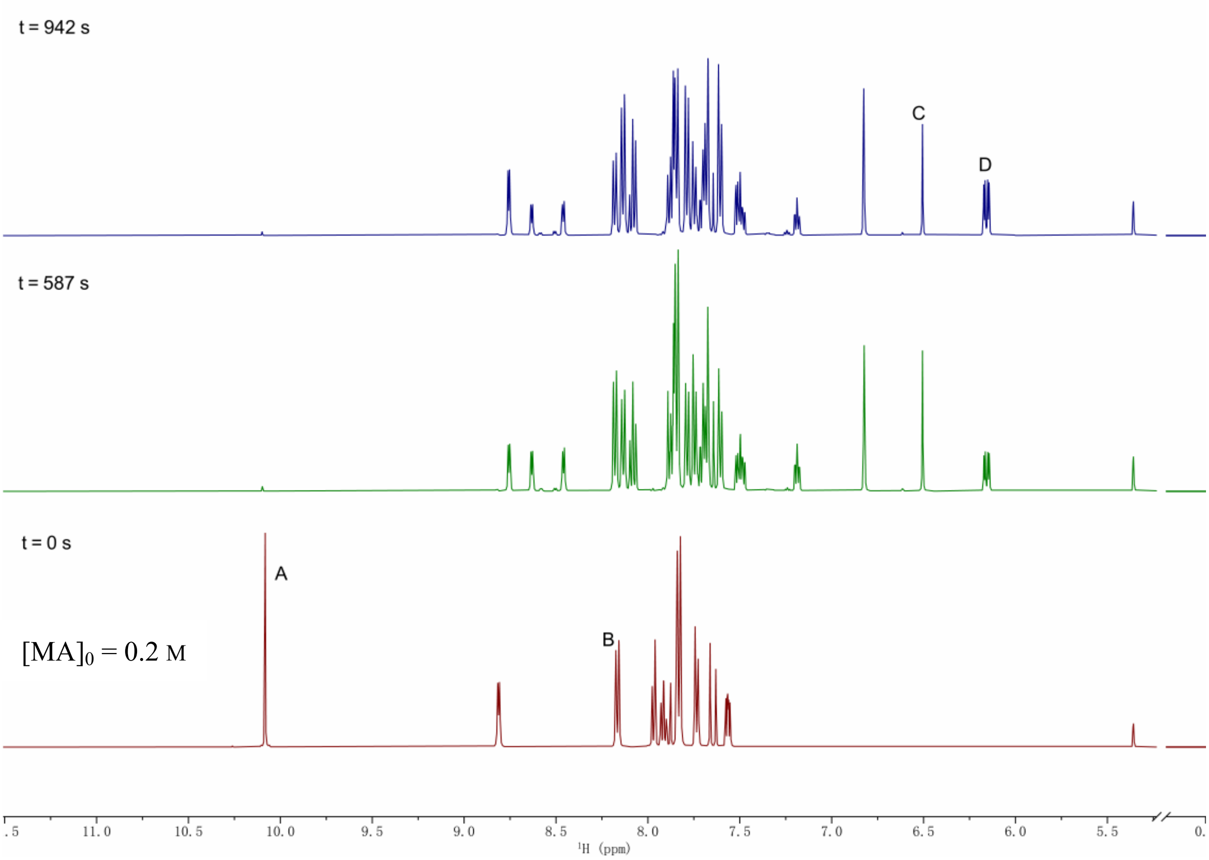
t = 942 s
C
D
t = 587 s
t = 0 s
A
[MA]0 = 0.2 M
B
.5
11.0
10.5
10.0
9.5
9.0
8.5
8.0
7.5
7.0
6.5
6.0
5.5
0.
1H (ppm)

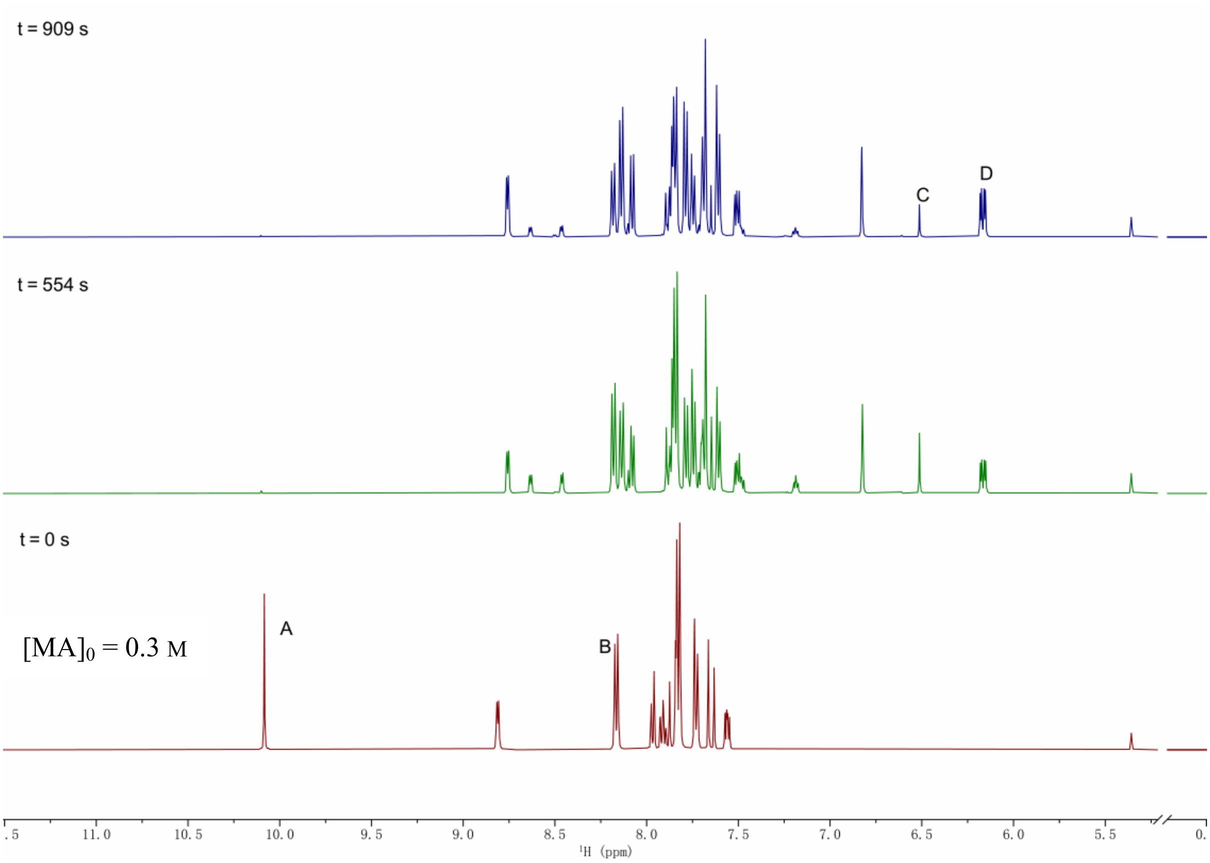
t = 909 s
D
C
t = 554 s
t = 0 s
A
[MA]0 = 0.3 M
B
.5
11.0
10.5
10.0
9.5
9.0
8.5
8.0
7.5
7.0
6.5
6.0
5.5
0.
1H (ppm)

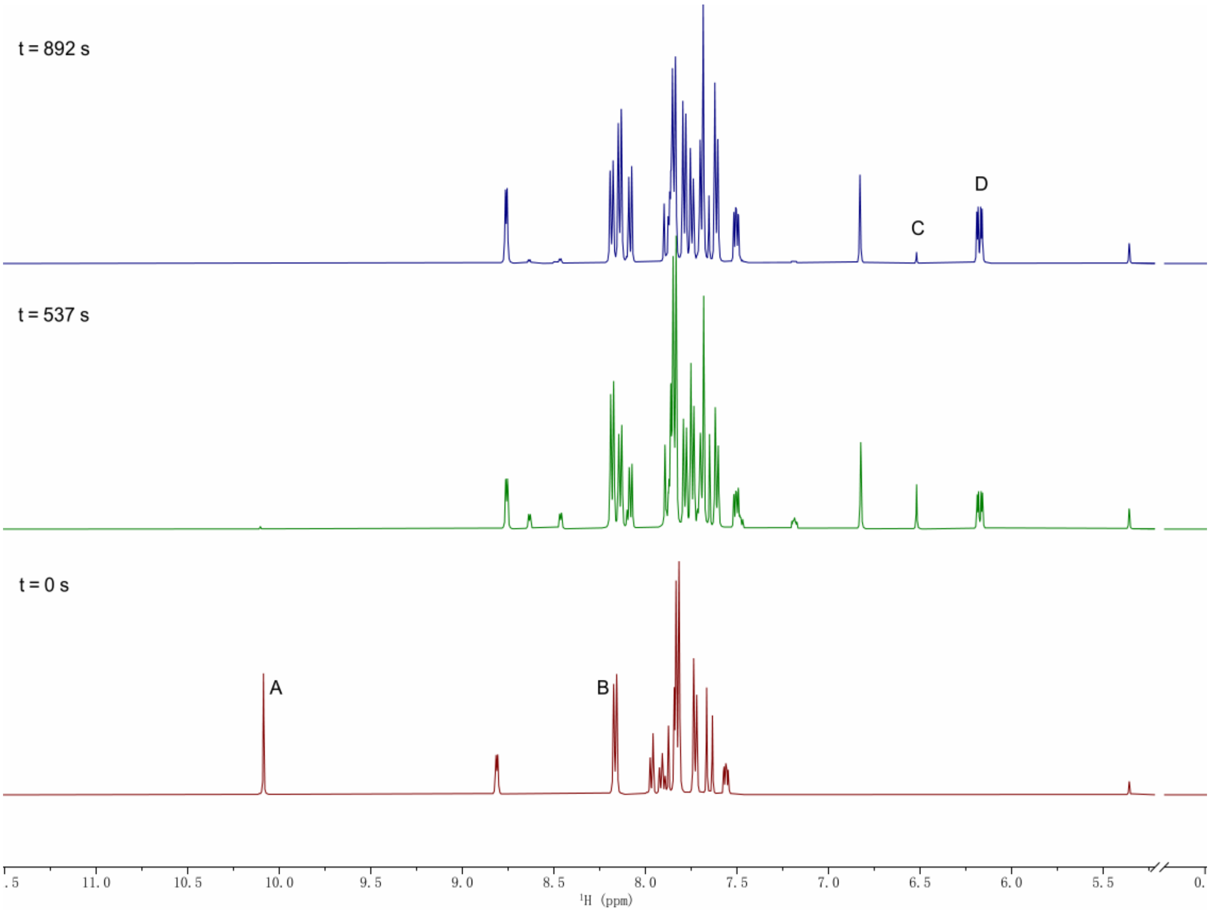

Figure S53. Representative ^{1}H NMR spectra (500 MHz) for reaction of pyridine-2-carboxaldehyde **14** (0.3 M) and chalcone **58** (0.2 M, 0.3 M and 0.4 M) catalysed by *N*-C_6F_5 NHC precursor **16** (5 mM) in NEt_3:NEt_3·HCl (2:1, 0.03 M) in CD_2Cl_2 at 25 °C. A = Aldehyde C*H*O, B = Chalcone Ph*H*, C = Benzoin C*H*, D = Stetter product C*H*.

Table S25. Reaction data and initial rates of Stetter product formation from chalcone **58** and pyridine-2-carboxaldehyde **14**, catalysed by C_6F_5 triazolium precatalyst **16** (5 mM) in 0.03 M triethylamine buffer (Et_3N:Et_3N·HCl, 2:1) in CD_2Cl_2 at 25 °C.

**[Aldehyde]$_0$ / M**	**[MA]$_0$ / M**	**Time / s**	**[Benzoin] / M**	**[Stetter] / M**	v_{max} / **10^{-5} M s^{-1}**	k_s' / **10^{-5} s^{-1}**	**Average / 10^{-5} s^{-1}**
0.3	0.2	0	0	0	17.60	88.00	87.16±2.60
		303	0.112205	0.052517			
		374	0.108349	0.068931			
		445	0.101889	0.08171			
		516	0.095737	0.093246			
		587	0.090491	0.103941			
		658	0.085651	0.113615			
		729	0.08129	0.122698			
		800	0.077209	0.130926			
		871	0.073411	0.138054			
		942	0.070055	0.145058			

		0	0	0			
		270	0.102365	0.0713			
		341	0.093204	0.095369			
		412	0.083488	0.115708			
		483	0.074288	0.13447			
0.3	0.3	554	0.066191	0.151254	27.10	90.33	
		625	0.059021	0.167064			
		696	0.052186	0.180757			
		767	0.04606	0.193535			
		838	0.040451	0.206045			
		909	0.035424	0.216506			
		0	0	0			
		253	0.099609	0.078533			
		324	0.087258	0.108521			
		395	0.074629	0.134523			
		466	0.063054	0.158418			
0.3	0.4	537	0.052463	0.180592	33.60	84.00	
		608	0.043252	0.199948			
		679	0.034521	0.218801			
		750	0.026663	0.2358			
		821	0.019424	0.251742			
		892	0.012929	0.265595			

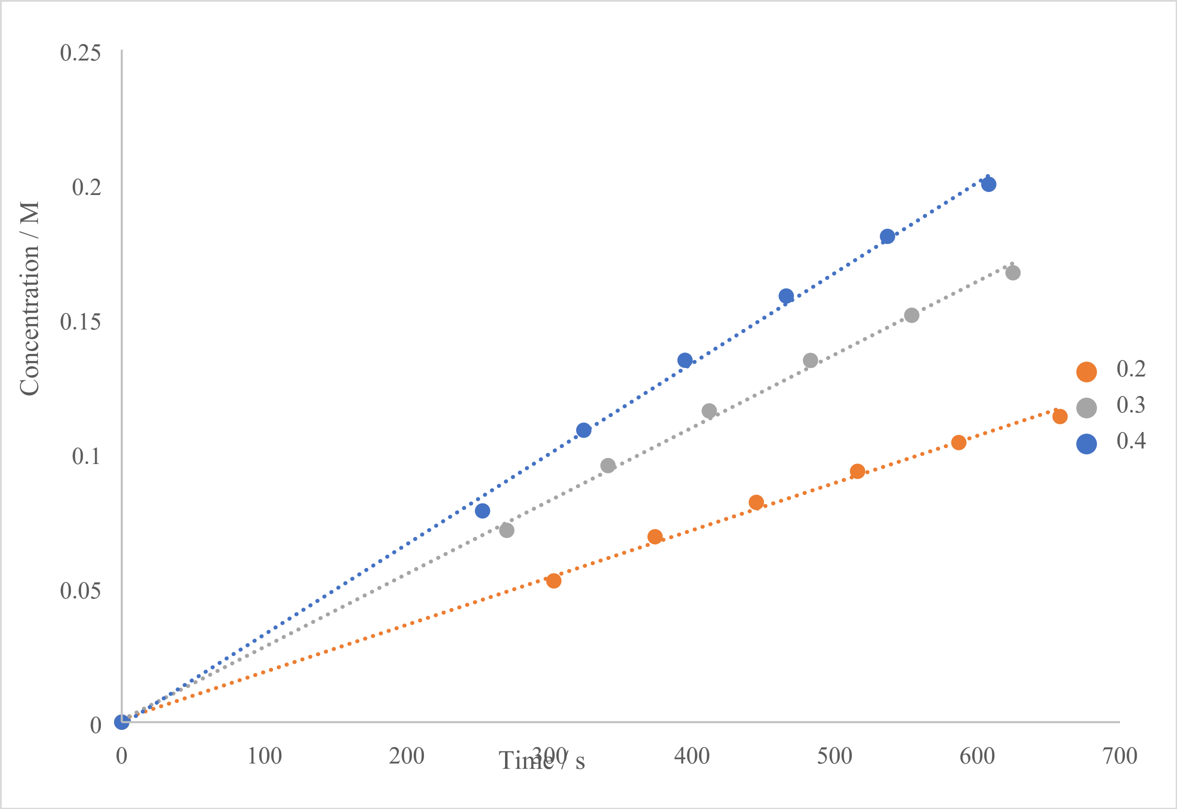

Figure S54. Plots of Stetter product concentration against time for the C_6F_5 triazolium precatalyst **16** (5 mM)

catalysed Stetter reaction, at initial chalcone **58** concentrations 0.2 M, 0.3 M, 0.4 M.

Entry 26

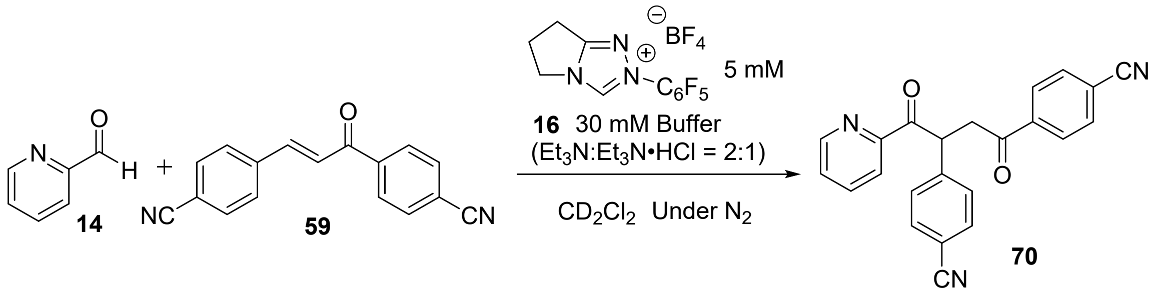

The reaction of pyridine-2-carboxaldehyde **14** and chalcone **59** catalysed by C_6F_5 triazolium salt **16** in triethylamine buffer was monitored using ^{1}H NMR spectra, with representative NMR spectra over the course of the experiment given in Figure S55.

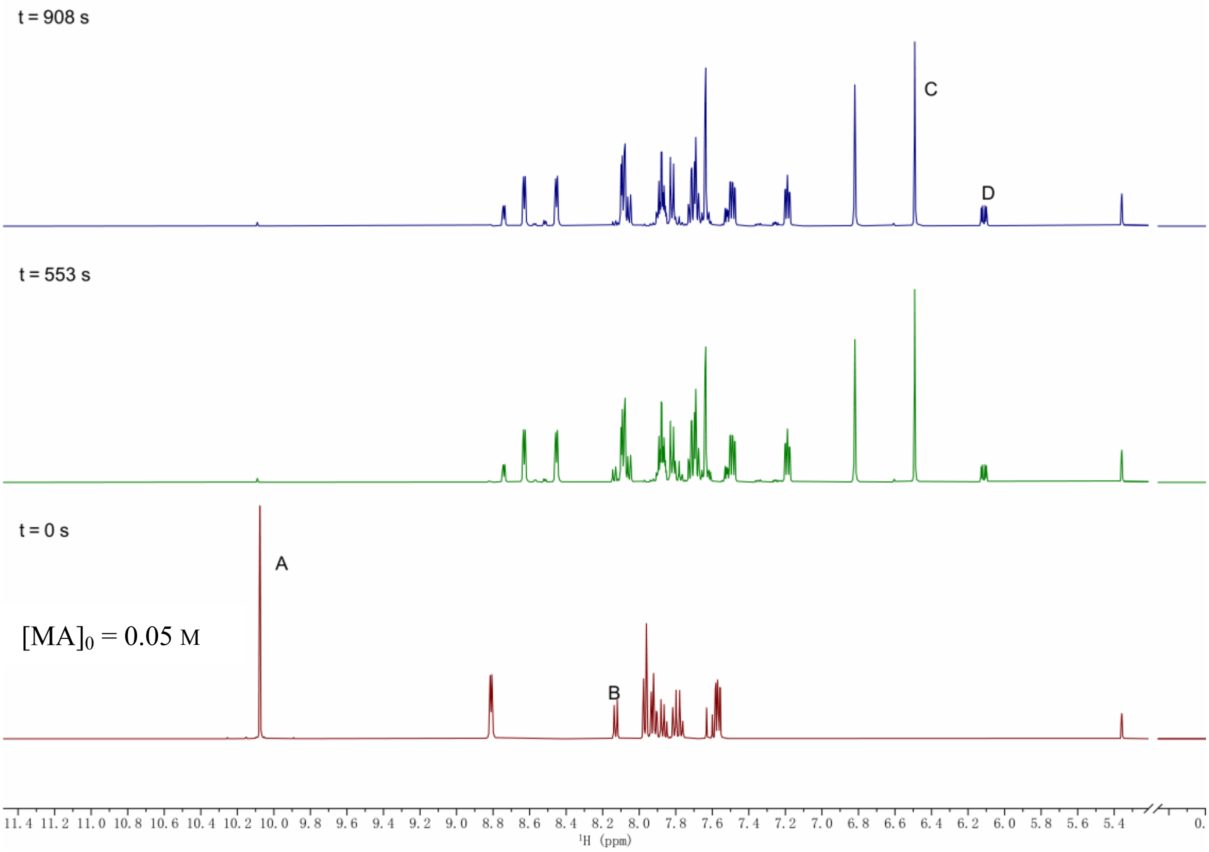

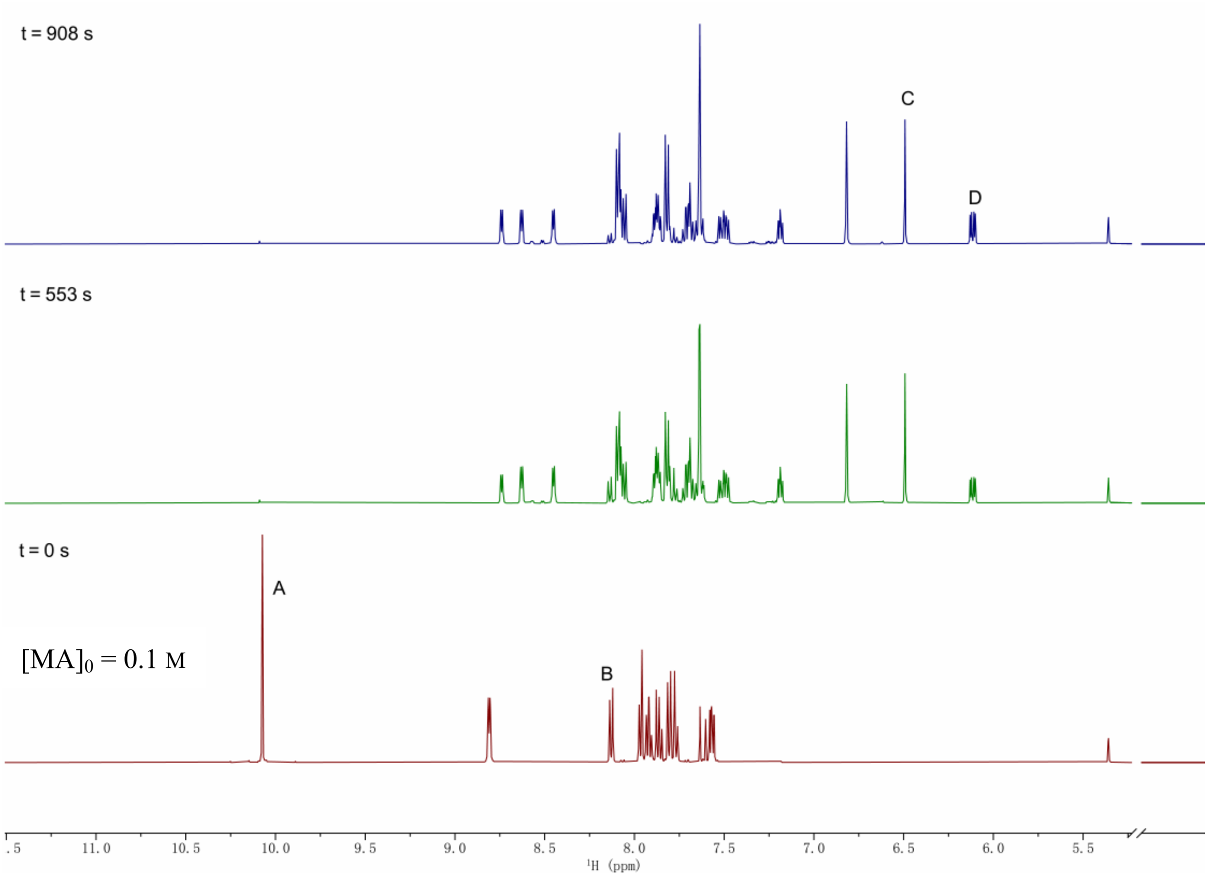

Figure S55. Representative ^{1}H NMR spectra (500 MHz) for reaction of pyridine-2-carboxaldehyde **14** (0.3 M) and chalcone **59** (0.05 M, and 0.1 M) catalysed by *N*-C_6F_5 NHC precursor **16** (5 mM) in NEt_3:NEt_3·HCl (2:1, 0.03 M) in CD_2Cl_2 at 25 °C. A = Aldehyde C*H*O, B = Chalcone Ph*H*, C = Benzoin C*H*, D = Stetter product C*H*.

Table S26. Reaction data and initial rates of Stetter product formation from chalcone **69** and pyridine-2-carboxaldehyde **14**, catalysed by C_6F_5 triazolium precatalyst **16** (5 mM) in 0.03 M triethylamine buffer (Et_3N:Et_3N·HCl, 2:1) in CD_2Cl_2 at 25 °C.

**[Aldehyde]$_0$ / M**	**[MA]$_0$ / M**	**Time / s**	**[Benzoin] / M**	**[Stetter] / M**	**v_{max} / 10^{-5} M s^{-1}**	**k'_s / 10^{-5} s^{-1}**	**Average / 10^{-5} s^{-1}**
		0	0	0			
		269	0.117374	0.046319			
		340	0.113665	0.05794			
		411	0.109458	0.066034			
		482	0.105966	0.072438			
0.3	0.05	553	0.103094	0.077536	8.30	166.0	
		624	0.100794	0.081742			165±1.00
		695	0.098816	0.085096			
		766	0.097183	0.087687			
		837	0.095563	0.089584			
		908	0.094363	0.091611			
0.3	0.1	0	0	0	16.40	164.0	

		269	0.129832	0.023443			
		340	0.129413	0.029062			
		411	0.126988	0.033568			
		482	0.12481	0.036871			
		553	0.123119	0.039327			
		624	0.121241	0.041036			
		695	0.120038	0.042476			
		766	0.119028	0.043805			
		837	0.118392	0.044947			
		908	0.117337	0.045662			

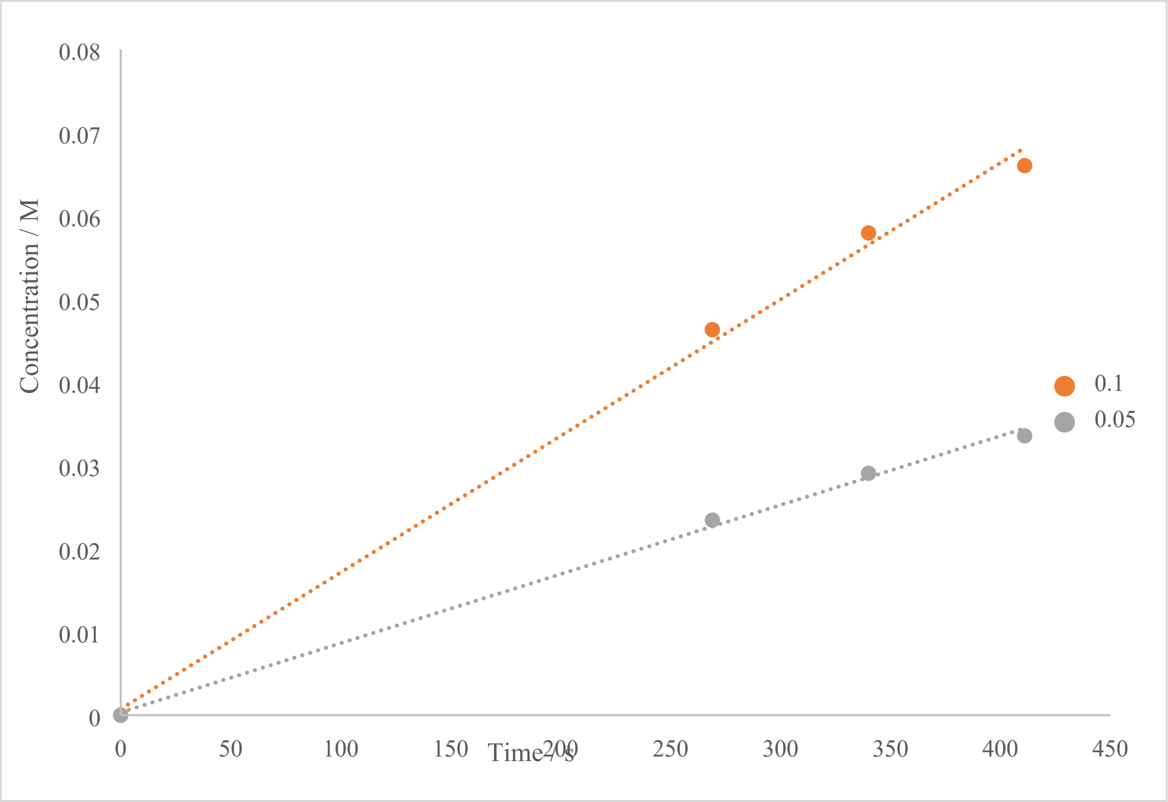

Figure S56. Plots of Stetter product concentration against time for the C_6F_5 triazolium precatalyst **16** (5 mM) catalysed Stetter reaction, at initial chalcone **59** concentrations 0.05 M, 0.1 M.

Entry 27

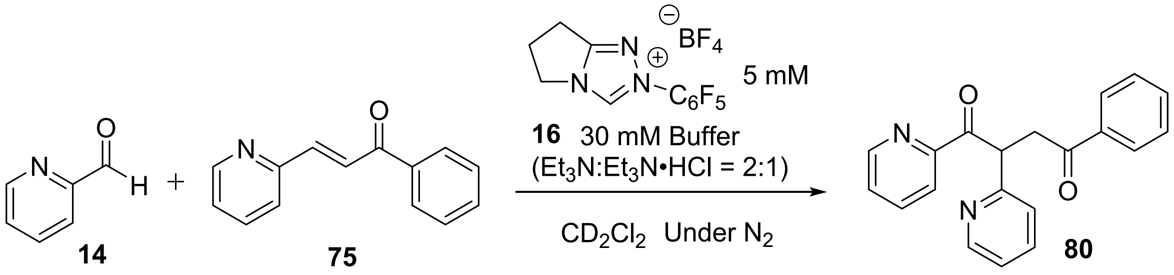

The reaction of pyridine-2-carboxaldehyde **14** and Michael acceptor **75** catalysed by C_6F_5 triazolium salt **16** in triethylamine buffer was monitored using ^1H NMR spectra, with representative NMR spectra over the

course of the experiment given in Figure S57.

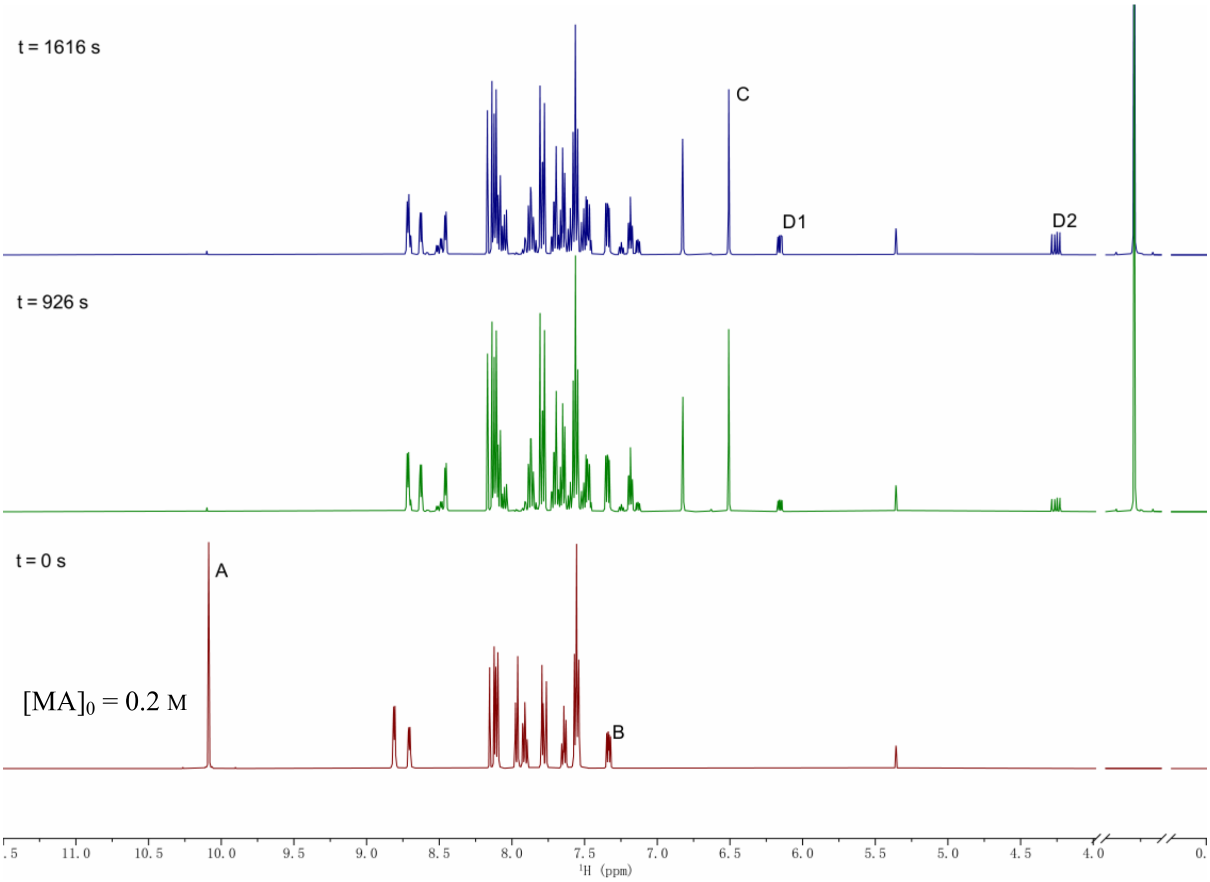

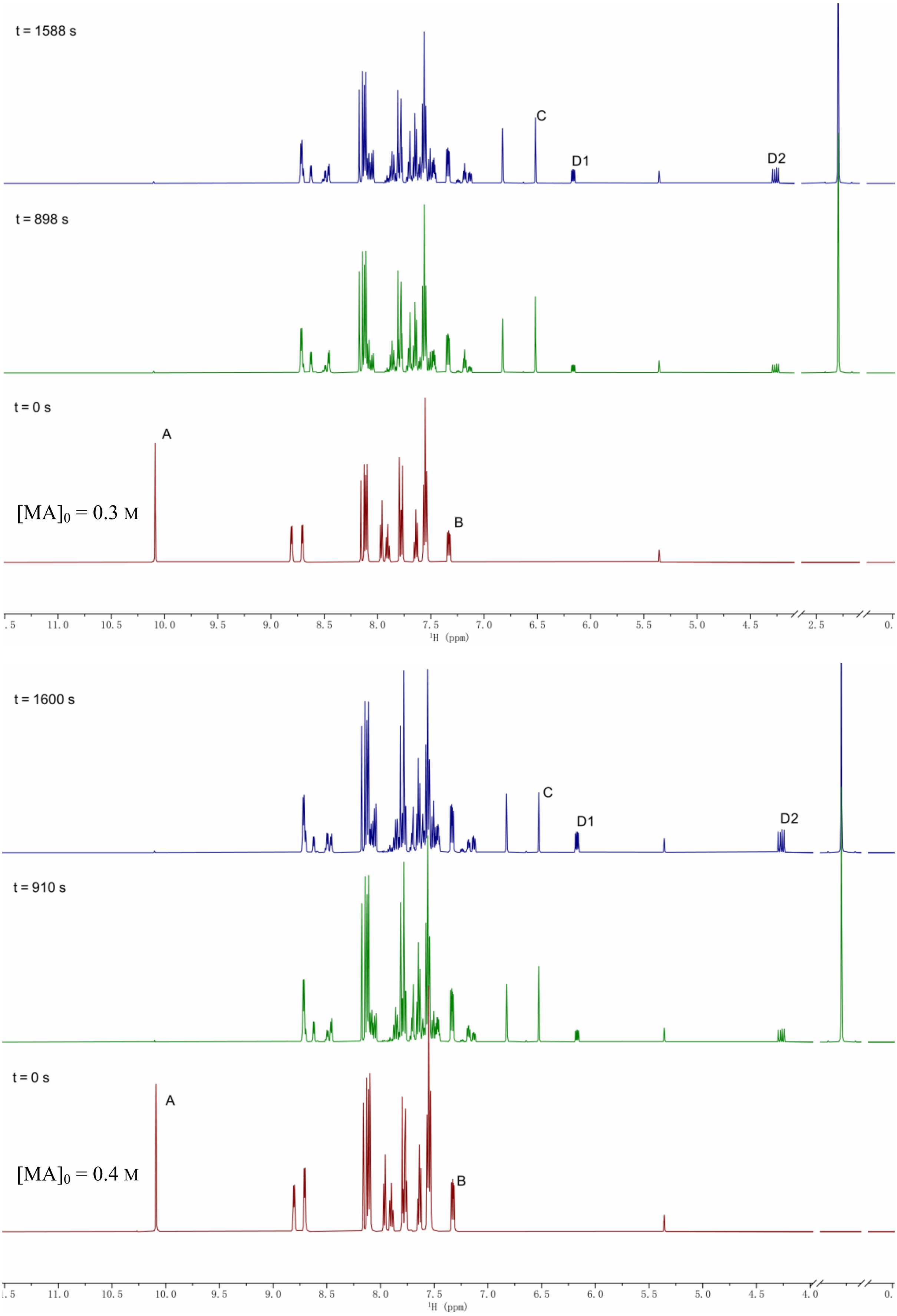

Figure S57. Representative ^{1}H NMR spectra (500 MHz) for reaction of pyridine-2-carboxaldehyde **14** (0.3

M) and Michael acceptor **75** (0.2 M, 0.3 M and 0.4 M) catalysed by *N*-C_6F_5 NHC precursor **16** (5 mM) in NEt_3:NEt_3·HCl (2:1, 0.03 M) in CD_2Cl_2 at 25 °C. A = Aldehyde C*H*O, B = Michael acceptor Ar*H*, C = Benzoin C*H*, D1 = Stetter product C*H*, D2 = Stetter product CH_AH_B.

Table S27. Reaction data and initial rates of Stetter product formation from Michael acceptor **75**, catalyzed by C_6F_5 triazolium precatalyst **16** (5 mM) in 0.03 M triethylamine buffer (Et_3N:Et_3N·HCl, 2:1) in CD_2Cl_2 at 25 °C.

**[Aldehyde]$_0$ / M**	**[MA]$_0$ / M**	**Time / s**	**[Benzoin] / M**	**[Stetter] / M**	**v_{max} / 10^{-5} M s^{-1}**	**k_s' / 10^{-5} s^{-1}**	**Average / 10^{-5} s^{-1}**
0.3	0.2	0	0	0	2.87	14.35	14.69±0.40
		374	0.131274	0.010527			
		512	0.127627	0.015051			
		650	0.124139	0.019556			
		788	0.120839	0.023785			
		926	0.117733	0.027472			
		1064	0.114967	0.030956			
		1202	0.112002	0.034574			
		1340	0.109355	0.038172			
		1478	0.107085	0.041401			
		1616	0.104038	0.044596			
0.3	0.3	0	0	0	4.34	14.47	
		346	0.126593	0.013906			
		484	0.121526	0.020877			
		622	0.117019	0.02754			
		760	0.112684	0.03356			
		898	0.108359	0.039435			
		1036	0.104769	0.045276			
		1174	0.100496	0.050858			
		1312	0.096955	0.056053			
		1450	0.093117	0.061369			
		1588	0.089958	0.06642			
0.3	0.4	0	0	0	6.10	15.25	
		358	0.12341	0.019933			
		496	0.117284	0.029839			
		634	0.111655	0.0389			
		772	0.106123	0.047448			
		910	0.100278	0.05564			
		1048	0.095298	0.064083			
		1186	0.089858	0.071741			
		1324	0.085687	0.079728			
		1462	0.080791	0.087559			
		1600	0.076102	0.094556			

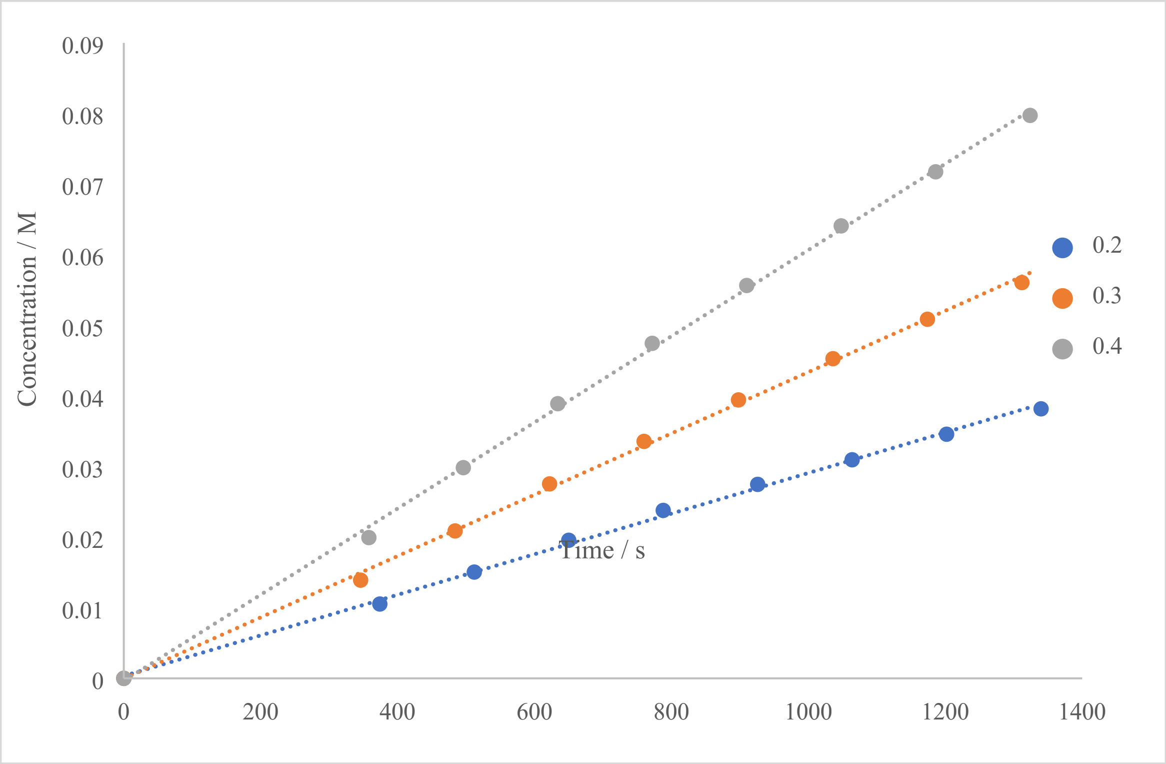

Figure S58. Plots of Stetter product concentration against time for the C_6F_5 triazolium precatalyst **16** (5 mM) catalysed Stetter reaction, at initial Michael acceptor **75** concentrations 0.2 M, 0.3 M, 0.4 M.

Entry 28

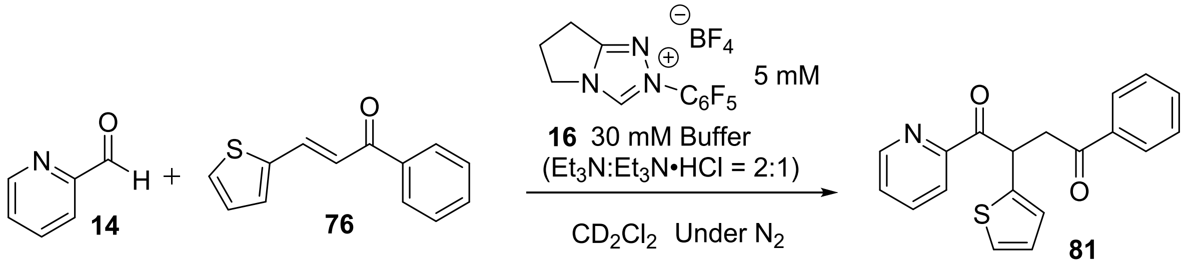

The reaction of pyridine-2-carboxaldehyde **14** and Michael acceptor **76** catalysed by C_6F_5 triazolium salt **16** in triethylamine buffer was monitored using ^{1}H NMR spectra, with representative NMR spectra over the course of the experiment given in Figure S59.

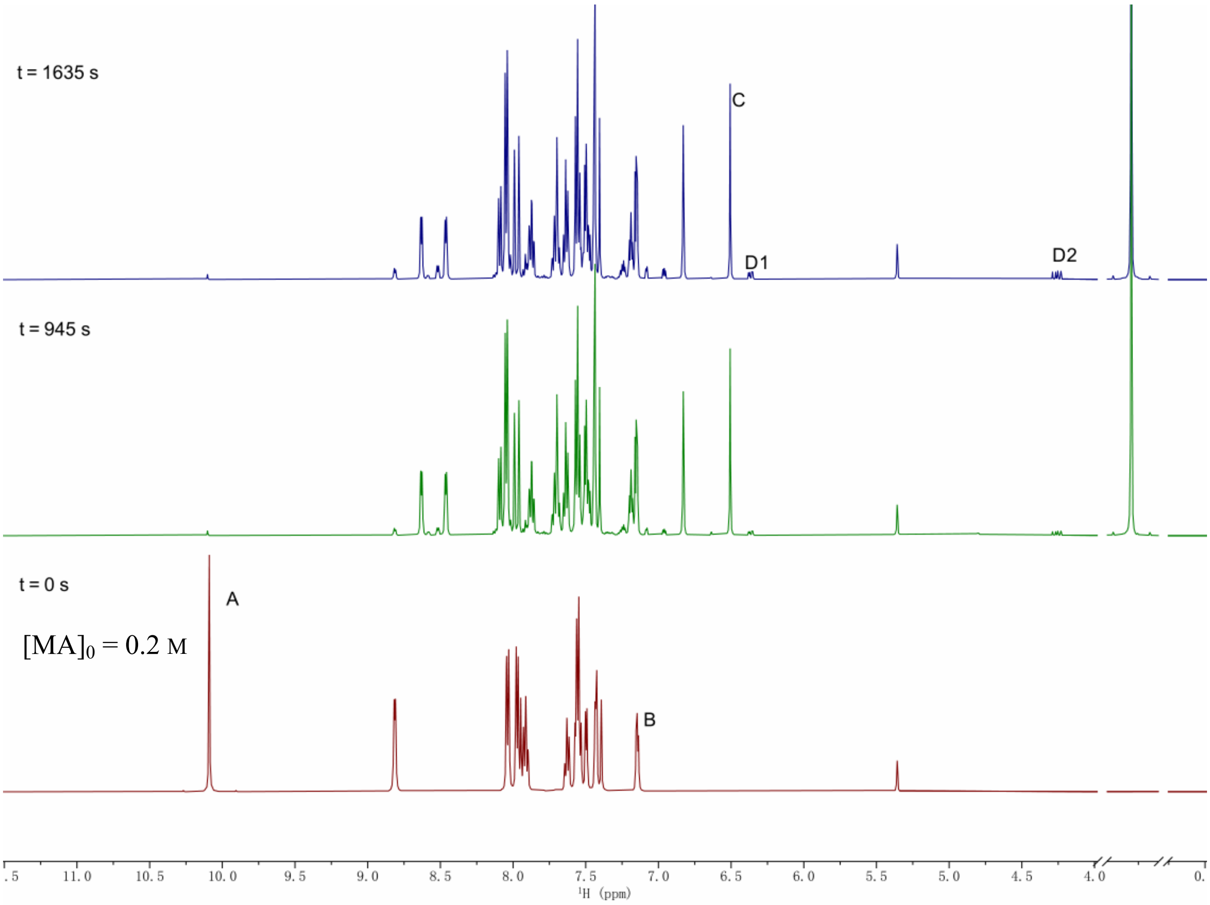
t = 1635 s
C
D1
D2
t = 945 s
t = 0 s
A
[MA]0 = 0.2 M
B
¹H (ppm)

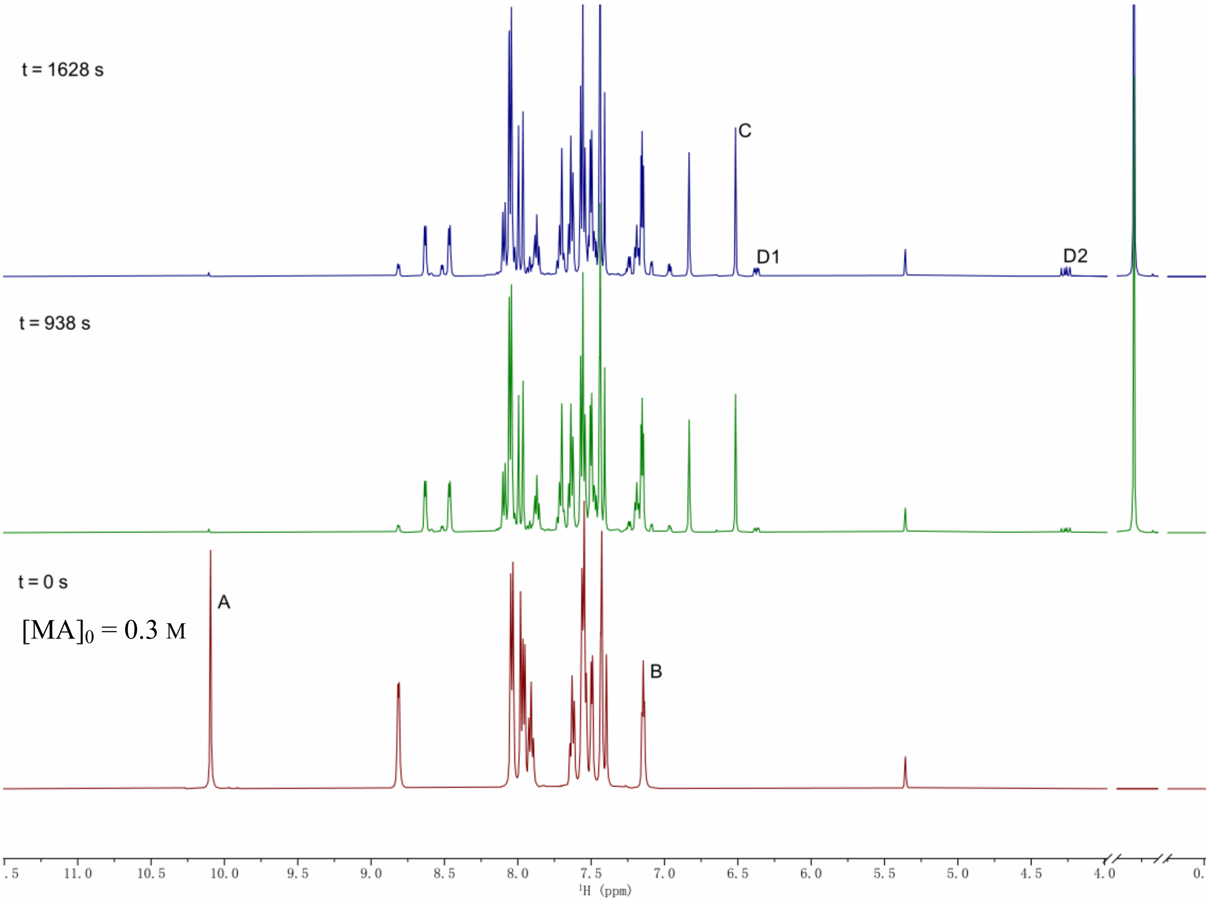
t = 1628 s
C
D1
D2
t = 938 s
t = 0 s
A
[MA]0 = 0.3 M
B
¹H (ppm)

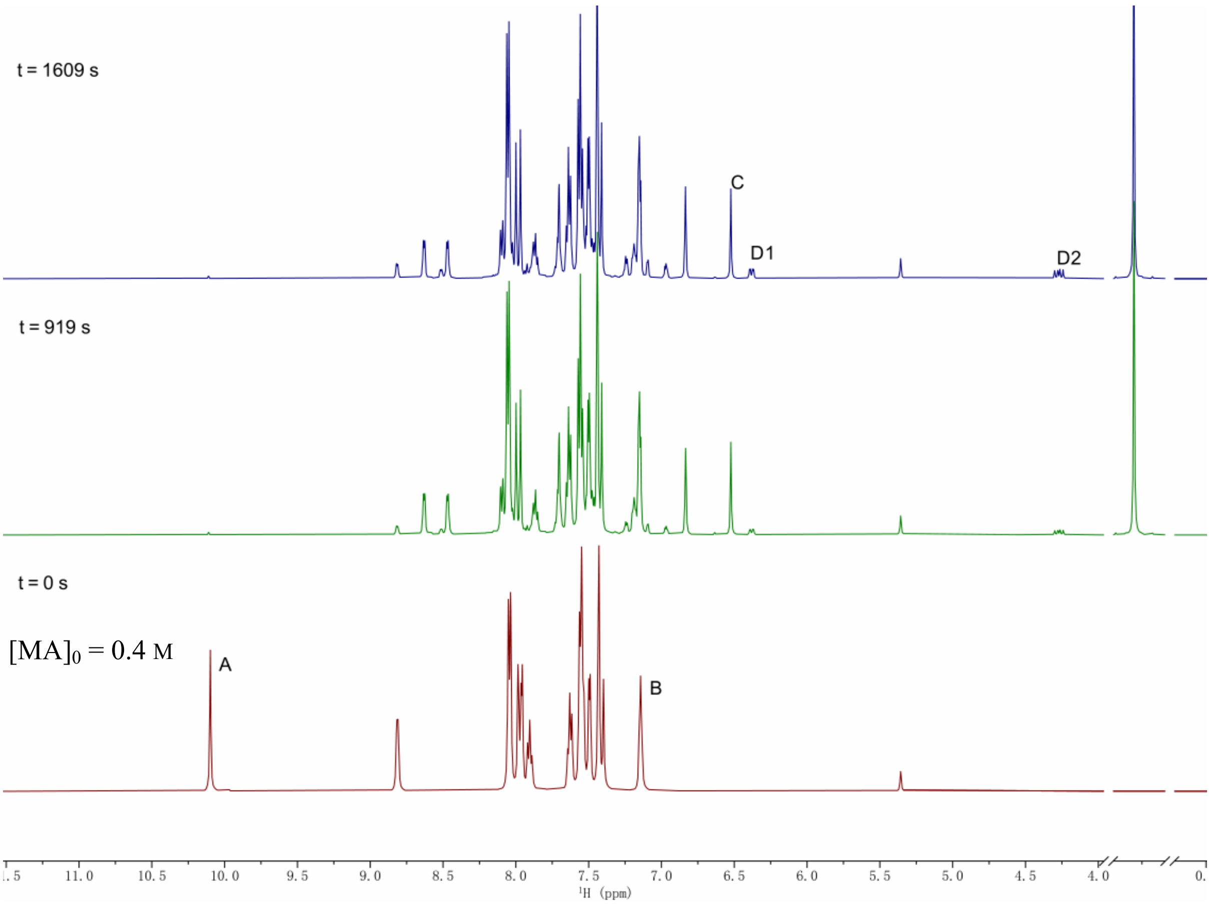

Figure S59. Representative ^{1}H NMR spectra (500 MHz) for reaction of pyridine-2-carboxaldehyde **14** (0.3 M) and Michael acceptor **76** (0.2 M, 0.3 M and 0.4 M) catalysed by *N*-C_6F_5 NHC precursor **16** (5 mM) in NEt_3:NEt_3·HCl (2:1, 0.03 M) in CD_2Cl_2 at 25 °C. A = Aldehyde C*H*O, B = Michael acceptor Ar*H*, C = Benzoin C*H*, D1 = Stetter product C*H*, D2 = Stetter product C$H_A$$H_B$.

Table S28. Reaction data and initial rates of Stetter product formation from Michael acceptor **76**, catalyzed by C_6F_5 triazolium precatalyst **16** (5 mM) in 0.03 M triethylamine buffer (Et_3N:Et_3N·HCl, 2:1) in CD_2Cl_2 at 25 °C.

**[Aldehyde]$_0$ / M**	**[MA]$_0$ / M**	**Time / s**	**[Benzoin] / M**	**[Stetter] / M**	**v_{max} / 10^{-5} M s^{-1}**	**k_s' / 10^{-5} s^{-1}**	**Average / 10^{-5} s^{-1}**
0.3	0.2	0	0	0	0.704	3.56	3.76±0.17
		393	0.127374	0.002669			
		531	0.126176	0.003907			
		669	0.123617	0.004878			
		807	0.119801	0.005816			
		945	0.118596	0.006571			
		1083	0.1162	0.007627			
		1221	0.113859	0.008528			
		1359	0.11194	0.009685			
		1497	0.110997	0.010718			
		1635	0.107285	0.011723			

0.3	0.3	0	0	0	1.16	3.93	
		386	0.124075	0.003999			
		524	0.122459	0.005823			
		662	0.120102	0.007582			
		800	0.116437	0.009038			
		938	0.114575	0.010585			
		1076	0.11322	0.012123			
		1214	0.111888	0.013763			
		1352	0.109898	0.01572			
		1490	0.108205	0.017277			
		1628	0.106211	0.019203			
0.3	0.4	0	0	0	1.56	3.90	
		367	0.120226	0.004996			
		505	0.119358	0.007551			
		643	0.116098	0.009889			
		781	0.11318	0.012348			
		919	0.110995	0.014592			
		1057	0.107092	0.01586			
		1195	0.105999	0.01884			
		1333	0.102775	0.020116			
		1471	0.099665	0.025258			
		1609	0.098746	0.02791			

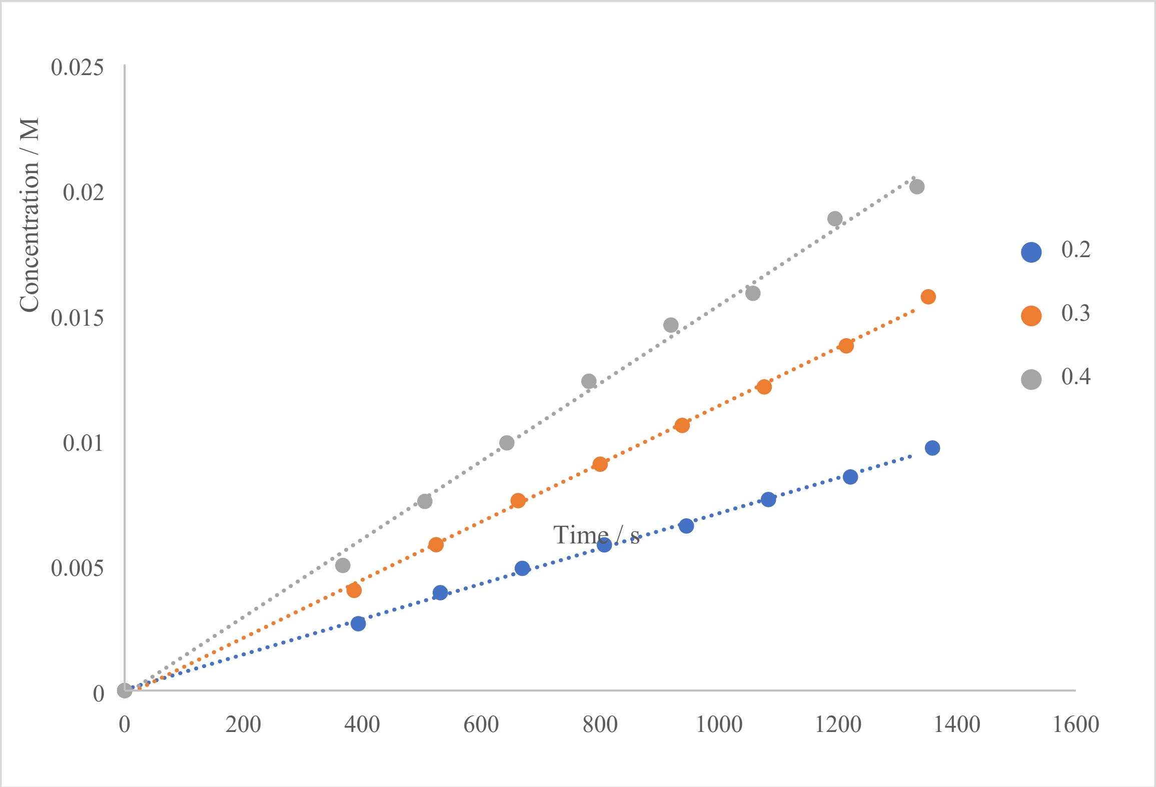

Figure S60. Plots of Stetter product concentration against time for the C_6F_5 triazolium precatalyst **16** (5 mM)

catalysed Stetter reaction, at initial Michael acceptor **76** concentrations 0.2 M, 0.3 M, 0.4 M.

Entry 29

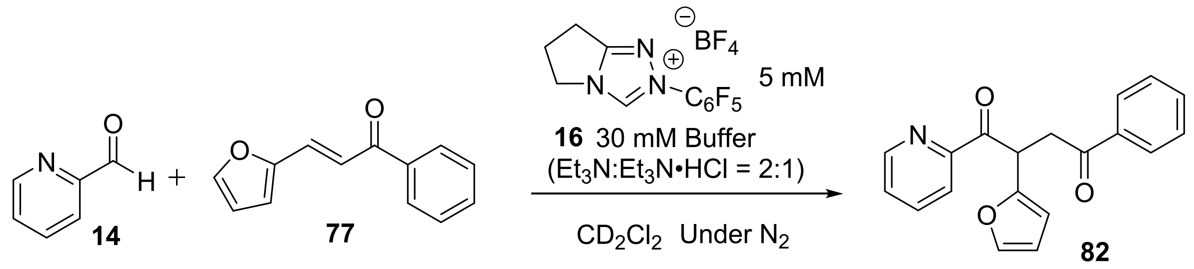

The reaction of pyridine-2-carboxaldehyde **14** and Michael acceptor **77** catalysed by C_6F_5 triazolium salt **16** in triethylamine buffer was monitored using ^{1}H NMR spectra, with representative NMR spectra over the course of the experiment given in Figure S61.

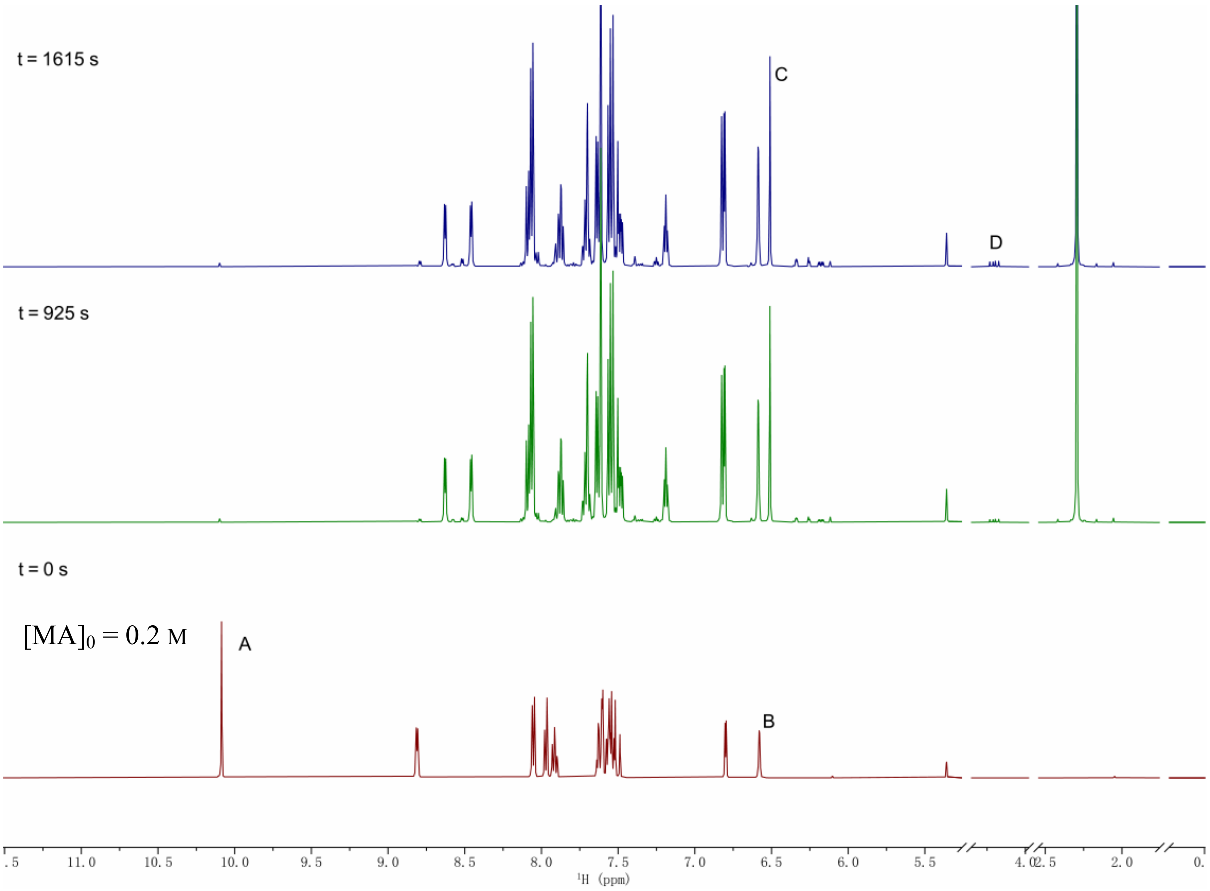

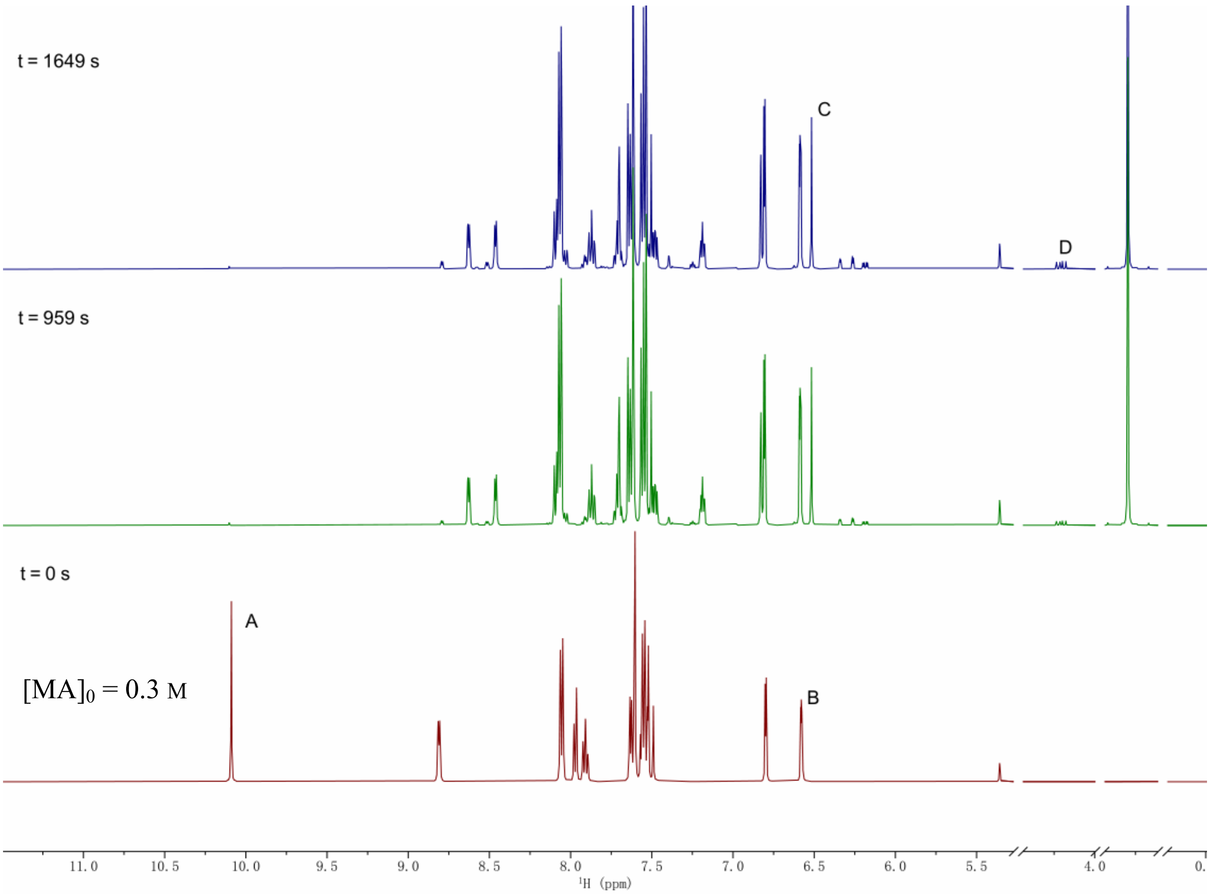
t = 1649 s
C
D
t = 959 s
t = 0 s
A
[MA]$_0$ = 0.3 M
B
11.0
10.5
10.0
9.5
9.0
8.5
8.0
7.5
7.0
6.5
6.0
5.5
4.0
0.
^{1}H (ppm)

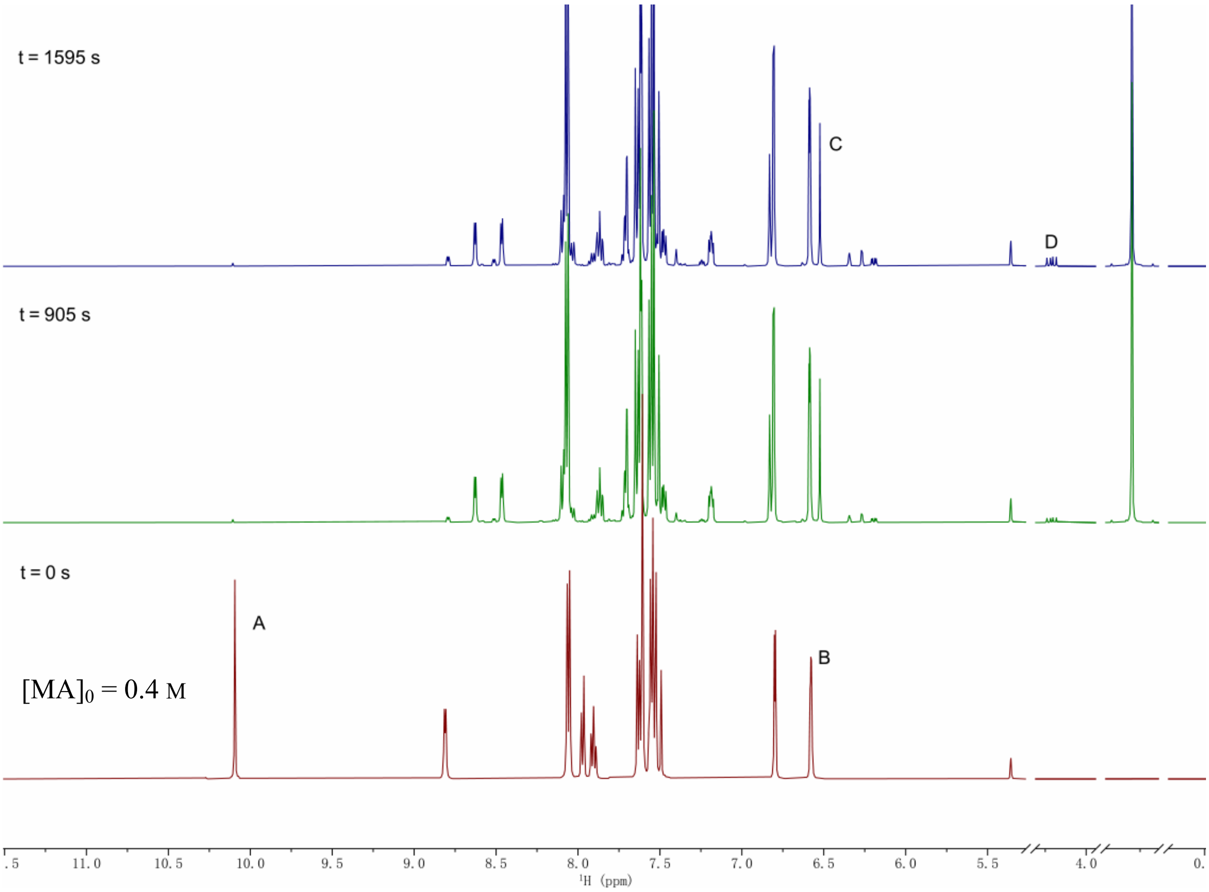
t = 1595 s
C
D
t = 905 s
t = 0 s
A
[MA]$_0$ = 0.4 M
B
.5
11.0
10.5
10.0
9.5
9.0
8.5
8.0
7.5
7.0
6.5
6.0
5.5
4.0
0.
^{1}H (ppm)

Figure S60. Representative ^{1}H NMR spectra (500 MHz) for reaction of pyridine-2-carboxaldehyde **14** (0.3 M) and Michael acceptor **77** (0.2 M, 0.3 M and 0.4 M) catalysed by *N*-C_6F_5 NHC precursor **16** (5 mM) in NEt_3:NEt_3·HCl (2:1, 0.03 M) in CD_2Cl_2 at 25 °C. A = Aldehyde C*H*O, B = Michael acceptor Ar*H*, C = Benzoin C*H*, D = Stetter product CH_AH_B.

Table S29. Reaction data and initial rates of Stetter product formation from Michael acceptor **77**, catalyzed by C_6F_5 triazolium precatalyst **16** (5 mM) in 0.03 M triethylamine buffer (Et_3N:Et_3N·HCl, 2:1) in CD_2Cl_2 at 25 °C.

[Aldehyde]$_0$ / M	[MA]$_0$ / M	Time / s	[Benzoin] / M	[Stetter] / M	v_{max} / 10^{-5} M s^{-1}	k_s' / 10^{-5} s^{-1}	Average / 10^{-5} s^{-1}
0.3	0.2	0	0	0	0.767	3.84	3.93±0.06
		373	0.137476	0.003005			
		511	0.135471	0.003789			
		649	0.133702	0.005264			
		787	0.132149	0.006561			
		925	0.131378	0.007044			
		1063	0.130314	0.008515			
		1201	0.127759	0.00918			
		1339	0.12707	0.010187			
		1477	0.125687	0.0115			
		1615	0.12528	0.012775			
0.3	0.3	0	0	0	1.19	3.97	
		407	0.131539	0.004904			
		545	0.128885	0.006229			
		683	0.126061	0.008626			
		821	0.125771	0.009684			
		959	0.123746	0.011433			
		1097	0.121926	0.013177			
		1235	0.119761	0.01475			
		1373	0.119515	0.016273			
		1511	0.117787	0.017862			
		1649	0.116353	0.01935			
0.3	0.4	0	0	0	1.59	3.98	
		353	0.13326	0.005662			
		491	0.131371	0.007971			
		629	0.126729	0.01028			
		767	0.126074	0.012613			
		905	0.122779	0.014714			
		1043	0.122671	0.016867			
		1181	0.119954	0.019058			
		1319	0.118734	0.020722			
		1457	0.117215	0.022888			

		1595	0.115494	0.025047			

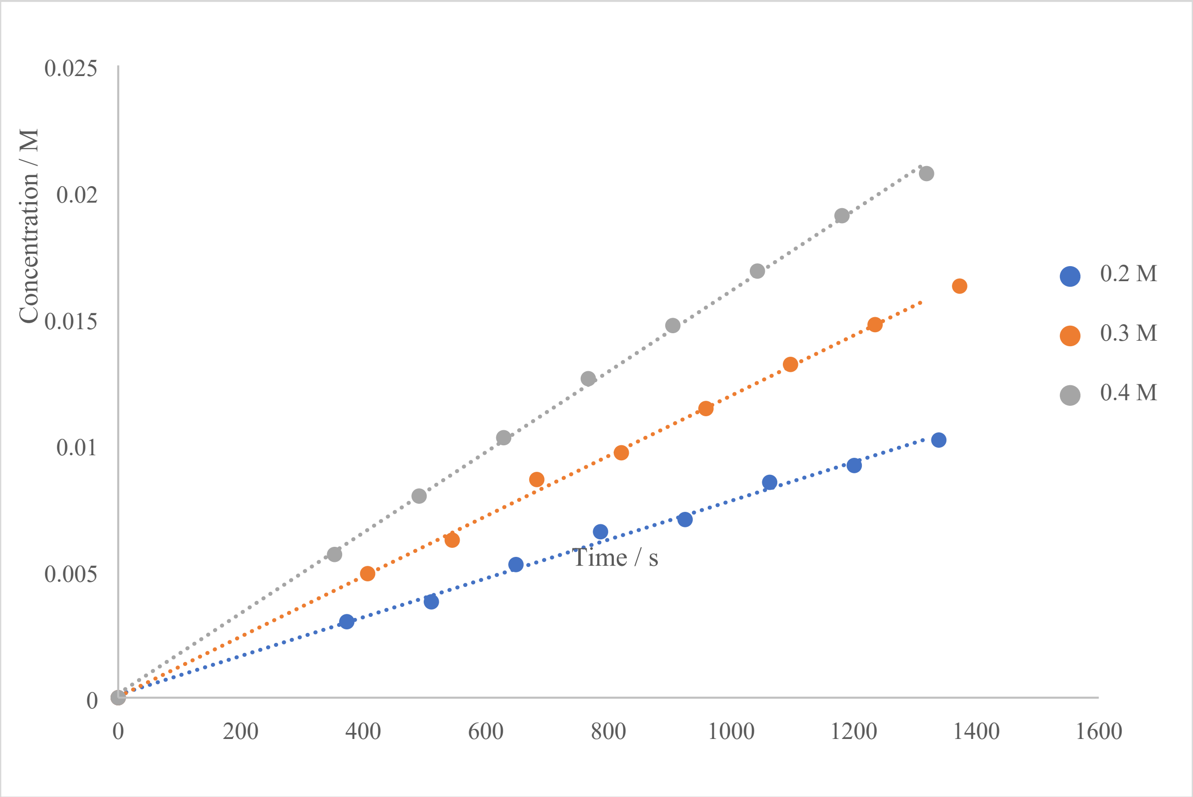

Figure S62. Plots of Stetter product concentration against time for the C_6F_5 triazolium precatalyst **16** (5 mM) catalysed Stetter reaction, at initial Michael acceptor **77** concentrations 0.2 M, 0.3 M, 0.4 M.

Entry 30

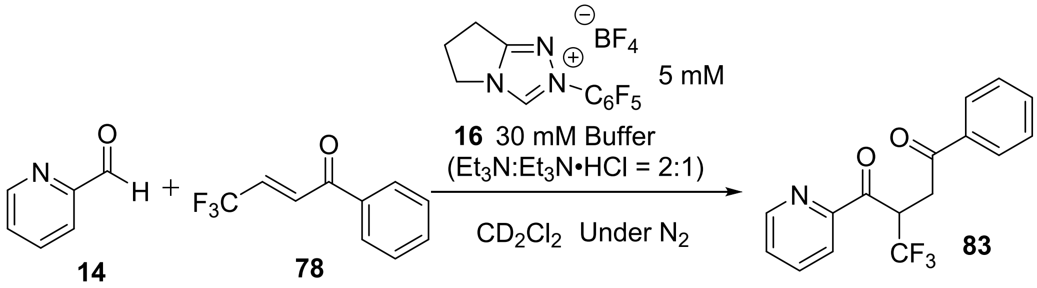

The reaction of pyridine-2-carboxaldehyde **14** and Michael acceptor **78** catalysed by C_6F_5 triazolium salt **16** in triethylamine buffer was monitored using ^{1}H NMR spectra, with representative NMR spectra over the course of the experiment given in Figure S63.

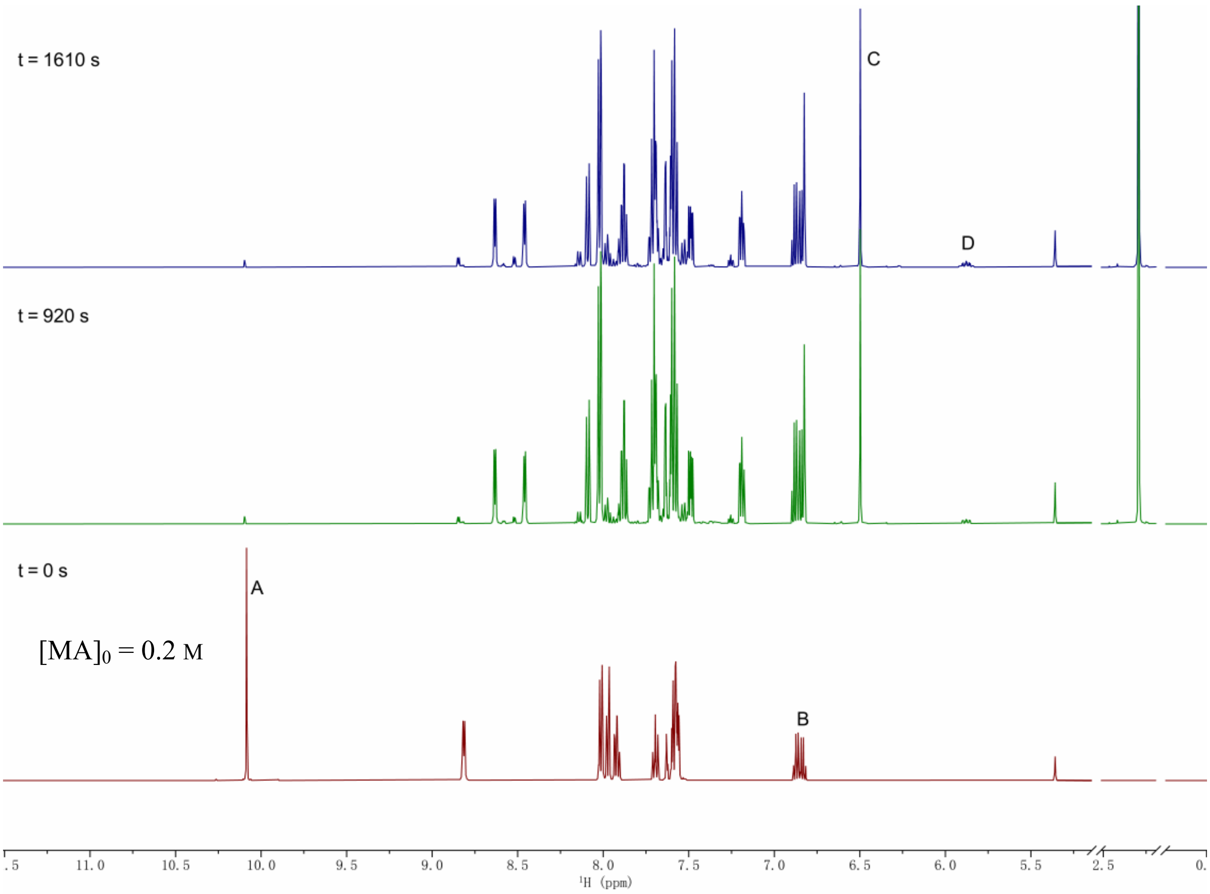
t = 1610 s
C
D
t = 920 s
t = 0 s
A
[MA]$_0$ = 0.2 M
B
.5 11.0 10.5 10.0 9.5 9.0 8.5 8.0 7.5 7.0 6.5 6.0 5.5 2.5 0.
^{1}H (ppm)

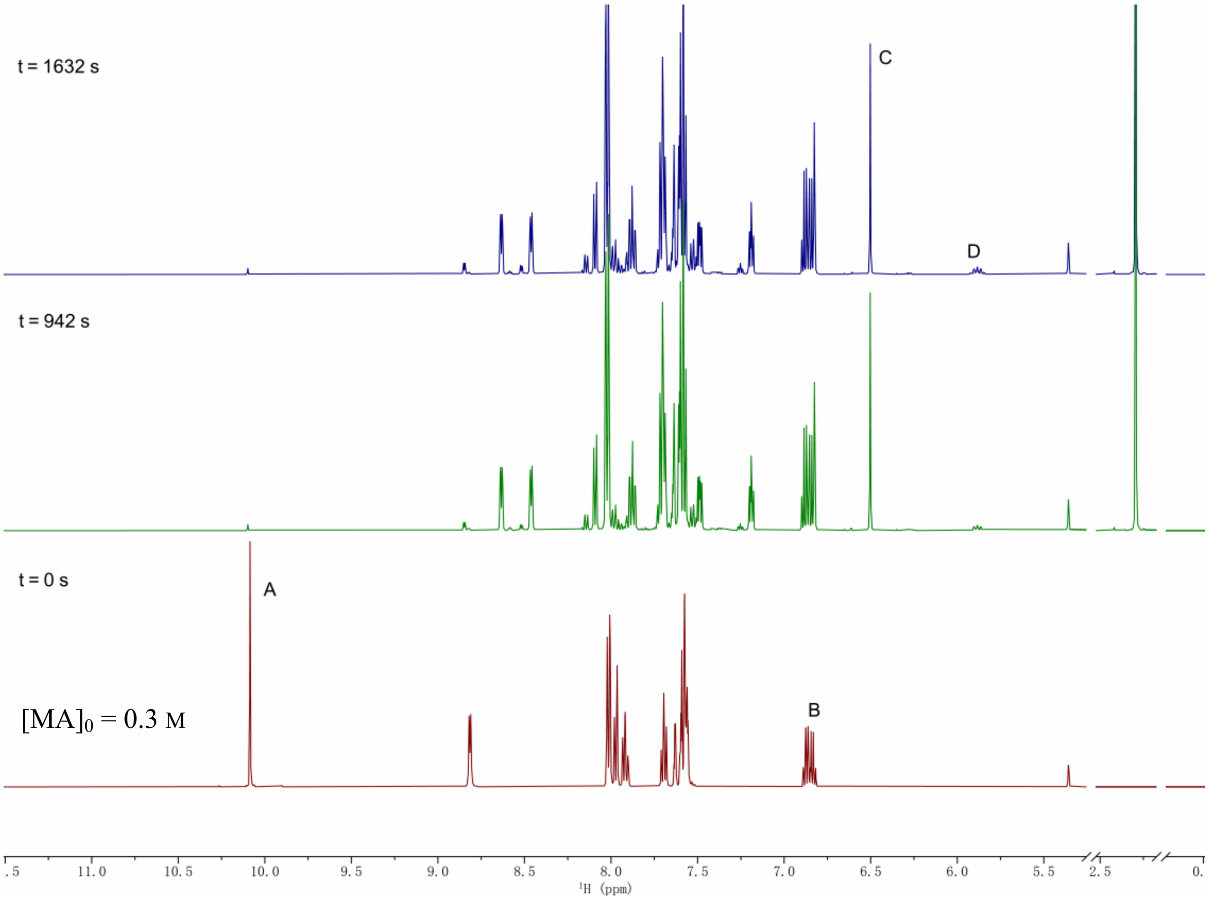
t = 1632 s
C
D
t = 942 s
t = 0 s
A
[MA]$_0$ = 0.3 M
B
.5 11.0 10.5 10.0 9.5 9.0 8.5 8.0 7.5 7.0 6.5 6.0 5.5 2.5 0.
^{1}H (ppm)

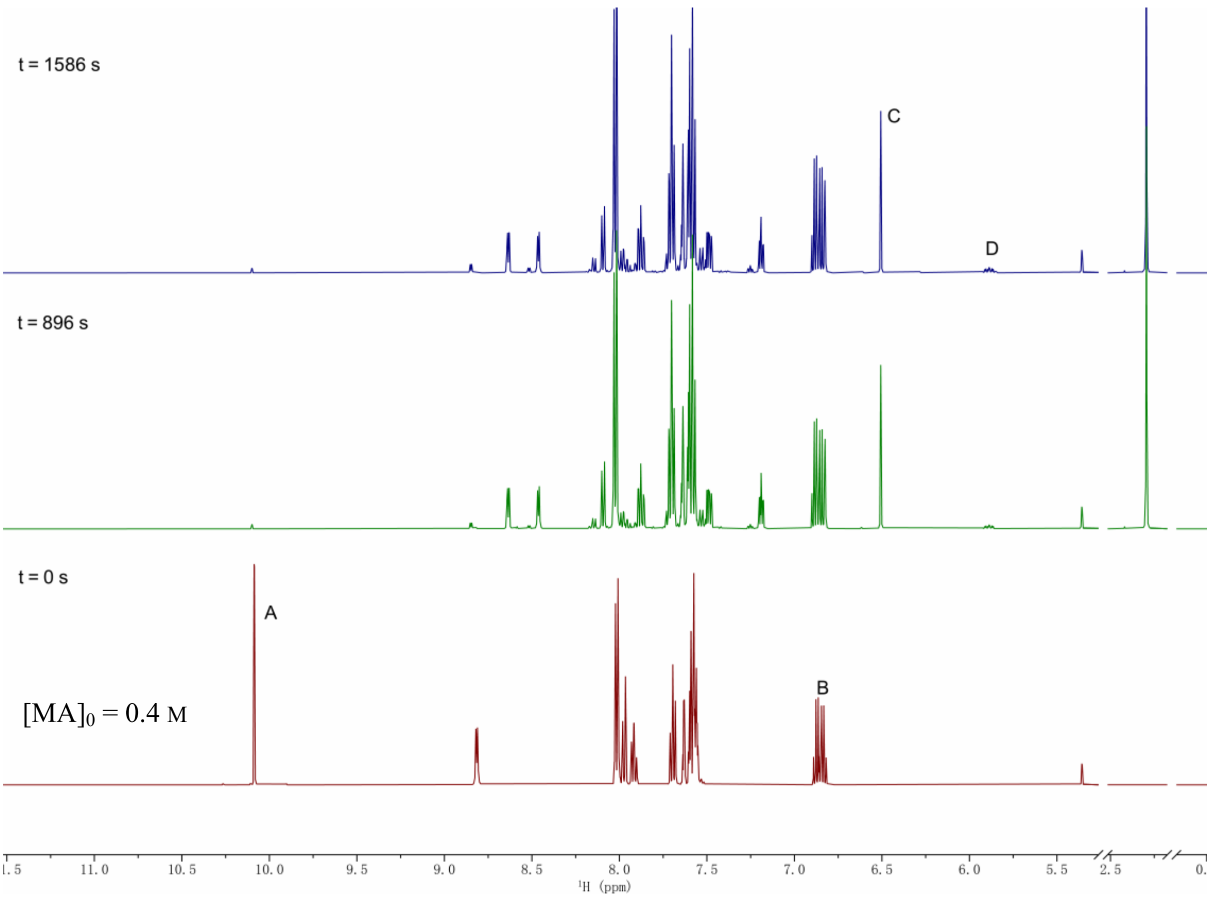

Figure S63. Representative ^{1}H NMR spectra (500 MHz) for reaction of pyridine-2-carboxaldehyde **14** (0.3 M) and Michael acceptor **78** (0.2 M, 0.3 M and 0.4 M) catalysed by *N*-C_6F_5 NHC precursor **16** (5 mM) in NEt_3:NEt_3·HCl (2:1, 0.03 M) in CD_2Cl_2 at 25 °C. A = Aldehyde C*H*O, B = Michael acceptor C*H*, C = Benzoin C*H*, D = Stetter product C*H*.

Table S30. Reaction data and initial rates of Stetter product formation from Michael acceptor **78**, catalyzed by C_6F_5 triazolium precatalyst **16** (5 mM) in 0.03 M triethylamine buffer (Et_3N:Et_3N·HCl, 2:1) in CD_2Cl_2 at 25 °C.

**[Aldehyde]$_0$ / M**	**[MA]$_0$ / M**	**Time / s**	**[Benzoin] / M**	**[Stetter] / M**	**v_{max} / 10^{-5} M s^{-1}**	**k'_S / 10^{-5} s^{-1}**	**Average / 10^{-5} s^{-1}**
0.3	0.2	0	0	0	1.21	6.05	6.18±0.13
		368	0.135039	0.004453			
		506	0.132425	0.00629			
		644	0.130558	0.008106			
		782	0.129039	0.009833			
		920	0.127585	0.011299			
		1058	0.126062	0.01252			
		1196	0.124891	0.013519			
		1334	0.123662	0.015061			
		1472	0.122512	0.016173			

		1610	0.121531	0.017295			
		0	0	0			
		390	0.132453	0.007751			
		528	0.129886	0.011149			
		666	0.128117	0.014008			
		804	0.126196	0.015984			
0.3	0.3	942	0.124285	0.017483	1.84	6.13	
		1080	0.123381	0.01985			
		1218	0.121728	0.020641			
		1356	0.120221	0.021859			
		1494	0.119256	0.023338			
		1632	0.118098	0.024379			
		0	0	0			
		344	0.126526	0.010592			
		482	0.123752	0.014483			
		620	0.121723	0.017639			
		758	0.119991	0.020317			
0.3	0.4	896	0.118448	0.022605	2.54	6.35	
		1034	0.117051	0.024678			
		1172	0.116021	0.02649			
		1310	0.114632	0.027418			
		1448	0.113674	0.028825			
		1586	0.112653	0.030299			

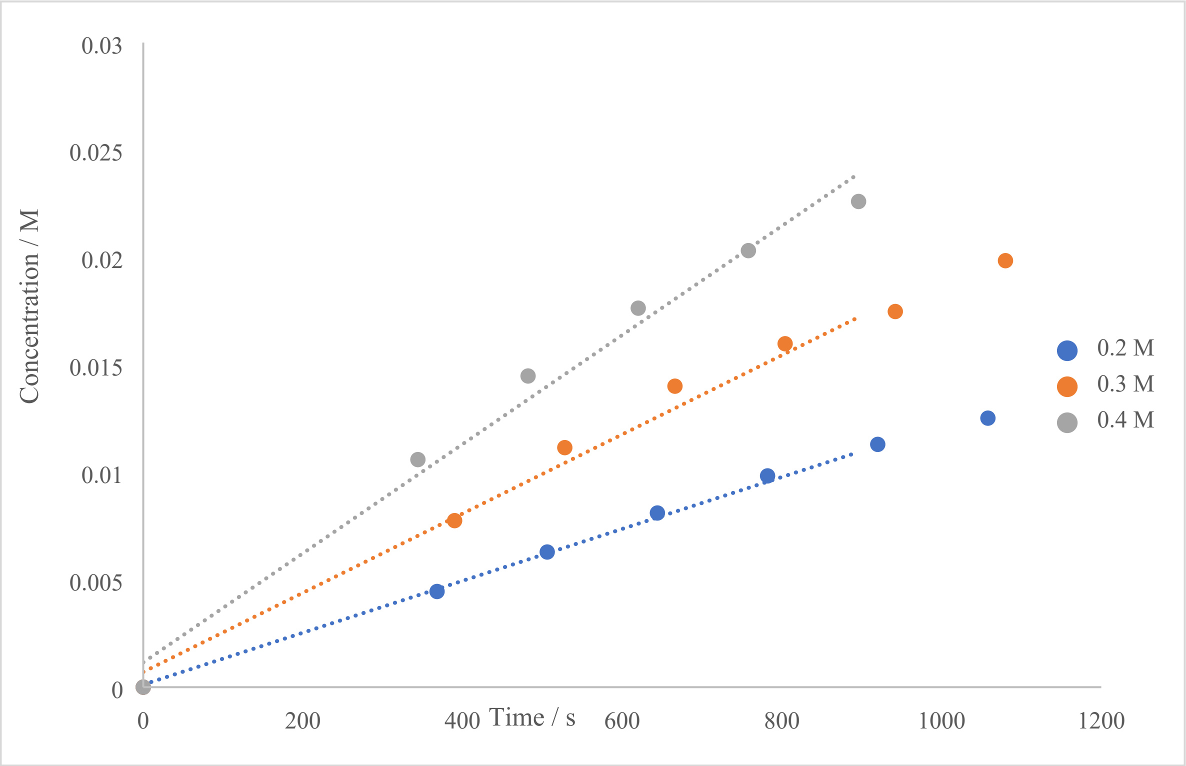

Figure S64. Plots of Stetter product concentration against time for the C_6F_5 triazolium precatalyst **16** (5 mM) catalysed Stetter reaction, at initial Michael acceptor **78** concentrations 0.2 M, 0.3 M, 0.4 M.

Entry 31

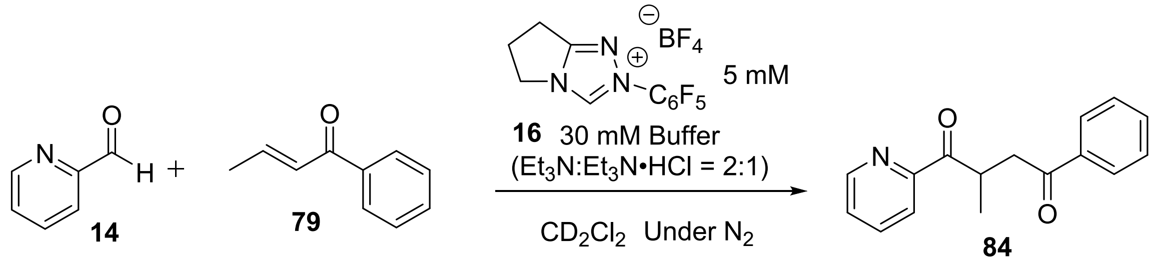

The reaction of pyridine-2-carboxaldehyde **14** and Michael acceptor **79** catalysed by C_6F_5 triazolium salt **16** in triethylamine buffer was monitored using ^{1}H NMR spectra, with representative NMR spectra over the course of the experiment given in Figure S65.

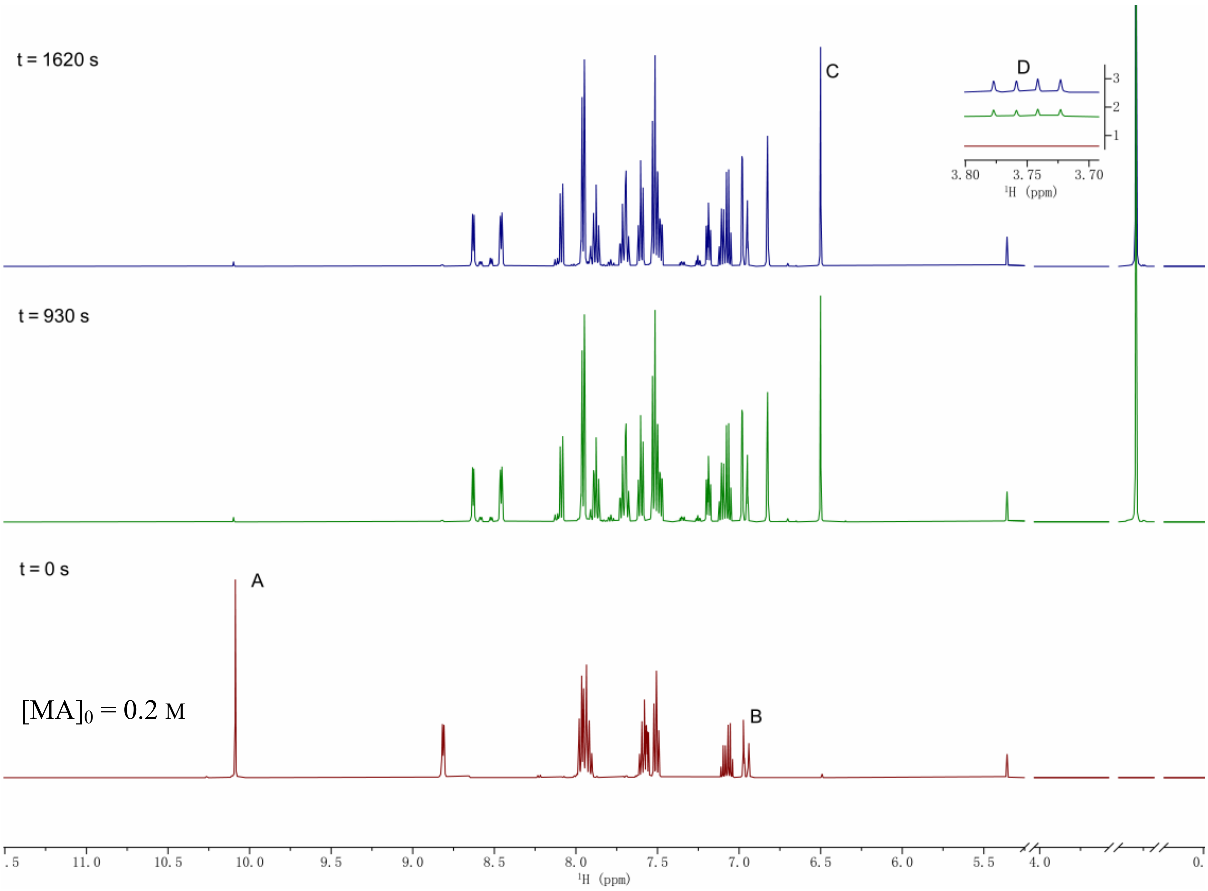

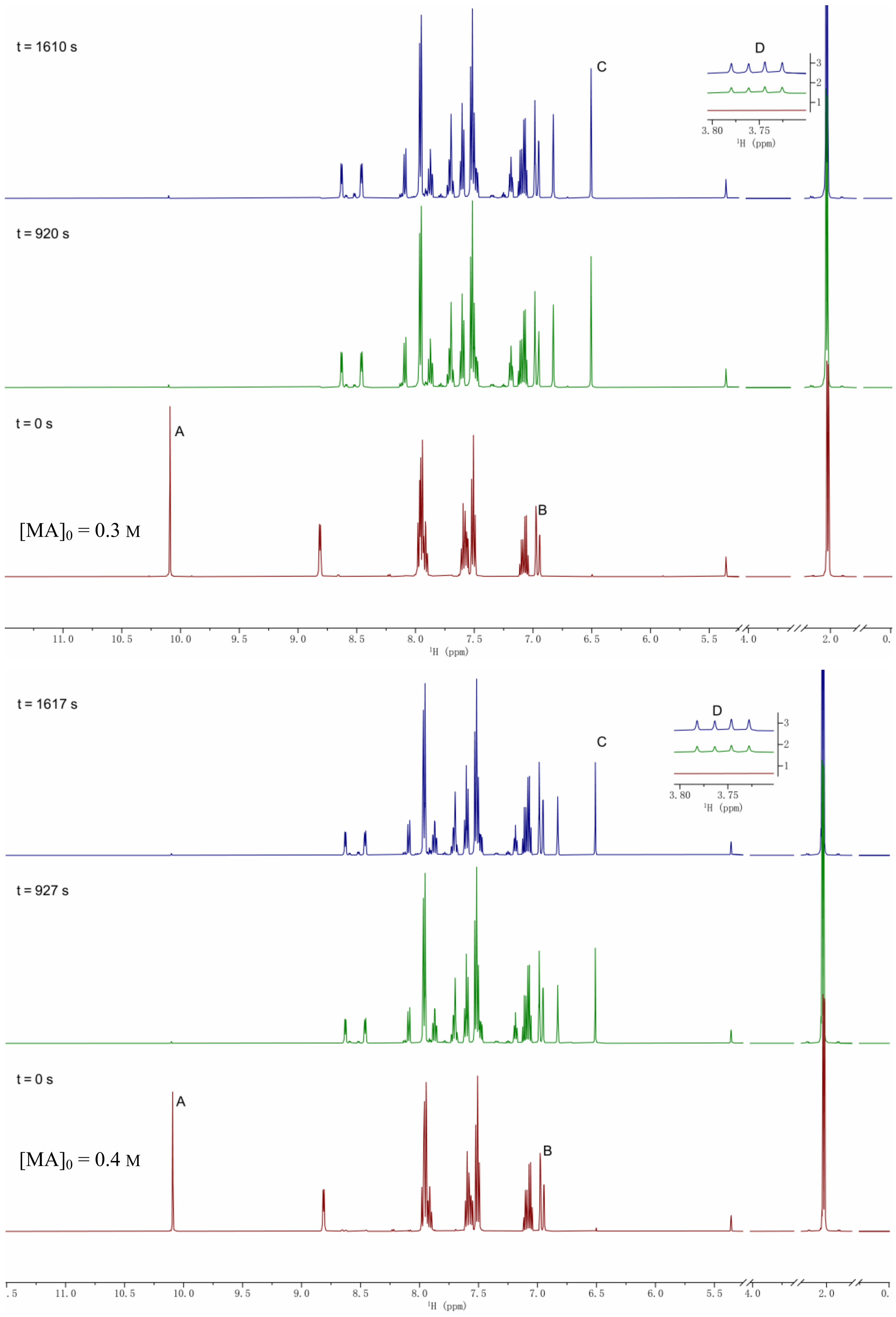

Figure S65. Representative ^{1}H NMR spectra (500 MHz) for reaction of pyridine-2-carboxaldehyde **14** (0.3

M) and Michael acceptor **79** (0.2 M, 0.3 M and 0.4 M) catalysed by *N*-C_6F_5 NHC precursor **16** (5 mM) in NEt_3:NEt_3·HCl (2:1, 0.03 M) in CD_2Cl_2 at 25 °C. A = Aldehyde C*H*O, B = Michael acceptor Ar*H*, C = Benzoin C*H*, D1 = Stetter product C*H*, D2 = Stetter product CH_AH_B.

Table S31. Reaction data and initial rates of Stetter product formation from Michael acceptor **79**, catalyzed by C_6F_5 triazolium precatalyst **16** (5 mM) in 0.03 M triethylamine buffer (Et_3N:Et_3N·HCl, 2:1) in CD_2Cl_2 at 25 °C.

**[Aldehyde]$_0$ / M**	**[MA]$_0$ / M**	**Time / s**	**[Benzoin] / M**	**[Stetter] / M**	v_{max} **/ 10^{-5} M s^{-1}**	k_s' **/ 10^{-5} s^{-1}**	**Average / 10^{-5} s^{-1}**
0.3	0.2	0	0	0	0.103	0.52	0.55±0.02
		378	0.141118	0.000292			
		516	0.143876	0.000401			
		654	0.142894	0.000541			
		792	0.14204	0.000734			
		930	0.141327	0.000942			
		1068	0.140518	0.00107			
		1206	0.139523	0.001137			
		1344	0.138619	0.001332			
		1482	0.137805	0.001678			
		1620	0.136943	0.001841			
0.3	0.3	0	0	0	0.170	0.57	
		368	0.14222	0.000459			
		506	0.144683	0.000712			
		644	0.143847	0.001013			
		782	0.142587	0.001257			
		920	0.141645	0.001479			
		1058	0.140827	0.001668			
		1196	0.139853	0.001932			
		1334	0.138783	0.002246			
		1472	0.137832	0.002364			
		1610	0.137207	0.002642			
0.3	0.4	0	0	0	0.57	3.90	
		375	0.140348	0.000681			
		513	0.142958	0.001027			
		651	0.141882	0.001436			
		789	0.141113	0.001722			
		927	0.140177	0.002047			
		1065	0.139036	0.002311			
		1203	0.138096	0.002715			
		1341	0.136944	0.003007			
		1479	0.136004	0.003257			
		1617	0.135119	0.003651			

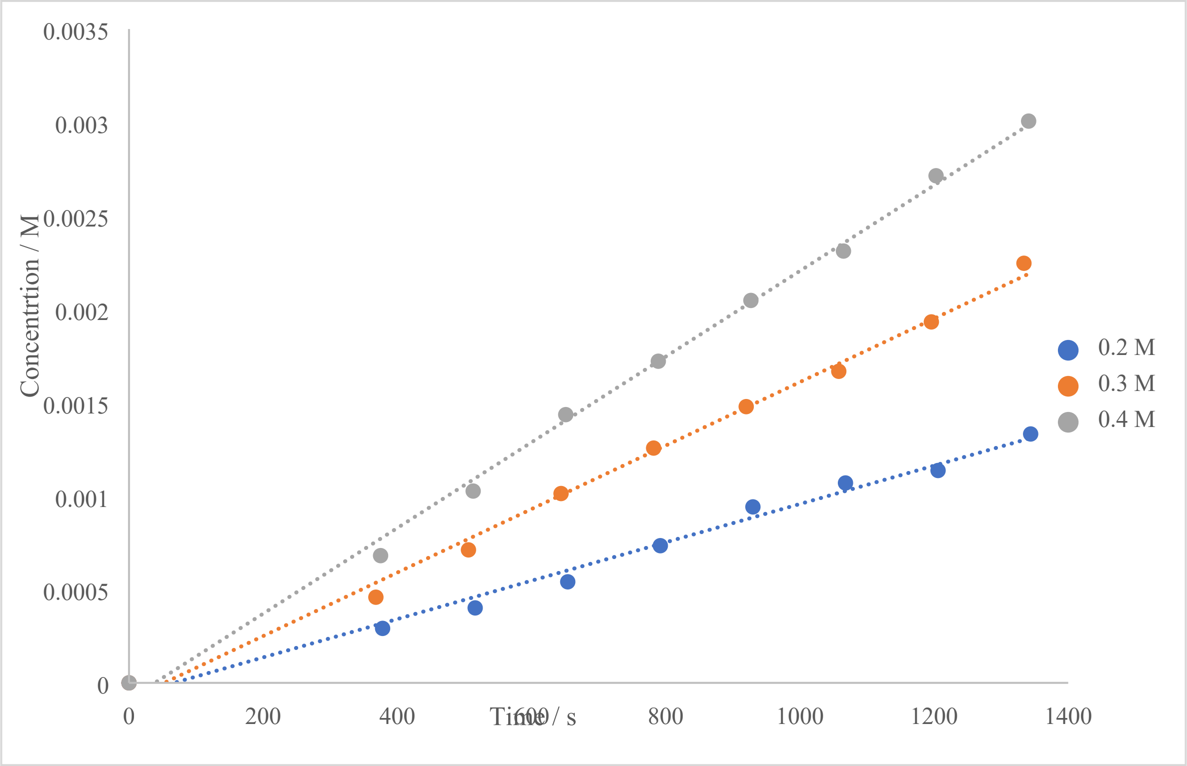

Figure S66. Plots of Stetter product concentration against time for the C_6F_5 triazolium precatalyst **16** (5 mM) catalysed Stetter reaction, at initial Michael acceptor **79** concentrations 0.2 M, 0.3 M, 0.4 M.

Entry 32

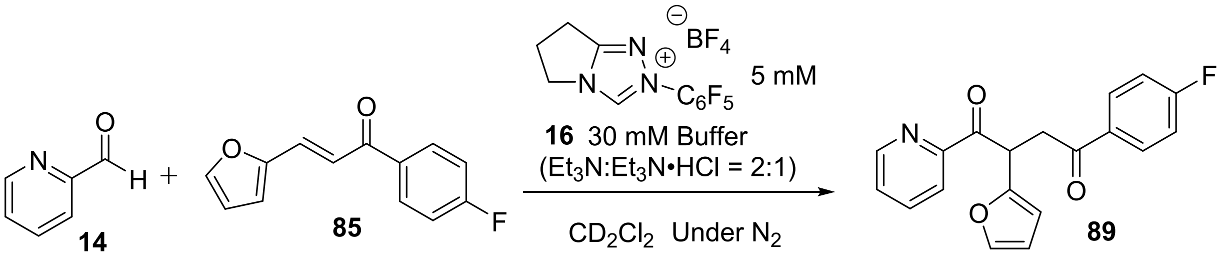

The reaction of pyridine-2-carboxaldehyde **14** and Michael acceptor **85** catalysed by C_6F_5 triazolium salt **16** in triethylamine buffer was monitored using ^{1}H NMR spectra, with representative NMR spectra over the course of the experiment given in Figure S67.

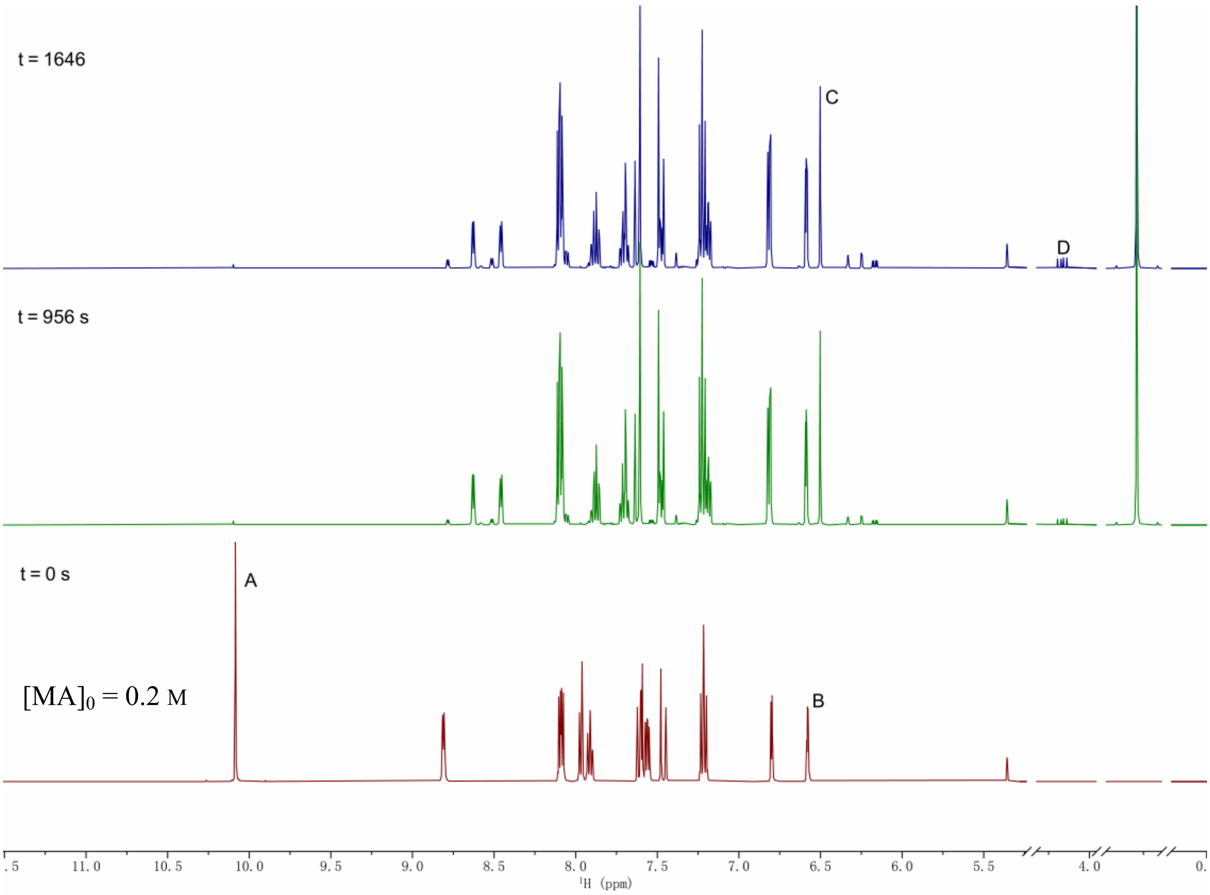
t = 1646
C
D
t = 956 s
t = 0 s
A
[MA]0 = 0.2 M
B
.5 11.0 10.5 10.0 9.5 9.0 8.5 8.0 7.5 7.0 6.5 6.0 5.5 4.0 0.
1H (ppm)

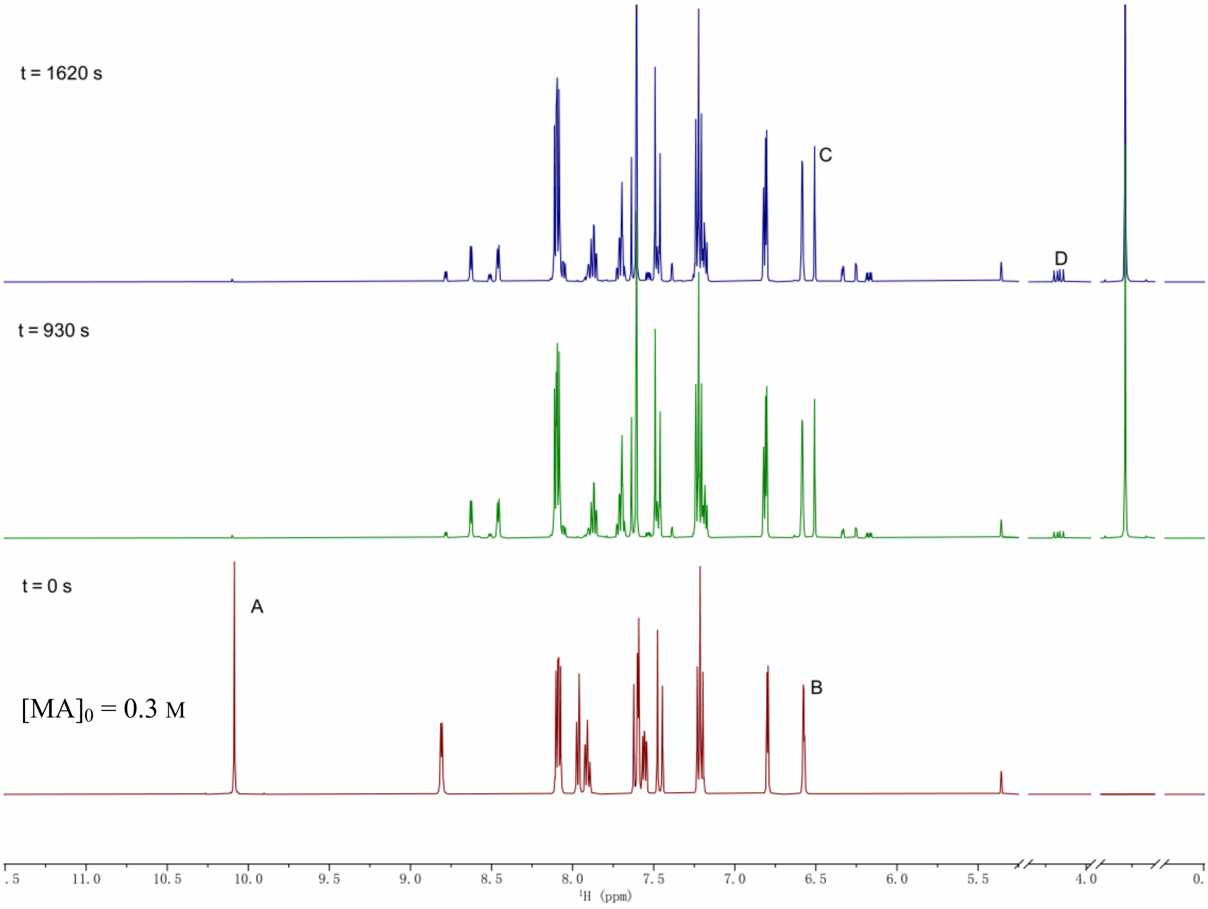
t = 1620 s
C
D
t = 930 s
t = 0 s
A
[MA]0 = 0.3 M
B
.5 11.0 10.5 10.0 9.5 9.0 8.5 8.0 7.5 7.0 6.5 6.0 5.5 4.0 0.
1H (ppm)

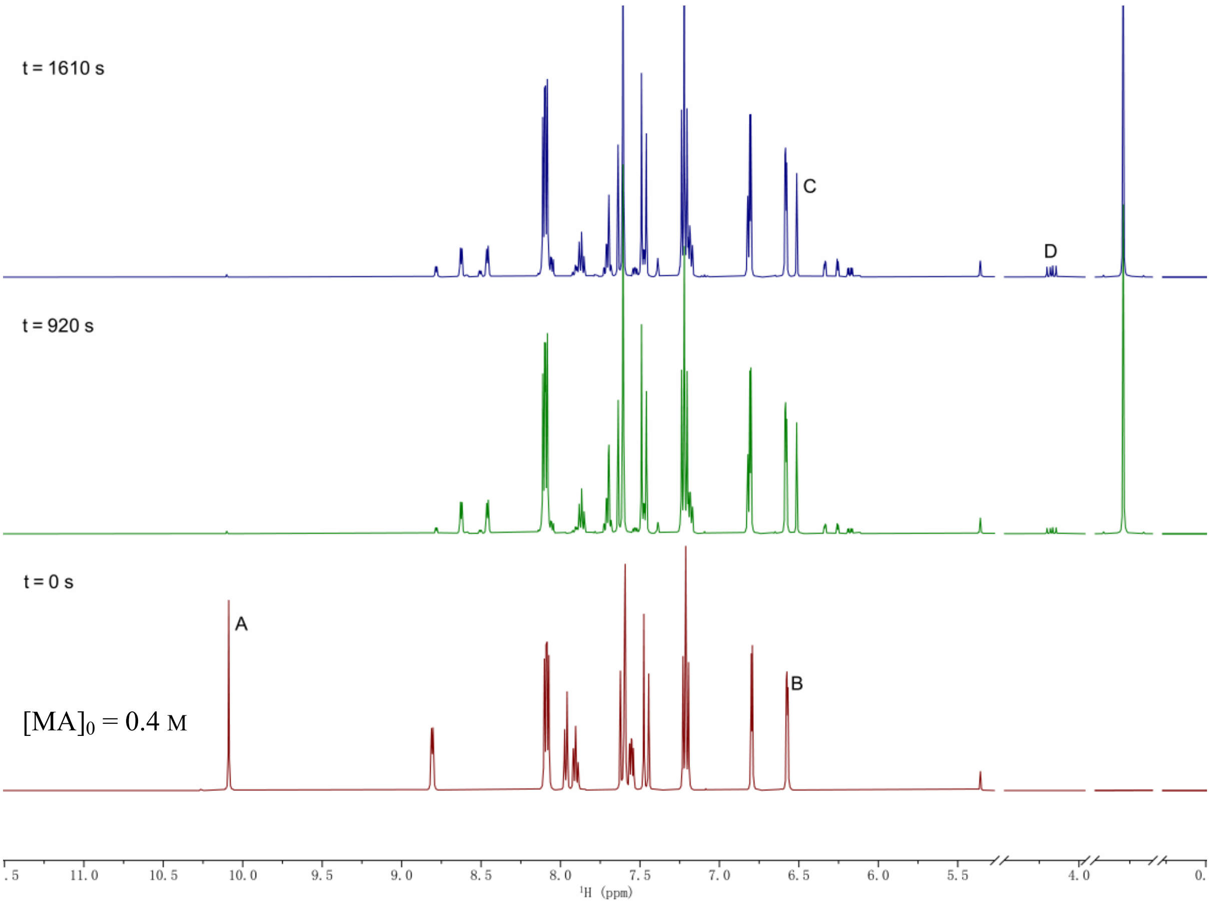

Figure S67. Representative ^{1}H NMR spectra (500 MHz) for reaction of pyridine-2-carboxaldehyde **14** (0.3 M) and Michael acceptor **85** (0.2 M, 0.3 M and 0.4 M) catalysed by *N*-C_6F_5 NHC precursor **16** (5 mM) in NEt_3:NEt_3·HCl (2:1, 0.03 M) in CD_2Cl_2 at 25 °C. A = Aldehyde C*H*O, B = Michael acceptor Ar*H*, C = Benzoin C*H*, D = Stetter product C*H*$_A$H$_B$.

Table S32. Reaction data and initial rates of Stetter product formation from Michael acceptor **85**, catalyzed by C_6F_5 triazolium precatalyst **16** (5 mM) in 0.03 M triethylamine buffer (Et_3N:Et_3N·HCl, 2:1) in CD_2Cl_2 at 25 °C.

[Aldehyde]$_0$ / M	[MA]$_0$ / M	Time / s	[Benzoin] / M	[Stetter] / M	v_{max} / 10^{-5} M s^{-1}	$k_s^{'}$ / 10^{-5} s^{-1}	Average / 10^{-5} s^{-1}
0.3	0.2	0	0	0	1.07	5.35	5.56±0.24
		404	0.133083	0.004224			
		542	0.130074	0.005771			
		680	0.128642	0.007268			
		818	0.126192	0.008822			
		956	0.124073	0.010275			
		1094	0.12207	0.01175			
		1232	0.120255	0.013104			
		1370	0.118696	0.014525			
		1508	0.116788	0.015601			
		1646	0.114911	0.017127			

0.3	0.3	0	0	0	1.77	5.90	
		378	0.131935	0.006302			
		516	0.127766	0.009203			
		654	0.125788	0.01165			
		792	0.123235	0.014365			
		930	0.120899	0.017017			
		1068	0.118042	0.019193			
		1206	0.115939	0.021174			
		1344	0.113209	0.023357			
		1482	0.111286	0.025545			
		1620	0.108986	0.027662			
0.3	0.4	0	0	0	2.17	5.16	
		368	0.128577	0.007247			
		506	0.124439	0.010585			
		644	0.123163	0.013743			
		782	0.120131	0.016942			
		920	0.116108	0.019963			
		1058	0.113245	0.022766			
		1196	0.11041	0.02573			
		1334	0.107792	0.028448			
		1472	0.106041	0.031041			
		1610	0.103465	0.033699			

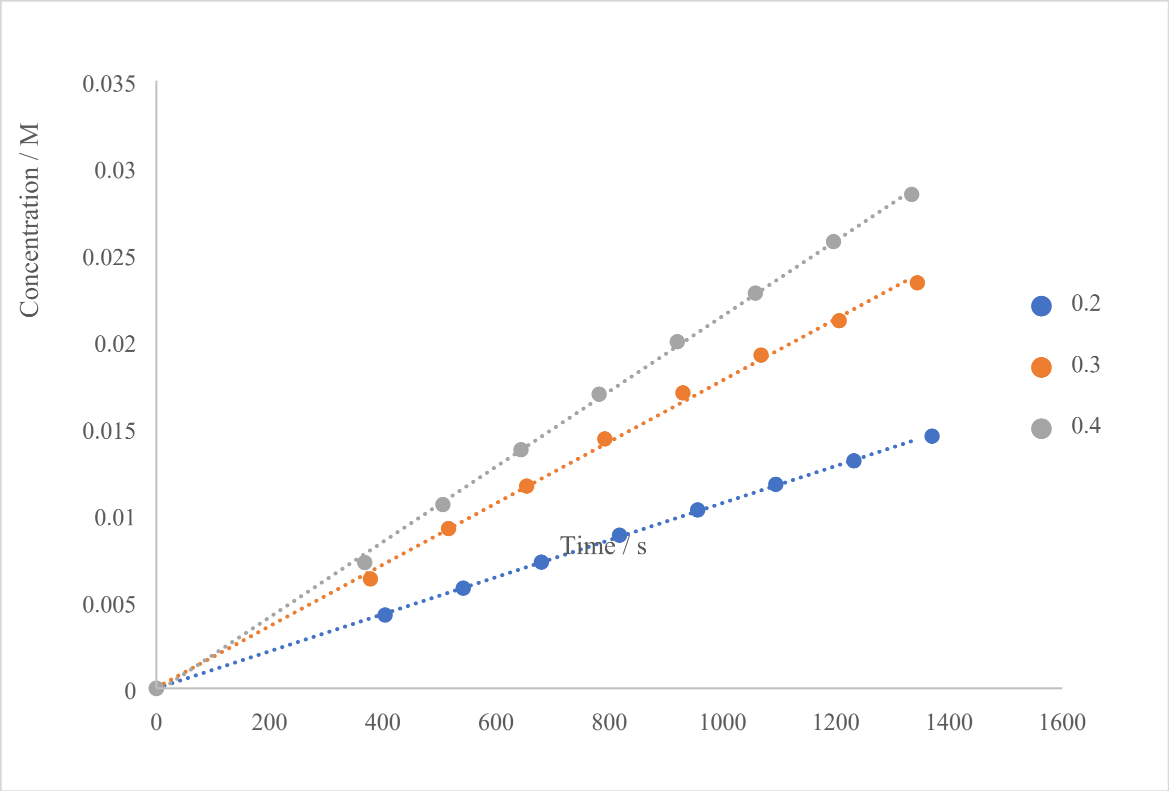

Figure S68. Plots of Stetter product concentration against time for the C_6F_5 triazolium precatalyst **16** (5 mM)

catalysed Stetter reaction, at initial Michael acceptor **85** concentrations 0.2 M, 0.3 M, 0.4 M.

Entry 33

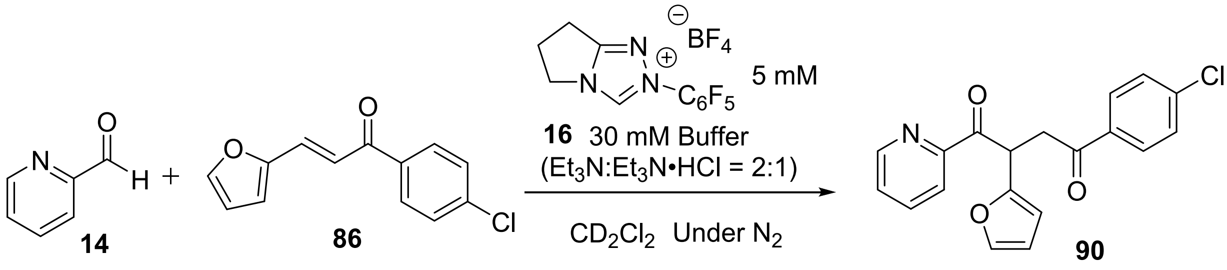

The reaction of pyridine-2-carboxaldehyde **14** and Michael acceptor **86** catalysed by C_6F_5 triazolium salt **16** in triethylamine buffer was monitored using ^1H NMR spectra, with representative NMR spectra over the course of the experiment given in Figure S69.

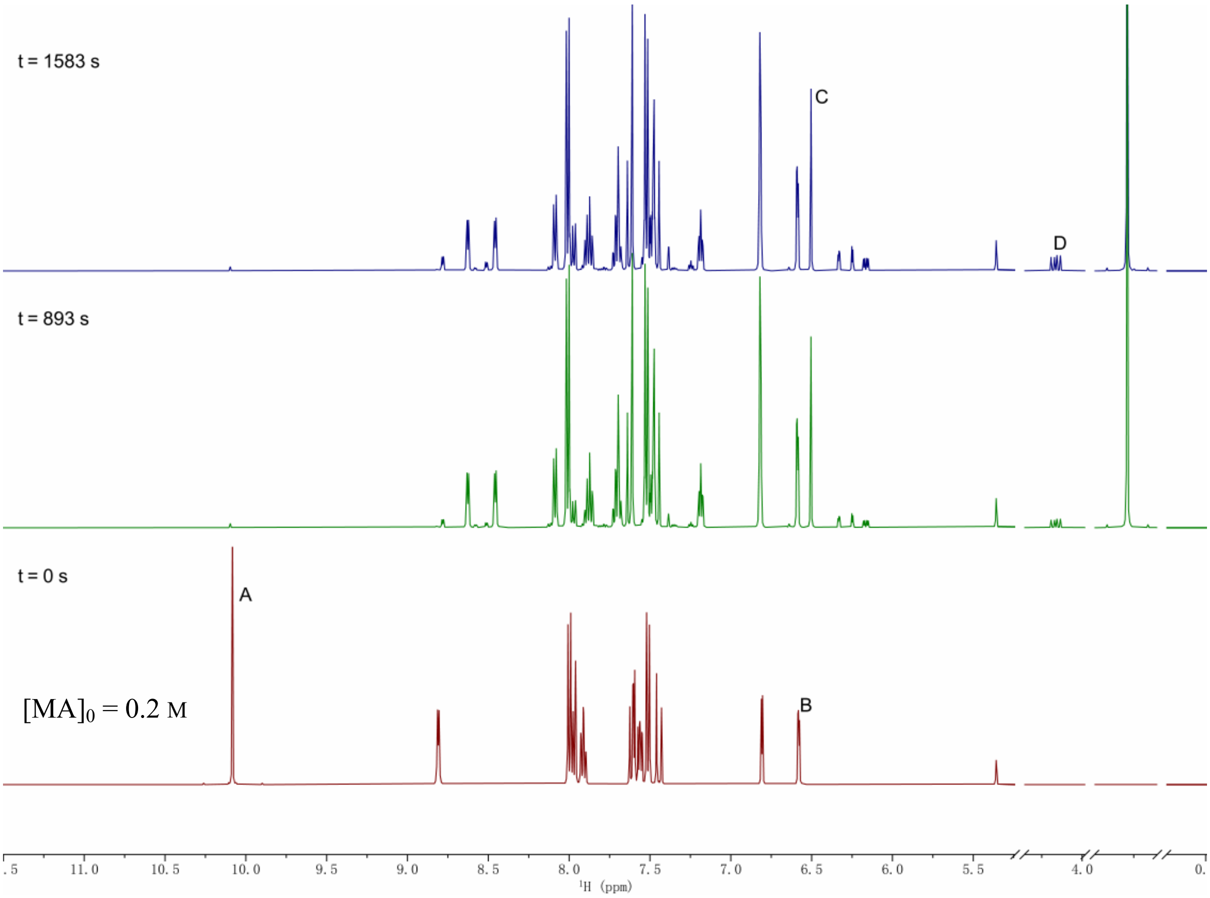

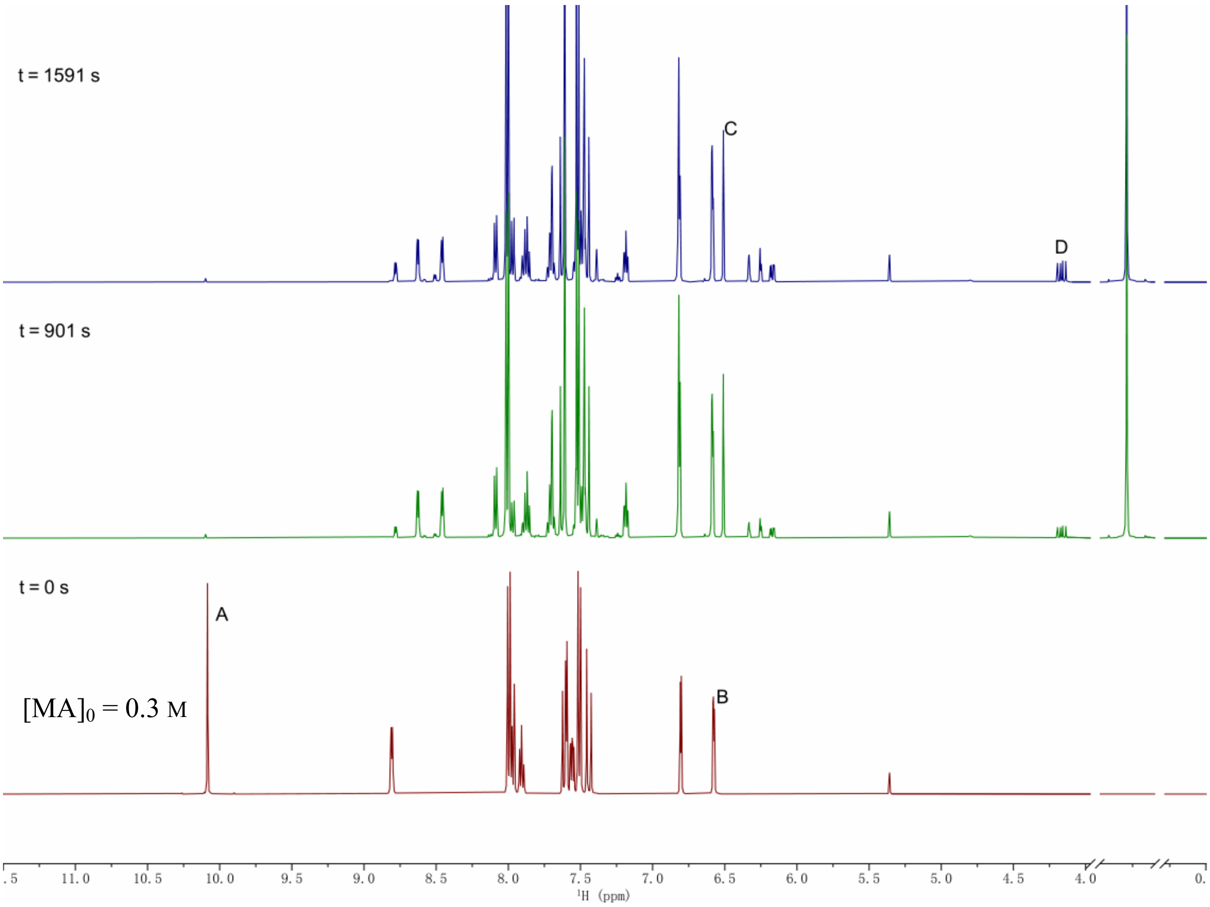
t = 1591 s
C
D
t = 901 s
t = 0 s
A
[MA]0 = 0.3 M
B
11.0
10.5
10.0
9.5
9.0
8.5
8.0
7.5
7.0
6.5
6.0
5.5
5.0
4.5
4.0
1H (ppm)

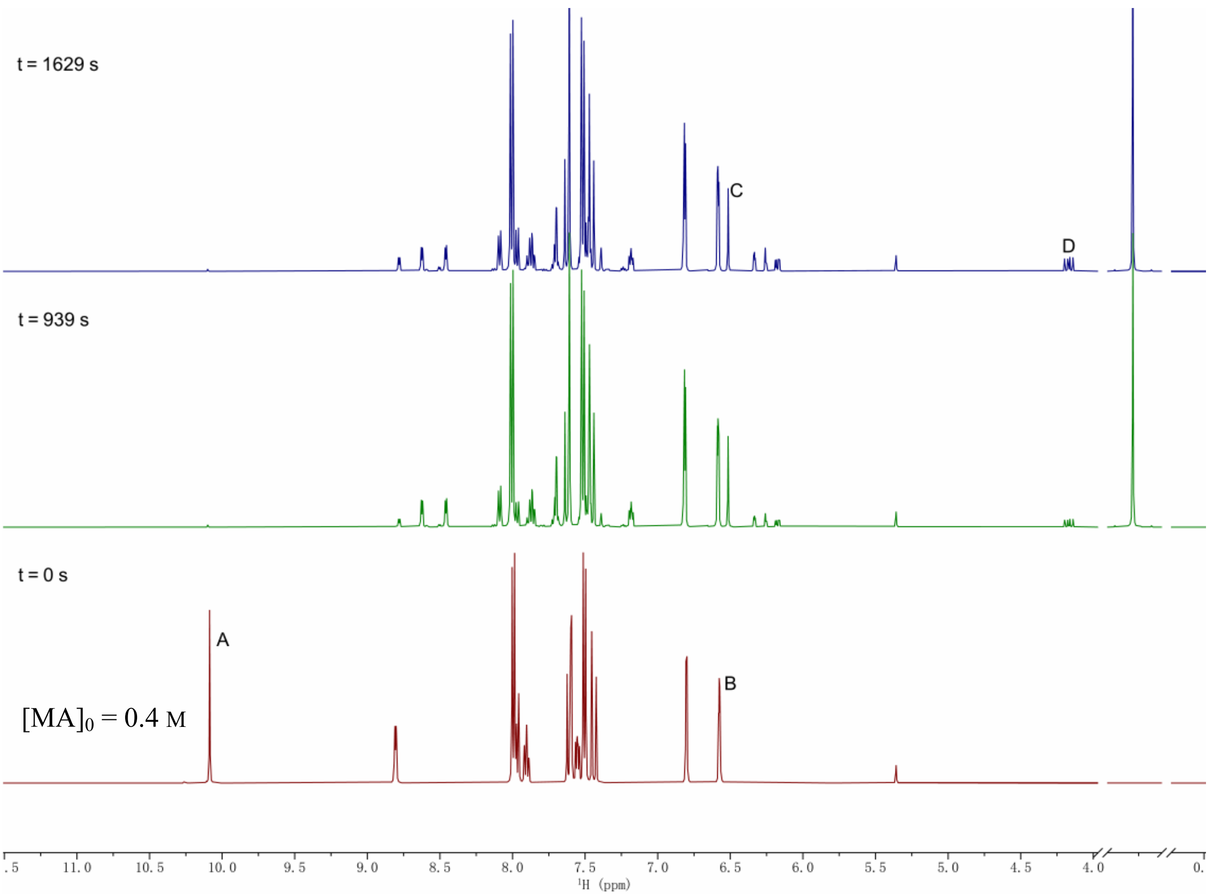
t = 1629 s
C
D
t = 939 s
t = 0 s
A
[MA]0 = 0.4 M
B
11.0
10.5
10.0
9.5
9.0
8.5
8.0
7.5
7.0
6.5
6.0
5.5
5.0
4.5
4.0
1H (ppm)

Figure S69. Representative ^{1}H NMR spectra (500 MHz) for reaction of pyridine-2-carboxaldehyde **14** (0.3 M) and Michael acceptor **86** (0.2 M, 0.3 M and 0.4 M) catalysed by *N*-C_6F_5 NHC precursor **16** (5 mM) in NEt_3:NEt_3·HCl (2:1, 0.03 M) in CD_2Cl_2 at 25 °C. A = Aldehyde C*H*O, B = Michael acceptor Ar*H*, C = Benzoin C*H*, D = Stetter product C$H_A$$H_B$.

Table S33. Reaction data and initial rates of Stetter product formation from Michael acceptor **86** , catalyzed by C_6F_5 triazolium precatalyst **16** (5 mM) in 0.03 M triethylamine buffer (Et_3N:Et_3N·HCl, 2:1) in CD_2Cl_2 at 25 °C.

**[Aldehyde]$_0$ / M**	**[MA]$_0$ / M**	**Time / s**	**[Benzoin] / M**	**[Stetter] / M**	v_{max} **/ 10^{-5} M s^{-1}**	k_s' **/ 10^{-5} s^{-1}**	**Average / 10^{-5} s^{-1}**
0.3	0.2	0	0	0	1.87	9.35	9.21±0.47
		341	0.130992	0.005169			
		479	0.128181	0.008193			
		617	0.125756	0.010892			
		755	0.123355	0.013914			
		893	0.121127	0.016317			
		1031	0.11888	0.018917			
		1169	0.116711	0.021491			
		1307	0.114758	0.023636			
		1445	0.113121	0.026242			
		1583	0.1107	0.02847			
0.3	0.3	0	0	0	2.91	9.70	
		349	0.130859	0.00855			
		487	0.127031	0.013463			
		625	0.124396	0.017554			
		763	0.120345	0.021717			
		901	0.117571	0.026066			
		1039	0.115089	0.029742			
		1177	0.112474	0.03361			
		1315	0.109714	0.037575			
		1453	0.106651	0.040933			
		1591	0.104466	0.044383			
0.3	0.4	0	0	0	3.16	8.58	
		387	0.128417	0.011675			
		525	0.124846	0.017045			
		663	0.120657	0.022193			
		801	0.116459	0.027244			
		939	0.113271	0.031711			
		1077	0.112969	0.036351			
		1215	0.107491	0.041076			
		1353	0.104947	0.045619			
		1491	0.101547	0.049641			

		1629	0.098708	0.054055			

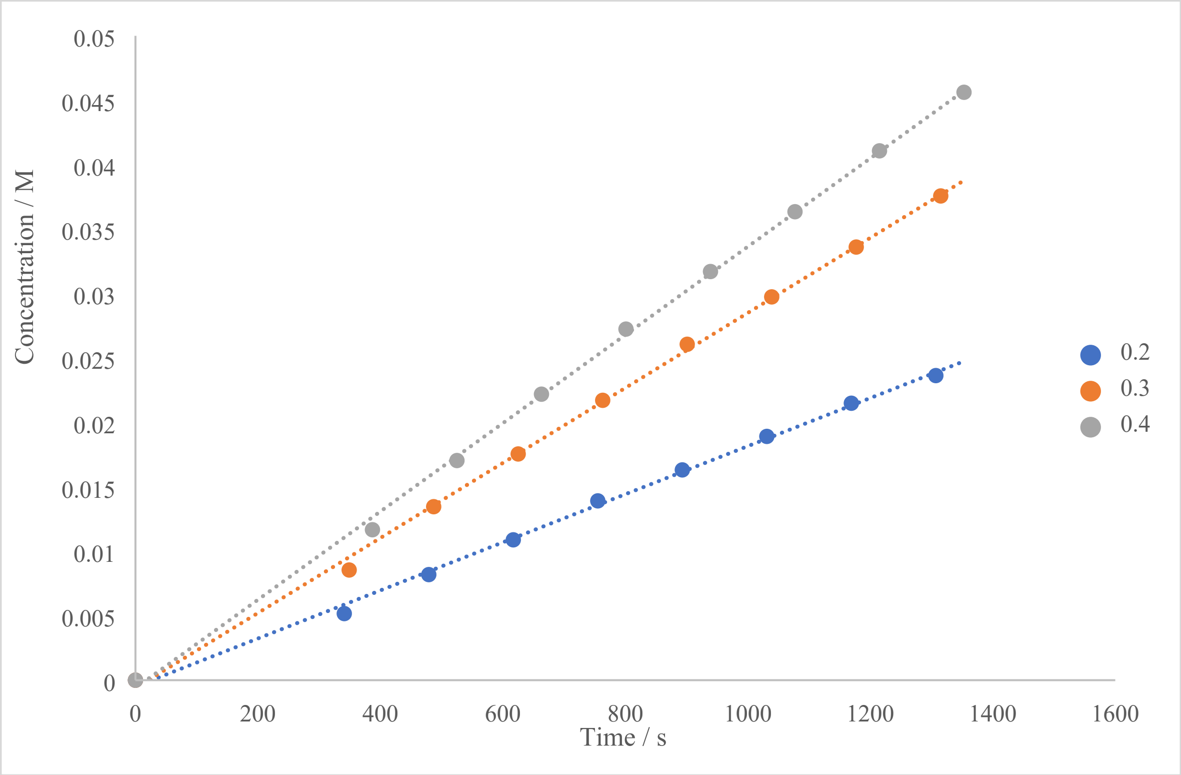

Figure S70. Plots of Stetter product concentration against time for the C_6F_5 triazolium precatalyst **16** (5 mM) catalysed Stetter reaction, at initial Michael acceptor **86** concentrations 0.2 M, 0.3 M, 0.4 M.

Entry 34

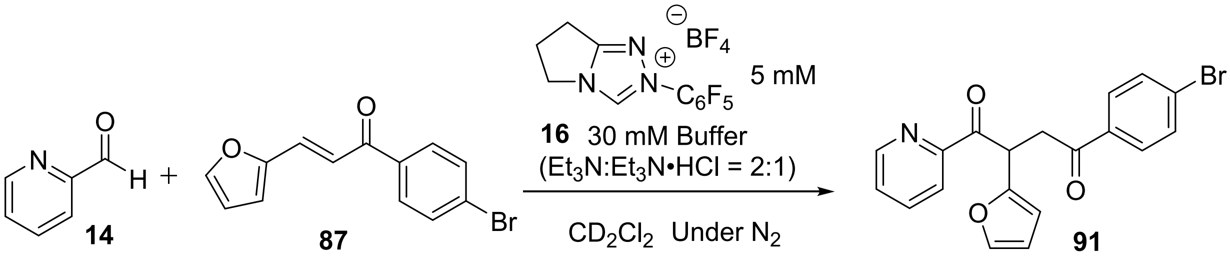

The reaction of pyridine-2-carboxaldehyde **14** and Michael acceptor **87** catalysed by C_6F_5 triazolium salt **16** in triethylamine buffer was monitored using ^{1}H NMR spectra, with representative NMR spectra over the course of the experiment given in Figure S71.

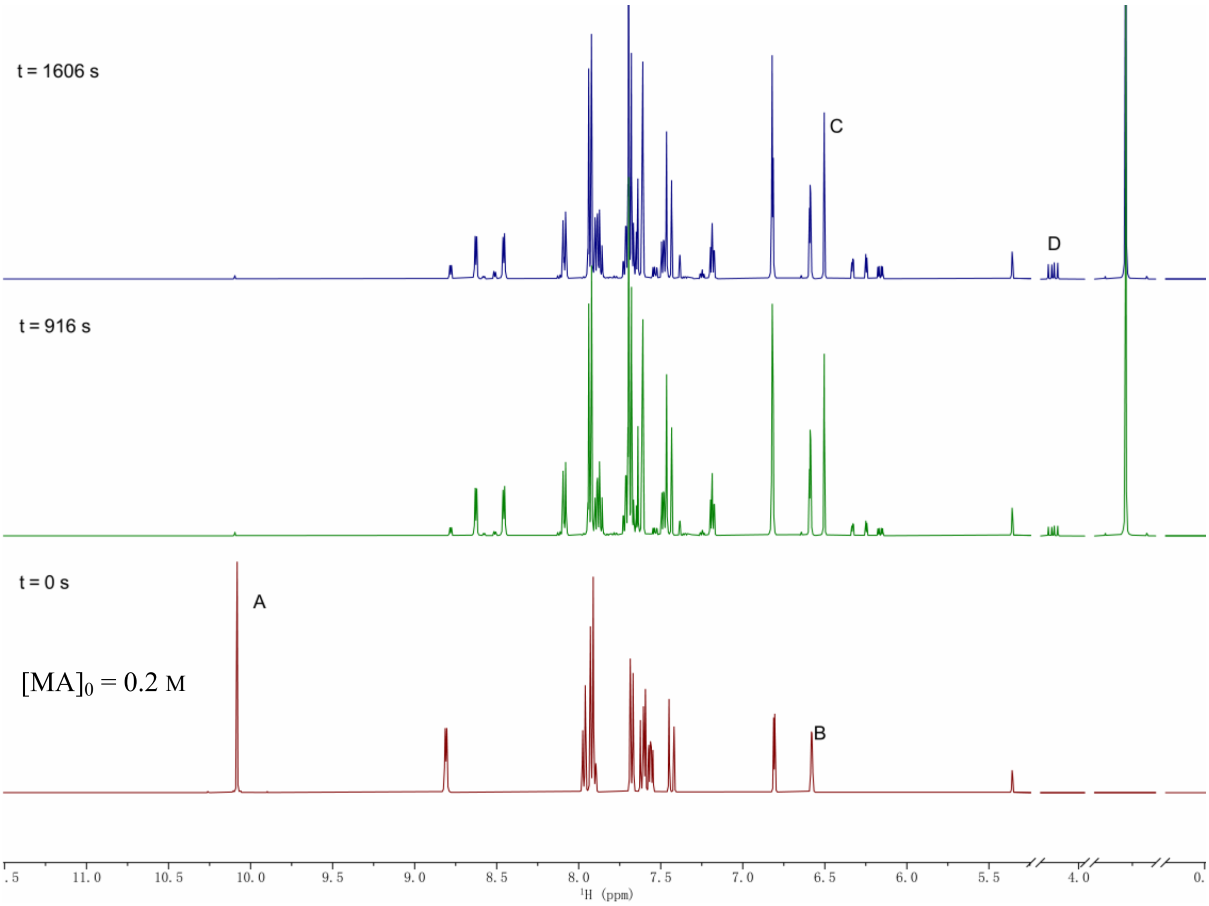
t = 1606 s
C
D
t = 916 s
t = 0 s
A
[MA]$_0$ = 0.2 M
B
.5
11.0
10.5
10.0
9.5
9.0
8.5
8.0
7.5
7.0
6.5
6.0
5.5
4.0
0.
^{1}H (ppm)

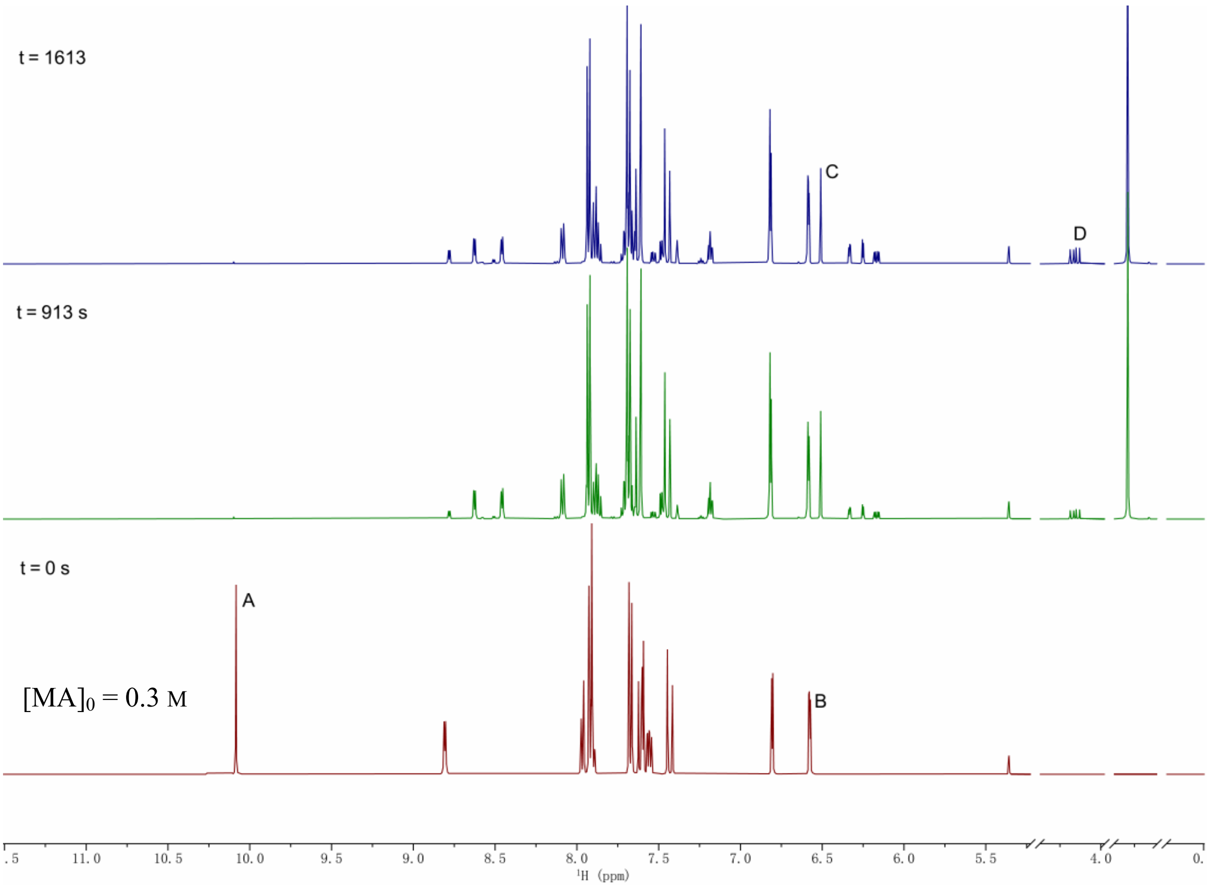
t = 1613
C
D
t = 913 s
t = 0 s
A
[MA]$_0$ = 0.3 M
B
.5
11.0
10.5
10.0
9.5
9.0
8.5
8.0
7.5
7.0
6.5
6.0
5.5
4.0
0.
^{1}H (ppm)

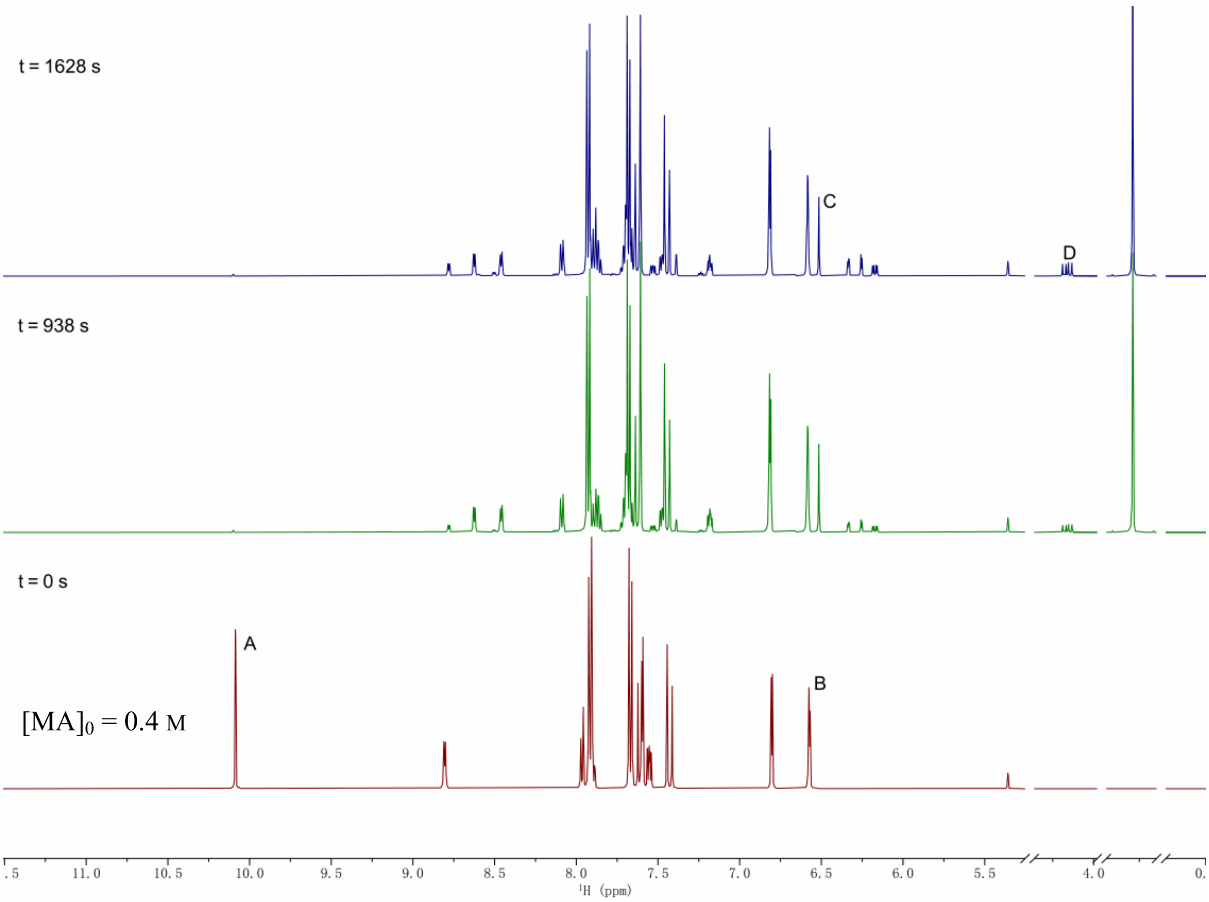

Figure S71. Representative ^{1}H NMR spectra (500 MHz) for reaction of pyridine-2-carboxaldehyde **14** (0.3 M) and Michael acceptor **87** (0.2 M, 0.3 M and 0.4 M) catalysed by *N*-C_6F_5 NHC precursor **16** (5 mM) in NEt_3:NEt_3·HCl (2:1, 0.03 M) in CD_2Cl_2 at 25 °C. A = Aldehyde C*H*O, B = Michael acceptor Ar*H*, C = Benzoin C*H*, D = Stetter product C$H_A$$H_B$.

Table S34. Reaction data and initial rates of Stetter product formation from Michael acceptor **87**, catalyzed by C_6F_5 triazolium precatalyst **16** (5 mM) in 0.03 M triethylamine buffer (Et_3N:Et_3N·HCl, 2:1) in CD_2Cl_2 at 25 °C.

**[Aldehyde]$_0$ / M**	**[MA]$_0$ / M**	**Time / s**	**[Benzoin] / M**	**[Stetter] / M**	v_{max} **/ 10^{-5} M s^{-1}**	k'_s **/ 10^{-5} s^{-1}**	**Average / 10^{-5} s^{-1}**
0.3	0.2	0	0	0	2.02	10.10	10.15±0.59
		364	0.126853	0.005549			
		502	0.123822	0.009155			
		640	0.121485	0.011944			
		778	0.119221	0.014827			
		916	0.116949	0.017712			
		1054	0.114329	0.020655			
		1192	0.112232	0.023476			
		1330	0.110097	0.026185			
		1468	0.10871	0.028726			
		1606	0.106802	0.031194			

		0	0	0			
		361	0.126013	0.009915			
		499	0.122477	0.01521			
		637	0.119548	0.019948			
		775	0.116259	0.024911			
0.3	0.3	913	0.112946	0.029285	3.27	10.90	
		1051	0.11015	0.033833			
		1189	0.10739	0.038163			
		1327	0.104495	0.042455			
		1465	0.101964	0.046438			
		1603	0.099329	0.050349			
		0	0	0			
		386	0.122418	0.01318			
		524	0.11782	0.018789			
		662	0.114626	0.023701			
		800	0.111322	0.030639			
0.3	0.4	938	0.107857	0.035082	3.78	9.45	
		1076	0.105052	0.04079			
		1214	0.098237	0.045285			
		1352	0.099854	0.050017			
		1490	0.09917	0.054145			
		1628	0.09455	0.058129			

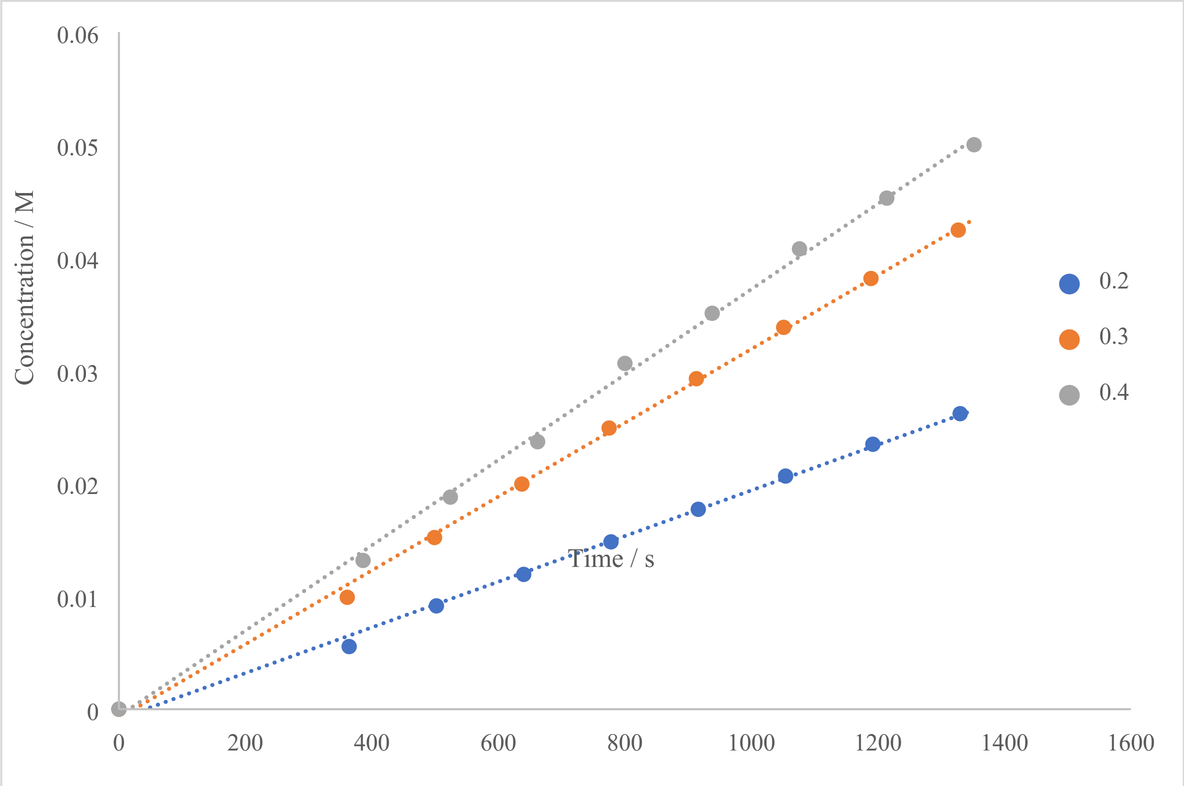

Figure S72. Plots of Stetter product concentration against time for the C_6F_5 triazolium precatalyst **16** (5 mM) catalysed Stetter reaction, at initial Michael acceptor **87** concentrations 0.2 M, 0.3 M, 0.4 M.

Entry 35

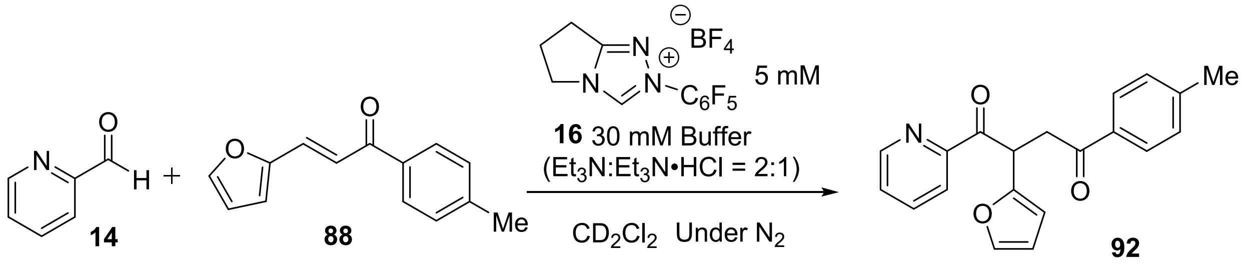

The reaction of pyridine-2-carboxaldehyde **14** and Michael acceptor **88** catalysed by C_6F_5 triazolium salt **16** in triethylamine buffer was monitored using ^{1}H NMR spectra, with representative NMR spectra over the course of the experiment given in Figure S73.

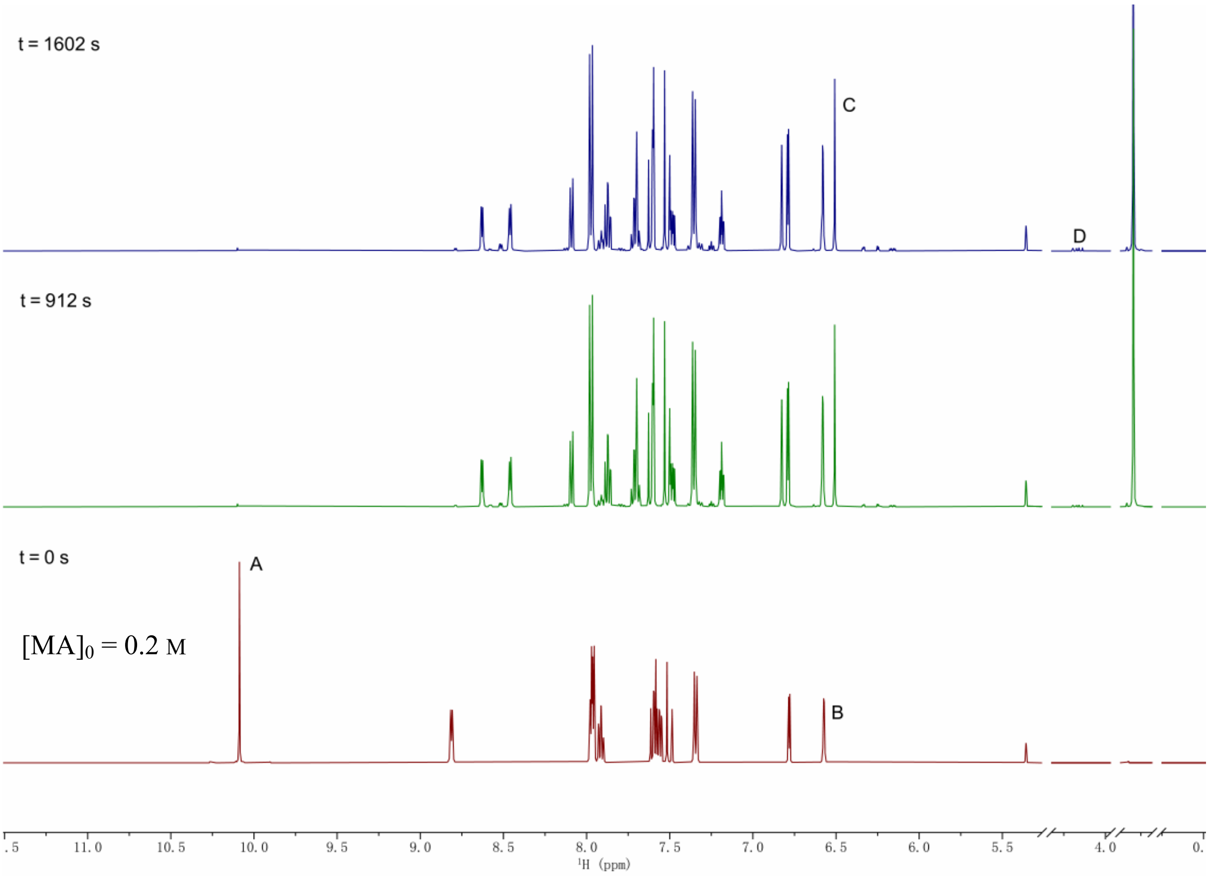

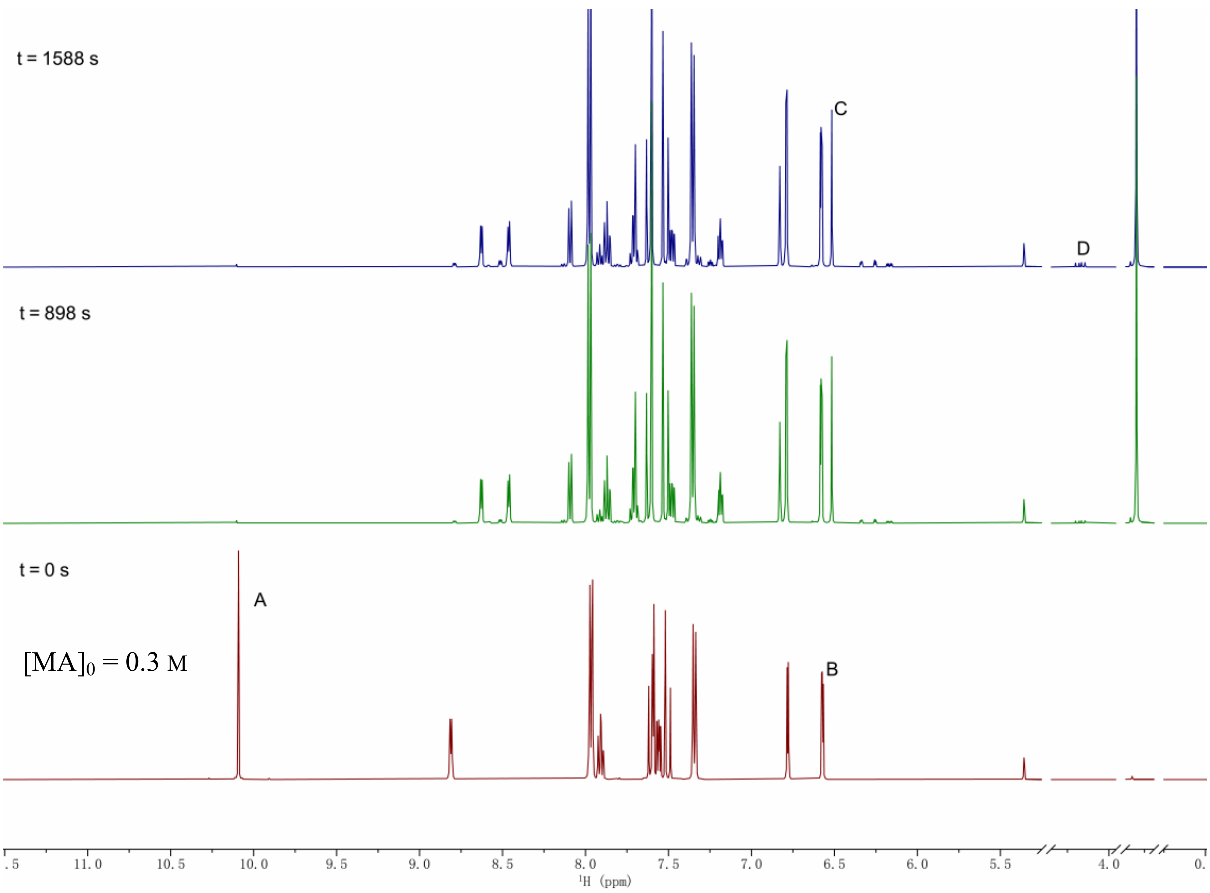
t = 1588 s
C
D
t = 898 s
t = 0 s
A
[MA]$_0$ = 0.3 M
B
.5 11.0 10.5 10.0 9.5 9.0 8.5 8.0 7.5 7.0 6.5 6.0 5.5 4.0 0.
^{1}H (ppm)

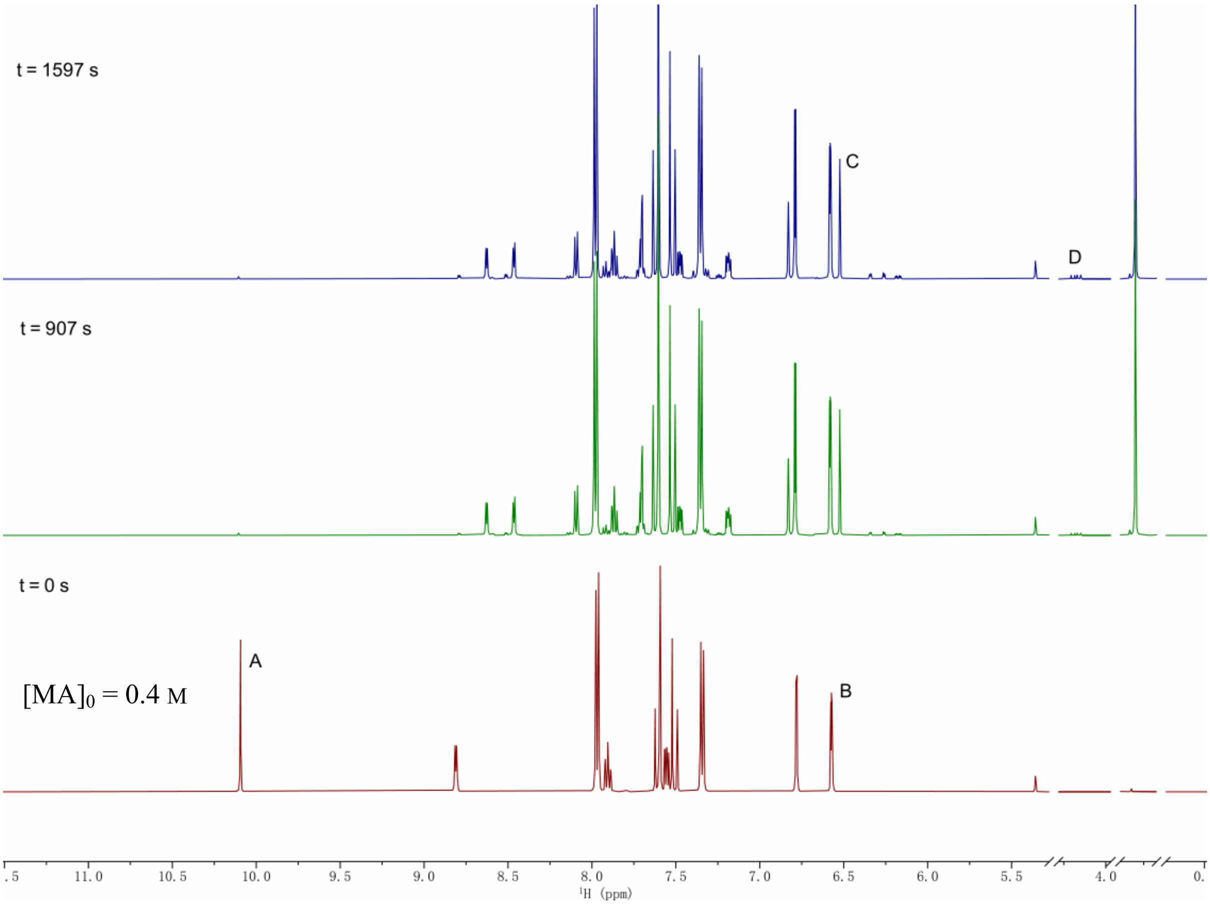
t = 1597 s
C
D
t = 907 s
t = 0 s
A
[MA]$_0$ = 0.4 M
B
.5 11.0 10.5 10.0 9.5 9.0 8.5 8.0 7.5 7.0 6.5 6.0 5.5 4.0 0.
^{1}H (ppm)

Figure S73. Representative ^{1}H NMR spectra (500 MHz) for reaction of pyridine-2-carboxaldehyde **14** (0.3 M) and Michael acceptor **88** (0.2 M, 0.3 M and 0.4 M) catalysed by *N*-C_6F_5 NHC precursor **16** (5 mM) in NEt_3:NEt_3·HCl (2:1, 0.03 M) in CD_2Cl_2 at 25 °C. A = Aldehyde C*H*O, B = Michael acceptor Ar*H*, C = Benzoin C*H*, D = Stetter product C$H_A$$H_B$.

Table S35. Reaction data and initial rates of Stetter product formation from Michael acceptor **88**, catalyzed by C_6F_5 triazolium precatalyst **16** (5 mM) in 0.03 M triethylamine buffer (Et_3N:Et_3N·HCl, 2:1) in CD_2Cl_2 at 25 °C.

[Aldehyde]$_0$ / M	[MA]$_0$ / M	Time / s	[Benzoin] / M	[Stetter] / M	v_{max} / 10^{-5} M s^{-1}	k_s' / 10^{-5} s^{-1}	Average / 10^{-5} s^{-1}
0.3	0.2	0	0	0	0.391	1.96	2.13±0.17
		360	0.135974	0.001192			
		498	0.134552	0.001573			
		636	0.133069	0.002267			
		774	0.131892	0.00284			
		912	0.130544	0.003346			
		1050	0.129256	0.003733			
		1188	0.127571	0.004664			
		1326	0.126851	0.005071			
		1464	0.125427	0.005818			
		1602	0.124715	0.006426			
0.3	0.3	0	0	0	0.709	2.36	
		346	0.135736	0.001855			
		484	0.133801	0.003146			
		622	0.131315	0.004106			
		760	0.130543	0.005007			
		898	0.129286	0.006069			
		1036	0.128701	0.006964			
		1174	0.125931	0.008294			
		1312	0.124805	0.009024			
		1450	0.123234	0.009661			
		1588	0.122441	0.010647			
0.3	0.4	0	0	0	0.826	2.07	
		355	0.136336	0.002383			
		493	0.135427	0.003489			
		631	0.132848	0.004811			
		769	0.131226	0.006134			
		907	0.130294	0.007118			
		1045	0.128569	0.008195			
		1183	0.126387	0.00956			
		1321	0.124977	0.010711			
		1459	0.12385	0.011743			

		1597	0.122094	0.012798			

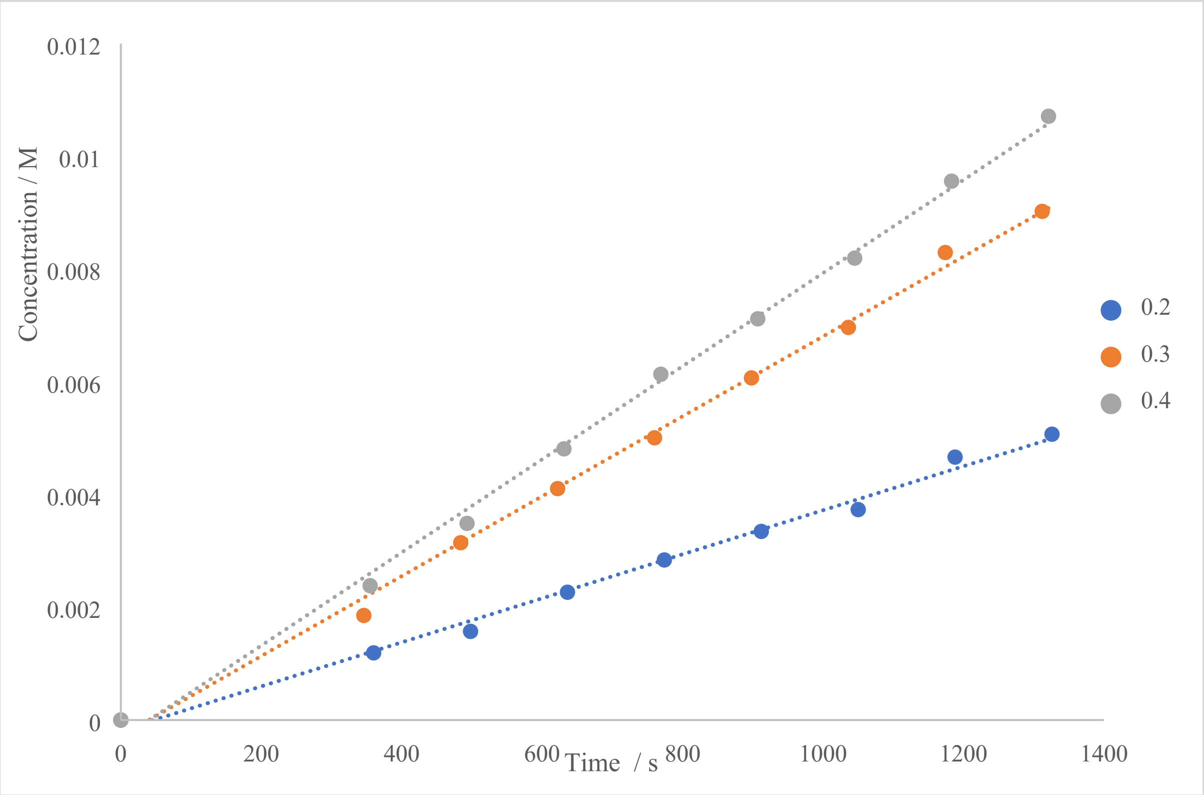

Figure S74. Plots of Stetter product concentration against time for the C_6F_5 triazolium precatalyst **16** (5 mM) catalysed Stetter reaction, at initial Michael acceptor **88** concentrations 0.2 M, 0.3 M, 0.4 M.

Entry 36

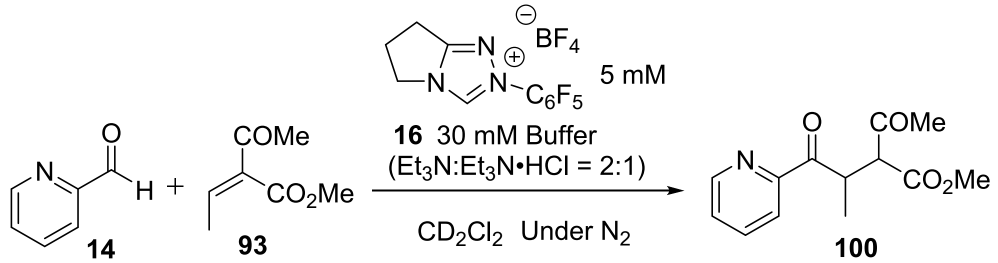

The reaction of pyridine-2-carboxaldehyde **14** and Michael acceptor **93** catalysed by C_6F_5 triazolium salt **16** in triethylamine buffer was monitored using ^1H NMR spectra, with representative NMR spectra over the course of the experiment given in Figure S75.

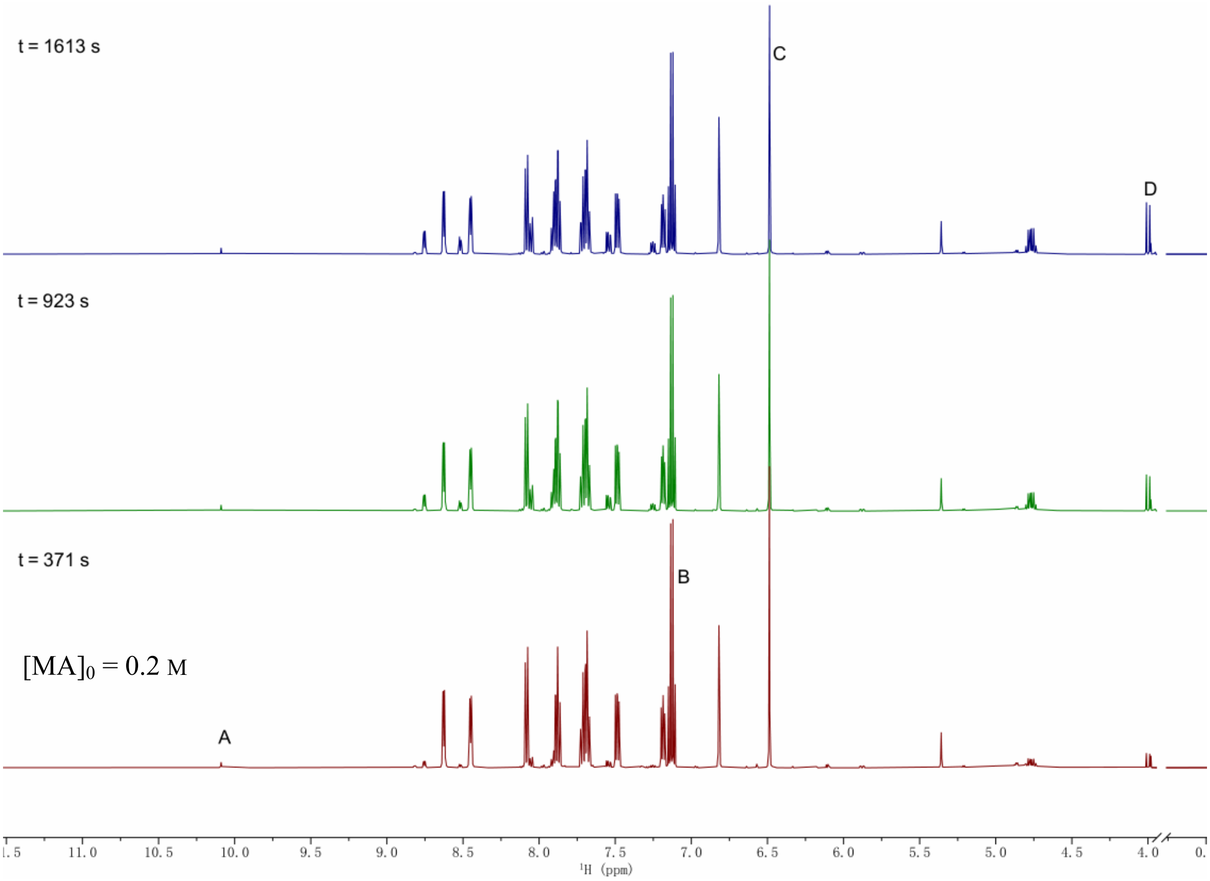
t = 1613 s
C
D
t = 923 s
t = 371 s
B
[MA]0 = 0.2 M
A
11.0
10.5
10.0
9.5
9.0
8.5
8.0
7.5
7.0
6.5
6.0
5.5
5.0
4.5
4.0
1H (ppm)

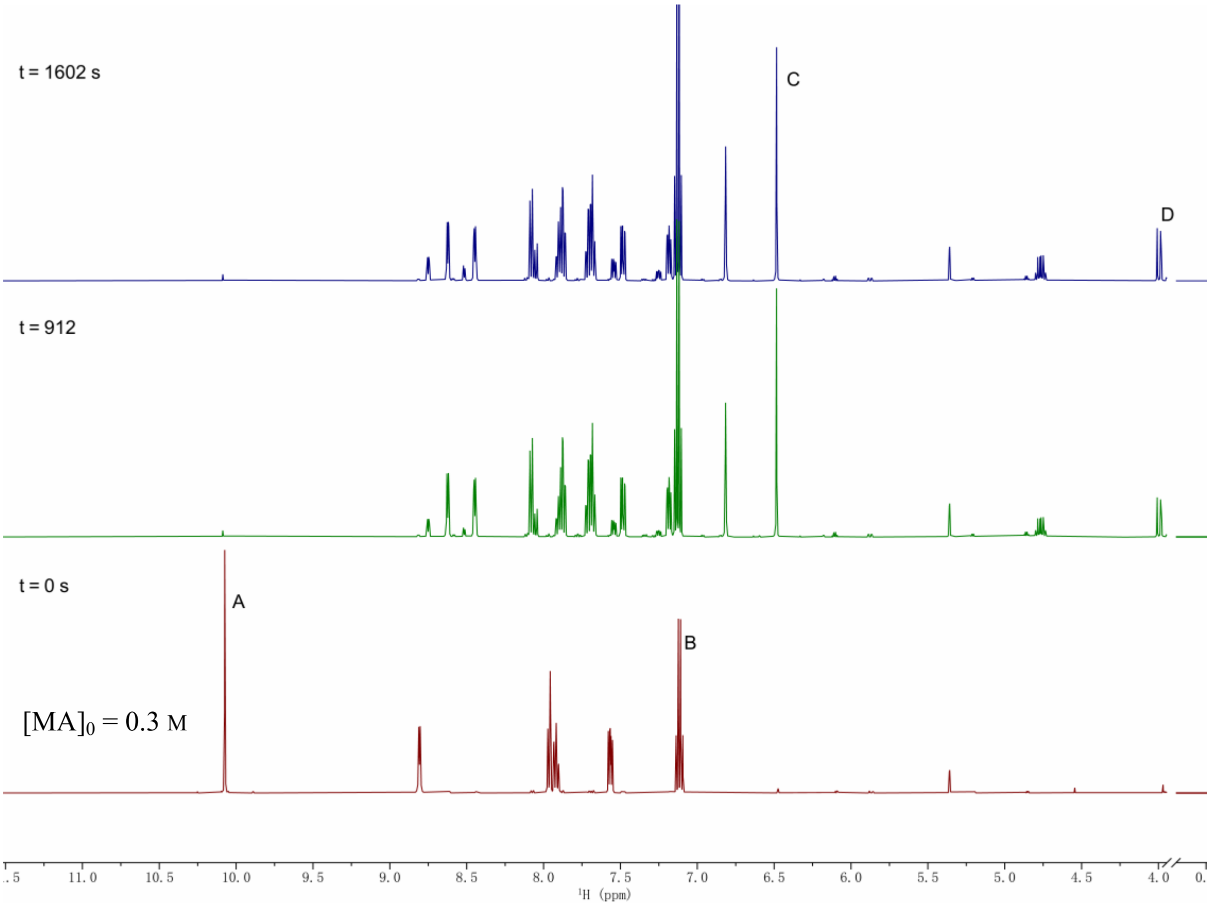
t = 1602 s
C
D
t = 912
t = 0 s
A
B
[MA]0 = 0.3 M
11.0
10.5
10.0
9.5
9.0
8.5
8.0
7.5
7.0
6.5
6.0
5.5
5.0
4.5
4.0
1H (ppm)

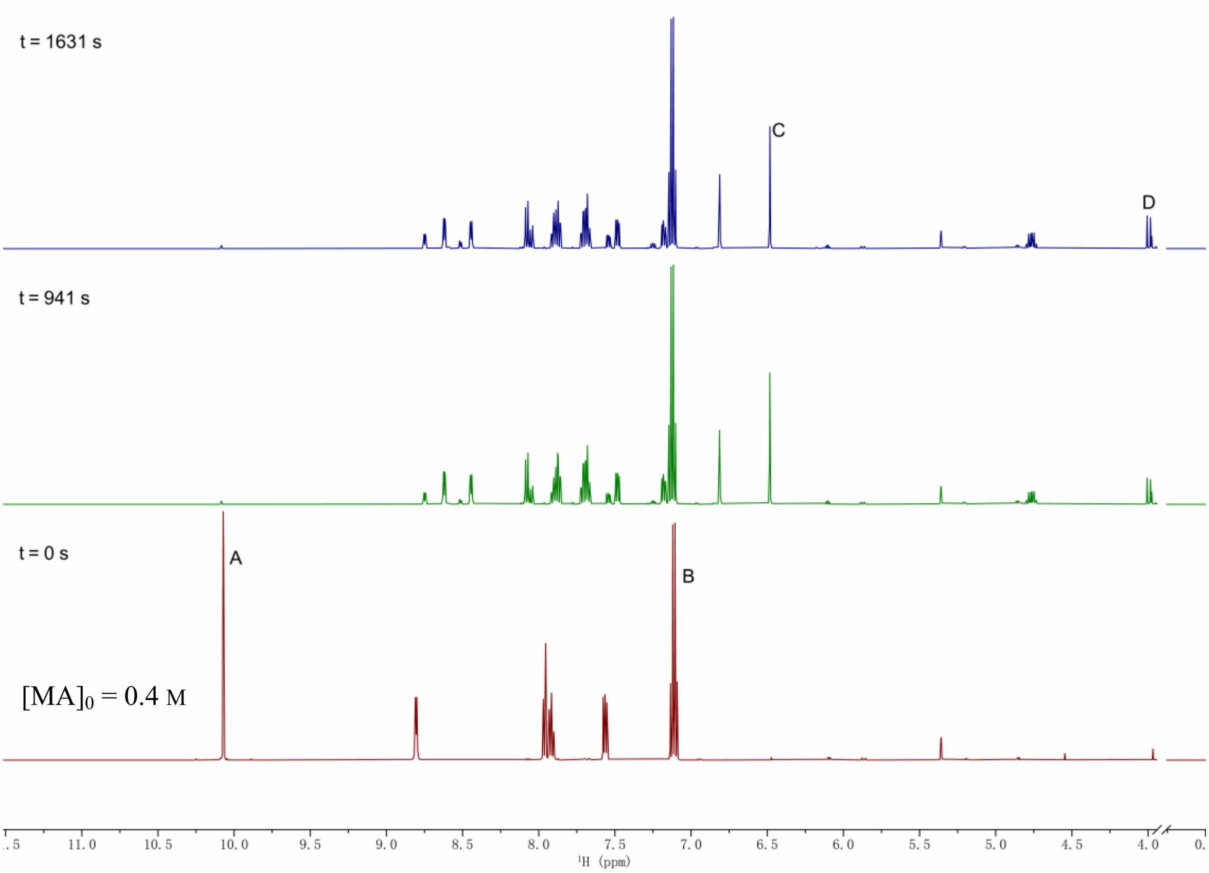

Figure S75. Representative ^{1}H NMR spectra (500 MHz) for reaction of pyridine-2-carboxaldehyde **14** (0.3 M) and Michael acceptor **93** (0.2 M, 0.3 M and 0.4 M) catalysed by *N*-C_6F_5 NHC precursor **16** (5 mM) in NEt_3:NEt_3·HCl (2:1, 0.03 M) in CD_2Cl_2 at 25 °C. A = Aldehyde C*H*O, B = Michael acceptor C*H*, C = Benzoin C*H*, D = Stetter product CH_2.

Table S36. Reaction data and initial rates of Stetter product formation from Michael acceptor **93**, catalyzed by C_6F_5 triazolium precatalyst **16** (5 mM) in 0.03 M triethylamine buffer (Et_3N:Et_3N·HCl, 2:1) in CD_2Cl_2 at 25 °C.

**[Aldehyde]$_0$ / M**	**[MA]$_0$ / M**	**Time / s**	**[Benzoin] / M**	**[Stetter] / M**	v_{max} **/ 10^{-5} M s^{-1}**	k_S' **/ 10^{-5} s^{-1}**	**Average / 10^{-5} s^{-1}**
0.3	0.2	0	0	0	2.91	14.55	13.07±1.15
		371	0.135119	0.009851			
		509	0.133986	0.015183			
		647	0.130756	0.019706			
		785	0.127558	0.023426			
		923	0.124874	0.026856			
		1061	0.122614	0.030112			
		1199	0.120466	0.032701			
		1337	0.11784	0.035297			
		1475	0.116112	0.037075			
		1613	0.113648	0.038671			

		0	0	0			
		360	0.131567	0.013498			
		498	0.13019	0.020402			
		636	0.126909	0.025207			
		774	0.123946	0.029293			
0.3	0.3	912	0.1212	0.032728	3.87	12.90	
		1050	0.118987	0.035375			
		1188	0.116914	0.037704			
		1326	0.115034	0.03943			
		1464	0.113293	0.041704			
		1602	0.111811	0.042474			
		0	0	0			
		389	0.123123	0.020846			
		527	0.121939	0.027375			
		665	0.118818	0.032754			
		803	0.115792	0.037119			
0.3	0.4	941	0.11356	0.03991	4.70	11.75	
		1079	0.111395	0.042407			
		1217	0.109608	0.045116			
		1355	0.108121	0.046768			
		1493	0.106602	0.048046			
		1631	0.105347	0.049062			

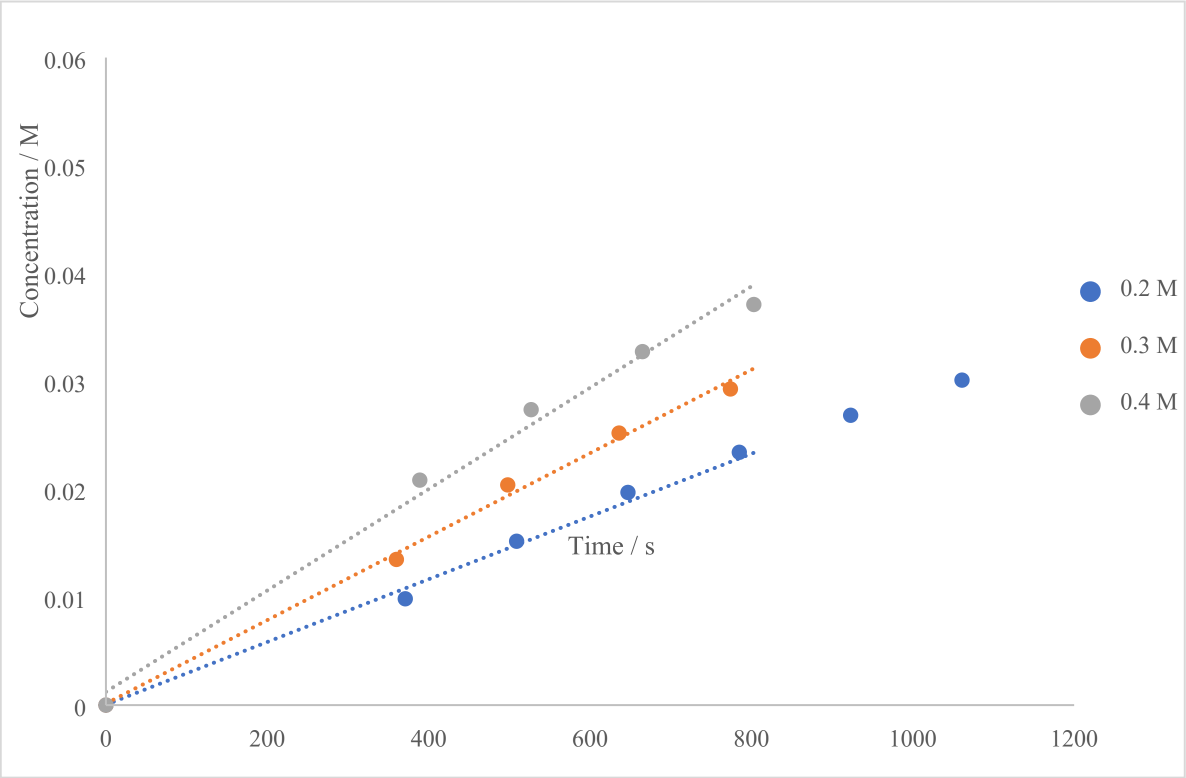

Figure S76. Plots of Stetter product concentration against time for the C_6F_5 triazolium precatalyst **16** (5 mM)

catalysed Stetter reaction, at initial Michael acceptor **93** concentrations 0.2 M, 0.3 M, 0.4 M.

Entry 37

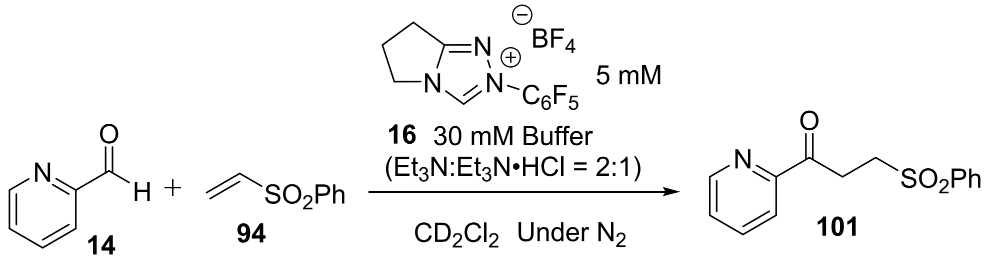

The reaction of pyridine-2-carboxaldehyde **14** and Michael acceptor **94** catalysed by C_6F_5 triazolium salt **16** in triethylamine buffer was monitored using ^{1}H NMR spectra, with representative NMR spectra over the course of the experiment given in Figure S77.

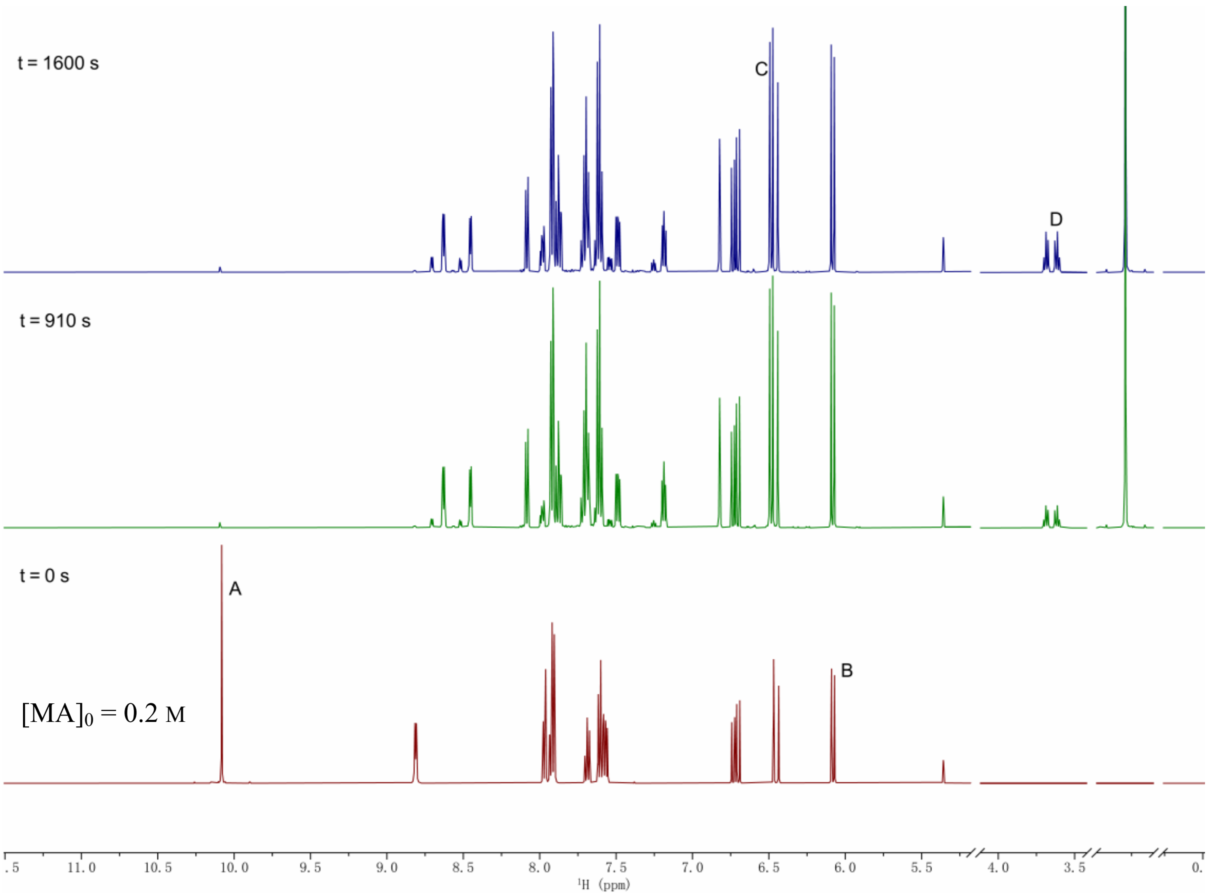

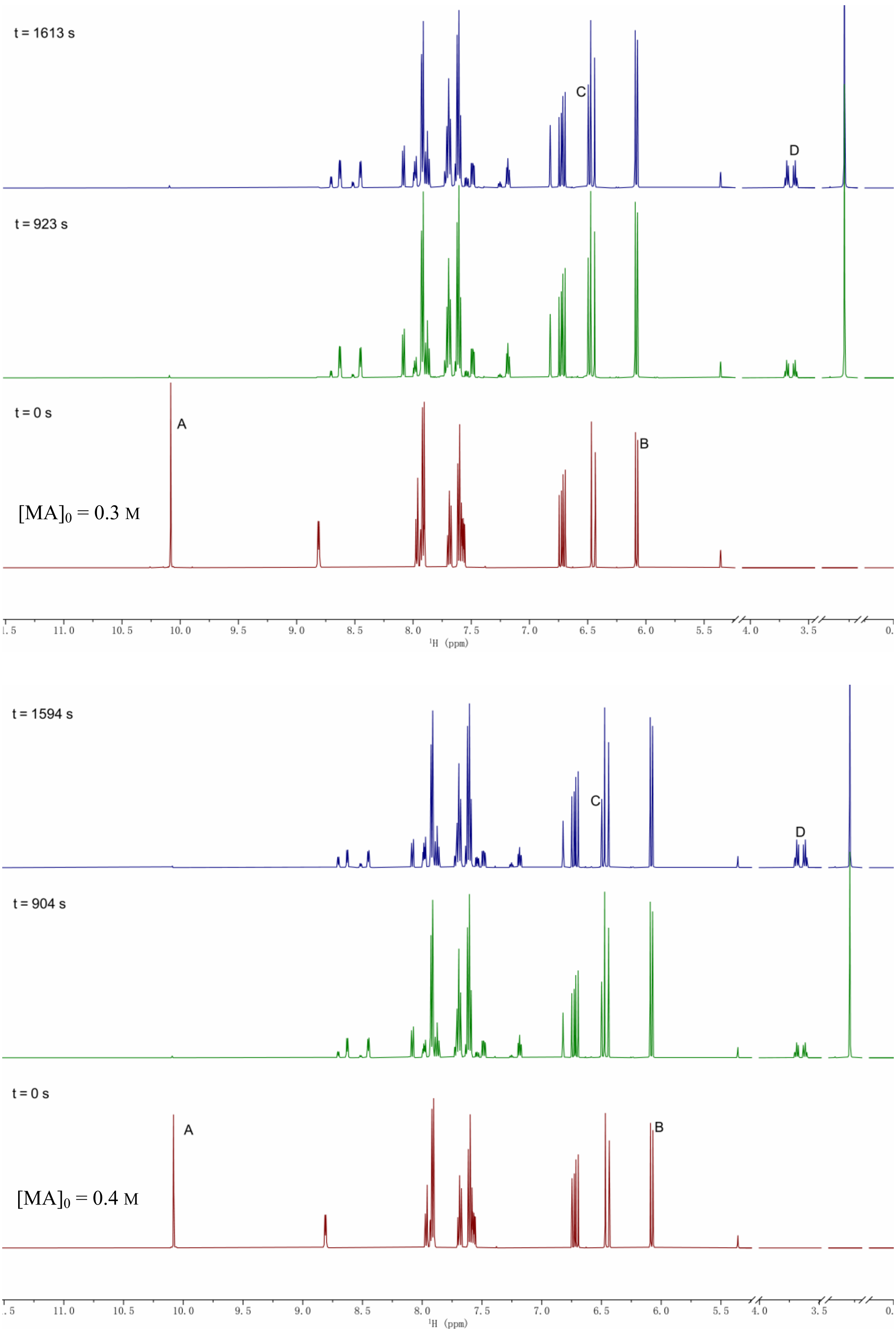

Figure S77. Representative ^{1}H NMR spectra (500 MHz) for reaction of pyridine-2-carboxaldehyde **14** (0.3

M) and Michael acceptor **94** (0.2 M, 0.3 M and 0.4 M) catalysed by *N*-C_6F_5 NHC precursor **16** (5 mM) in NEt_3:NEt_3·HCl (2:1, 0.03 M) in CD_2Cl_2 at 25 °C. A = Aldehyde C*H*O, B = Michael acceptor C*H*, C = Benzoin C*H*, D = Stetter product C*H*$_2$.

Table S37. Reaction data and initial rates of Stetter product formation from Michael acceptor **94**, catalyzed by C_6F_5 triazolium precatalyst **16** (5 mM) in 0.03 M triethylamine buffer (Et_3N:Et_3N·HCl, 2:1) in CD_2Cl_2 at 25 °C.

[Aldehyde]$_0$ / M	[MA]$_0$ / M	Time / s	[Benzoin] / M	[Stetter] / M	v_{max} / 10^{-5} M s^{-1}	k_s' / 10^{-5} s^{-1}	Average / 10^{-5} s^{-1}
0.3	0.2	0	0	0	2.17	10.85	11.16±0.22
		358	0.140788	0.006263			
		496	0.137303	0.009949			
		634	0.134856	0.013385			
		772	0.131814	0.016861			
		910	0.129798	0.019632			
		1048	0.126772	0.022549			
		1186	0.12443	0.025229			
		1324	0.122034	0.027825			
		1462	0.120238	0.03029			
		1600	0.118067	0.032582			
0.3	0.3	0	0	0	3.39	11.30	
		371	0.138871	0.011006			
		509	0.134594	0.016607			
		647	0.131677	0.022068			
		785	0.128195	0.026941			
		923	0.125304	0.031536			
		1061	0.122574	0.035985			
		1199	0.120894	0.040336			
		1337	0.116703	0.0442			
		1475	0.114961	0.048137			
		1613	0.112401	0.051957			
0.3	0.4	0	0	0	4.53	11.33	
		352	0.133987	0.01413			
		490	0.127769	0.021684			
		628	0.124947	0.028697			
		766	0.120409	0.035217			
		904	0.117374	0.041422			
		1042	0.113916	0.047472			
		1180	0.109745	0.053053			
		1318	0.106554	0.058367			
		1456	0.10314	0.063736			
		1594	0.100192	0.068905			

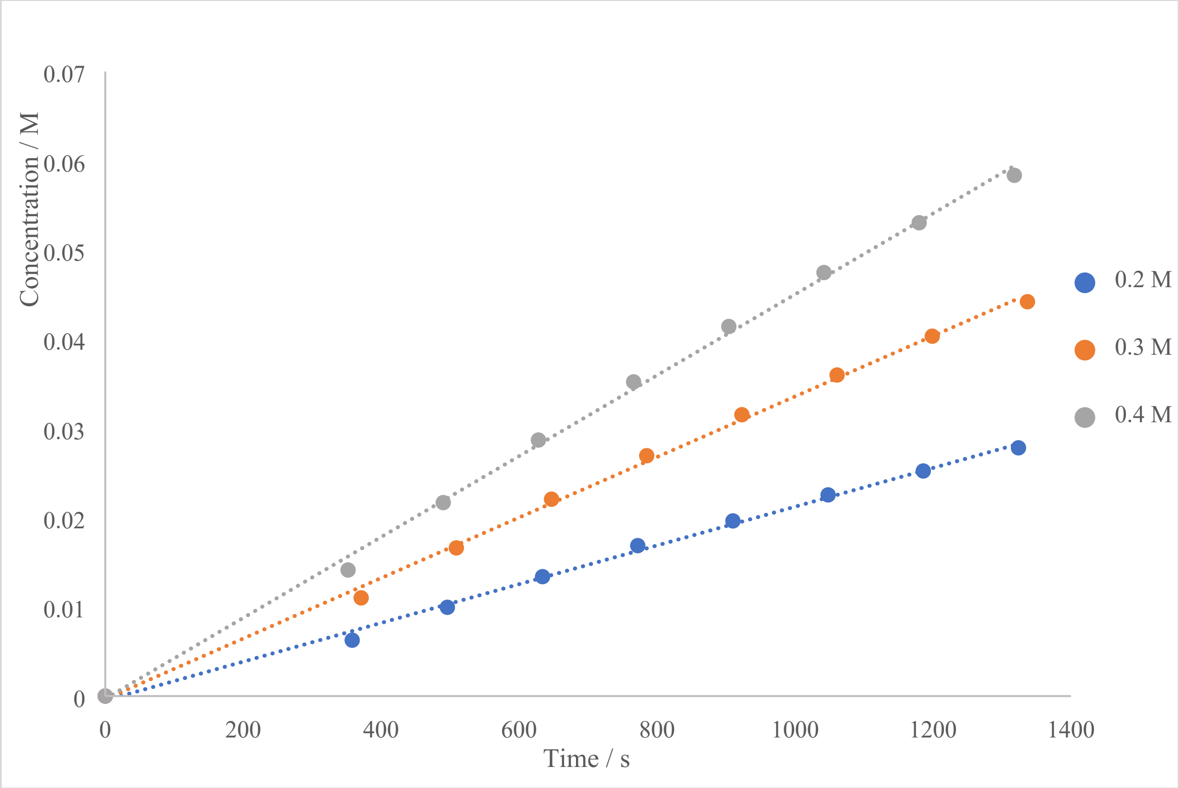

Figure S78. Plots of Stetter product concentration against time for the C_6F_5 triazolium precatalyst **16** (5 mM) catalysed Stetter reaction, at initial Michael acceptor **94** concentrations 0.2 M, 0.3 M, 0.4 M.

Entry 38

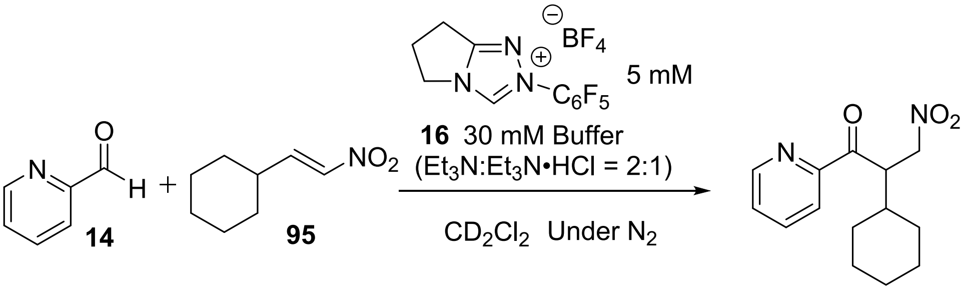

The reaction of pyridine-2-carboxaldehyde **14** and Michael acceptor **102** catalysed by C_6F_5 triazolium salt **16** in triethylamine buffer was monitored using ^{1}H NMR spectra, with representative NMR spectra over the course of the experiment given in Figure S79.

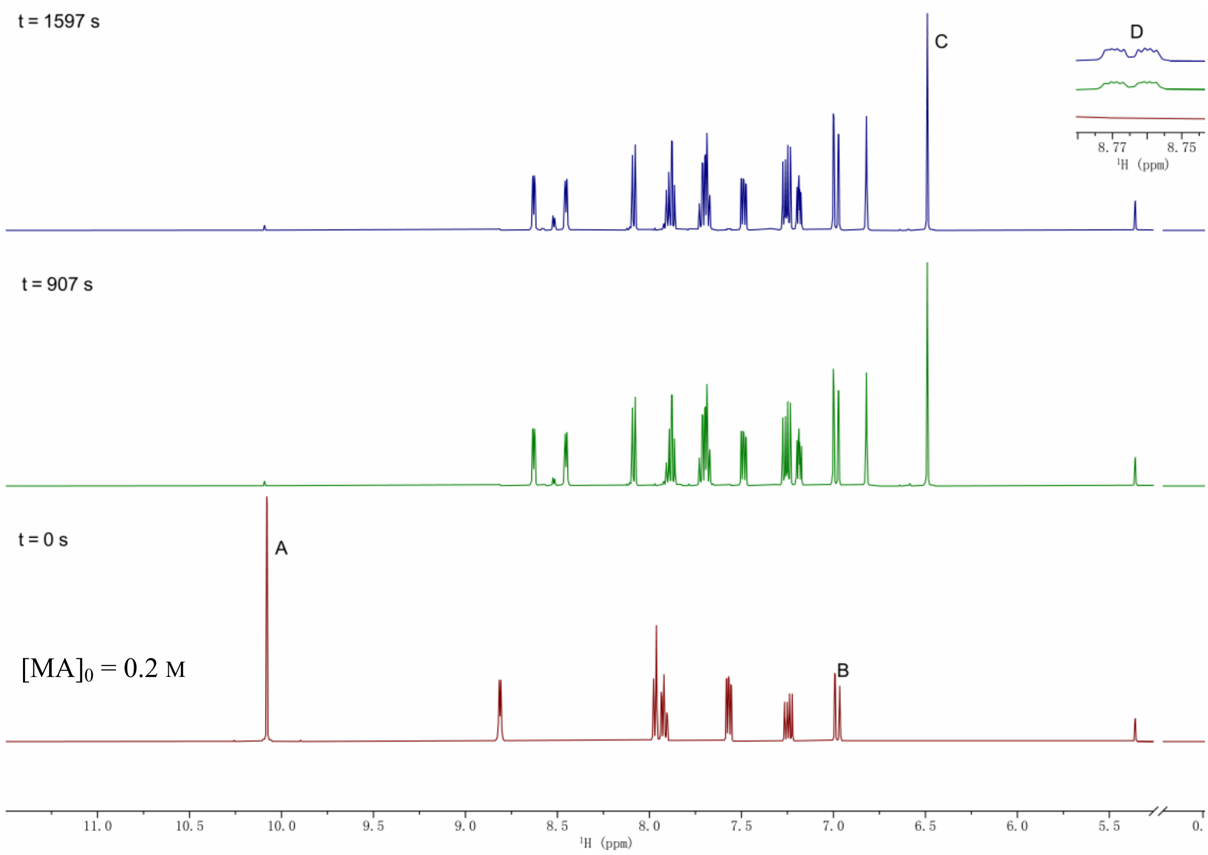

t = 1597 s
C
D
8.77
8.75
¹H (ppm)
t = 907 s
t = 0 s
A
B
[MA]₀ = 0.2 M
11.0
10.5
10.0
9.5
9.0
8.5
8.0
7.5
7.0
6.5
6.0
5.5
0.
¹H (ppm)

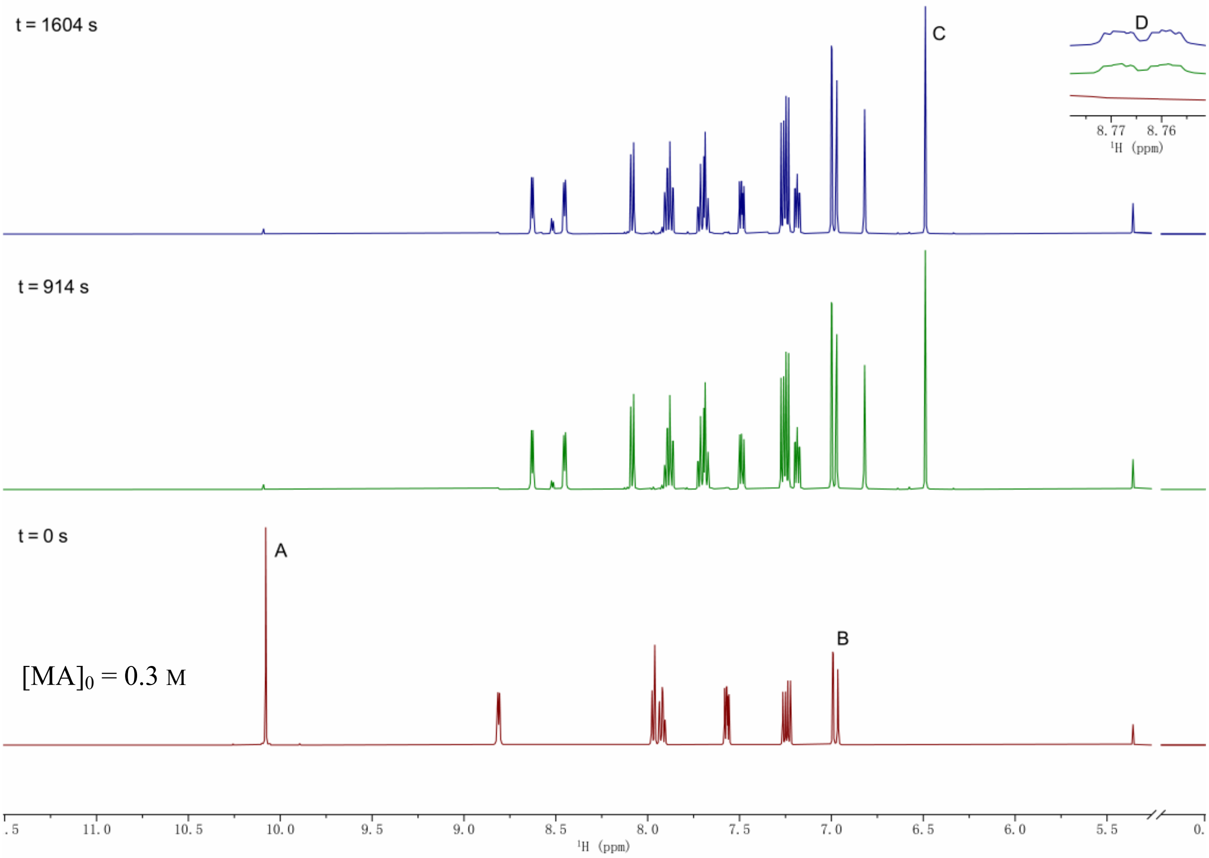

t = 1604 s
C
D
8.77
8.76
¹H (ppm)
t = 914 s
t = 0 s
A
B
[MA]₀ = 0.3 M
.5
11.0
10.5
10.0
9.5
9.0
8.5
8.0
7.5
7.0
6.5
6.0
5.5
0.
¹H (ppm)

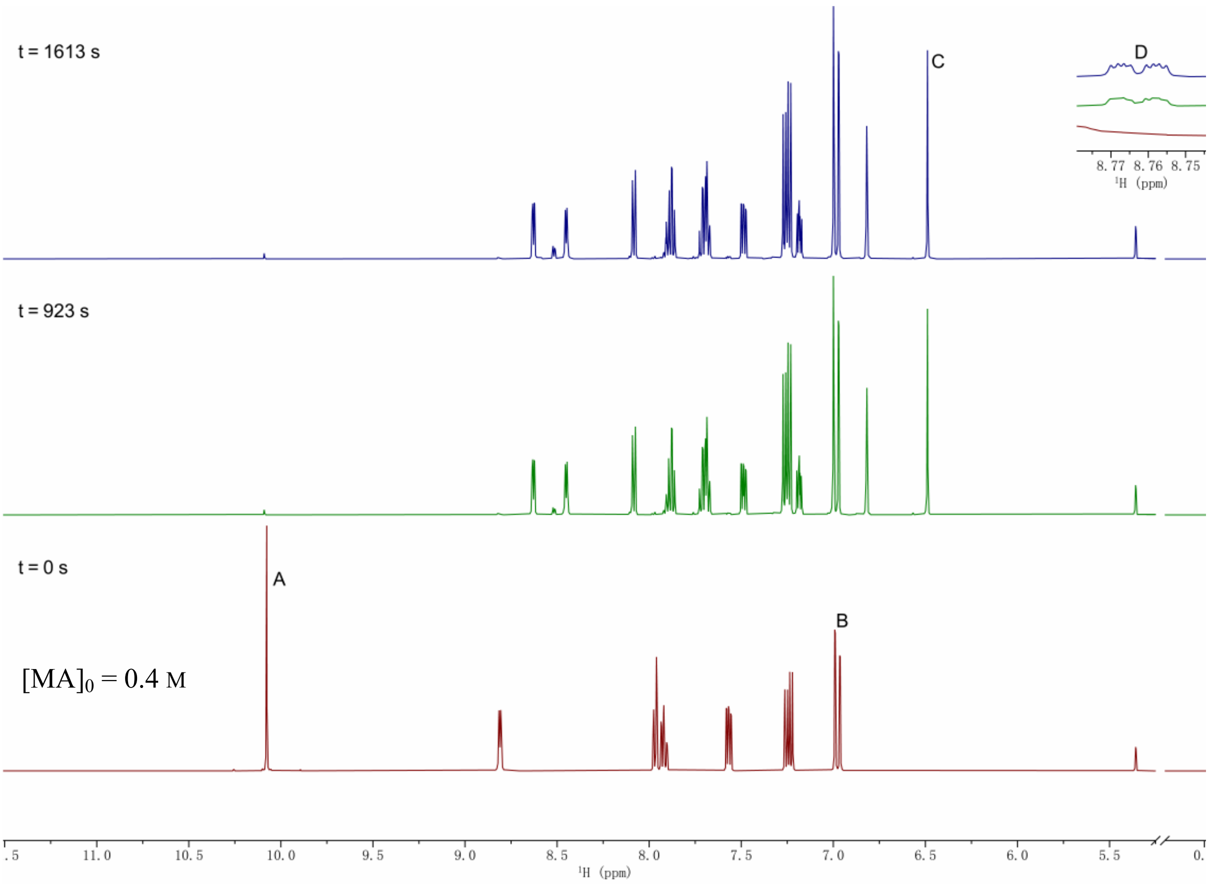

Figure S79. Representative ^{1}H NMR spectra (500 MHz) for reaction of pyridine-2-carboxaldehyde **14** (0.3 M) and Michael acceptor **95** (0.2 M, 0.3 M and 0.4 M) catalysed by *N*-C_6F_5 NHC precursor **16** (5 mM) in NEt_3:NEt_3·HCl (2:1, 0.03 M) in CD_2Cl_2 at 25 °C. A = Aldehyde C*H*O, B = Michael acceptor C*H*, C = Benzoin C*H*, D = Stetter product Ar*H*.

Table S38. Reaction data and initial rates of Stetter product formation from Michael acceptor **95**, catalyzed by C_6F_5 triazolium precatalyst **16** (5 mM) in 0.03 M triethylamine buffer (Et_3N:Et_3N·HCl, 2:1) in CD_2Cl_2 at 25 °C.

[Aldehyde]$_0$ / M	[MA]$_0$ / M	Time / s	[Benzoin] / M	[Stetter] / M	v_{max} / 10^{-5} M s^{-1}	k_S' / 10^{-5} s^{-1}	Average / 10^{-5} s^{-1}
0.3	0.2	0	0	0	0.0522	0.261	0.288±0.02
		355	0.142222	8.88E-05			
		493	0.143835	0.000274			
		631	0.142348	0.000343			
		769	0.140619	0.000252			
		907	0.139162	0.000601			
		1045	0.13737	0.00046			
		1183	0.135999	0.00064			
		1321	0.134638	0.000632			
		1459	0.133165	0.000713			
		1597	0.131898	0.000757			

		0	0	0			
		362	0.13545	0.000382			
		500	0.136735	0.000269			
		638	0.13545	0.000407			
		776	0.134474	0.000897			
0.3	0.3	914	0.132882	0.000888	0.0924	0.308	
		1052	0.131312	0.000858			
		1190	0.13025	0.001108			
		1328	0.128903	0.001171			
		1466	0.127483	0.00103			
		1604	0.126362	0.001321			
		0	0	0			
		371	0.120249	0.000567			
		509	0.121662	0.000717			
		647	0.120622	0.000896			
		785	0.119561	0.001148			
0.3	0.4	923	0.11876	0.001404	0.118	0.295	
		1061	0.117361	0.001183			
		1199	0.116536	0.001423			
		1337	0.115562	0.001681			
		1475	0.114587	0.001524			
		1613	0.11365	0.001613			

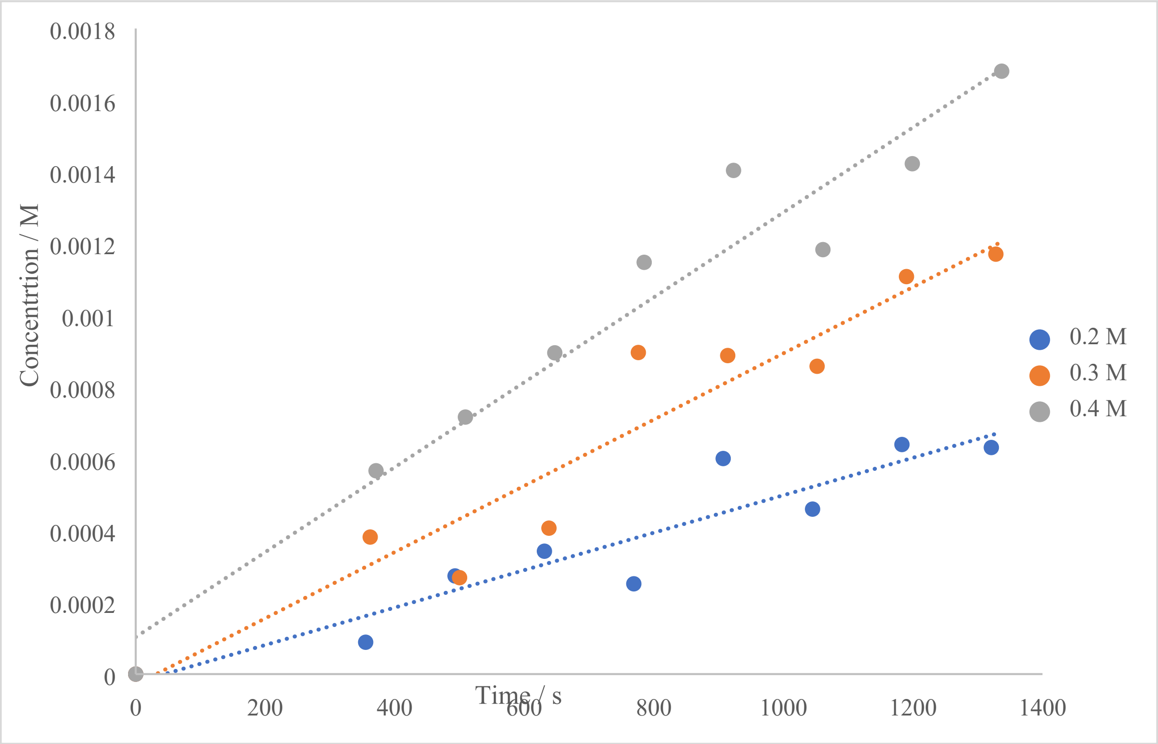

Figure S80. Plots of Stetter product concentration against time for the C_6F_5 triazolium precatalyst **16** (5 mM) catalysed Stetter reaction, at initial Michael acceptor **95** concentrations 0.2 M, 0.3 M, 0.4 M.

Entry 39

a)

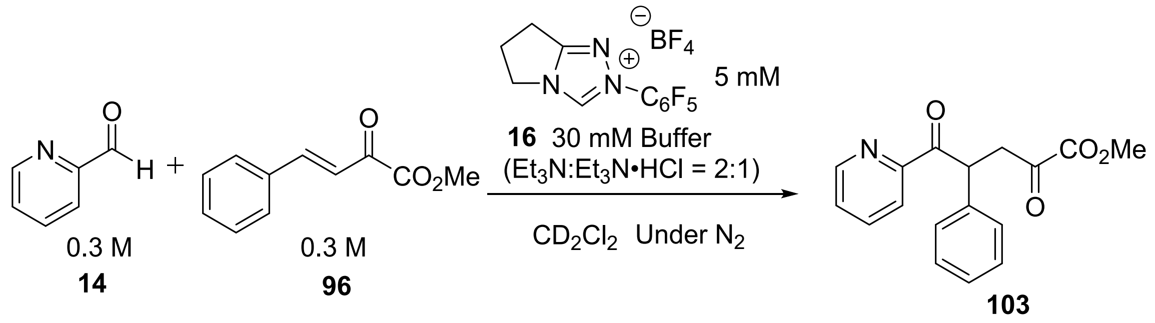

The reaction of pyridine-2-carboxaldehyde **14** and Michael acceptor **96** catalysed by C_6F_5 triazolium salt **16** in triethylamine buffer was monitored using ^{1}H NMR spectra, with representative NMR spectra over the course of the experiment given in Figure S81.

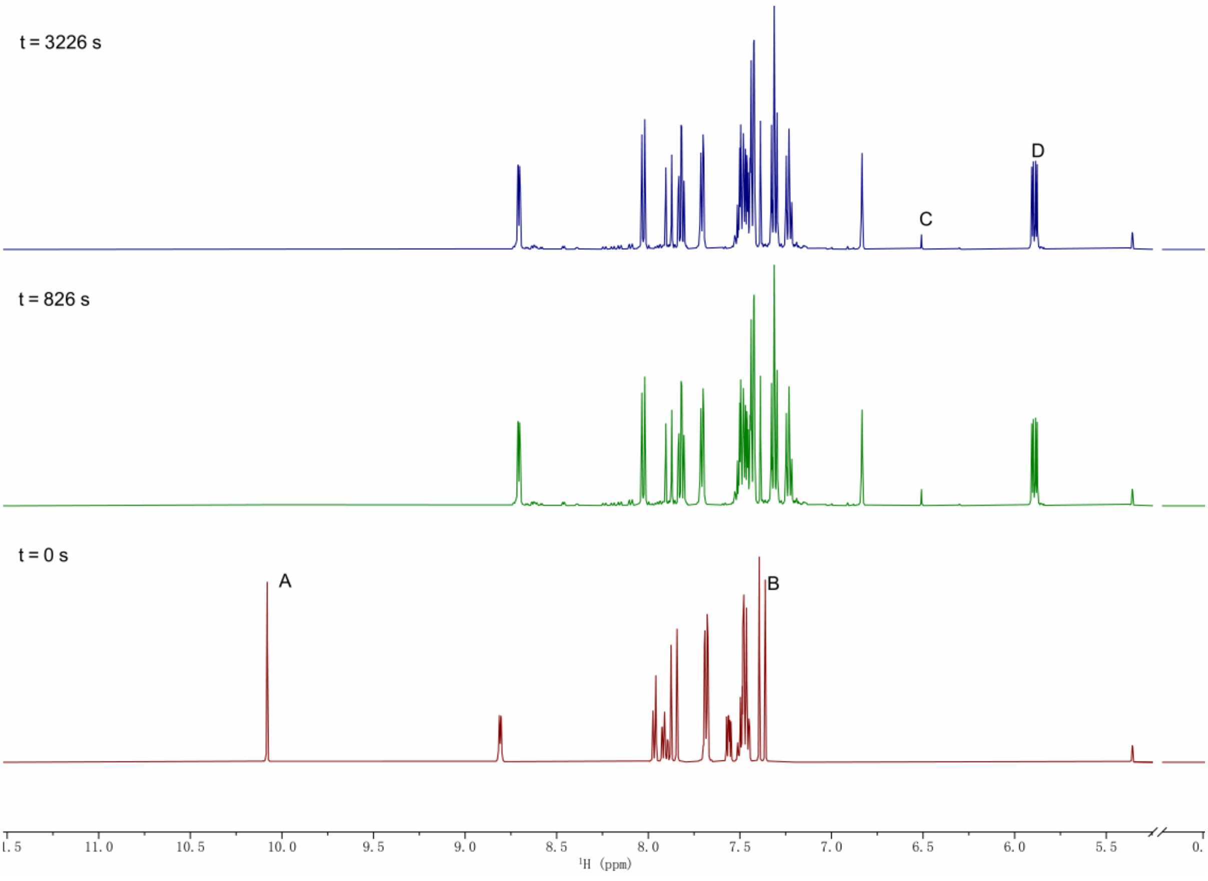

Figure S81. Representative ^{1}H NMR spectra (500 MHz) for reaction of pyridine-2-carboxaldehyde **14** (0.3 M) and Michael acceptor **96** (0.3 M) catalysed by *N*-C_6F_5 NHC precursor **16** (5 mM) in NEt_3:NEt_3·HCl (2:1, 0.03 M) in CD_2Cl_2 at 25 °C. A = Aldehyde C*H*O, B = Michael acceptor C*H*, C = Benzoin C*H*, D = Stetter product C*H*.

b)

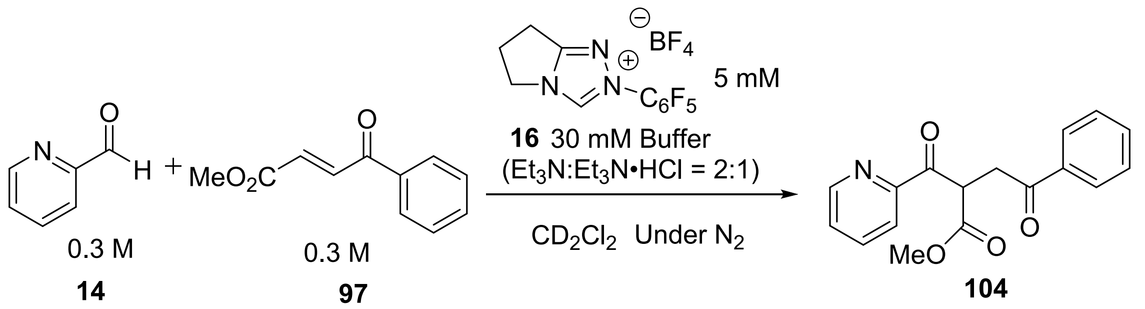

The reaction of pyridine-2-carboxaldehyde **14** and Michael acceptor **97** catalysed by C_6F_5 triazolium salt **16** in triethylamine buffer was monitored using ^{1}H NMR spectra, with representative NMR spectra over the course of the experiment given in Figure S82.

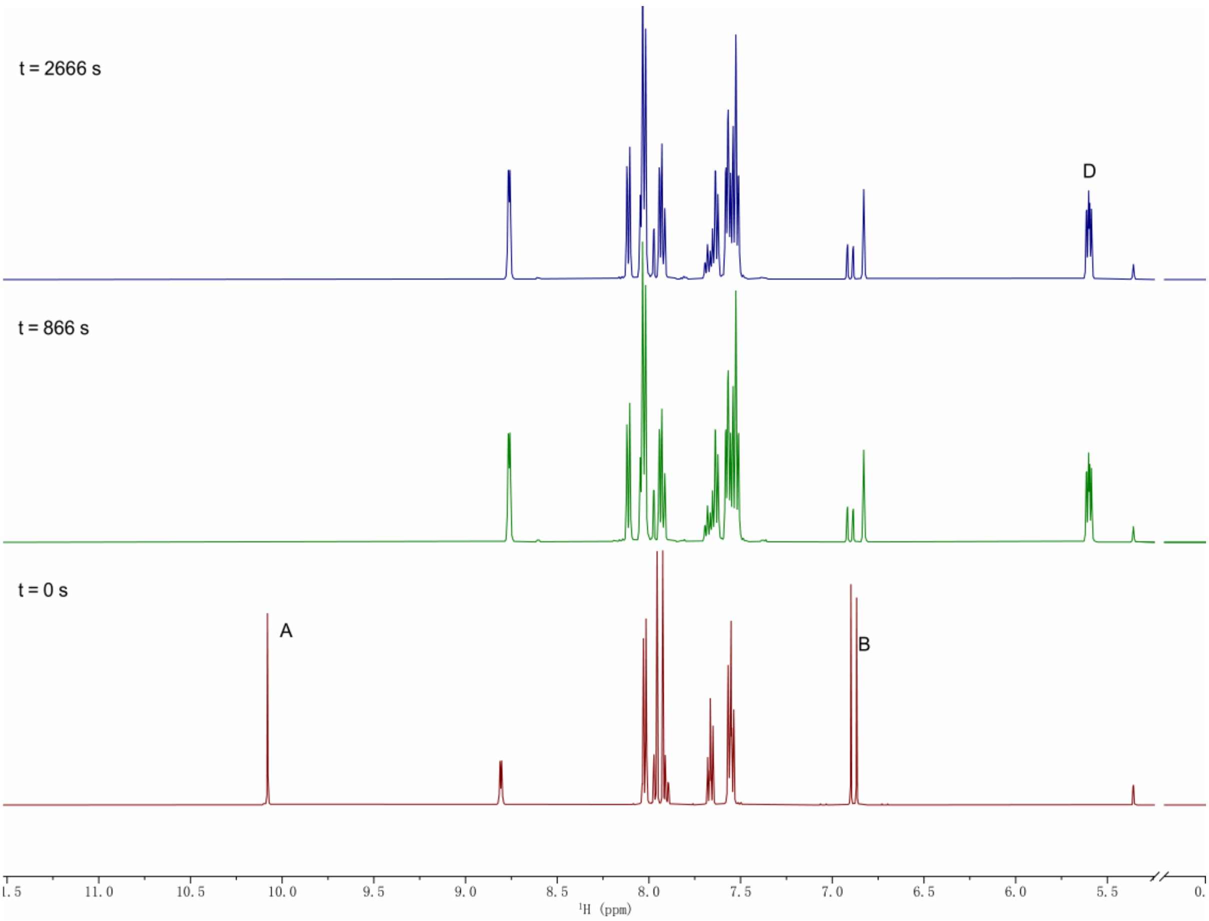

Figure S82. Representative ^{1}H NMR spectra (500 MHz) for reaction of pyridine-2-carboxaldehyde **14** (0.3 M) and Michael acceptor **97** (0.3 M) catalysed by *N*-C_6F_5 NHC precursor **16** (5 mM) in NEt_3:NEt_3·HCl (2:1, 0.03 M) in CD_2Cl_2 at 25 °C. A = Aldehyde C*H*O, B = Michael acceptor C*H*, D = Stetter product C*H*.

c)

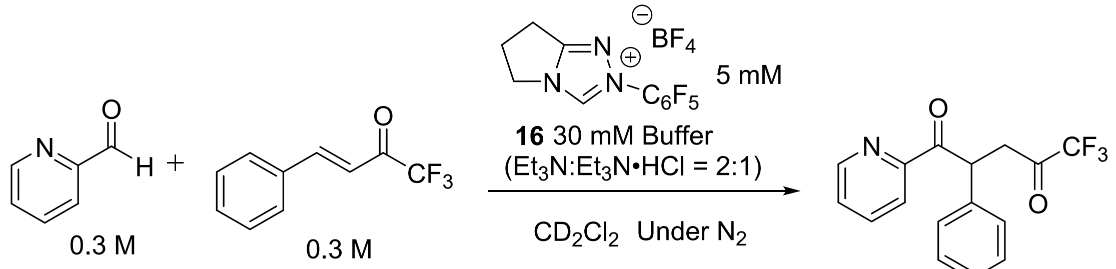

14 98 105

The reaction of pyridine-2-carboxaldehyde **14** and Michael acceptor **98** catalysed by C_6F_5 triazolium salt **16** in triethylamine buffer was monitored using ^{1}H NMR spectra, with representative NMR spectra over the course of the experiment given in Figure S83.

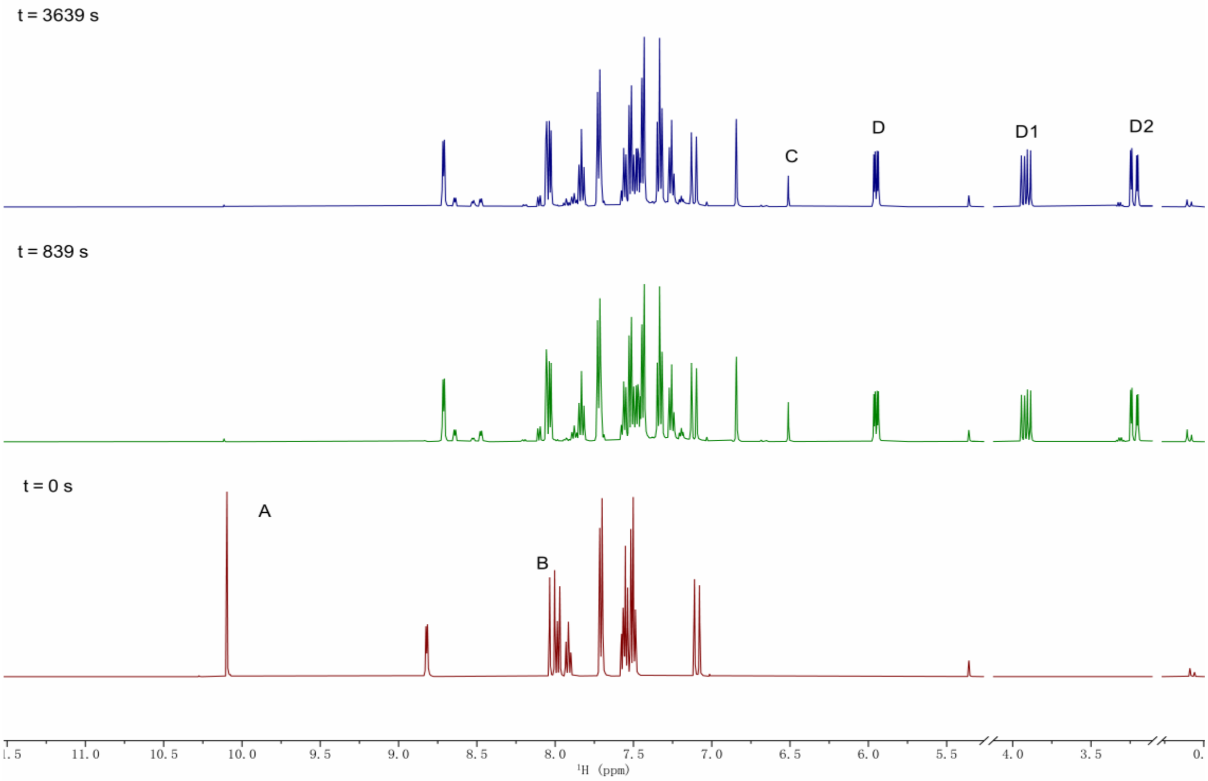

Figure S83. Representative ^{1}H NMR spectra (500 MHz) for reaction of pyridine-2-carboxaldehyde **14** (0.3 M) and Michael acceptor **98** (0.3 M) catalysed by *N*-C_6F_5 NHC precursor **16** (5 mM) in NEt_3:NEt_3·HCl (2:1, 0.03 M) in CD_2Cl_2 at 25 °C. A = Aldehyde C*H*O, B = Michael acceptor C*H*, C = Benzoin C*H*, D = Stetter product C*H*, D1 = Stetter product CH_AH$_B$, D = Stetter product CH$_A$$H_B$.

d)

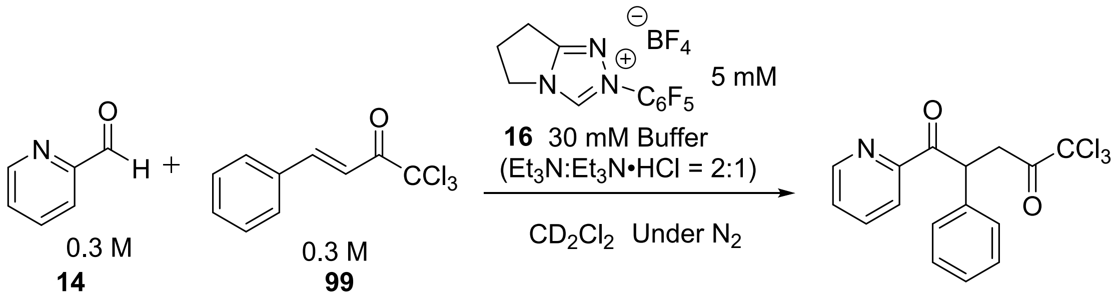

14 99 106

The reaction of pyridine-2-carboxaldehyde **14** and Michael acceptor **99** catalysed by C_6F_5 triazolium salt **16** in triethylamine buffer was monitored using ^{1}H NMR spectra, with representative NMR spectra over the course of the experiment given in Figure S84.

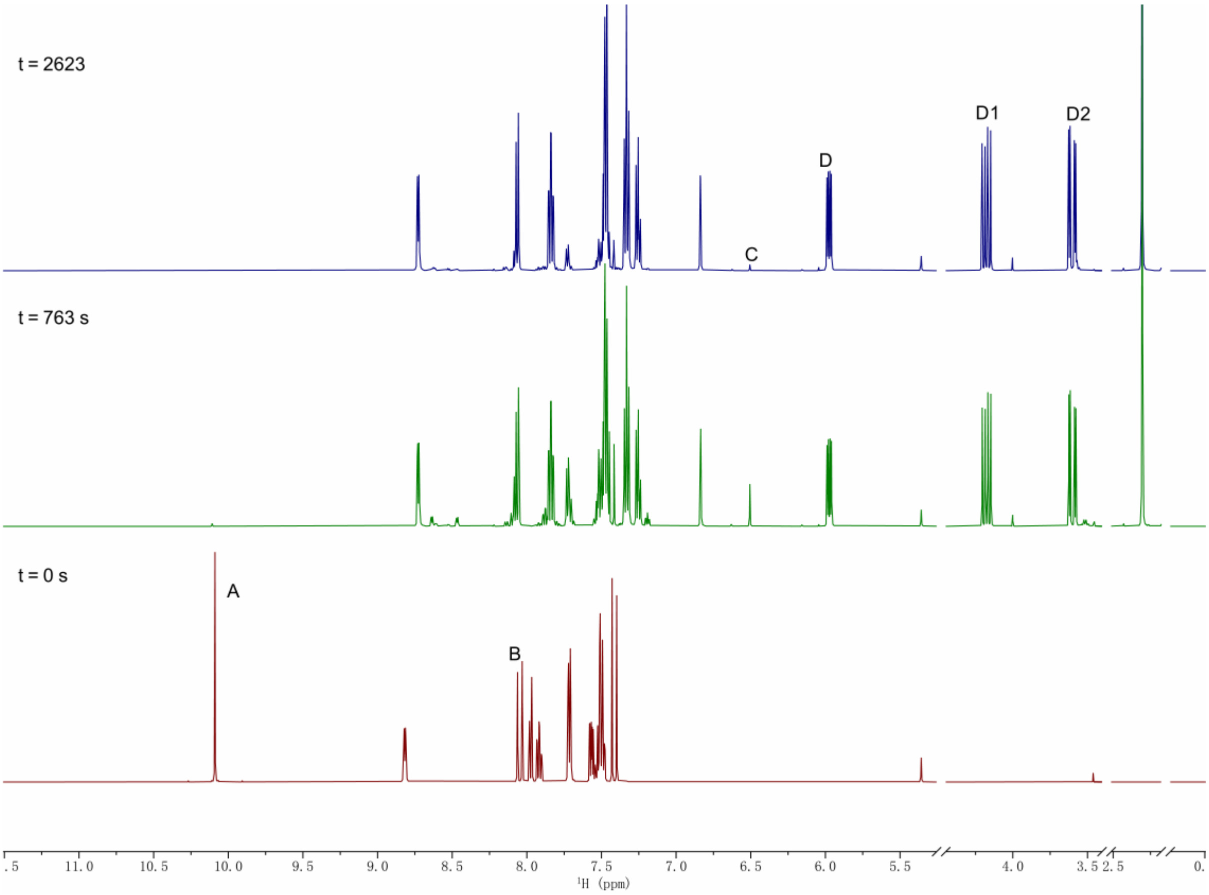

Figure S84. Representative ^{1}H NMR spectra (500 MHz) for reaction of pyridine-2-carboxaldehyde **14** (0.3 M) and Michael acceptor **99** (0.3 M) catalysed by *N*-C_6F_5 NHC precursor **16** (5 mM) in NEt_3:NEt_3·HCl (2:1, 0.03 M) in CD_2Cl_2 at 25 °C. A = Aldehyde C*H*O, B = Michael acceptor C*H*, C = Benzoin C*H*, D = Stetter product C*H,* D1 = Stetter product C$H_A$$H_B$, D = Stetter product C$H_A$$H_B$.

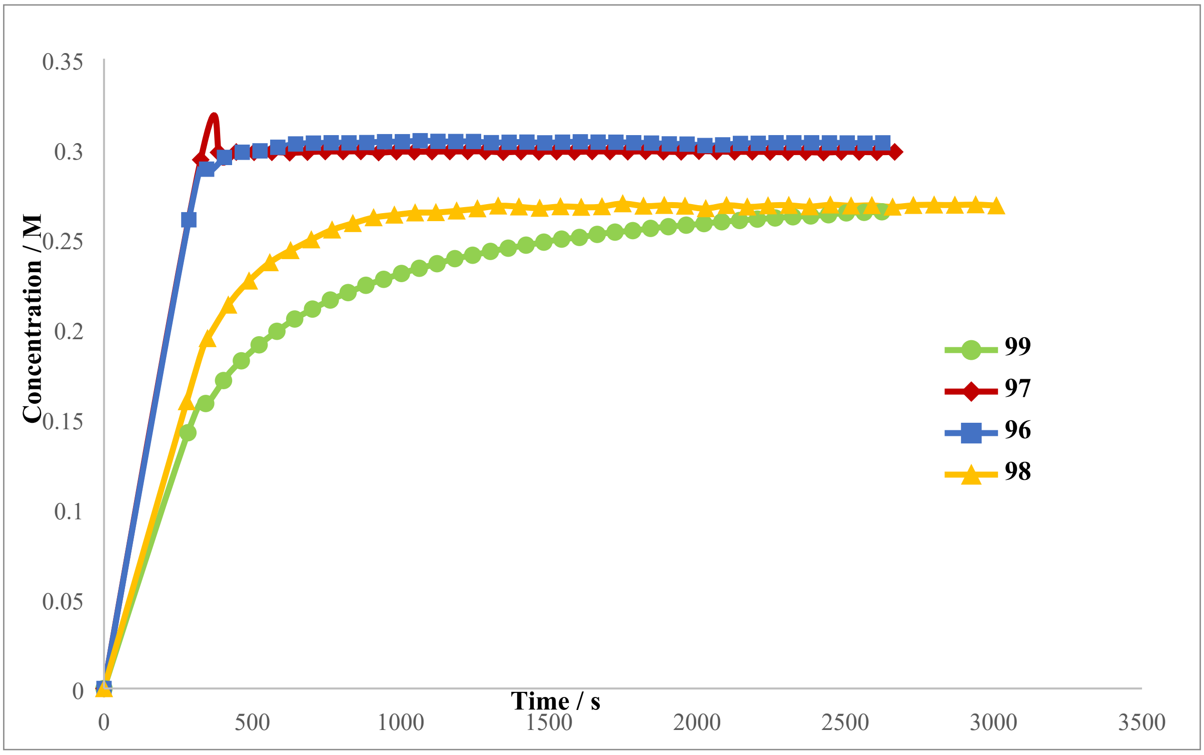

Figure S85. Plots of Stetter product concentration against time for the C_6F_5 triazolium precatalyst **16** (5 mM) catalysed Stetter reaction with Michael acceptors **96-99**.

4. Synthesis of Stetter Products

General Procedure C

Michael acceptor (0.6 mmol, 1.0 equiv) and triazolium salt (0.06 mmol, 0.1 equiv) was added into a 5 mL vial with a magnetic stir bar, and 2-pyridinecarboxylaldehyde (0.6 mmol, 1.0 equiv) and anhydrous dichloromethane (2 mL) was added into the mixture. The reaction was initiated by adding triethylamine (0.06 mmol, 0.1 equiv), and then stirred under nitrogen at room temperature, and stopped the reaction until benzoin totally disappeared using TLC to detect the benzoin (0.5-4h), followed by concentration in vacuo. Column chromatography (petroleum ether:ethyl acetate, 8:1) of the resulting residue gave the Stetter product (Some Stetter products are the oil state after column chromatography, but it will slowly transfer the solid after 1-3 days) and enediol product (Some enediol cannot be isolated as a result of overlap with chalcones during doing column chromatography).

2,4-diphenyl-1-(pyridin-2-yl)butane-1,4-dione 18

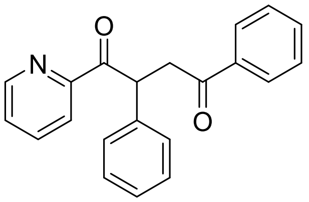

White solid; Yield, 52%; $R_f = 0.28$ (petroleum ether:ethyl acetate, 5:1); **mp**, 84-85 °C (literature: 88-89 °C[42]). **IR** ν_{max} (film): 3053, 2907, 1694, 1682, 1331, 1229, 995; **^{1}H NMR** (400 MHz, CD_2Cl_2) δ_H: 3.48 (1H, dd, *J* 18.1, 3.6, CH_AH$_B$), 4.21 (1H, dd, *J* 18.1, 10.9, CH$_A$$H_B$), 6.02 (1H, dd, *J* 11.0, 3.6, C*H*), 7.22-7.26 (1H, m, Ar*H*), 7.30-7.35 (2H, m, Ar*H*), 7.48-7.53 (5H, m, Ar*H*), 7.60-7.64 (1H, m, Ar*H*), 7.85 (1H, td, *J* 7.7, 1.8, Ar*H*), 8.00-8.07 (3H, m, Ar*H*), 8.75 (1H, ddd, *J* 4.7, 1.7, 0.9, Ar*H*). Data in accordance with literature.[42]

2-(4-methoxyphenyl)-4-phenyl-1-(pyridin-2-yl)butane-1,4-dione 35

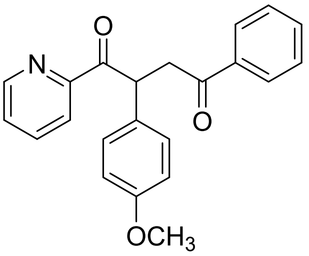

White solid; Yield, 46%; $R_f = 0.18$ (petroleum ether:ethyl acetate, 5:1); **mp**, 94-95 °C. **IR** ν_{max} (film): 3055, 2907, 1678, 1508, 1244, 995; **^{1}H NMR** (400 MHz, $CDCl_3$) δ_H: 3.49 (1H, dd, *J* 18.1, 3.7, CH_AH$_B$), 3.77 (3H, s, CH_3), 4.21 (1H, dd, *J* 18.1, 10.9, CH$_A$$H_B$), 6.03 (1H, dd, *J* 10.9, 3.7, C*H*), 6.83-6.87 (2H, m, 2′,6′-*H*-2-Ph), 7.42-7.49 (5H, m, 5-Ar*H* + 3′,5′-*H*-2-Ph + 3′,5′-*H*-4-Ph), 7.55-7.60 (1H, m, 4′-*H*-4-Ph), 7.84 (1H, td, *J* 7.7, 1.7, 3-Ar*H*), 7.99-8.01 (2H, m, 2′,6′-*H*-4-Ph), 8.10 (1H, dt, *J* 7.9, 1.1, 6-Ar*H*), 8.78 (1H, ddd, *J* 4.8, 1.8, 0.9, 4-Ar*H*). **^{13}C{^{1}H} NMR** (101 MHz, $CDCl_3$) δ_C: 16.7 (*C*H$_2$), 45.4 (*C*H), 55.2 (*C*H$_3$), 114.2 (2′,6′-*C*H-2-Ph), 123.1 (6-Ar*C*H), 127.0 (5-Ar*C*H), 128.2 (2′,6′-*C*H-4-Ph), 128.6 (3′,5′-*C*H-4-Ph), 129.8 (1′-*C*-2-Ph), 130.0 (3′,5′-*C*H-2-Ph), 133.2 (4′-*C*H-4-Ph), 136.4 (1′-*C*-4-Ph), 137.3 (3-Ar*C*H), 148.7 (4-Ar*C*H), 152.2 (2-Ar*C*), 158.8 (4′-*C*-2-Ph), 198.2 (4-*C*=O), 199.7 (1-*C*=O); **HRMS** (ESI$^+$) $C_{22}H_{20}NO_3$ [M+H]$^+$ found 346.1427, requires 346.1443 (–3.2ppm).

4-phenyl-1-(pyridin-2-yl)-2-(p-tolyl)butane-1,4-dione 36

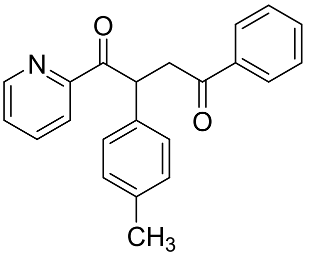

Colorless oil; Yield, 45%; R_f = 0.20 (petroleum ether:ethyl acetate, 5:1); **IR** ν_{max} (film): 3053, 2916, 1682, 1449, 1229, 995; **^{1}H NMR** (400 MHz, $CDCl_3$) δ_H: 2.31 (3H, s, C*H₃*), 3.48 (1H, dd, *J* 18.2, 3.6, CH_AH$_B$), 4.25 (1H, dd, *J* 18.2, 11.1, CH$_A$$H_B$), 6.05 (1H, dd, *J* 11.1, 3.5, C*H*), 7.11-7.15 (2H, m, 3′,5′-*H*-2-Ph), 7.39-7.49 (5H, m, 5-Ar*H* + 2′,6′-*H*-2-Ph + 3′,5′-*H*-4-Ph), 7.56-7.60 (1H, m, 4′-*H*-4-Ph), 7.80 (1H, td, *J* 7.7, 1.7, 3-Ar*H*), 7.99-8.02 (2H, m, 2′,6′-*H*-4-Ph), 8.08 (1H, dt, *J* 7.8, 1.1, 6-Ar*H*), 8.77 (1H, ddd, *J* 4.8, 1.7, 0.9, 4-Ar*H*). **^{13}C{^{1}H} NMR** (101 MHz, $CDCl_3$) δ_C: 21.1 (*C*H$_3$), 16.7 (*C*H$_2$), 45.4 (*C*H), 122.9 (6-Ar*C*H), 126.9 (5-Ar*C*H), 128.2 (2′,6′-*C*H-4-Ph), 128.6 (3′,5′-*C*H-4-Ph), 128.8 (2′, 6′-*C*H-2-Ph), 129.5 (3′,5′-*C*H-2-Ph), 133.2 (4′-*C*H-4-Ph), 135.0 (1′-*C*-2-Ph), 135.0 (1′-*C*-4-Ph), 136.4 (4′-*C*-2-Ph), 136.8 (3-Ar*C*H), 149.0 (4-Ar*C*H), 152.6 (2-Ar*C*), 198.2 (4-*C*=O), 200.1 (1-*C*=O); **HRMS** (ESI$^+$) $C_{22}H_{20}NO_2$ [M+H]$^+$ found 330.1482, requires 330.1494 (–2.0 ppm).

2-(4-fluorophenyl)-4-phenyl-1-(pyridin-2-yl)butane-1,4-dione 37

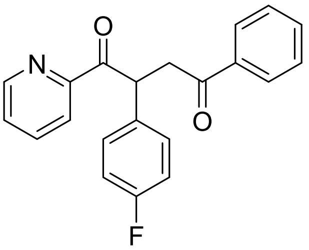

White solid; Yield, 51%; R_f = 0.27 (petroleum ether:ethyl acetate, 5:1); **mp**, 104-105 °C. **IR** ν_{max} (film): 3059, 2924, 1697, 1680, 1499, 1217, 999; **^{1}H NMR** (400 MHz, $CDCl_3$) δ_H: 3.50 (1H, dd, *J* 18.1, 3.8, CH_AH$_B$), 4.20 (1H, dd, *J* 18.1, 10.8, CH$_A$$H_B$), 6.08 (1H, dd, *J* 10.8, 3.8, C*H*), 6.97-7.02 (2H, m, 3′,5′-*H*-2-Ph), 7.45-7.52 (5H, m, 5-Ar*H* + 2′,6′-*H*-2-Ph + 3′,5′-*H*-4-Ph), 7.56-7.61 (1H, m, 4′-*H*-4-Ph), 7.84 (1H, td, *J* 7.7, 1.8, 3-Ar*H*), 7.99-8.02 (2H, m, 2′,6′-*H*-4-Ph), 8.09 (1H, dt, *J* 7.9, 1.1, 6-Ar*H*), 8.76 (1H, ddd, *J* 4.8, 1.8, 0.9, 4-Ar*H*). **^{13}C{^{1}H} NMR** (101 MHz, $CDCl_3$) δ_C: 16.6 (*C*H$_2$), 45.1 (*C*H), 115.5 (3′,5′-*C*H-2-Ph), 115.7 (3′,5′-*C*H-2-Ph), 123.0 (6-Ar*C*H), 127.2 (5-Ar*C*H), 128.2 (2′,6′-*C*H-4-Ph), 128.6 (3′,5′-*C*H-4-Ph), 130.5

(2′,6′-*C*H-2-Ph), 130.5 (2′,6′-*C*H-2-Ph), 133.3 (4′-*C*H-4-Ph), 133.7 (1′-*C*-2-Ph), 133.7 (1′-*C*-4-Ph), 136.3 (4′-*C*-2-Ph), 137.2 (3-Ar*C*H), 148.8 (4-Ar*C*H), 152.1 (2-Ar*C*), 197.9 (4-*C*=O), 199.7 (1-*C*=O); **^{19}F NMR** (377 MHz, $CDCl_3$) δ_F: -115.5 (s, 4-*F*-2-Ph); **HRMS** (ESI^+) $C_{21}H_{17}FNO_2$ $[M+H]^+$ found 334.1238, requires 334.1243 (–1.8 ppm).

2-(4-chlorophenyl)-4-phenyl-1-(pyridin-2-yl)butane-1,4-dione 38

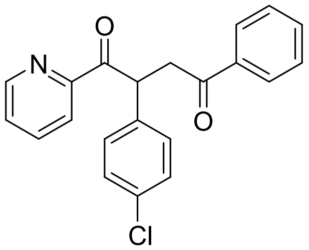

Colorless oil; Yield, 61%; R_f = 0.38 (petroleum ether:ethyl acetate, 5:1); **IR** ν_{max} (film): 3055, 2909, 1678, 1489, 1227, 995; **^{1}H NMR** (400 MHz, CD_2Cl_2) δ_H: 3.50 (1H, dd, *J* 18.1, 3.8, CH_AH$_B$), 4.20 (1H, dd, *J* 18.1, 10.9, CH$_A$$H_B$), 6.03 (1H, dd, *J* 10.8, 3.8, C*H*), 7.29-7.32 (2H, m, 3′,5′-*H*-2-Ph), 7.45-7.54 (5H, m, 5-Ar*H* + 2′,6′-*H*-2-Ph + 2′,6′-*H*-4-Ph), 7.60-7.65 (1H, m, 4′-*H*-4-Ph), 7.86 (1H, td, *J* 7.7, 1.7, 3-Ar*H*), 7.99-8.02 (2H, m, 3′,5′-*H*-4-Ph), 8.04-8.07 (1H, m, 6-Ar*H*), 8.76 (1H, ddd, *J* 4.8, 1.8, 0.9, 4-Ar*H*). **^{13}C{^{1}H} NMR** (101 MHz, CD_2Cl_2) δ_C: 16.1 (*C*H_2), 45.3 (*C*H), 122.6 (6-Ar*C*H), 127.2 (5-Ar*C*H), 128.0 (3′,5′-*C*H-4-Ph), 128.6 (3′,5′-*C*H-2-Ph), 128.7 (2′, 6′-*C*H-4-Ph), 130.4 (2′,6′-*C*H-2-Ph), 132.8 (1′-*C*-2-Ph), 133.3 (4′-*C*H-4-Ph), 136.4 (1′-*C*-4-Ph), 137.0 (4′-*C*-2-Ph), 137.0 (3-Ar*C*H), 149.0 (4-Ar*C*H), 152.4 (2-Ar*C*), 197.7 (4-*C*=O), 199.5 (1-*C*=O); **HRMS** (ESI^+) $C_{21}H_{17}ClNO_2$ $[M+H]^+$ found 350.0937, requires 350.0948 (–1.5 ppm).

2-(4-bromophenyl)-4-phenyl-1-(pyridin-2-yl)butane-1,4-dione 39

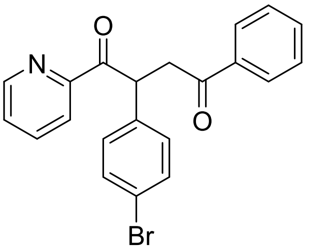

Colorless oil; Yield, 54%; R_f = 0.23 (petroleum ether:ethyl acetate, 5:1); **IR** ν_{max} (film): 3053, 2905, 1694, 1678, 1485, 1227, 995; **^{1}H NMR** (400 MHz, $CDCl_3$) δ_H: 3.45 (1H, dd, *J* 18.1, 3.8, CH_AH$_B$), 4.19 (1H, dd, *J* 18.1, 10.8, CH$_A$$H_B$), 6.05 (1H, dd, *J* 10.8, 3.8, C*H*), 7.38-7.49 (7H, m, 3′,5′-*H*-2-Ph + 5-Ar*H* + 2′,6′-*H*-2-Ph + 3′,5′-*H*-4-Ph), 7.56-7.61 (1H, m, 4′-*H*-4-Ph), 7.83 (1H, td, *J* 7.7, 1.8, 3-Ar*H*), 7.98-8.01 (2H, m, 2′,6′-*H*-4-

Ph), 8.07 (1H, dt, *J* 7.9, 1.1, 6-Ar*H*), 8.74 (1H, ddd, *J* 4.8, 1.8, 0.9, 4-Ar*H*). **^{13}C{^{1}H} NMR** (101 MHz, $CDCl_3$) δ_C: 16.3 (*C*H_2), 45.4 (*C*H), 121.2 (4′-*C*-2-Ph), 123.0 (6-Ar*C*H), 127.2 (5-Ar*C*H), 128.2 (2′,6′-*C*H-4-Ph), 128.6 (3′,5′-*C*H-4-Ph), 130.7 (3′,5′-*C*H-2-Ph), 131.9 (2′,6′-*C*H-2-Ph), 133.3 (4′-*C*H-4-Ph), 136.3 (1′-*C*-4-Ph), 137.1 (1′-*C*-2-Ph), 137.2 (3-Ar*C*H), 148.9 (4-Ar*C*H), 152.2 (2-Ar*C*), 197.8 (4-*C*=O), 199.5 (1-*C*=O); **HRMS** (ESI$^+$) $C_{21}H_{17}BrNO_2$ $[M+H]^+$ found 394.0429, requires 394.0443 (–2.2 ppm).

4-phenyl-1-(pyridin-2-yl)-2-(4-(trifluoromethyl)phenyl)butane-1,4-dione 40

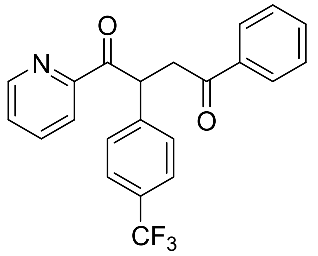

White solid; Yield, 71%; R_f = 0.39 (petroleum ether:ethyl acetate, 5:1); **mp**, 102-104 °C. **IR** ν_{max} (film): 3053, 2905, 1694, 1672, 1325, 1107, 845; **^{1}H NMR** (400 MHz, $CDCl_3$) δ_H: 3.48 (1H, dd, *J* 18.1, 3.8, C*H$_A$*H$_B$), 4.23 (1H, dd, *J* 18.1, 10.8, CH$_A$*H$_B$*), 6.15 (1H, dd, *J* 10.8, 3.8, C*H*), 7.45-7.50 (3H, m, 5-Ar*H* + 3′,5′-*H*-4-Ph), 7.56-7.66 (5H, m, 3′,5′-*H*-2-Ph + 2′,6′-*H*-2-Ph + 4′-*H*-4-Ph), 7.82 (1H, td, *J* 7.7, 1.7, 3-Ar*H*), 7.99-8.02 (2H, m, 2′,6′-*H*-4-Ph), 8.07 (1H, dt, *J* 7.8, 1.1, 6-Ar*H*), 8.74 (1H, ddd, *J* 4.8, 1.7, 0.9, 4-Ar*H*). **^{13}C{^{1}H} NMR** (101 MHz, $CDCl_3$) δ_C: 16.3 (*C*H_2), 45.8 (*C*H), 122.9 (6-Ar*C*H), 125.7 (q, 3′,5′-*C*H-2-Ph), 127.3 (5-Ar*C*H), 128.2 (2′,6′-*C*H-4-Ph), 128.7 (3′,5′-*C*H-4-Ph), 129.0 (m, *C*F_3), 129.3 (2′,6′-*C*H-2-Ph), 133.4 (4′-*C*H-4-Ph), 136.2 (1′-*C*-4-Ph), 136.9 (3-Ar*C*H), 142.4 (1′-*C*-2-Ph), 149.1 (4-Ar*C*H), 152.2 (2-Ar*C*), 197.6 (4-*C*=O), 199.4 (1-*C*=O); **^{19}F NMR** (377 MHz, $CDCl_3$) δ_F: -62.6 (s, C*F_3*); **HRMS** (ESI$^+$) $C_{22}H_{17}F_3NO_2$ $[M+H]^+$ found 384.1206, requires 384.1211 (–1.8 ppm).

4-(1,4-dioxo-4-phenyl-1-(pyridin-2-yl)butan-2-yl)benzonitrile 41

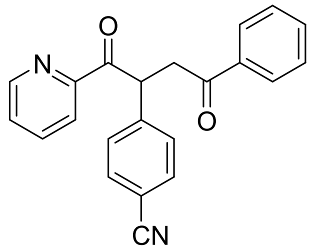

Yellow solid; Yield, 85%; R_f = 0.41 (petroleum ether:ethyl acetate, 2:1); **mp**, 95-99 °C. **IR** ν_{max} (film): 3057, 2920, 2228, 1697, 1674, 1445, 1229, 993; **^{1}H NMR** (400 MHz, $CDCl_3$) δ_H: 3.48 (1H, dd, *J* 18.0, 4.0,

CH_AH$_B$), 4.20 (1H, dd, *J* 18.1, 10.6, CH$_A$$H_B$), 6.13 (1H, dd, *J* 10.6, 4.0, C*H*), 7.16-7.49 (3H, m, 5-Ar*H* + 3′,5′-*H*-2-Ph), 7.57-7.66 (5H, m, 4′-*H*-4-Ph + 2′,6′-*H*-4-Ph + 3′,5′-*H*-4-Ph), 7.82 (1H, td, *J* 7.7, 1.7, 3-Ar*H*), 7.98-8.01 (2H, m, 2′,6′-*H*-2-Ph), 8.07 (1H, dt, *J* 7.8, 1.1, 6-Ar*H*), 8.72 (1H, ddd, *J* 4.8, 1.7, 0.9, 4-Ar*H*). **^{13}C{^{1}H} NMR** (101 MHz, $CDCl_3$) δ_C: 16.0 (CH_2), 46.1 (*C*H), 111.0 (*C*N), 118.8 (4′-*C*-2-Ph), 122.9 (6-Ar*C*H), 127.4 (5-Ar*C*H), 128.2 (2′,6′-*C*H-2-Ph), 128.7 (3′,5′-*C*H-2-Ph), 129.8 (2′, 6′-*C*H-4-Ph), 132.5 (3′,5′-*C*H-4-Ph), 133.5 (4′-*C*H-4-Ph), 136.1 (1′-*C*-2-Ph), 137.0 (3-Ar*C*H), 143.9 (1′-*C*-4-Ph), 149.1 (4-Ar*C*H), 152.1 (2-Ar*C*), 197.4 (4-*C*=O), 199.1 (1-*C*=O); **HRMS** (ESI$^+$) $C_{22}H_{16}N_2O_2$ [M+Na]$^+$ found 363.1098, requires 363.1109 (–1.7 ppm).

4-(4-methoxyphenyl)-2-phenyl-1-(pyridin-2-yl)butane-1,4-dione 42

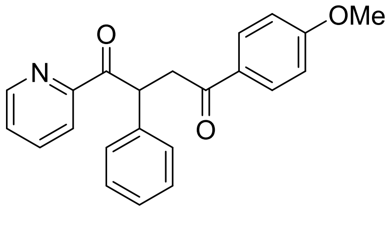

White solid; Yield, 36%; R_f = 0.17 (petroleum ether:ethyl acetate, 5:1); **mp**, 169-170 °C. **IR** ν_{max} (film): 3051, 2905, 1697, 1663, 1595, 1244, 1169, 995; **^{1}H NMR** (400 MHz, $CDCl_3$) δ_H: 3.44 (1H, dd, *J* 17.9, 3.6, CH_AH$_B$), 3.88 (3H, s, CH_3), 4.20 (1H, dd, *J* 18.0, 11.1, CH$_A$$H_B$), 6.07 (1H, dd, *J* 11.1, 3.5, C*H*), 6.91-6.95 (2H, m, 3′,5′-*H*-4-Ph), 7.20-7.24 (1H, m, 4′-*H*-2-Ph), 7.28-7.33 (2H, m, 2′,6′-*H*-2-Ph), 7.44 (1H, ddd, *J* 7.6, 4.8, 1.3, 5-Ar*H*), 7.50-7.54 (2H, m, 3′,5′-*H*-2-Ph), 7.80 (1H, td, *J* 7.7, 1.8, 3-Ar*H*), 7.96-8.00 (2H, m, 2′,6′-*H*-4-Ph), 8.07 (1H, dt, *J* 7.8, 1.1, 6-Ar*H*), 8.75 (1H, ddd, *J* 4.8, 1.7, 0.9, 4-Ar*H*). **^{13}C{^{1}H} NMR** (101 MHz, $CDCl_3$) δ_C: 16.5 (CH_2), 45.9 (*C*H), 55.5 (CH_3), 113.7 (3′,5′-*C*H-4-Ph), 122.9 (6-Ar*C*H), 127.0 (5-Ar*C*H), 127.1 (4′-*C*H-2-Ph), 128.7 (2′,6′-*C*H-2-Ph), 128.9 (3′,5′-*C*H-2-Ph), 129.5 (1′-*C*-4-Ph), 130.5 (2′,6′-*C*H-4-Ph), 137.0 (3-Ar*C*H), 138.2 (1′-*C*-2-Ph), 148.9 (4-Ar*C*H), 152.5 (2-Ar*C*), 163.6 (4′-*C*-4-Ph), 196.6 (4-*C*=O), 200.0 (1-*C*=O); **HRMS** (ESI$^+$) $C_{22}H_{20}NO_3$ [M+H]$^+$ found 346.1425, requires 346.1443 (–3.7 ppm).

2-phenyl-1-(pyridin-2-yl)-4-(p-tolyl)butane-1,4-dione 43

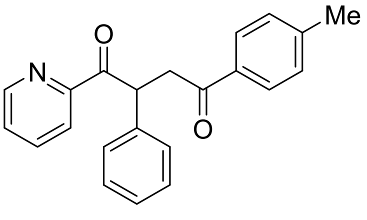

White solid; Yield, 38%; R_f = 0.18 (petroleum ether:ethyl acetate, 5:1); **mp**, 104-106 °C. **IR** ν_{max} (film):

3036, 2905, 1695, 1674, 1607, 1231, 995; **^{1}H NMR** (500 MHz, $CDCl_3$) δ_H: 2.42 (3H, s, C*H$_3$*), 3.46 (1H, dd, *J* 18.1, 3.6, C*H$_A$*H$_B$), 4.23 (1H, dd, *J* 18.1, 11.1, CH$_A$*H$_B$*), 6.08 (1H, dd, *J* 11.1, 3.5, C*H*), 7.21-7.27 (3H, m, 4′-*H*-2-Ph + 3′,5′-*H*-4-Ph), 7.30-7.33 (2H, m, 2′,6′-*H*-2-Ph), 7.42 (1H, ddd, *J* 7.6, 4.8, 1.3, 5-Ar*H*), 7.51-7.53 (2H, m, 3′,5′-*H*-2-Ph), 7.79 (1H, td, *J* 7.7, 1.8, 3-Ar*H*), 7.90-7.91 (2H, m, 2′,6′-*H*-4-Ph), 8.07 (1H, dd, *J* 7.9, 1.2, 6-Ar*H*), 8.75 (1H, dt, *J* 4.8, 1.2, 4-Ar*H*). **^{13}C{^{1}H} NMR** (126 MHz, $CDCl_3$) δ_C: 21.7 (*C*H$_3$), 16.6 (*C*H$_2$), 45.8 (*C*H), 122.8 (6-Ar*C*H), 126.9 (5-Ar*C*H), 127.1 (4′-*C*H-2-Ph), 128.3 (3′,5′-*C*H-4-Ph), 128.7 (2′,6′-*C*H-2-Ph), 128.9 (3′,5′-*C*H-2-Ph), 129.3 (2′,6′-*C*H-4-Ph), 134.0 (1′-*C*-4-Ph), 136.7 (3-Ar*C*H), 138.3 (1′-*C*-2-Ph), 144.0 (4′-*C*-4-Ph), 149.1 (4-Ar*C*H), 152.7 (2-Ar*C*), 197.8 (4-*C*=O), 200.2 (1-*C*=O); **HRMS** (ESI$^+$) $C_{22}H_{20}NO_2$ $[M+H]^+$ found 330.1478, requires 330.1494 (–3.1 ppm).

4-(4-fluorophenyl)-2-phenyl-1-(pyridin-2-yl)butane-1,4-dione 44

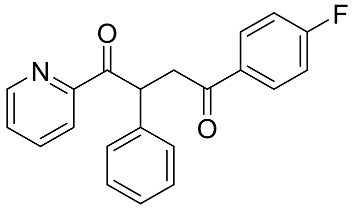

White solid; Yield, 54%; R_f = 0.21 (petroleum ether:ethyl acetate, 5:1); **mp**, 109-111 °C. **IR** ν_{max} (film): 3063, 2909, 1695, 1682, 1595, 1229, 995; **^{1}H NMR** (400 MHz, $CDCl_3$) δ_H: 3.42 (1H, dd, *J* 18.0, 3.6, C*H$_A$*H$_B$), 4.21 (1H, dd, *J* 18.0, 11.1, CH$_A$*H$_B$*), 6.07 (1H, dd, *J* 11.1, 3.5, C*H*), 7.11-7.16 (2H, m, 3′,5′-*H*-4-Ph), 7.20-7.25 (1H, m, 4′-*H*-2-Ph), 7.28-7.34 (2.35H, m, Solvent + 2′,6′-*H*-2-Ph), 7.16 (1H, ddd, *J* 7.6, 4.7, 1.2, 5-Ar*H*), 7.48-7.51 (2H, m, 3′,5′-*H*-2-Ph), 7.79 (1H, td, *J* 7.7, 1.8, 3-Ar*H*), 8.00-8.08 (3H, m, 2′,6′-*H*-4-Ph + 6-Ar*H*), 8.74 (1H, ddd, *J* 4.7, 1.7, 0.9, 4-Ar*H*). **^{13}C{^{1}H} NMR** (101 MHz, $CDCl_3$) δ_C: 16.5 (*C*H$_2$), 45.9 (*C*H), 115.6 (3′,5′-*C*H-4-Ph), 115.8 (3′,5′-*C*H-4-Ph), 122.8 (6-Ar*C*H), 127.0 (5-Ar*C*H), 127.2 (4′-*C*H-2-Ph), 128.8 (2′,6′-*C*H-2-Ph), 128.9 (3′,5′-*C*H-2-Ph), 130.8 (2′,6′-*C*H-4-Ph), 130.9 (2′,6′-*C*H-4-Ph), 132.9 (1′-*C*-4-Ph), 132.9 (1′-*C*-4-Ph), 136.8 (3-Ar*C*H), 138.0 (1′-*C*-2-Ph), 149.1 (4-Ar*C*H), 152.6 (2-Ar*C*), 164.6 (4′-*C*-4-Ph), 167.1 (4′-*C*-4-Ph), 196.6 (4-*C*=O), 200.0 (1-*C*=O); **^{19}F NMR** (377 MHz, $CDCl_3$) δ_F: -105.0 (s, 4-*F*-4-Ph); **HRMS** (ESI$^+$) $C_{21}H_{17}FNO_2$ $[M+H]^+$ found 334.1223, requires 334.1243 (–4.4 ppm).

4-(4-chlorophenyl)-2-phenyl-1-(pyridin-2-yl)butane-1,4-dione 45

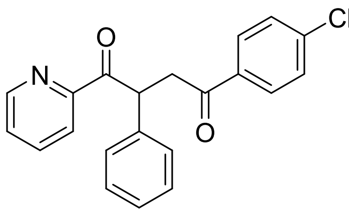

White solid; Yield, 72%; R_f = 0.26 (petroleum ether:ethyl acetate, 5:1); **mp**, 136-137 °C. **IR** ν_{max} (film): 3057, 2903, 1695, 1680, 1587, 1231, 995; **^{1}H NMR** (400 MHz, $CDCl_3$) δ_H: 3.41 (1H, dd, *J* 18.1, 3.6, C*H_A*H_B), 4.20 (1H, dd, *J* 18.1, 11.0, CH_A*H_B*), 6.07 (1H, dd, *J* 11.0, 3.6, C*H*), 7.20-7.25 (1H, m, 4′-*H*-2-Ph), 7.28-7.33 (2.57H, m, Solvent + 2′,6′-*H*-2-Ph), 7.42-7.45 (3H, m, 3′,5′-*H*-4-Ph + 5-Ar*H*), 7.48-7.51 (2H, m, 3′,5′-*H*-2-Ph), 7.79 (1H, td, *J* 7.7, 1.7, 3-Ar*H*), 7.93-7.96 (2H, m, 2′,6′-*H*-4-Ph), 8.06 (1H, dt, *J* 7.8, 1.1, 6-Ar*H*), 8.74 (1H, ddd, *J* 4.8, 1.7, 0.9, 4-Ar*H*). **^{13}C{^{1}H} NMR** (101 MHz, $CDCl_3$) δ_C: 16.5 (*C*H_2), 45.9 (*C*H), 122.8 (6-Ar*C*H), 127.0 (5-Ar*C*H), 127.2 (4′-*C*H-2-Ph), 128.8 (2′,6′-*C*H-2-Ph), 128.9 (3′,5′-*C*H-2-Ph), 128.9 (3′,5′-*C*H-4-Ph), 129.6 (2′,6′-*C*H-4-Ph), 134.8 (1′-*C*-4-Ph), 136.8 (3-Ar*C*H), 138.0 (1′-*C*-2-Ph), 139.7 (4′-*C*-4-Ph), 149.1 (4-Ar*C*H), 152.5 (2-Ar*C*), 197.0 (4-*C*=O), 200.0 (1-*C*=O); **HRMS** (ESI$^+$) $C_{21}H_{17}ClNO_2$ $[M+H]^+$ found 350.0942, requires 350.0948 (–3.0 ppm).

4-(4-bromophenyl)-2-phenyl-1-(pyridin-2-yl)butane-1,4-dione 46

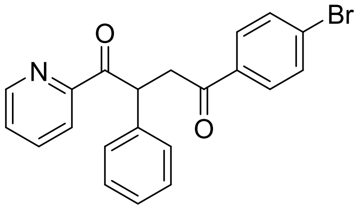

White solid; Yield, 65%; R_f = 0.36 (petroleum ether:ethyl acetate, 5:1); **mp**, 143-144 °C. **IR** ν_{max} (film): 3055, 2901, 1695, 1676, 1582, 1231, 995; **^{1}H NMR** (400 MHz, $CDCl_3$) δ_H: 3.41 (1H, dd, *J* 18.1, 3.6, C$H_A$$H_B$), 4.19 (1H, dd, *J* 18.1, 11.0, C$H_A$$H_B$), 6.06 (1H, dd, *J* 11.0, 3.5, C*H*), 7.20-7.25 (1H, m, 4′-*H*-2-Ph), 7.28-7.33 (2.51H, m, Solvent + 2′,6′-*H*-2-Ph), 7.16 (1H, ddd, *J* 7.6, 4.8, 1.3, 5-Ar*H*), 7.48-7.50 (2H, m, 3′,5′-*H*-2-Ph), 7.59-7.63 (2H, m, 3′,5′-*H*-4-Ph), 7.79 (1H, td, *J* 7.7, 1.8, 3-Ar*H*), 7.85-7.88 (2H, m, 2′,6′-*H*-4-Ph), 8.06 (1H, dt, *J* 7.9, 1.1, 6-Ar*H*), 8.74 (1H, ddd, *J* 4.7, 1.7, 0.9, 4-Ar*H*). **^{13}C{^{1}H} NMR** (101 MHz, $CDCl_3$) δ_C: 16.5 (*C*H_2), 45.8 (*C*H), 122.8 (6-Ar*C*H), 127.0 (5-Ar*C*H), 127.2 (4′-*C*H-2-Ph), 128.4 (1′-*C*-4-Ph), 128.8 (2′,6′-*C*H-2-Ph), 128.9 (3′,5′-*C*H-2-Ph), 129.7 (2′,6′-*C*H-4-Ph), 131.9 (3′,5′-*C*H-4-Ph), 135.2 (4′-*C*-4-Ph), 136.8 (3-Ar*C*H), 138.0 (1′-*C*-2-Ph), 149.1 (4-Ar*C*H), 152.5 (2-Ar*C*), 197.2 (4-*C*=O), 200.0 (1-*C*=O); **HRMS** (ESI$^+$) $C_{21}H_{17}BrNO_2$ $[M+H]^+$ found 394.0437, requires 394.0443 (–3.6 ppm).

2-phenyl-1-(pyridin-2-yl)-4-(4-(trifluoromethyl)phenyl)butane-1,4-dione 47

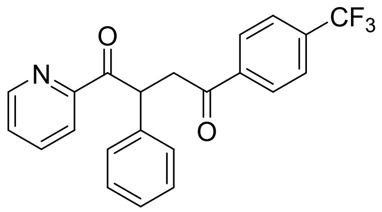

White solid; Yield, 88%; R_f = 0.16 (petroleum ether:ethyl acetate, 5:1); **mp**, 85-86 °C. **IR** ν_{max} (film): 3061, 2909, 1686, 1319, 1065, 845; **^{1}H NMR** (400 MHz, $CDCl_3$) δ_H: 3.46 (1H, dd, *J* 18.2, 3.6, C$H_A$$H_B$), 4.25 (1H, dd, *J* 18.2, 10.9, CH$_A$$H_B$), 6.09 (1H, dd, *J* 11.0, 3.6, C*H*), 7.21-7.26 (1H, m, 4′-*H*-2-Ph), 7.28-7.34 (2H, m, 2′,6′-*H*-2-Ph), 7.45 (1H, ddd, *J* 7.5, 4.8, 1.3, 5-Ar*H*), 7.48-7.51 (2H, m, 3′,5′-*H*-2-Ph), 7.73-7.75 (2H, m, 3′,5′-*H*-4-Ph), 7.81 (1H, td, *J* 7.7, 1.7, 3-Ar*H*), 8.06-8.12 (3H, m, 6-Ar*H* + 2′,6′-*H*-4-Ph), 8.74 (1H, ddd, *J* 4.8, 1.8, 0.9, 4-Ar*H*). **^{13}C{^{1}H} NMR** (101 MHz, $CDCl_3$) δ_C: 16.7 (*C*H_2), 45.9 (*C*H), 122.2 (q, 1′-*C*-4-Ph), 122.9 (6-Ar*C*H), 125.7 (q, 3′,5′-*C*H-4-Ph), 127.1 (5-Ar*C*H), 127.3 (4′-*C*H-2-Ph), 128.5 (2′,6′-*C*H-4-Ph), 128.8 (2′,6′-*C*H-2-Ph), 128.9 (3′,5′-*C*H-2-Ph), 134.7 (q, *C*F_3), 136.9 (3-Ar*C*H), 137.8 (1′-*C*-2-Ph), 139.1 (4′-*C*-4-Ph), 149.0 (4-Ar*C*H), 152.4 (2-Ar*C*), 197.3 (4-*C*=O), 199.8 (1-*C*=O); **^{19}F NMR** (377 MHz, $CDCl_3$) δ_F: -63.1 (s, CF_3); **HRMS** (ESI$^+$) $C_{22}H_{17}F_3NO_2$ $[M+H]^+$ found 384.1192, requires 384.1211 (–3.7 ppm).

4-(4-oxo-3-phenyl-4-(pyridin-2-yl)butanoyl)benzonitrile 48

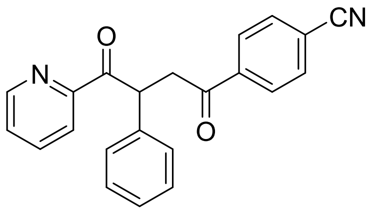

White solid; Yield, 92%; R_f = 0.55 (petroleum ether:ethyl acetate, 2:1); **mp**, 145-150 °C. **IR** ν_{max} (film): 3057, 2905, 2226, 1686, 1398,1231, 995; **^{1}H NMR** (400 MHz, $CDCl_3$) δ_H: 3.42 (1H, dd, *J* 18.2, 3.6, C$H_A$$H_B$), 4.22 (1H, dd, *J* 18.2, 10.9, CH$_A$$H_B$), 6.08 (1H, dd, *J* 10.9, 3.6, C*H*), 7.20-7.25 (1H, m, 4′-*H*-2-Ph), 7.28-7.33 (2.18H, m, Solvent + 2′,6′-*H*-2-Ph), 7.16 (1H, ddd, *J* 7.5, 4.7, 1.2, 5-Ar*H*), 7.46-7.49 (2H, m, 3′,5′-*H*-2-Ph), 7.75-7.81 (3H, m, 3-Ar*H* + 3′,5′-*H*-4-Ph), 8.04-8.09 (3H, m, 6-Ar*H* + 2′,6′-*H*-4-Ph), 8.73 (1H, ddd, *J* 4.8, 1.8, 0.9, 4-Ar*H*). **^{13}C{^{1}H} NMR** (101 MHz, $CDCl_3$) δ_C: 16.6 (*C*H_2), 46.0 (*C*H), 17.5 (*C*N), 118.0 (4′-*C*-4-Ph), 122.9 (6-Ar*C*H), 127.1 (5-Ar*C*H), 127.3 (4′-*C*H-2-Ph), 128.6 (2′,6′-*C*H-4-Ph), 128.9 (3′,5′-*C*H-2-Ph + 2′,6′-*C*H-2-Ph), 132.5 (3′,5′-*C*H-4-Ph), 136.8 (3-Ar*C*H), 137.7 (1′-*C*-2-Ph), 139.4 (1′-*C*-4-Ph), 149.1 (4-Ar*C*H), 152.4 (2-Ar*C*), 196.9 (4-*C*=O), 199.8 (1-*C*=O); **HRMS** (ESI$^+$) $C_{22}H_{16}N_2O_2$ $[M+Na]^+$ found

363.1097, requires 363.1109 (–1.9 ppm).

2,4-bis(4-methoxyphenyl)-1-(pyridin-2-yl)butane-1,4-dione 60

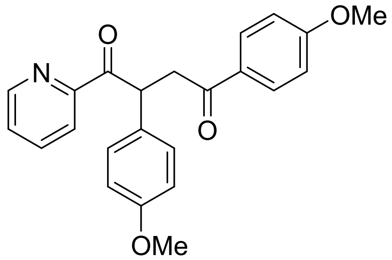

White solid; Yield, 29%; R_f = 0.35 (petroleum ether:ethyl acetate, 2:1); **mp**, 130-132 °C. **IR** ν_{max} (film): 3032, 2902, 2839, 1695, 1670, 1595, 1510, 1233, 1167, 995; **^{1}H NMR** (400 MHz, $CDCl_3$) δ_H: 3.40 (1H, dd, *J* 17.9, 3.7, C*H*H_AH$_B$), 3.77 (3H, s, OC*H*$_3$-4-Ph), 3.88 (3H, s, OC*H*$_3$-2-Ph), 4.16 (1H, dd, *J* 17.9, 11.0, CH$_A$*H*$_B$), 6.01 (1H, dd, *J* 11.0, 3.7, C*H*), 6.82-6.86 (2H, m, 3′,5′-*H*-4-Ph), 6.91-6.95 (2H, m, 3′,5′-*H*-2-Ph), 7.40-7.45 (3H, m, 5-Ar*H* + 2′,6′-*H*-4-Ph), 7.78 (1H, td, *J* 7.7, 1.7, 3-Ar*H*), 7.96-8.00 (2H, m, 2′,6′-*H*-2-Ph), 8.06 (1H, dt, *J* 7.8, 1.1, 6-Ar*H*), 8.74 (1H, ddd, *J* 4.8, 1.8, 0.9, 4-Ar*H*). **^{13}C{^{1}H} NMR** (101 MHz, $CDCl_3$) δ_C: 16.3 (*C*H$_2$), 44.9 (*C*H), 55.2 (O*C*H$_3$-4-Ph), 55.5 (O*C*H$_3$-2-Ph), 113.7 (3′,5′-*C*H-2-Ph), 114.1 (3′,5′-*C*H-4-Ph), 122.8 (6-Ar*C*H), 126.8 (5-Ar*C*H), 129.6 (1′-*C*-4-Ph), 130.0 (2′,6′-*C*H-4-Ph), 130.1 (1′-*C*-2-Ph), 130.4 (2′,6′-*C*H-2-Ph), 136.7 (3-Ar*C*H), 149.0 (4-Ar*C*H), 152.8 (2-Ar*C*), 158.7 (4′-*C*-4-Ph), 163.5 (4′-*C*-2-Ph), 196.8 (4-*C*=O), 200.3 (1-*C*=O); **HRMS** (ESI$^+$) $C_{23}H_{21}NO_4$ $[M+Na]^+$ found 398.1353, requires 398.1368 (–2.4 ppm).

1-(pyridin-2-yl)-2,4-di-p-tolylbutane-1,4-dione 61

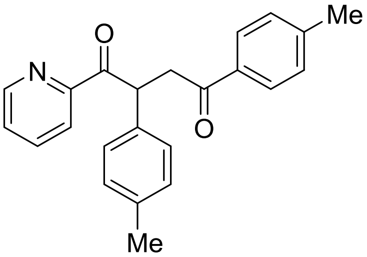

White solid; Yield, 31%; R_f = 0.20 (petroleum ether:ethyl acetate, 5:1); **mp**, 94-95 °C. **IR** ν_{max} (film): 3053, 2918, 1694, 1674, 1337, 1228, 995; **^{1}H NMR** (400 MHz, $CDCl_3$) δ_H: 2.30 (3H, s, 4′-C*H*$_3$-2-Ph), 2.42 (3H, s, 4′-C*H*$_3$-4-Ph), 3.16 (1H, dd, *J* 18.1, 3.6, C*H*$_A$H$_B$), 4.21 (1H, dd, *J* 18.1, 11.1, CH$_A$*H*$_B$), 6.03 (1H, dd, *J* 11.1, 3.5, C*H*), 7.11-7.13 (2H, m, 3′,5′-*H*-2-Ph), 7.25-7.28 (2.37H, m, Solvent + 3′,5′-*H*-4-Ph), 7.39-7.46 (3H, m, 5-Ar*H* + 2′,6′-*H*-2-Ph), 7.80 (1H, td, *J* 7.7, 1.7, 3-Ar*H*), 7.88-7.91 (2H, m, 2′,6′-*H*-4-Ph), 8.07 (1H, dt, *J* 7.9, 1.1, 6-Ar*H*), 8.75 (1H, ddd, *J* 4.8, 1.8, 0.9, 4-Ar*H*). **^{13}C{^{1}H} NMR** (101 MHz, $CDCl_3$) δ_C: 21.1 (4′-*C*H$_3$-2-

Ph), 21.7 (4′-CH_3-4-Ph), 16.7 (CH_2), 45.4 (*C*H), 122.9 (6-Ar*C*H), 126.9 (5-Ar*C*H), 128.3 (2′,6′-*C*H-4-Ph), 128.8 (2′,6′-*C*H-2-Ph), 129.2 (3′,5′-*C*H-4-Ph), 129.5 (3′,5′-*C*H-2-Ph), 134.0 (1′-*C*-4-Ph), 135.0 (1′-*C*-2-Ph), 136.8 (4′-*C*-2-Ph), 137.0 (3-Ar*C*H), 144.0 (4′-*C*-4-Ph), 148.9 (4-Ar*C*H), 152.5 (2-Ar*C*), 197.8 (4-*C*=O), 200.0 (1-*C*=O); **HRMS** (ESI^+) $C_{23}H_{22}NO_2$ $[M+H]^+$ found 344.1637, requires 344.1651 (–2.2 ppm).

4-(4-methoxyphenyl)-1-(pyridin-2-yl)-2-(4-(trifluoromethyl)phenyl)butane-1,4-dione 62

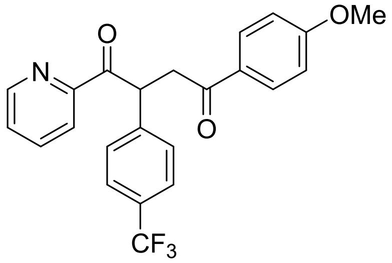

White solid; Yield, 74%; R_f = 0.13 (petroleum ether:ethyl acetate, 5:1); **mp**, 89-90 °C. **IR** ν_{max} (film): 3055, 2913, 1697, 1670, 1599, 1321, 1109, 833; **^{1}H NMR** (400 MHz, $CDCl_3$) δ_H: 3.44 (1H, dd, *J* 17.9, 3.9, CH_AH_B), 3.89 (3H, s, OCH_3), 4.18 (1H, dd, *J* 17.9, 10.8, CH_AH_B), 6.14 (1H, dd, *J* 10.8, 3.8, C*H*), 6.92-6.96 (2H, m, 3′,5′-*H*-4-Ph), 7.45 (1H, ddd, *J* 7.6, 4.8, 1.3, 5-Ar*H*), 7.54-7.59 (2H, m, 2′,6′-*H*-2-Ph), 7.63-7.66 (2H, m, 3′,5′-*H*-2-Ph), 7.82 (1H, td, *J* 7.7, 1.7, 3-Ar*H*), 7.96-8.00 (2H, m, 2′,6′-*H*-4-Ph), 8.07 (1H, dt, *J* 7.9, 1.1, 6-Ar*H*), 8.74 (1H, ddd, *J* 4.8, 1.8, 0.9, 4-Ar*H*). **^{13}C{^{1}H} NMR** (101 MHz, $CDCl_3$) δ_C: 16.0 (CH_2), 45.8 (*C*H), 55.5 (OCH_3), 113.8 (3′,5′-*C*H-4-Ph), 122.8 (6-Ar*C*H), 125.6 (q, 2′,6′-CH-2-Ph), 127.2 (5-Ar*C*H), 129.1 (m, CF_3), 129.3 (m, 3′,5′-*C*H-2-Ph), 130.5 (2′,6′-*C*H-4-Ph), 136.9 (1′-*C*-4-Ph), 142.6 (1′-*C*-2-Ph), 149.1 (4-Ar*C*H), 152.3 (2-Ar*C*), 163.7 (4′-*C*-4-Ph), 196.1 (4-*C*=O), 199.6 (1-*C*=O); **^{19}F NMR** (377 MHz, $CDCl_3$) δ_F: -62.6 (s, CF_3); **HRMS** (ESI^+) $C_{23}H_{19}F_3NO_3$ $[M+H]^+$ found 414.1312, requires 414.1317 (–1.3 ppm).

4-(4-(4-methoxyphenyl)-1,4-dioxo-1-(pyridin-2-yl)butan-2-yl)benzonitrile 63

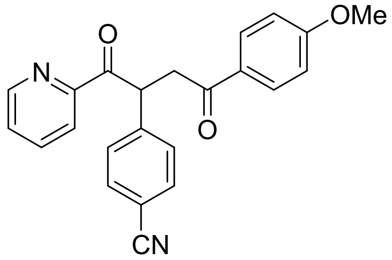

White solid; Yield, 74%; R_f = 0.21 (petroleum ether:ethyl acetate, 2:1); **mp**, 131-132 °C. **IR** ν_{max} (film): 3061, 2903, 2845, 2226, 1695, 1673, 1595, 1167, 835; **^{1}H NMR** (400 MHz, $CDCl_3$) δ_H: 3.16 (1H, dd, *J* 17.8, 4.0, CH_AH_B), 3.88 (3H, s, OCH_3), 4.15 (1H, dd, *J* 17.8, 10.6, CH_AH_B), 6.11 (1H, dd, *J* 10.6, 4.0, C*H*), 6.92-

6.95 (2H, m, 3′,5′-*H*-4-Ph), 7.46 (1H, ddd, *J* 7.6, 4.8, 1.3, 5-Ar*H*), 7.57-7.60 (2H, m, 2′,6′-*H*-2-Ph), 7.62-7.65 (2H, m, 3′,5′-*H*-2-Ph), 7.82 (1H, td, *J* 7.7, 1.7, 3-Ar*H*), 7.94-7.98 (2H, m, 2′,6′-*H*-4-Ph), 8.07 (1H, dt, *J* 7.9, 1.1, 6-Ar*H*), 8.73 (1H, ddd, *J* 4.8, 1.7, 0.9, 4-Ar*H*). **$^{13}C\{^1H\}$ NMR** (101 MHz, $CDCl_3$) δ_C: 42.7 (CH_2), 46.1 (*C*H), 55.5 (OCH_3), 111.0 (*C*N), 113.8 (3′,5′-*C*H-4-Ph), 118.8 (4′-*C*-2-Ph), 122.9 (6-Ar*C*H), 127.3 (5-Ar*C*H), 129.2 (1′-*C*-4-Ph), 129.8 (3′,5′-*C*H-2-Ph), 130.5 (2′,6′-*C*H-4-Ph), 132.4 (2′,6′-*C*H-2-Ph), 136.9 (3-Ar*C*H), 144.0 (1′-*C*-2-Ph), 149.1 (4-Ar*C*H), 152.2 (2-Ar*C*), 163.8 (4′-*C*-4-Ph), 195.8 (4-*C*=O), 199.2 (1-*C*=O); **HRMS** (ESI^+) $C_{23}H_{18}N_2O_3$ $[M+Na]^+$ found 393.1209, requires 393.1215 (–0.9 ppm).

2-(4-chlorophenyl)-1-(pyridin-2-yl)-4-(p-tolyl)butane-1,4-dione 64

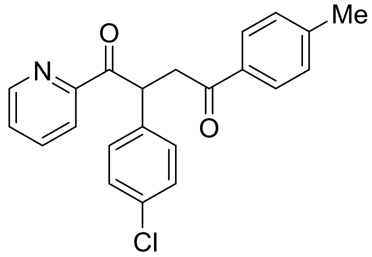

White solid; Yield, 44%; R_f = 0.26 (petroleum ether:ethyl acetate, 5:1); **mp**, 151-152 °C. **IR** ν_{max} (film): 3051, 2920, 1697, 1682, 1339, 1229, 995; **^1H NMR** (400 MHz, $CDCl_3$) δ_H: 2.42 (3H, s, CH_3), 3.16 (1H, dd, *J* 18.0, 3.8, CH_AH_B), 4.17 (1H, dd, *J* 18.0, 10.8, CH_AH_B), 6.04 (1H, dd, *J* 10.8, 3.8, C*H*), 7.25-7.29 (4.28H, m, Solvent + 3′,5′-*H*-2-Ph + 3′,5′-*H*-4-Ph), 7.16-7.47 (3H, m, 2′,6′-*H*-2-Ph + 5-Ar*H*), 7.81 (1H, td, *J* 7.7, 1.7, 3-Ar*H*), 7.88-7.91 (2H, m, 2′,6′-*H*-4-Ph), 8.06 (1H, dt, *J* 7.9, 1.1, 6-Ar*H*), 8.74 (1H, ddd, *J* 4.7, 1.8, 0.9, 4-Ar*H*). **$^{13}C\{^1H\}$ NMR** (101 MHz, $CDCl_3$) δ_C: 21.7 (CH_3), 16.3 (CH_2), 45.2 (*C*H), 122.9 (6-Ar*C*H), 127.1 (5-Ar*C*H), 128.3 (2′,6′-*C*H-4-Ph), 128.9 (3′,5′-*C*H-4-Ph), 129.3 (3′,5′-*C*H-2-Ph), 130.3 (2′,6′-*C*H-2-Ph), 133.0 (4′-*C*-2-Ph), 133.9 (1′-*C*-4-Ph), 136.8 (1′-*C*-2-Ph), 136.9 (3-Ar*C*H), 144.1 (4′-*C*-4-Ph), 149.0 (4-Ar*C*H), 152.4 (2-Ar*C*), 197.5 (4-*C*=O), 199.8 (1-*C*=O); **HRMS** (ESI^+) $C_{22}H_{19}ClNO_2$ $[M+H]^+$ found 364.1085, requires 364.1104 (–3.8 ppm).

2-(4-methoxyphenyl)-1-(pyridin-2-yl)-4-(4-(trifluoromethyl)phenyl)butane-1,4-dione 65

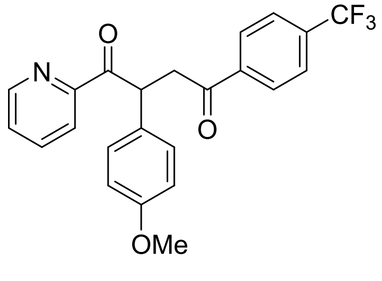

Colorless oil; Yield, 83%; R_f = 0.71 (petroleum ether:ethyl acetate, 2:1); **IR** ν_{max} (film): 3055, 2909, 2837, 1686, 1510, 1319, 1065, 831; **^{1}H NMR** (400 MHz, $CDCl_3$) δ_H: 3.16 (1H, dd, *J* 18.2, 3.8, C*H$_A$*H$_B$), 3.77 (3H, s, OC*H$_3$*), 4.21 (1H, dd, *J* 18.2, 10.8, CH$_A$*H$_B$*), 6.03 (1H, dd, *J* 10.8, 3.7, C*H*), 6.83-6.87 (2H, m, 3′,5′-*H*-2-Ph), 7.39-7.45 (3H, m, 5-Ar*H* + 2′,6′-*H*-2-Ph), 7.72-7.75 (2H, m, 2′,6′-*H*-4-Ph), 7.80 (1H, td, *J* 7.7, 1.7, 3-Ar*H*), 8.05 (1H, dt, *J* 7.8, 1.1, 6-Ar*H*), 8.09-8.12 (2H, m, 3′,5′-*H*-4-Ph), 8.74 (1H, ddd, *J* 4.8, 1.7, 0.9, 4-Ar*H*). **^{13}C{^{1}H} NMR** (101 MHz, $CDCl_3$) δ_C: 16.6 (*C*H$_2$), 45.0 (*C*H), 55.2 (O*C*H$_3$), 114.2 (3′,5′-*C*H-2-Ph), 122.3 (*C*F$_3$), 122.9 (6-Ar*C*H), 125.0 (4′-*C*-4-Ph), 125.7 (q, 2′,6′-*C*H-4-Ph), 127.0 (5-Ar*C*H), 128.5 (Quint, 3′,5′-*C*H-4-Ph), 129.6 (1′-*C*-2-Ph), 130.0 (2′,6′-*C*H-2-Ph), 134.3 (q, *C*F$_3$), 136.8 (3-Ar*C*H), 139.1 (1′-*C*-4-Ph), 149.1 (4-Ar*C*H), 152.5 (2-Ar*C*), 158.8(4′-*C*-2-Ph), 197.4 (4-*C*=O), 200.0 (1-*C*=O); **^{19}F NMR** (377 MHz, $CDCl_3$) δ_F: -63.1 (s, C*F$_3$*); **HRMS** (ESI$^+$) $C_{23}H_{18}F_3NO_3$ $[M+Na]^+$ found 436.1123, requires 436.1136 (–1.8 ppm).

4-(4-chlorophenyl)-1-(pyridin-2-yl)-2-(p-tolyl)butane-1,4-dione 66

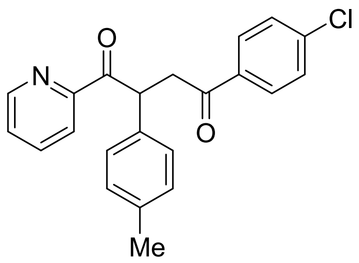

White solid; Yield, 58%; R_f = 0.25 (petroleum ether:ethyl acetate, 5:1); **mp**, 125-126 °C. **IR** ν_{max} (film): 3088, 2914, 1692, 1682, 1398, 1215, 993; **^{1}H NMR** (400 MHz, $CDCl_3$) δ_H: 2.30 (3H, s, C*H$_3$*), 3.39 (1H, dd, *J* 18.1, 3.6, C*H$_A$*H$_B$), 4.18 (1H, dd, *J* 18.2, 11.0, CH$_A$*H$_B$*), 6.02 (1H, dd, *J* 11.0, 3.6, C*H*), 7.10-7.13 (2H, m, 3′,5′-*H*-2-Ph), 7.36-7.39 (2H, m, 2′,6′-*H*-2-Ph), 7.41-7.45 (3H, m, 5-Ar*H* + 3′,5′-*H*-4-Ph), 7.79 (1H, td, *J* 7.7, 1.7, 3-Ar*H*), 7.92-7.95 (2H, m, 2′,6′-*H*-4-Ph), 8.05 (1H, dt, *J* 7.9, 1.1, 6-Ar*H*), 8.74 (1H, ddd, *J* 4.7, 1.8, 0.9, 4-Ar*H*). **^{13}C{^{1}H} NMR** (101 MHz, $CDCl_3$) δ_C: 21.1 (*C*H$_3$), 16.5 (*C*H$_2$), 45.4 (*C*H), 122.9 (6-Ar*C*H), 127.0 (5-Ar*C*H), 128.8 (2′,6′-*C*H-2-Ph), 128.9 (3′,5′-*C*H-4-Ph), 129.5 (3′,5′-*C*H-2-Ph), 129.6 (2′,6′-*C*H-2-Ph), 134.8 (1′-*C*-4-Ph), 134.9 (4′-*C*-4-Ph), 136.8 (3-Ar*C*H), 136.9 (4′-*C*-2-Ph), 139.6 (1′-*C*-2-Ph), 149.1 (4-Ar*C*H), 152.6 (2-Ar*C*), 197.1 (4-*C*=O), 200.0 (1-*C*=O); **HRMS** (ESI$^+$) $C_{22}H_{19}ClNO_2$ $[M+H]^+$ found 364.1094, requires 364.1104 (–1.3 ppm).

2,4-bis(4-chlorophenyl)-1-(pyridin-2-yl)butane-1,4-dione 67

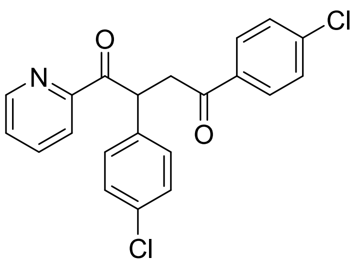

White solid; Yield, 76%; R_f = 0.49 (petroleum ether:ethyl acetate, 5:1); **mp**, 108-109 °C. **IR** ν_{max} (film): 3051, 2905, 1695, 1682, 1339, 1229, 995; **^{1}H NMR** (400 MHz, $CDCl_3$) δ_H: 3.39 (1H, dd, *J* 18.0, 3.8, CH_AH$_B$), 4.15 (1H, dd, *J* 18.1, 10.8, CH$_A$$H_B$), 6.04 (1H, dd, *J* 10.8, 3.8, C*H*), 7.25-7.29 (2.22H, m, Solvent + 3′,5′-*H*-2-Ph), 7.42-7.47 (5H, m, 2′,6′-*H*-2-Ph + 5-Ar*H* + 3′,5′-*H*-4-Ph), 7.81 (1H, td, *J* 7.7, 1.7, 3-Ar*H*), 7.92-7.95 (2H, m, 2′,6′-*H*-4-Ph), 8.05 (1H, dt, *J* 7.9, 1.1, 6-Ar*H*), 8.73 (1H, ddd, *J* 4.7, 1.8, 0.9, 4-Ar*H*). **^{13}C{^{1}H} NMR** (101 MHz, $CDCl_3$) δ_C: 16.2 (*C*H_2), 45.3 (*C*H), 122.9 (6-Ar*C*H), 127.2 (5-Ar*C*H), 128.9 (3′,5′-*C*H-4-Ph), 129.0 (3′,5′-*C*H-2-Ph), 129.6 (2′,6′-*C*H-4-Ph), 130.3 (2′,6′-*C*H-2-Ph), 133.1 (4′-*C*-2-Ph), 134.6 (4′-*C*-4-Ph), 136.5 (1′-*C*-2-Ph), 136.9 (3-Ar*C*H), 139.8 (1′-*C*-4-Ph), 149.0 (4-Ar*C*H), 152.2 (2-Ar*C*), 196.7 (4-*C*=O), 199.6 (1-*C*=O); **HRMS** (ESI$^+$) $C_{22}H_{16}Cl_2NO_2$ $[M+H]^+$ found 384.0544, requires 384.0558 (–2.2 ppm).

4-(3-(4-methoxyphenyl)-4-oxo-4-(pyridin-2-yl)butanoyl)benzonitrile 68

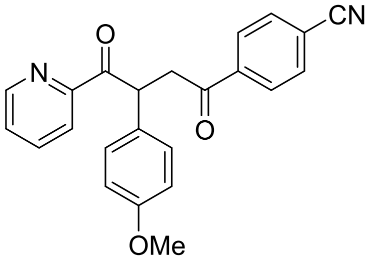

Yellow oil; Yield, 89%; R_f = 0.48 (petroleum ether:ethyl acetate, 2:1); **IR** ν_{max} (film): 3051, 2907, 2837, 2230, 1684, 1508, 1244, 995, 831; **^{1}H NMR** (400 MHz, $CDCl_3$) δ_H: 3.40 (1H, dd, *J* 18.2, 3.7, CH_AH$_B$), 3.76 (3H, s, OCH_3), 4.18 (1H, dd, *J* 18.2, 10.8, CH$_A$$H_B$), 6.02 (1H, dd, *J* 10.8, 3.7, C*H*), 6.82-6.85 (2H, m, 3′,5′-*H*-2-Ph), 7.37-7.44 (3H, m, 5-Ar*H* + 2′,6′-*H*-2-Ph), 7.75-7.81 (3H, m, 3-Ar*H* + 2′,6′-*H*-4-Ph), 8.04 (1H, dt, *J* 7.8, 1.1, 6-Ar*H*), 8.06-8.09 (2H, m, 3′,5′-*H*-4-Ph), 8.73 (1H, ddd, *J* 4.7, 1.7, 0.9, 4-Ar*H*). **^{13}C{^{1}H} NMR** (101 MHz, $CDCl_3$) δ_C: 16.6 (*C*H_2), 45.0 (*C*H), 55.2 (O*C*H_3), 114.3 (3′,5′-*C*H-2-Ph), 17.4 (4′-*C*-4-Ph), 118.0 (*C*N), 122.9 (6-Ar*C*H), 127.1 (5-Ar*C*H), 128.6 (3′,5′-*C*H-4-Ph), 129.5 (1′-*C*-2-Ph), 129.9 (2′,6′-*C*H-2-Ph), 132.5 (2′,6′-*C*H-4-Ph), 136.8 (3-Ar*C*H), 139.4 (1′-*C*-4-Ph), 149.1 (4-Ar*C*H), 152.4 (2-Ar*C*), 158.8 (4′-*C*-2-Ph), 197.1 (4-*C*=O), 199.9 (1-*C*=O); **HRMS** (ESI$^+$) $C_{23}H_{18}N_2O_3$ $[M+Na]^+$ found 393.1205, requires 393.1215 (–

1.1 ppm).

1-(pyridin-2-yl)-2,4-bis(4-(trifluoromethyl)phenyl)butane-1,4-dione 69

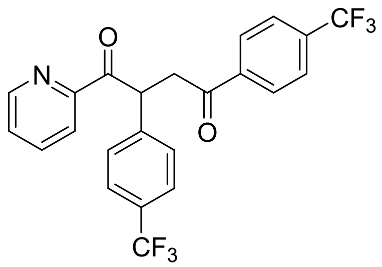

Colorless oil; Yield, 97%; R_f = 0.83 (petroleum ether:ethyl acetate, 2:1); **IR** ν_{max} (film): 3059, 2914, 1687, 1317, 1109, 1065, 835; **^{1}H NMR** (400 MHz, $CDCl_3$) δ_H: 3.47 (1H, dd, *J* 18.2, 3.8, CH_AH$_B$), 4.24 (1H, dd, *J* 18.2, 10.7, CH$_A$$H_B$), 6.17 (1H, dd, *J* 10.7, 3.8, C*H*), 7.47 (1H, ddd, *J* 7.6, 4.8, 1.3, 5-Ar*H*), 7.54-7.58 (2H, m, 2′,6′-*H*-2-Ph), 7.63-7.65 (2H, m, 3′,5′-*H*-2-Ph), 7.73-7.76 (2H, m, 2′,6′-*H*-4-Ph), 7.83 (1H, td, *J* 7.7, 1.7, 3-Ar*H*), 8.06-8.12 (3H, m, 6-Ar*H* + 3′,5′-*H*-4-Ph), 8.74 (1H, ddd, *J* 4.8, 1.8, 0.9, 4-Ar*H*). **^{13}C{^{1}H} NMR** (101 MHz, $CDCl_3$) δ_C: 16.3 (CH_2), 45.8 (*C*H), 122.2 (4′-*C*-2-Ph), 122.2 (4′-*C*-4-Ph), 122.9 (6-Ar*C*H), 124.9 (4′-*C*F$_3$-4-Ph), 125.4 (4′-*C*F$_3$-2-Ph), 125.7 (q, 2′,6′-*C*H-2-Ph + 2′,6′-*C*H-4-Ph), 127.4 (5-Ar*C*H), 128.5 (3′,5′-*C*H-4-Ph), 129.3 (3′,5′-*C*H-2-Ph), 129.7 (q, 4′-*C*F$_3$-4-Ph), 134.8 (q, 4′-*C*F$_3$-2-Ph), 137.0 (3-Ar*C*H), 138.9 (1′-*C*-4-Ph), 142.1 (1′-*C*-2-Ph), 149.1 (4-Ar*C*H), 152.1 (2-Ar*C*), 196.8 (4-*C*=O), 199.3 (1-*C*=O); **^{19}F NMR** (377 MHz, $CDCl_3$) δ_F: -63.1 (s, C*F$_3$*); **HRMS** (ESI$^+$) $C_{23}H_{15}F_6NO_2$ $[M+Na]^+$ found 474.0895, requires 474.0905 (–1.0 ppm).

4,4'-(1,4-dioxo-4-(pyridin-2-yl)butane-1,3-diyl)dibenzonitrile 70

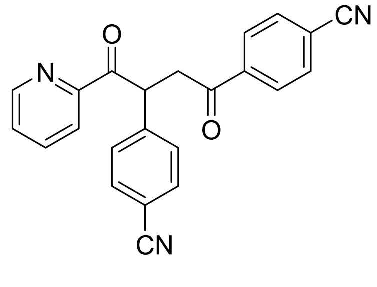

White solid; Yield, 98%; R_f = 0.75 (petroleum ether:ethyl acetate, 1:1); **mp**, 133-134 °C. **IR** ν_{max} (film): 3051, 2918, 2228, 1686, 1674, 1406, 1207, 995; **^{1}H NMR** (400 MHz, $CDCl_3$) δ_H: 3.16 (1H, dd, *J* 18.2, 3.9, CH_AH$_B$), 4.20 (1H, dd, *J* 18.2, 10.6, CH$_A$$H_B$), 6.12 (1H, dd, *J* 10.6, 3.9, C*H*), 7.48 (1H, ddd, *J* 7.6, 4.8, 1.2, 5-Ar*H*), 7.58-7.63 (4H, m, 2′,6′-*H*-2-Ph + 3′,5′-*H*-2-Ph), 7.76-7.79 (2H, m, 3′,5′-*H*-4-Ph), 7.83 (1H, td, *J* 7.7, 1.7, 3-Ar*H*), 8.05-8.09 (3H, m, 6-Ar*H* + 2′,6′-*H*-4-Ph), 8.72 (1H, ddd, *J* 4.8, 1.7, 0.9, 4-Ar*H*). **^{13}C{^{1}H} NMR**

(101 MHz, $CDCl_3$) δ_C: 16.0 (CH_2), 46.2 (*C*H), 111.3 (4-*C*N), 17.8 (4'-*C*N), 117.8 (4′-*C*-4-Ph), 118.6 (4′-*C*-4-Ph), 123.0 (6-Ar*C*H), 127.6 (5-Ar*C*H), 128.6 (2′,6′-*C*H-4-Ph), 129.7 (3′,5′-*C*H-2-Ph), 132.6 (3′,5′-*C*H-4-Ph), 132.6 (2′,6′-*C*H-2-Ph), 137.1 (3-Ar*C*H), 139.1 (1′-*C*-4-Ph), 143.4 (1′-*C*-2-Ph), 149.1 (4-Ar*C*H), 151.8 (2-Ar*C*), 196.2 (4-*C*=O), 198.8 (1-*C*=O); **HRMS** (ESI^+) $C_{23}H_{15}N_3O_2$ $[M+Na]^+$ found 388.1054, requires 388.1062 (–0.7 ppm).

4-phenyl-1,2-di(pyridin-2-yl)butane-1,4-dione 80

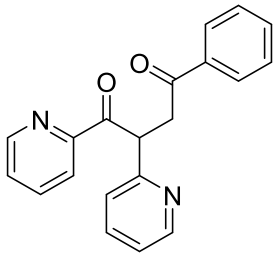

Colorless oil; Yield, 70%; R_f = 0.39 (petroleum ether:ethyl acetate, 5:1); **IR** ν_{max} (film): 3055, 2909, 1699, 1678, 1207, 993; **^{1}H NMR** (400 MHz, $CDCl_3$) δ_H: 3.60 (1H, dd, *J* 18.2, 4.5, CH_AH$_B$), 4.30 (1H, dd, *J* 18.2, 9.6, CH$_A$$H_B$), 6.20 (1H, dd, *J* 11.1, 3.6, C*H*), 7.05-7.11 (1H, m, 5-Ar(2)*H*), 7.37-7.45 (3H, m, 5-Ar(1)*H* +3,5-Ph*H*), 7.51-7.61 (3H, m, 3-Ar(2)*H* + 6-Ar(2)*H* +4-Ph*H*), 7.77 (1H, td, *J* 7.7, 1.1, 3-Ar(1)*H*), 8.01-8.03 (2H, m, 2,6-Ph*H*), 8.08 (1H, dt, *J* 7.8, 1.1, 6-Ar(1)*H*), 8.50 (1H, dt, *J* 4.8, 1.4, 4-Ar(2)*H*), 8.67 (1H, ddd, *J* 4.8, 1.8, 0.9, 4-Ar(1)*H*). **^{13}C{^{1}H} NMR** (101 MHz, $CDCl_3$) δ_C: 41.6 (CH_2), 48.8 (*C*H), 121.8 (5-Ar(2)*C*H), 122.9 (6-Ar(1)*C*H), 124.7 (3-Ar(2)*C*H), 127.0 (5-Ar(1)*C*H), 128.2 (2,6-Ph*C*H), 128.5 (3,5-Ph*C*H), 133.2 (4-Ph*C*H), 136.6 (1-Ph*C*), 136.7 (6-Ar(2)*C*H), 136.8 (3-Ar(1)*C*H), 148.9 (4-Ar(1)*C*H), 149.5 (4-Ar(2)*C*H), 152.7 (2-Ar(1)*C*), 158.4 (2-Ar(1)*C*), 198.2 (4-*C*=O), 198.8 (1-*C*=O); **HRMS** (ESI^+) $C_{20}H_{16}N_2O_2Na$ $[M+H]^+$ found 339.1100, requires 339.1109 (–1.2 ppm).

4-phenyl-1-(pyridin-2-yl)-2-(thiophen-2-yl)butane-1,4-dione 81

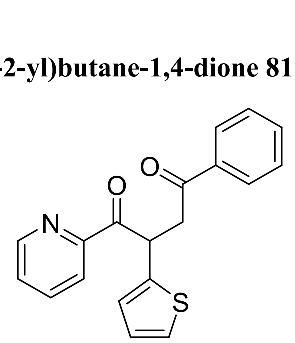

Light yellow oil; Yield, 38%; R_f = 0.15 (petroleum ether:ethyl acetate, 5:1); **IR** ν_{max} (film): 3057, 2907, 1697, 1680, 1219, 995; **^{1}H NMR** (400 MHz, $CDCl_3$) δ_H: 3.62 (1H, dd, *J* 18.1, 3.6, CH_AH$_B$), 4.29 (1H, dd, *J* 18.1, 11.1, CH$_A$$H_B$), 6.41 (1H, dd, *J* 11.1, 3.6, C*H*), 6.93 (1H, dd, *J* 5.1, 3.5, SC*H*), 7.07 (1H, dd, *J* 3.6, 0.9,

SCHC*H*), 7.19 (1H, dd, *J* 5.1, 1.2, SCHCHC*H*), 7.16-7.50 (3H, m, 5-Ar*H* +3,5-Ph*H*), 7.56-7.61 (1H, m, 4-Ph*H*), 7.82 (1H, td, *J* 7.7, 1.1, 3-Ar*H*), 8.00-8.03 (2H, m, 2,6-Ph*H*), 8.10 (1H, dt, *J* 7.8, 1.1, 6-Ar*H*), 8.80 (1H, ddd, *J* 4.8, 1.8, 0.9, 4-Ar*H*). **$^{13}C\{^1H\}$ NMR** (101 MHz, $CDCl_3$) δ_C: 40.4 (*C*H), 16.9 (*C*H_2), 123.0 (6-Ar*C*H), 125.1 (S*C*H), 126.3 (SCH*C*H), 127.0 (SCHCH*C*H), 127.2 (5-Ar*C*H), 128.2 (2,6-Ph*C*H), 128.6 (3,5-Ph*C*H), 133.4 (4-Ph*C*H), 136.2 (1-Ph*C*), 136.9 (3-Ar*C*H), 140.4 (S*C*CH), 149.1 (4-Ar*C*H), 152.2 (2-Ar*C*), 197.7 (4-*C*=O), 197.7 (1-*C*=O); **HRMS** (ESI$^+$) $C_{19}H_{15}NO_2SNa$ $[M+H]^+$ found 344.0711, requires 344.0721 (–1.4 ppm).

2-(furan-2-yl)-4-phenyl-1-(pyridin-2-yl)butane-1,4-dione 82

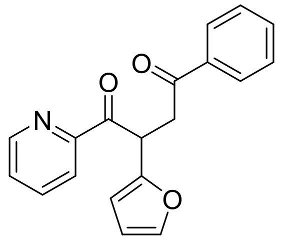

Light yellow oil; Yield, 40%; R_f = 0.25 (petroleum ether:ethyl acetate, 5:1); **IR** ν_{max} (film): 3055, 2914, 1701, 1680, 1209, 995; **^1H NMR** (400 MHz, $CDCl_3$) δ_H: 3.59 (1H, dd, *J* 18.2, 3.8, C*H_A*H_B), 4.24 (1H, dd, *J* 18.2, 10.8, CH_A*H_B*), 6.20-6.24 (2H, m, C*H* + OC*H*), 6.29 (1H, dd, *J* 3.3, 1.9, OCHC*H*), 7.36 (1H, dd, *J* 1.9, 0.8, OCHCHC*H*), 7.46-7.51 (3H, m, 5-Ar*H* +3,5-Ph*H*), 7.57-7.61 (1H, m, 4-Ph*H*), 7.85 (1H, td, *J* 7.7, 1.7, 3-Ar*H*), 8.01-8.04 (2H, m, 2,6-Ph*H*), 8.11 (1H, dt, *J* 7.8, 1.1, 6-Ar*H*), 8.79 (1H, ddd, *J* 4.8, 1.7, 0.9, 4-Ar*H*). **$^{13}C\{^1H\}$ NMR** (101 MHz, $CDCl_3$) δ_C: 39.7 (*C*H), 40.5 (*C*H_2), 107.8 (O*C*H), 110.6 (OCH*C*H), 122.9 (6-Ar*C*H), 127.2 (5-Ar*C*H), 128.2 (2,6-Ph*C*H), 128.6 (3,5-Ph*C*H), 133.3 (4-Ph*C*H), 136.3 (1-Ph*C*), 136.9 (3-Ar*C*H), 142.3 (OCHCH*C*H), 149.1 (4-Ar*C*H), 151.2 (O*C*CH), 152.4 (2-Ar*C*), 197.6 (1-*C*=O), 197.7 (4-*C*=O); **HRMS** (ESI$^+$) $C_{19}H_{16}NO_3$ $[M+H]^+$ found 306.1122, requires 306.1130 (–0.9 ppm).

4-phenyl-1-(pyridin-2-yl)-2-(trifluoromethyl)butane-1,4-dione 83

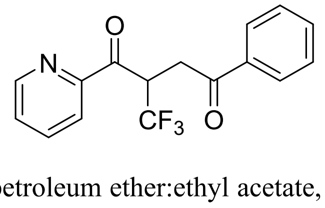

Colorless oil; Yield, 48%; R_f = 0.46 (petroleum ether:ethyl acetate, 5:1); **IR** ν_{max} (film): 3059, 2924, 1707, 1682, 1113, 995; **^1H NMR** (400 MHz, $CDCl_3$) δ_H: 3.64 (1H, dd, *J* 18.2, 2.9, C*H_A*H_B), 4.12 (1H, dd, *J* 18.1, 11.6, CH_A*H_B*), 5.89 (1H, dqd, *J* 11.8, 8.9, 2.9, C*H*), 7.47-7.51 (2H, m, 2,6-Ph*H*), 7.56-7.64 (2H, m, 5-Ar*H* + 4-Ph*H*), 7.91 (1H, td, *J* 7.7, 1.7, 3-Ar*H*), 7.96-7.99 (2H, m, 3,5-Ph*H*), 8.15 (1H, dt, *J* 7.9, 1.1, 6-Ar*H*), 8.84

(1H, ddd, *J* 4.7, 1.8, 0.9, 4-Ar*H*). **^{13}C{^{1}H} NMR** (101 MHz, $CDCl_3$) δ_C: 37.0 (CH_2), 45.2 (q, *C*H), 122.6 (6-Ar*C*H), 126.5 (q, CF_3), 127.7 (5-Ar*C*H), 128.3 (2,6-Ph*C*H), 128.8 (3,5-Ph*C*H), 133.8 (4-Ph*C*H), 135.5 (1-Ph*C*), 137.1 (3-Ar*C*H), 149.3 (4-Ar*C*H), 152.2 (2-Ar*C*), 194.7 (1-*C*=O), 196.0 (4-*C*=O); **^{19}F NMR** (377 MHz, $CDCl_3$) δ_F: -65.8 (s, CF_3); **HRMS** (ESI$^+$) $C_{16}H_{13}F_3NO_2$ [M+H]$^+$ found 308.0882, requires 308.0898 (–3.6 ppm).

2-methyl-4-phenyl-1-(pyridin-2-yl)butane-1,4-dione 84

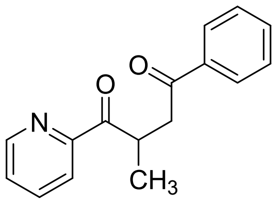

Colorless oil; Yield, 27%; R_f = 0.41 (petroleum ether:ethyl acetate, 5:1); **IR** ν_{max} (film): 3055, 2970, 1682, 1582, 1217, 982; **^{1}H NMR** (400 MHz, $CDCl_3$) δ_H: 1.33 (1H, d, *J* 7.2, CH_3), 3.18 (1H, dd, *J* 17.9, 4.7, CH_AH_B), 3.77 (1H, dd, *J* 17.8, 9.3, CH_AH_B), 4.71 (1H, dqd, *J* 9.3, 7.2, 4.7, C*H*), 7.45-7.52 (3H, m, 5-Ar*H* +2,6-Ph*H*), 7.54-7.59 (1H, m, 4-Ph*H*), 7.87 (1H, td, *J* 7.7, 1.8, 3-Ar*H*), 8.00-8.03 (2H, m, 3,5-Ph*H*), 8.10 (1H, dt, *J* 7.9, 1.1, 6-Ar*H*), 8.75 (1H, ddd, *J* 4.8, 1.8, 0.9, 4-Ar*H*). **^{13}C{^{1}H} NMR** (101 MHz, $CDCl_3$) δ_C: 17.4 (CH_3), 35.2 (*C*H), 42.7 (CH_2), 122.5 (6-Ar*C*H), 127.0 (5-Ar*C*H), 128.2 (2,6-Ph*C*H), 128.6 (3,5-Ph*C*H), 133.1 (4-Ph*C*H), 136.6 (1-Ph*C*), 136.9 (3-Ar*C*H), 149.1 (4-Ar*C*H), 152.7 (2-Ar*C*), 198.5 (4-*C*=O), 204.3 (1-*C*=O); **HRMS** (ESI$^+$) $C_{16}H_{16}NO_2$ [M+H]$^+$ found 354.1168, requires 354.1181 (–3.1 ppm).

4-(4-fluorophenyl)-2-(furan-2-yl)-1-(pyridin-2-yl)butane-1,4-dione 89

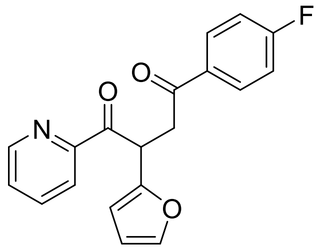

Light yellow oil; Yield, 51%; R_f = 0.21 (petroleum ether:ethyl acetate, 5:1); **IR** ν_{max} (film): 3055, 2914, 1701, 1680, 1207, 995; **^{1}H NMR** (400 MHz, $CDCl_3$) δ_H: 3.54 (1H, dd, *J* 18.1, 3.8, CH_AH_B), 4.20 (1H, dd, *J* 18.1, 10.8, CH_AH_B), 6.19-6.24 (2H, m, C*H* + OC*H*), 6.29 (1H, dd, *J* 3.3, 1.9, OCHC*H*), 7.12-7.17 (2H, m, 3,5-Ph*H*), 7.35 (1H, dd, *J* 1.9, 0.8, OCHCHC*H*), 7.48 (1H, ddd, *J* 7.6, 4.8, 1.3, 5-Ar*H*), 7.85 (1H, td, *J* 7.7, 1.8, 3-Ar*H*), 8.02-8.07 (2H, m, 2,6-Ph*H*), 8.09 (1H, dt, *J* 7.8, 1.1, 6-Ar*H*), 8.78 (1H, ddd, *J* 4.7, 1.7, 0.9, 4-Ar*H*). **^{13}C{^{1}H} NMR** (101 MHz, $CDCl_3$) δ_C: 39.7 (*C*H), 40.3 (CH_2), 107.9 (O*C*H), 110.6 (OCH*C*H), 115.6

(3,5-Ph*C*H), 115.8 (3,5-Ph*C*H), 122.9 (6-Ar*C*H), 127.2 (5-Ar*C*H), 130.8 (2,6-Ph*C*H), 130.9 (2,6-Ph*C*H), 132.7 (4-Ar*C*), 132.8 (4-Ph*C*), 136.9 (3-Ar*C*H), 142.3 (OCHCH*C*H), 149.1 (5-Ar*C*H), 151.0 (O*C*CH), 152.4 (2-Ar*C*), 164.6 (1-Ph*C*), 167.2 (1-Ph*C*), 196.1 (4-*C*=O), 197.5 (1-*C*=O); **^{19}F NMR** (377 MHz, $CDCl_3$) δ_F: -107.8 (s, C*F*); **HRMS** (ESI$^+$) $C_{19}H_{14}NO_3FNa$ [M+Na]$^+$ found 346.0850, requires 346.0855 (–4.0 ppm).

4-(4-chlorophenyl)-2-(furan-2-yl)-1-(pyridin-2-yl)butane-1,4-dione 90

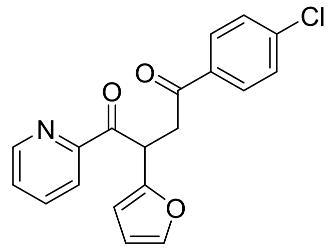

Light yellow oil; Yield, 59%; R_f = 0.21 (petroleum ether:ethyl acetate, 5:1); **IR** ν_{max} (film): 3057, 2914, 1699, 1682, 1209, 993; **^{1}H NMR** (400 MHz, $CDCl_3$) δ_H: 3.55 (1H, dd, *J* 18.2, 3.8, C*H$_A$*H$_B$), 4.20 (1H, dd, *J* 18.1, 10.8, CH$_A$*H$_B$*), 6.19-6.23 (2H, m, C*H* + OC*H*), 6.28 (1H, dd, *J* 3.3, 1.9, OCHC*H*), 7.35 (1H, dd, *J* 1.9, 0.8, OCHCHC*H*), 7.16-7.50 (3H, m, 5-Ar*H* +3,5-Ph*H*), 7.85 (1H, td, *J* 7.7, 1.8, 3-Ar*H*), 7.94-7.98 (2H, m, 2,6-Ph*H*), 8.09 (1H, dt, *J* 7.9, 1.1, 6-Ar*H*), 8.78 (1H, ddd, *J* 4.7, 1.7, 0.9, 4-Ar*H*). **^{13}C{^{1}H} NMR** (101 MHz, $CDCl_3$) δ_C: 39.7 (*C*H), 40.4 (*C*H$_2$), 107.9 (O*C*H), 110.6 (OCH*C*H), 122.9 (6-Ar*C*H), 127.2 (5-Ar*C*H), 129.0 (3,5-Ph*C*H), 129.6 (2,6-Ph*C*H), 134.6 (4-Ph*C*), 136.9 (3-Ar*C*H), 139.8 (1-Ph*C*), 142.3 (OCHCH*C*H), 149.1 (4-Ar*C*H), 150.9 (O*C*CH), 152.3 (2-Ar*C*), 196.5 (4-*C*=O), 197.4 (1-*C*=O); **HRMS** (ESI$^+$) $C_{19}H_{14}NO_3ClNa$ [M+Na]$^+$ found 362.0554, requires 362.0560 (–3.2 ppm).

4-(4-bromophenyl)-2-(furan-2-yl)-1-(pyridin-2-yl)butane-1,4-dione 91

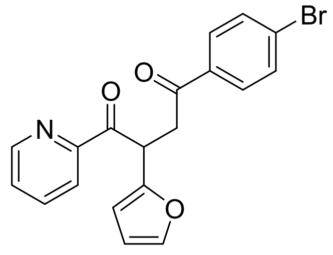

Light yellow oil; Yield, 61%; R_f = 0.22 (petroleum ether:ethyl acetate, 5:1); **IR** ν_{max} (film): 3055, 2913, 1699, 1682, 1207, 993; **^{1}H NMR** (400 MHz, $CDCl_3$) δ_H: 3.53 (1H, dd, *J* 18.2, 3.8, C*H$_A$*H$_B$), 4.19 (1H, dd, *J* 18.1, 10.8, CH$_A$*H$_B$*), 6.19-6.23 (2H, m, C*H* + OC*H*), 6.29 (1H, dd, *J* 3.3, 1.8, OCHC*H*), 7.35 (1H, dd, *J* 1.9, 0.8, OCHCHC*H*), 7.49 (1H, ddd, *J* 7.6, 4.7, 1.3, 5-Ar*H*), 7.59-7.64 (2H, m, 2,6-Ph*H*), 7.82-7.90 (3H, m, 3-

Ar*H* +3,5-Ph*H*), 8.09 (1H, dt, *J* 7.9, 1.1, 6-Ar*H*), 8.78 (1H, ddd, *J* 4.8, 1.7, 0.9, 4-Ar*H*). **^{13}C{^{1}H} NMR** (101 MHz, $CDCl_3$) δ_C: 39.7 (*C*H), 40.3 (*C*H_2), 107.9 (O*C*H), 110.6 (OCH*C*H), 122.9 (6-Ar*C*H), 127.2 (5-Ar*C*H), 128.6 (4-Ph*C*), 129.7 (3,5-Ph*C*H), 131.9 (2,6-Ph*C*H), 135.0 (1-Ph*C*), 136.9 (3-Ar*C*H), 142.3 (OCHCH*C*H), 149.1 (4-Ar*C*H), 150.9 (O*C*CH), 152.3 (2-Ar*C*), 196.7 (4-*C*=O), 197.4 (1-*C*=O); **HRMS** (ESI$^+$) $C_{19}H_{15}NO_3Br$ $[M+H]^+$ found 384.0230, requires 384.0235 (–2.6 ppm).

2-(furan-2-yl)-1-(pyridin-2-yl)-4-(p-tolyl)butane-1,4-dione 92

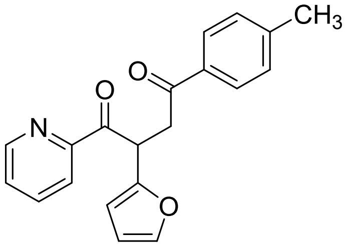

Light yellow oil; Yield, 35%; R_f = 0.18 (petroleum ether:ethyl acetate, 5:1); **IR** ν_{max} (film): 3053, 2916, 1701, 1674, 1206, 995; **^{1}H NMR** (400 MHz, $CDCl_3$) δ_H: 2.16 (3H, s, C*H₃*), 3.58 (1H, dd, *J* 18.1, 3.8, C*H*$_A$H$_B$), 4.21 (1H, dd, *J* 18.1, 10.8, CH$_A$*H*$_B$), 6.19-6.24 (2H, m, C*H* + OC*H*), 6.29 (1H, dd, *J* 3.3, 1.9, OCHC*H*), 7.26-7.28 (2.38H, m, Solvent +3,5-Ph*H*), 7.35 (1H, dd, *J* 1.9, 0.8, OCHCHC*H*), 7.48 (1H, ddd, *J* 7.5, 4.8, 1.3, 5-Ar*H*), 7.84 (1H, td, *J* 7.7, 1.8, 3-Ar*H*), 7.90-7.93 (2H, m, 2,6-Ph*H*), 8.10 (1H, dt, *J* 7.9, 1.1, 6-Ar*H*), 8.78 (1H, ddd, *J* 4.8, 1.8, 0.9, 4-Ar*H*). **^{13}C{^{1}H} NMR** (101 MHz, $CDCl_3$) δ_C: 21.7 (*C*H_3), 39.7 (*C*H), 40.5 (*C*H_2), 107.8 (O*C*H), 110.6 (OCH*C*H), 122.9 (6-Ar*C*H), 127.1 (5-Ar*C*H), 128.4 (2,6-Ph*C*H), 129.3 (3,5-Ph*C*H), 133.8 (1-Ph*C*), 136.9 (3-Ar*C*H), 142.3 (OCHCH*C*H), 144.1 (4-Ph*C*), 149.1 (4-Ar*C*H), 151.2 (O*C*CH), 152.5 (2-Ar*C*), 197.3 (1-*C*=O), 197.7 (4-*C*=O); **HRMS** (ESI$^+$) $C_{20}H_{17}NO_3Na$ $[M+Na]^+$ found 342.1101, requires 342.1106 (–3.7 ppm).

dimethyl 2-(1-oxo-1-(pyridin-2-yl)propan-2-yl)malonate 100

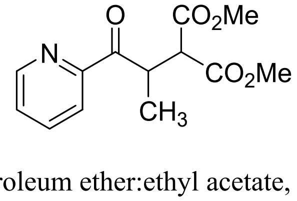

White solid; Yield, 66%; R_f = 0.25 (petroleum ether:ethyl acetate, 5:1); **mp**, 60-61 °C. **IR** ν_{max} (film): 2957, 1757, 1736, 1690, 1435, 1277, 1053, 982; **^{1}H NMR** (400 MHz, $CDCl_3$) δ_H: 1.25 (3H, d, *J* 7.2, C*H₃*), 3.67 (3H, s, CO_2C*H₃*), 3.81 (3H, s, CO_2C*H₃*), 4.06 (1H, d, *J* 10.5, CH_3CHC*H*), 4.81 (1H, dq, *J* 10.9, 7.2, CH_3C*H*CH), 7.50 (1H, ddd, *J* 7.6, 4.7, 1.2, 5-Ar*H*), 7.87 (1H, td, *J* 7.7, 1.7, 3-Ar*H*), 8.07 (1H, dt, *J* 7.8, 1.1,

6-Ar*H*), 8.74 (1H, ddd, *J* 4.7, 1.7, 0.9, 4-Ar*H*). **^{13}C{^{1}H} NMR** (101 MHz, $CDCl_3$) δ_C: 15.5 (*C*H_3), 39.4 (CH_3*C*H), 52.6 (CO*C*H_3), 52.7 (CO*C*H_3), 54.5 (CH_3CH*C*H), 122.7 (5-Ar*C*H), 127.3 (3-Ar*C*H), 137.0 (6-Ar*C*H), 149.1 (4-Ar*C*H), 152.0 (2-Ar*C*), 169.0 (*C*O_2CH_3), 169.1 (*C*O_2CH_3), 202.2 (*C*=O); **HRMS** (ESI$^+$) $C_{13}H_{16}NO_5$ $[M+H]^+$ found 266.1014, requires 266.1028 (–3.3 ppm).

3-(phenylsulfonyl)-1-(pyridin-2-yl)propan-1-one 101

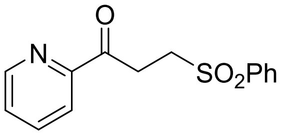

White solid; Yield, 53%; R_f = 0.32 (petroleum ether:ethyl acetate, 1:1); **mp**, 83-84 °C. **IR** ν_{max} (film): 3063, 2947, 1703,1302, 1142, 974; **^{1}H NMR** (400 MHz, $CDCl_3$) δ_H: 3.55-3.64 (2H, m, $COCH_2C$*H_2*), 3.71-3.77 (2H, m, COC*H_2*CH_2), 7.52 (1H, ddd, *J* 7.6, 4.7, 1.3, 3-Ar*H*), 7.57-7.73 (3H, m, 3,4,5-Ph*H*), 7.85 (1H, dd, *J* 7.7, 1.7, 6-Ar*H*), 7.95-8.09 (3H, m, 5-Ar*H* + 2,6-Ph*H*), 8.70 (1H, ddd, *J* 4.8, 1.8, 0.9, 4-Ar*H*). **^{13}C{^{1}H} NMR** (101 MHz, $CDCl_3$) δ_C: 31.3 (CO*C*H_2CH_2), 51.1 ($COCH_2$*C*H_2), 121.9 (5-Ar*C*H), 127.7 (3-Ar*C*H), 128.2 (2,6-Ph*C*H), 129.4 (3,5-Ph*C*H), 133.8 (4-Ph*C*H), 137.0 (6-Ar*C*H), 139.0 (1-Ph*C*), 149.2 (4-Ar*C*H), 152.3 (2-Ar*C*), 197.5 (*C*=O); **HRMS** (ESI$^+$) $C_{14}H_{14}NO_3S$ $[M+H]^+$ found 276.0681, requires 276.0694 (–2.9 ppm).

2-cyclohexyl-3-nitro-1-(pyridin-2-yl)propan-1-one 102

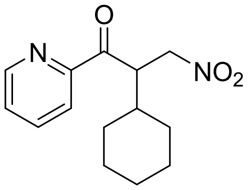

White solid, Yield, 25%; R_f = 0.42 (petroleum ether:ethyl acetate, 5:1); **mp**,107-110 °C (literature: 111-113 °C[43]); **^{1}H NMR** (400 MHz, CD_2Cl_2) δ_H: 0.87-1.02 (1H, m, Cy*H*), 1.08-1.35 (4H, m, Cy*H*), 1.56-1.80 (5.65H, m, H_2O + Cy*H*), 1.82-1.93 (1H, m, Cy*H*), 4.63 (1H, dd, *J* 14.5, 11.0, CH_AH_B), 4.90 (1H, ddd, *J* 11.0, 5.2, 3.3, C*H*), 5.10 (1H, dd, *J* 14.5, 11.0, CH_AH_B), 7.56 (1H, ddd, *J* 7.5, 4.7, 1.3, 3-Ar*H*), 7.93 (1H, td, *J* 7.7, 1.7, 3-Ar*H*), 8.09 (1H, dt, *J* 7.9, 1.1, 6-Ar*H*), 8.76 (1H, ddd, *J* 4.7, 1.7, 0.9, 4-Ar*H*). Data in accordance with literature.[43]

methyl 2,5-dioxo-4-phenyl-5-(pyridin-2-yl)pentanoate 103

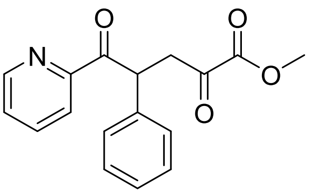

White solid; Yield, 98%; R_f = 0.18 (petroleum ether:ethyl acetate, 5:1); **mp**, 96-97 °C. **IR** ν_{max} (film): 3061, 2916, 1722, 1688, 1281, 1055, 957; **^{1}H NMR** (400 MHz, $CDCl_3$) δ_H: 3.31 (1H, dd, *J* 19.3, 4.2, CH_AH$_B$), 3.89 (3H, s, CH_3), 4.00 (1H, dd, *J* 19.3, 10.5, CH$_A$$H_B$), 5.89 (1H, dd, *J* 10.5, 4.2, C*H*), 7.18-7.22 (1H, m, 4-Ph*H*), 7.26-7.30 (2.66H, m, Solvent + 3,5-Ph*H*), 7.40-7.16 (3H, m, 5-Ar*H* + 2,6-Ph*H*), 7.78 (1H, td, *J* 7.7, 1.7, 3-Ar*H*), 8.01 (1H, dt, *J* 7.9, 1.1, 6-Ar*H*), 8.68 (1H, ddd, *J* 4.8, 1.7, 0.9, 4-Ar*H*). **^{13}C{^{1}H} NMR** (101 MHz, $CDCl_3$) δ_C: 16.4 (CH_2), 45.9 (*C*H), 53.0 (CH_3), 122.9 (6-Ar*C*H), 127.1 (5-Ar*C*H), 127.3 (4-Ph*C*H), 128.8 (2,6-Ph*C*H), 128.8 (3,5-Ph*C*H), 136.7 (3-Ar*C*H), 137.4 (1-Ph*C*), 149.0 (4-Ar*C*H), 152.2 (2-Ar*C*), 160.9 (*C*OOMe), 192.1 (2-*C*=O), 199.2 (5-*C*=O); **HRMS** (ESI$^+$) $C_{17}H_{16}NO_4$ $[M+H]^+$ found 298.1063, requires 298.1079 (–3.7 ppm).

methyl 4-oxo-4-phenyl-2-picolinoylbutanoate 104

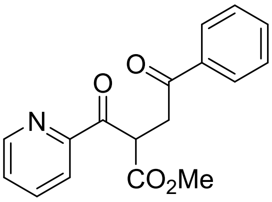

Colorless oil; Yield, 97%; R_f = 0.80 (petroleum ether:ethyl acetate, 5:1); **IR** ν_{max} (film): 3057, 2953, 1734, 1682, 1219, 995; **^{1}H NMR** (400 MHz, $CDCl_3$) δ_H: 3.68-3.74 (4H, m, CH_AH$_B$ + CH_3), 3.91 (1H, dd, *J* 18.0, 8.9, CH$_A$$H_B$), 5.65 (1H, dd, *J* 8.9, 5.2, C*H*), 7.44-7.53 (3H, m, 5-Ar*H* + 3,5-Ph*H*), 7.56-7.61 (1H, m, 4-Ph*H*), 7.87 (1H, td, *J* 7.7, 1.7, 3-Ar*H*), 7.99-8.02 (2H, m, 2,6-Ph*H*), 8.12 (1H, dt, *J* 7.9, 1.1, 6-Ar*H*), 8.74 (1H, ddd, *J* 4.8, 1.7, 0.9, 4-Ar*H*). **^{13}C{^{1}H} NMR** (101 MHz, $CDCl_3$) δ_C: 38.1 (CH_2), 47.3 (*C*H), 52.6 (CH_3), 122.6 (6-Ar*C*H), 127.5 (5-Ar*C*H), 128.2 (2,6-Ph*C*H), 128.7 (3,5-Ph*C*H), 133.4 (4-Ph*C*H), 136.1 (1-Ph*C*), 137.0 (3-Ar*C*H), 149.1 (4-Ar*C*H), 152.2 (2-Ar*C*), 170.8 (*C*OOMe), 196.2 (Ar*C*=O), 196.7 (Ph*C*=O); **HRMS** (ESI$^+$) $C_{17}H_{16}NO_4$ $[M+H]^+$ found 298.1063, requires 298.1072 (–0.7 ppm).

5,5,5-trichloro-2-phenyl-1-(pyridin-2-yl)pentane-1,4-dione 106

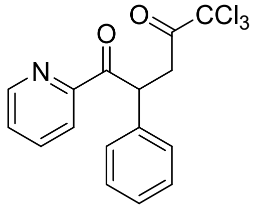

White solid; Yield, 90%; R_f = 0.40 (petroleum ether:ethyl acetate, 5:1); **mp**, 65-66 °C. **IR** ν_{max} (film): 3051, 2920, 1751, 1694, 1225, 984; **^{1}H NMR** (400 MHz, $CDCl_3$) δ_H: 3.55 (1H, dd, *J* 18.8, 4.4, CH_AH$_B$), 4.17 (1H, dd, *J* 18.7, 10.2, CH$_A$$H_B$), 5.98 (1H, dd, *J* 10.2, 4.4, C*H*), 7.20-7.25 (1H, m, 4-Ph*H*), 7.29-7.33 (2.21H, m, Solvent + 3,5-Ph*H*), 7.41-7.47 (3H, m, 5-Ar*H* + 2,6-Ph*H*), 7.79 (1H, td, *J* 7.7, 1.7, 3-Ar*H*), 8.05 (1H, dt, *J* 7.8, 1.1, 6-Ar*H*), 8.70 (1H, ddd, *J* 4.7, 1.7, 0.9, 4-Ar*H*). **^{13}C{^{1}H} NMR** (101 MHz, $CDCl_3$) δ_C: 38.0 (CH_2), 46.5 (*C*H), 95.7 (*C*Cl_3), 123.0 (6-Ar*C*H), 127.3 (5-Ar*C*H), 127.5 (4-Ph*C*H), 128.9 (2,6-Ph*C*H + 3,5-Ph*C*H), 136.8 (3-Ar*C*H), 136.9 (1-Ph*C*), 149.0 (4-Ar*C*H), 152.1 (2-Ar*C*), 189.9 (CCl_3*C*=O), 199.0 (Ar*C*=O); **HRMS** (ESI^+) $C_{16}H_{13}Cl_3NO_2$ $[M+H]^+$ found 356.0006, requires 356.0012 (–0.7 ppm).

5. Computational Details

Geometry optimisations were performed with the meta-hybrid M06-2X functional[44] using the double-ζ, def2-SVP basis set from the redefinition of the Ahlrichs family of basis sets.[45-48] Implicit solvation was considered through the use of the SMD model employing the parameters of DCM (ε = 7.4257).[49] An ultrafine integration grid (99 radial shells with 590 angular points per shell) was used for all calculations and all species were formally treated as closed-shell systems with restricted Kohn-Sham DFT used throughout. The nature of minima and transition states located were verified by the computation of harmonic frequencies at the same level of theory. Single-point energies (E_{sp}) were also evaluated using the M06-2X functional[44] with a larger, triple-ζ, def2-TZVP basis. Implicit solvation was also included at this level of theory using the same ultrafine integration grid (99,590). Thermochemistry was evaluated at 1 atm and 298.15 K using thermodynamic calculations at the level of geometry optimisation (thermal corrections to enthalpy, $\delta H_{298.15}$, and entropies $S_{298.15}$) in combination with energetics obtained from single-point calculations. Gibbs free energy was calculated at 298.15 K using **Equation S13** and additional Martin Hay Pratt empirical entropic corrections included (S_{MHP} = 2.18 kcal/mol per particle, evaluated at 254 atm to mimic bulk DCM).[50] Hirshfeld charges[51] were obtained on the chalcone-BI adduct intermediate structure (**vii**) by population analysis (charge = atomic number − population) and are reported relative to the charge on the unsubstituted chalcone for clarity across the series. All computations were performed using the Gaussian16, C.01

programme[52] with visualisation of structures using CYLview.[53]

$$G_{298.15} = E_{sp} + \delta H_{298.15} - TS_{298.15} + \mathrm{S_{MHP}} \quad \textbf{Equation S13}$$

6. Conformational Approach

Conformational flexibility was considered by using semi-empirical GFN2-xTB[54] and CREST meta-dynamics[55] with an energy window 'ewin' of 10kcal/mol. The number of conformations were reduced using dynamic RMSD cutoffs to N_{max}=40 before performing crude preoptimisations at the $\mathrm{TPSS_{SMD(DCM)}}$/def2SV[56] level of theory with 'loose' optimisation criteria and a coarser 'fine' integration grid. This was performed to avoid over-dependence on the GFN2-xTB energies from CREST and to ensure sampling over the broader conformational space at a reduced expense compared to 'production' DFT. The conformational ensemble was then reduced to N_{max}=30 with an RMSD filter to remove conformations optimised to the same minimum (within 0.25 Å) and an energy ranking (of electronic energies) to remove the higher energy structures. Preoptimisations at the $\mathrm{M062X_{SMD(DCM)}}$/def2SVP level of theory were then performed, with 'loose' optimisation criteria and an 'ultrafine' integration grid. Again, the conformational ensemble was reduced to N_{max}=20, with an RMSD filter to remove structures optimised to the same minimum (within 0.25 Å) and energy ranking (of electronic energies) to remove the higher energy structures. Full DFT calculations were then performed at the level of theory described above, including frequency calculations on the optimised structures. The structure with the lowest Gibbs free energy was used as representative of the global minimum after this treatment. Transition states were approached in a similar manner, with a constraint placed upon the TS atoms during the meta-dynamics (force constant = 5 a.u.) and preoptimisations were performed with the TS bonds frozen before full relaxation for the final full DFT TS calculation.

7. Computed Reactivity

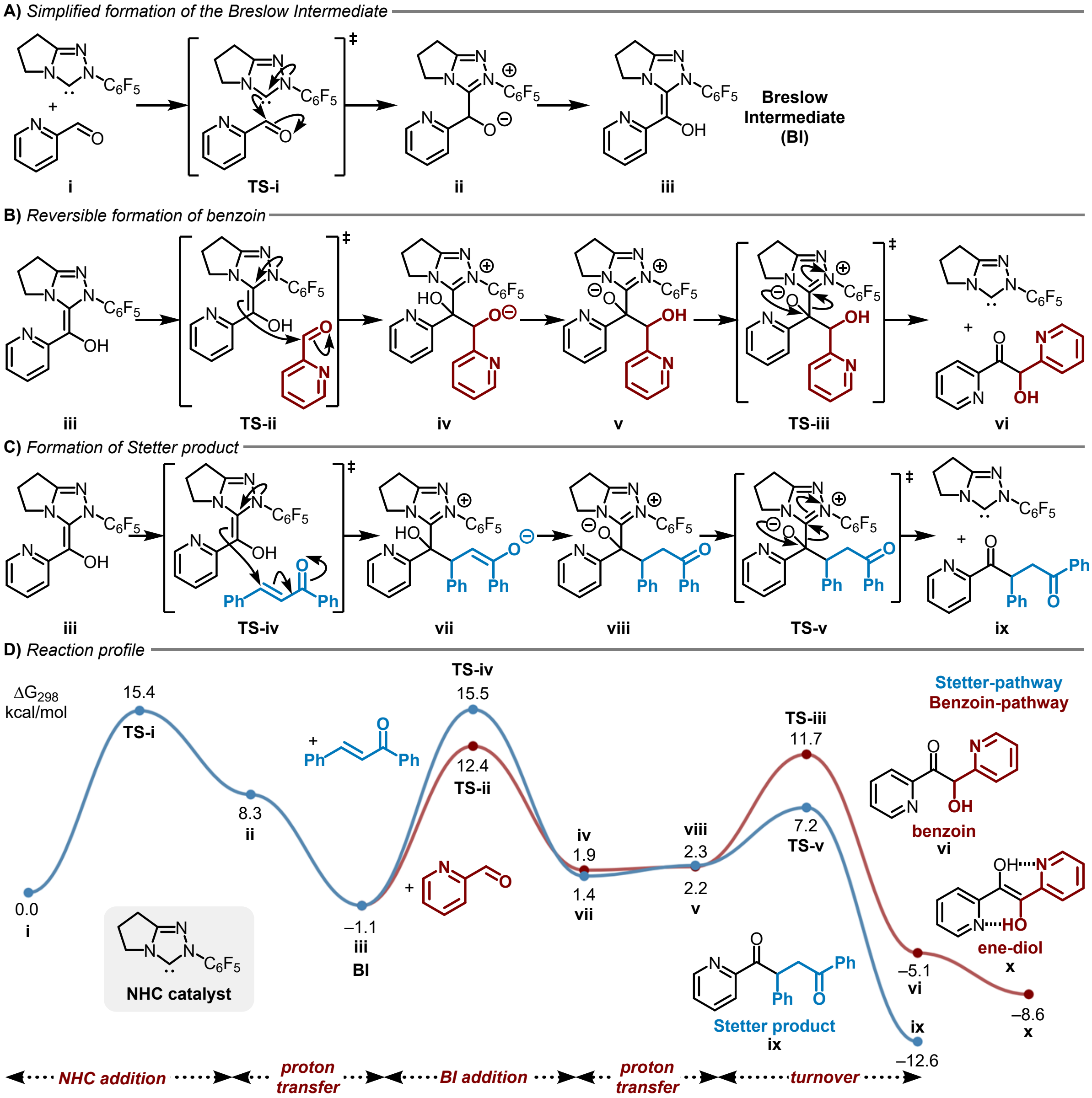

Figure S86. Simplified reactivity for the formation of Breslow intermediate and reactivity in intermolecular Stetter reactions. Gibbs free energies (ΔG_{298}) are shown in kcal/mol.

The conversion of benzoin (**vi**) to the ene-diol (**x**) involves an intramolecular proton transfer. Based on the reaction monitoring performed, this proton transfer is likely to proceed with a barrier slightly lower than the conversion of benzoin (**vi**) to the Stetter product (**ix**) via **TS-iv**, as the formation of the ene-diol (**x**) was observed to be faster than the formation of the Stetter product (**ix**). Computation of this pathway (and of other proton transfers) was not modelled due to challenges in accurately describing such transformations which may be mediated by protic sources in the reaction mixture.

8. Chalcone Reactivity Trends

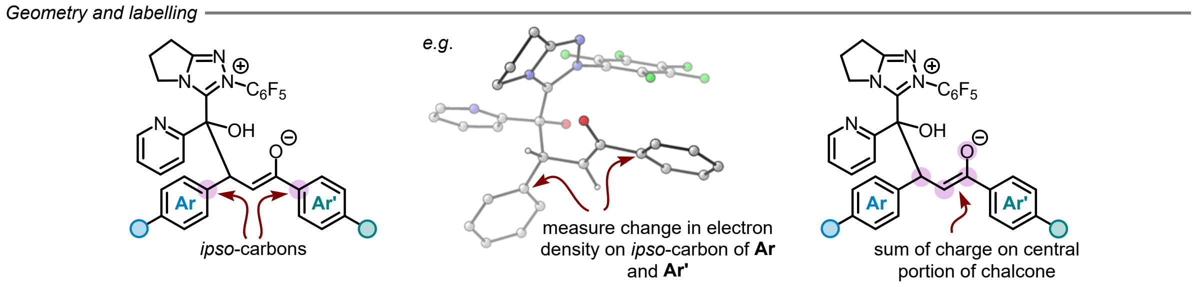

Figure S87. Labelling and structure of optimised intermediate **vii**.

Hammett parameters were found to correlate well to Hirshfeld charges on the *ipso*-carbons as described by Luchini and Paton[57] (**Figure S88A**). The charges at each position were shown to be independent of substitution on each Ar group (**Figure S88B**). Note the different effect of substitution with the change in $\log(k_X/k_H)$. Electron-withdrawing groups were found to react fastest, consistent with a stabilisation of a negative charge in the chalcone component of intermediate **vii**. Summation of the charges on the central chalcone component of intermediate **vii** allows for a single feature to describe the electronic effects of substitution. This feature can be used to identify a relative accumulation of charge from the computed electron densities with electron-donating substituents (more −ve charge) and *vice-versa*. This feature also correlates well to the empirically observed Hammett value for disubstituted chalcones ($\sigma + 2.4\sigma'$) (**Figure S88C**).

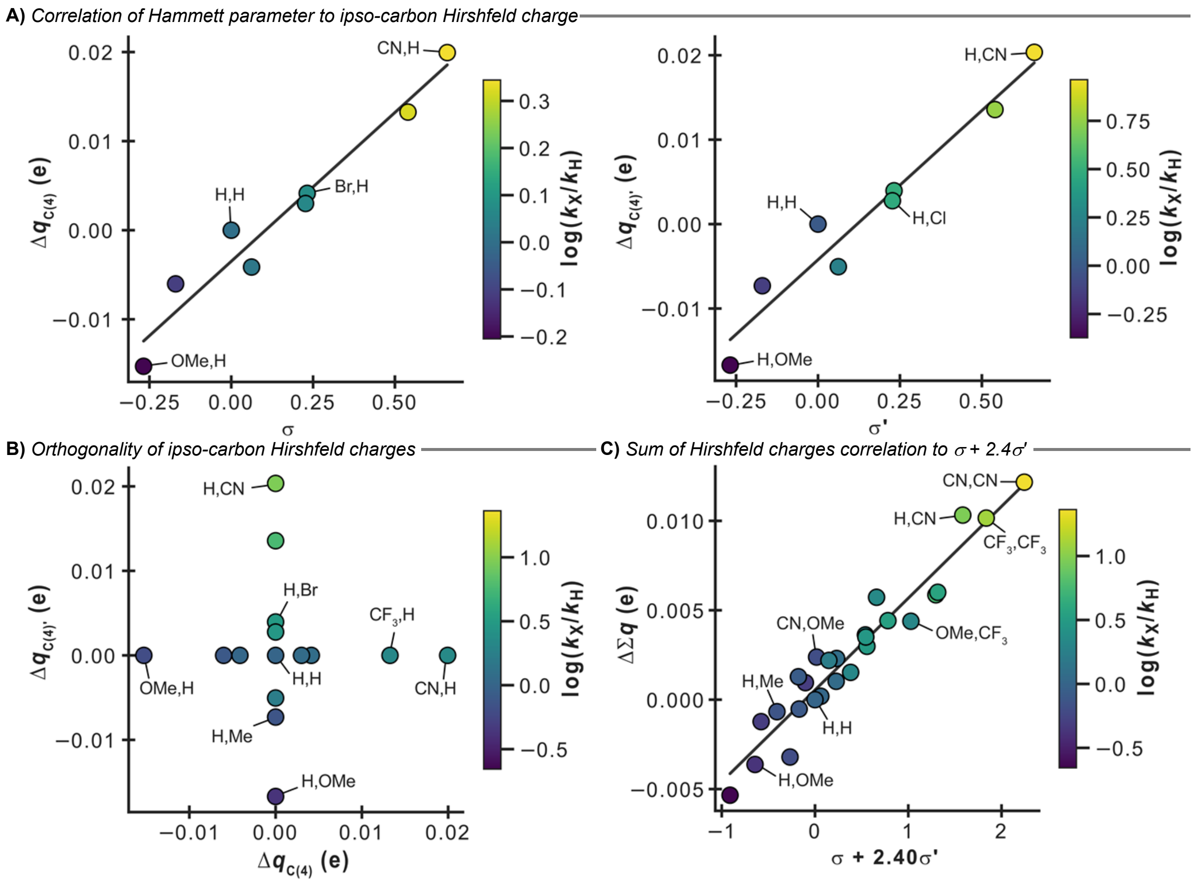

Figure S88. A) Hirshfeld charge (***q***) correlation of *ipso*-carbons, **C(4)** and **C(4)'**, to Hammett parameters and the experimental rates. Hirshfeld charges are displayed relative (**Δ**) to the parent unsubstituted chalcone (Ar = Ar' = Ph). **B)** Orthogonality of the charges for the mono-substituted series of chalcones. **C)** The empirically observed Hammett value for disubstitued chalcones (σ + 2.4σ') correlation to the sum of charge on the chalcone component ($\boldsymbol{\Sigma q}$ = [$\boldsymbol{q_{C(1)}}$ + $\boldsymbol{q_{C(2)}}$ + $\boldsymbol{q_{C(3)}}$ + $\boldsymbol{q_{O(1)}}$]), relative (**Δ**) to the parent unsubstituted chalcone.

Transition states were located for a subset of the chalcones, and the behaviour of the transition state was found to be consistent with the Hammond postulate and Bell-Evans-Polanyi principle, whereby lower enthalpic barriers are found for reactions with stronger enthalpic driving forces and a correspondingly earlier (*i.e.* more reactant-like) transition state (**Figure S89**). Enthalpies were found to correlate well to the experimental rates, though after inclusion of the entropic component, Gibbs free energies exhibited a poorer correlation with $R^2 = 0.94$ between $\boldsymbol{\Delta_r H}$ and $\mathbf{log(}k_X/k_H\mathbf{)}$ compared to 0.87 for $\boldsymbol{\Delta_r G}$ and $\mathbf{log(}k_X/k_H\mathbf{)}$. This may be related to inaccuracies in the rigid-rotor treatment, and evaluation of entropies with *quasi*-harmonic rigid-rotor approximations through GoodVibes v3.2[58] using the Truhlar[59] or Grimme[60] methods (cutoff = 100 cm^{-1}) improve the correlation of $\boldsymbol{\Delta_r G}$ with $\mathbf{log(}k_X/k_H\mathbf{)}$ to $R^2 = 0.95$ and 0.92 respectively.

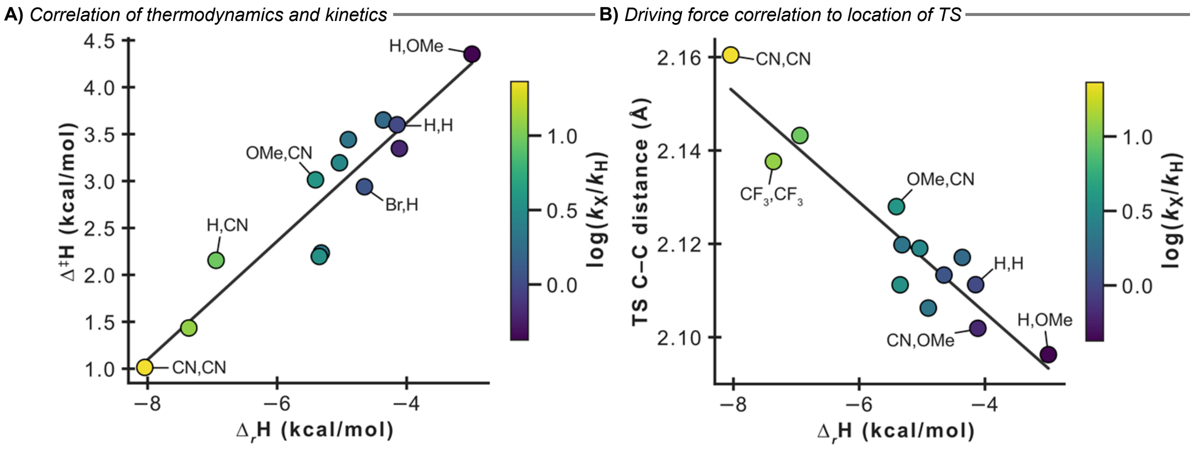

Figure S89. A) Driving force and barrier height correlation, exhibiting Hammond-like behaviour. **B)** Early transition state (longer bond length in TS) correlates to a stronger driving force, with a more reactant-like TS (and *vice-versa*).

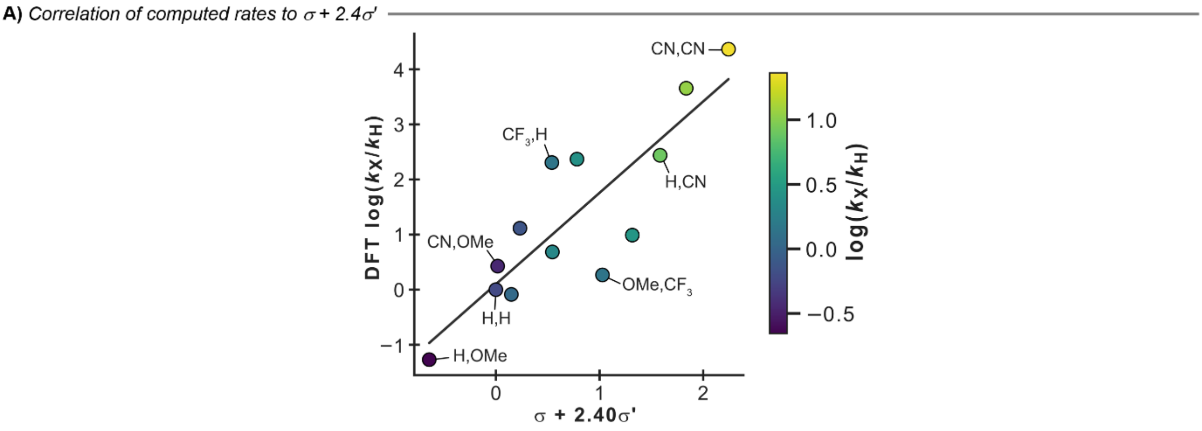

Figure S90. A) Correlation of computed rates (from **Δ‡H**) with combined Hammett parameter.

9. References

1. R. M. Moriarty, R. Penmasta, I. Prakash and A. K. Awasthi, *The Journal of Organic Chemistry*, 1988, **53**, 1022-1025.
2. H. Wang and J. Zeng, *Canadian Journal of Chemistry*, 2009, **87**, 1209-1212.
3. F. Fang, Y. Li and S.-K. Tian, *European Journal of Organic Chemistry*, 2011, **2011**, 1084-1091.
4. R. Rezaie, M. Heidary, M. N. Soltani Rad and S. Behrouz, *Chinese Journal of Chemistry*, 2011, **29**, 1221-1226.
5. A. Stroba, F. Schaeffer, V. Hindie, L. Lopez-Garcia, I. Adrian, W. Frohner, R. W. Hartmann, R. M. Biondi and M. Engel, *J Med Chem*, 2009, **52**, 4683-4693.
6. R. U. Braun, M. Ansorge and T. J. J. Müller, *Chemistry – A European Journal*, 2006, **12**, 9081-9094.
7. Y.-C. Guo, K.-W. Chiu and R.-J. Chein, *The Journal of Organic Chemistry*, 2023, **88**, 559-572.
8. X.-F. Wu, H. Neumann, A. Spannenberg, T. Schulz, H. Jiao and M. Beller, *Journal of the American Chemical Society*, 2010, **132**, 14596-14602.
9. P. R. Salokhe, K. Mote, A. G. Suryavanshi and R. Salunkhe, *ChemInform*, 2009, 41.
10. M. J. Hall, S. O. McDonnell, J. Killoran and D. F. O'Shea, *The Journal of Organic Chemistry*, 2005, **70**,

5571-5578.
11. R. S. Sarda, N. W. Jadhav, U. S. Tekale, V. G. Jadhav, R. B. Patil, S. G. Suryawanshi and P. R. Pawar, *Letters in Organic Chemistry*, 2009, **6**, 481-484.
12. J. Cody, S. Mandal, L. Yang and C. J. Fahrni, *Journal of the American Chemical Society*, 2008, **130**, 13023-13032.
13. C. Niu, A. Tuerxuntayi, G. Li, M. Kabas, C.-Z. Dong and H. A. Aisa, *Chinese Chemical Letters*, 2017, **28**, 1533-1538.
14. P.-S. Gao, K. Zhang, M.-M. Yang, S. Xu, H.-M. Sun, J.-L. Zhang, Z.-W. Gao, W.-Q. Zhang and L.-W. Xu, *Chemical Communications*, 2018, **54**, 5074-5077.
15. S. Ducki, D. Rennison, M. Woo, A. Kendall, J. F. D. Chabert, A. T. McGown and N. J. Lawrence, *Bioorganic & Medicinal Chemistry*, 2009, **17**, 7698-7710.
16. J. W. Choi, B. K. Jang, N.-c. Cho, J.-H. Park, S. K. Yeon, E. J. Ju, Y. S. Lee, G. Han, A. N. Pae, D. J. Kim and K. D. Park, *Bioorganic & Medicinal Chemistry*, 2015, **23**, 6486-6496.
17. M. Liu, P. Wilairat and M.-L. Go, *Journal of Medicinal Chemistry*, 2001, **44**, 4443-4452.
18. S. Zhang, L. Wang, X. Feng and M. Bao, *Organic & Biomolecular Chemistry*, 2014, **12**, 7233-7237.
19. F. Hayat, A. Salahuddin, S. Umar and A. Azam, *European Journal of Medicinal Chemistry*, 2010, **45**, 4669-4675.
20. C.-K. Chan, Y.-L. Tsai and M.-Y. Chang, *Tetrahedron*, 2017, **73**, 3368-3376.
21. C. Reichardt, M. Eschner and G. Schäfer, *Journal of Physical Organic Chemistry*, 2001, **14**, 737-751.
22. D. Gornati, R. Ciccone, A. Vinciguerra, S. Ippati, A. Pannaccione, T. Petrozziello, E. Pizzi, A. Hassan, E. Colombo, S. Barbini, M. Milani, C. Caccavone, P. Randazzo, L. Muzio, L. Annunziato, A. Menegon, A. Secondo, E. Mastrangelo, G. Pignataro and P. Seneci, *Journal of Medicinal Chemistry*, 2021, **64**, 8333-8353.
23. X.-Y. Yang, Y.-X. Jia, W. S. Tay, Y. Li, S. A. Pullarkat and P.-H. Leung, *Dalton Transactions*, 2016, **45**, 13449-13455.
24. W. Tully, L. Main and B. K. Nicholson, *Journal of Organometallic Chemistry*, 2005, **690**, 3348-3356.
25. Y. Liu, C. Wang, Y. Tong, Y. Ling, C. Zhou and B. Xiong, *Advanced Synthesis & Catalysis*, 2021, **363**, 4422-4429.
26. K. Nicholson, T. Langer and S. P. Thomas, *Organic Letters*, 2021, **23**, 2498-2504.
27. D. G. Stark, L. C. Morrill, P.-P. Yeh, A. M. Z. Slawin, T. J. C. O'Riordan and A. D. Smith, *Angewandte Chemie International Edition*, 2013, **52**, 11642-11646.
28. D. von der Heiden, S. Bozkus, M. Klussmann and M. Breugst, *The Journal of Organic Chemistry*, 2017, **82**, 4037-4043.
29. B. I. Roman, T. De Ryck, A. Patronov, S. H. Slavov, B. W. A. Vanhoecke, A. R. Katritzky, M. E. Bracke and C. V. Stevens, *European Journal of Medicinal Chemistry*, 2015, **101**, 627-639.
30. N. Upadhyay, K. Tilekar, F. Loiodice, N. Y. Anisimova, T. S. Spirina, D. V. Sokolova, G. B. Smirnova, J.-y. Choe, F.-J. Meyer-Almes, V. S. Pokrovsky, A. Lavecchia and C. S. Ramaa, *Bioorganic Chemistry*, 2021, **107**, 104527.
31. A. P. G. Nikalje, S. V. Tiwari, J. N. Sangshetti and M. D. Damale, *Research on Chemical Intermediates*, 2018, **44**, 3031-3059.
32. P. Kumar, N. Kaur, R. Kumar and P. Banerjee, *The Journal of Organic Chemistry*, 2022, **87**, 7167-7178.
33. C.-J. Zheng, S.-M. Jiang, Z.-H. Chen, B.-J. Ye and H.-R. Piao, *Archiv der Pharmazie*, 2011, **344**, 689-695.
34. A. Sultan, S. Shajahan, T. Ahamad, S. M. Alshehri, N. Sajjad, N. Mehr un, M. H. U. Rehman, L. Torun, M. Khalid and R. Acevedo, *Monatshefte für Chemie - Chemical Monthly*, 2020, **151**, 123-133.
35. Z. Li, H. Zhao, H. Han, Y. Liu, J. Song, W. Guo, W. Chu and Z. Sun, *Tetrahedron Letters*, 2017, **58**, 3984-

3988.

36. F. Dumas, M. Sylla, D. Joseph, E. Chevallier and C. Camara, *Synthesis*, 2006, DOI: 10.1055/s-2006-926344, 1045-1049.
37. M. Weber, W. Frey and R. Peters, *Angewandte Chemie International Edition*, 2013, **52**, 13223-13227.
38. E. Sánchez-Larios, K. Thai, F. Bilodeau and M. Gravel, *Organic Letters*, 2011, **13**, 4942-4945.
39. Y.-C. Wu, L. Liu, H.-J. Li, D. Wang and Y.-J. Chen, *The Journal of Organic Chemistry*, 2006, **71**, 6592-6595.
40. C. Xu, X. Bai, J. Xu, J. Ren, Y. Xing, Z. Li, J. Wang, J. Shi, L. Yu and Y. Wang, *RSC Advances*, 2017, **7**, 4763-4775.
41. S. Zhang, J. E. Taylor, A. M. Z. Slawin and A. D. Smith, *Organic Letters*, 2018, **20**, 5482-5485.
42. H. Stetter and M. Schreckenberg, *Chemische Berichte*, 1974, **107**, 2453-2458.
43. B.-C. Hong, N. S. Dange, C.-S. Hsu, J.-H. Liao and G.-H. Lee, *Organic Letters*, 2011, **13**, 1338-1341.
44. Y. Zhao and D. G. Truhlar, *Theor. Chem. Acc.*, 2008, **120**, 215–241.
45. Schäfer, H. Horn and R. Ahlrichs*, J. Chem. Phys.*, 1992, **97**, 2571–2577.
46. Schäfer, C. Huber and R. Ahlrichs, *J. Chem. Phys.*, 1994, **100**, 5829–5835.
47. F. Weigend and R. Ahlrichs, *Phys. Chem. Chem. Phys.*, 2005, **7**, 3297–3305.
48. F. Weigend, *Phys. Chem. Chem. Phys.*, 2006, **8**, 1057–1065.
49. V. Marenich, C. J. Cramer and D. G. Truhlar, *J. Phys. Chem. B*, 2009, **113**, 6378–6396.
50. R. L. Martin, P. J. Hay and L. R. Pratt, *J. Phys. Chem. A*, 1998, **102**, 3565–3573.
51. F. L. Hirshfeld, *Theor. Chim. Acta*, 1977, **44**, 129–138.
52. M. J. Frisch, G. W. Trucks, H. B. Schlegel, G. E. Scuseria, M. A. Robb, J. R. Cheeseman, G. Scalmani, V. Barone, G. A. Petersson, H. Nakatsuji, X. Li, M. Caricato, A. V Marenich, J. Bloino, B. G. Janesko, R. Gomperts, B. Mennucci, H. P. Hratchian, J. V Ortiz, A. F. Izmaylov, J. L. Sonnenberg, D. Williams-Young, F. Ding, F. Lipparini, F. Egidi, J. Goings, B. Peng, A. Petrone, T. Henderson, D. Ranasinghe, V. G. Zakrzewski, J. Gao, N. Rega, G. Zheng, W. Liang, M. Hada, M. Ehara, K. Toyota, R. Fukuda, J. Hasegawa, M. Ishida, T. Nakajima, Y. Honda, O. Kitao, H. Nakai, T. Vreven, K. Throssell, J. A. Montgomery, Jr., J. E. Peralta, F. Ogliaro, M. J. Bearpark, J. J. Heyd, E. N. Brothers, K. N. Kudin, V. N. Staroverov, T. A. Keith, R. Kobayashi, J. Normand, K. Raghavachari, A. P. Rendell, J. C. Burant, S. S. Iyengar, J. Tomasi, M. Cossi, J. M. Millam, M. Klene, C. Adamo, R. Cammi, J. W. Ochterski, R. L. Martin, K. Morokuma, O. Farkas, J. B. Foresman and D. J. Fox, *Gaussian 16, Revision C.01*, Gaussian Inc., Wallingford CT, 2019.
53. Y. Legault, Univ. Sherbrooke, 2020, www.cylview.org.
54. Bannwarth, S. Ehlert and S. Grimme, *J. Chem. Theory Comput.*, 2019, **15**, 1652–1671.
55. P. Pracht, F. Bohle and S. Grimme, *Phys. Chem. Chem. Phys.*, 2020, **22**, 7169–7192.
56. J. Tao, J. P. Perdew, V. N. Staroverov and G. E. Scuseria, *Phys. Rev. Lett.*, 2003, **91**, 146401.
57. G. Luchini and R. S. Paton, *ACS Phys. Chem. Au*, 2024, **4**, 259–267.
58. G. Luchini, J. V Alegre-Requena, I. Funes-Ardoiz and R. S. Paton, *F1000Research (Chem. Inf. Sci.)*, 2020, **9**, 291.
59. R. F. Ribeiro, A. V. Marenich, C. J. Cramer and D. G. Truhlar, *J. Phys. Chem. B*, 2011, **115**, 14556–14562.
60. S. Grimme, *Chem. - A Eur. J.*, 2012, **18**, 9955–9964.

10. ^{1}H, C^{13}{^{1}H}, F^{19}{^{1}H} NMR Spectra

1. Michael Acceptors

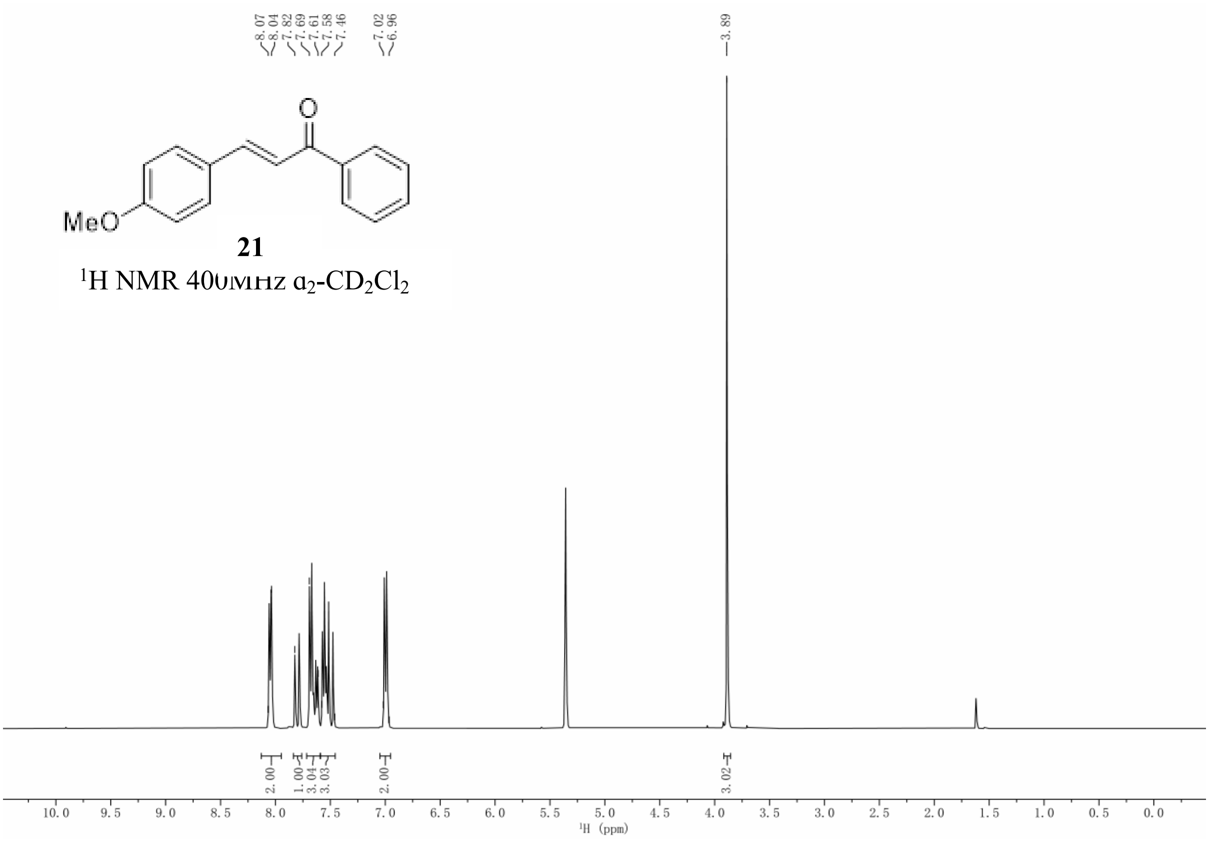

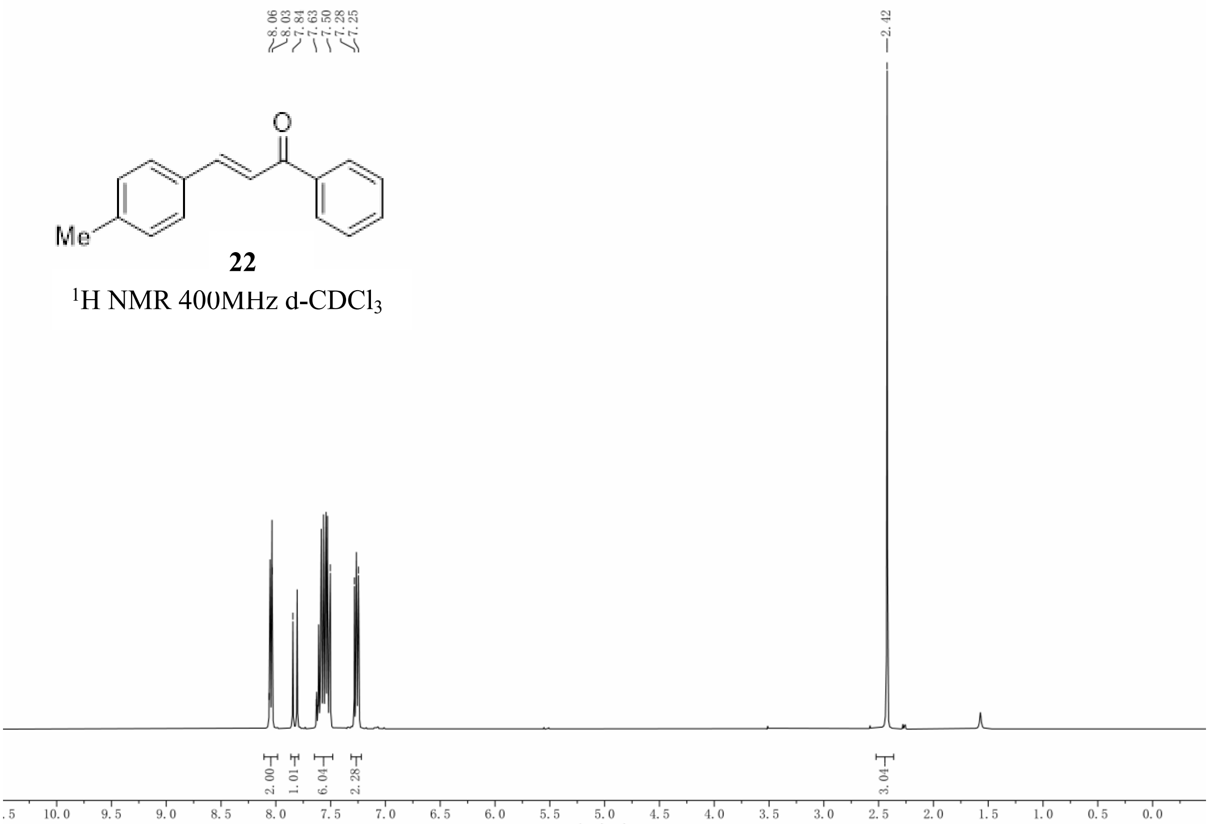

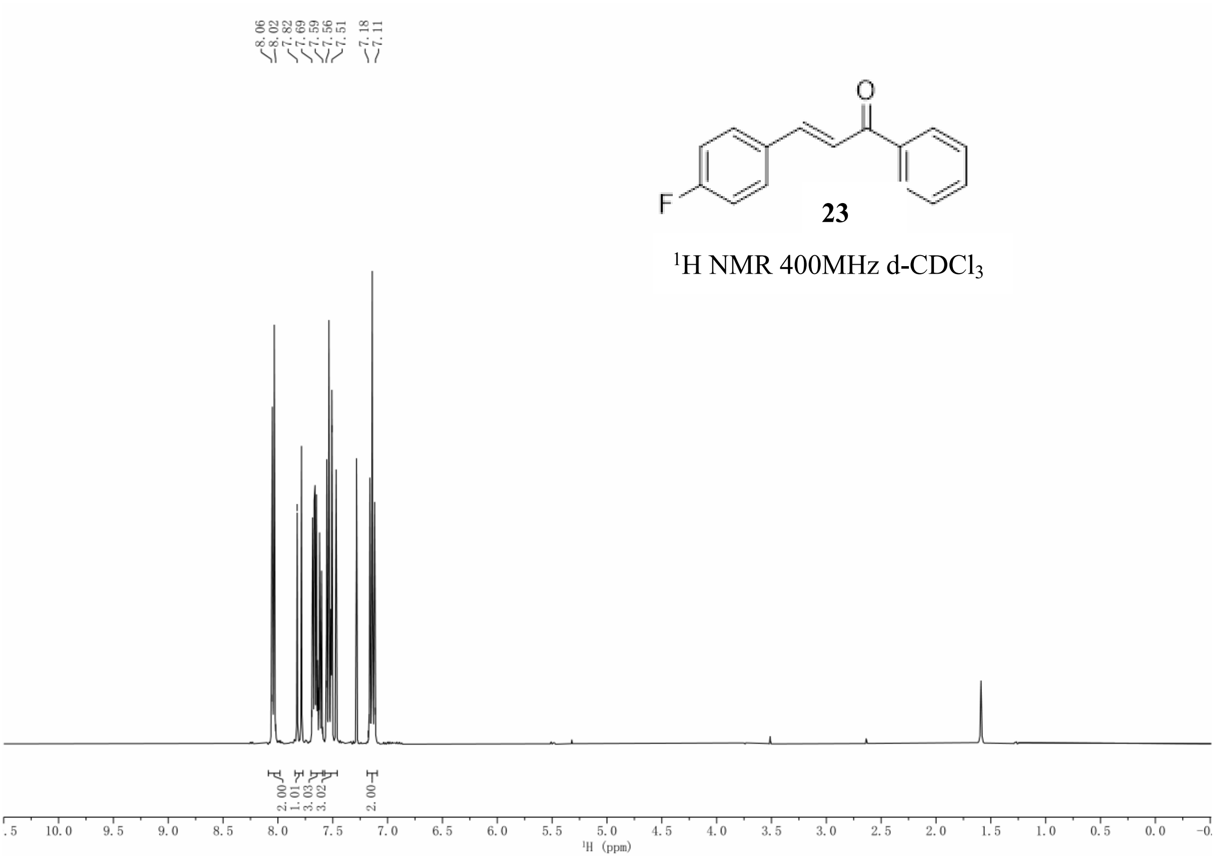
8.06 8.02 7.82 7.69 7.59 7.56 7.51 7.18 7.11
23
1H NMR 400MHz d-CDCl3
2.00 1.01 3.03 3.02 2.00
1H (ppm)

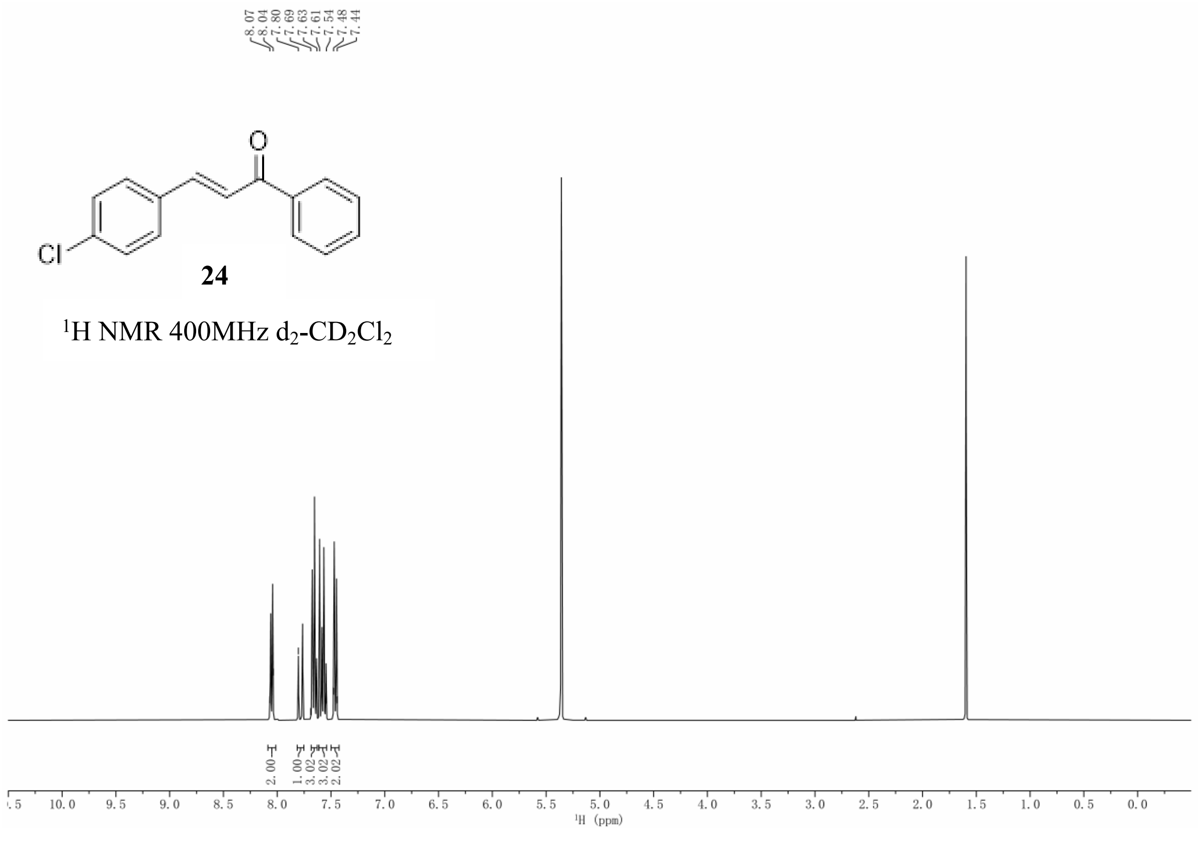
8.07 8.04 7.80 7.69 7.63 7.61 7.54 7.48 7.44
24
1H NMR 400MHz d2-CD2Cl2
2.00 1.00 3.02 3.02 2.02
1H (ppm)

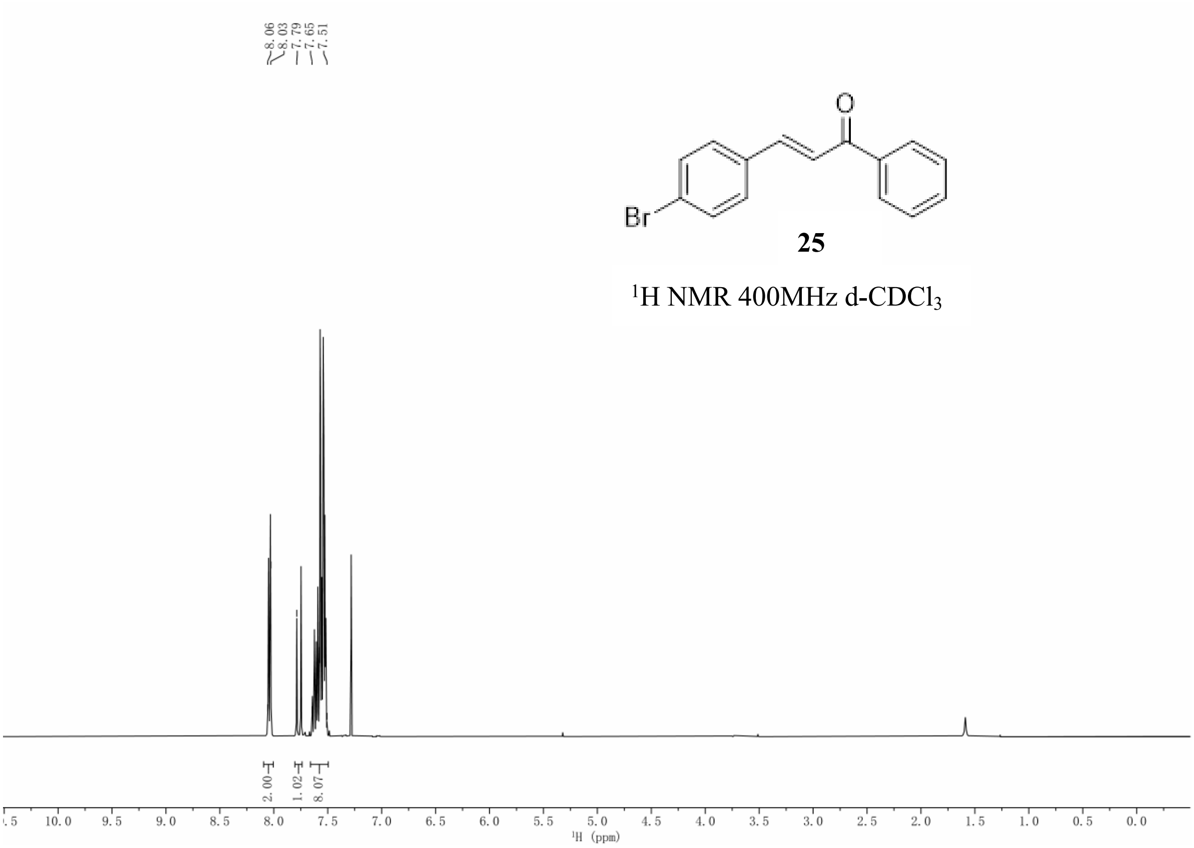
8.06
8.03
7.79
7.65
7.51
Br
O
25
^{1}H NMR 400MHz d-CDCl$_3$
2.00
1.02
8.07
10.0 9.5 9.0 8.5 8.0 7.5 7.0 6.5 6.0 5.5 5.0 4.5 4.0 3.5 3.0 2.5 2.0 1.5 1.0 0.5 0.0
^{1}H (ppm)

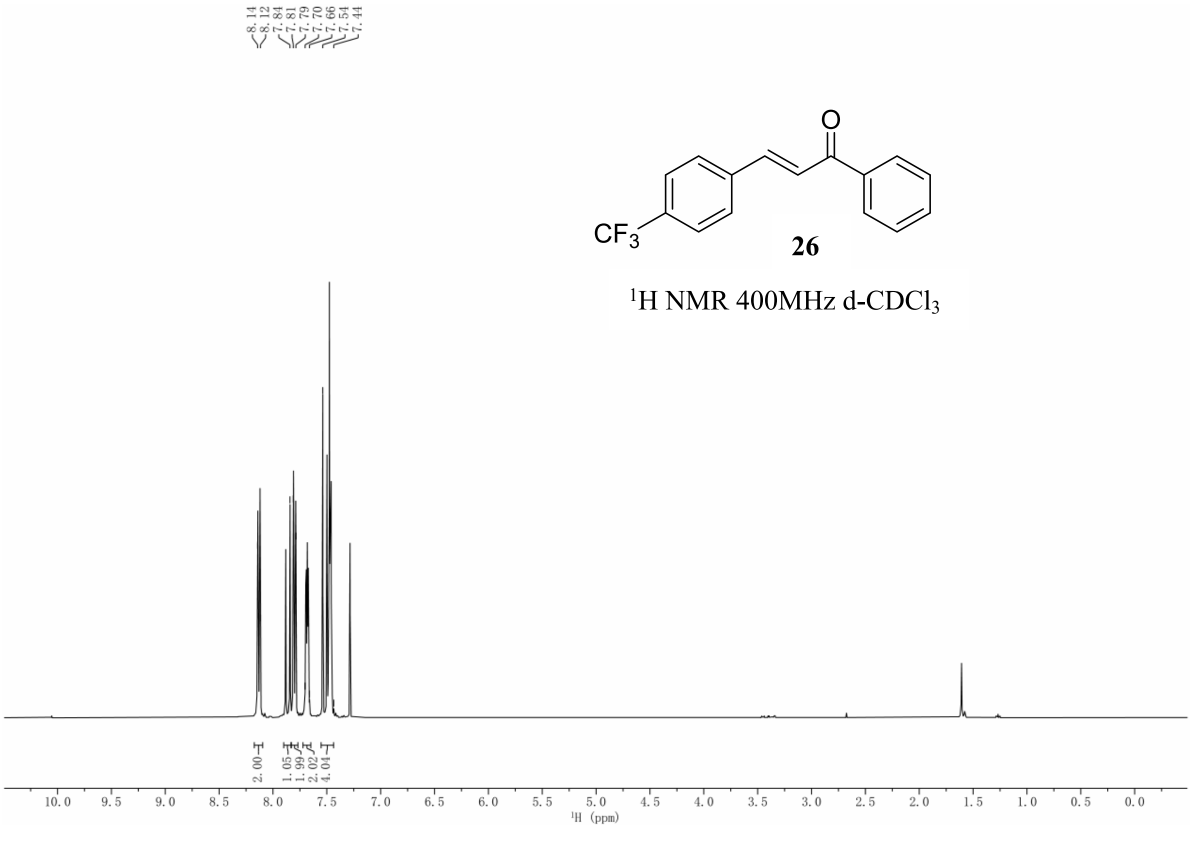
8.14
8.12
7.84
7.81
7.79
7.70
7.66
7.54
7.44
O
CF$_3$
26
^{1}H NMR 400MHz d-CDCl$_3$
2.00
1.05
1.99
2.02
4.04
10.0 9.5 9.0 8.5 8.0 7.5 7.0 6.5 6.0 5.5 5.0 4.5 4.0 3.5 3.0 2.5 2.0 1.5 1.0 0.5 0.0
^{1}H (ppm)

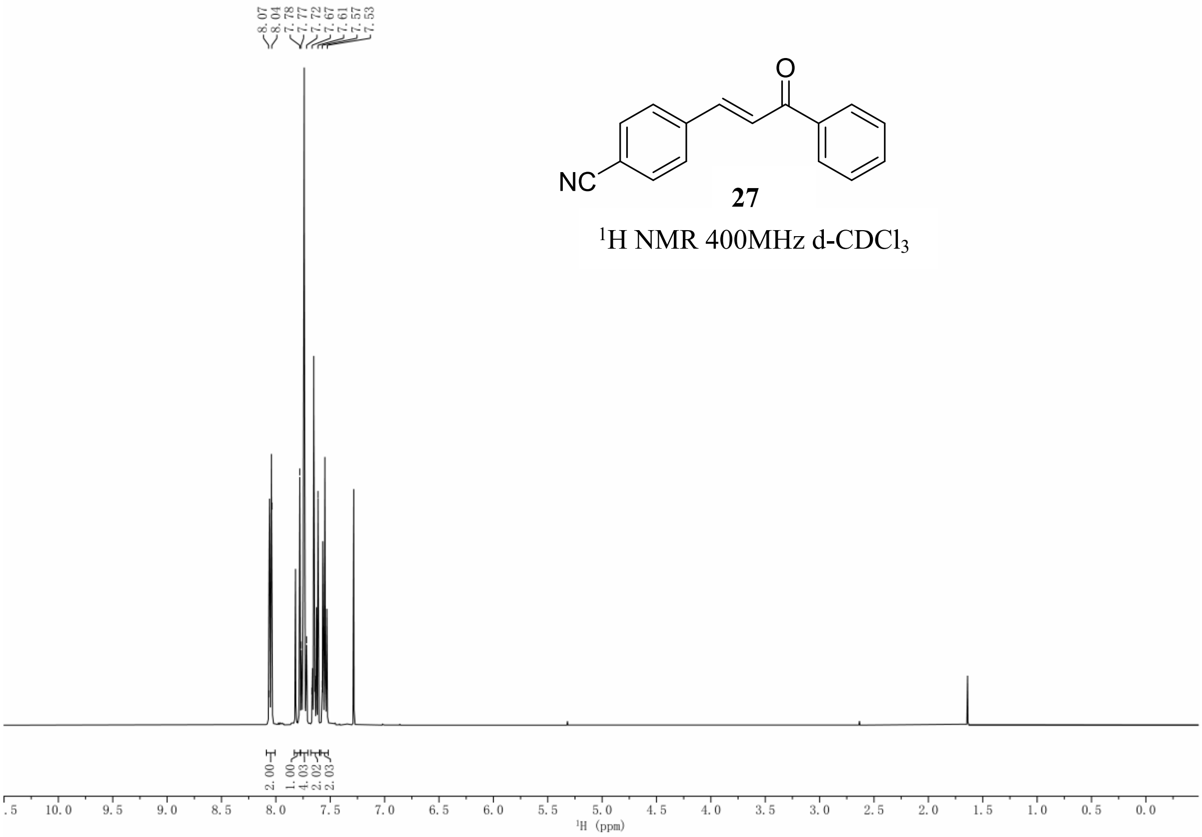
NC
O
27
1H NMR 400MHz d-CDCl3
1H (ppm)

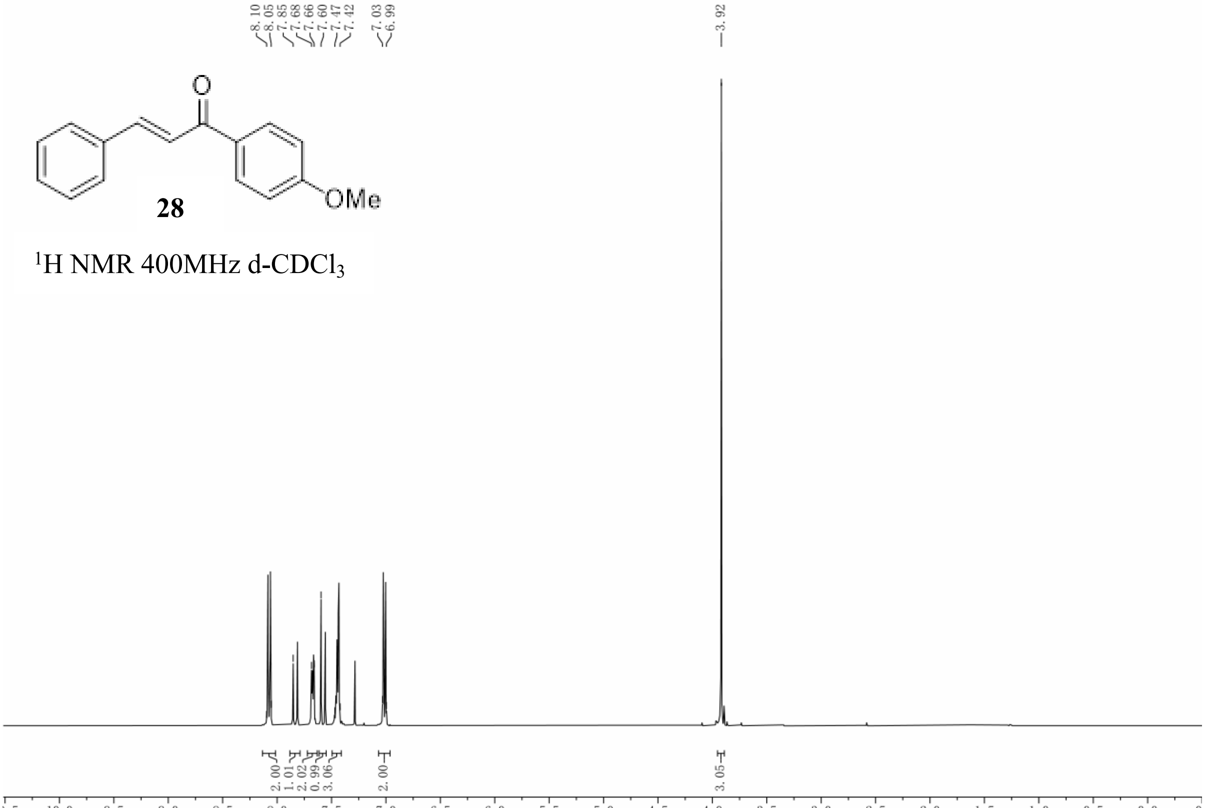
O
OMe
28
1H NMR 400MHz d-CDCl3
1H (ppm)

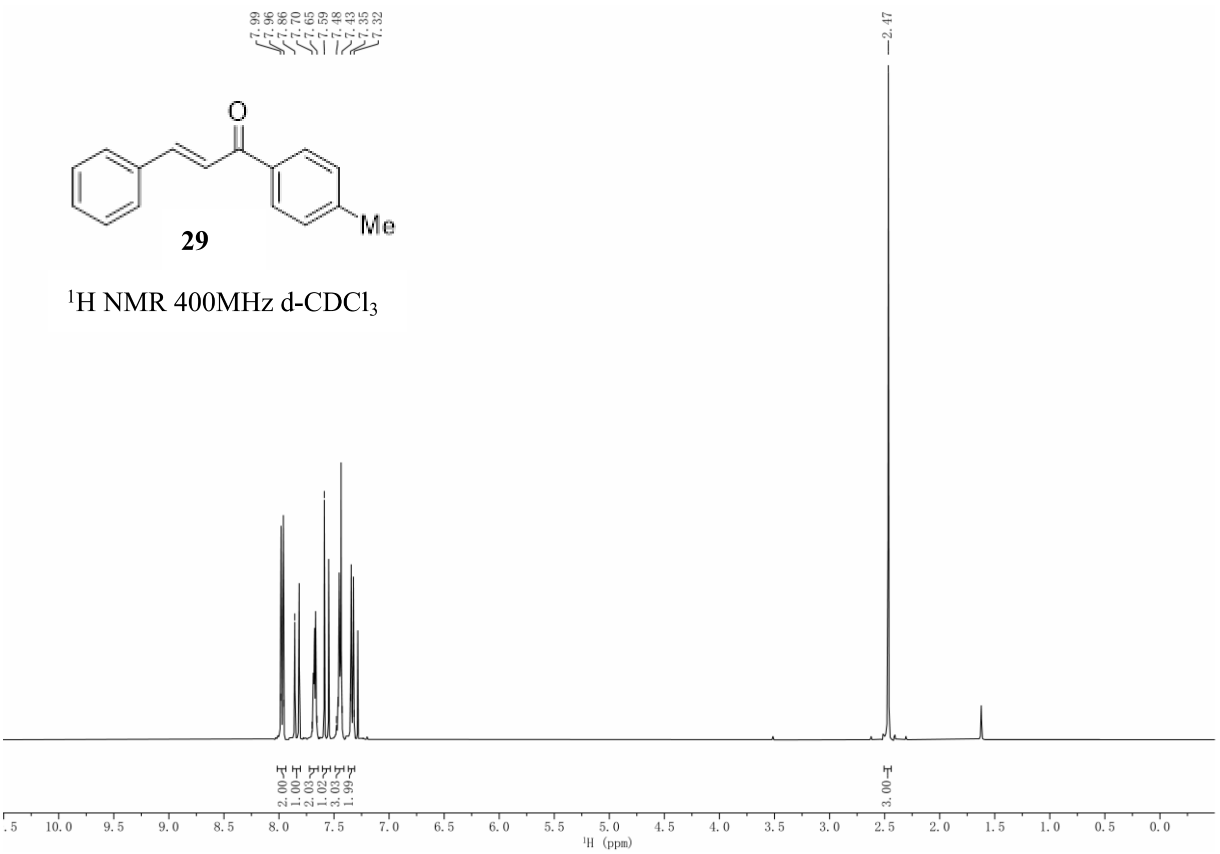
29
^{1}H NMR 400MHz d-CDCl$_3$
Me
O
7.99 7.96 7.86 7.70 7.65 7.59 7.48 7.43 7.35 7.32
2.47
2.00 1.00 2.03 1.02 3.03 1.99
3.00
^{1}H (ppm)

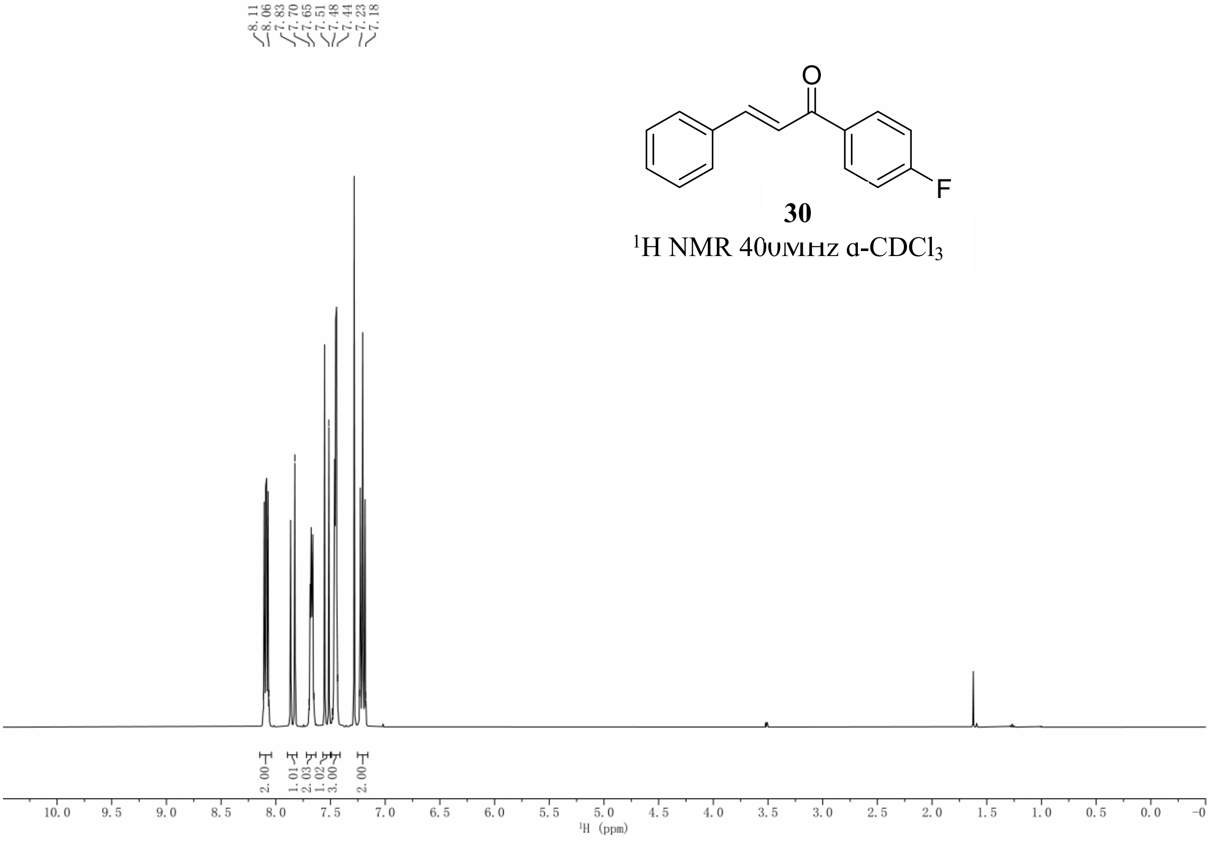
30
^{1}H NMR 40υMHz α-CDCl$_3$
O
F
8.11 8.06 7.83 7.70 7.65 7.51 7.48 7.44 7.23 7.18
2.00 1.01 2.03 1.02 3.00 2.00
^{1}H (ppm)

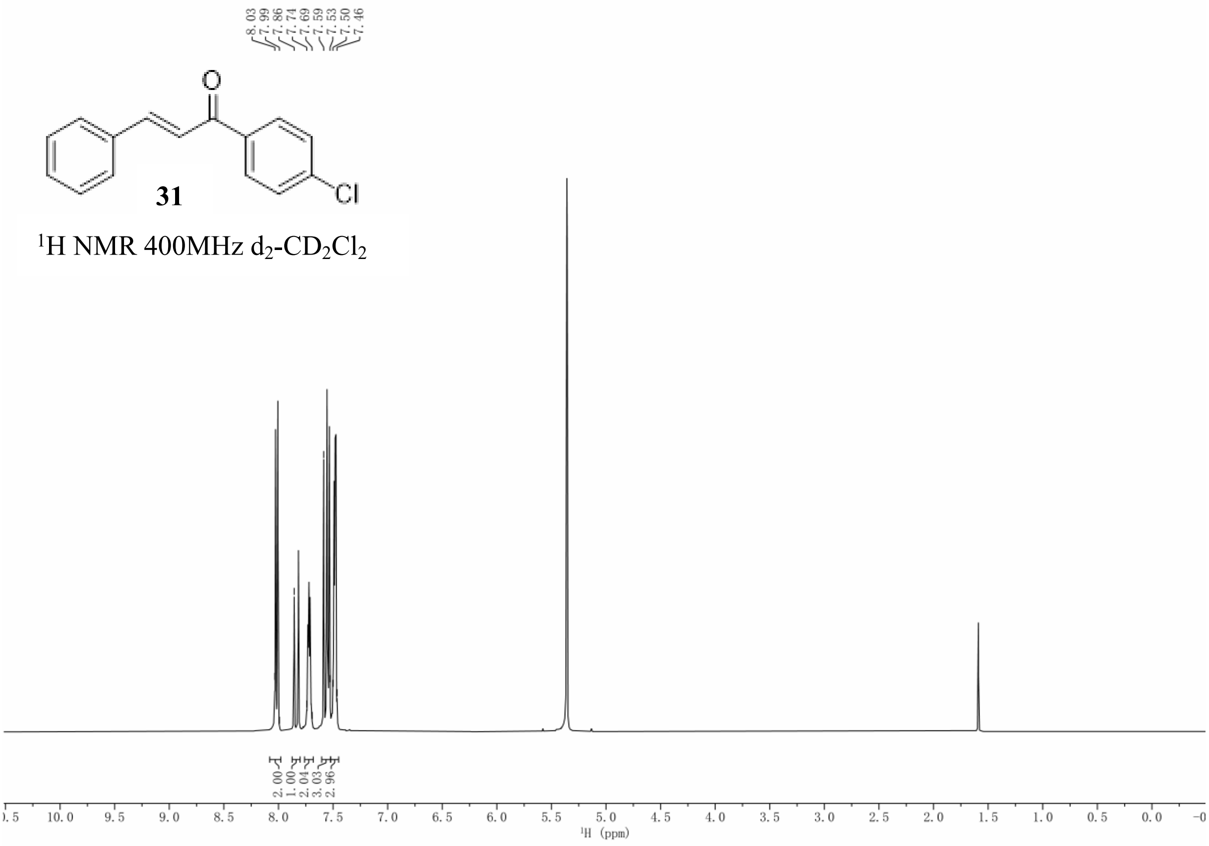
8.03 7.99 7.86 7.74 7.69 7.59 7.53 7.50 7.46
31
Cl
^{1}H NMR 400MHz d_2-CD_2Cl_2
2.00 1.00 2.04 3.03 2.96
^{1}H (ppm)

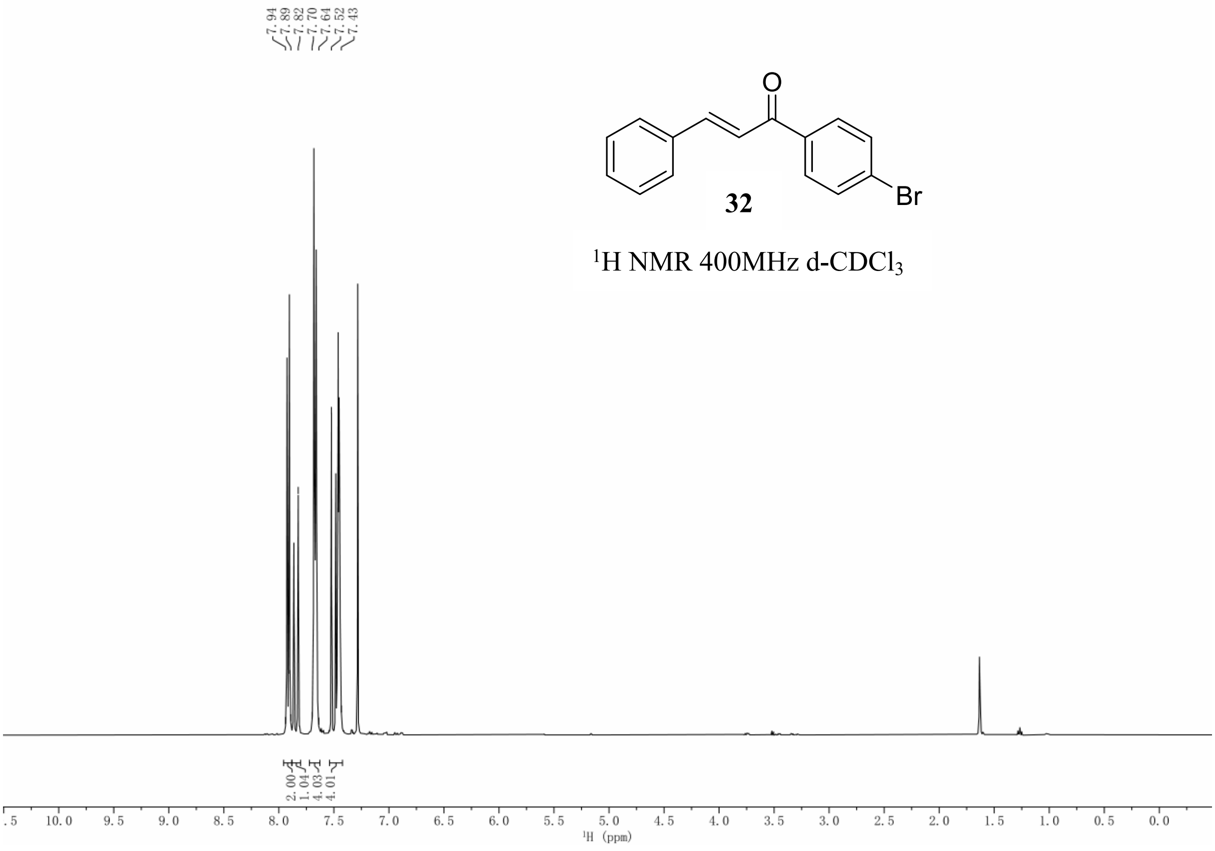
7.94 7.89 7.82 7.70 7.64 7.52 7.43
32
Br
^{1}H NMR 400MHz d-$CDCl_3$
2.00 1.04 4.03 4.01
^{1}H (ppm)

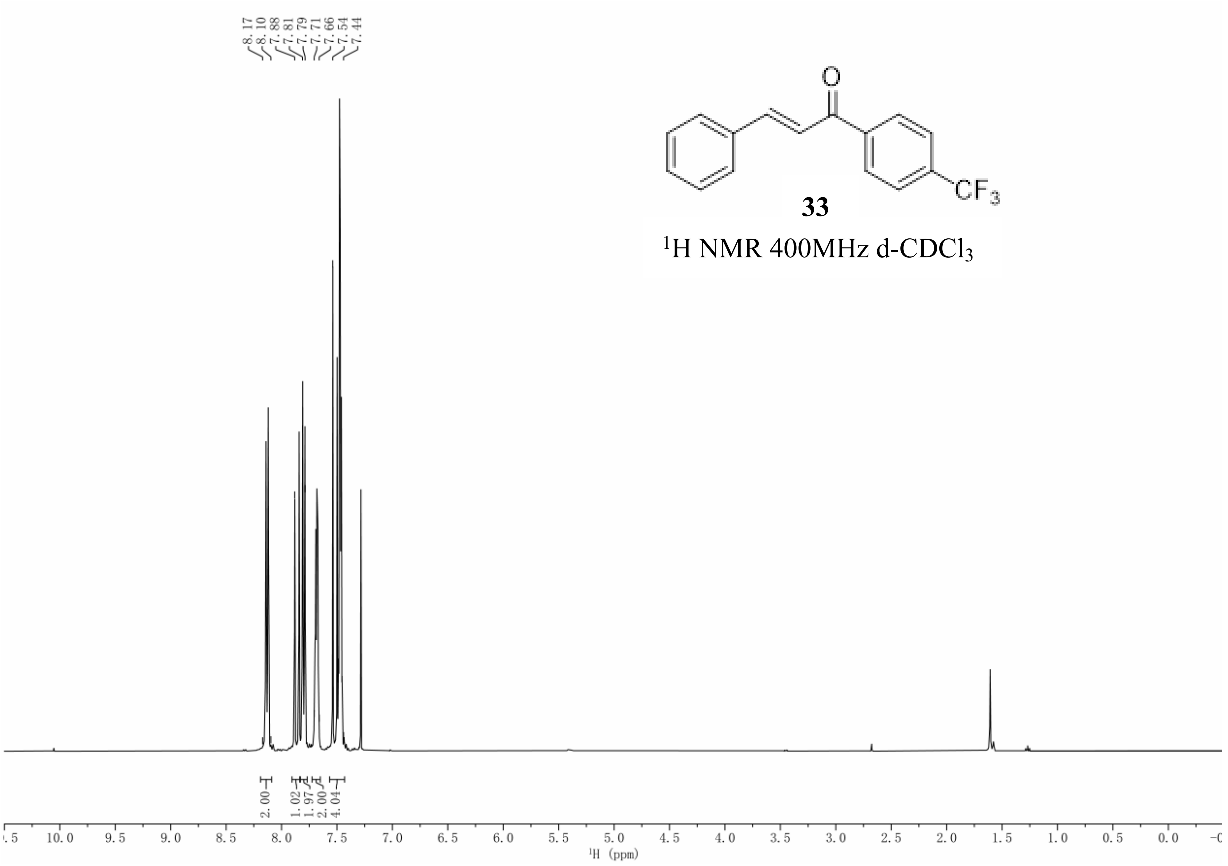
8.17
8.10
7.88
7.81
7.79
7.71
7.66
7.54
7.44
O
CF3
33
1H NMR 400MHz d-CDCl3
2.00
1.02
1.97
2.00
4.04
1H (ppm)

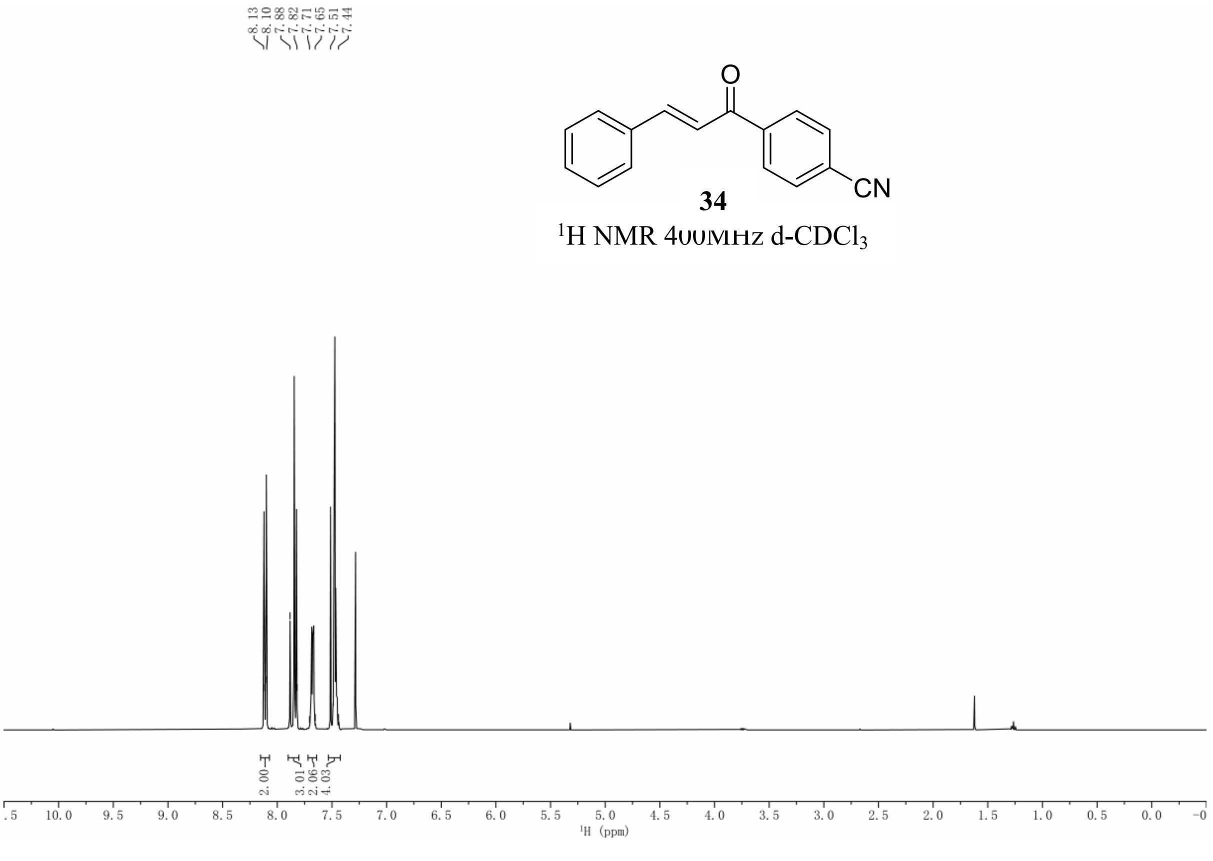
8.13
8.10
7.88
7.82
7.71
7.65
7.51
7.44
O
CN
34
1H NMR 400MHz d-CDCl3
2.00
3.01
2.06
4.03
1H (ppm)

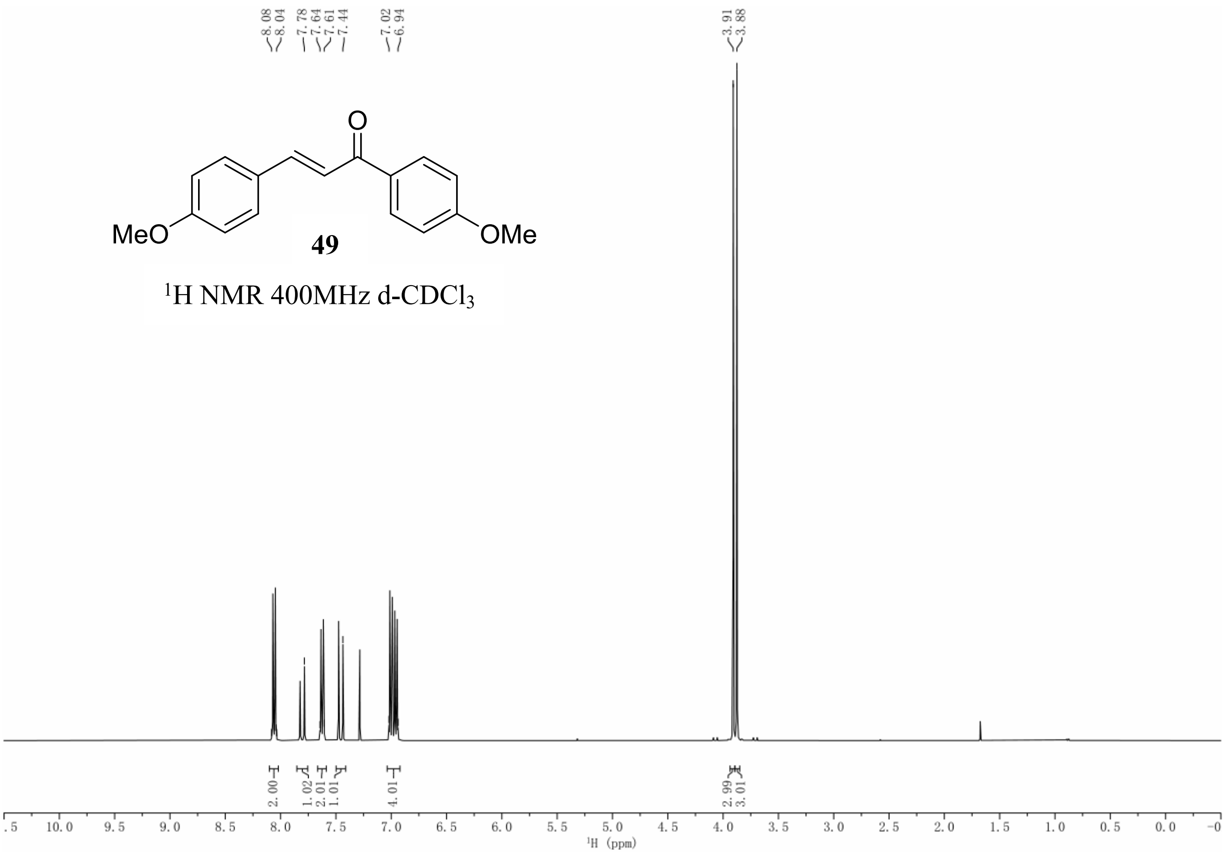
MeO
OMe
49
^{1}H NMR 400MHz d-CDCl$_3$
^{1}H (ppm)

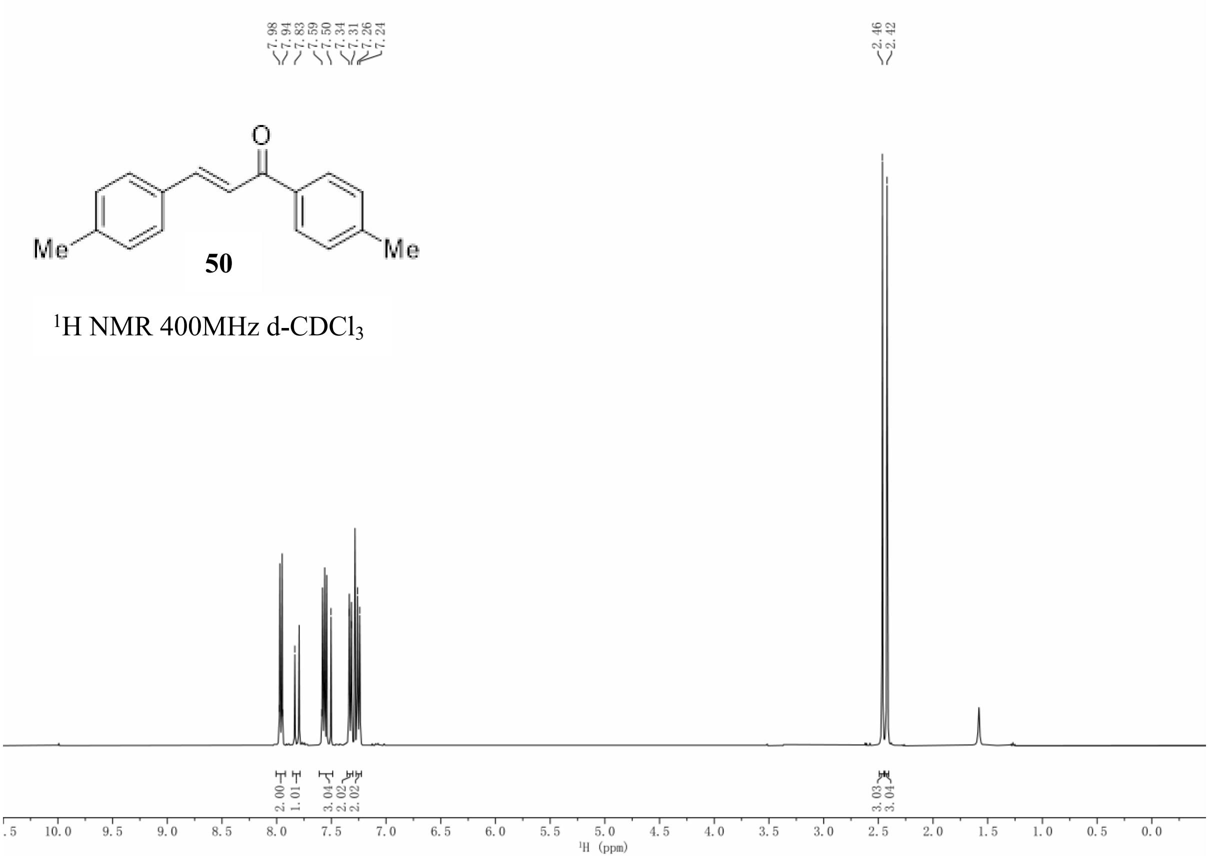
Me
Me
50
^{1}H NMR 400MHz d-CDCl$_3$
^{1}H (ppm)

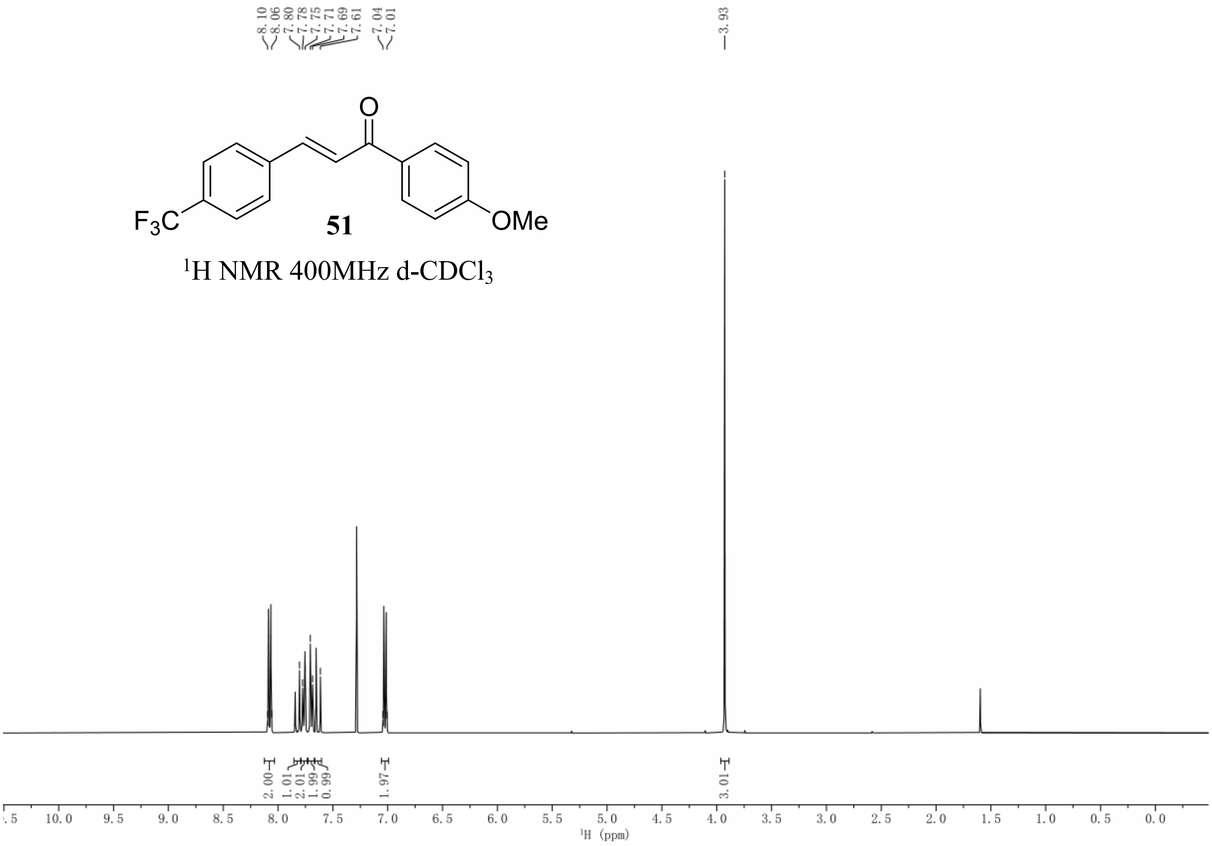
51
¹H NMR 400MHz d-CDCl3
¹H (ppm)

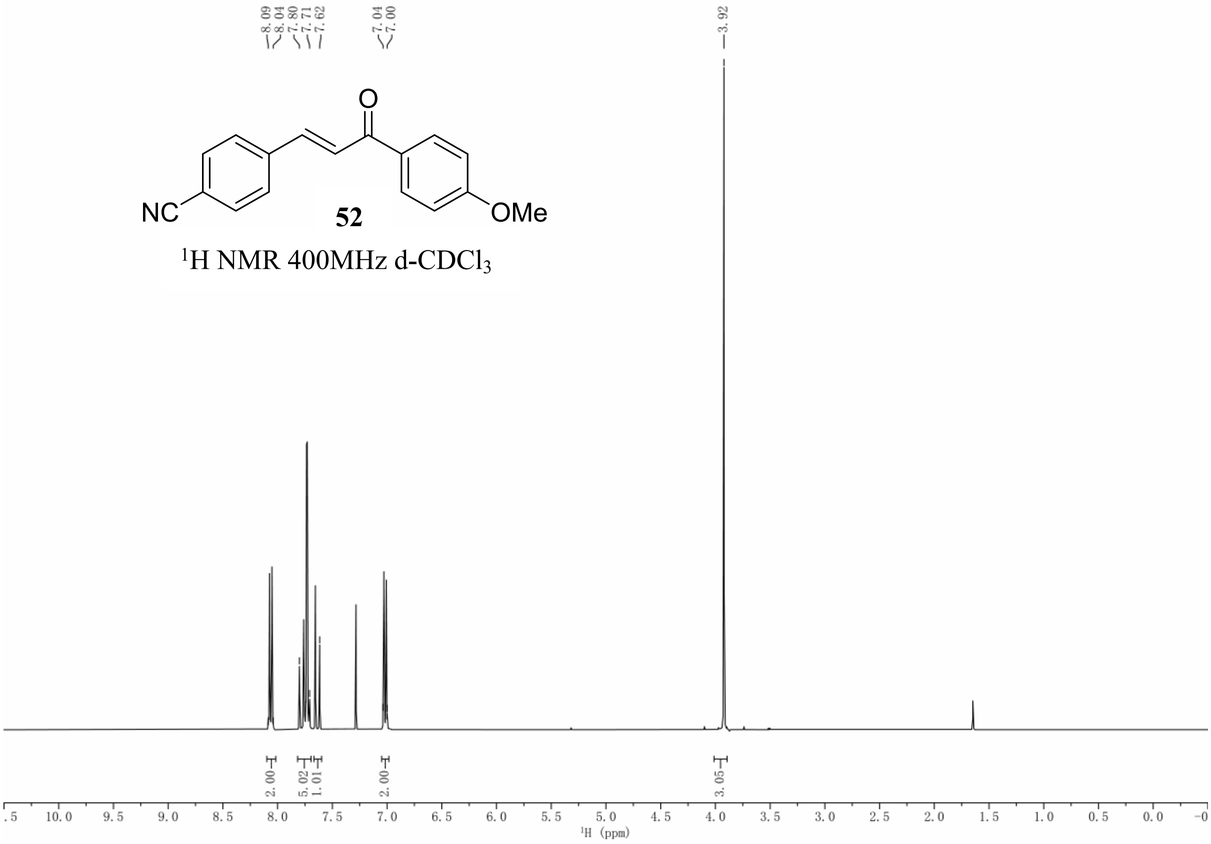
52
¹H NMR 400MHz d-CDCl3
¹H (ppm)

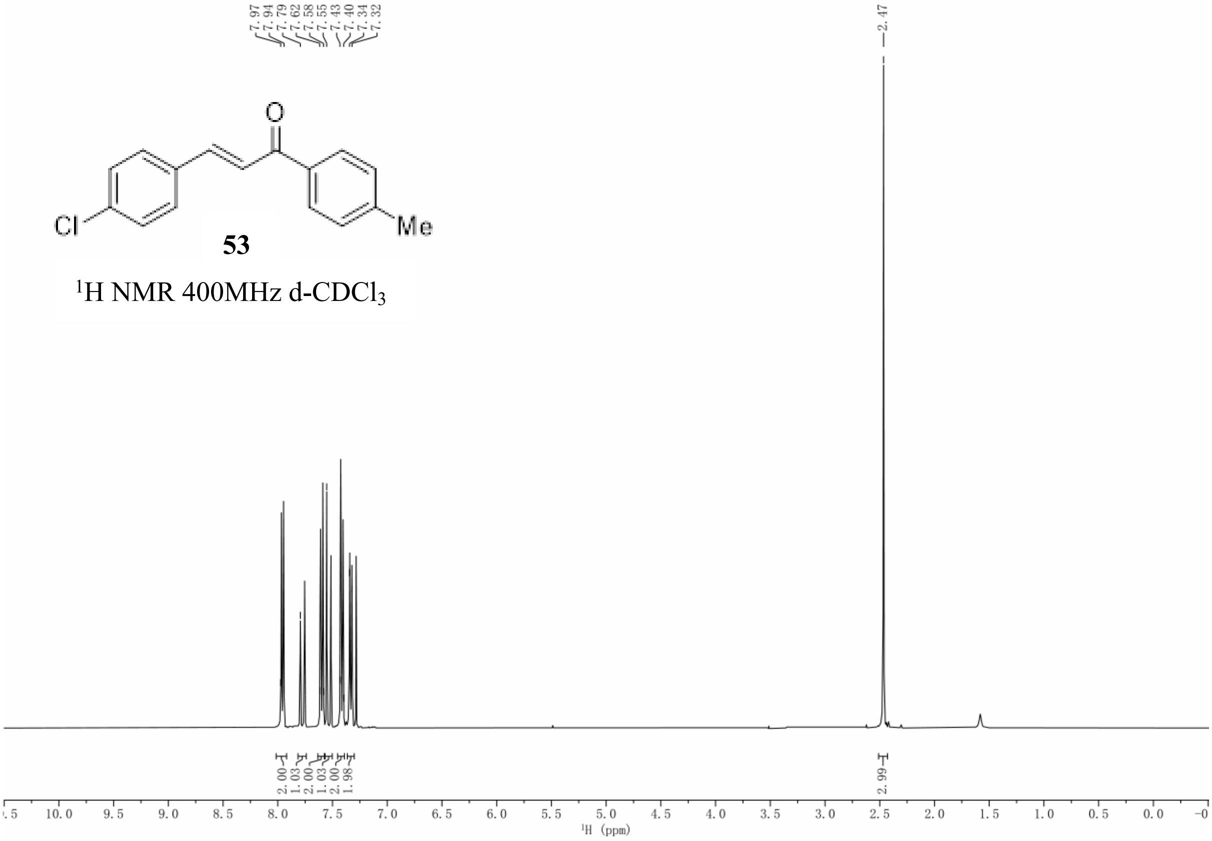
7.97 7.94 7.79 7.62 7.58 7.55 7.43 7.40 7.34 7.32
2.47
O
Cl
Me
53
^{1}H NMR 400MHz d-CDCl$_3$
2.00 1.03 2.00 1.03 2.00 1.98
2.99
^{1}H (ppm)

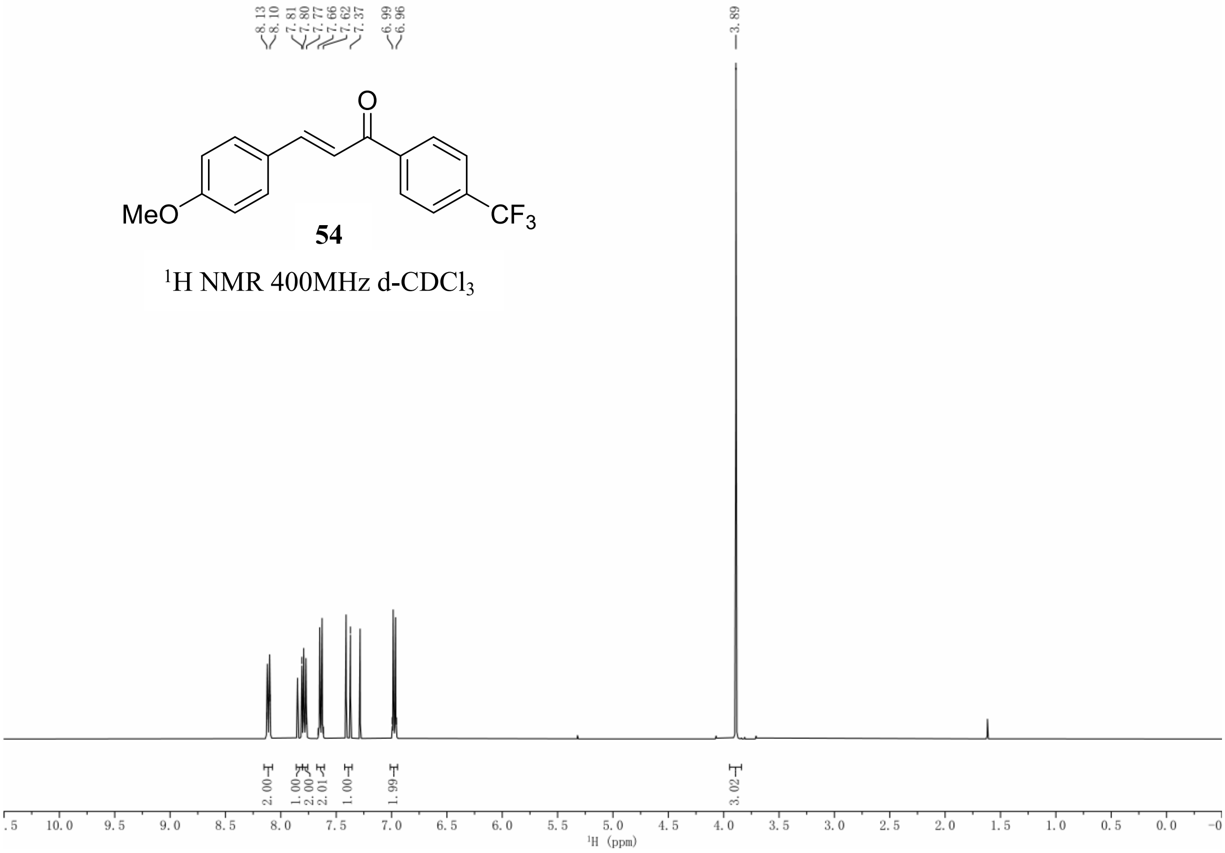
8.13 8.10 7.81 7.80 7.77 7.66 7.62 7.37 6.99 6.96
3.89
O
MeO
CF$_3$
54
^{1}H NMR 400MHz d-CDCl$_3$
2.00 1.00 2.00 2.01 1.00 1.99
3.02
^{1}H (ppm)

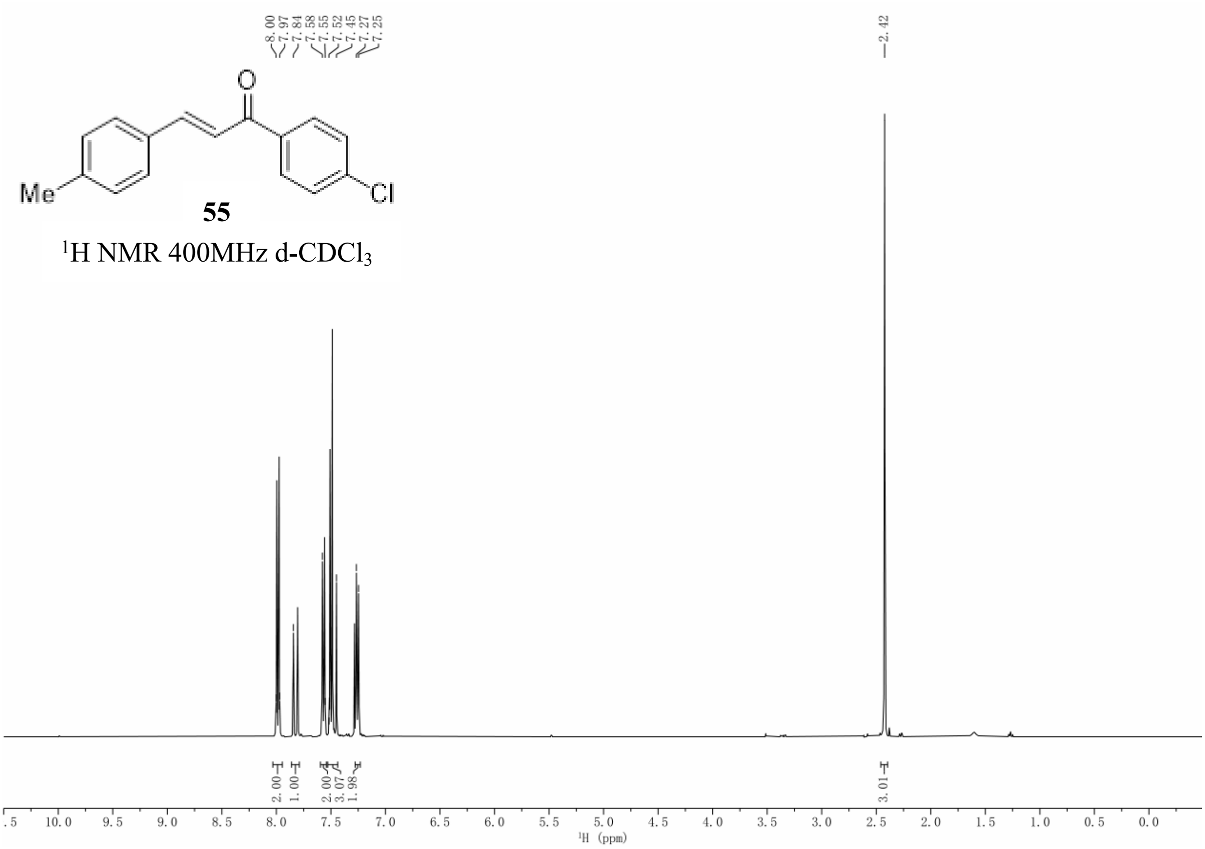
8.00
7.97
7.84
7.58
7.55
7.52
7.45
7.27
7.25
2.42
Me
Cl
55
1H NMR 400MHz d-CDCl3
2.00
1.00
2.00
3.07
1.98
3.01
1H (ppm)

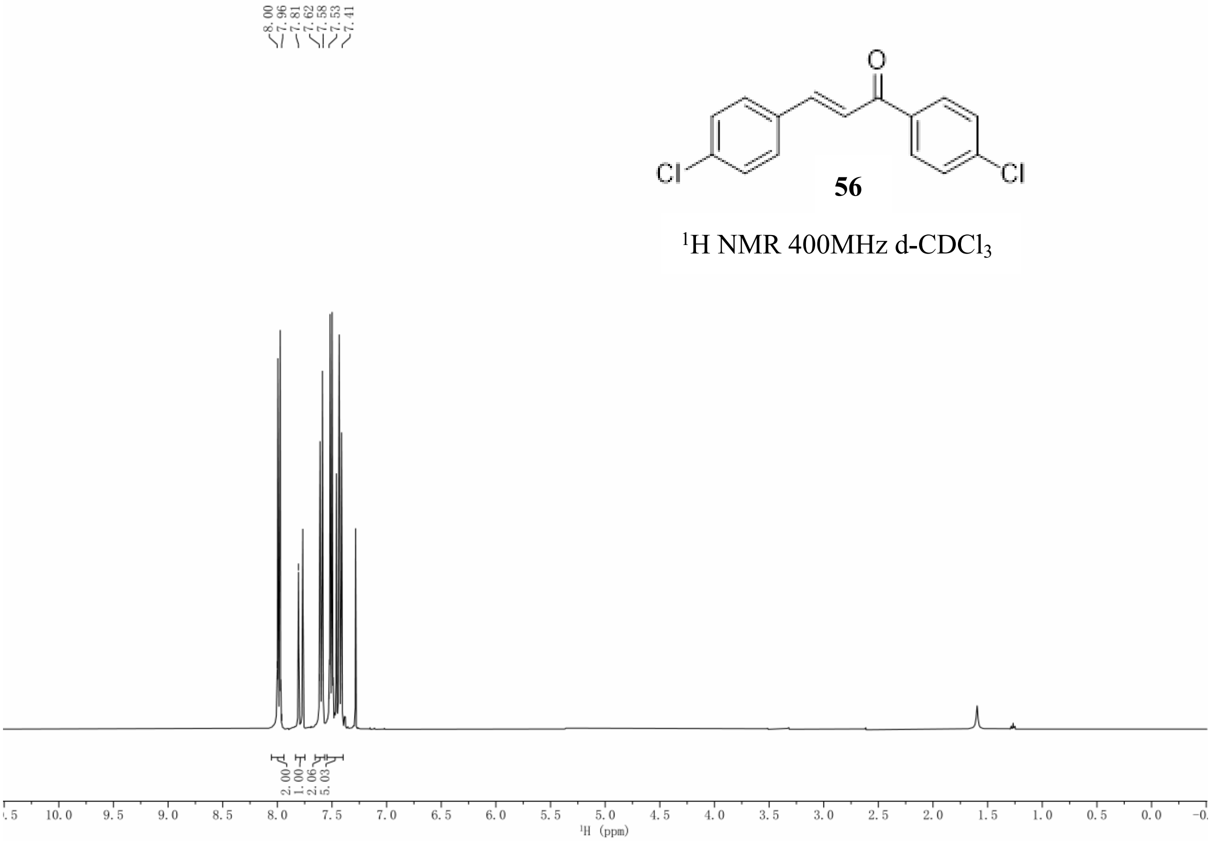
8.00
7.96
7.81
7.62
7.58
7.53
7.41
Cl
Cl
56
1H NMR 400MHz d-CDCl3
2.00
1.00
2.06
5.03
1H (ppm)

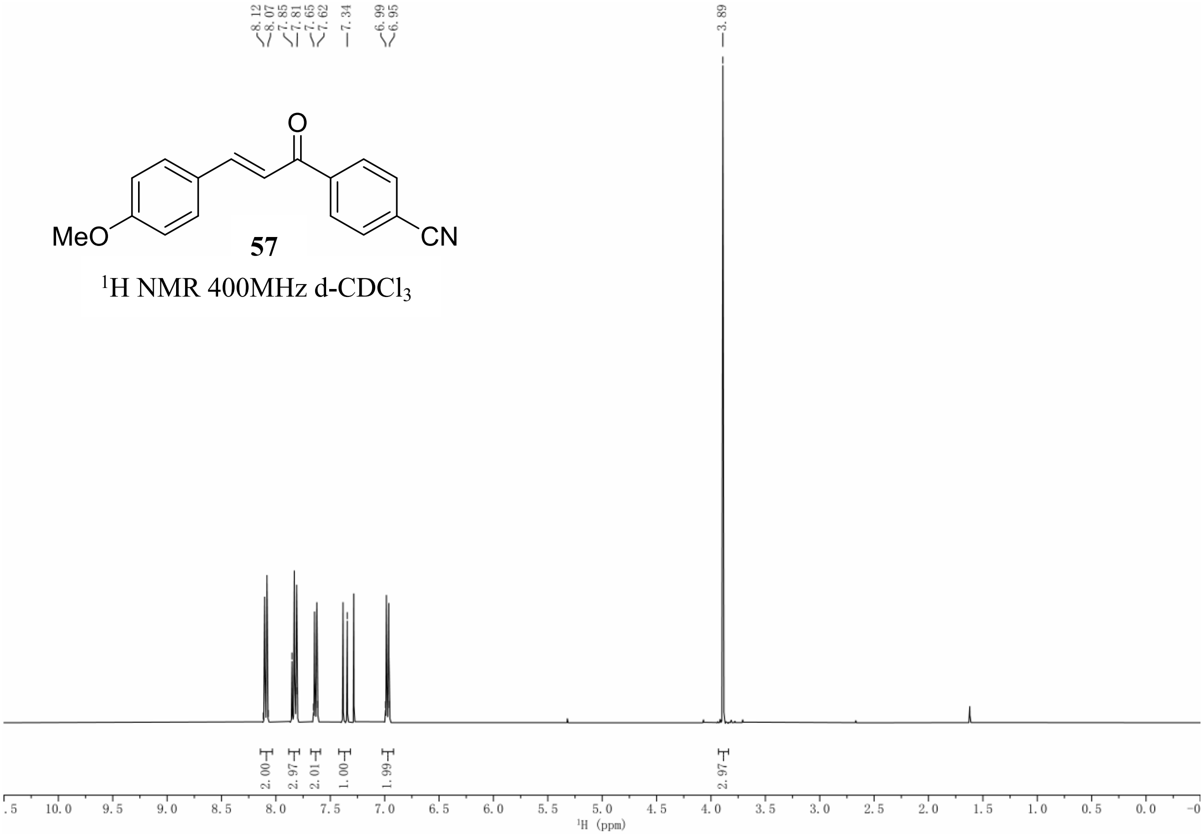
8.12
8.07
7.85
7.81
7.65
7.62
7.34
6.99
6.95
3.89
MeO
CN
57
¹H NMR 400MHz d-CDCl₃
2.00
2.97
2.01
1.00
1.99
2.97
10.0
9.5
9.0
8.5
8.0
7.5
7.0
6.5
6.0
5.5
5.0
4.5
4.0
3.5
3.0
2.5
2.0
1.5
1.0
0.5
0.0
¹H (ppm)

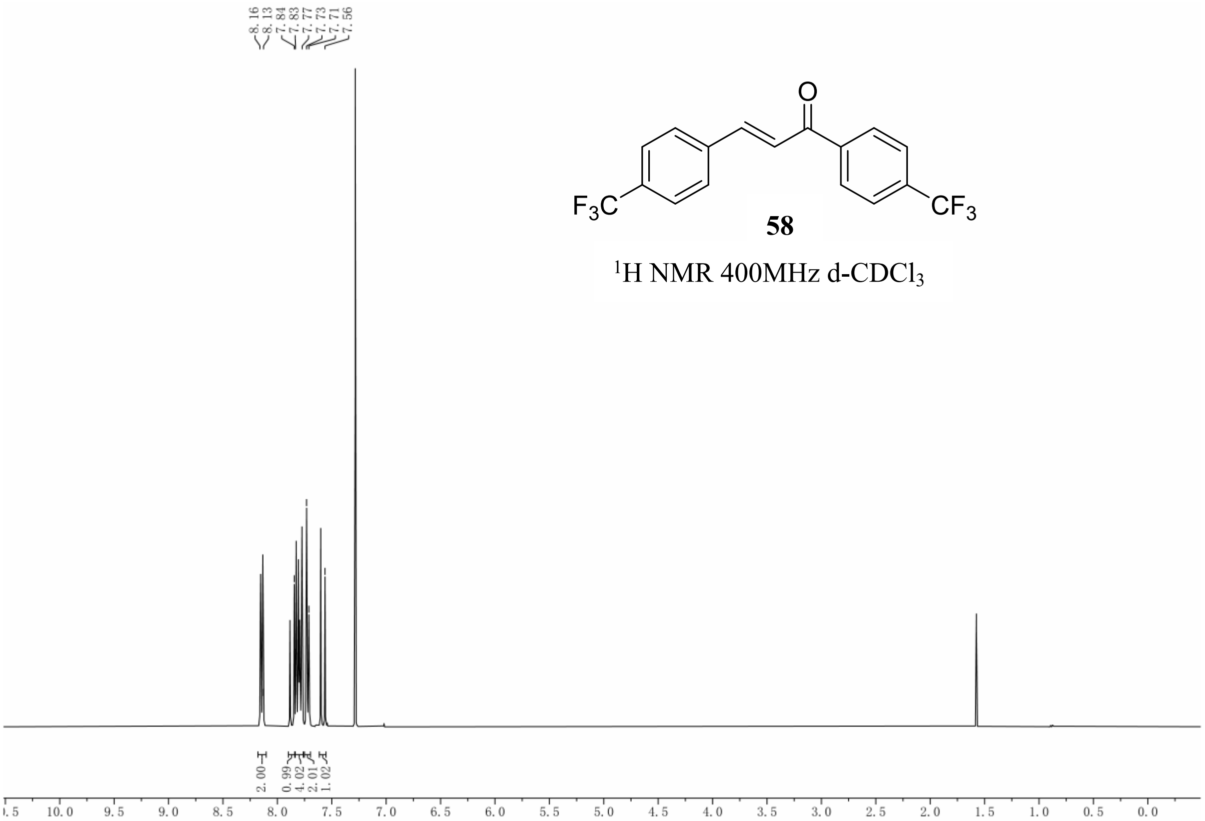
8.16
8.13
7.84
7.83
7.77
7.73
7.71
7.56
O
F₃C
CF₃
58
¹H NMR 400MHz d-CDCl₃
2.00
0.99
4.02
2.01
1.02
10.0
9.5
9.0
8.5
8.0
7.5
7.0
6.5
6.0
5.5
5.0
4.5
4.0
3.5
3.0
2.5
2.0
1.5
1.0
0.5
0.0
¹H (ppm)

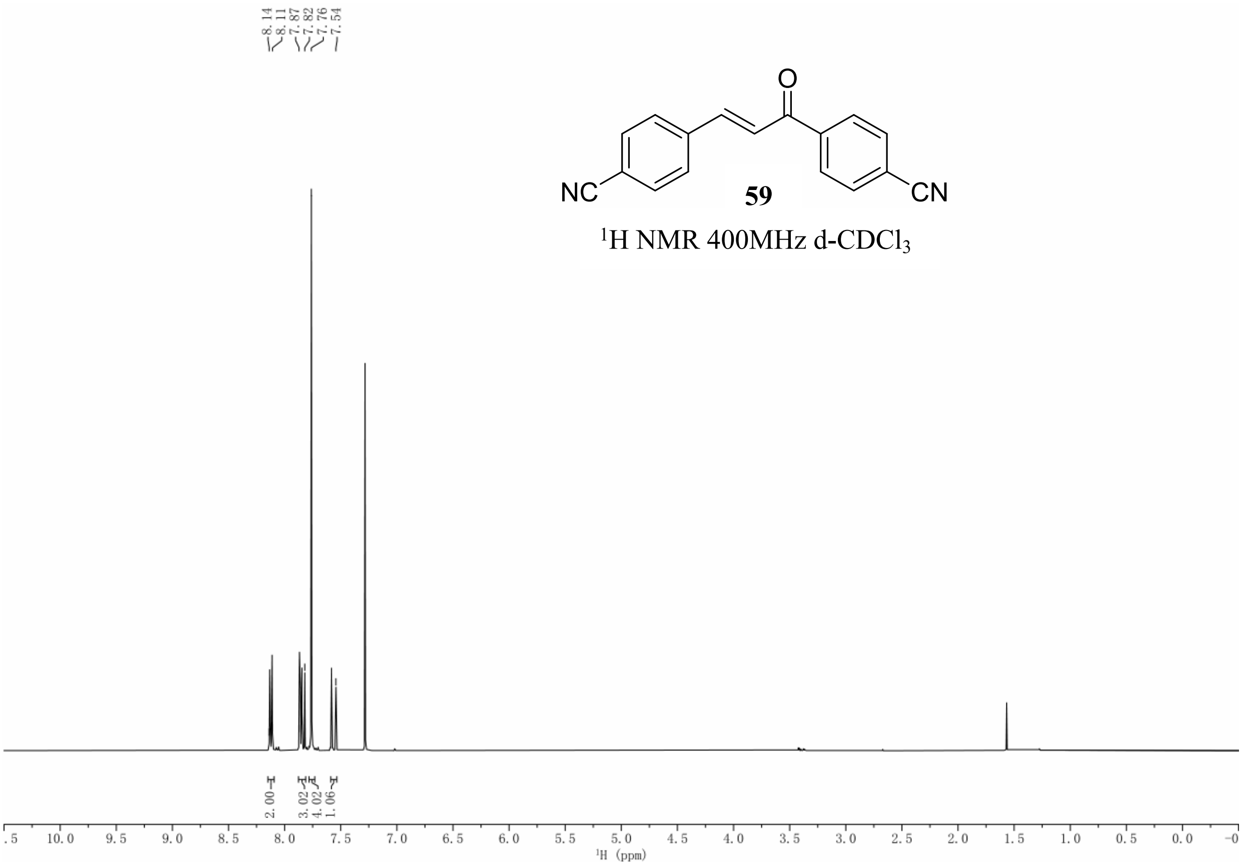
8.14
8.11
7.87
7.82
7.76
7.54
O
NC
CN
59
^{1}H NMR 400MHz d-$CDCl_3$
2.00
3.02
4.02
1.06
.5 10.0 9.5 9.0 8.5 8.0 7.5 7.0 6.5 6.0 5.5 5.0 4.5 4.0 3.5 3.0 2.5 2.0 1.5 1.0 0.5 0.0 -0
^{1}H (ppm)

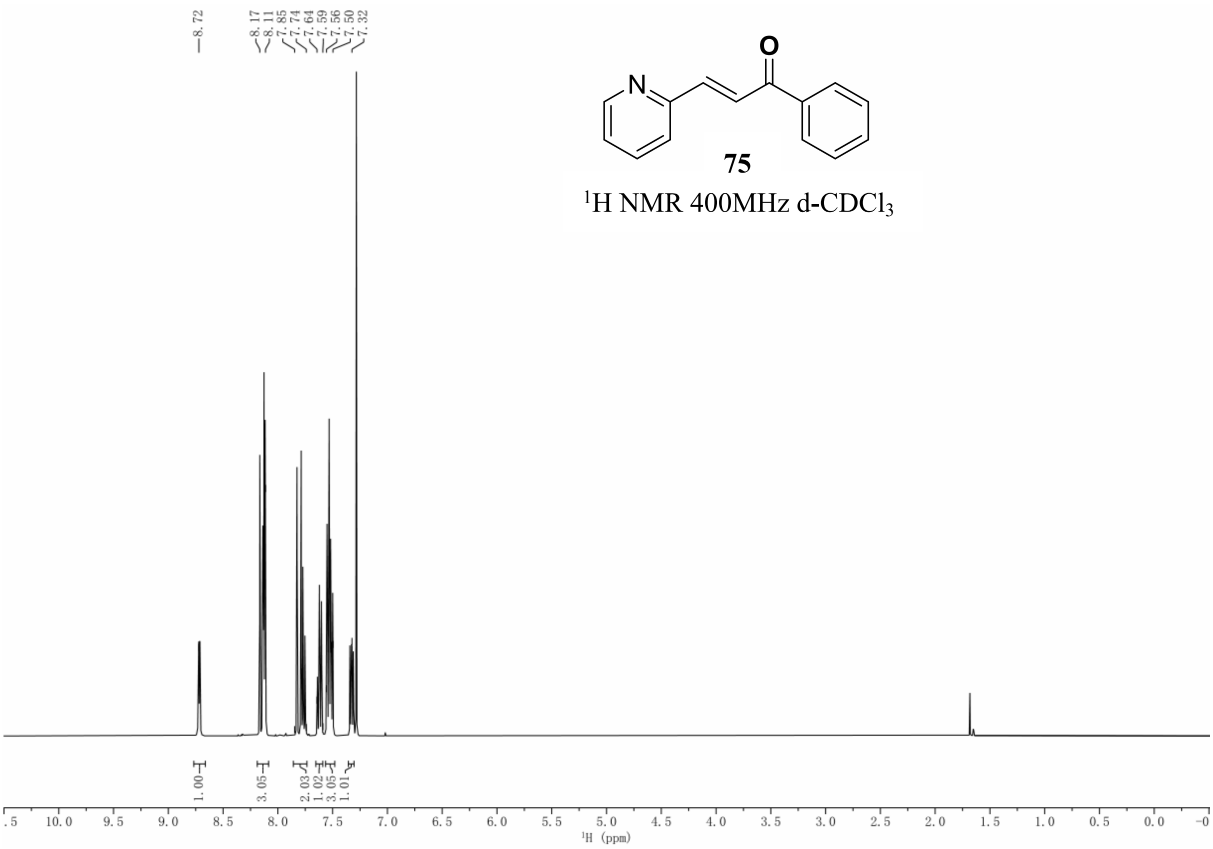
8.72
8.17
8.11
7.85
7.74
7.64
7.59
7.56
7.50
7.32
O
N
75
^{1}H NMR 400MHz d-$CDCl_3$
1.00
3.05
2.03
1.02
3.05
1.01
.5 10.0 9.5 9.0 8.5 8.0 7.5 7.0 6.5 6.0 5.5 5.0 4.5 4.0 3.5 3.0 2.5 2.0 1.5 1.0 0.5 0.0 -0
^{1}H (ppm)

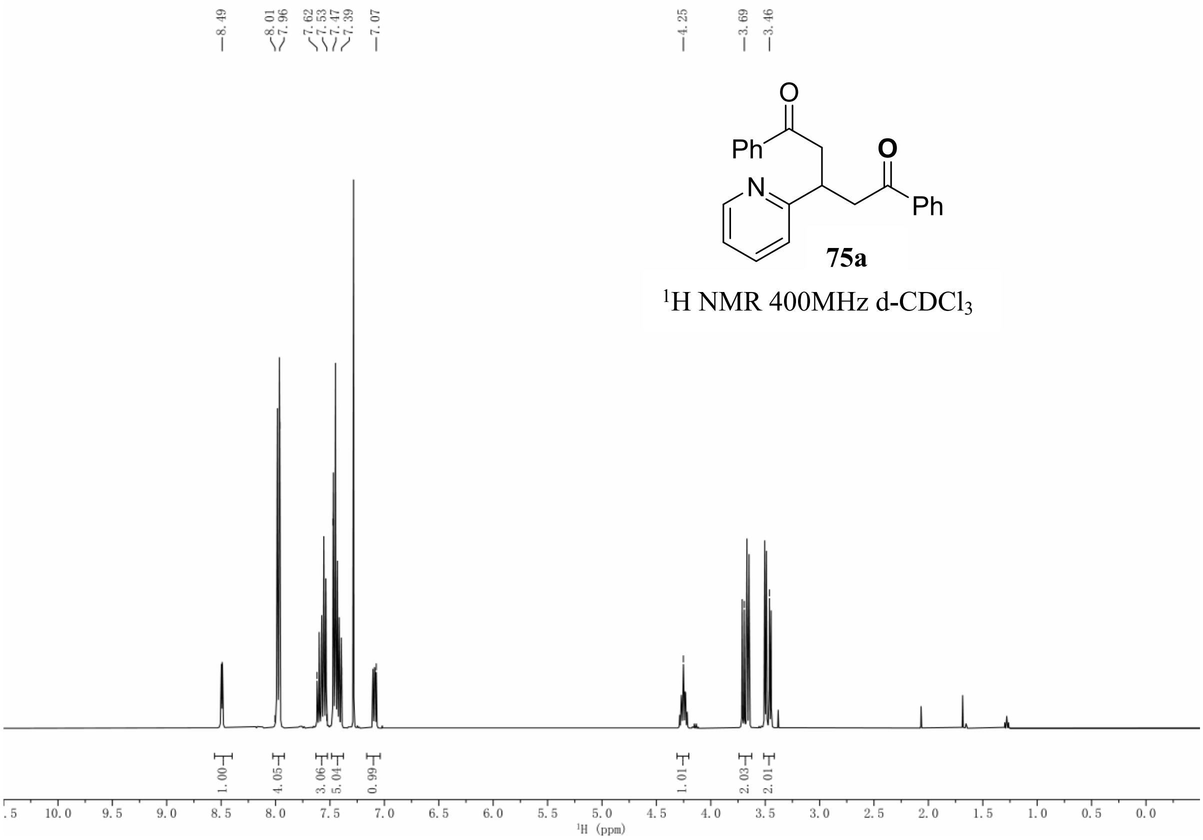
8.49
8.01
7.96
7.62
7.53
7.47
7.39
7.07
4.25
3.69
3.46
Ph
O
N
O
Ph
75a
1H NMR 400MHz d-CDCl3
1.00
4.05
3.06
5.04
0.99
1.01
2.03
2.01
1H (ppm)

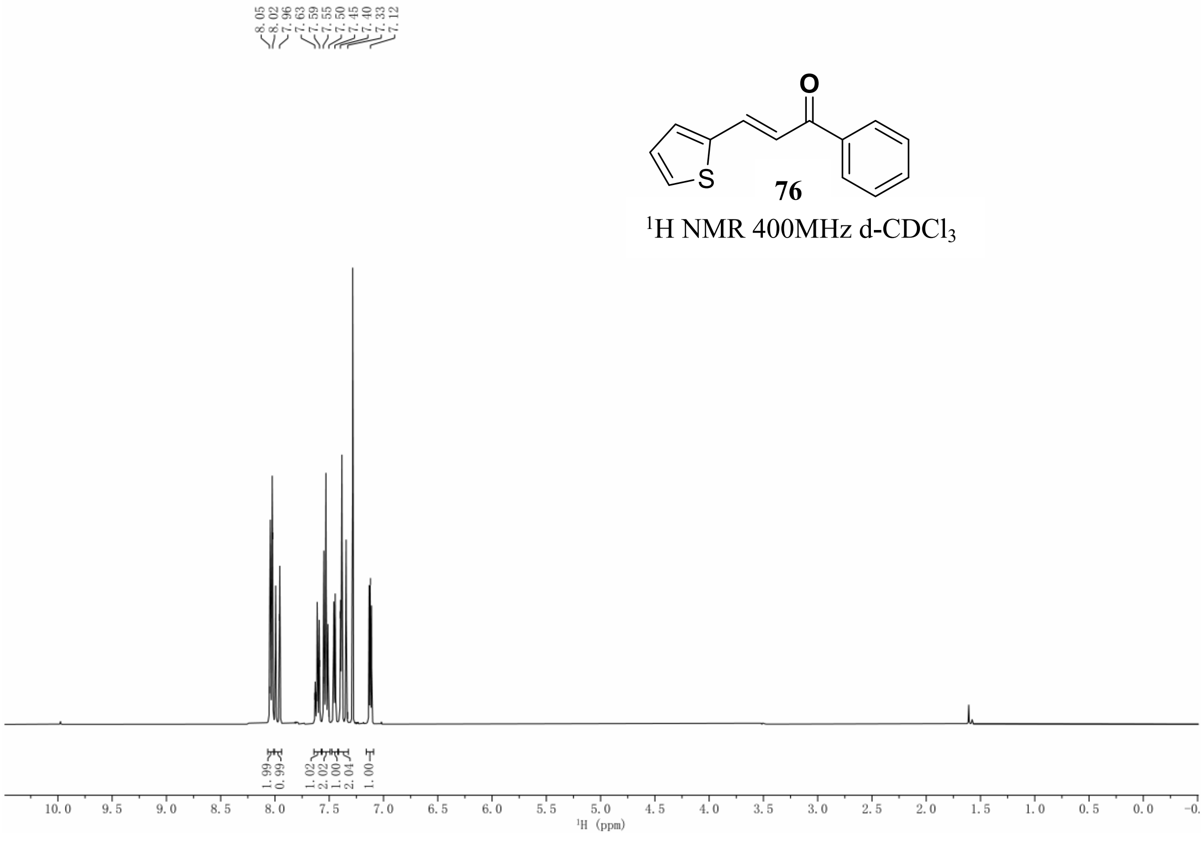
8.05
8.02
7.96
7.63
7.59
7.55
7.50
7.45
7.40
7.33
7.12
O
S
76
1H NMR 400MHz d-CDCl3
1.99
0.99
1.02
2.02
1.00
2.04
1.00
1H (ppm)

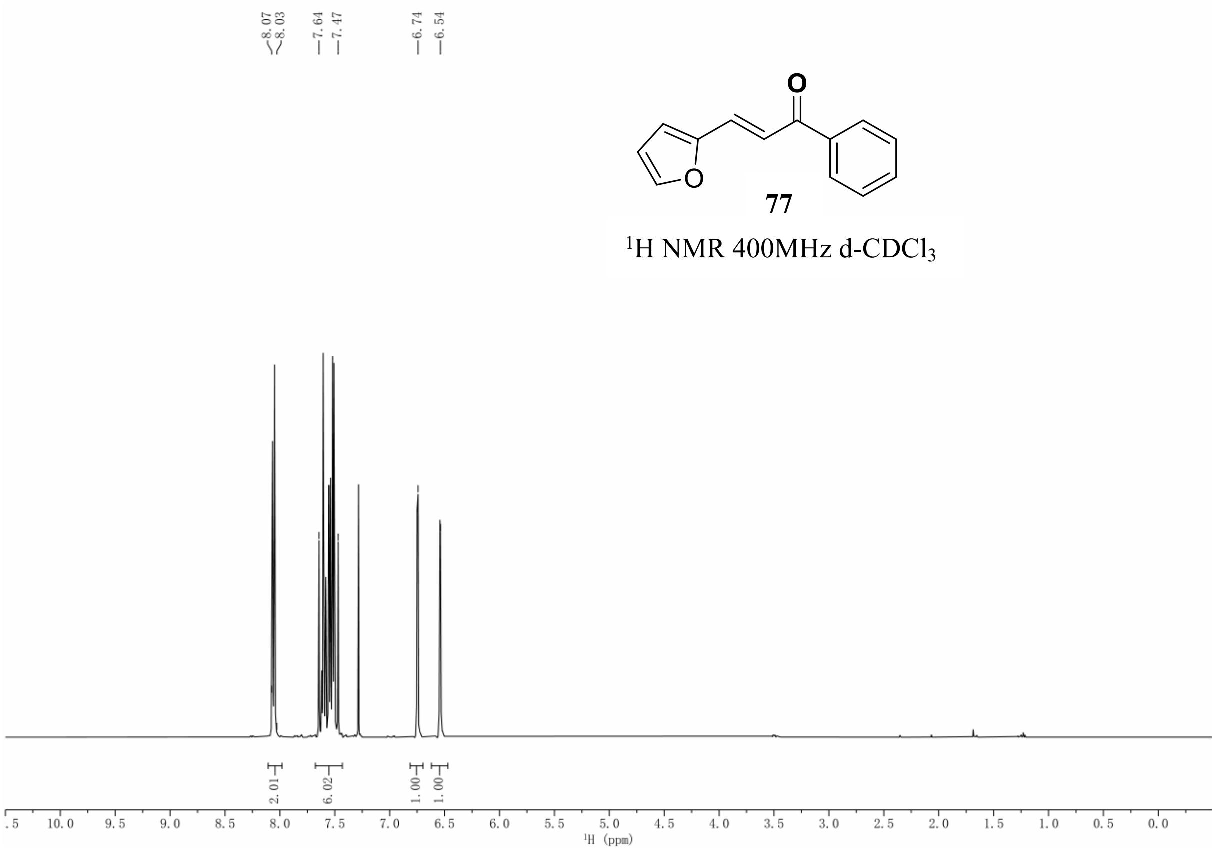
8.07
8.03
7.64
7.47
6.74
6.54
O
O
77
^{1}H NMR 400MHz d-CDCl$_3$
2.01
6.02
1.00
1.00
^{1}H (ppm)

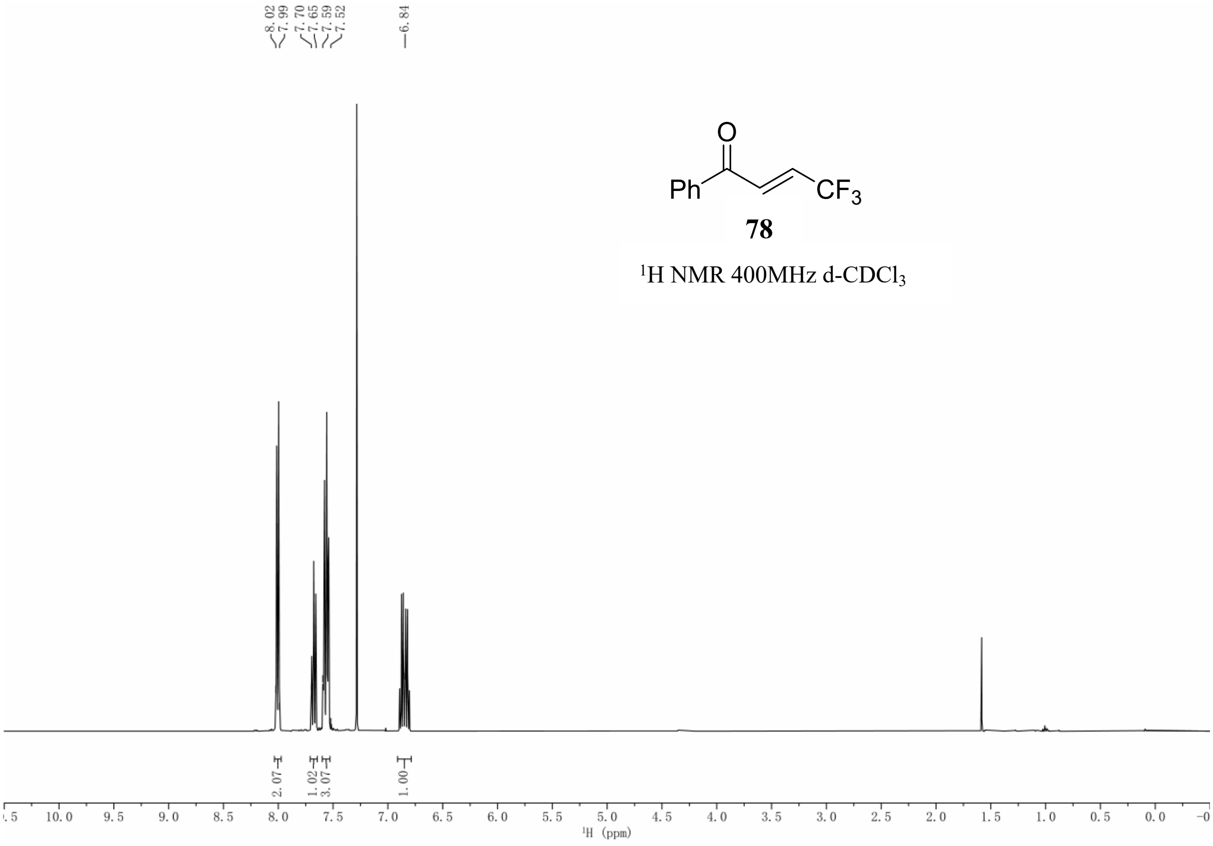
8.02
7.99
7.70
7.65
7.59
7.52
6.84
O
Ph
CF$_3$
78
^{1}H NMR 400MHz d-CDCl$_3$
2.07
1.02
3.07
1.00
^{1}H (ppm)

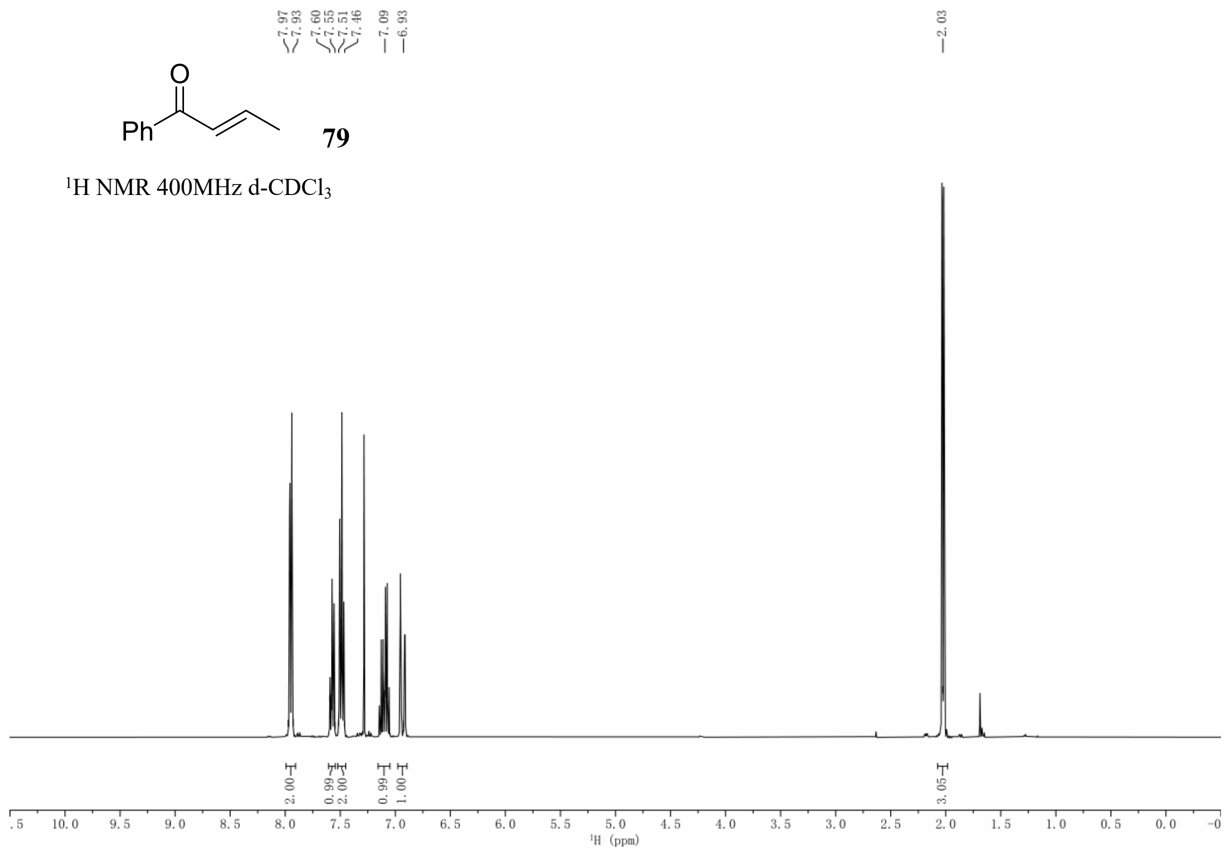
7.97
7.93
7.60
7.55
7.51
7.46
7.09
6.93
2.03
O
Ph
79
¹H NMR 400MHz d-CDCl₃
2.00
0.99
2.00
0.99
1.00
3.05
¹H (ppm)

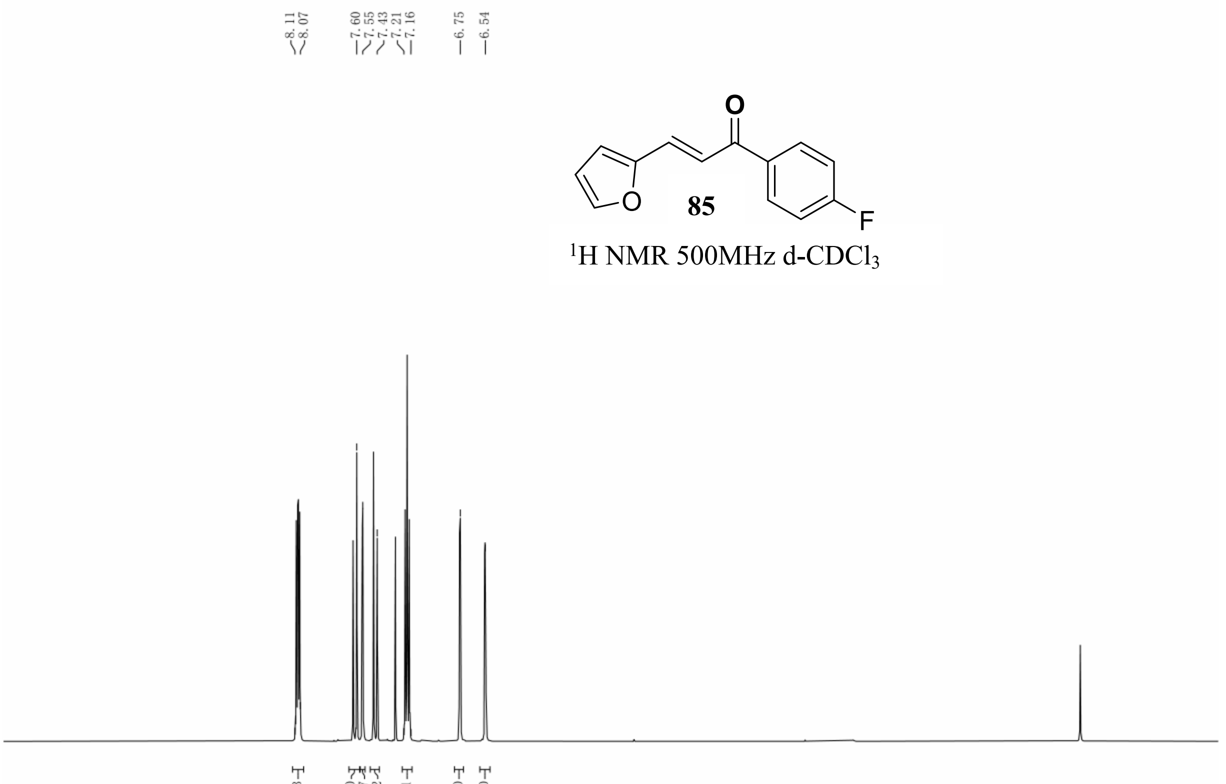
8.11
8.07
7.60
7.55
7.43
7.21
7.16
6.75
6.54
O
O
85
F
¹H NMR 500MHz d-CDCl₃
2.03
1.00
0.97
1.02
2.01
1.00
1.00
¹H (ppm)

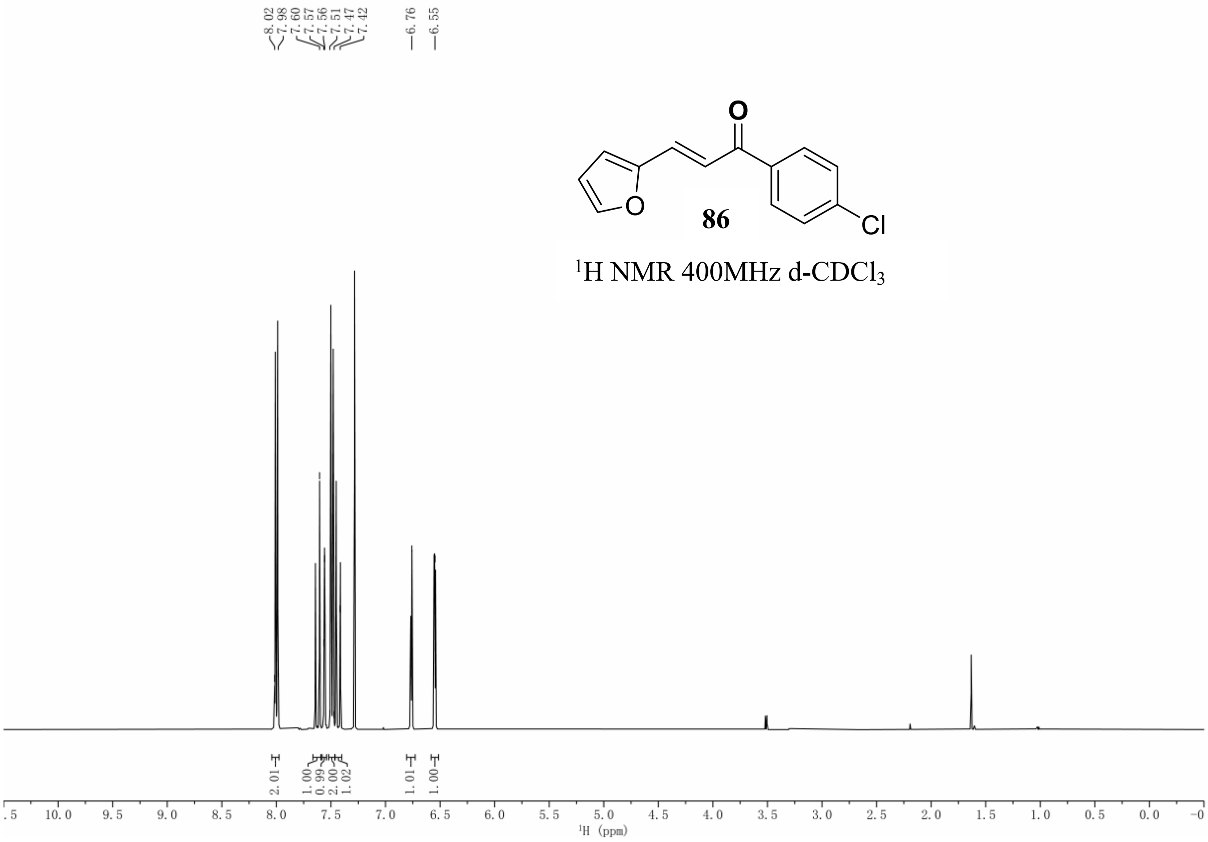
8.02
7.98
7.60
7.57
7.56
7.51
7.47
7.42
6.76
6.55
O
O
86
Cl
1H NMR 400MHz d-CDCl3
2.01
1.00
0.99
2.00
1.02
1.01
1.00
1H (ppm)

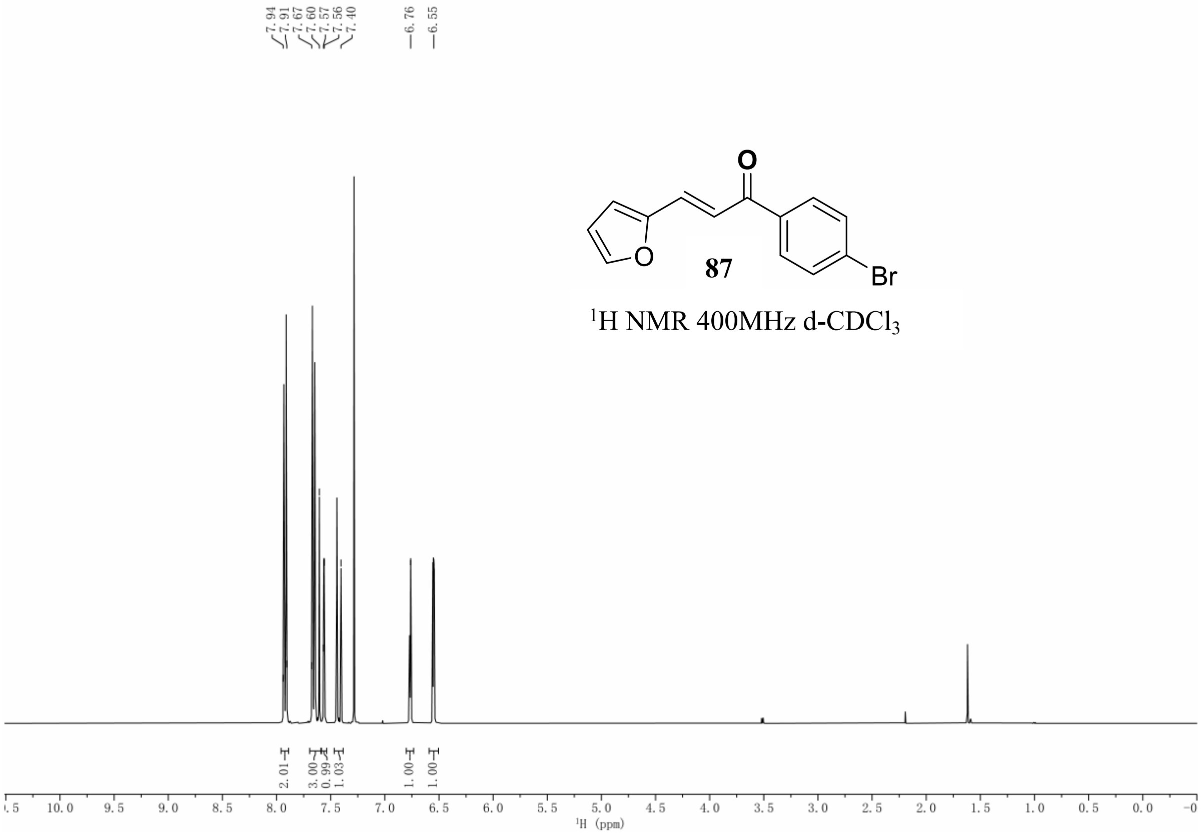
7.94
7.91
7.67
7.60
7.57
7.56
7.40
6.76
6.55
O
O
87
Br
1H NMR 400MHz d-CDCl3
2.01
3.00
0.99
1.03
1.00
1.00
1H (ppm)

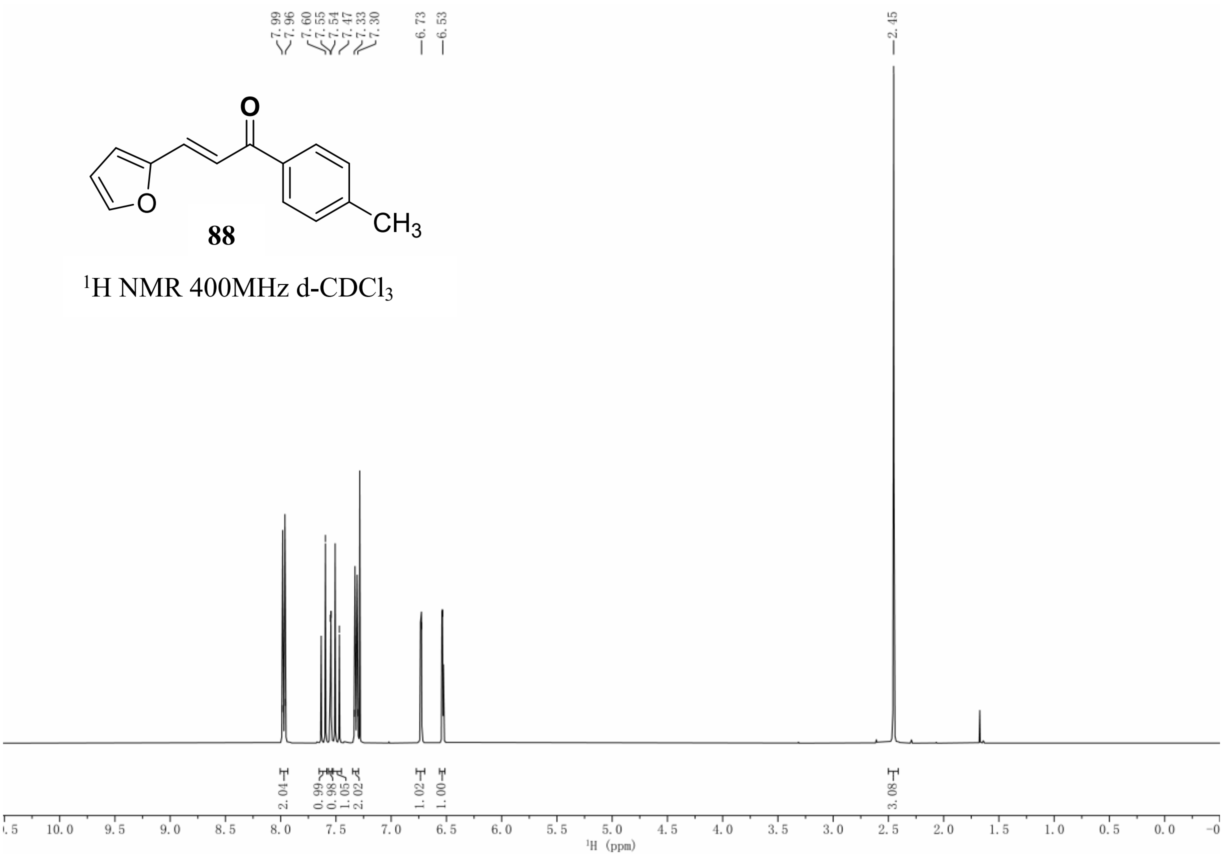
O
CH3
88
1H NMR 400MHz d-CDCl3
7.99
7.96
7.60
7.55
7.54
7.47
7.33
7.30
6.73
6.53
2.45
2.04
0.99
0.98
1.05
2.02
1.02
1.00
3.08
1H (ppm)

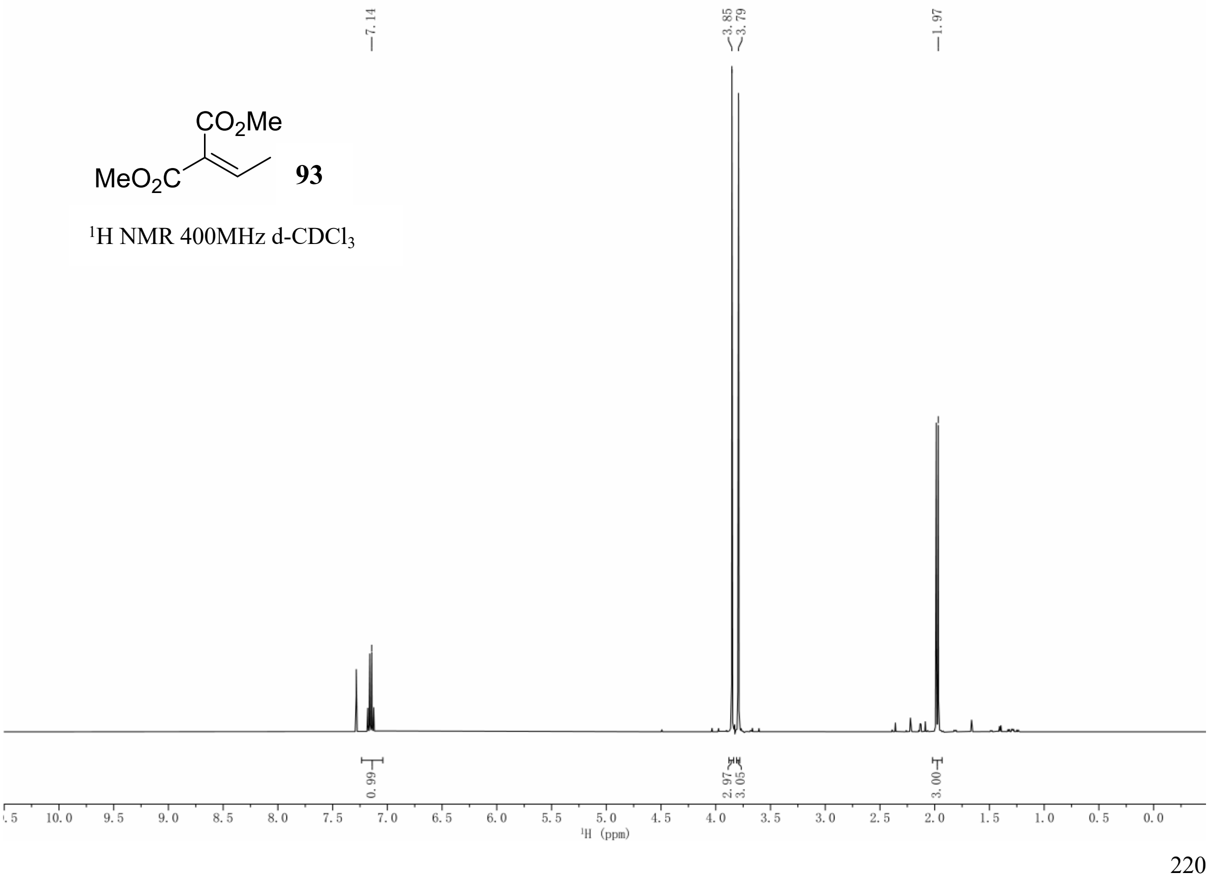
CO2Me
MeO2C
93
1H NMR 400MHz d-CDCl3
7.14
3.85
3.79
1.97
0.99
2.97
3.05
3.00
1H (ppm)

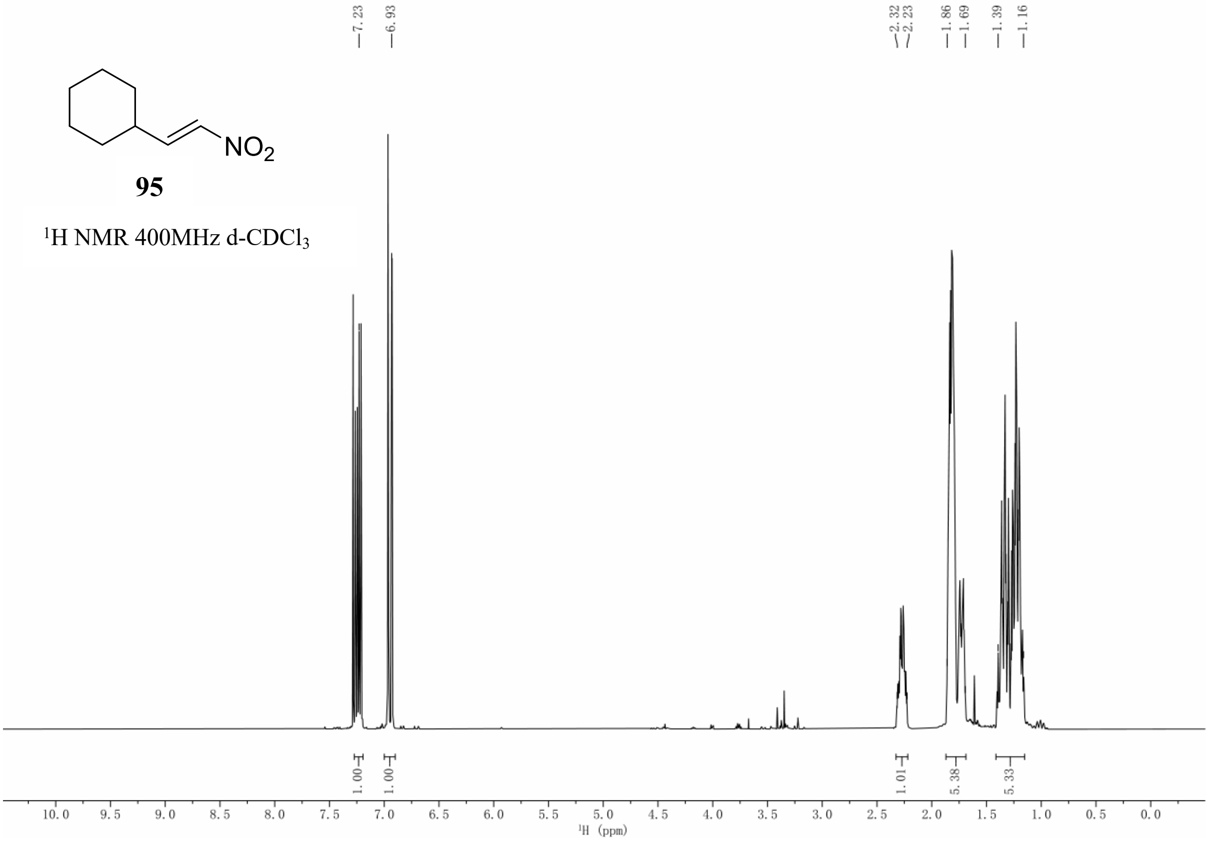
7.23
6.93
2.32
2.23
1.86
1.69
1.39
1.16
NO2
95
1H NMR 400MHz d-CDCl3
1.00
1.00
1.01
5.38
5.33
10.0 9.5 9.0 8.5 8.0 7.5 7.0 6.5 6.0 5.5 5.0 4.5 4.0 3.5 3.0 2.5 2.0 1.5 1.0 0.5 0.0
1H (ppm)

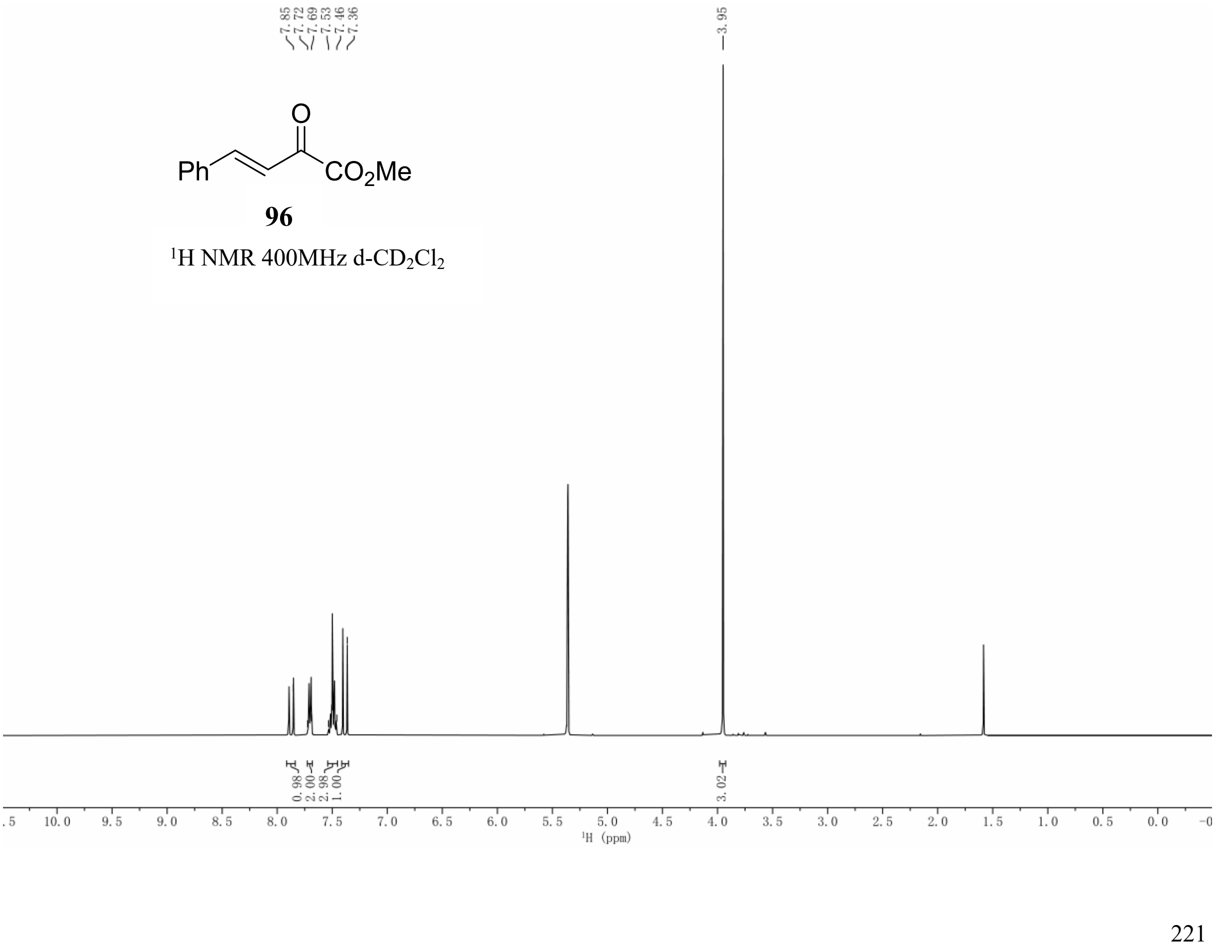
7.85
7.72
7.69
7.53
7.46
7.36
3.95
O
Ph
CO2Me
96
1H NMR 400MHz d-CD2Cl2
0.98
2.00
2.98
1.00
3.02
10.0 9.5 9.0 8.5 8.0 7.5 7.0 6.5 6.0 5.5 5.0 4.5 4.0 3.5 3.0 2.5 2.0 1.5 1.0 0.5 0.0
1H (ppm)

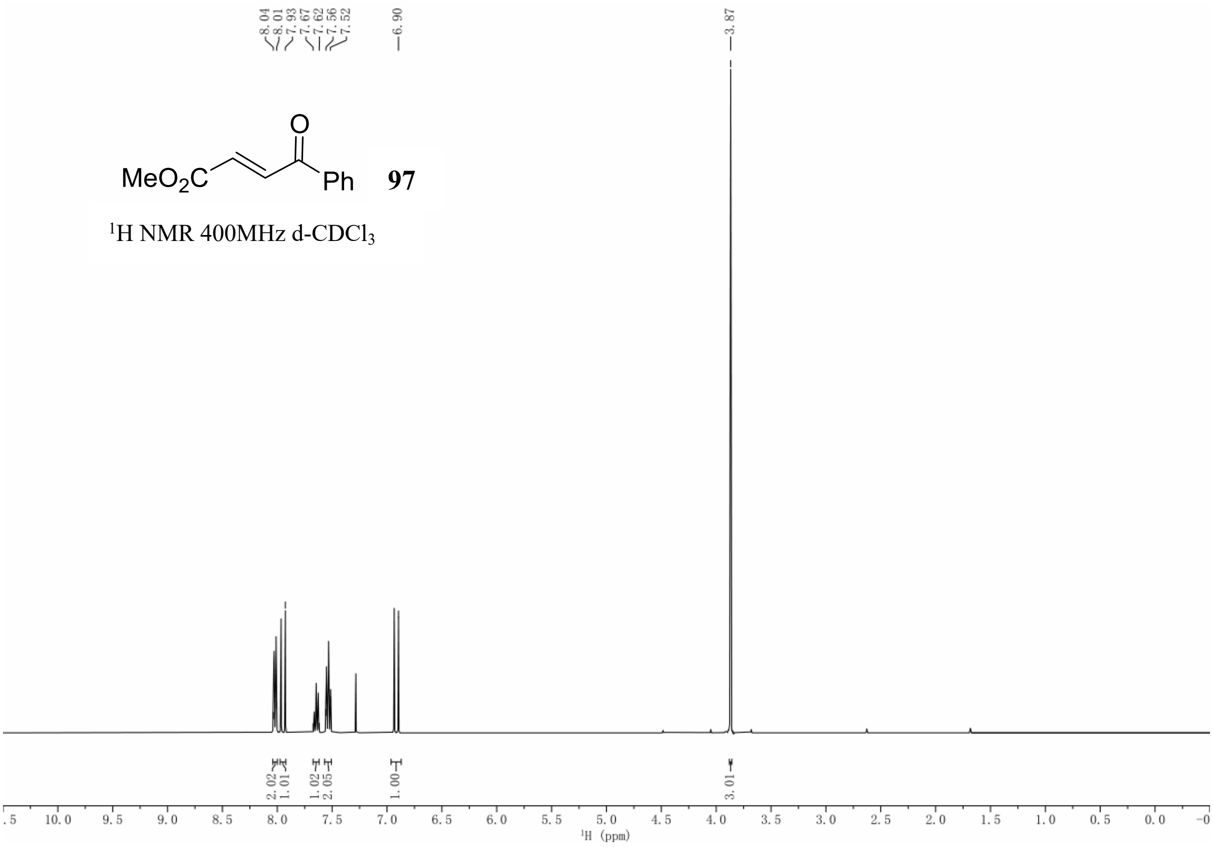
8.04
8.01
7.93
7.67
7.62
7.56
7.52
6.90
3.87
MeO2C
O
Ph
97
1H NMR 400MHz d-CDCl3
2.02
1.01
1.02
2.05
1.00
3.01
10.0 9.5 9.0 8.5 8.0 7.5 7.0 6.5 6.0 5.5 5.0 4.5 4.0 3.5 3.0 2.5 2.0 1.5 1.0 0.5 0.0
1H (ppm)

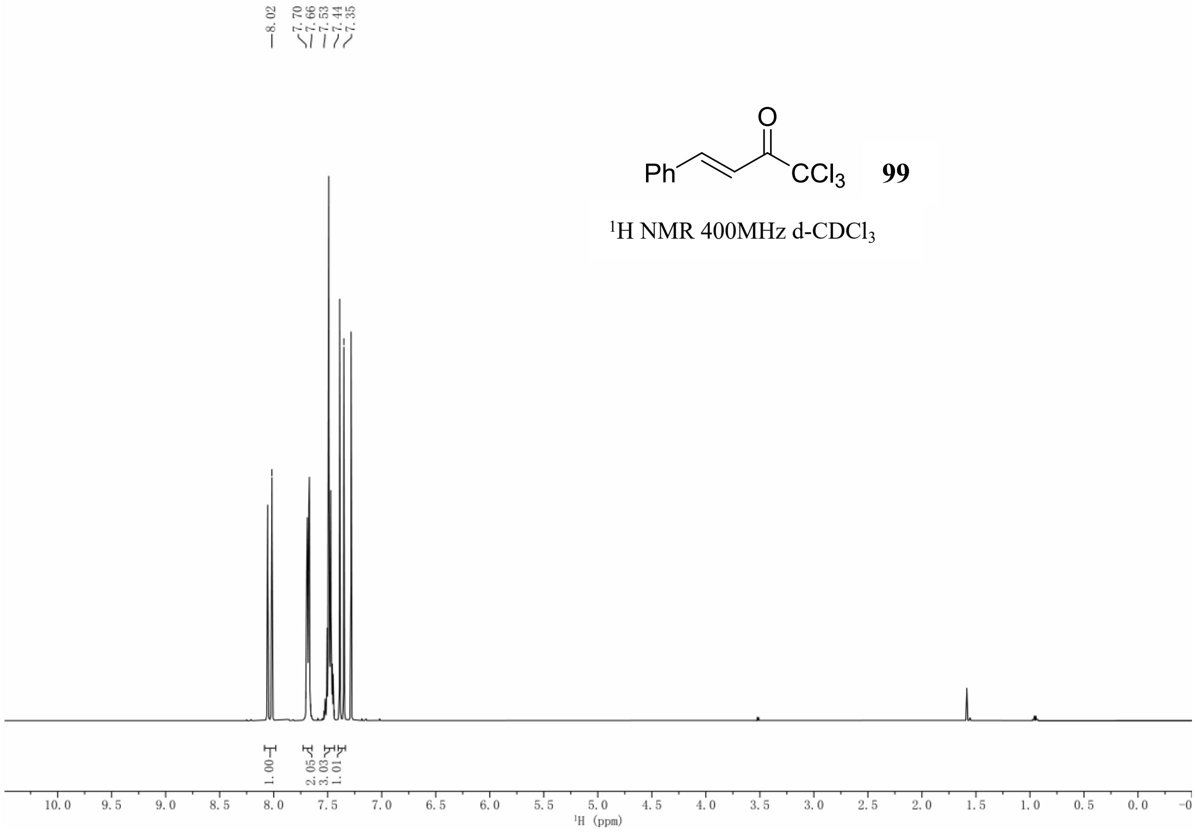
8.02
7.70
7.66
7.53
7.44
7.35
O
Ph
CCl3
99
1H NMR 400MHz d-CDCl3
1.00
2.05
3.03
1.01
10.0 9.5 9.0 8.5 8.0 7.5 7.0 6.5 6.0 5.5 5.0 4.5 4.0 3.5 3.0 2.5 2.0 1.5 1.0 0.5 0.0
1H (ppm)

2. Stetter Adducts

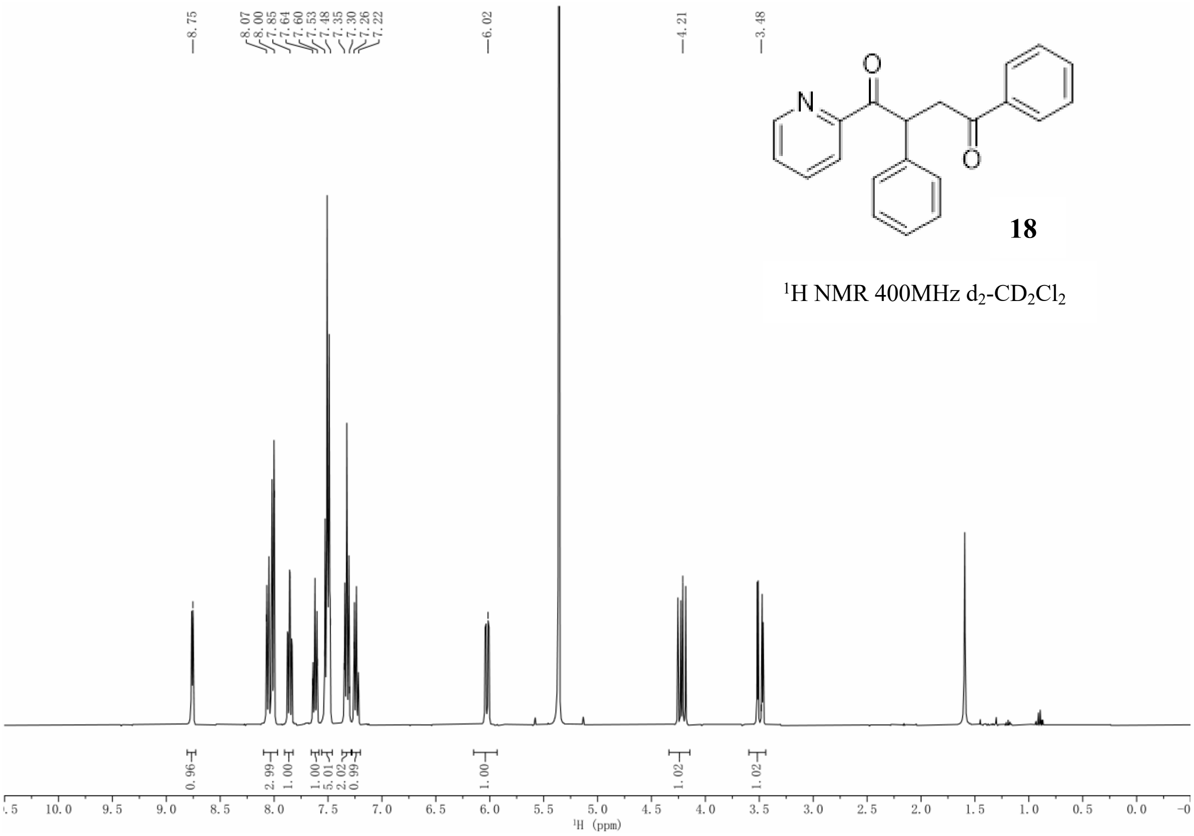

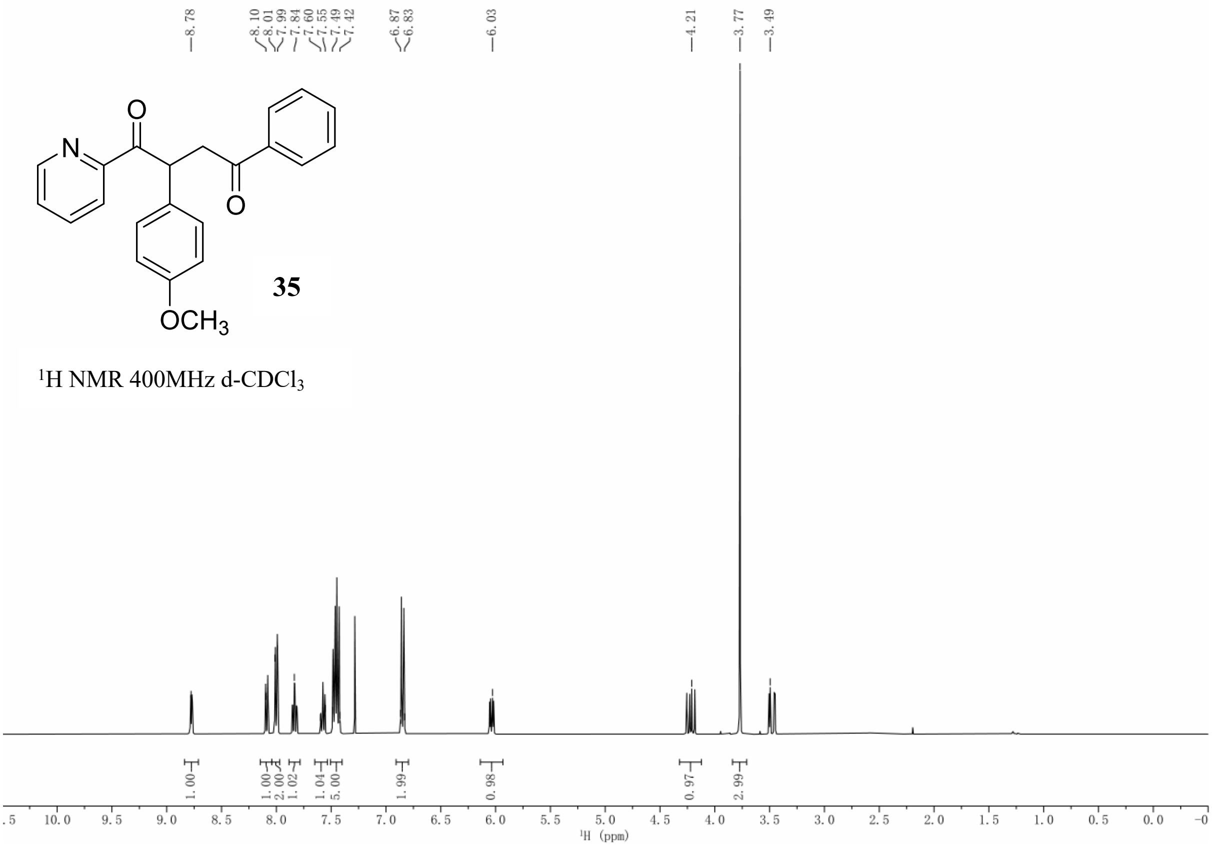

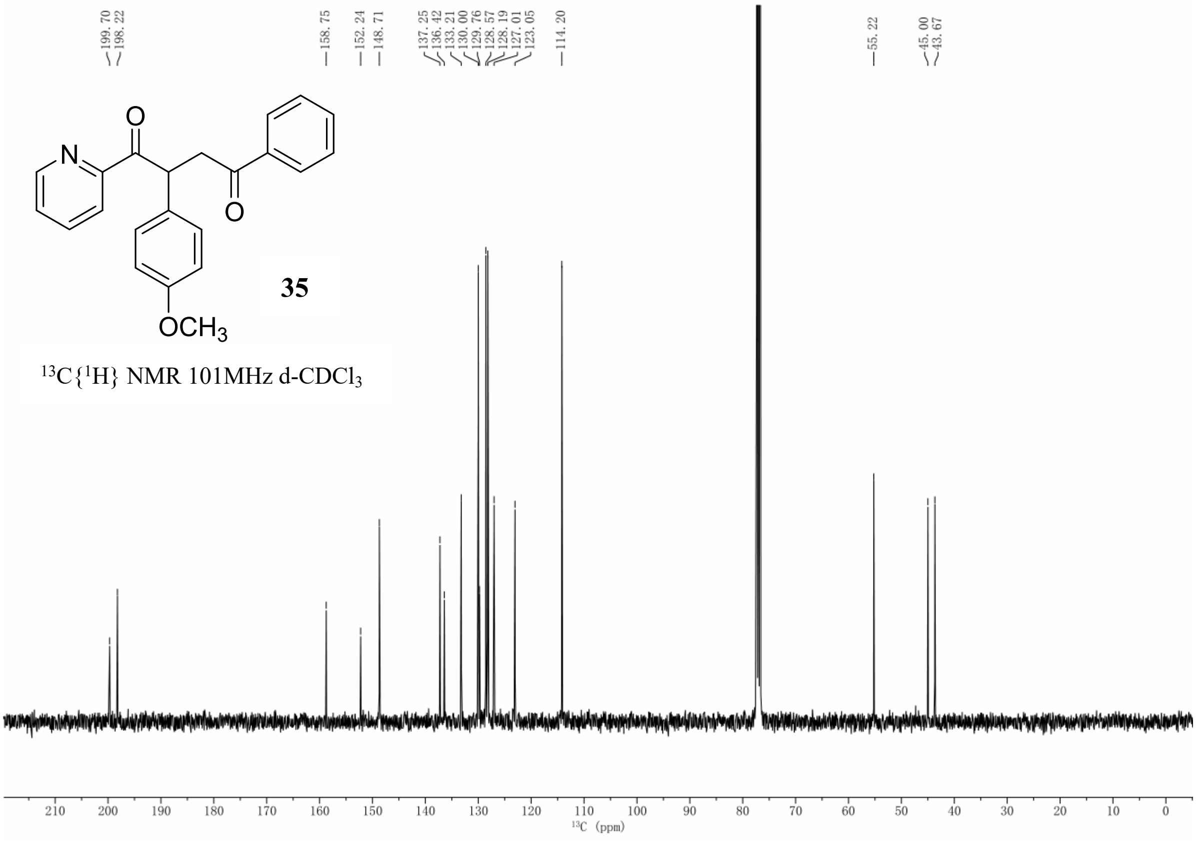

199.70
198.22
158.75
152.24
148.71
137.25
136.42
133.21
130.00
129.76
128.57
128.19
127.01
123.05
114.20
55.22
45.00
43.67
35
$^{13}C\{^{1}H\}$ NMR 101MHz d-CDCl$_3$
210 200 190 180 170 160 150 140 130 120 110 100 90 80 70 60 50 40 30 20 10 0
^{13}C (ppm)

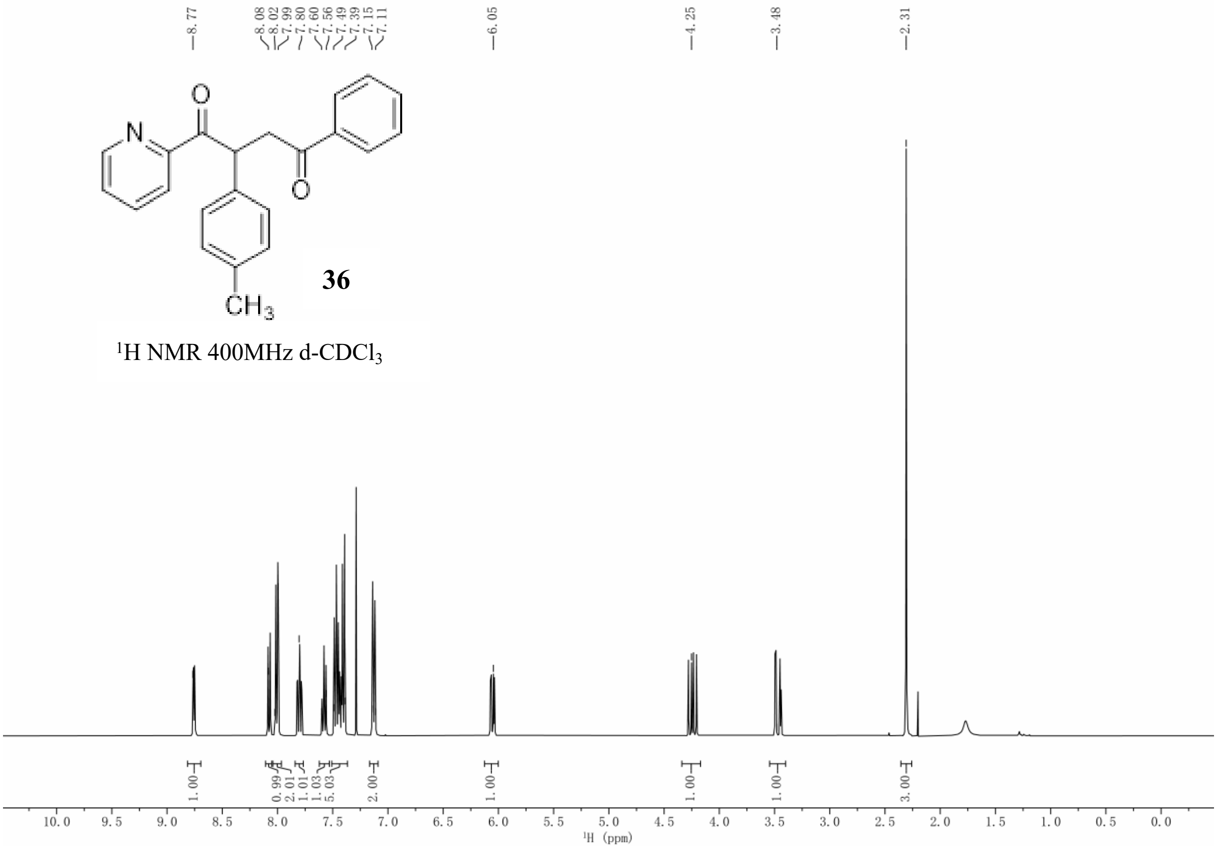

8.77
8.08
8.02
7.99
7.80
7.60
7.56
7.49
7.39
7.15
7.11
6.05
4.25
3.48
2.31
36
^{1}H NMR 400MHz d-CDCl$_3$
1.00
0.99
2.01
1.01
1.03
5.03
2.00
1.00
1.00
1.00
3.00
10.0 9.5 9.0 8.5 8.0 7.5 7.0 6.5 6.0 5.5 5.0 4.5 4.0 3.5 3.0 2.5 2.0 1.5 1.0 0.5 0.0
^{1}H (ppm)

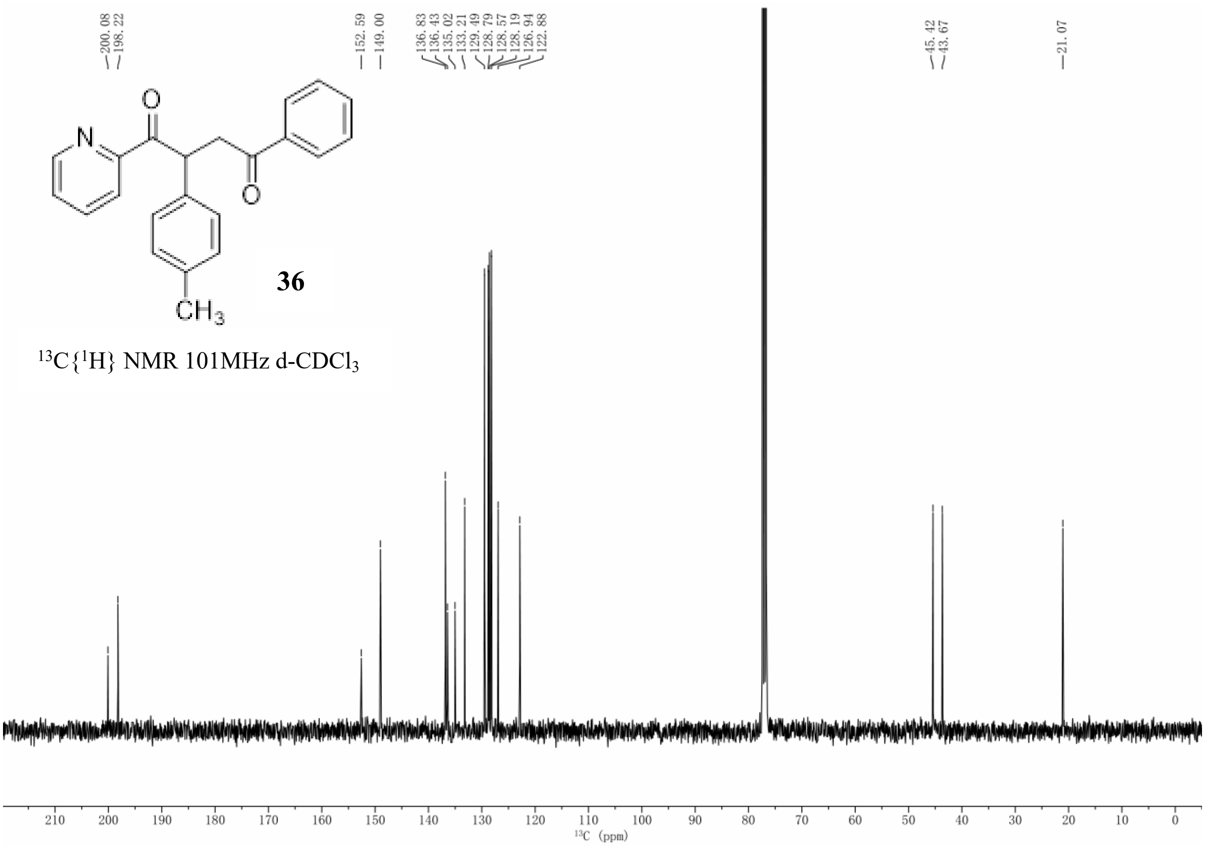
200.08
198.22
152.59
149.00
136.83
136.43
135.02
133.21
129.49
128.79
128.57
128.19
126.94
122.88
45.42
43.67
21.07
36
$^{13}C\{^{1}H\}$ NMR 101MHz d-$CDCl_3$
^{13}C (ppm)

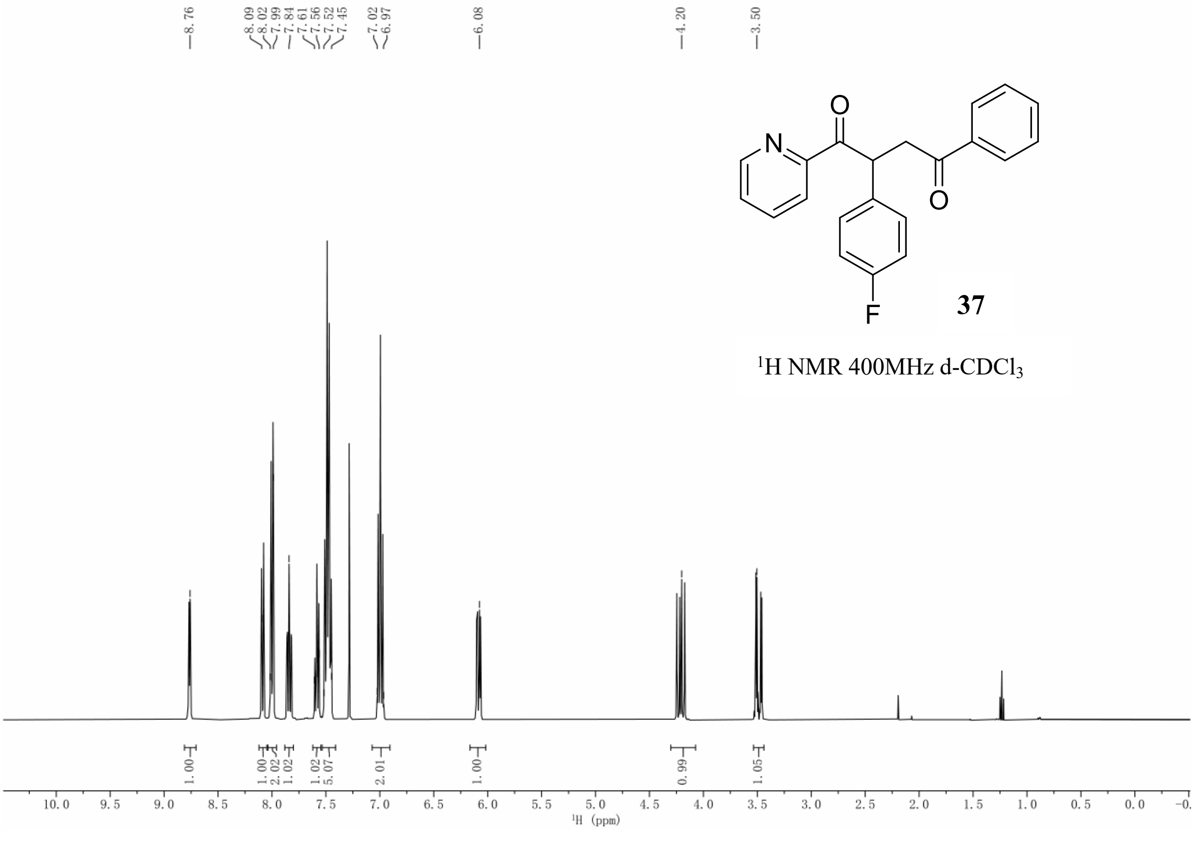
8.76
8.09
8.02
7.99
7.84
7.61
7.56
7.52
7.45
7.02
6.97
6.08
4.20
3.50
37
^{1}H NMR 400MHz d-$CDCl_3$
1.00
1.00
2.02
1.02
1.02
5.07
2.01
1.00
0.99
1.05
^{1}H (ppm)

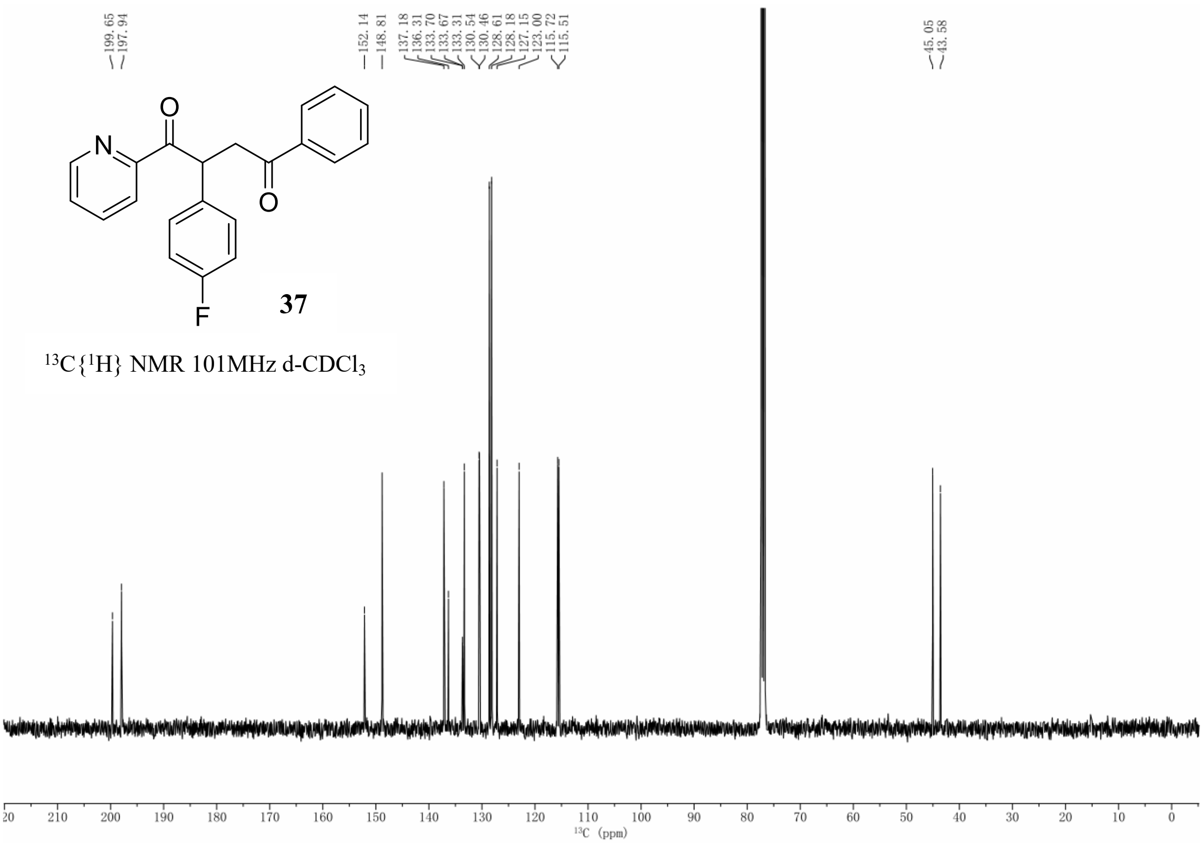
199.65
197.94
152.14
148.81
137.18
136.31
133.70
133.67
133.31
130.54
130.46
128.61
128.18
127.15
123.00
115.72
115.51
45.05
43.58
37
$^{13}C\{^{1}H\}$ NMR 101MHz d-$CDCl_3$
^{13}C (ppm)

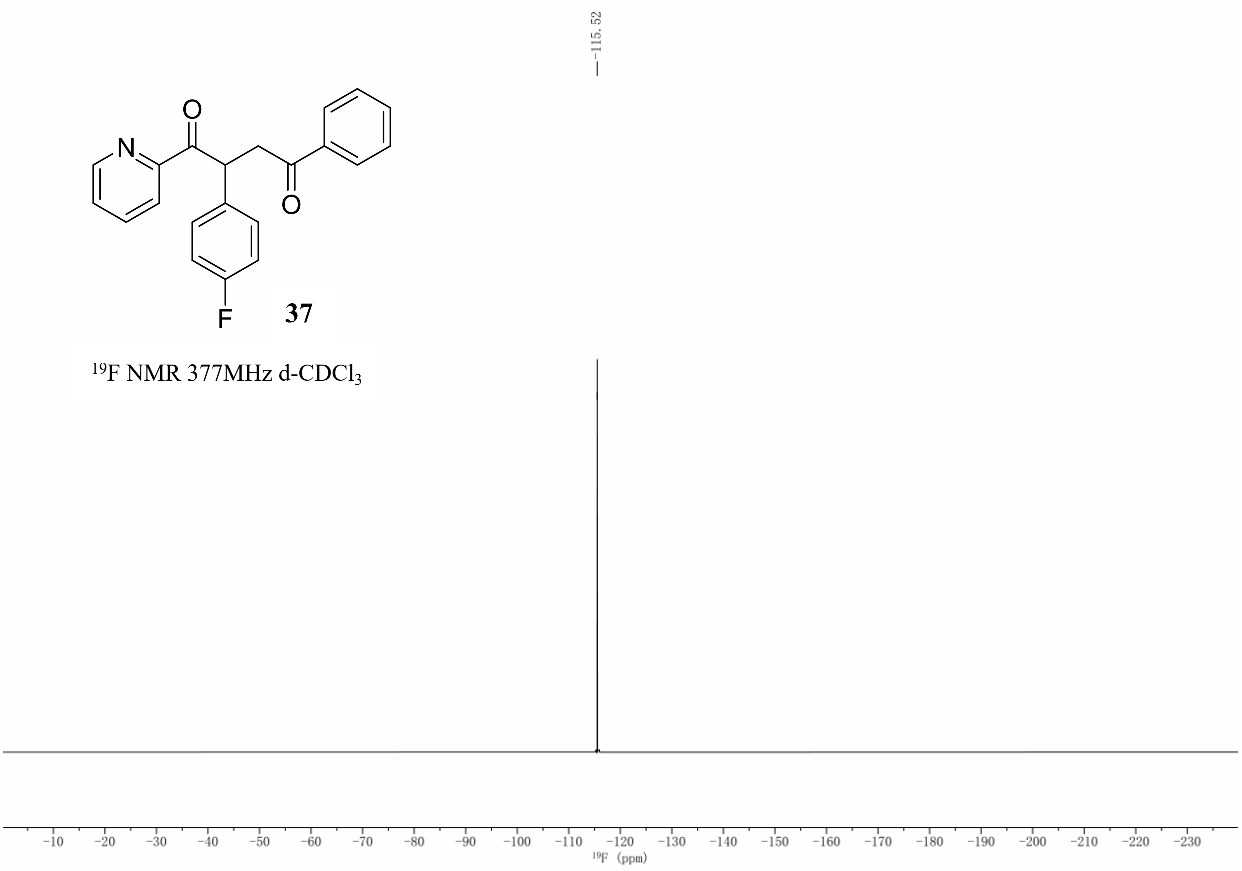
−115.52
37
^{19}F NMR 377MHz d-$CDCl_3$
^{19}F (ppm)

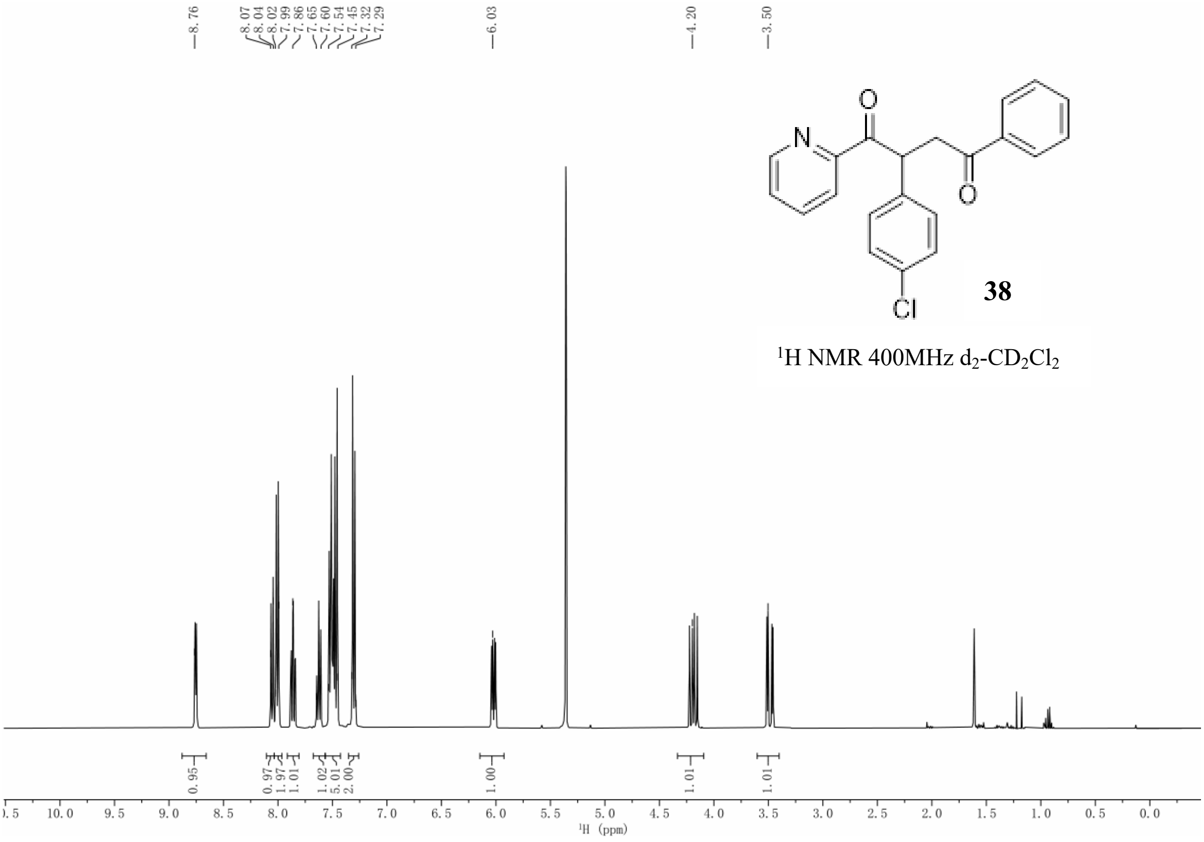
8.76
8.07
8.04
8.02
7.99
7.86
7.65
7.60
7.54
7.45
7.32
7.29
6.03
4.20
3.50
38
¹H NMR 400MHz d₂-CD₂Cl₂
0.95
0.97
1.97
1.01
1.02
5.01
2.00
1.00
1.01
1.01
¹H (ppm)

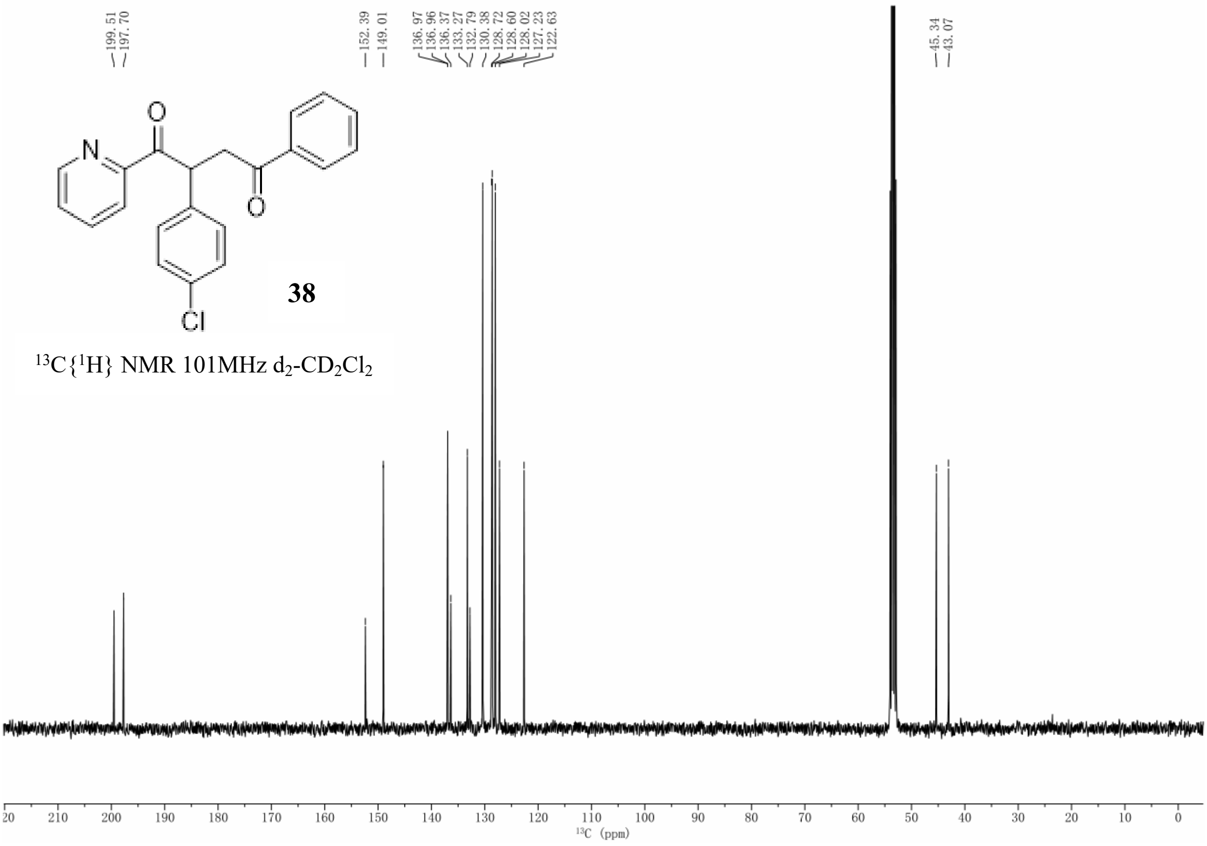
199.51
197.70
152.39
149.01
136.97
136.96
136.37
133.27
132.79
130.38
128.72
128.60
128.02
127.23
122.63
45.34
43.07
38
¹³C{¹H} NMR 101MHz d₂-CD₂Cl₂
¹³C (ppm)

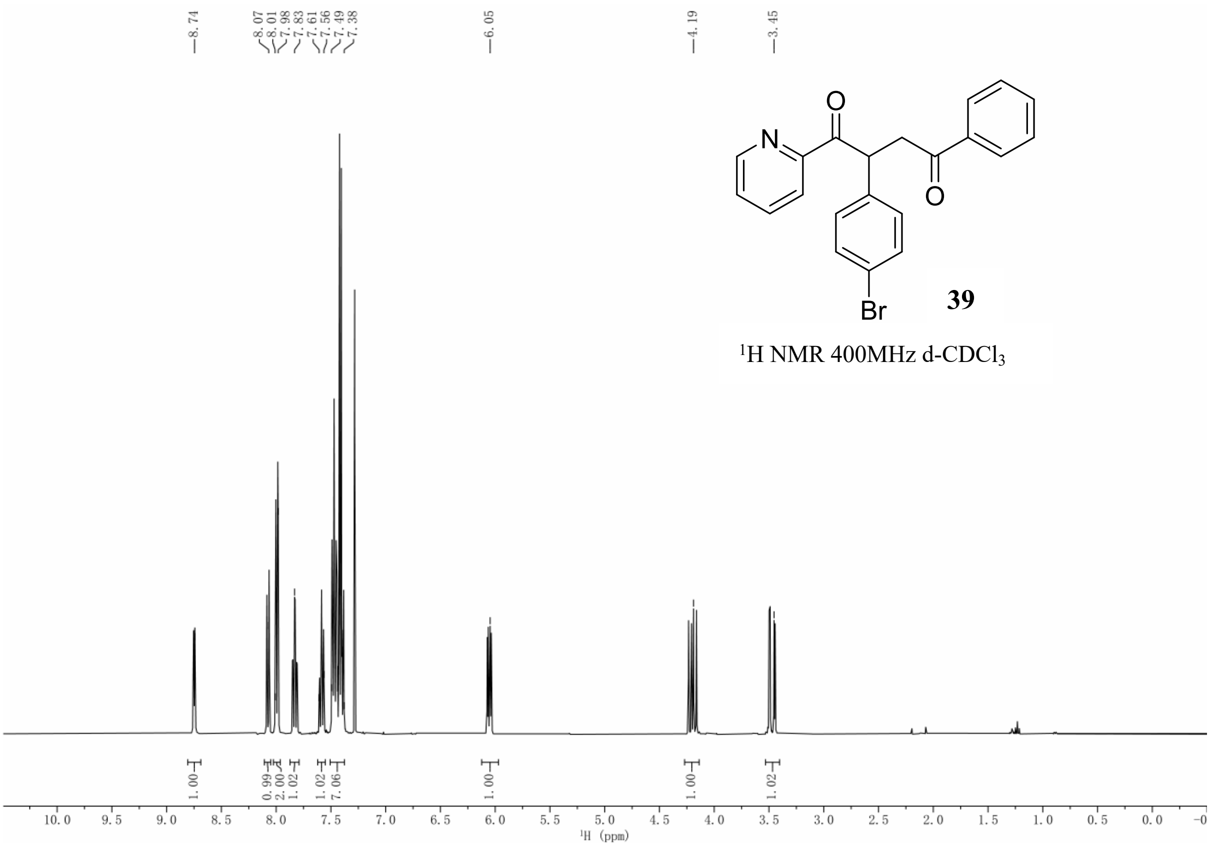
8.74
8.07
8.01
7.98
7.83
7.61
7.56
7.49
7.38
6.05
4.19
3.45
O
N
O
Br
39
¹H NMR 400MHz d-CDCl₃
1.00
0.99
2.00
1.02
1.02
7.06
1.00
1.00
1.02
¹H (ppm)

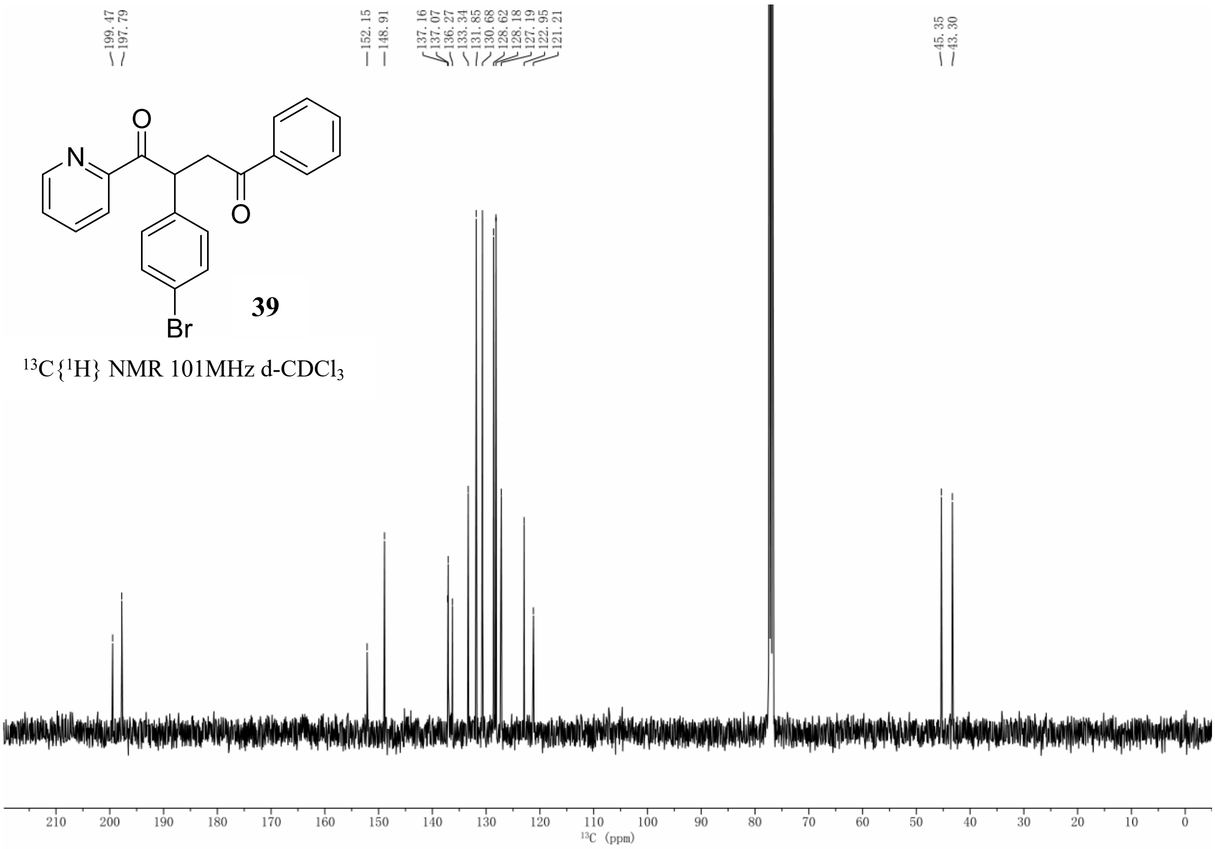
199.47
197.79
152.15
148.91
137.16
137.07
136.27
133.34
131.85
130.68
128.62
128.18
127.19
122.95
121.21
45.35
43.30
O
N
O
Br
39
¹³C{¹H} NMR 101MHz d-CDCl₃
¹³C (ppm)

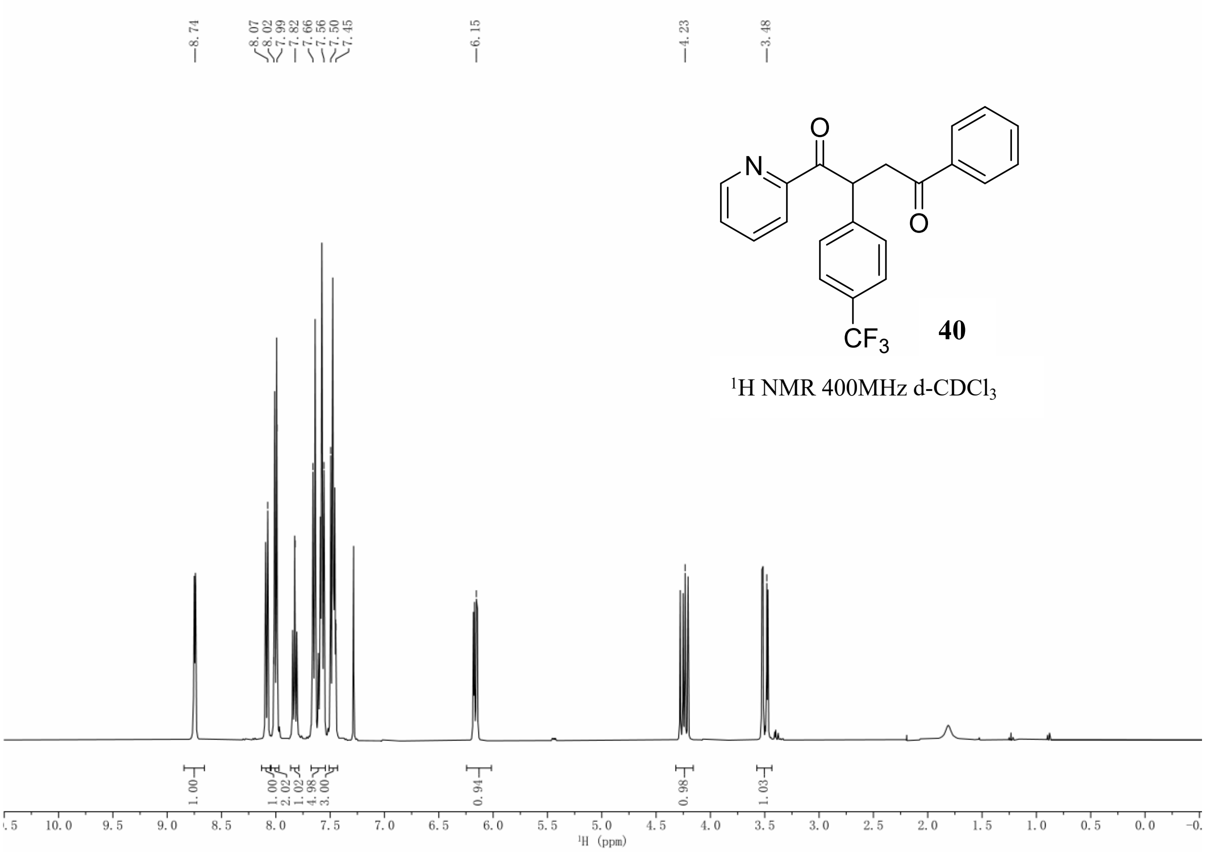
8.74
8.07 8.02 7.99 7.82 7.66 7.56 7.50 7.45
6.15
4.23
3.48
40
^{1}H NMR 400MHz d-CDCl$_3$
1.00 1.00 2.02 1.02 4.98 3.00 0.94 0.98 1.03
^{1}H (ppm)

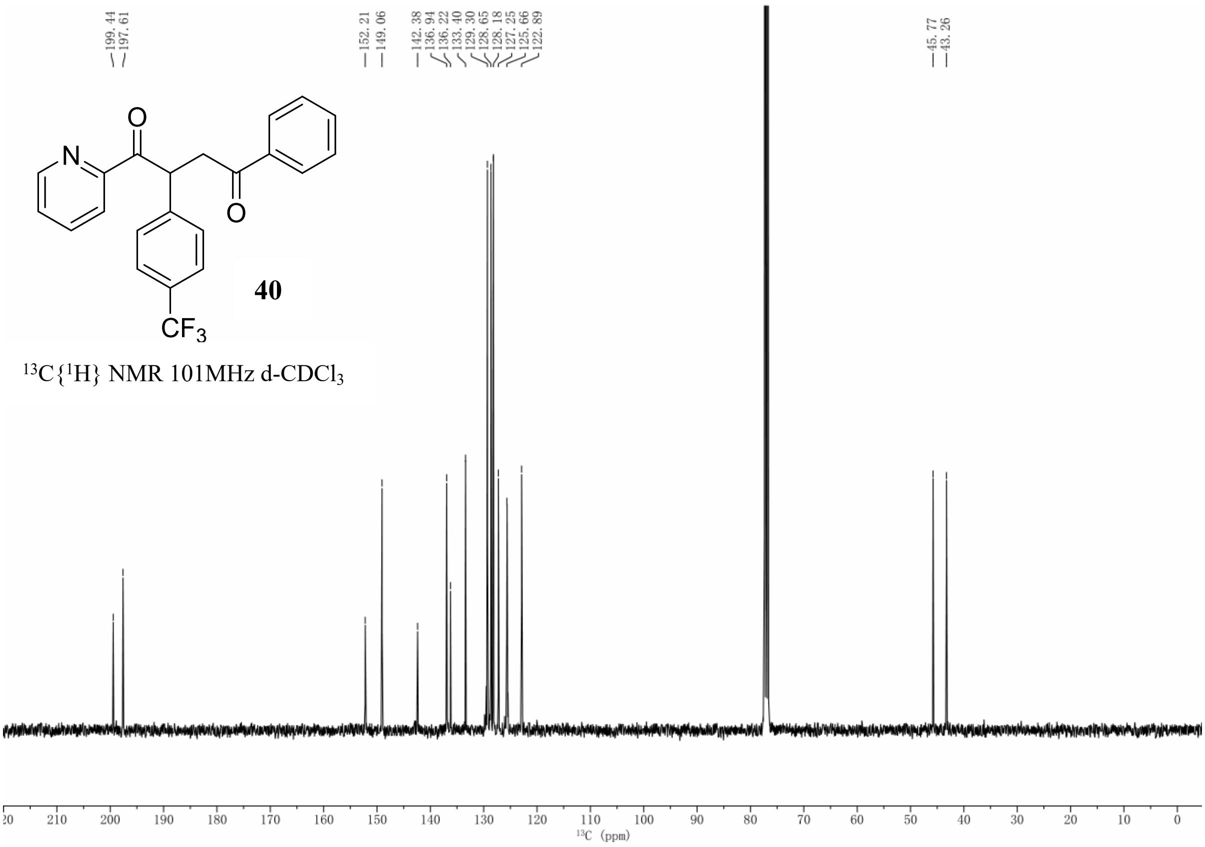
199.44 197.61
152.21 149.06
142.38 136.94 136.22 133.40 129.30 128.65 128.18 127.25 125.66 122.89
45.77 43.26
40
^{13}C{^{1}H} NMR 101MHz d-CDCl$_3$
^{13}C (ppm)

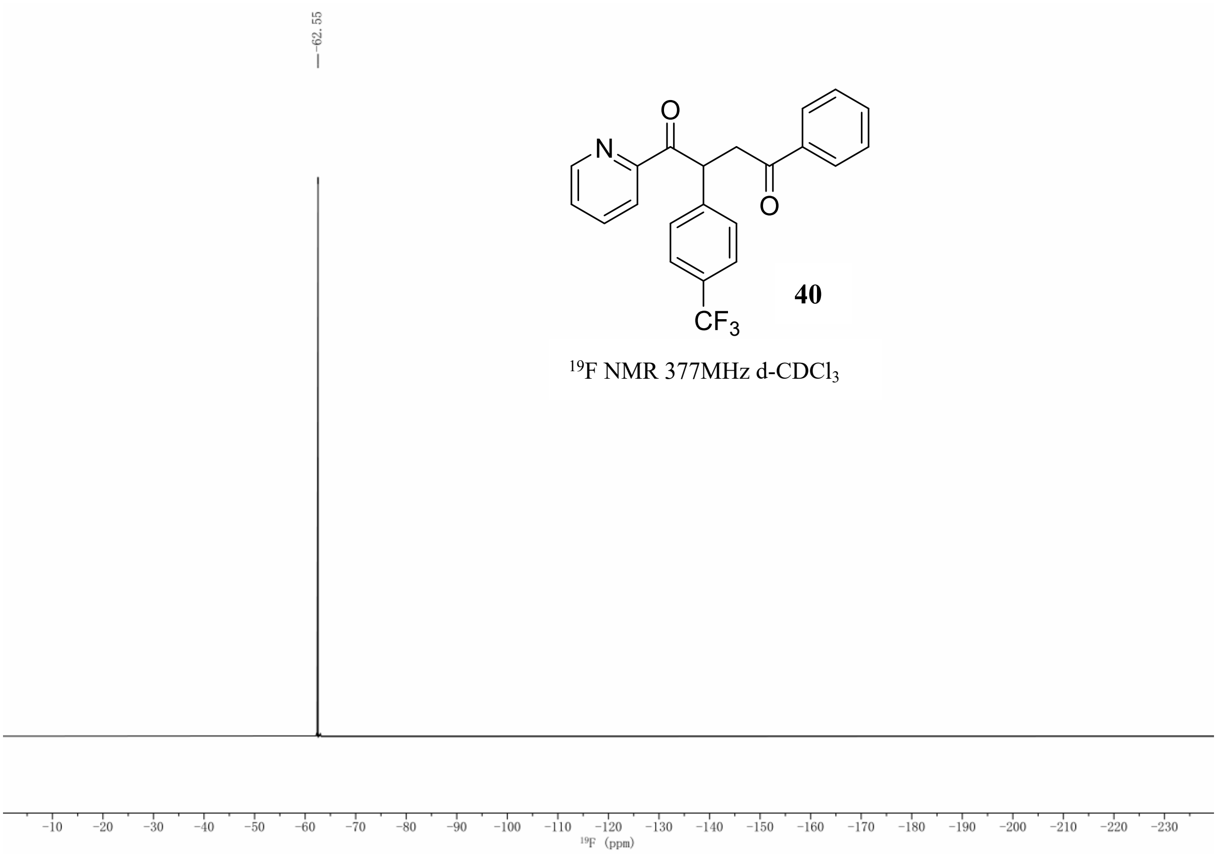
—-62.55
O
N
O
40
CF3
19F NMR 377MHz d-CDCl3
-10 -20 -30 -40 -50 -60 -70 -80 -90 -100 -110 -120 -130 -140 -150 -160 -170 -180 -190 -200 -210 -220 -230
19F (ppm)

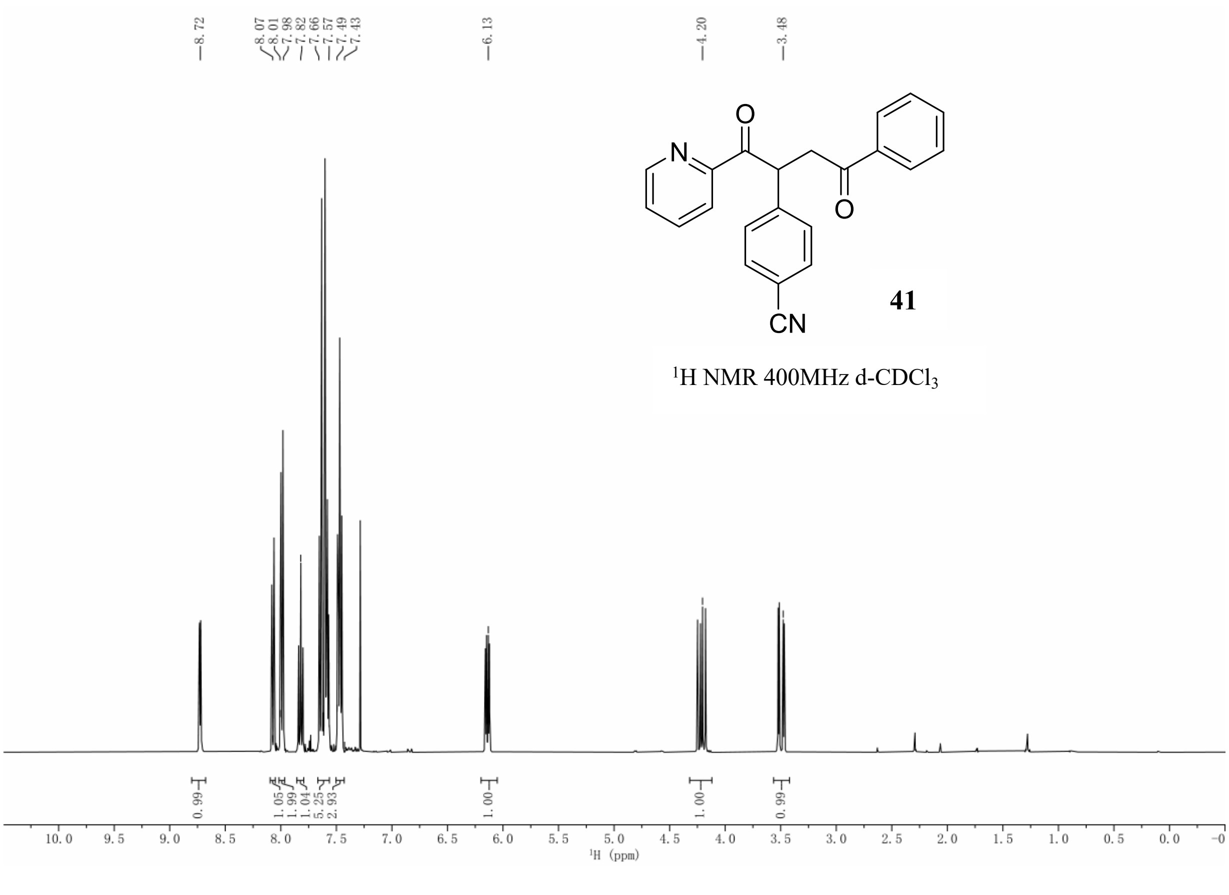
—8.72
8.07 8.01 7.98 7.82 7.66 7.57 7.49 7.43
—6.13
—4.20
—3.48
O
N
O
41
CN
1H NMR 400MHz d-CDCl3
0.99
1.05 1.99 1.04 5.25 2.93
1.00
1.00
0.99
10.0 9.5 9.0 8.5 8.0 7.5 7.0 6.5 6.0 5.5 5.0 4.5 4.0 3.5 3.0 2.5 2.0 1.5 1.0 0.5 0.0
1H (ppm)

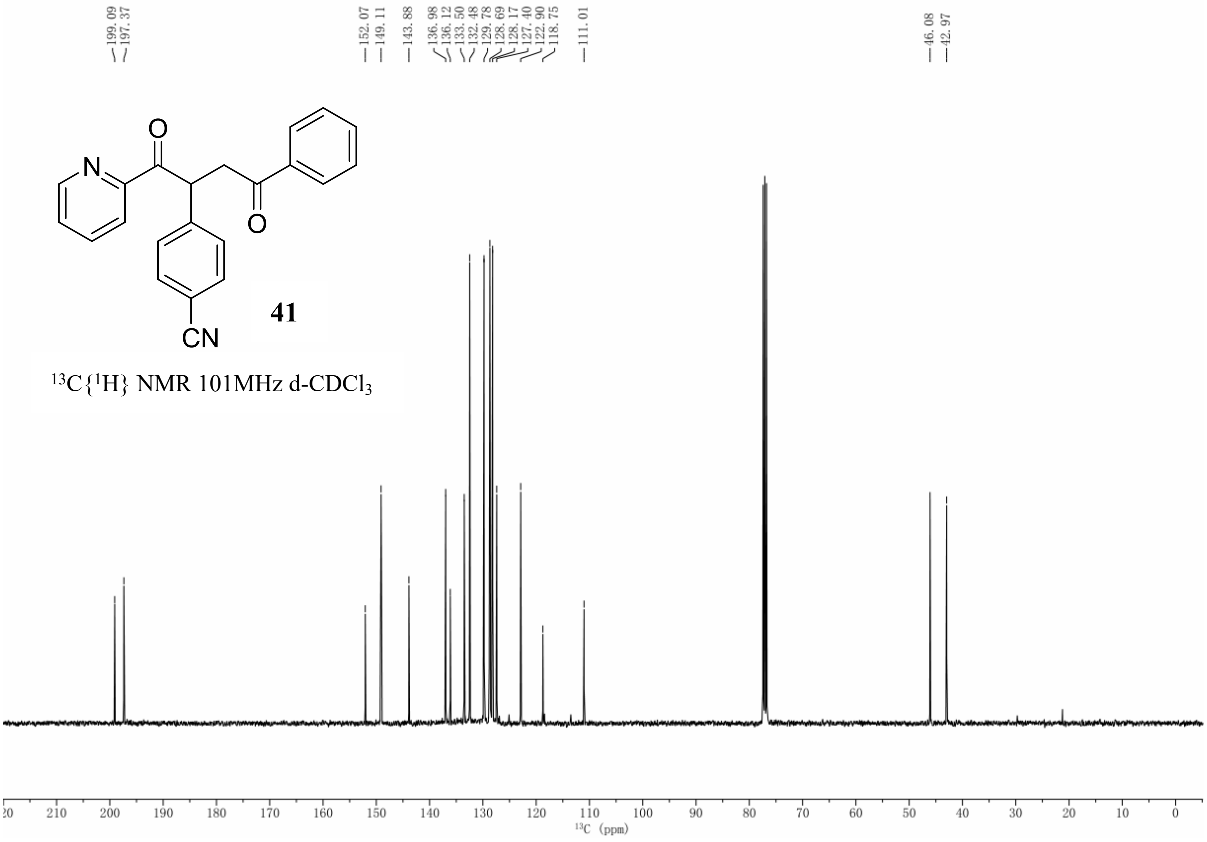
199.09
197.37
152.07
149.11
143.88
136.98
136.12
133.50
132.48
129.78
128.69
128.17
127.40
122.90
118.75
111.01
46.08
42.97
O
N
O
CN
41
$^{13}C\{^{1}H\}$ NMR 101MHz d-$CDCl_3$
^{13}C (ppm)

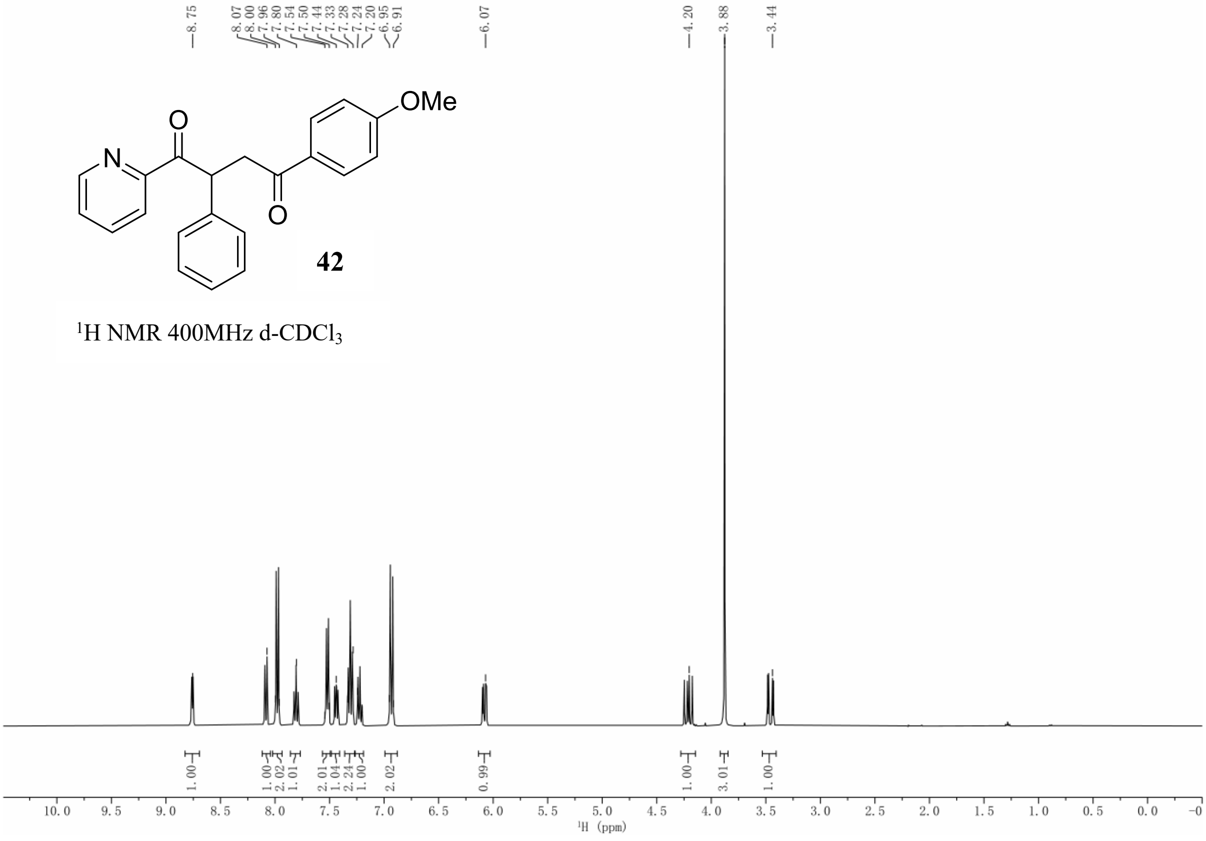
8.75
8.07
8.00
7.96
7.80
7.54
7.50
7.44
7.33
7.28
7.24
7.20
6.95
6.91
6.07
4.20
3.88
3.44
OMe
O
N
O
42
^{1}H NMR 400MHz d-$CDCl_3$
1.00
1.00
2.02
1.01
2.01
1.04
2.24
1.00
2.02
0.99
1.00
3.01
1.00
^{1}H (ppm)

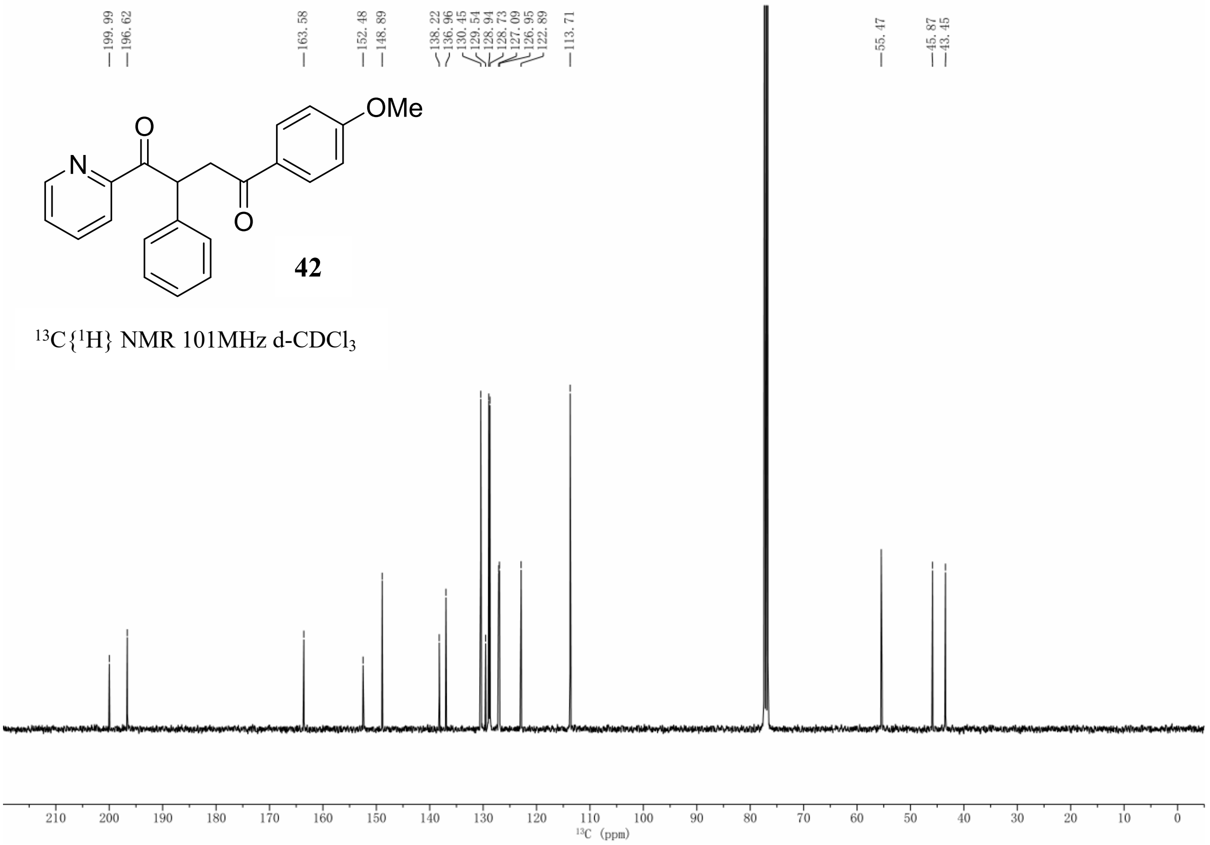
199.99
196.62
163.58
152.48
148.89
138.22
136.96
130.45
129.54
128.94
128.73
127.09
126.95
122.89
113.71
55.47
45.87
43.45
OMe
N
O
O
42
$^{13}C\{^{1}H\}$ NMR 101MHz d-$CDCl_3$
210 200 190 180 170 160 150 140 130 120 110 100 90 80 70 60 50 40 30 20 10 0
^{13}C (ppm)

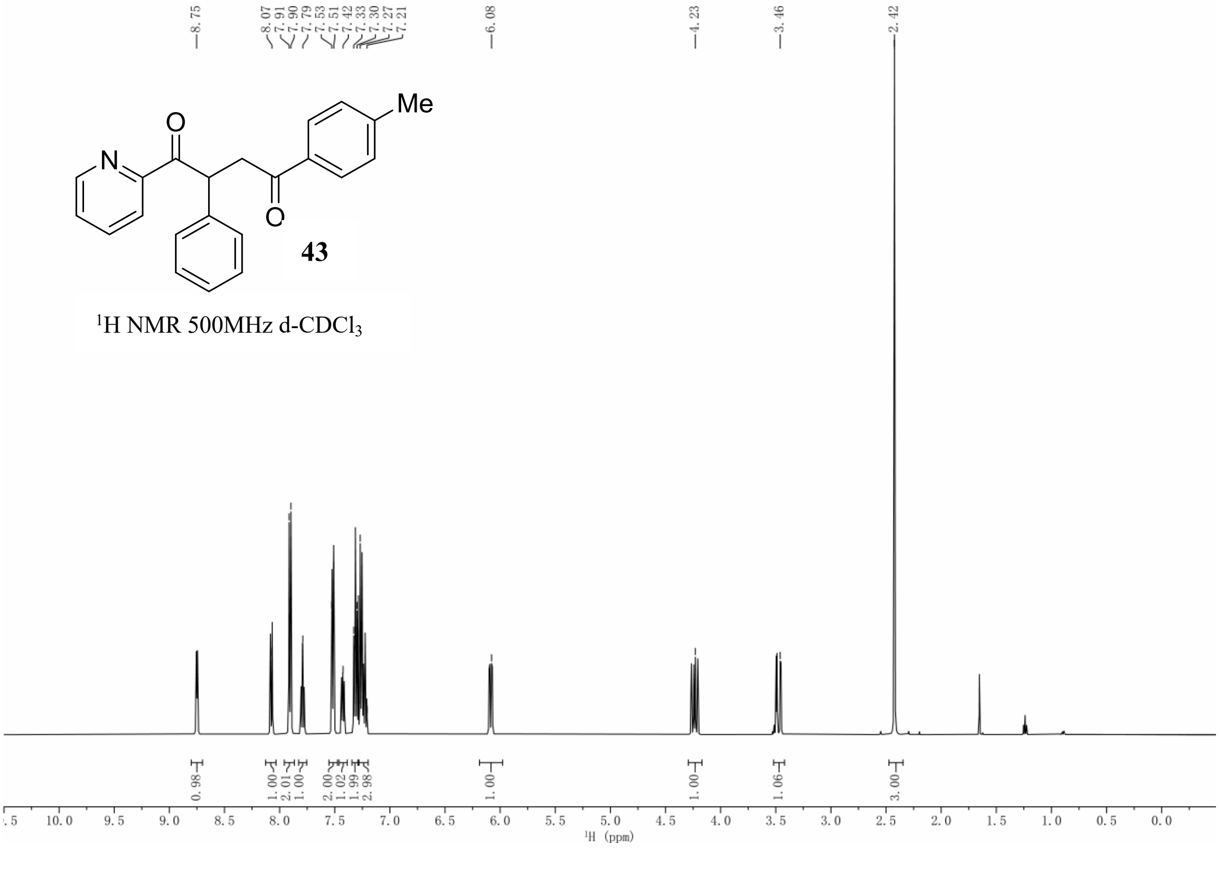
8.75
8.07
7.91
7.90
7.79
7.53
7.51
7.42
7.33
7.30
7.27
7.21
6.08
4.23
3.46
2.42
Me
N
O
O
43
^{1}H NMR 500MHz d-$CDCl_3$
0.98
1.00
2.01
1.00
2.00
1.02
1.99
2.98
1.00
1.00
1.06
3.00
10.0 9.5 9.0 8.5 8.0 7.5 7.0 6.5 6.0 5.5 5.0 4.5 4.0 3.5 3.0 2.5 2.0 1.5 1.0 0.5 0.0
^{1}H (ppm)

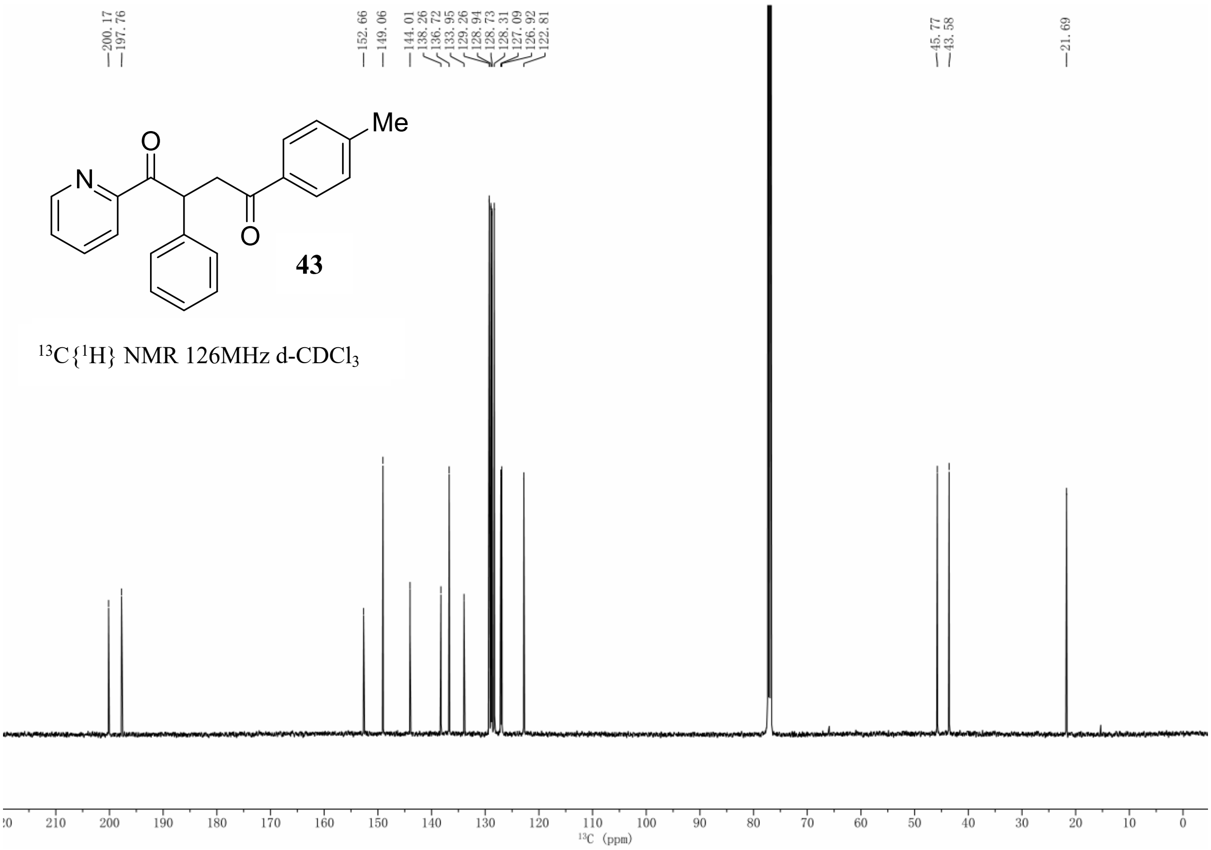

200.17
197.76
152.66
149.06
144.01
138.26
136.72
133.95
129.26
128.94
128.73
128.31
127.09
126.92
122.81
45.77
43.58
21.69
Me
N
O
O
43
$^{13}C\{^{1}H\}$ NMR 126MHz d-CDCl$_3$
210 200 190 180 170 160 150 140 130 120 110 100 90 80 70 60 50 40 30 20 10 0
^{13}C (ppm)

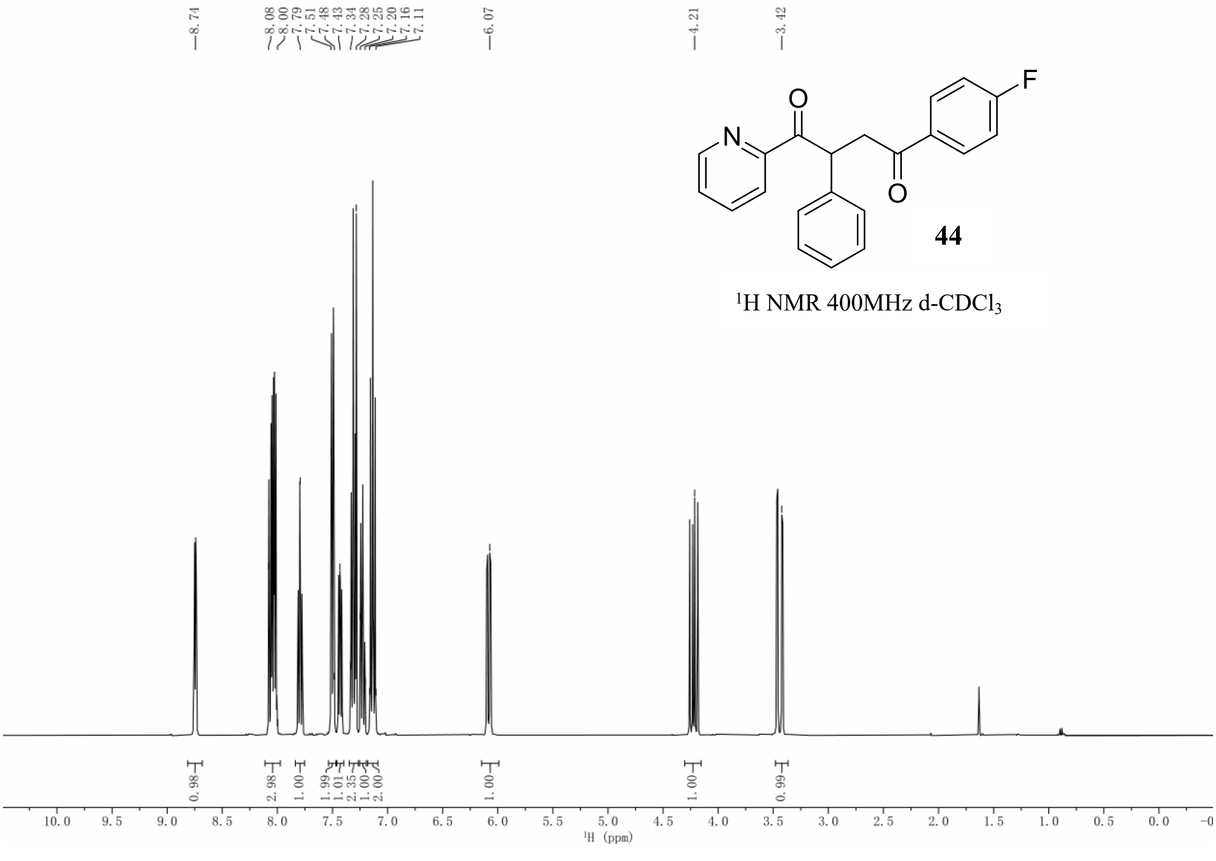

8.74
8.08
8.00
7.79
7.51
7.48
7.43
7.34
7.28
7.25
7.20
7.16
7.11
6.07
4.21
3.42
F
N
O
O
44
^{1}H NMR 400MHz d-CDCl$_3$
0.98
2.98
1.00
1.99
1.01
2.35
1.00
2.00
1.00
1.00
0.99
10.0 9.5 9.0 8.5 8.0 7.5 7.0 6.5 6.0 5.5 5.0 4.5 4.0 3.5 3.0 2.5 2.0 1.5 1.0 0.5 0.0
^{1}H (ppm)

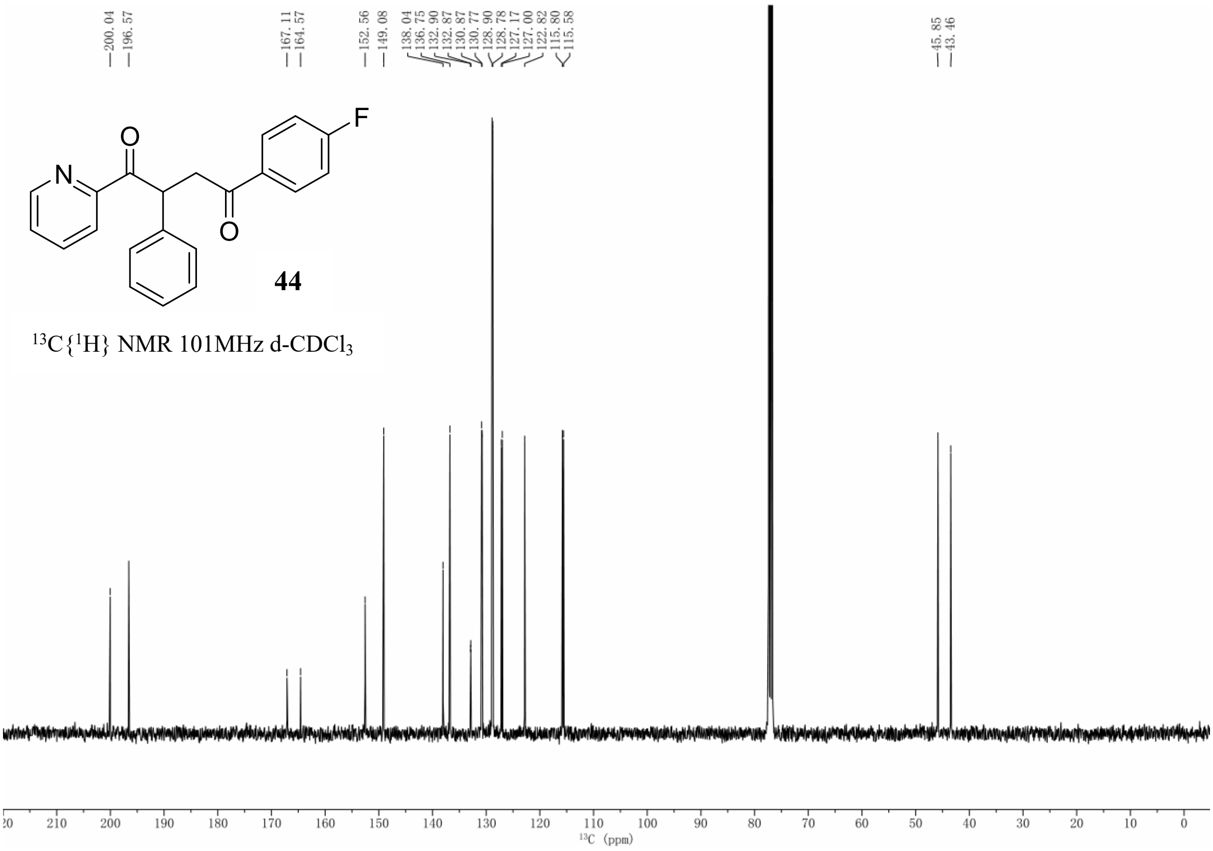
200.04
196.57
167.11
164.57
152.56
149.08
138.04
136.75
132.90
132.87
130.87
130.77
128.90
128.78
127.17
127.00
122.82
115.80
115.58
45.85
43.46
F
O
N
O
44
$^{13}C\{^{1}H\}$ NMR 101MHz d-CDCl$_3$
^{13}C (ppm)

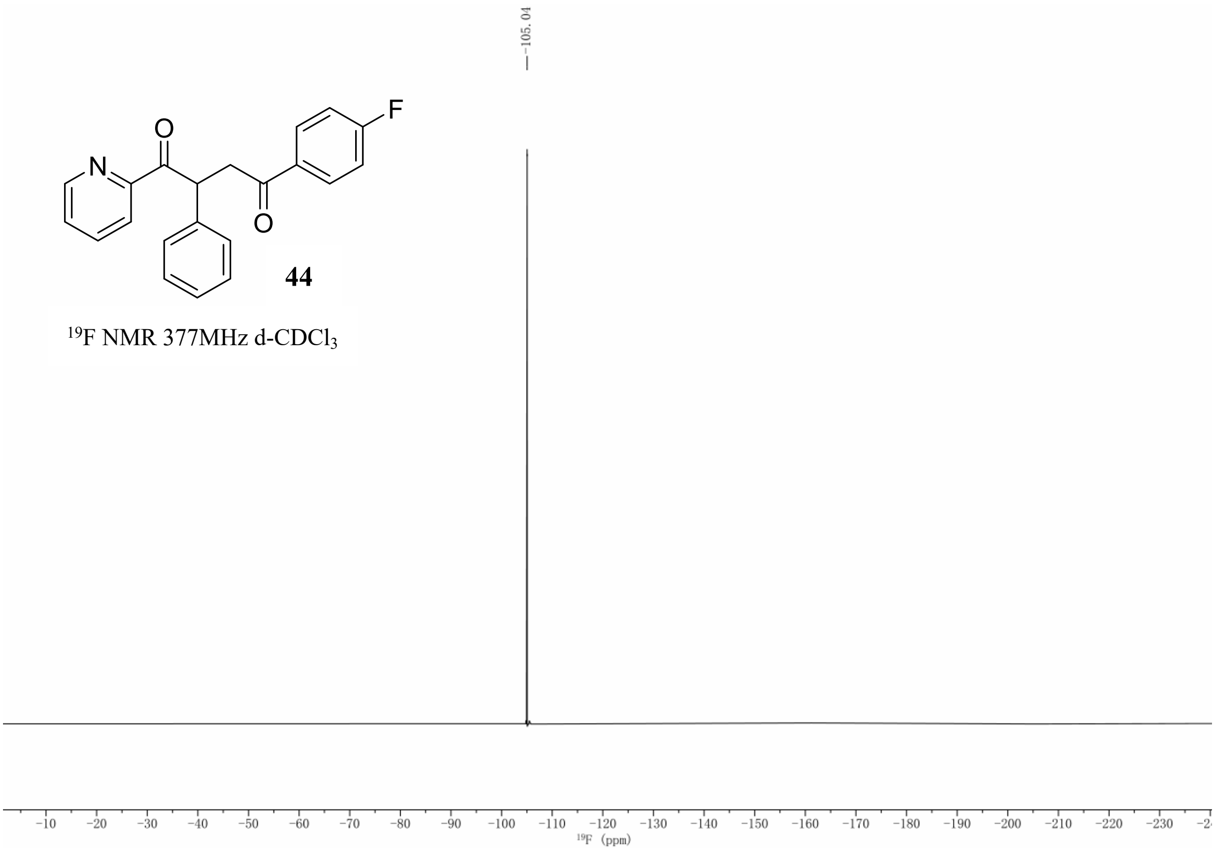
-105.04
F
O
N
O
44
^{19}F NMR 377MHz d-CDCl$_3$
^{19}F (ppm)

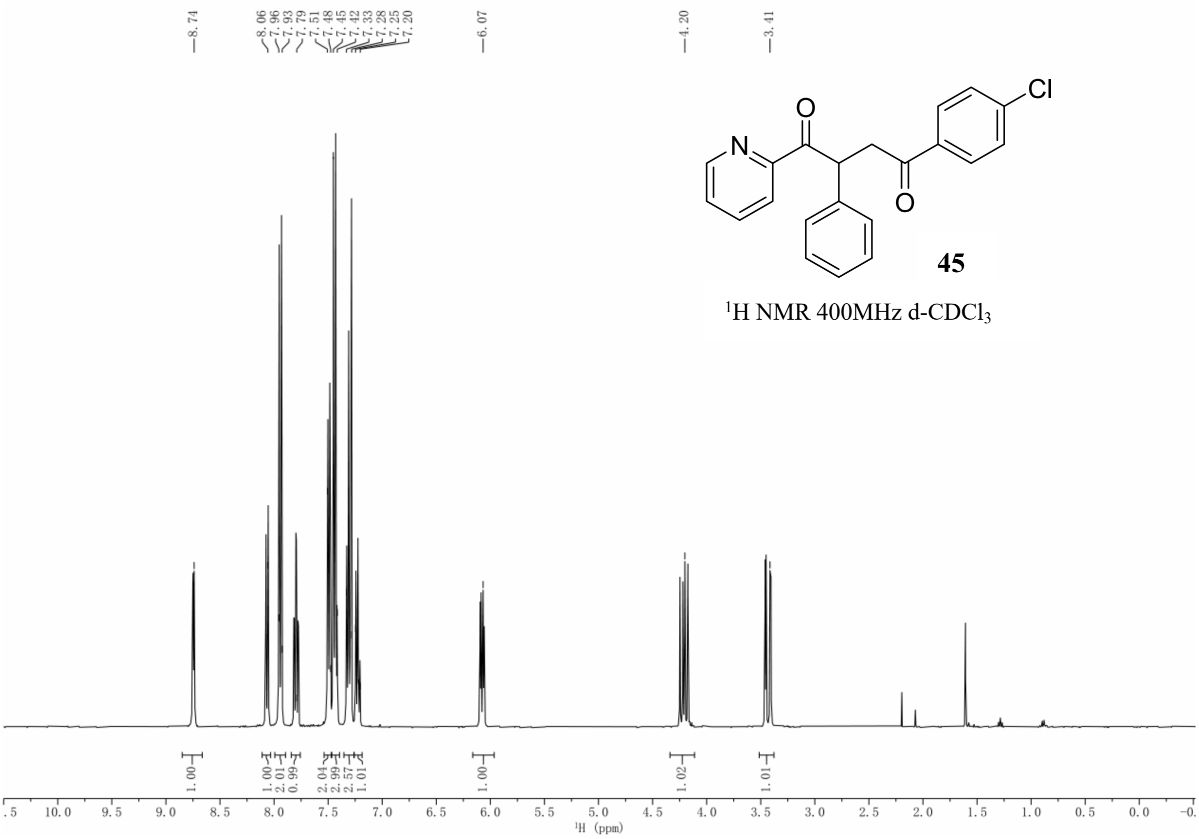

8.74
8.06
7.96
7.93
7.79
7.51
7.48
7.45
7.42
7.33
7.28
7.25
7.20
6.07
4.20
3.41
O
N
Cl
O
45
1H NMR 400MHz d-CDCl3
1.00
1.00
2.01
0.99
2.04
2.99
2.57
1.01
1.00
1.02
1.01
1H (ppm)

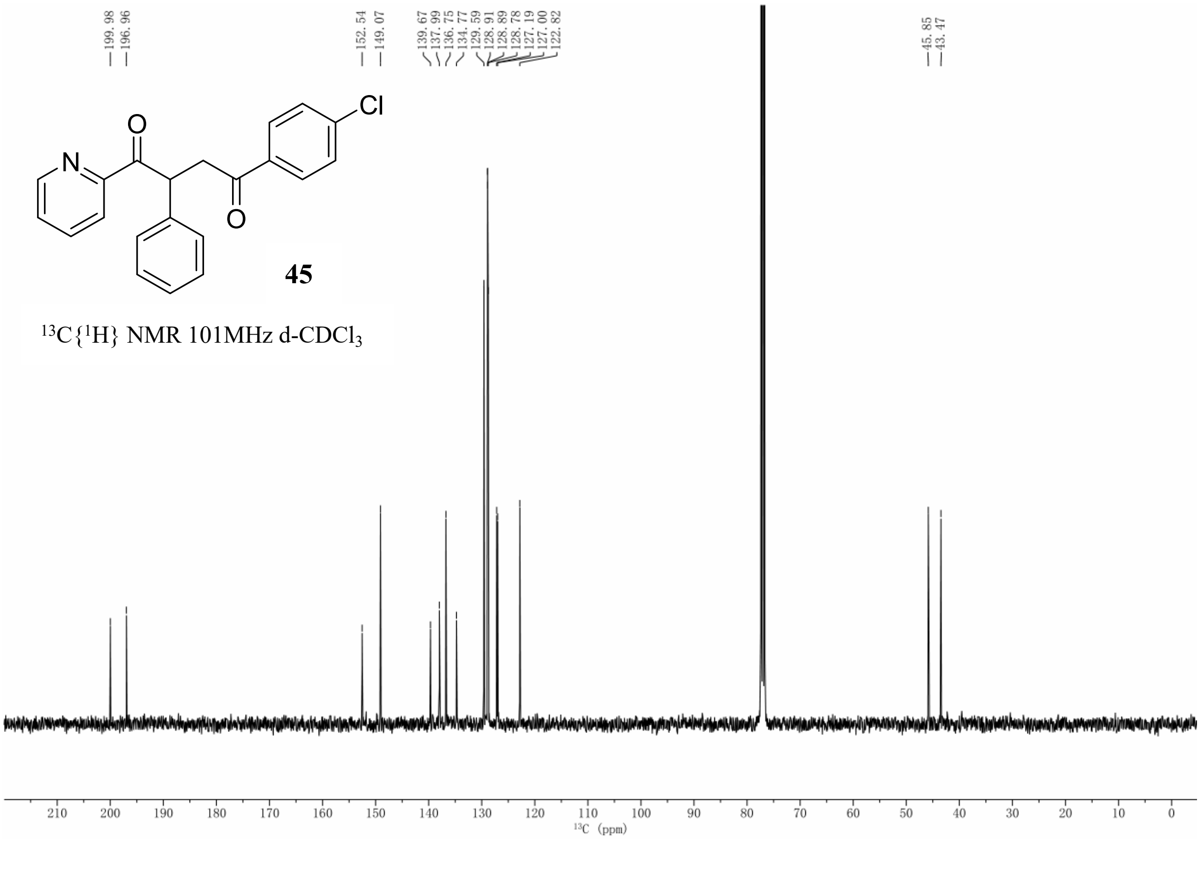

199.98
196.96
152.54
149.07
139.67
137.99
136.75
134.77
129.59
128.91
128.89
128.78
127.19
127.00
122.82
45.85
43.47
O
N
Cl
O
45
13C{1H} NMR 101MHz d-CDCl3
13C (ppm)

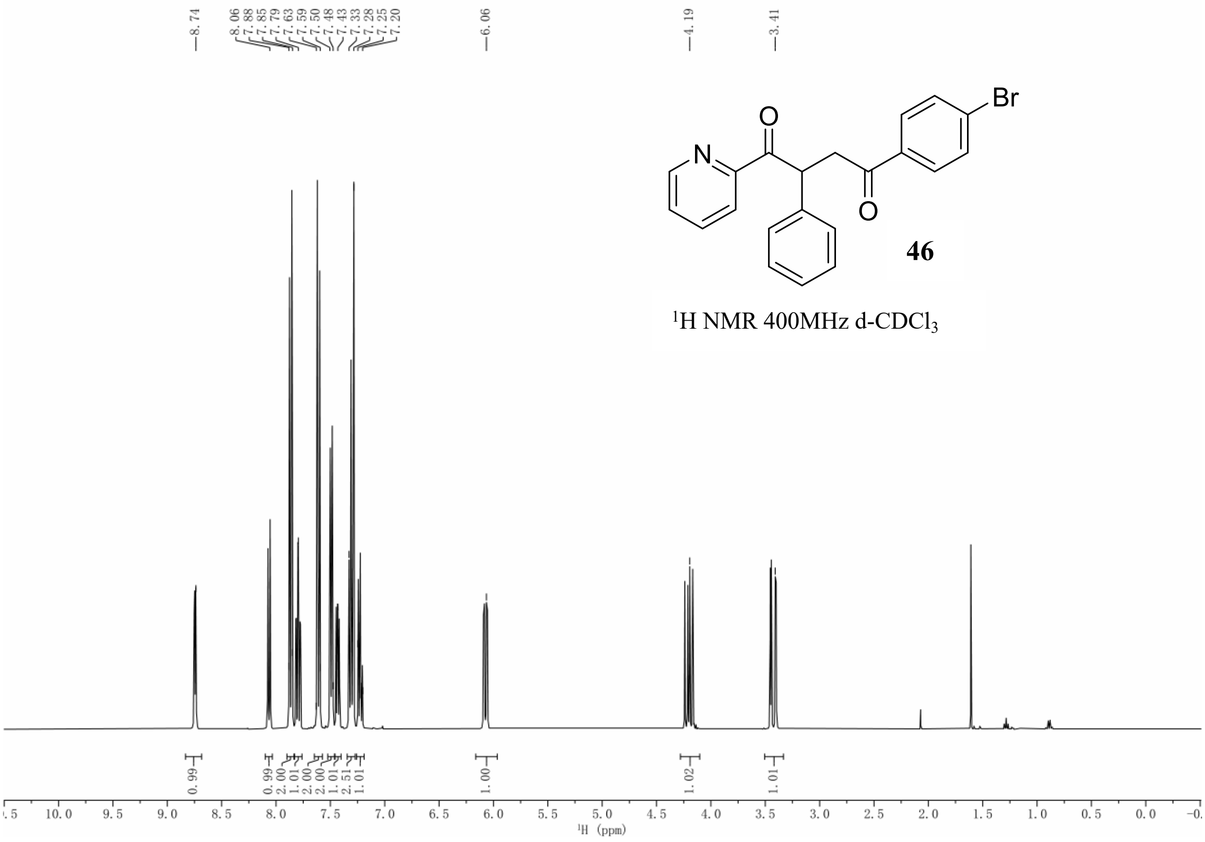

8.74
8.06 7.88 7.85 7.79 7.63 7.59 7.50 7.48 7.43 7.33 7.28 7.25 7.20
6.06
4.19
3.41
Br
N
O
O
46
1H NMR 400MHz d-CDCl3
0.99 0.99 2.00 1.01 2.00 2.00 1.01 2.51 1.01 1.00 1.02 1.01
1H (ppm)

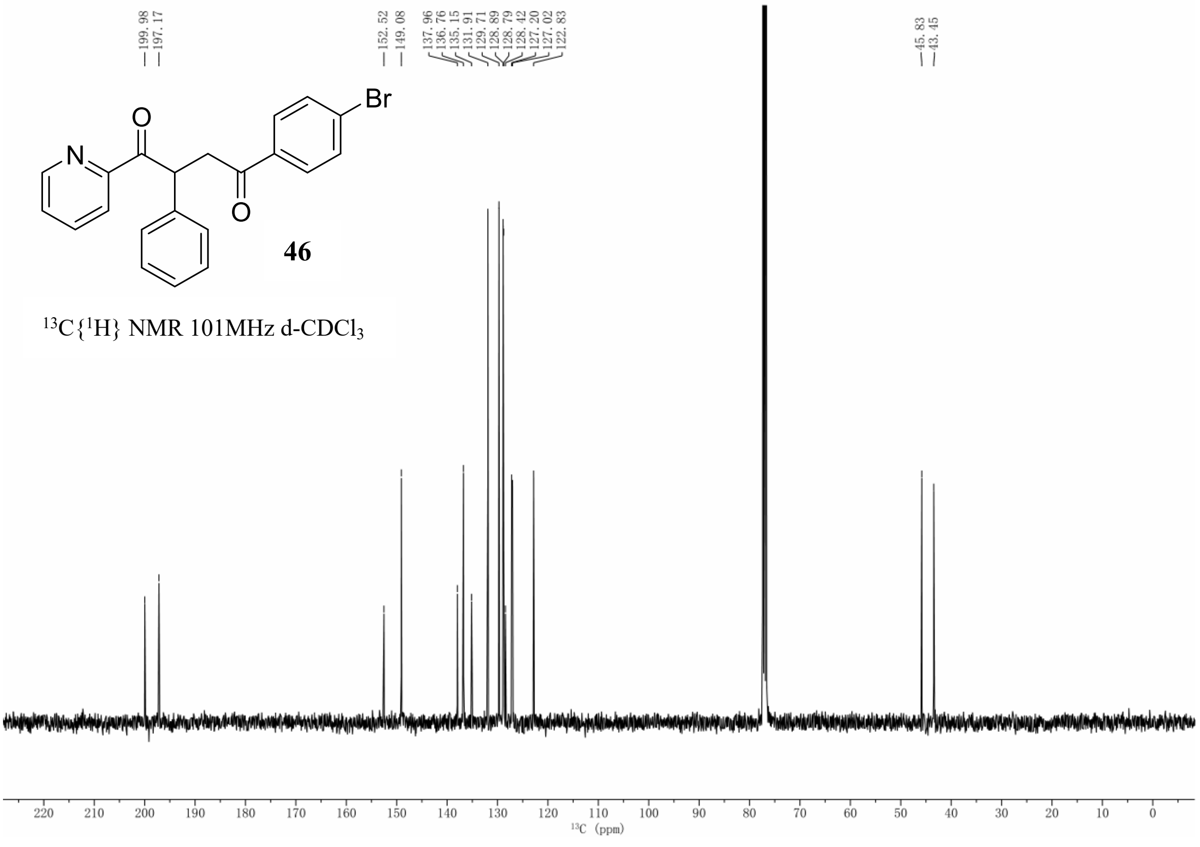

199.98
197.17
152.52
149.08
137.96 136.76 135.15 131.91 129.71 128.89 128.79 128.42 127.20 127.02 122.83
45.83
43.45
Br
N
O
O
46
13C{1H} NMR 101MHz d-CDCl3
13C (ppm)

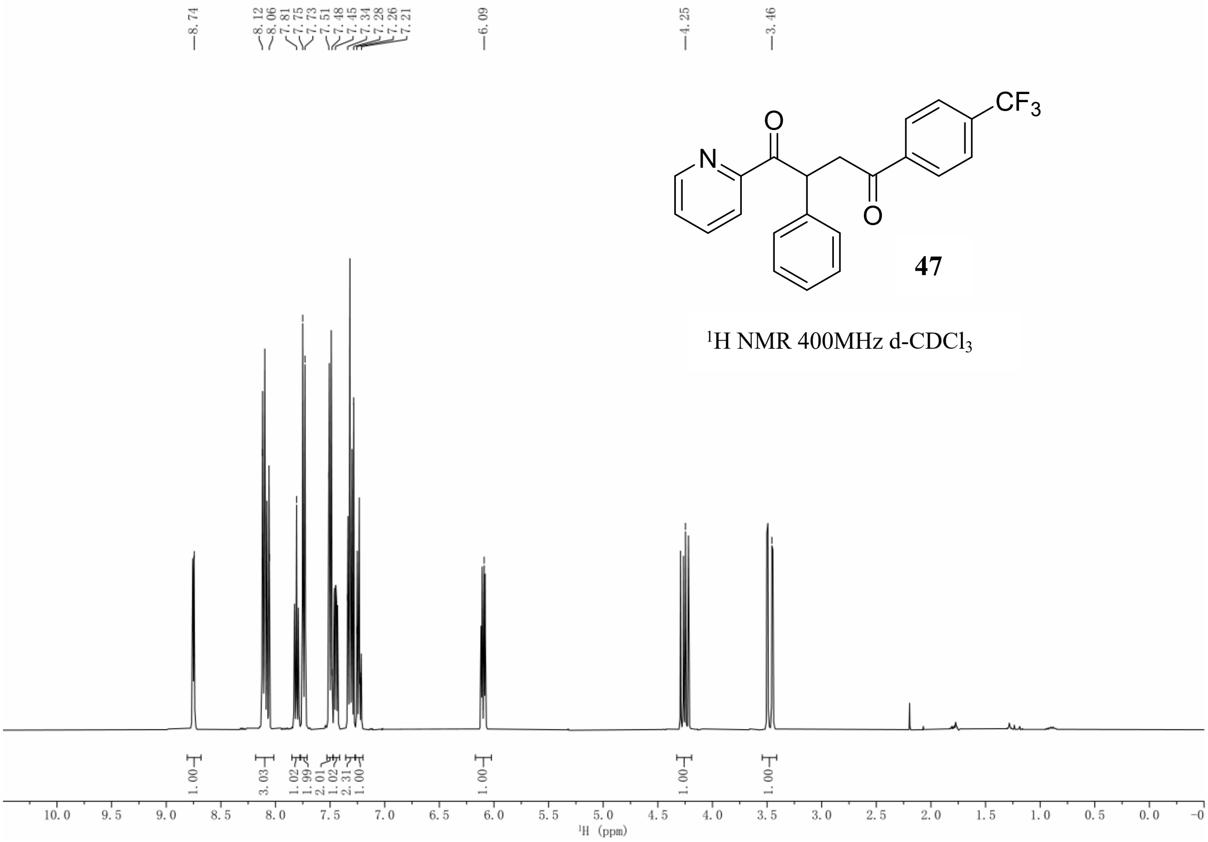
8.74
8.12 8.06 7.81 7.75 7.73 7.51 7.48 7.45 7.34 7.28 7.26 7.21
6.09
4.25
3.46
CF3
O
N
O
47
1H NMR 400MHz d-CDCl3
1.00 3.03 1.02 1.99 2.01 1.02 2.31 1.00 1.00 1.00 1.00
10.0 9.5 9.0 8.5 8.0 7.5 7.0 6.5 6.0 5.5 5.0 4.5 4.0 3.5 3.0 2.5 2.0 1.5 1.0 0.5 0.0
1H (ppm)

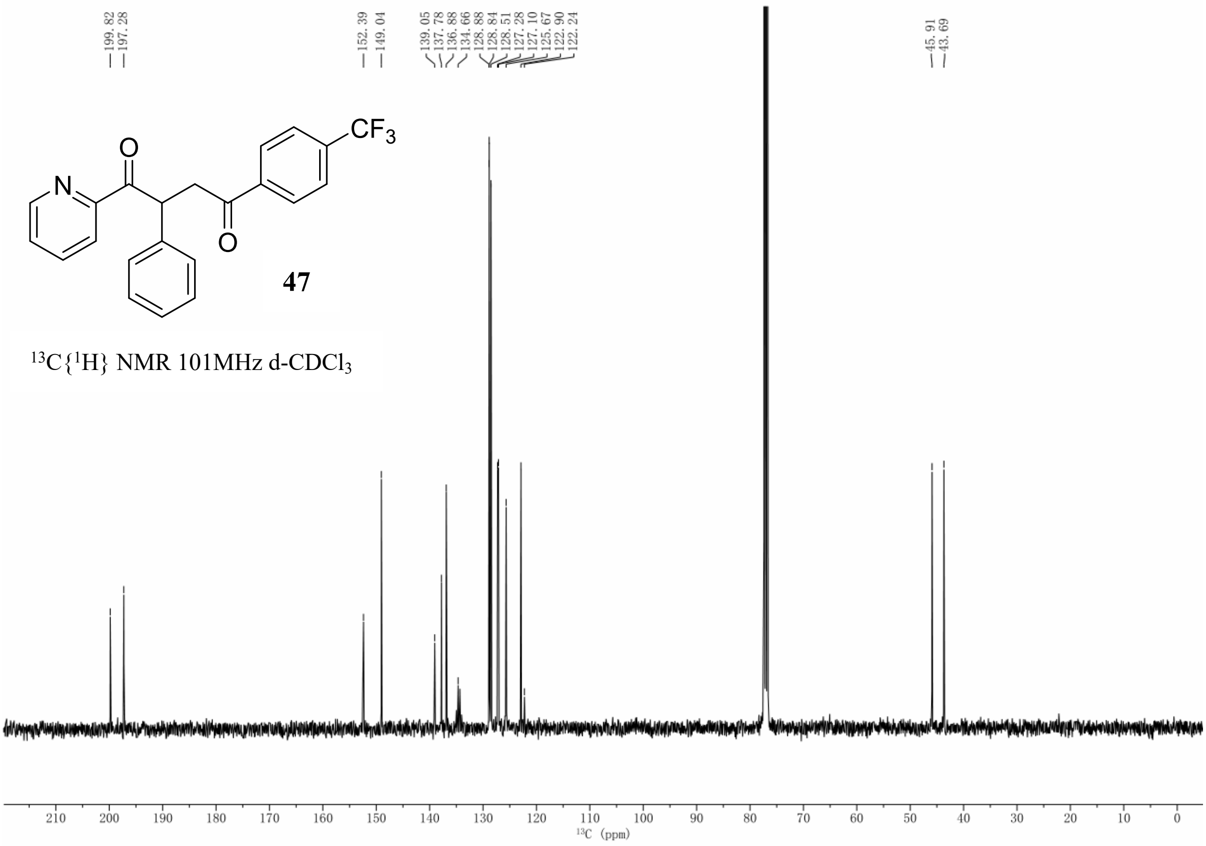
199.82 197.28
152.39 149.04
139.05 137.78 136.88 134.66 128.88 128.84 128.51 127.28 127.10 125.67 122.90 122.24
45.91 43.69
CF3
O
N
O
47
13C{1H} NMR 101MHz d-CDCl3
210 200 190 180 170 160 150 140 130 120 110 100 90 80 70 60 50 40 30 20 10 0
13C (ppm)

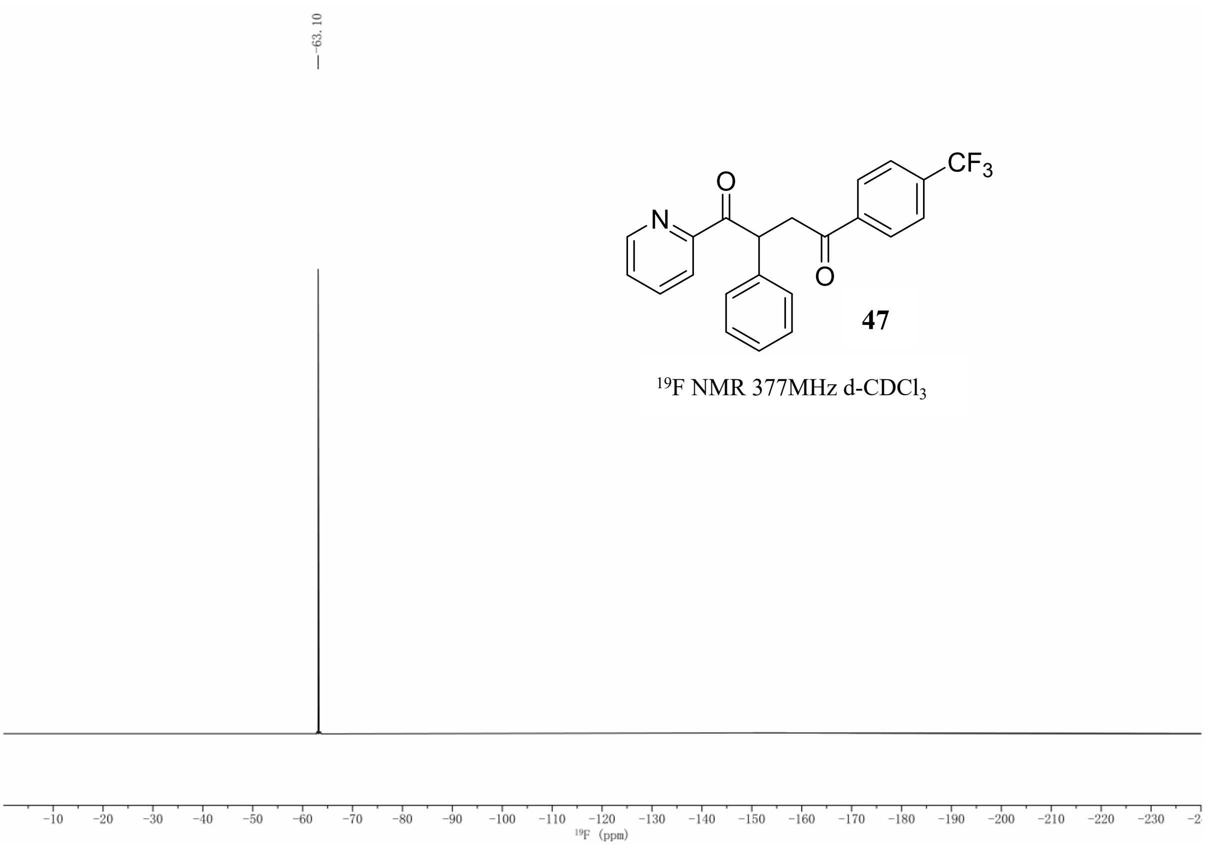
-63.10
CF3
O
N
O
47
19F NMR 377MHz d-CDCl3
19F (ppm)

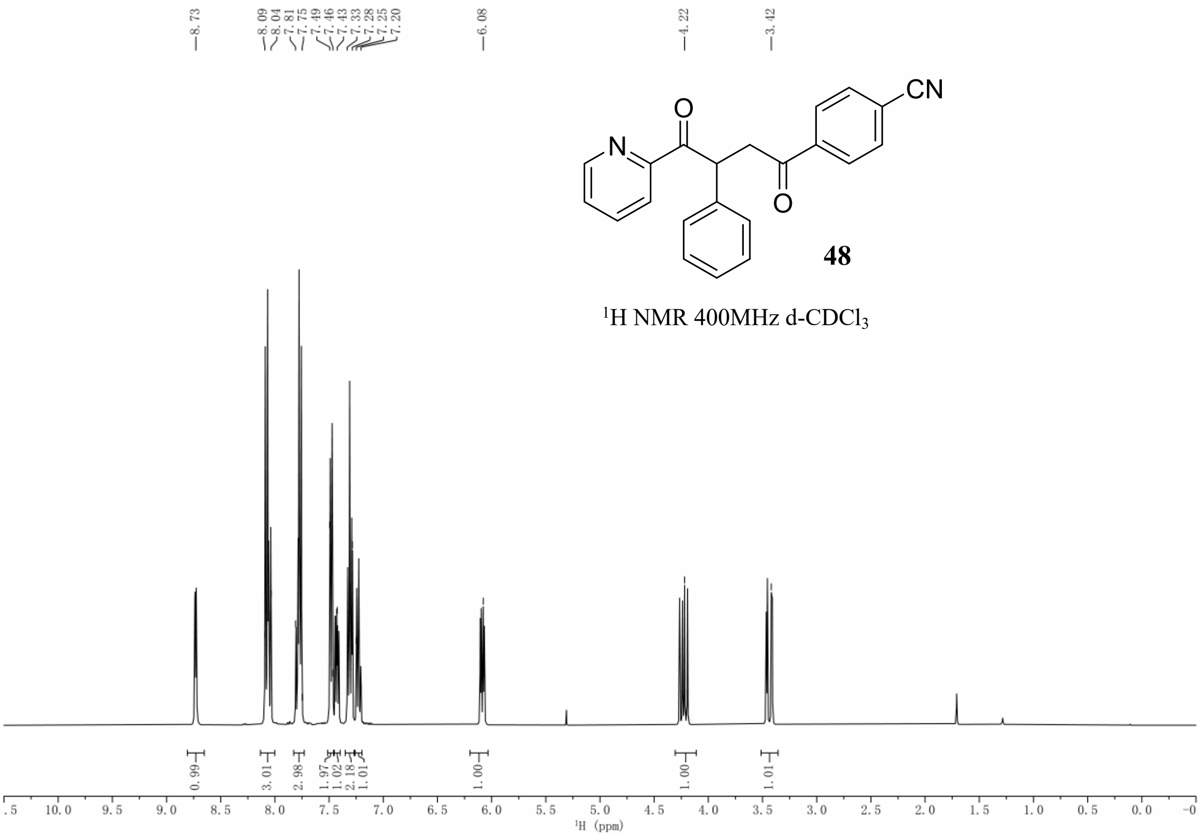
8.73
8.09
8.04
7.81
7.75
7.49
7.46
7.43
7.33
7.28
7.25
7.20
6.08
4.22
3.42
CN
O
N
O
48
1H NMR 400MHz d-CDCl3
0.99
3.01
2.98
1.97
1.02
2.18
1.01
1.00
1.00
1.01
1H (ppm)

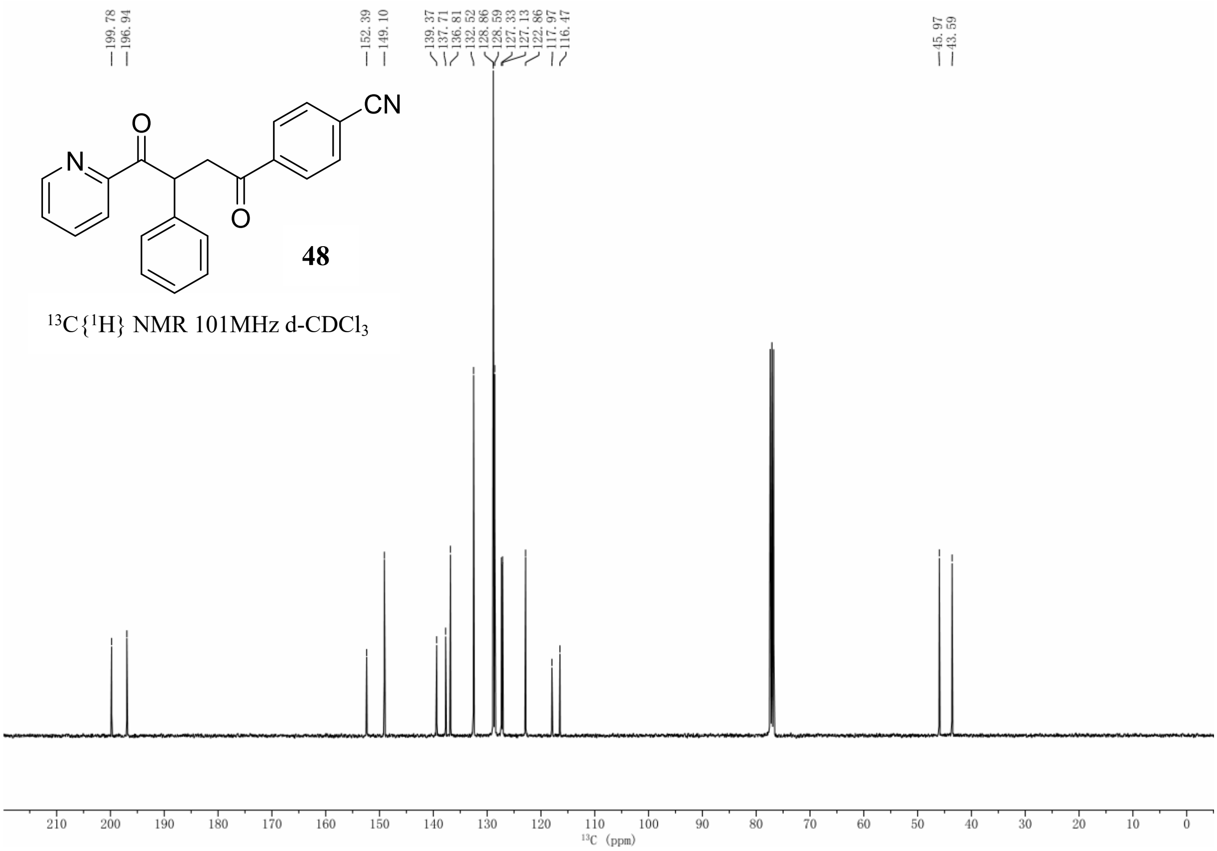
199.78
196.94
152.39
149.10
139.37
137.71
136.81
132.52
128.86
128.59
127.33
127.13
122.86
117.97
116.47
45.97
43.59
CN
O
N
O
48
13C{1H} NMR 101MHz d-CDCl3
13C (ppm)

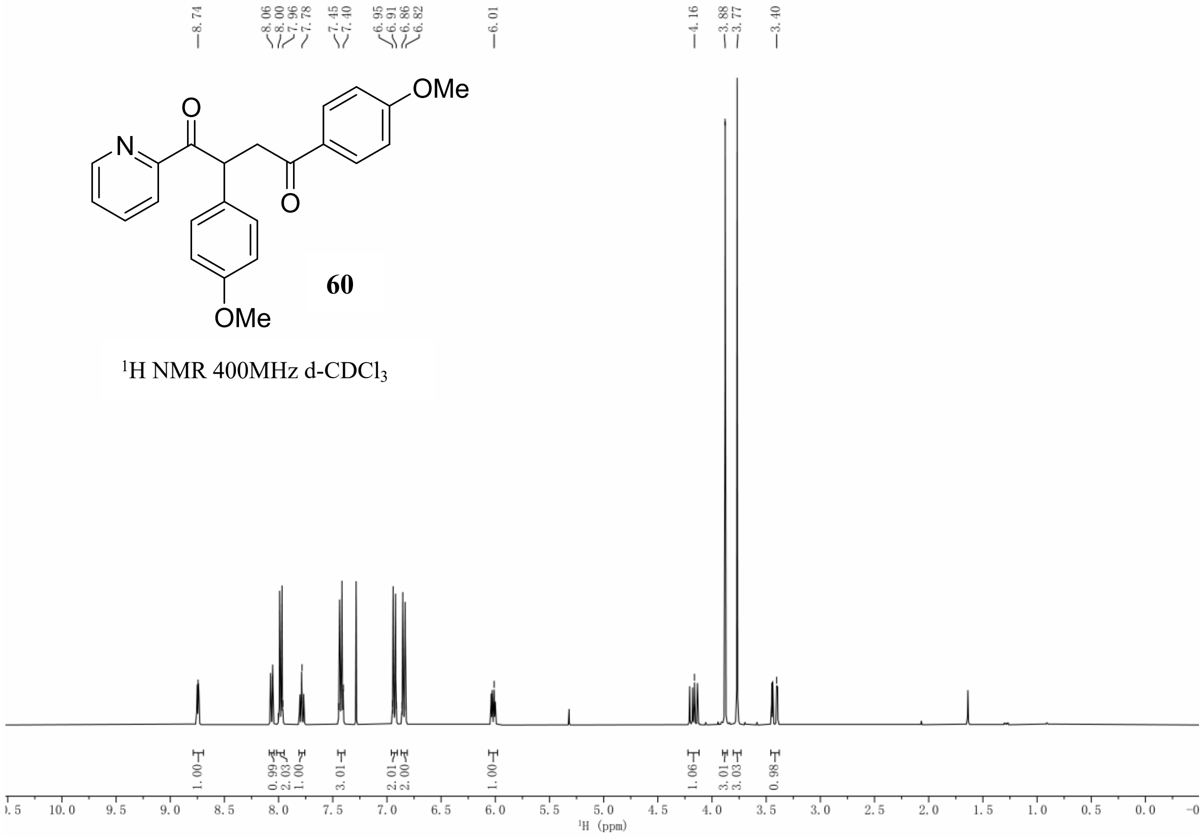
8.74
8.06
8.00
7.96
7.78
7.45
7.40
6.95
6.91
6.86
6.82
6.01
4.16
3.88
3.77
3.40
OMe
O
N
O
OMe
60
1H NMR 400MHz d-CDCl3
1H (ppm)

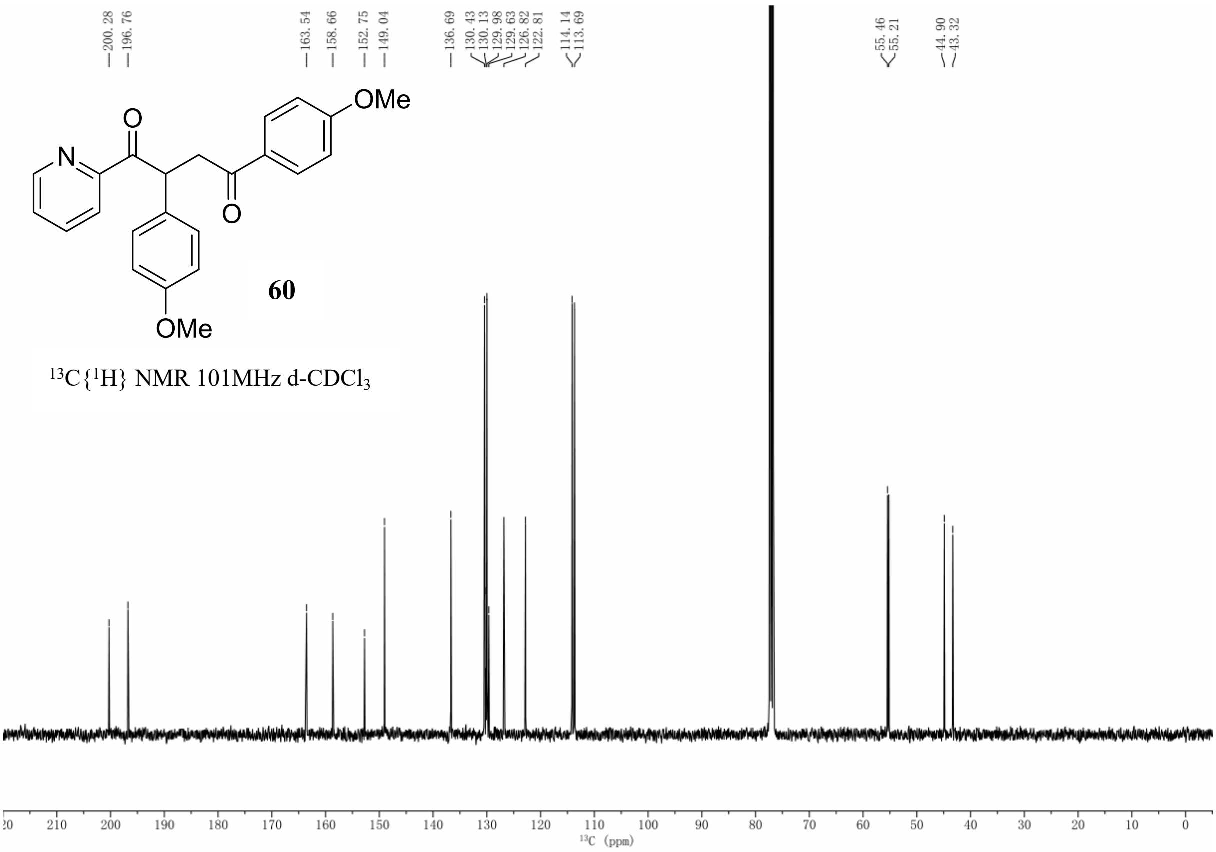
200.28
196.76
163.54
158.66
152.75
149.04
136.69
130.43
130.13
129.98
129.63
126.82
122.81
114.14
113.69
55.46
55.21
44.90
43.32
OMe
O
N
O
OMe
60
13C{1H} NMR 101MHz d-CDCl3
13C (ppm)

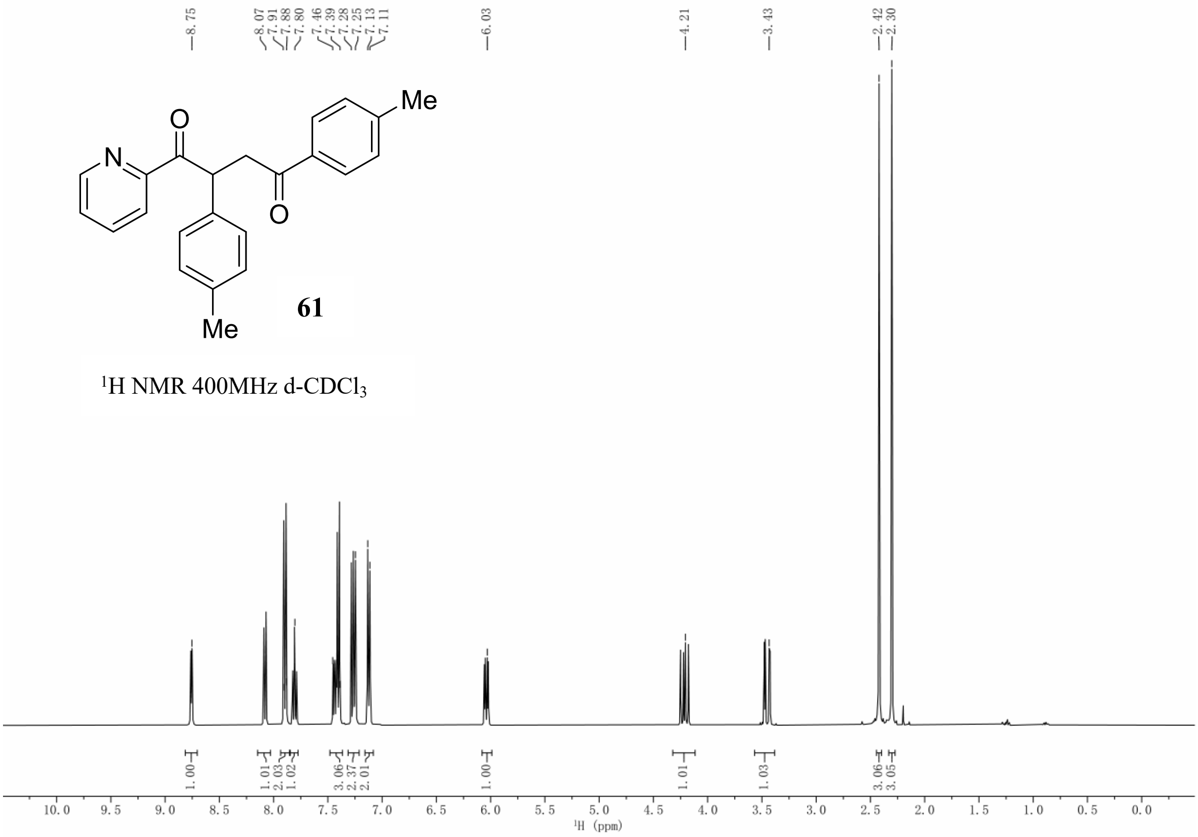
8.75
8.07
7.91
7.88
7.80
7.46
7.39
7.28
7.25
7.13
7.11
6.03
4.21
3.43
2.42
2.30
Me
O
N
O
Me
61
1H NMR 400MHz d-CDCl3
1.00
1.01
2.03
1.02
3.06
2.37
2.01
1.00
1.01
1.03
3.06
3.05
1H (ppm)

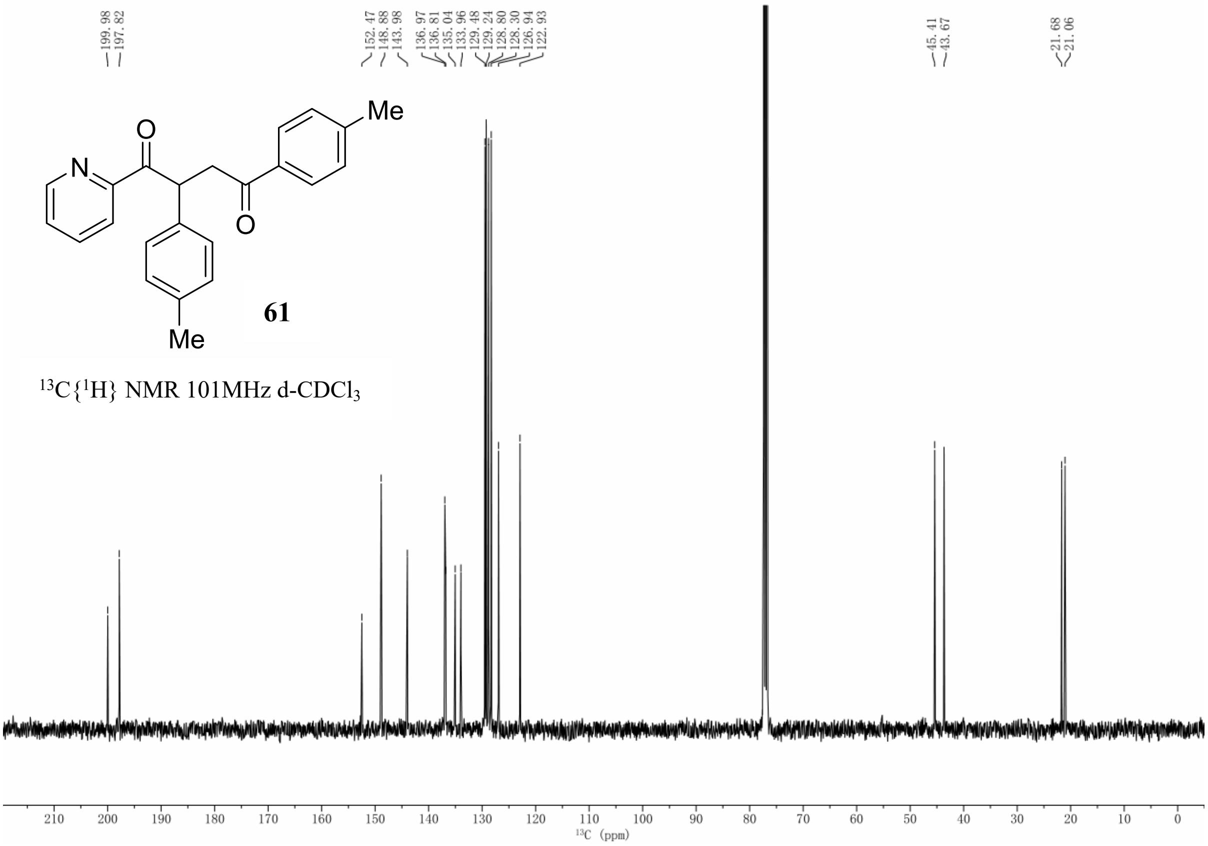
199.98
197.82
152.47
148.88
143.98
136.97
136.81
135.04
133.96
129.48
129.24
128.80
128.30
126.94
122.93
45.41
43.67
21.68
21.06
Me
O
N
O
Me
61
13C{1H} NMR 101MHz d-CDCl3
13C (ppm)

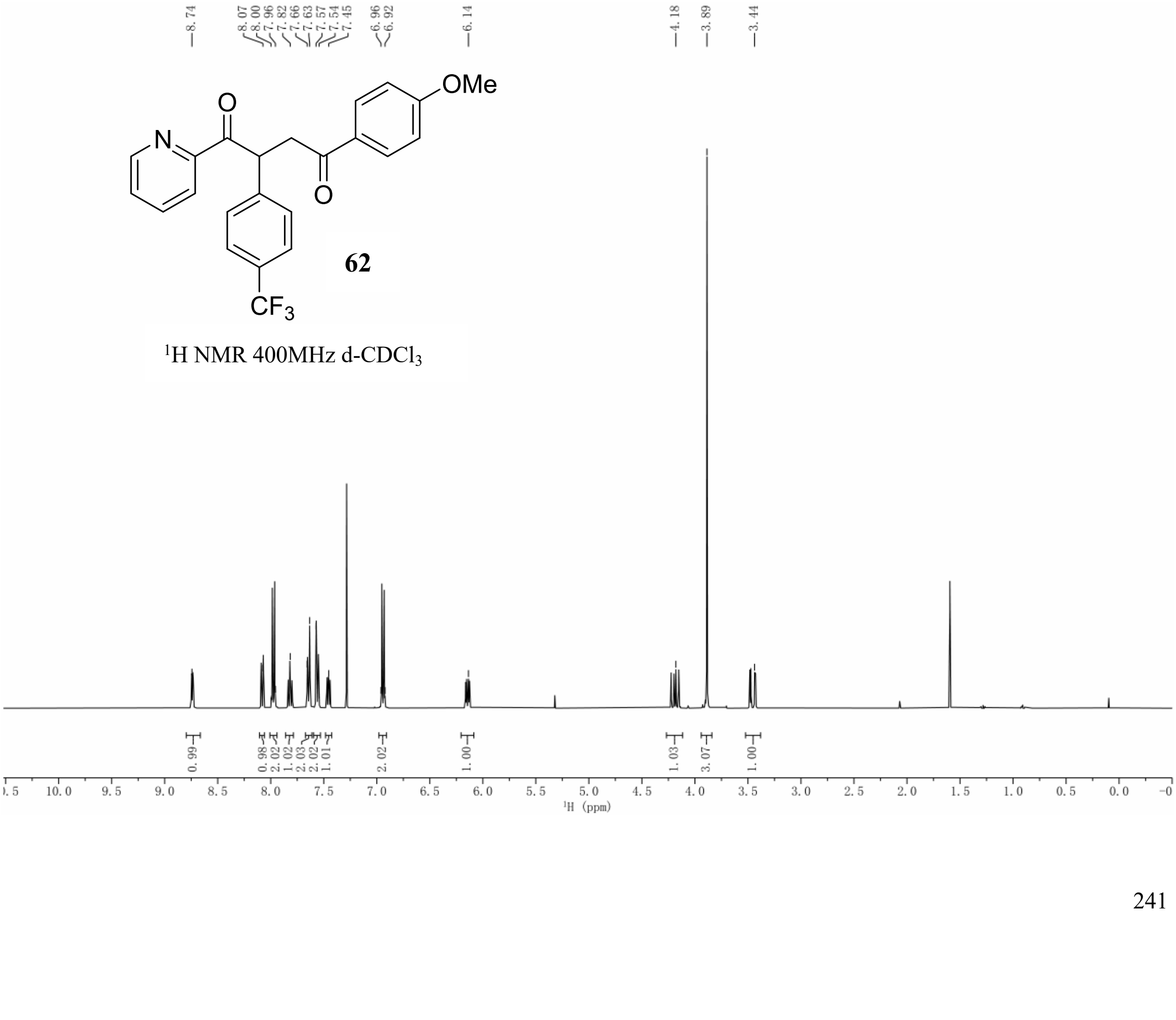
8.74
8.07
8.00
7.96
7.82
7.66
7.63
7.57
7.54
7.45
6.96
6.92
6.14
4.18
3.89
3.44
OMe
O
N
O
CF3
62
1H NMR 400MHz d-CDCl3
0.99
0.98
2.02
1.02
2.03
2.02
1.01
2.02
1.00
1.03
3.07
1.00
1H (ppm)

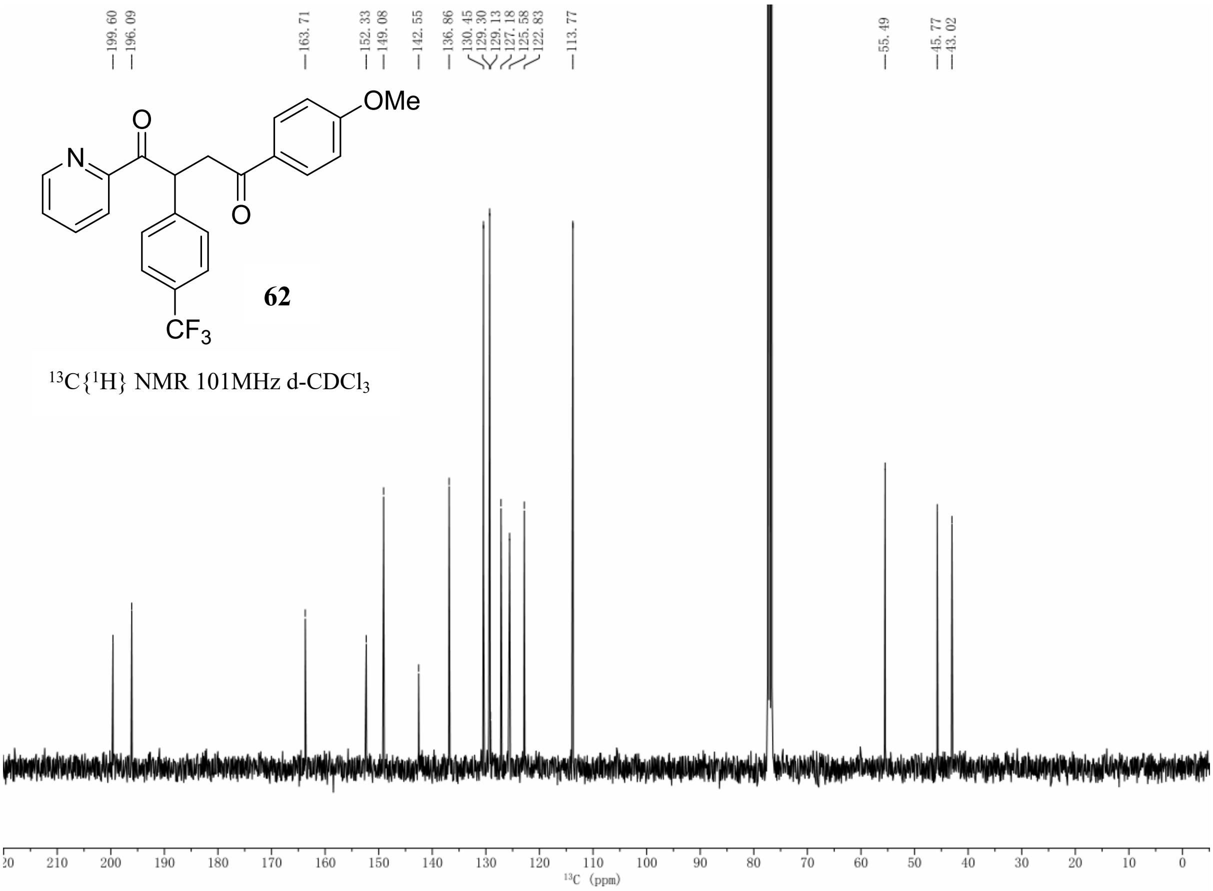
199.60
196.09
163.71
152.33
149.08
142.55
136.86
130.45
129.30
129.13
127.18
125.58
122.83
113.77
55.49
45.77
43.02
OMe
O
N
O
CF3
62
$^{13}C\{^{1}H\}$ NMR 101MHz d-CDCl3
13C (ppm)

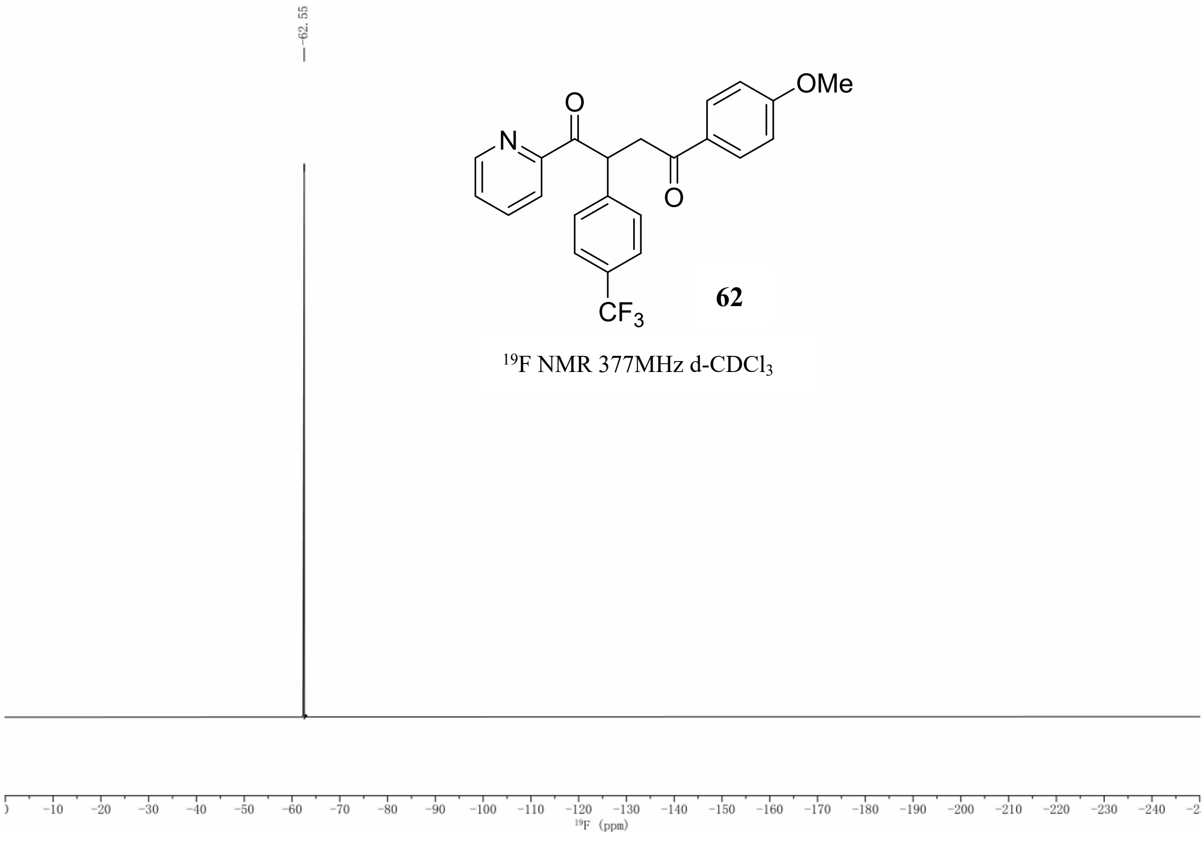
-62.55
OMe
O
N
O
CF3
62
^{19}F NMR 377MHz d-CDCl3
19F (ppm)

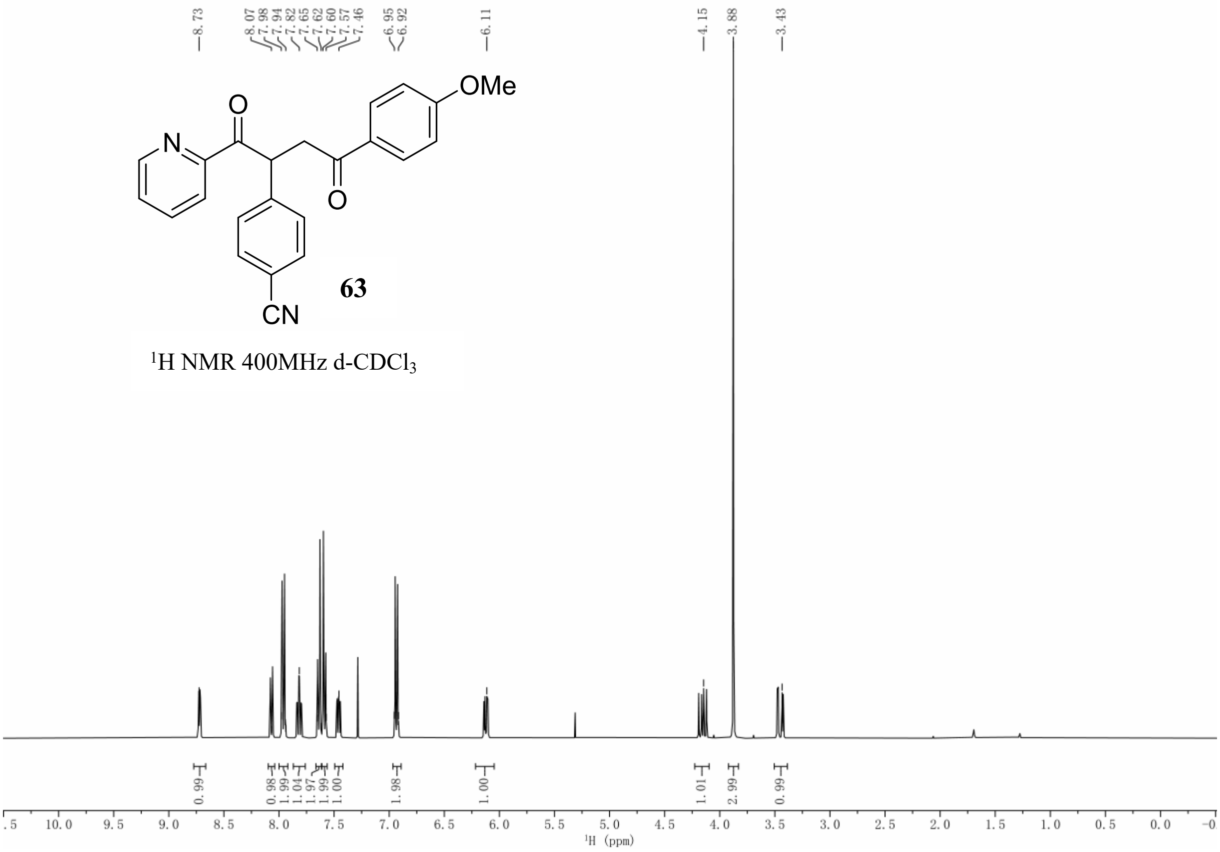
8.73
8.07
7.98
7.94
7.82
7.65
7.62
7.60
7.57
7.46
6.95
6.92
6.11
4.15
3.88
3.43
OMe
O
N
O
CN
63
1H NMR 400MHz d-CDCl3
0.99
0.98
1.99
1.04
1.97
1.99
1.00
1.98
1.00
1.01
2.99
0.99
1H (ppm)

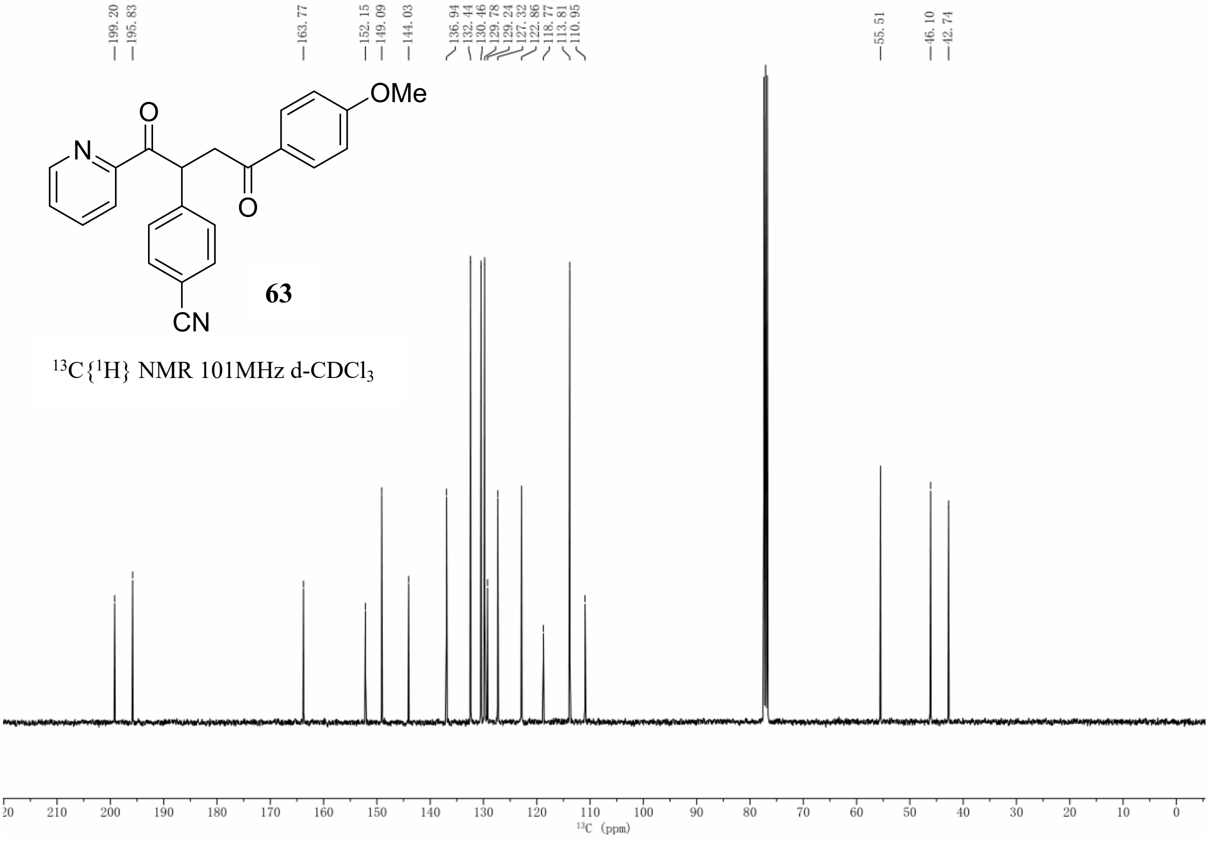
199.20
195.83
163.77
152.15
149.09
144.03
136.94
132.44
130.46
129.78
129.24
127.32
122.86
118.77
113.81
110.95
55.51
46.10
42.74
OMe
O
N
O
CN
63
13C{1H} NMR 101MHz d-CDCl3
13C (ppm)

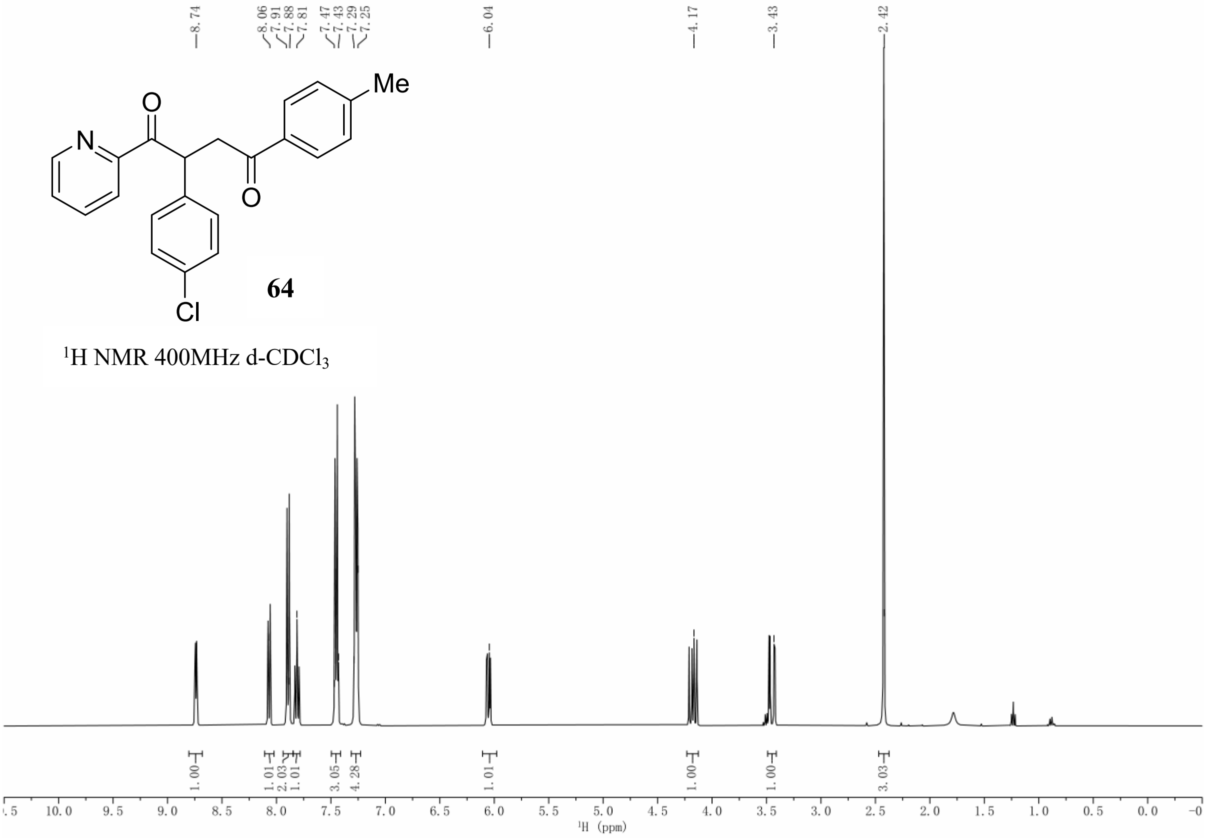
8.74
8.06
7.91
7.88
7.81
7.47
7.43
7.29
7.25
6.04
4.17
3.43
2.42
Me
O
N
O
Cl
64
^{1}H NMR 400MHz d-CDCl3
1.00
1.01
2.03
1.01
3.05
4.28
1.01
1.00
1.00
3.03
[1]H (ppm)

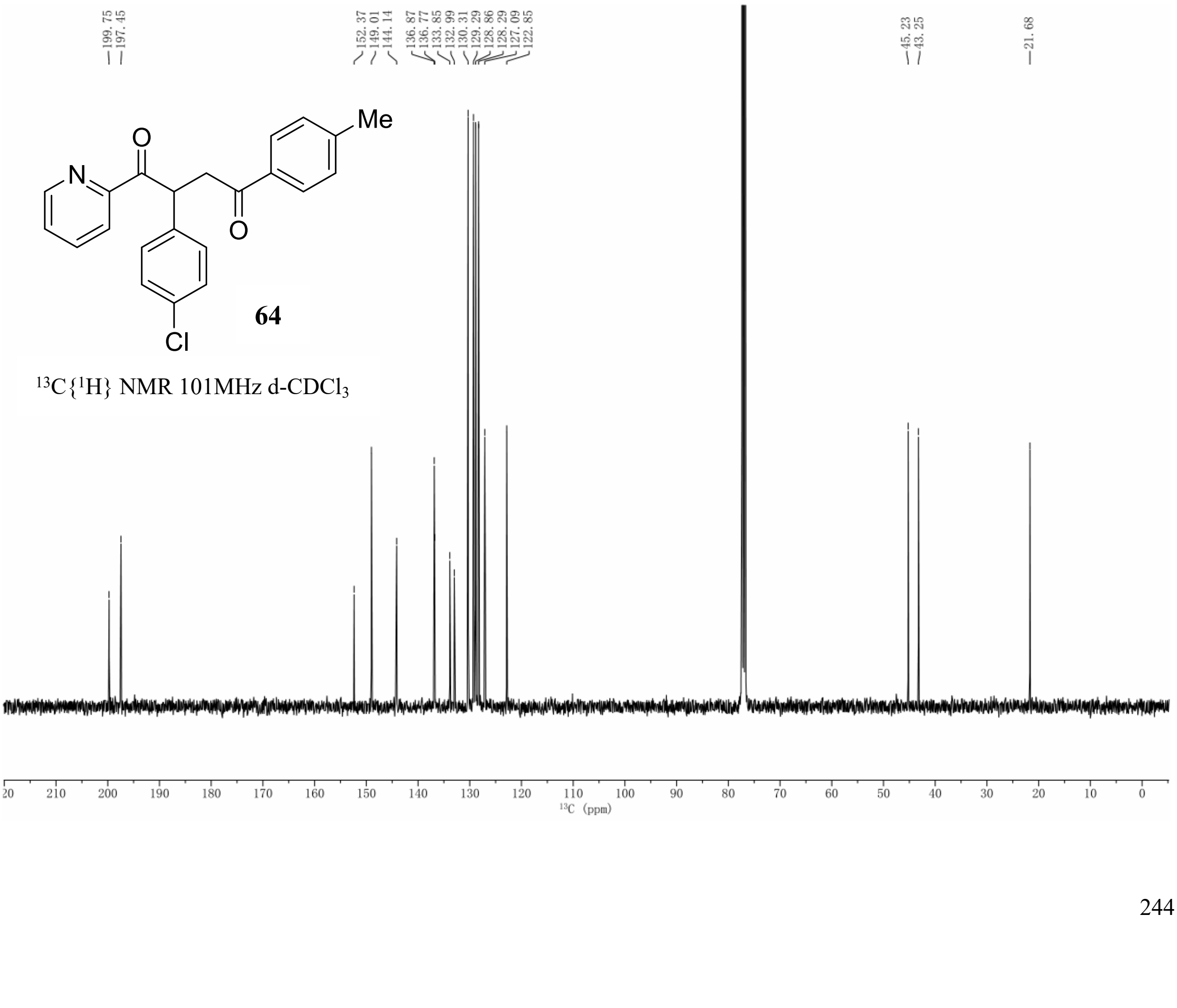
199.75
197.45
152.37
149.01
144.14
136.87
136.77
133.85
132.99
130.31
129.29
128.86
128.29
127.09
122.85
45.23
43.25
21.68
Me
O
N
O
Cl
64
^{13}C{^{1}H} NMR 101MHz d-CDCl3
[13]C (ppm)

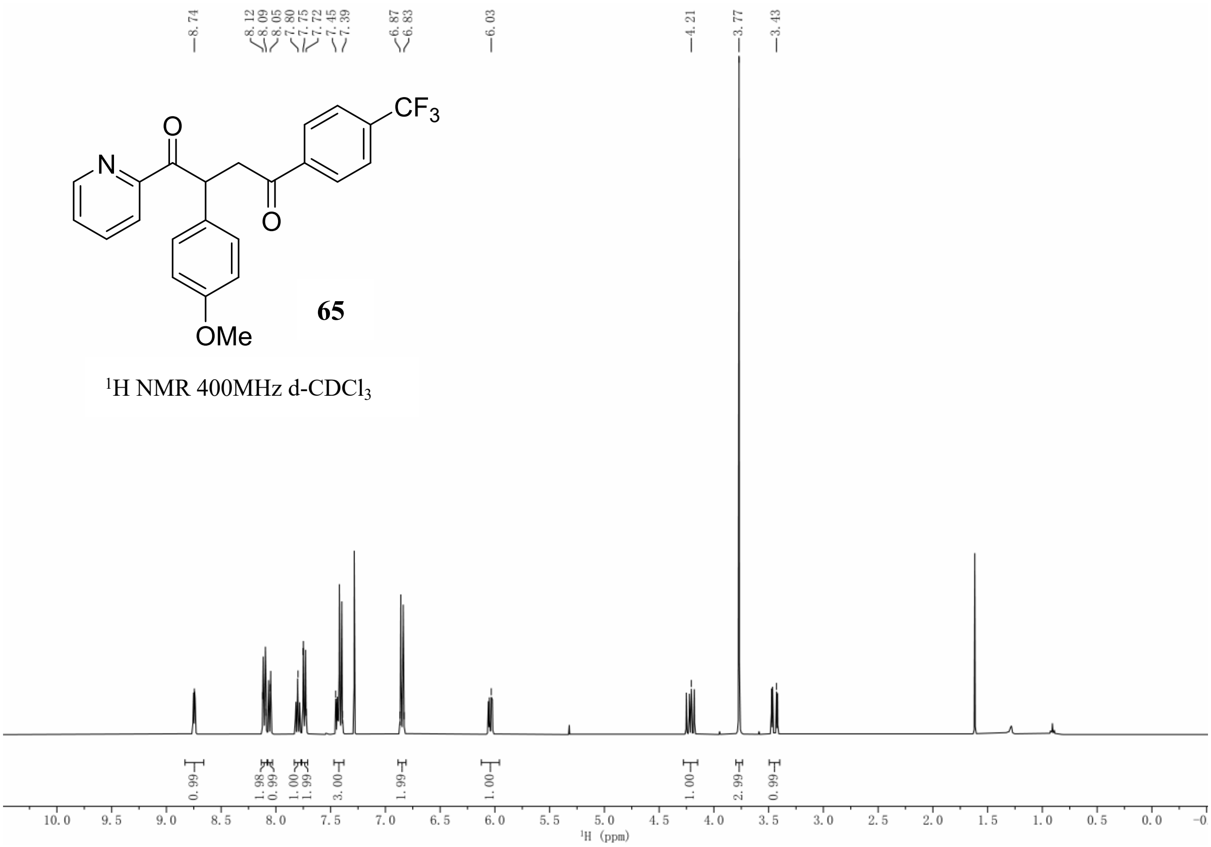
8.74
8.12
8.09
8.05
7.80
7.75
7.72
7.45
7.39
6.87
6.83
6.03
4.21
3.77
3.43
CF3
O
N
O
OMe
65
1H NMR 400MHz d-CDCl3
0.99
1.98
0.99
1.00
1.99
3.00
1.99
1.00
1.00
2.99
0.99
1H (ppm)

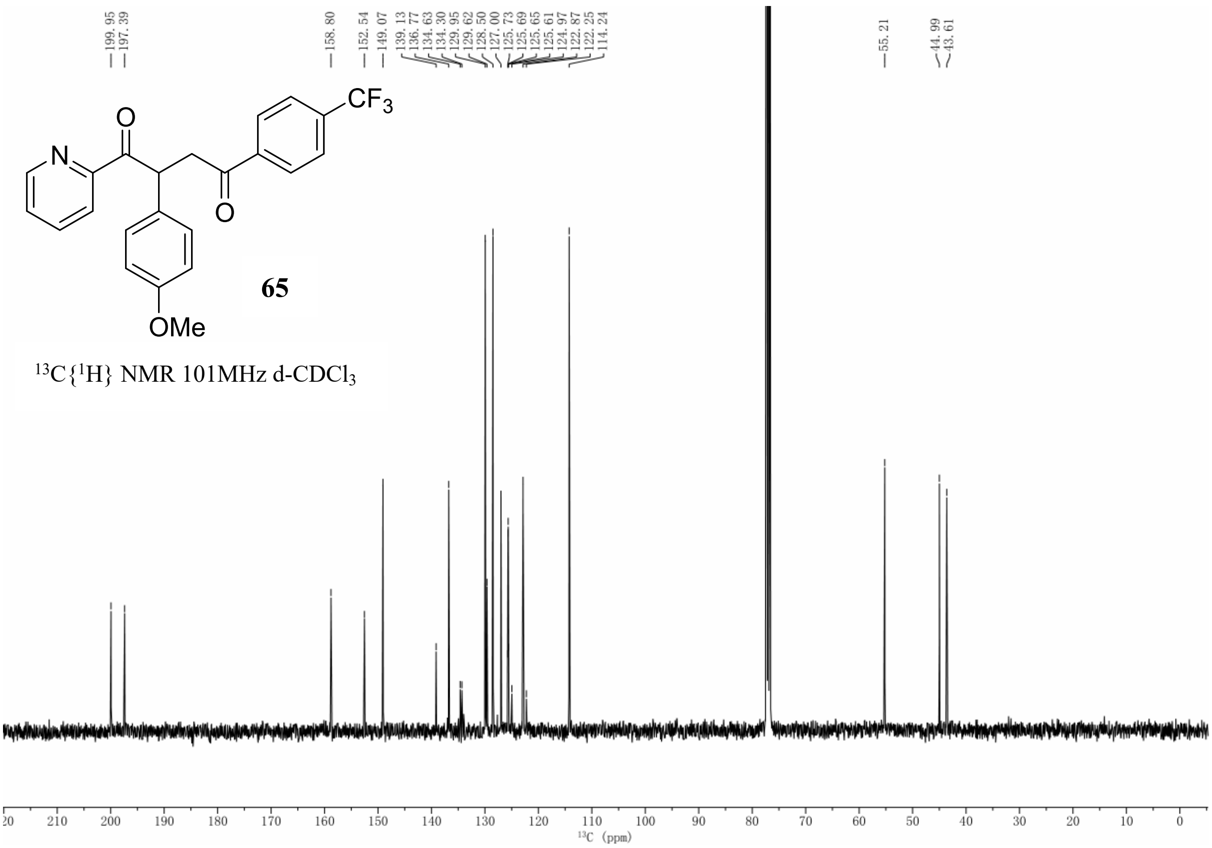
199.95
197.39
158.80
152.54
149.07
139.13
136.77
134.63
134.30
129.95
129.62
128.50
127.00
125.73
125.69
125.65
125.61
124.97
122.87
122.25
114.24
55.21
44.99
43.61
CF3
O
N
O
OMe
65
13C{1H} NMR 101MHz d-CDCl3
13C (ppm)

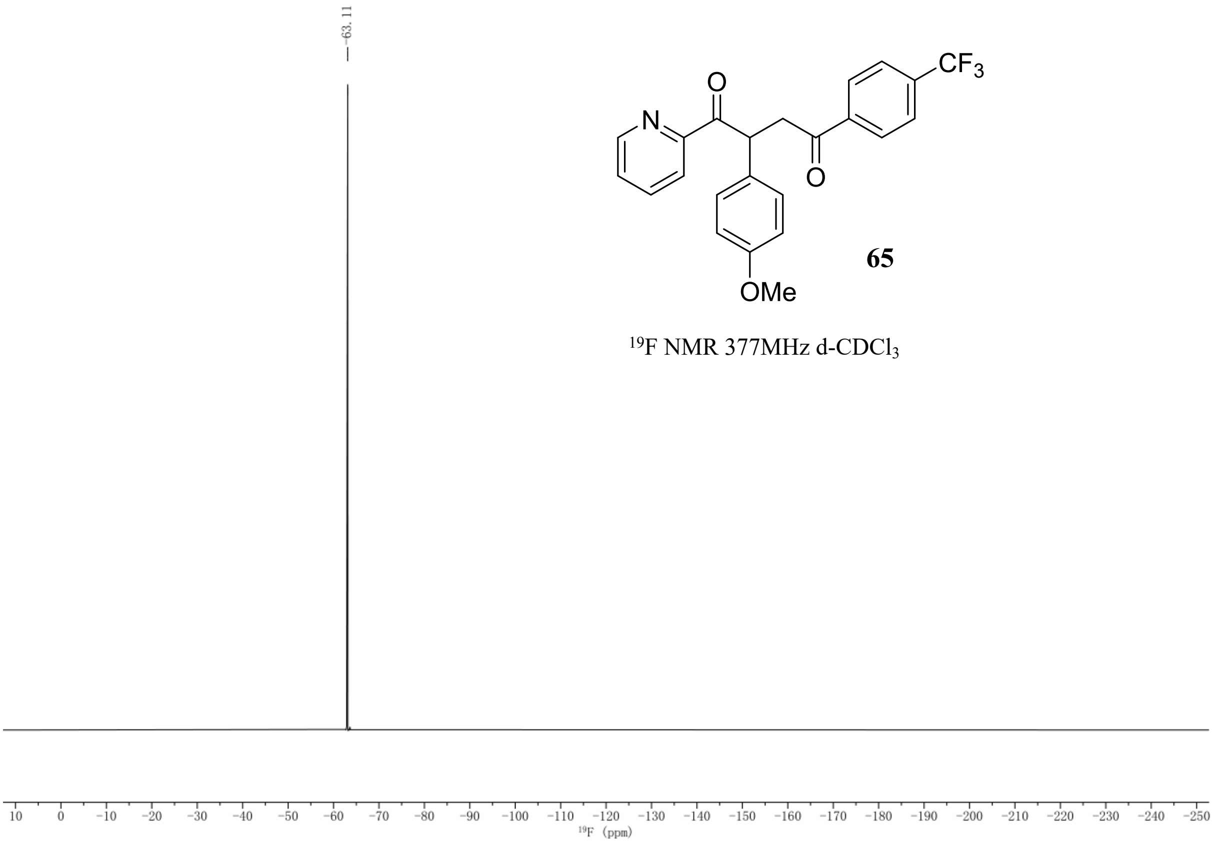

−63.11
CF3
O
N
O
OMe
65
19F NMR 377MHz d-CDCl3
10 0 −10 −20 −30 −40 −50 −60 −70 −80 −90 −100 −110 −120 −130 −140 −150 −160 −170 −180 −190 −200 −210 −220 −230 −240 −250
19F (ppm)

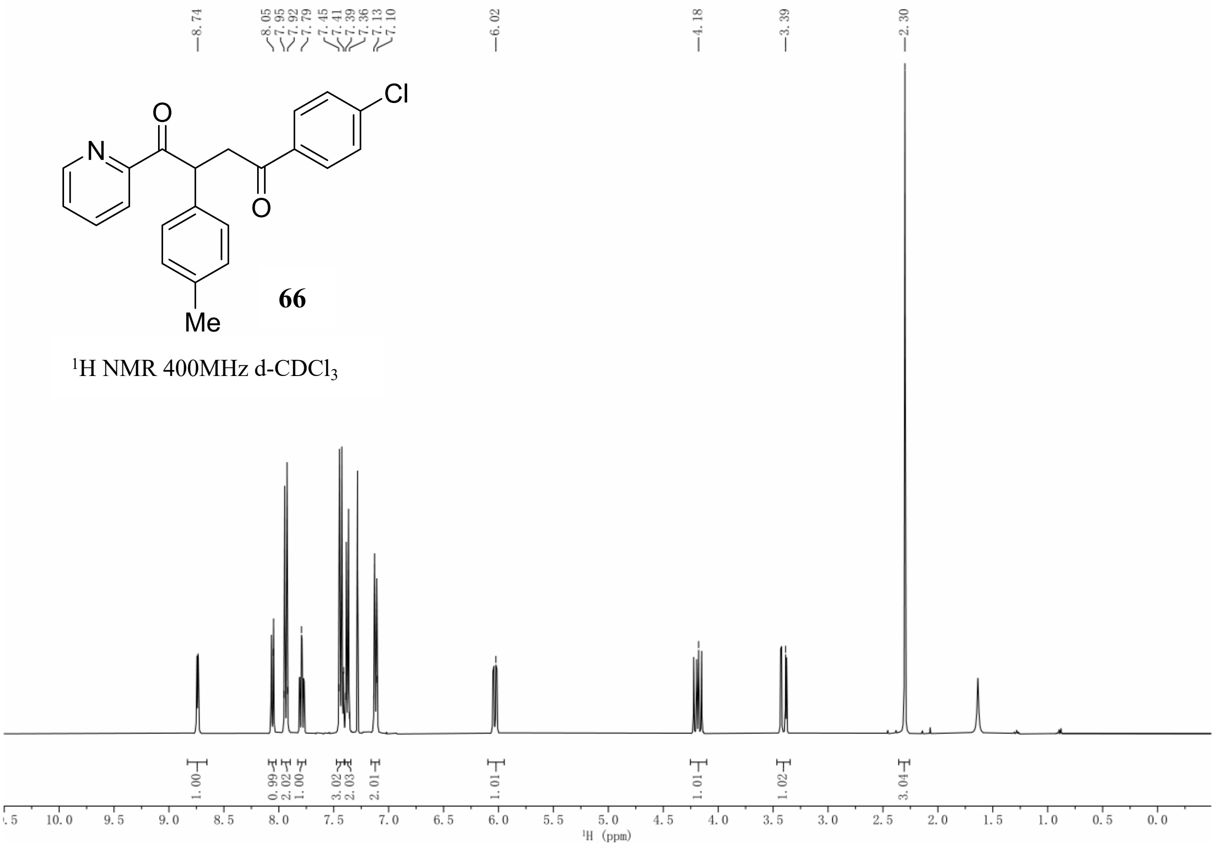

8.74
8.05
7.95
7.92
7.79
7.45
7.41
7.39
7.36
7.13
7.10
6.02
4.18
3.39
2.30
Cl
O
N
O
Me
66
1H NMR 400MHz d-CDCl3
1.00
0.99
2.02
1.00
3.02
2.03
2.01
1.01
1.01
1.02
3.04
10.0 9.5 9.0 8.5 8.0 7.5 7.0 6.5 6.0 5.5 5.0 4.5 4.0 3.5 3.0 2.5 2.0 1.5 1.0 0.5 0.0
1H (ppm)

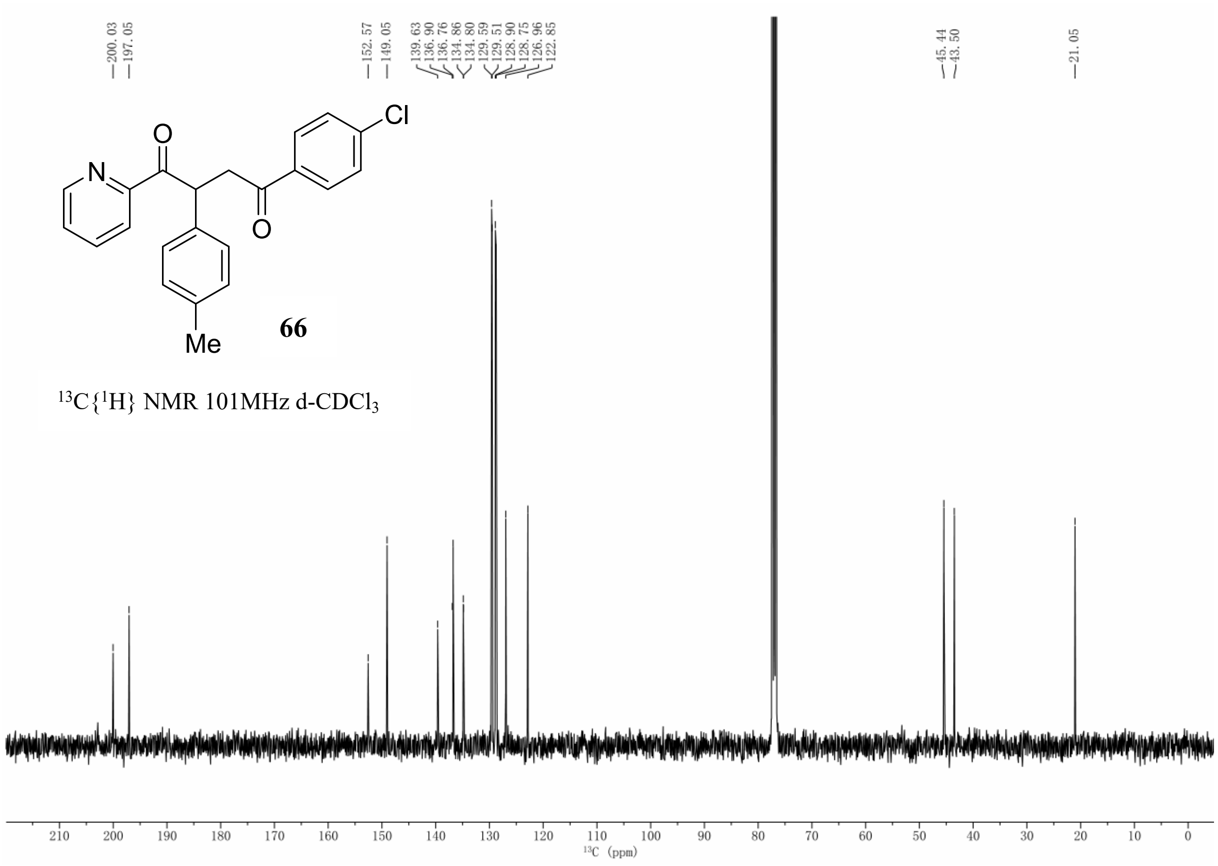
200.03
197.05
152.57
149.05
139.63
136.90
136.76
134.86
134.80
129.59
129.51
128.90
128.75
126.96
122.85
45.44
43.50
21.05
O
N
Cl
O
Me
66
$^{13}C\{^1H\}$ NMR 101MHz d-CDCl3
210 200 190 180 170 160 150 140 130 120 110 100 90 80 70 60 50 40 30 20 10 0
13C (ppm)

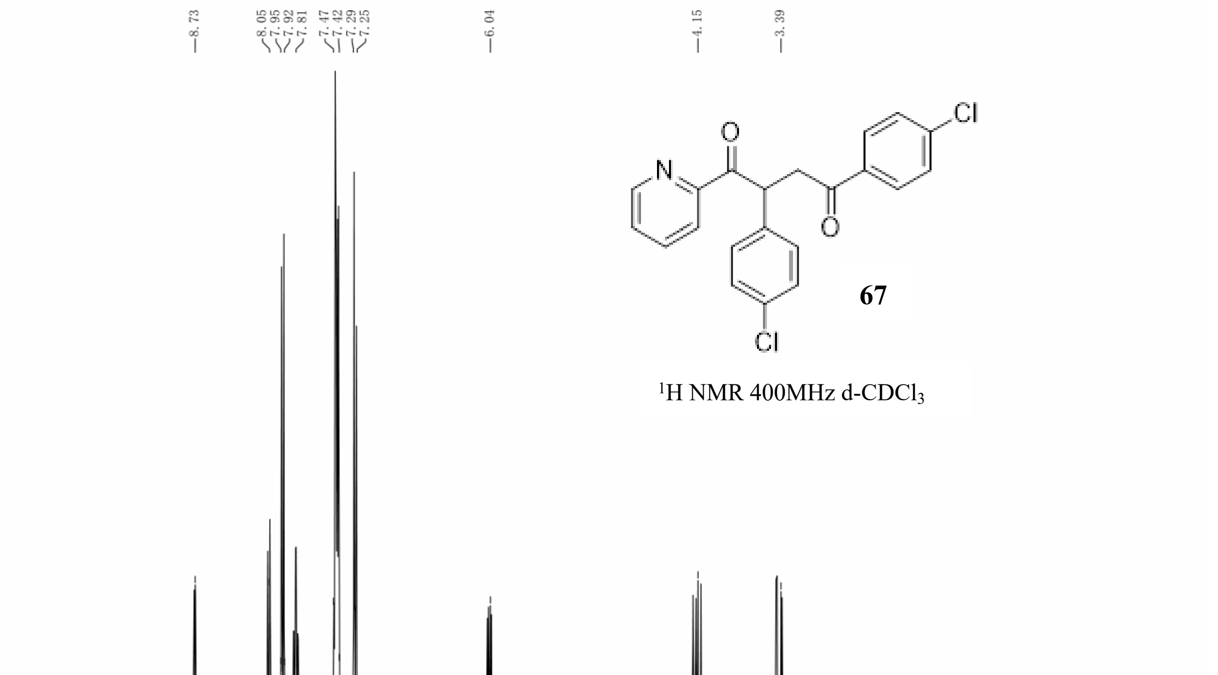
8.73
8.05
7.95
7.92
7.81
7.47
7.42
7.29
7.25
6.04
4.15
3.39
O
N
Cl
O
67
Cl
^1H NMR 400MHz d-CDCl3
1.00
1.00
2.01
2.01
1.01
5.05
2.22
1.01
1.01
0.99
10.0 9.5 9.0 8.5 8.0 7.5 7.0 6.5 6.0 5.5 5.0 4.5 4.0 3.5 3.0 2.5 2.0 1.5 1.0 0.5 0.0
1H (ppm)

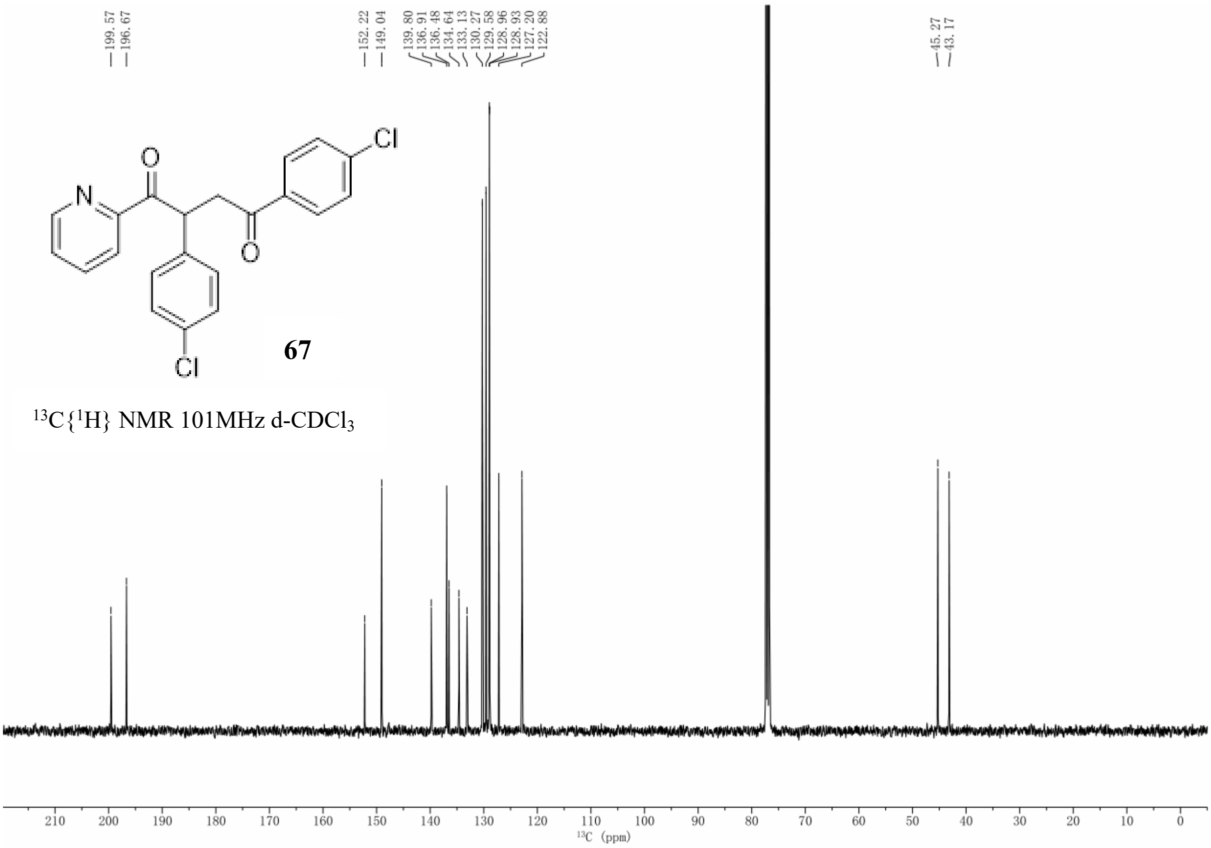
199.57
196.67
152.22
149.04
139.80
136.91
136.48
134.64
133.13
130.27
129.58
128.96
128.93
127.20
122.88
45.27
43.17
Cl
N
O
O
Cl
67
$^{13}C\{^1H\}$ NMR 101MHz d-CDCl$_3$
210 200 190 180 170 160 150 140 130 120 110 100 90 80 70 60 50 40 30 20 10 0
^{13}C (ppm)

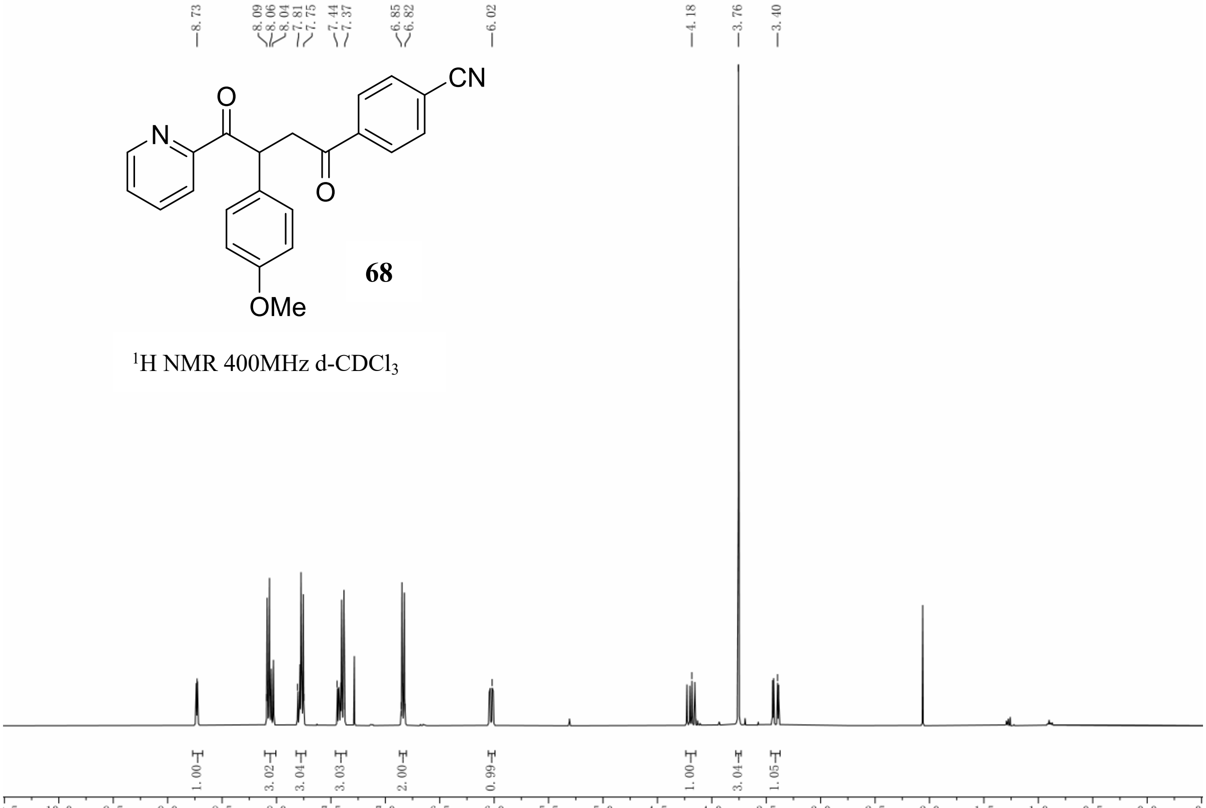
8.73
8.09
8.06
8.04
7.81
7.75
7.44
7.37
6.85
6.82
6.02
4.18
3.76
3.40
CN
O
N
O
68
OMe
^{1}H NMR 400MHz d-CDCl$_3$
1.00
3.02
3.04
3.03
2.00
0.99
1.00
3.04
1.05
10.0 9.5 9.0 8.5 8.0 7.5 7.0 6.5 6.0 5.5 5.0 4.5 4.0 3.5 3.0 2.5 2.0 1.5 1.0 0.5 0.0
^{1}H (ppm)

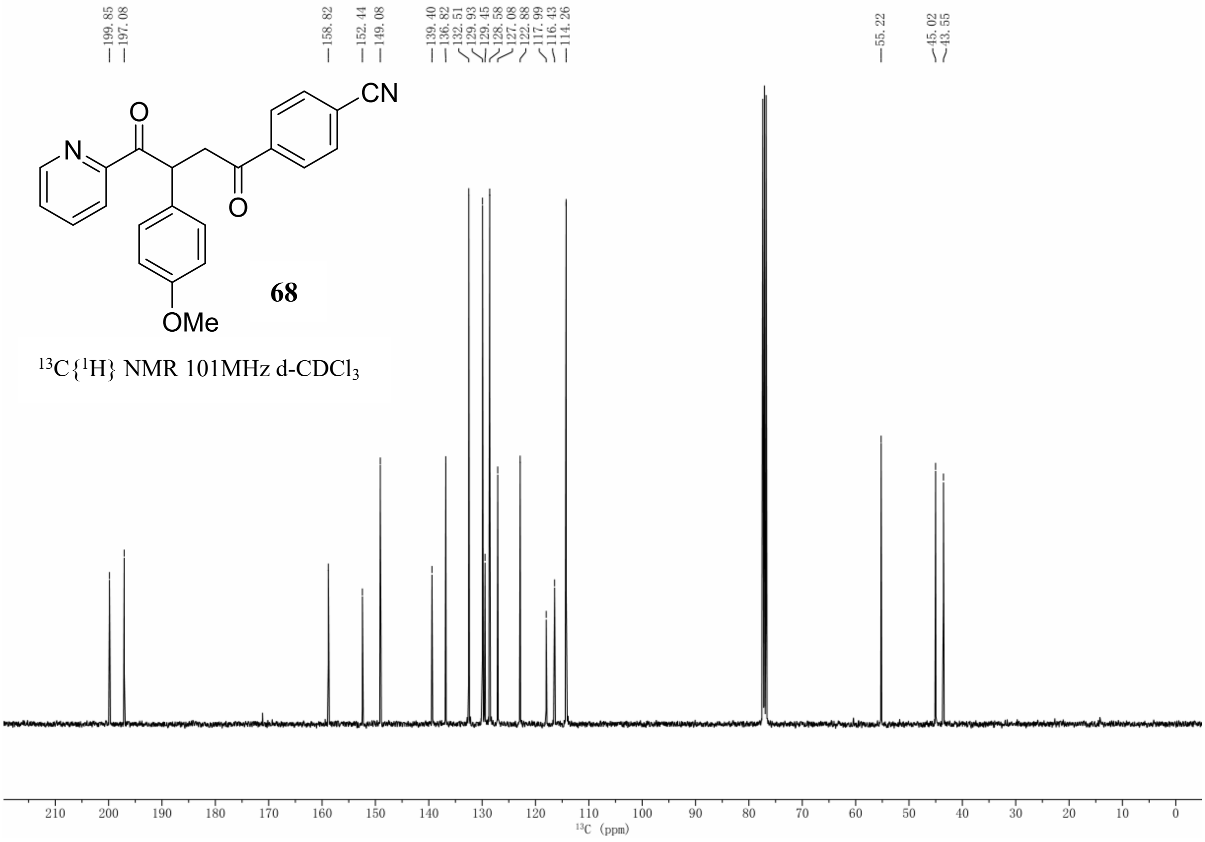

199.85
197.08
158.82
152.44
149.08
139.40
136.82
132.51
129.93
129.45
128.58
127.08
122.88
117.99
116.43
114.26
55.22
45.02
43.55
CN
N
O
O
OMe
68
$^{13}C\{^1H\}$ NMR 101MHz d-$CDCl_3$
210 200 190 180 170 160 150 140 130 120 110 100 90 80 70 60 50 40 30 20 10 0
^{13}C (ppm)

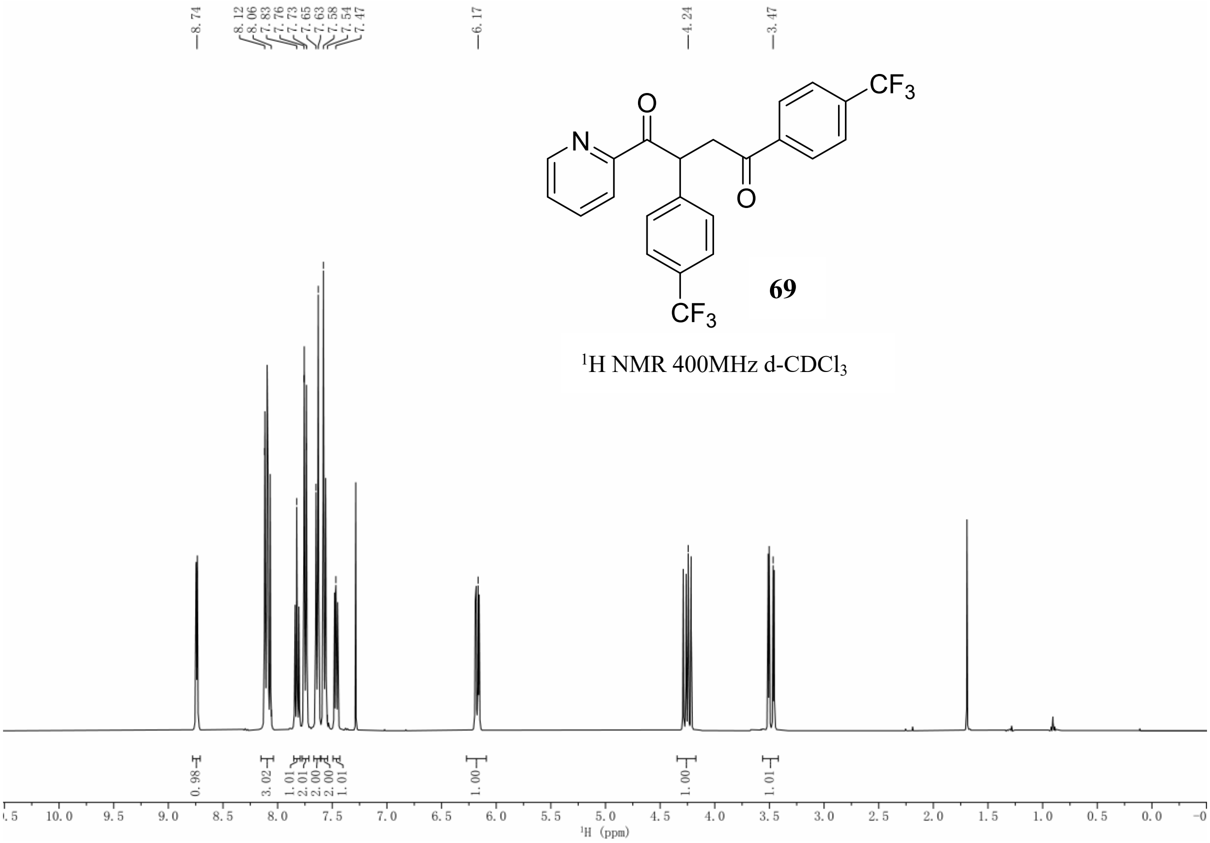

8.74
8.12
8.06
7.83
7.76
7.73
7.65
7.63
7.58
7.54
7.47
6.17
4.24
3.47
CF_3
N
O
O
CF_3
69
^{1}H NMR 400MHz d-$CDCl_3$
0.98
3.02
1.01
2.01
2.00
2.00
1.01
1.00
1.00
1.01
10.0 9.5 9.0 8.5 8.0 7.5 7.0 6.5 6.0 5.5 5.0 4.5 4.0 3.5 3.0 2.5 2.0 1.5 1.0 0.5 0.0
^{1}H (ppm)

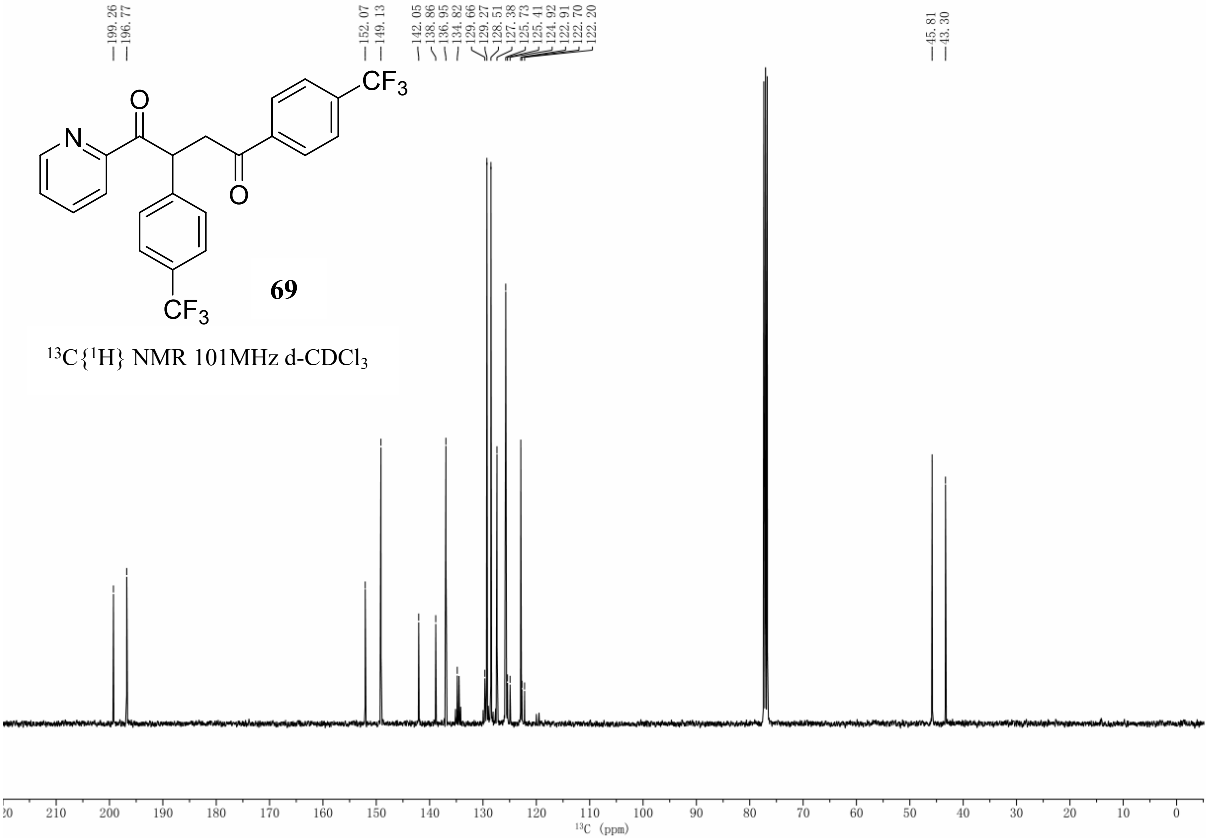
199.26
196.77
152.07
149.13
142.05
138.86
136.95
134.82
129.66
129.27
128.51
127.38
125.73
125.41
124.92
122.91
122.70
122.20
45.81
43.30
CF3
O
N
O
CF3
69
13C{1H} NMR 101MHz d-CDCl3
13C (ppm)

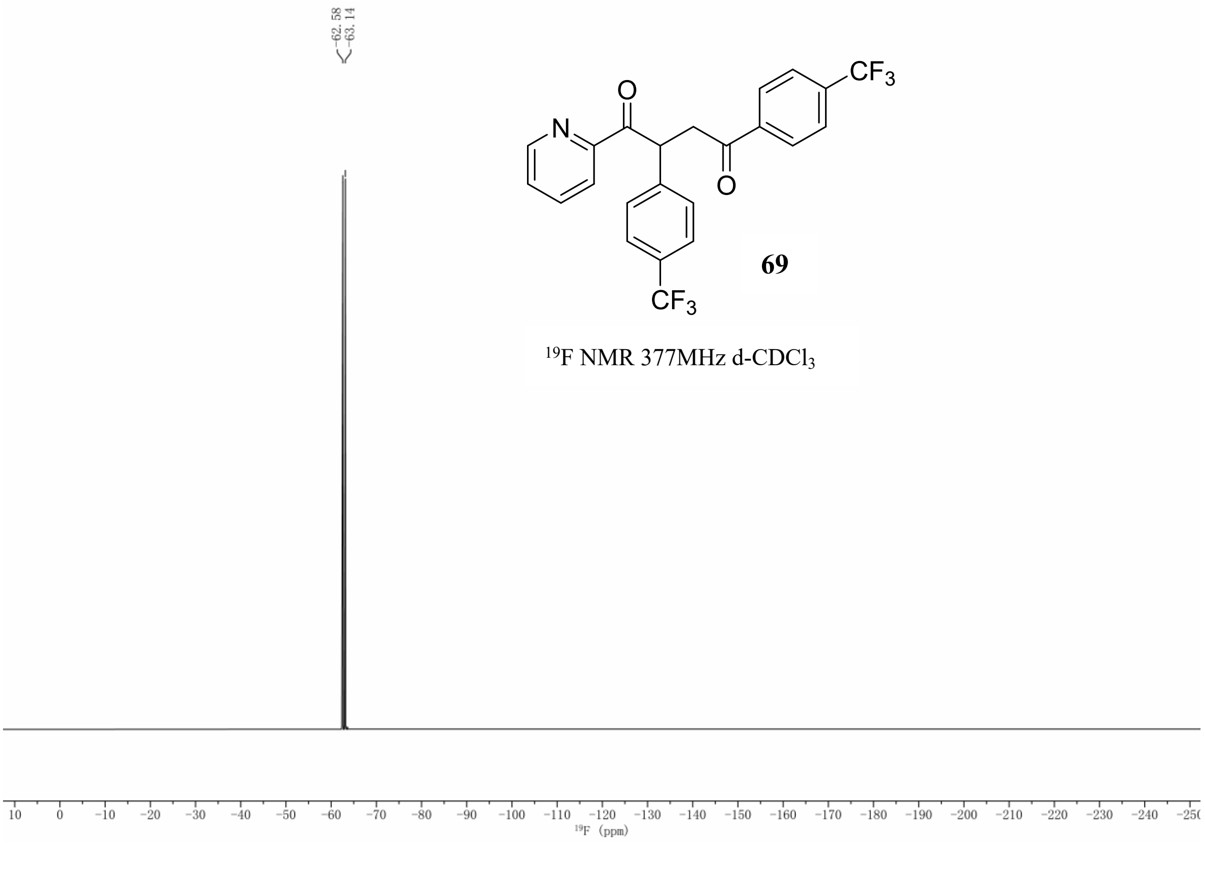
-62.58
-63.14
CF3
O
N
O
CF3
69
19F NMR 377MHz d-CDCl3
19F (ppm)

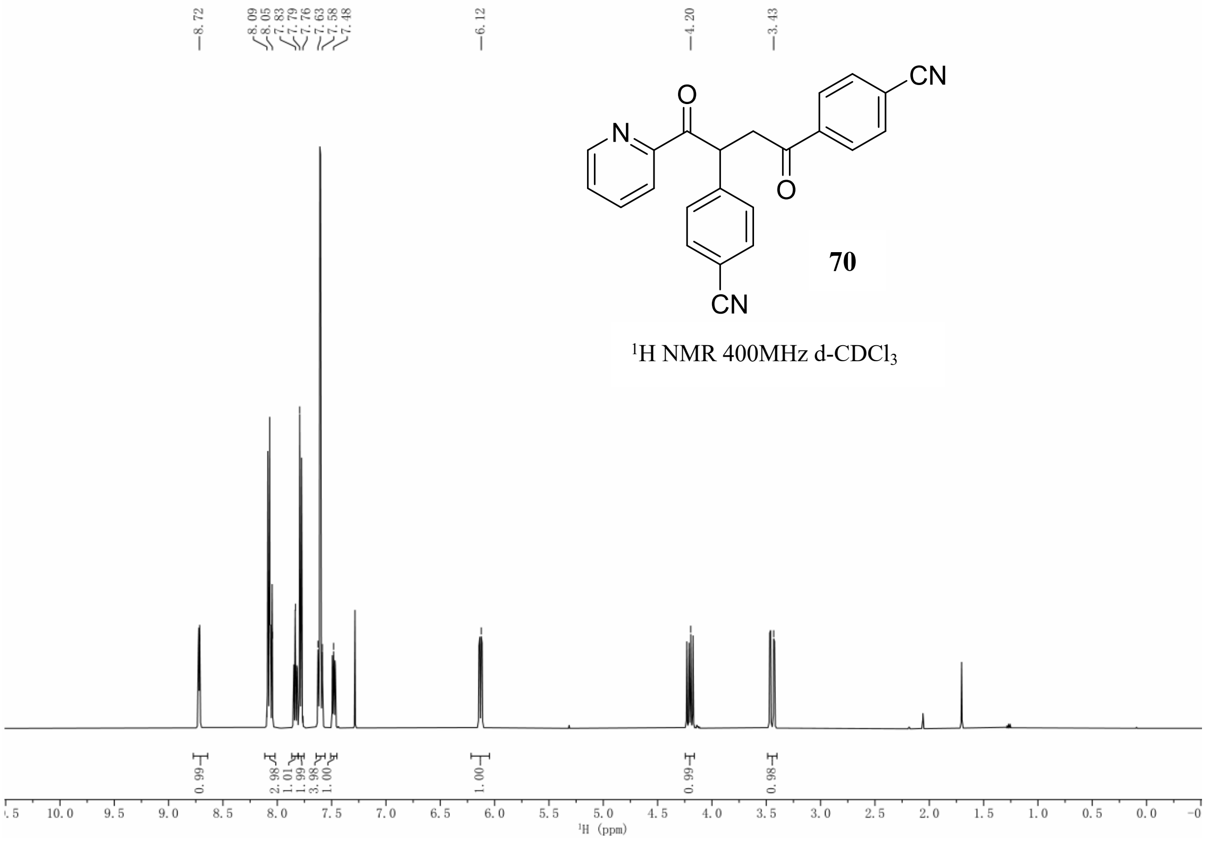
8.72
8.09
8.05
7.83
7.79
7.76
7.63
7.58
7.48
6.12
4.20
3.43
O
N
CN
O
CN
70
1H NMR 400MHz d-CDCl3
0.99
2.98
1.01
1.99
3.98
1.00
1.00
0.99
0.98
1H (ppm)

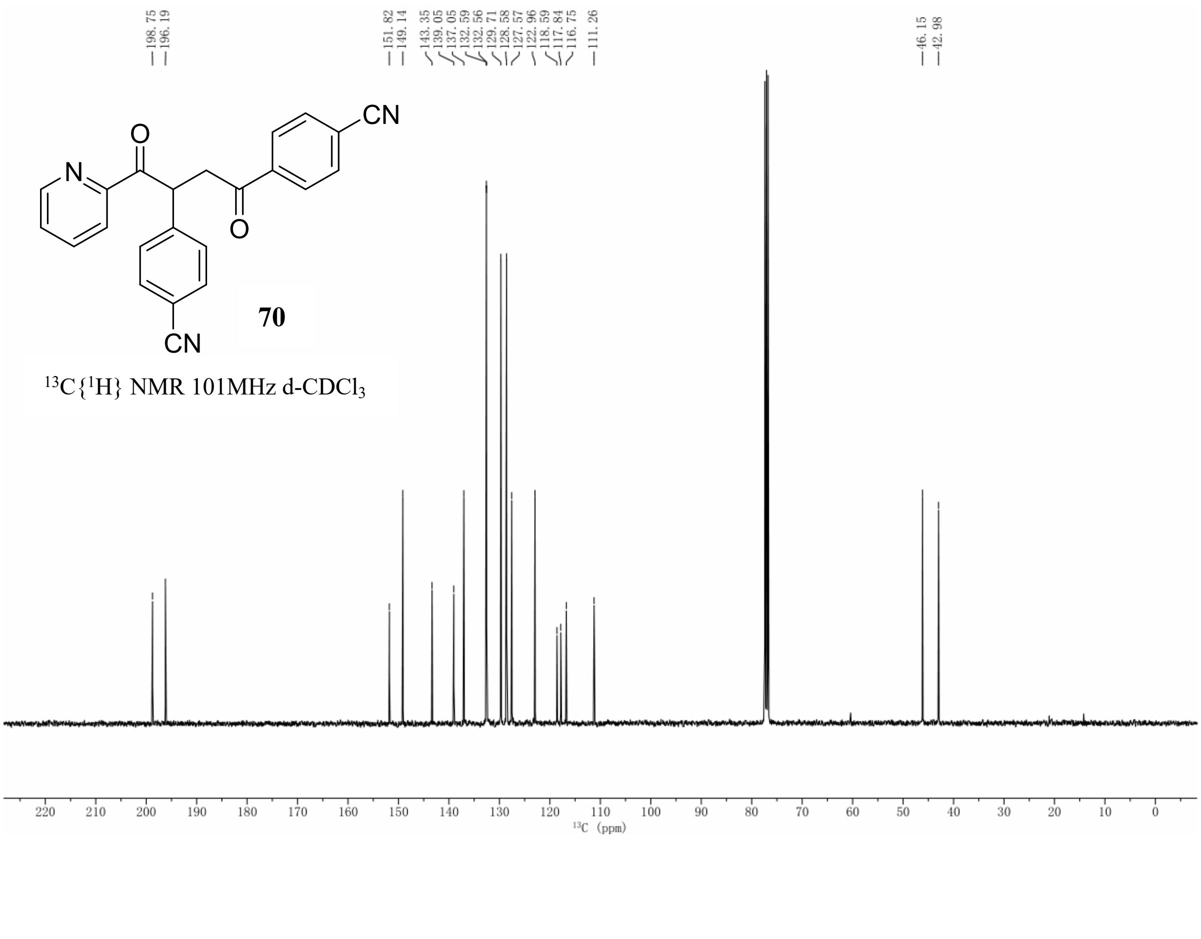
198.75
196.19
151.82
149.14
143.35
139.05
137.05
132.59
132.56
129.71
128.58
127.57
122.96
118.59
117.84
116.75
111.26
46.15
42.98
O
N
CN
O
CN
70
13C{1H} NMR 101MHz d-CDCl3
13C (ppm)

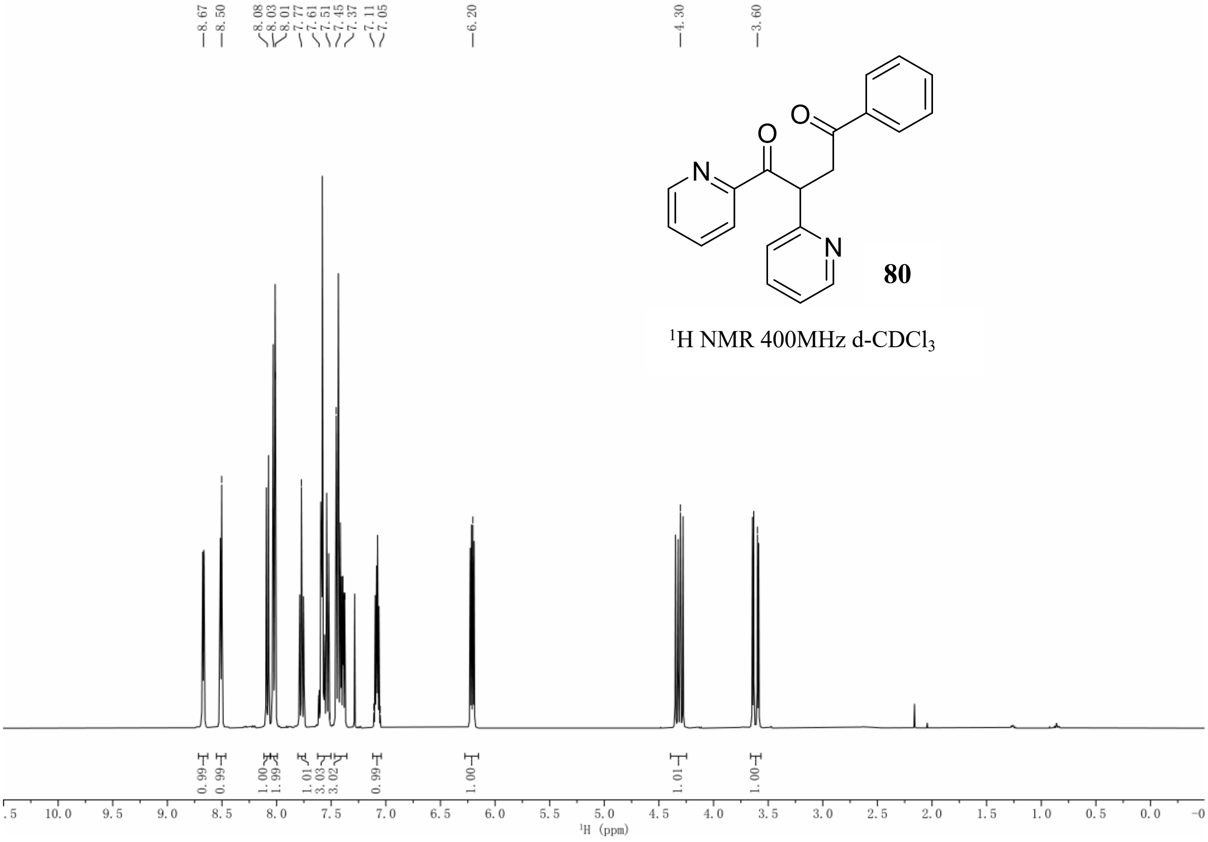
8.67
8.50
8.08
8.03
8.01
7.77
7.61
7.51
7.45
7.37
7.11
7.05
6.20
4.30
3.60
80
^{1}H NMR 400MHz d-CDCl$_3$
0.99
0.99
1.00
1.99
1.01
3.03
3.02
0.99
1.00
1.01
1.00
^{1}H (ppm)

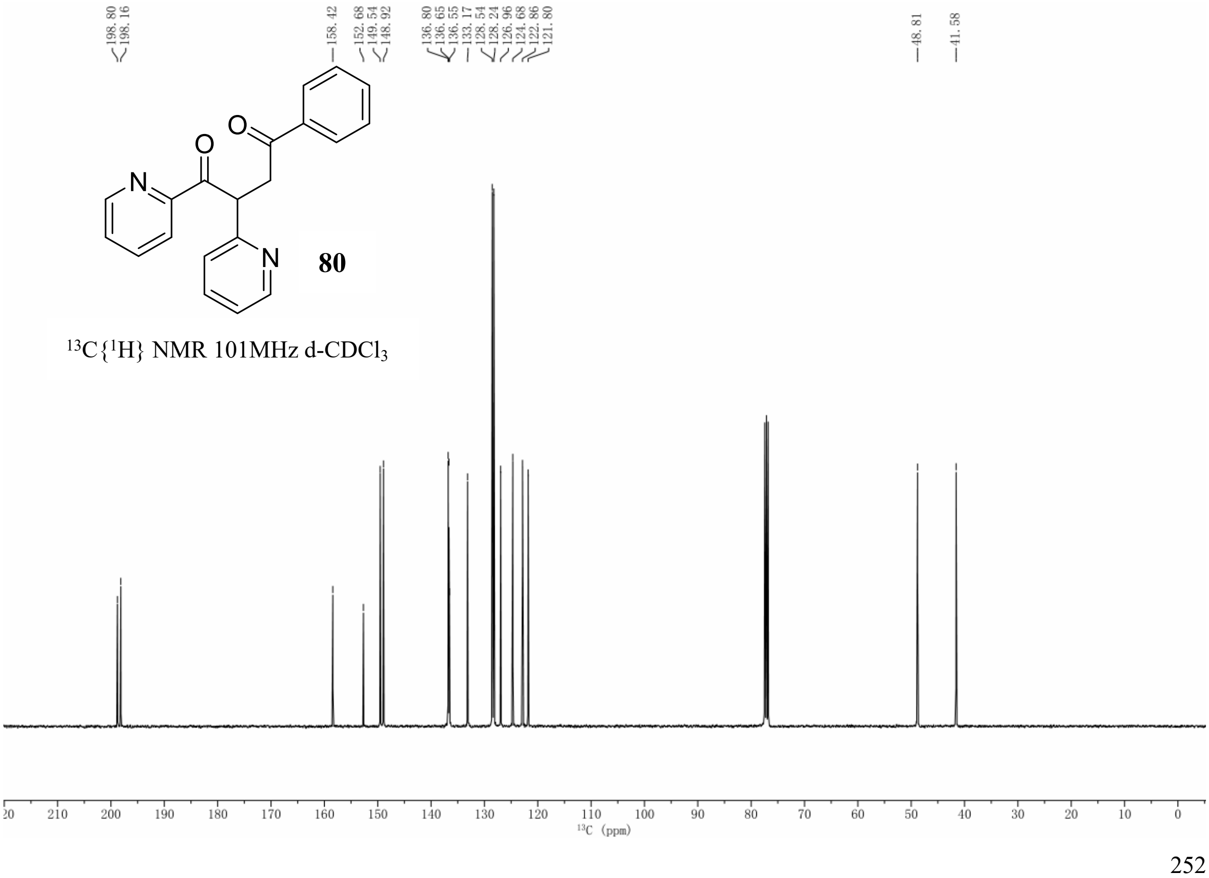
198.80
198.16
158.42
152.68
149.54
148.92
136.80
136.65
136.55
133.17
128.54
128.24
126.96
124.68
122.86
121.80
48.81
41.58
80
^{13}C{^{1}H} NMR 101MHz d-CDCl$_3$
^{13}C (ppm)

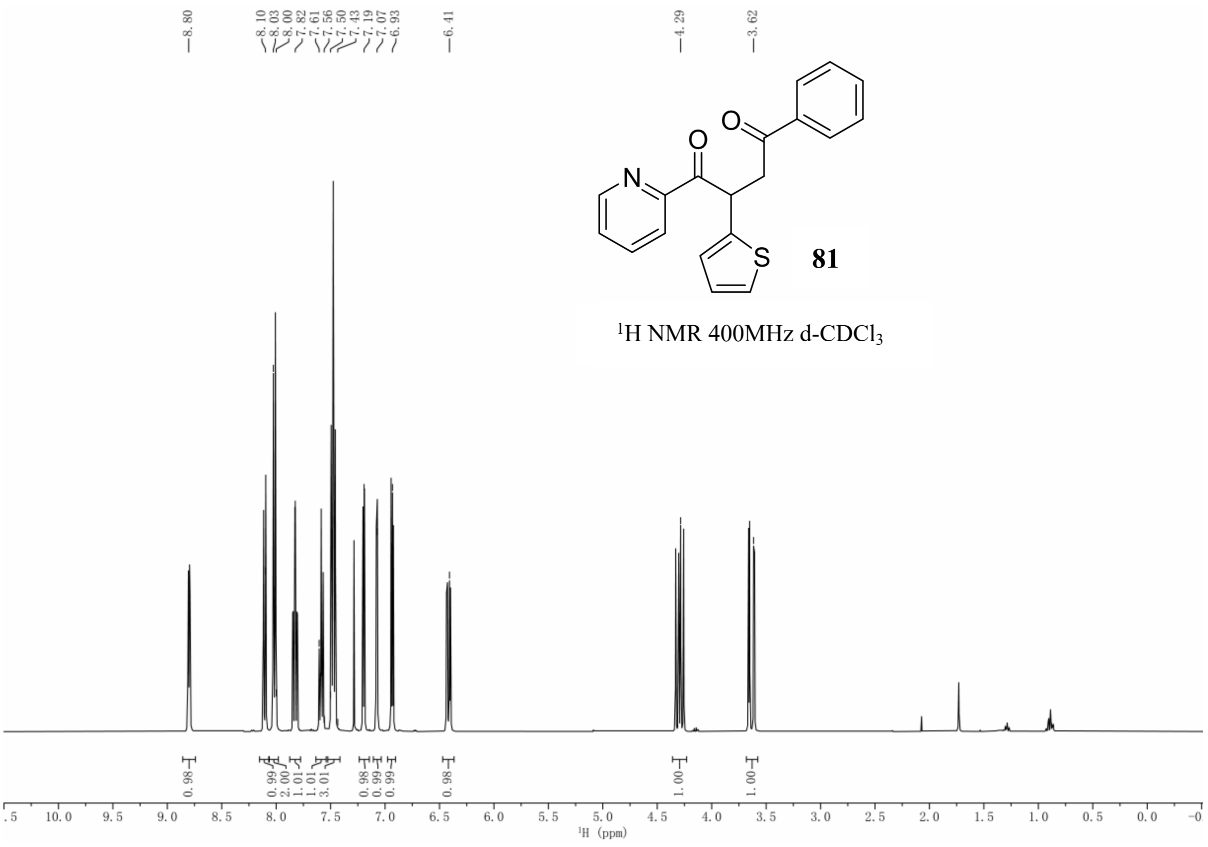

8.80
8.10 8.03 8.00 7.82 7.61 7.56 7.50 7.43 7.19 7.07 6.93
6.41
4.29
3.62
81
¹H NMR 400MHz d-CDCl₃
0.98 0.99 2.00 1.01 1.01 3.01 0.98 0.99 0.99 0.98 1.00 1.00
¹H (ppm)

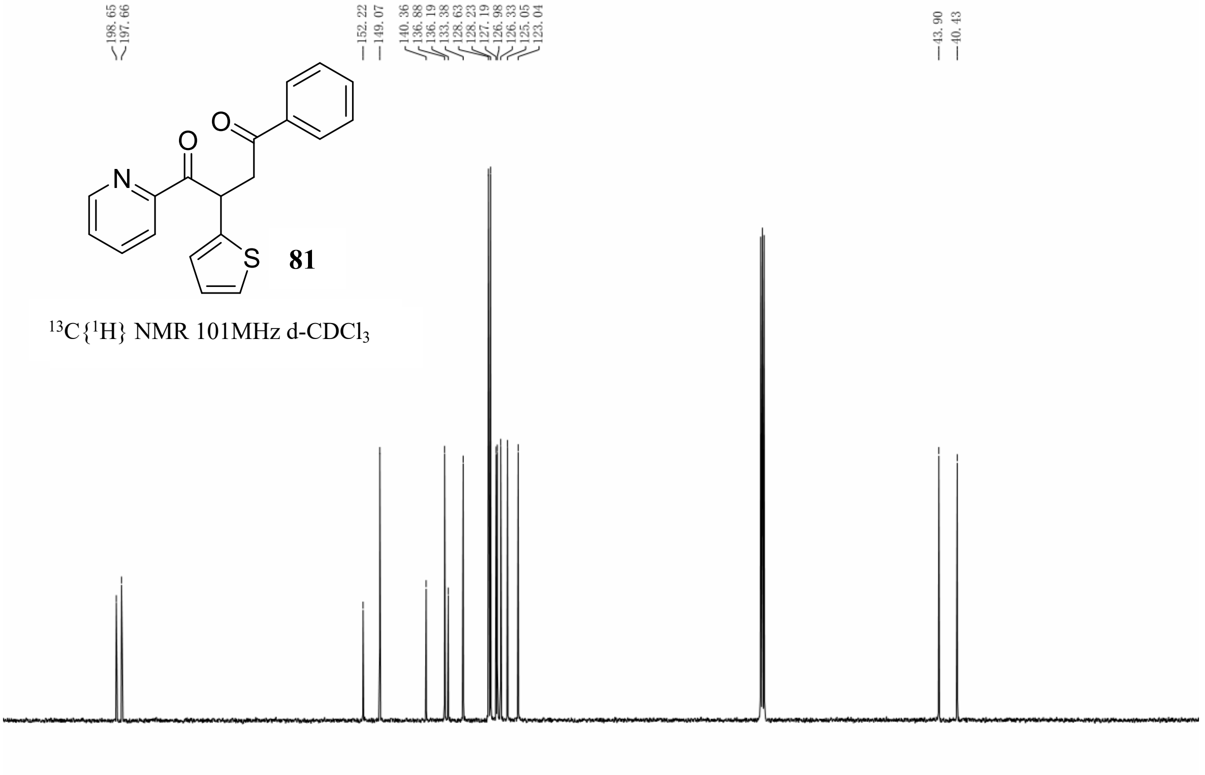

198.65 197.66
152.22 149.07
140.36 136.88 136.19 133.38 128.63 128.23 127.19 126.98 126.33 125.05 123.04
43.90 40.43
81
¹³C{¹H} NMR 101MHz d-CDCl₃
¹³C (ppm)

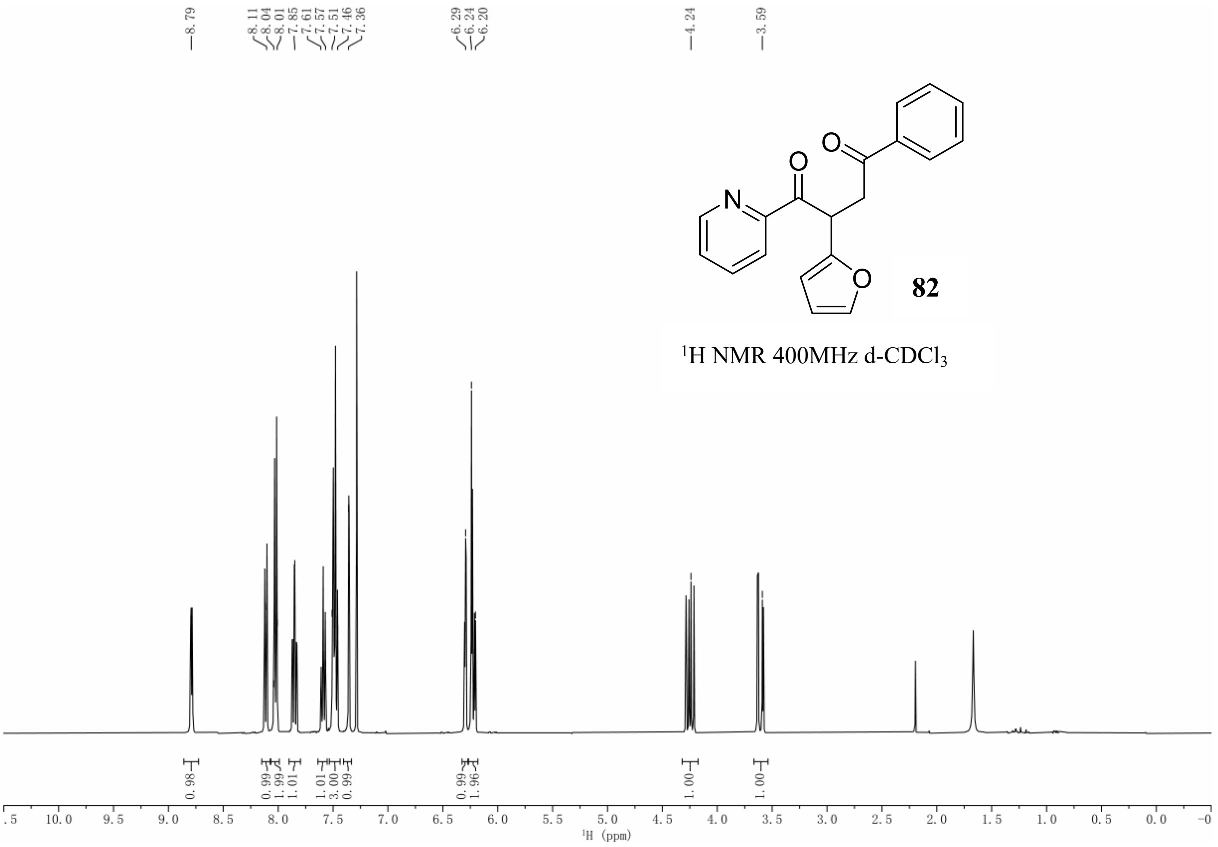
8.79
8.11 8.04 8.01 7.85 7.61 7.57 7.51 7.46 7.36
6.29 6.24 6.20
4.24
3.59
82
¹H NMR 400MHz d-CDCl₃
0.98 0.99 1.99 1.01 1.01 3.00 0.99 0.99 1.96 1.00 1.00
¹H (ppm)

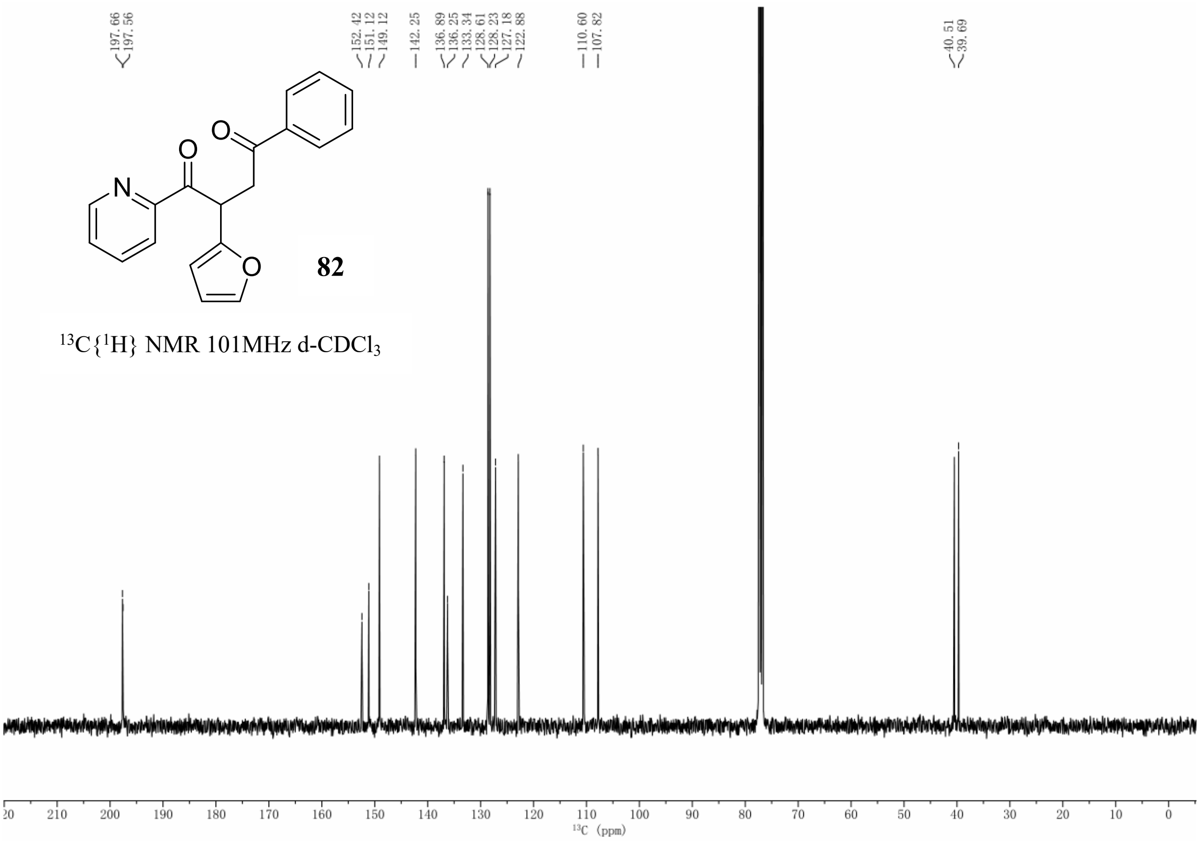
197.66 197.56
152.42 151.12 149.12
142.25
136.89 136.25 133.34 128.61 128.23 127.18 122.88
110.60 107.82
40.51 39.69
82
¹³C{¹H} NMR 101MHz d-CDCl₃
¹³C (ppm)

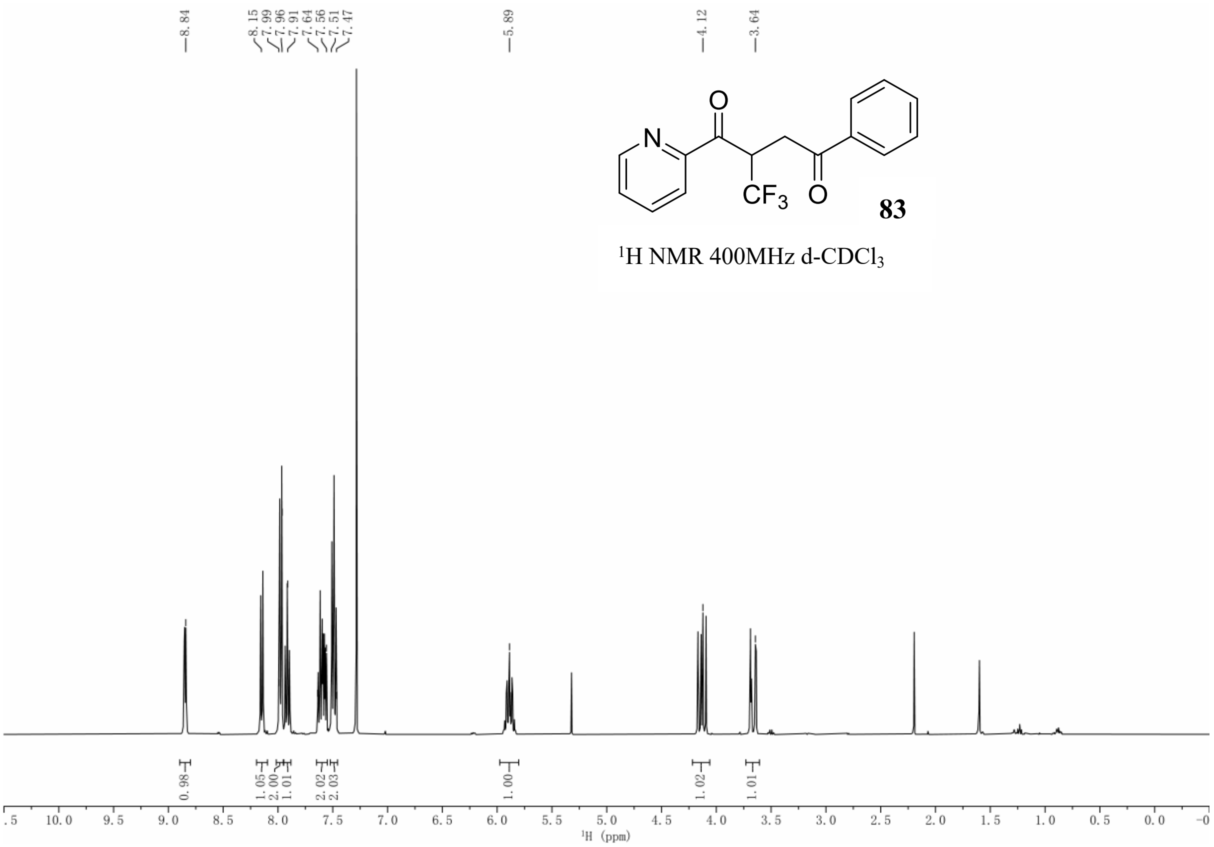
8.84
8.15
7.99
7.96
7.91
7.64
7.56
7.51
7.47
5.89
4.12
3.64
N
O
CF3
O
83
1H NMR 400MHz d-CDCl3
0.98
1.05
2.00
1.01
2.02
2.03
1.00
1.02
1.01
10.0
9.5
9.0
8.5
8.0
7.5
7.0
6.5
6.0
5.5
5.0
4.5
4.0
3.5
3.0
2.5
2.0
1.5
1.0
0.5
0.0
1H (ppm)

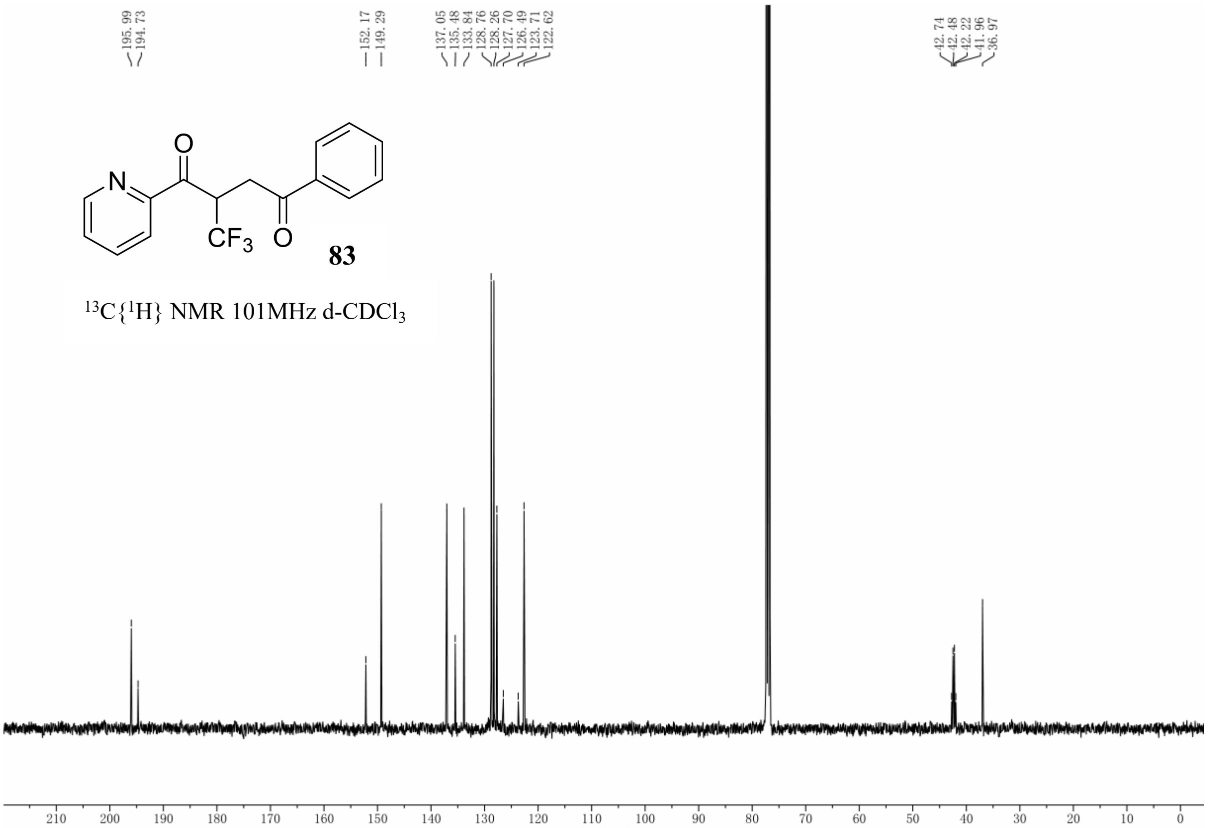
195.99
194.73
152.17
149.29
137.05
135.48
133.84
128.76
128.26
127.70
126.49
123.71
122.62
42.74
42.48
42.22
41.96
36.97
N
O
CF3
O
83
13C{1H} NMR 101MHz d-CDCl3
210
200
190
180
170
160
150
140
130
120
110
100
90
80
70
60
50
40
30
20
10
0
13C (ppm)

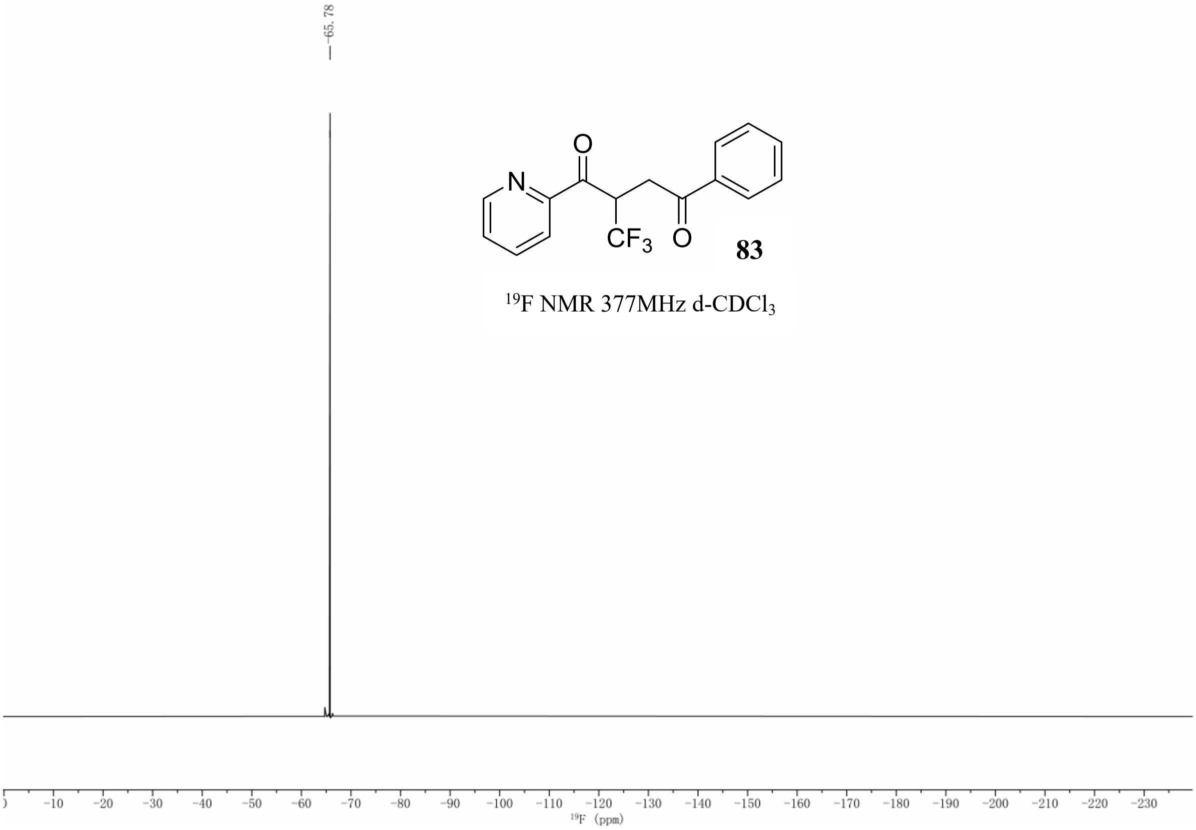

—65.78
83
^{19}F NMR 377MHz d-CDCl$_3$
^{19}F (ppm)

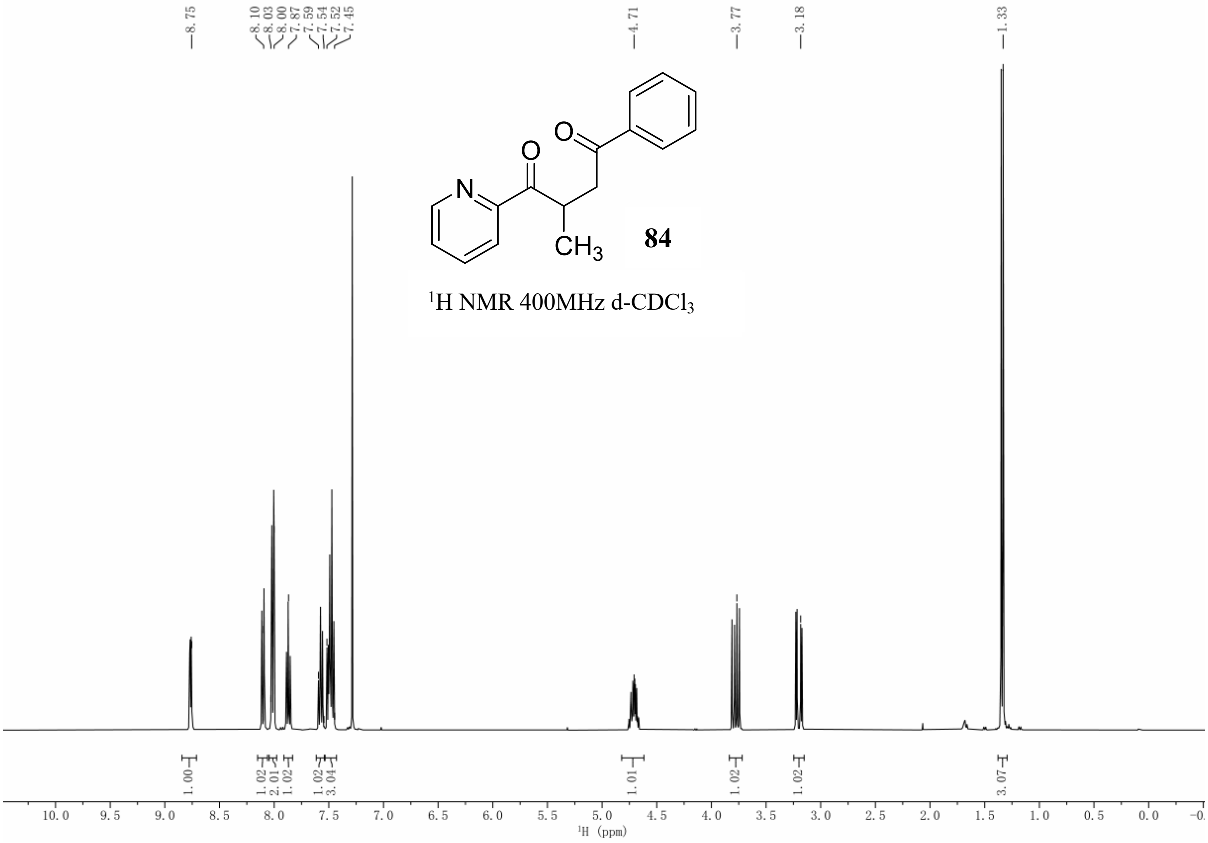

8.75
8.10
8.03
8.00
7.87
7.59
7.54
7.52
7.45
4.71
3.77
3.18
1.33
84
^{1}H NMR 400MHz d-CDCl$_3$
1.00
1.02
2.01
1.02
1.02
3.04
1.01
1.02
1.02
3.07
^{1}H (ppm)

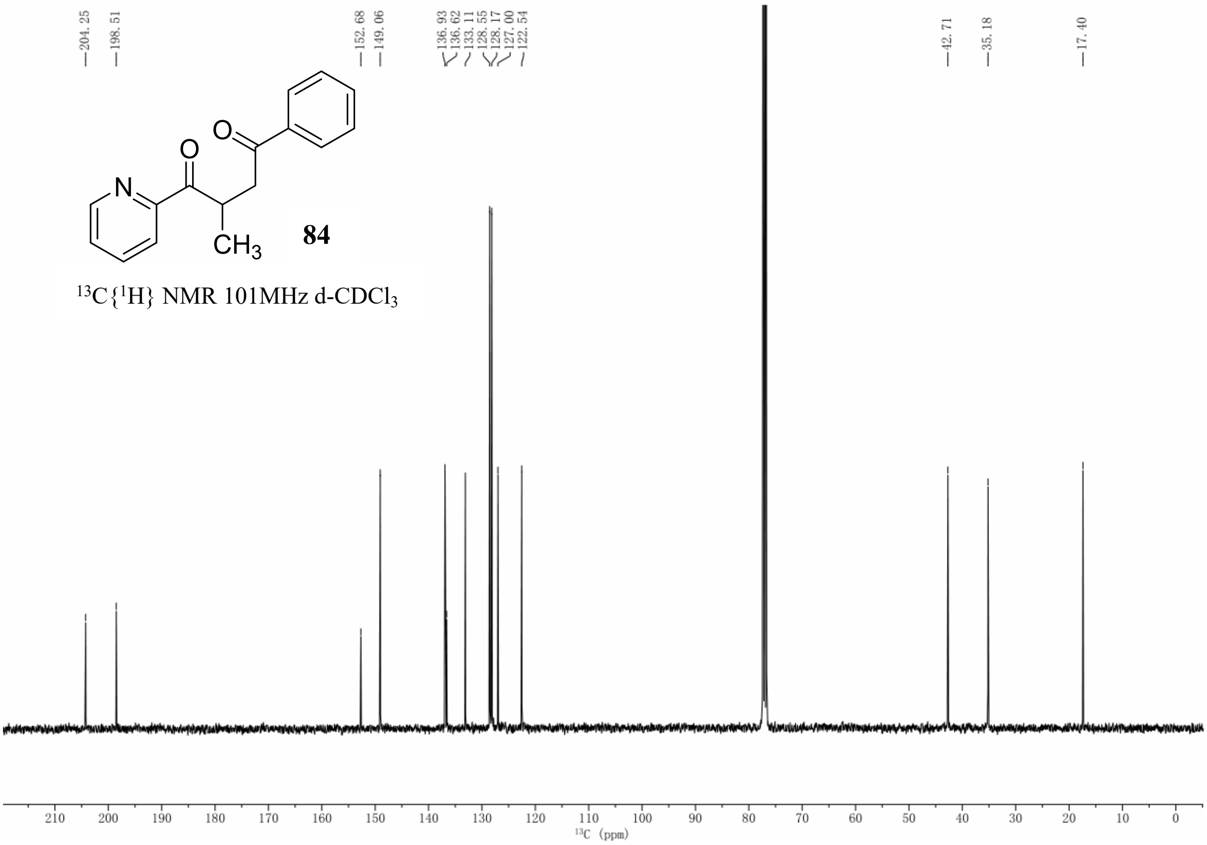
204.25
198.51
152.68
149.06
136.93
136.62
133.11
128.55
128.17
127.00
122.54
42.71
35.18
17.40
N
O
O
CH3
84
13C{1H} NMR 101MHz d-CDCl3
210 200 190 180 170 160 150 140 130 120 110 100 90 80 70 60 50 40 30 20 10 0
13C (ppm)

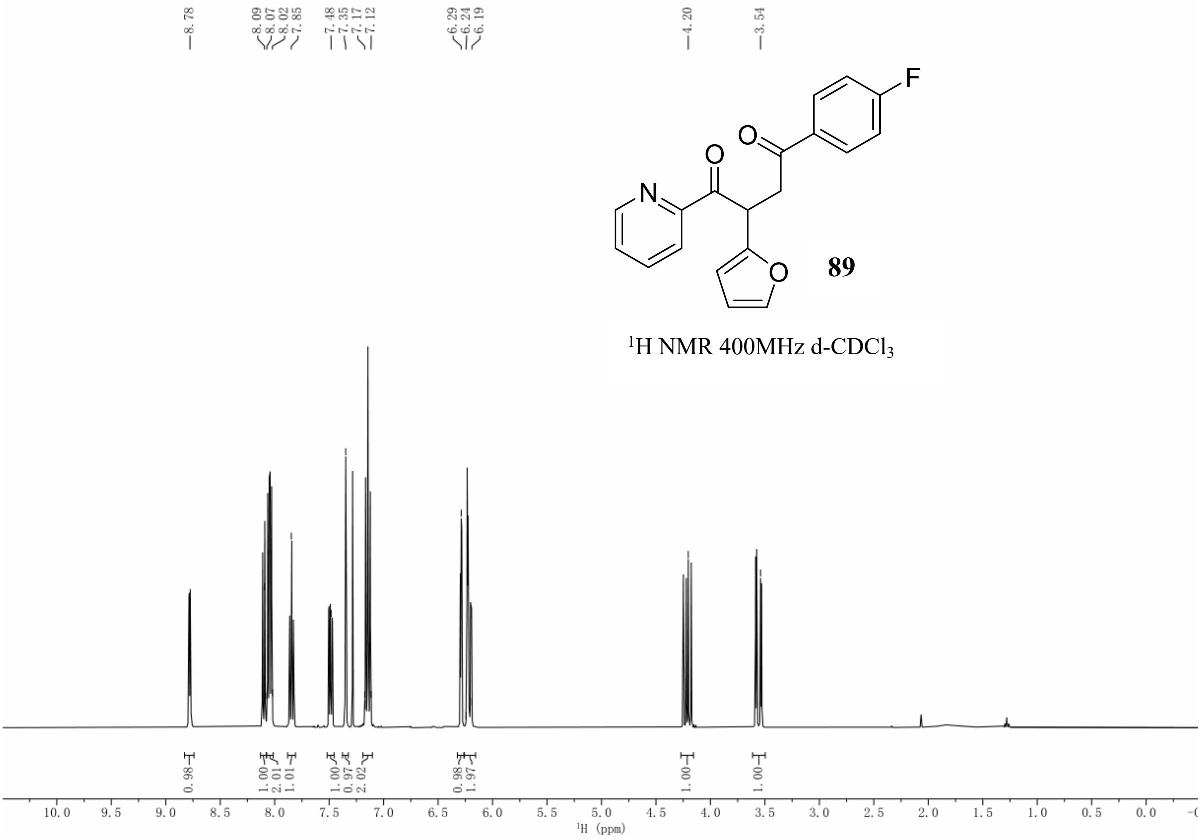
8.78
8.09
8.07
8.02
7.85
7.48
7.35
7.17
7.12
6.29
6.24
6.19
4.20
3.54
F
O
O
N
O
89
1H NMR 400MHz d-CDCl3
0.98
1.00
2.01
1.01
1.00
0.97
2.02
0.98
1.97
1.00
1.00
10.0 9.5 9.0 8.5 8.0 7.5 7.0 6.5 6.0 5.5 5.0 4.5 4.0 3.5 3.0 2.5 2.0 1.5 1.0 0.5 0.0
1H (ppm)

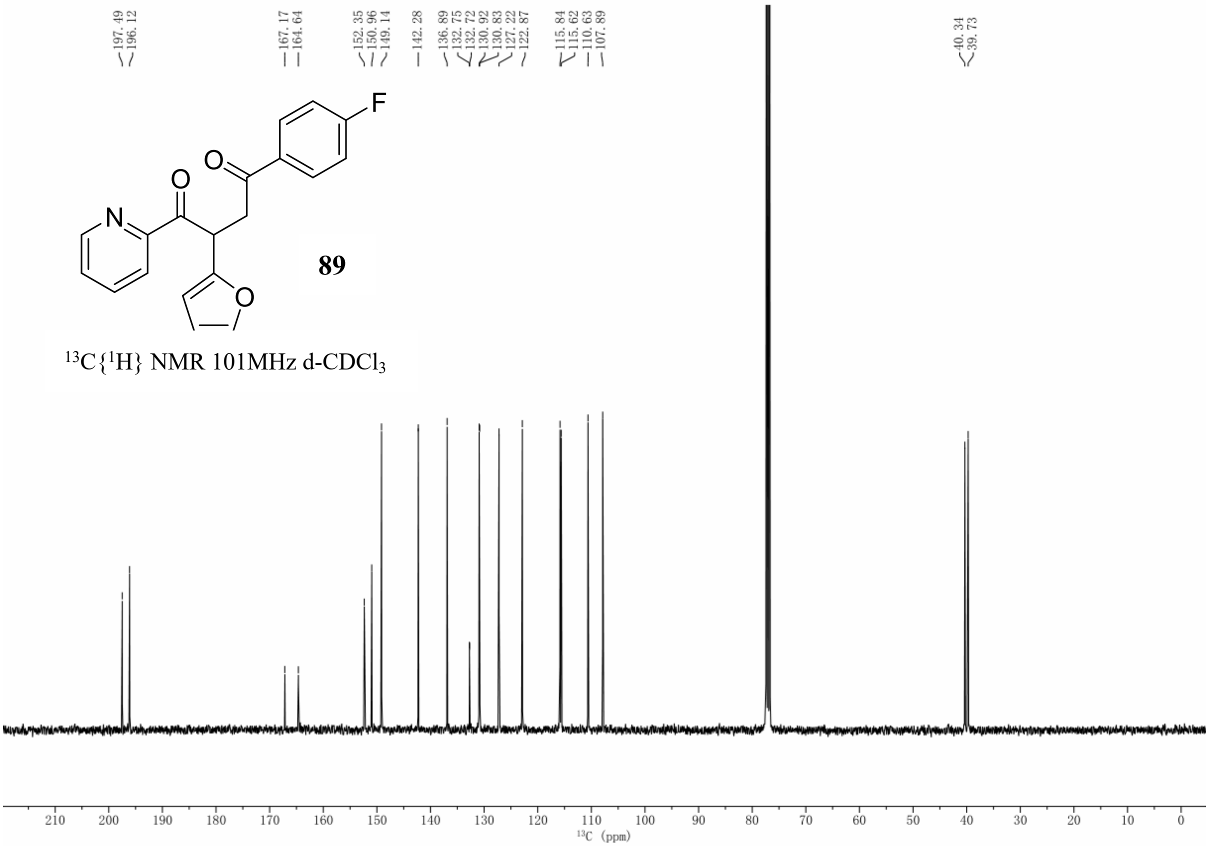
197.49
196.12
167.17
164.64
152.35
150.96
149.14
142.28
136.89
132.75
132.72
130.92
130.83
127.22
122.87
115.84
115.62
110.63
107.89
40.34
39.73
F
O
O
N
O
89
$^{13}C\{^1H\}$ NMR 101MHz d-CDCl$_3$
210 200 190 180 170 160 150 140 130 120 110 100 90 80 70 60 50 40 30 20 10 0
^{13}C (ppm)

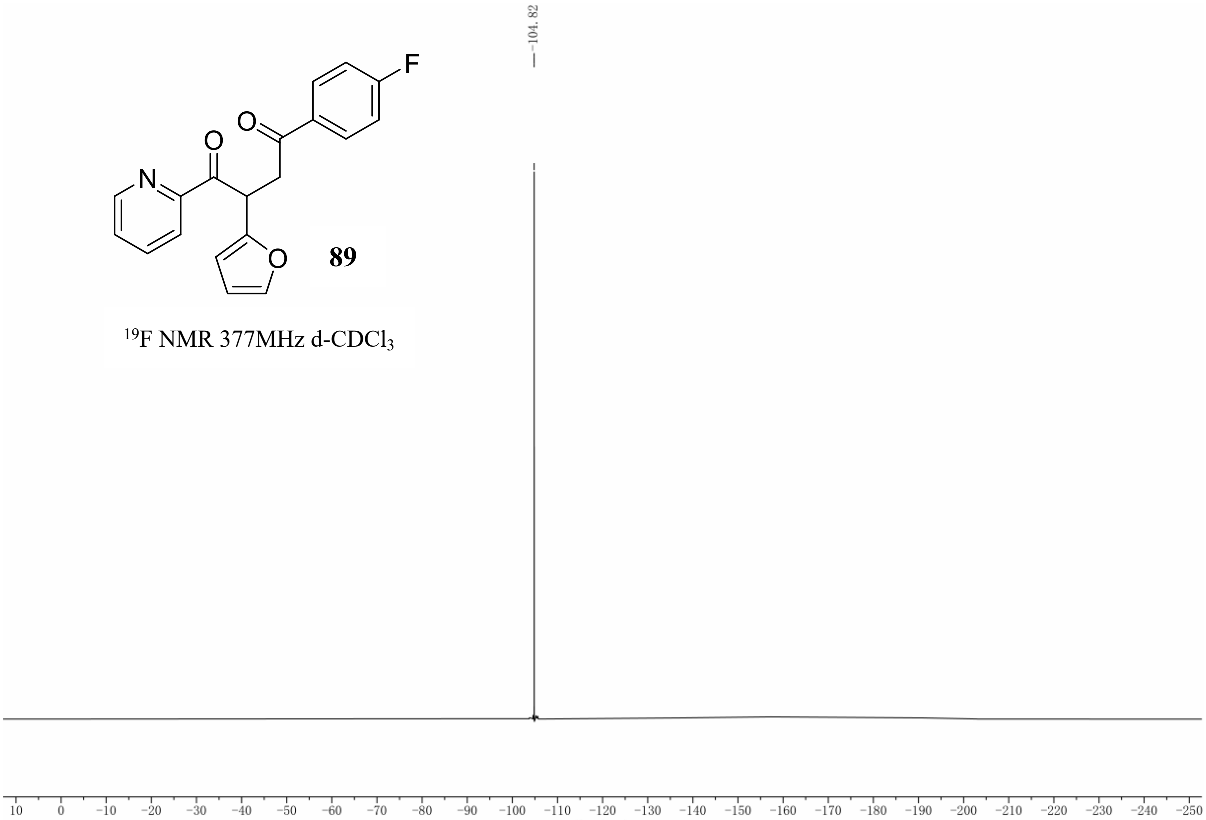
-104.82
F
O
O
N
O
89
^{19}F NMR 377MHz d-CDCl$_3$
10 0 -10 -20 -30 -40 -50 -60 -70 -80 -90 -100 -110 -120 -130 -140 -150 -160 -170 -180 -190 -200 -210 -220 -230 -240 -250
^{19}F (ppm)

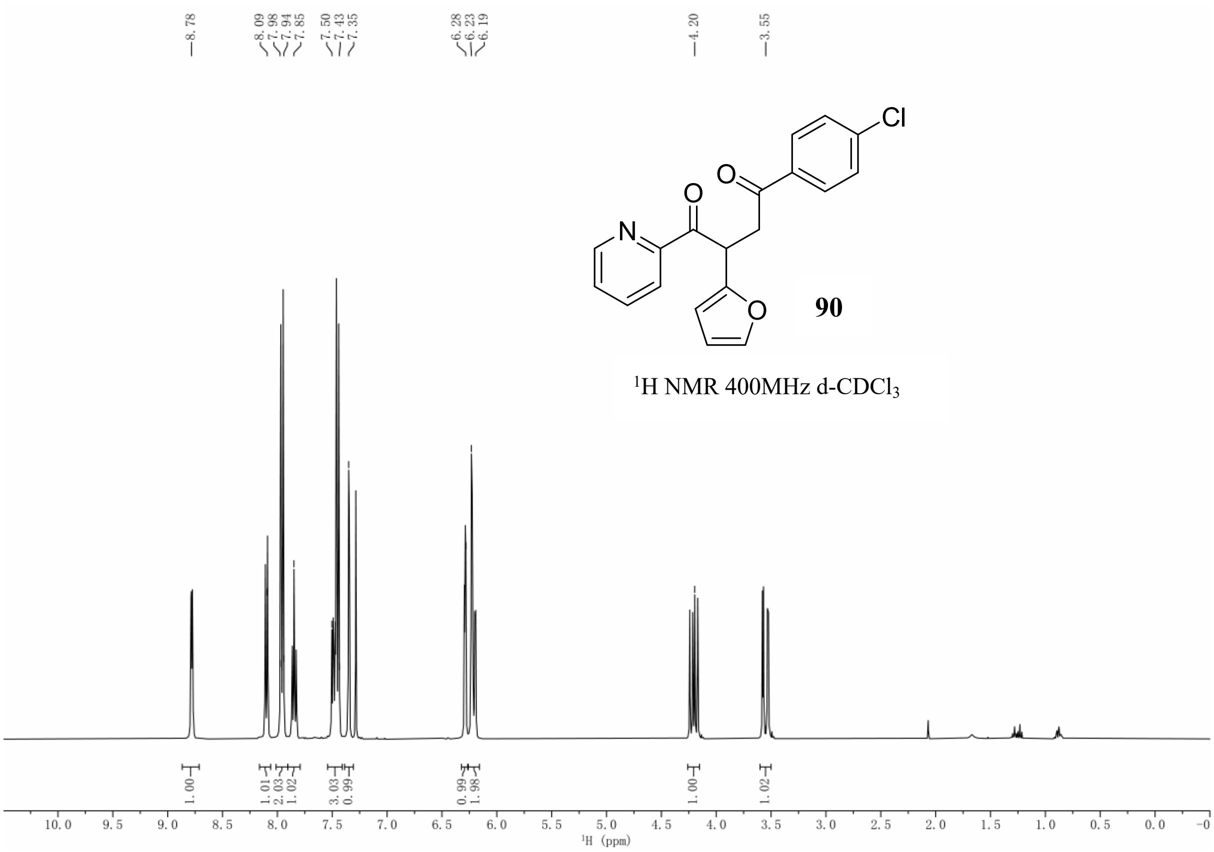
8.78
8.09
7.98
7.94
7.85
7.50
7.43
7.35
6.28
6.23
6.19
4.20
3.55
Cl
O
O
N
O
90
1H NMR 400MHz d-CDCl3
1.00
1.01
2.03
1.02
3.03
0.99
0.99
1.98
1.00
1.02
1H (ppm)

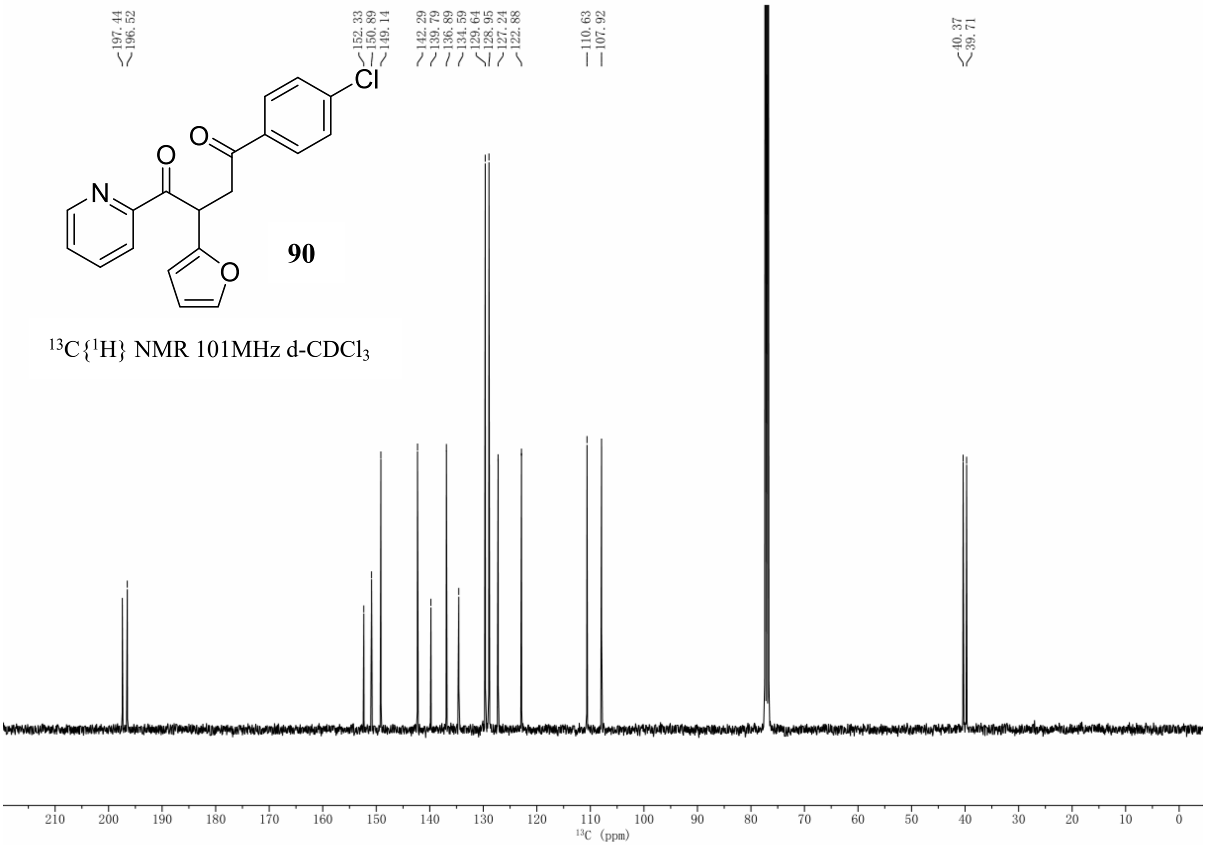
197.44
196.52
152.33
150.89
149.14
142.29
139.79
136.89
134.59
129.64
128.95
127.24
122.88
110.63
107.92
40.37
39.71
Cl
O
O
N
O
90
13C{1H} NMR 101MHz d-CDCl3
13C (ppm)

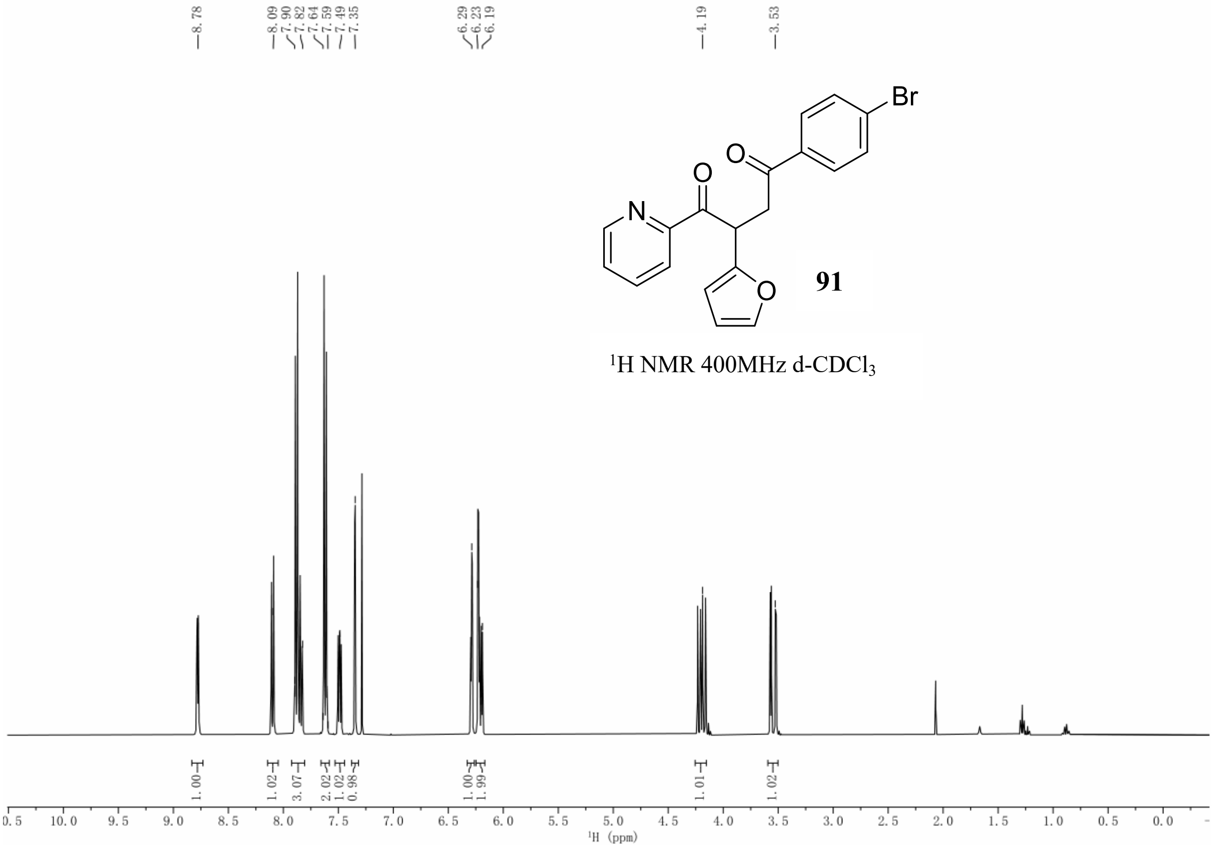
8.78
8.09
7.90
7.82
7.64
7.59
7.49
7.35
6.29
6.23
6.19
4.19
3.53
Br
O
O
N
O
91
1H NMR 400MHz d-CDCl3
1.00
1.02
3.07
2.02
1.02
0.98
1.00
1.99
1.01
1.02
1H (ppm)

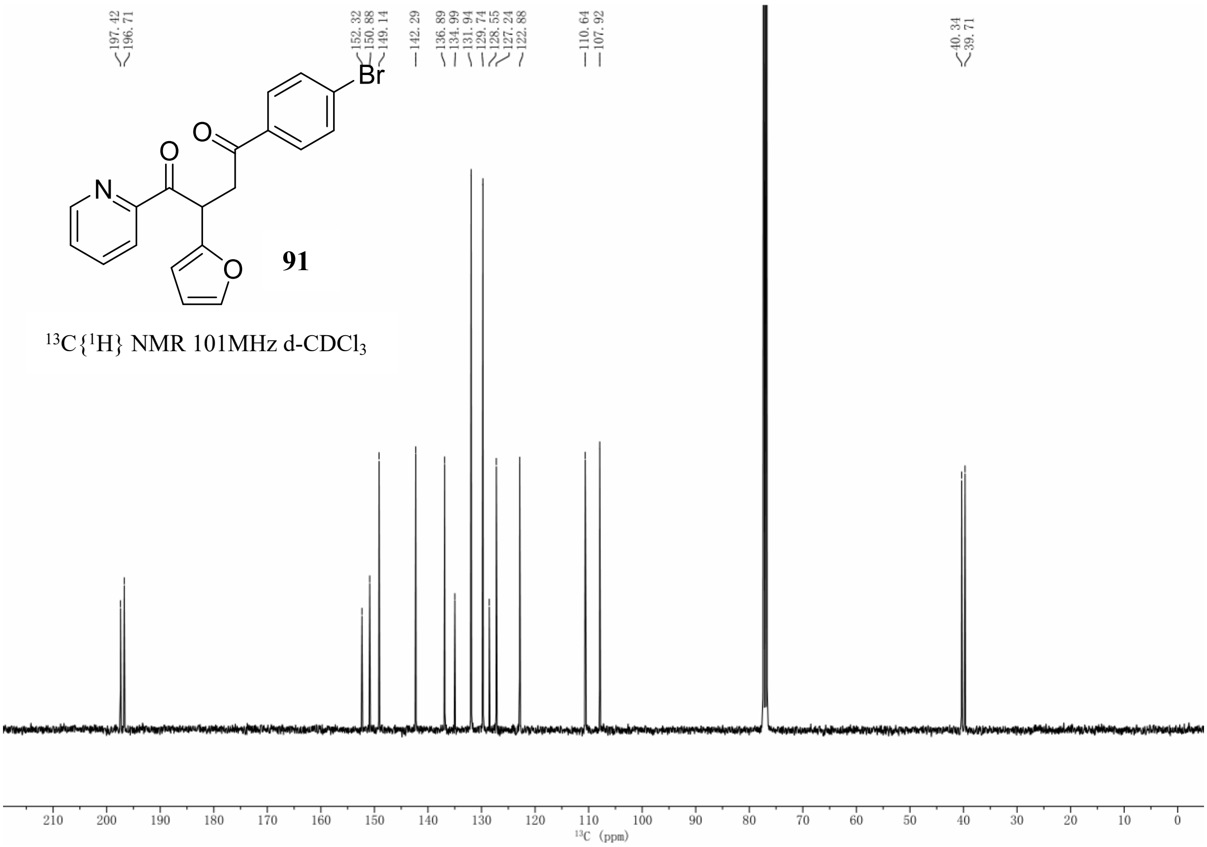
197.42
196.71
152.32
150.88
149.14
142.29
136.89
134.99
131.94
129.74
128.55
127.24
122.88
110.64
107.92
40.34
39.71
Br
O
O
N
O
91
13C{1H} NMR 101MHz d-CDCl3
13C (ppm)

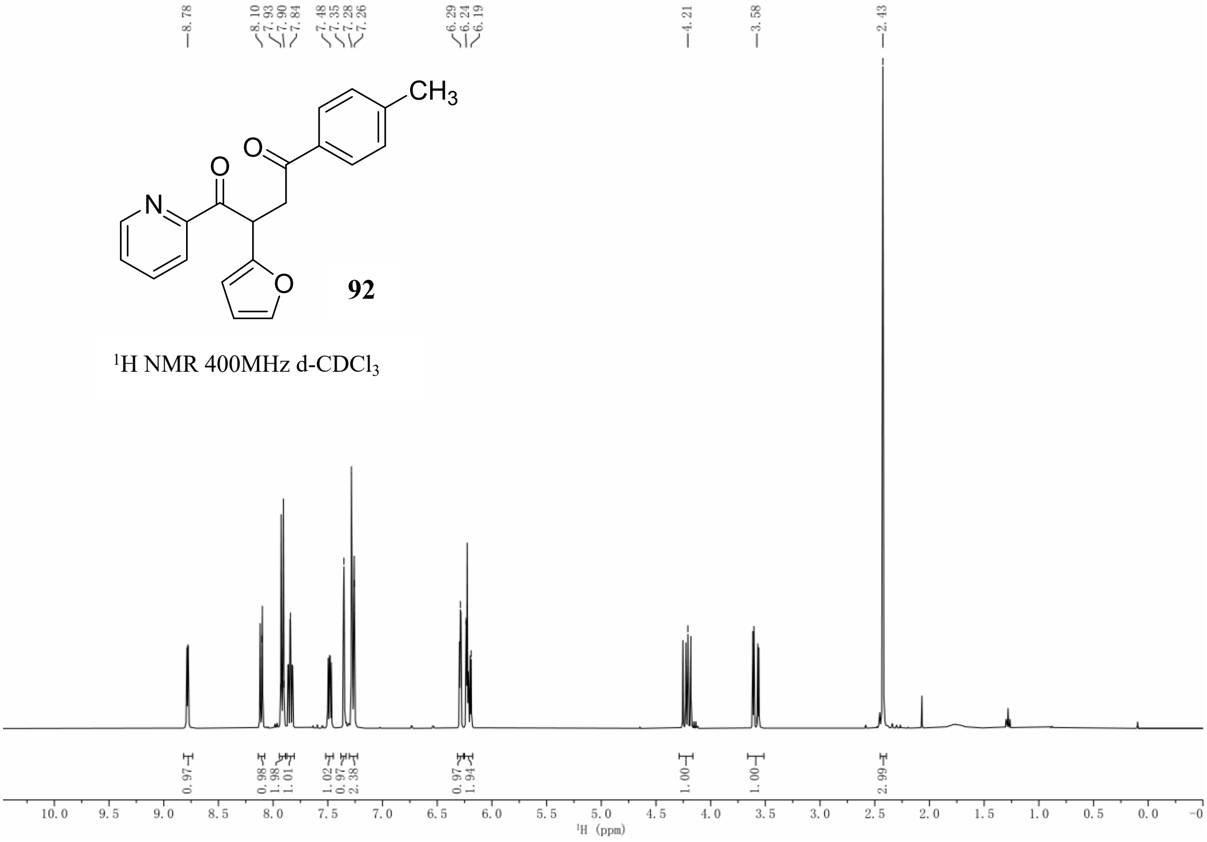

8.78
8.10
7.93
7.90
7.84
7.48
7.35
7.28
7.26
6.29
6.24
6.19
4.21
3.58
2.43
CH3
O
O
N
O
92
1H NMR 400MHz d-CDCl3
0.97
0.98
1.98
1.01
1.02
0.97
2.38
0.97
1.94
1.00
1.00
2.99
10.0 9.5 9.0 8.5 8.0 7.5 7.0 6.5 6.0 5.5 5.0 4.5 4.0 3.5 3.0 2.5 2.0 1.5 1.0 0.5 0.0
1H (ppm)

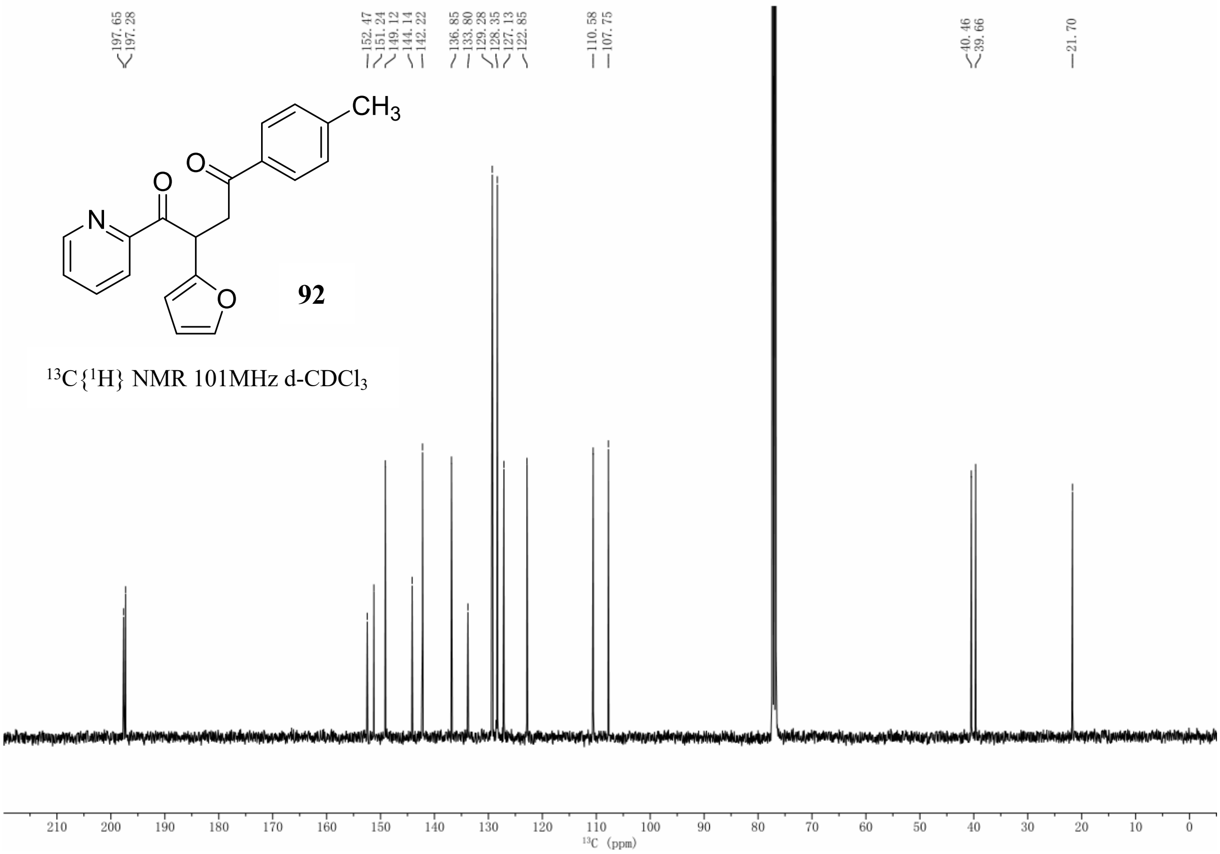

197.65
197.28
152.47
151.24
149.12
144.14
142.22
136.85
133.80
129.28
128.35
127.13
122.85
110.58
107.75
40.46
39.66
21.70
CH3
O
O
N
O
92
13C{1H} NMR 101MHz d-CDCl3
210 200 190 180 170 160 150 140 130 120 110 100 90 80 70 60 50 40 30 20 10 0
13C (ppm)

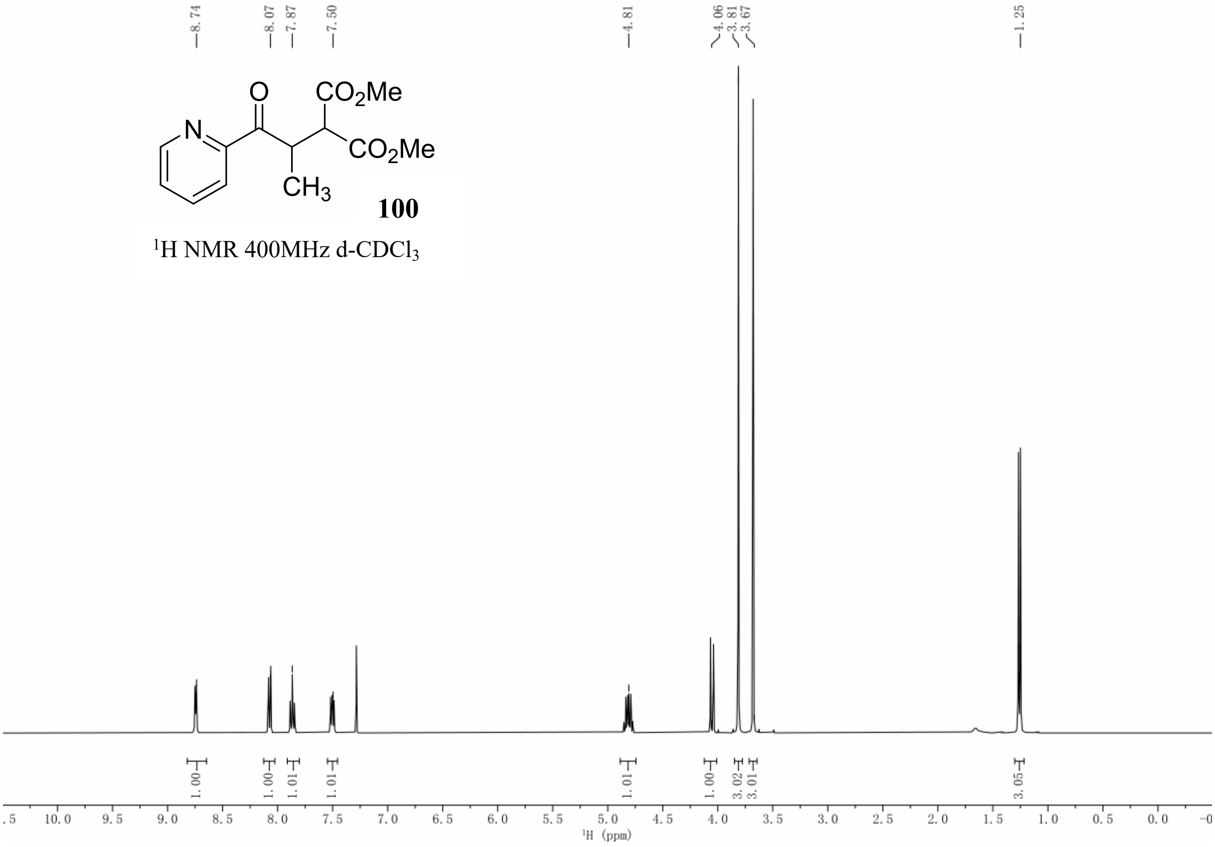
8.74
8.07
7.87
7.50
4.81
4.06
3.81
3.67
1.25
O
N
CO2Me
CO2Me
CH3
100
1H NMR 400MHz d-CDCl3
1.00
1.00
1.01
1.01
1.01
1.00
3.02
3.01
3.05
1H (ppm)

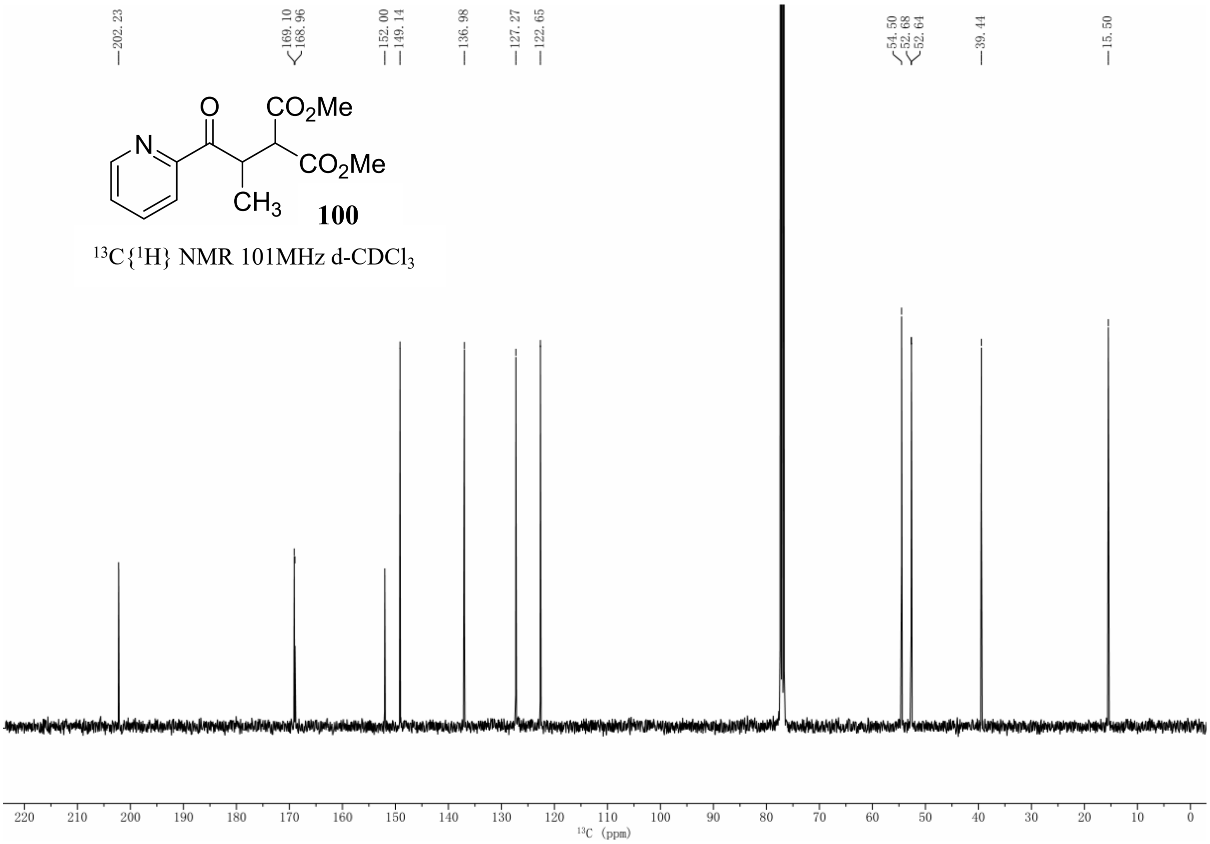
202.23
169.10
168.96
152.00
149.14
136.98
127.27
122.65
54.50
52.68
52.64
39.44
15.50
O
N
CO2Me
CO2Me
CH3
100
13C{1H} NMR 101MHz d-CDCl3
13C (ppm)

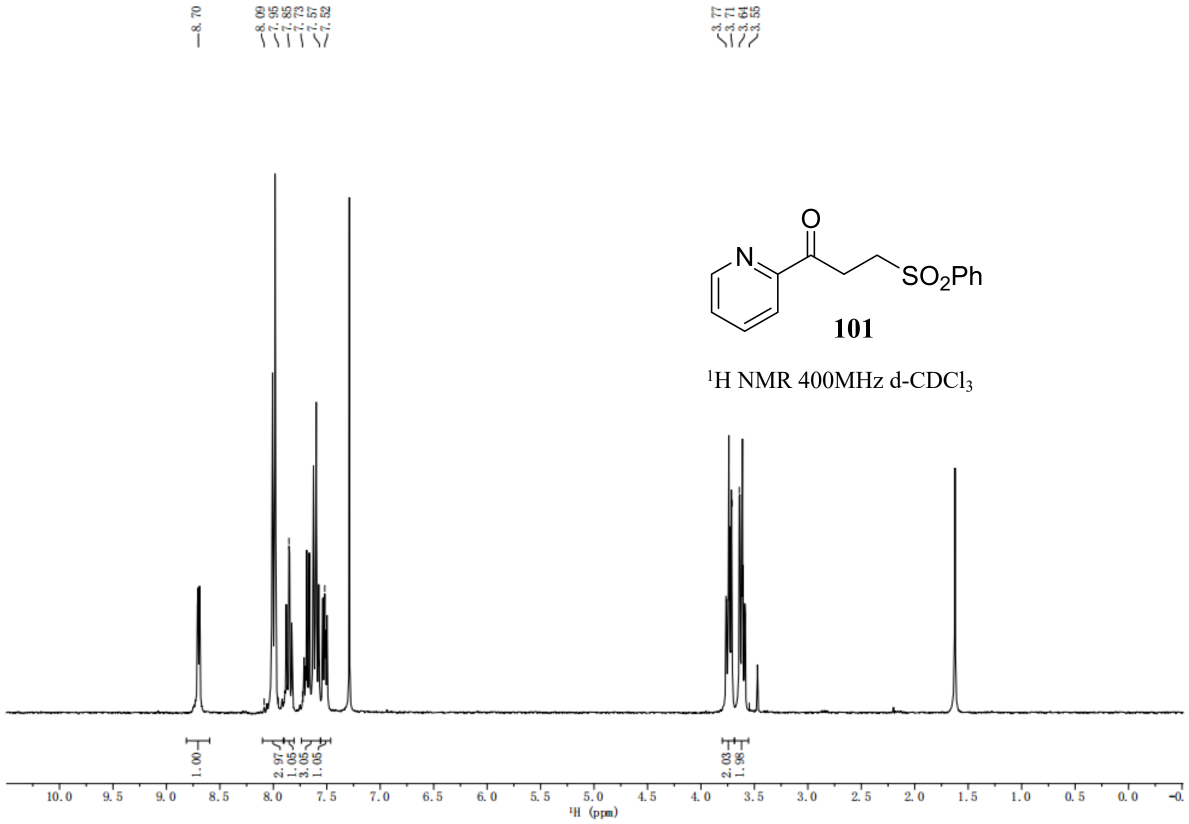
8.70
8.09
7.95
7.85
7.73
7.57
7.52
3.77
3.71
3.64
3.55
O
N
SO2Ph
101
1H NMR 400MHz d-CDCl3
1.00
2.97
1.05
3.05
1.05
2.03
1.98
1H (ppm)

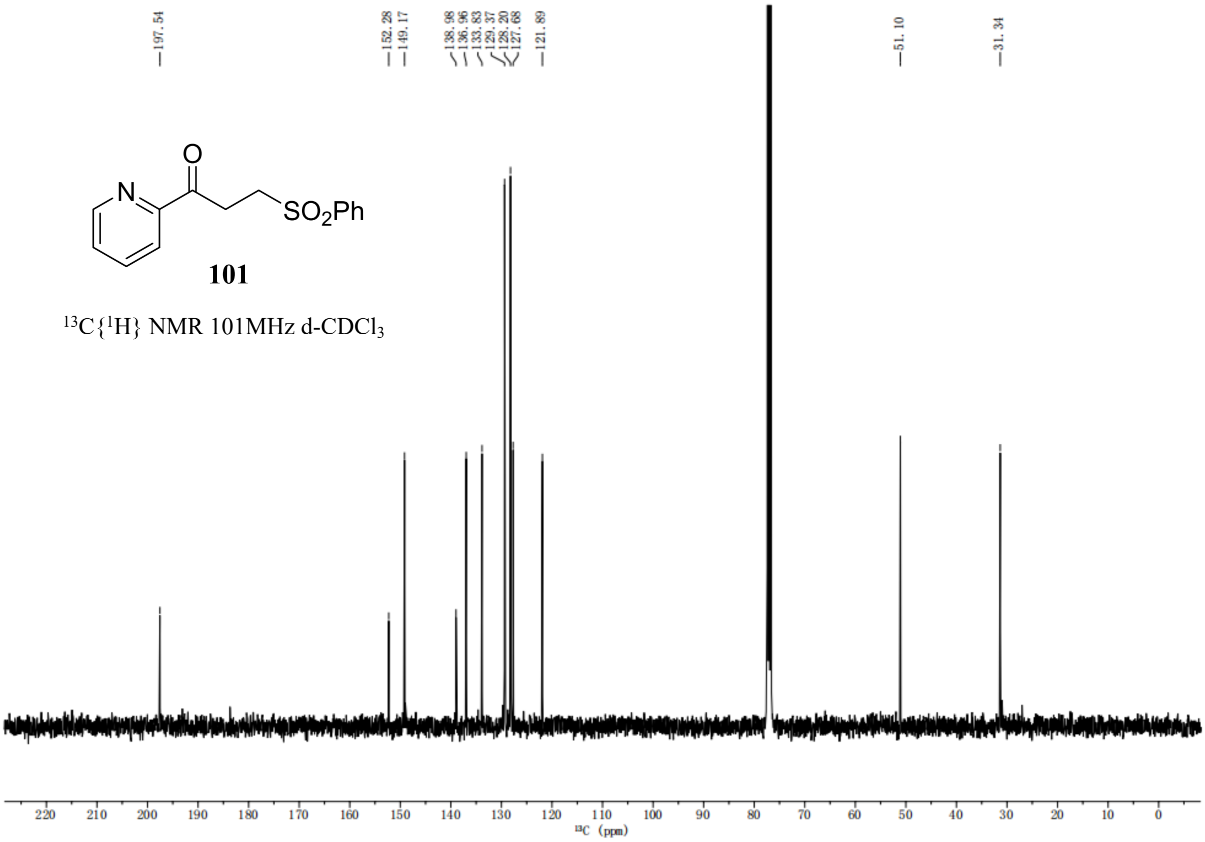
197.54
152.28
149.17
138.98
136.96
133.83
129.37
128.20
127.68
121.89
51.10
31.34
O
N
SO2Ph
101
13C{1H} NMR 101MHz d-CDCl3
13C (ppm)

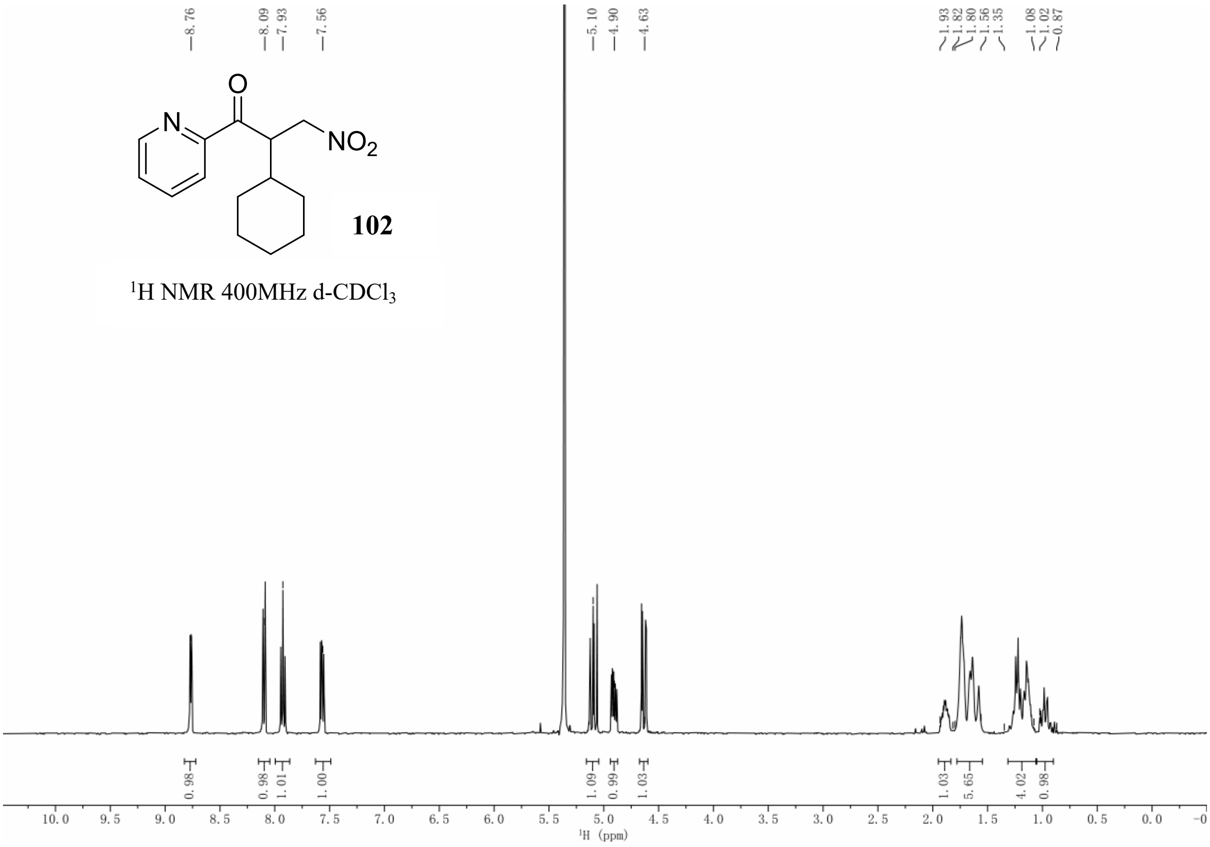

102

^{1}H NMR 400MHz d-$CDCl_3$

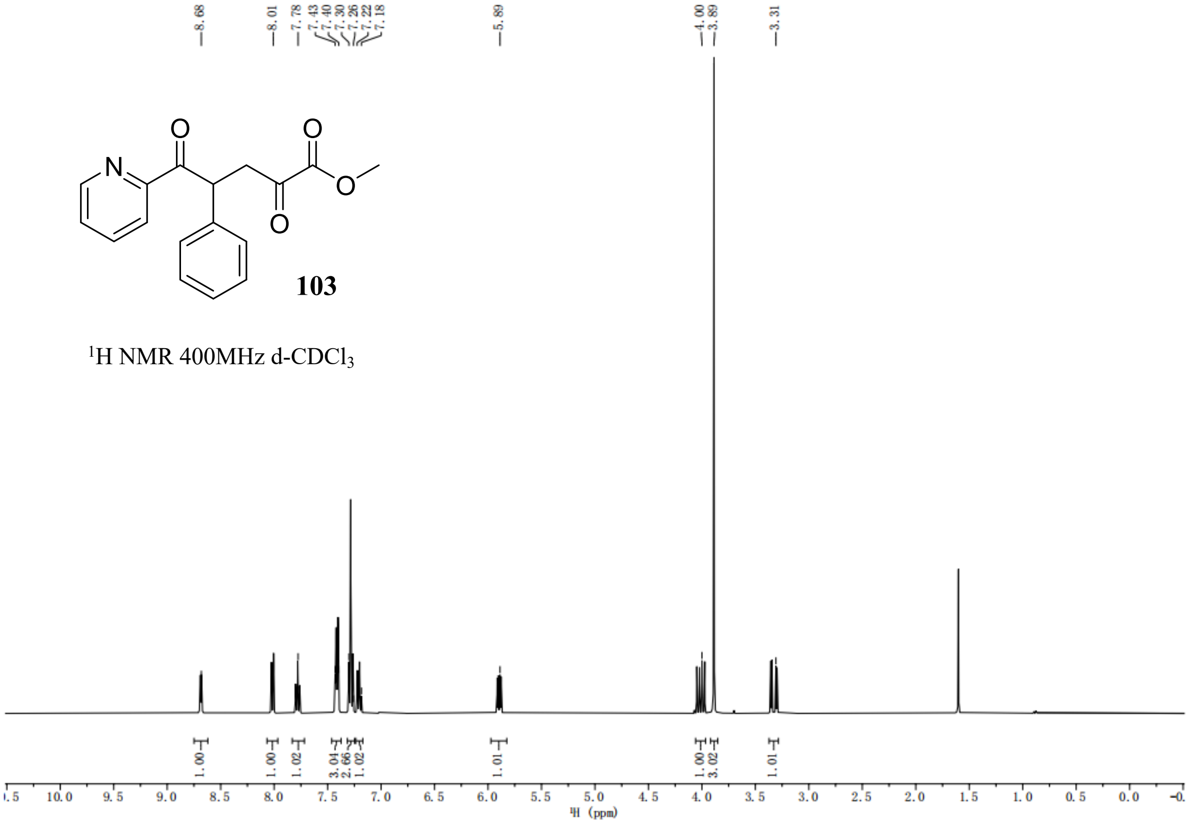

103

^{1}H NMR 400MHz d-$CDCl_3$

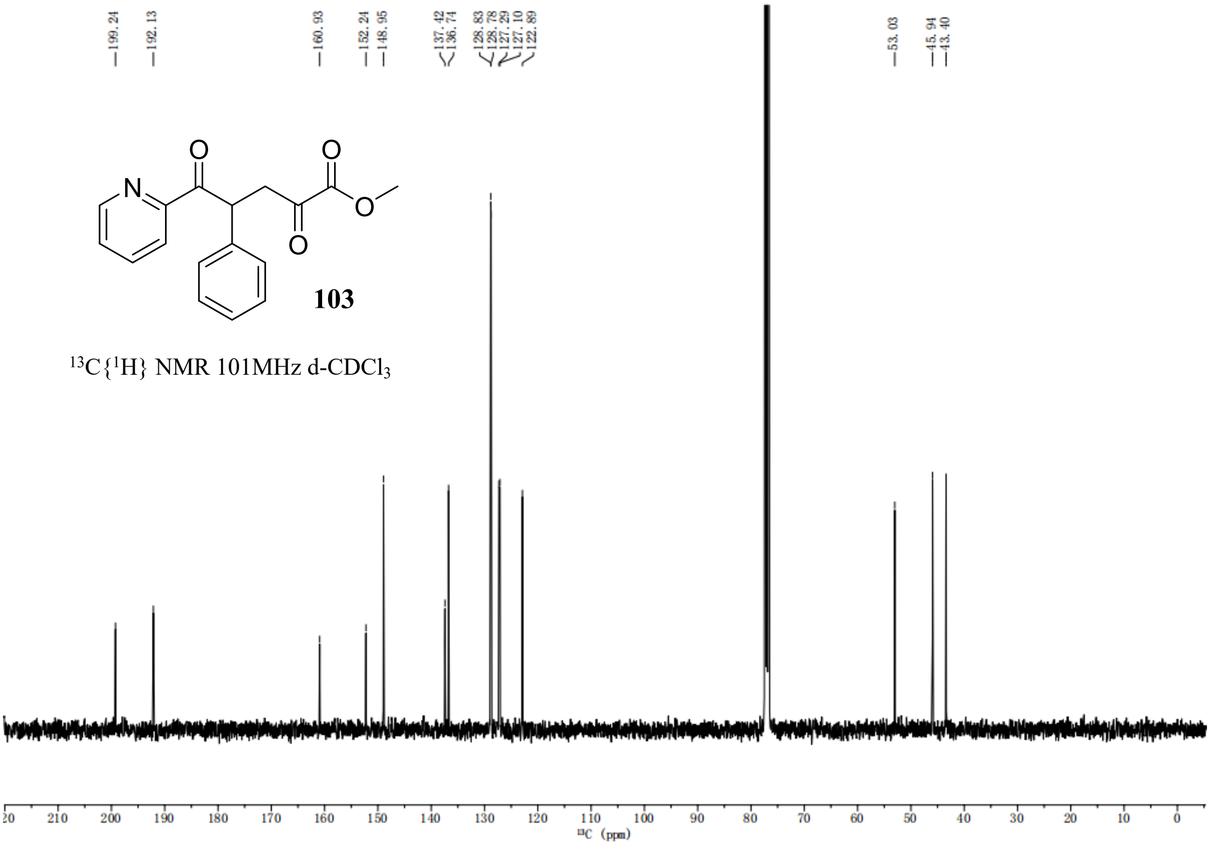
199.24
192.13
160.93
152.24
148.95
137.42
136.74
128.83
128.78
127.29
127.10
122.89
53.03
45.94
43.40
103
13C{1H} NMR 101MHz d-CDCl3
13C (ppm)

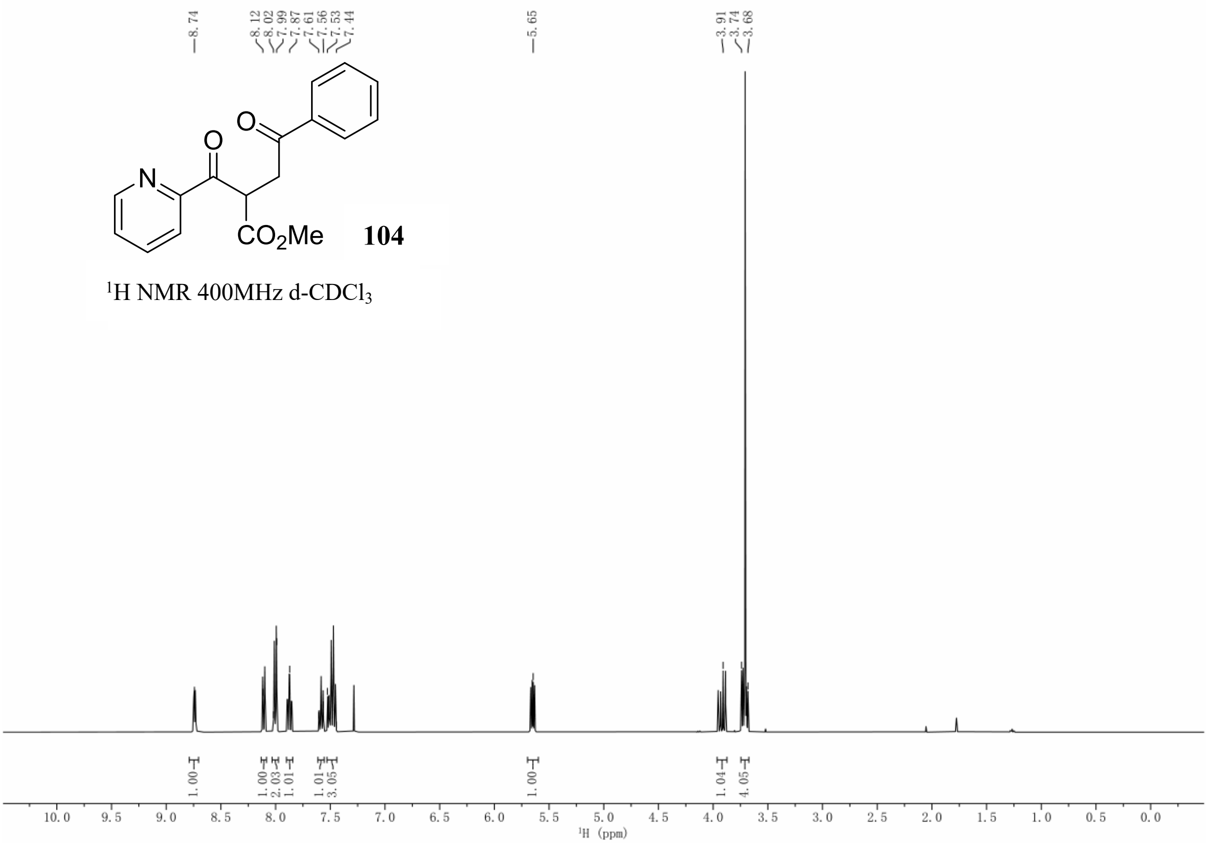
8.74
8.12
8.02
7.99
7.87
7.61
7.56
7.53
7.44
5.65
3.91
3.74
3.68
CO2Me
104
1H NMR 400MHz d-CDCl3
1.00
1.00
2.03
1.01
1.01
3.05
1.00
1.04
4.05
1H (ppm)

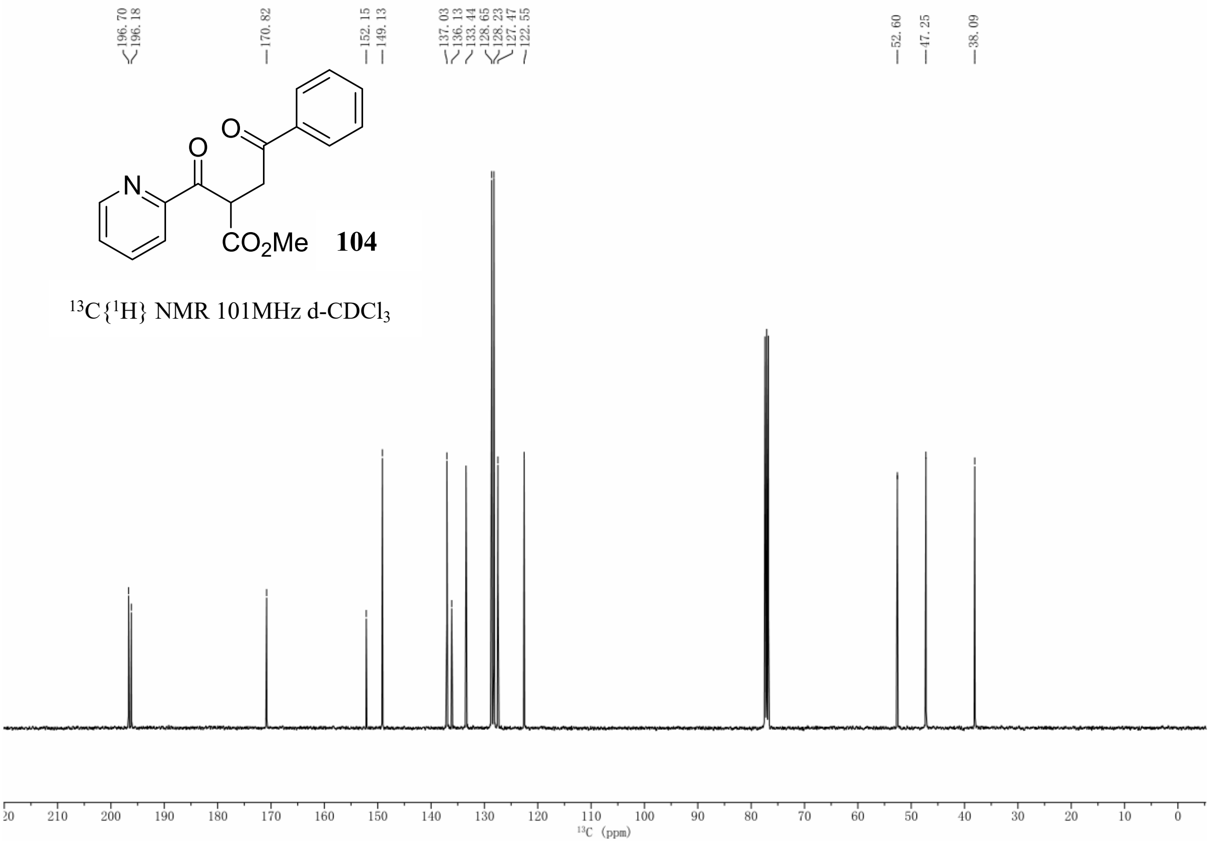
196.70
196.18
170.82
152.15
149.13
137.03
136.13
133.44
128.65
128.23
127.47
122.55
52.60
47.25
38.09
O
O
N
CO2Me
104
13C{1H} NMR 101MHz d-CDCl3
13C (ppm)

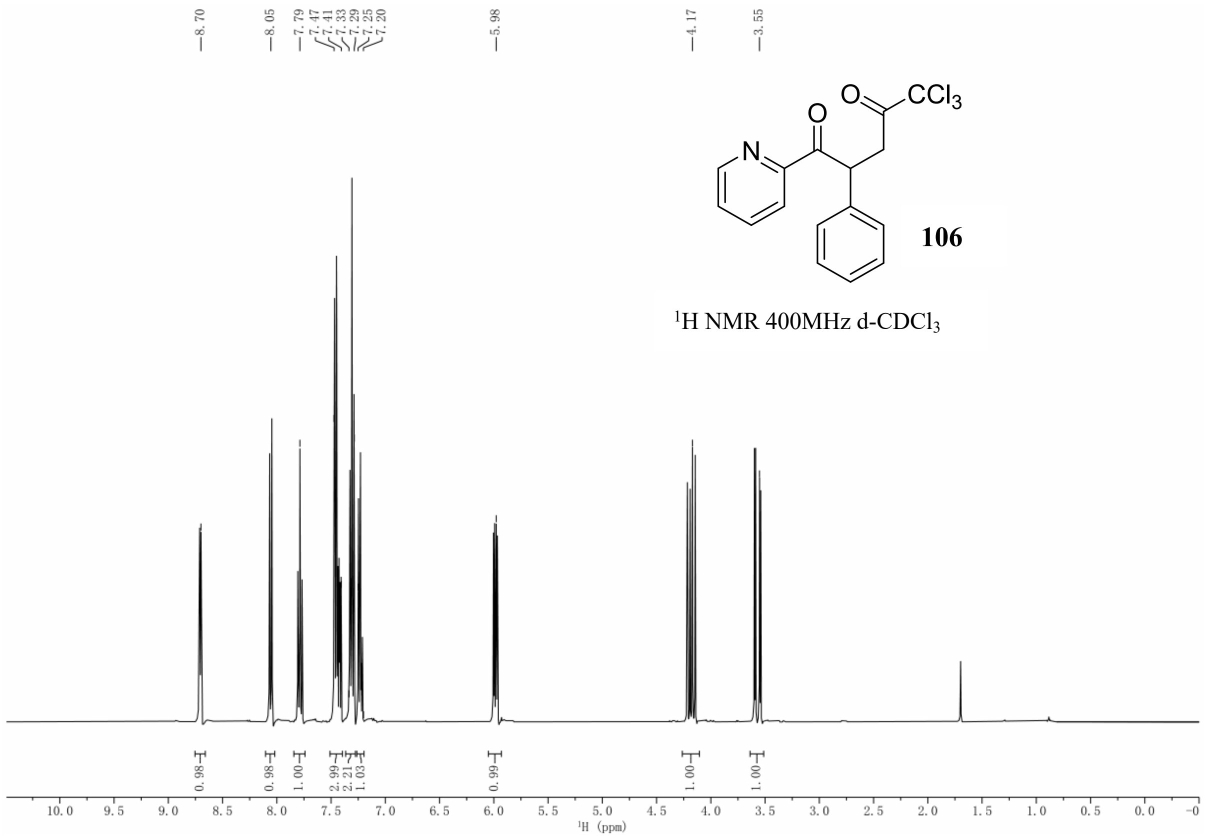
8.70
8.05
7.79
7.47
7.41
7.33
7.29
7.25
7.20
5.98
4.17
3.55
O
CCl3
O
N
106
1H NMR 400MHz d-CDCl3
0.98
0.98
1.00
2.99
2.21
1.03
0.99
1.00
1.00
1H (ppm)

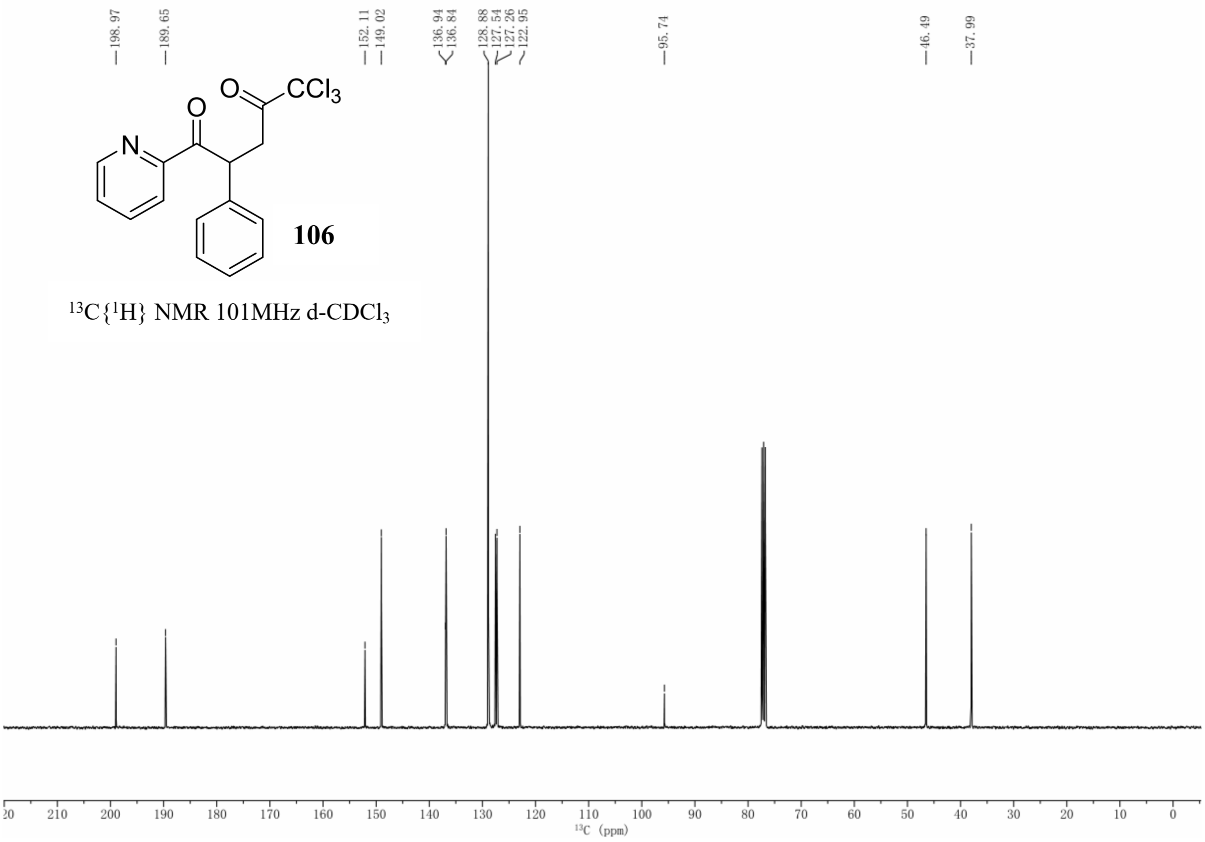
198.97
189.65
152.11
149.02
136.94
136.84
128.88
127.54
127.26
122.95
95.74
46.49
37.99
CCl3
106
13C{1H} NMR 101MHz d-CDCl3
13C (ppm)

11. Computational Raw Data

```
profile/NHC_cat
Frequencies, energies and thermodynamic properties:
 Lowest Vibrational Mode (1/cm) =                      32.3157
 2nd Lowest Vibrational Mode (1/cm) =                  47.0838
 E(RM062X) (a.u.) =                                  -1084.95309714
 Thermal correction to Enthalpy (a.u.) =                0.181652
 Thermal correction to Gibbs Free Energy (a.u.) =        0.122280
 Total Entropy (cal/Kmol) =                           124.960
 Esp(RM062X) (a.u.) =                                -1086.21061124
Optimised cartesian coordinates (Angstrom):
 C      1.686357  -0.842405  -0.920237
 C      4.290826  -0.843269  -0.853053
 C      2.858535   0.367838   0.573828
 C      5.094232   0.268445  -0.147492
 H      4.411098  -0.844210  -1.942084
 H      4.529473  -1.843470  -0.464527
 C      4.229222   0.717183   1.055309
 H      6.089327  -0.075480   0.156246
 H      4.457390   0.134532   1.960718
 H      5.220039   1.114467  -0.837295
 H      4.336615   1.780791   1.297803
 N      2.918311  -0.499075  -0.487164
 N      0.937680  -0.120471  -0.044653
 N      1.631636   0.631683   0.879745
 C     -0.470189  -0.063155  -0.030909
 C     -1.234365  -1.222315   0.104502
 C     -1.122925   1.165559  -0.144795
 C     -2.623924  -1.157723   0.107870
 C     -2.510686   1.238477  -0.122137
 C     -3.261702   0.073474  -0.000367
 F     -0.425088   2.277673  -0.290562
 F     -3.119372   2.407971  -0.231586
 F     -4.580496   0.136924   0.012050
 F     -3.340699  -2.262466   0.235607
 F     -0.653190  -2.400573   0.236552
---------------------------------------
profile/TS-i
Frequencies, energies and thermodynamic properties:
 Lowest Vibrational Mode (1/cm) =                    -186.9190
 2nd Lowest Vibrational Mode (1/cm) =                  12.4217
 E(RM062X) (a.u.) =                                  -1446.15251808
 Thermal correction to Enthalpy (a.u.) =                0.289401
```

Thermal correction to Gibbs Free Energy (a.u.) = 0.214461

Total Entropy (cal/Kmol) = 157.724

Esp(RM062X) (a.u.) = -1447.81386654

Optimised cartesian coordinates (Angstrom):

C	0.084630	2.767664	0.382468
C	1.386219	2.984167	0.827550
C	2.445105	2.511639	0.052744
C	2.148738	1.832976	-1.131648
C	-0.103746	2.066004	-0.809306
H	-0.792079	3.124587	0.923936
H	1.575455	3.516767	1.761734
H	3.482559	2.657658	0.356569
H	2.958881	1.450481	-1.761927
N	0.904623	1.604778	-1.551948
C	-1.517875	1.826392	-1.321676
O	-2.463412	2.501382	-0.877750
C	-1.773545	0.032173	-0.561156
C	-4.366293	0.278917	-0.458579
C	-3.024675	-1.238791	0.760016
C	-5.255968	-0.837561	0.125595
H	-4.505549	0.438792	-1.532752
H	-4.469252	1.242290	0.056087
C	-4.414146	-1.527373	1.226712
H	-6.201445	-0.440859	0.511560
H	-4.566308	-1.059962	2.211207
H	-5.486719	-1.565935	-0.663920
H	-4.614452	-2.600695	1.322802
N	-3.027330	-0.247492	-0.184889
N	-1.063692	-0.835699	0.189023
N	-1.817455	-1.630344	1.018402
C	0.343457	-0.958456	0.195373
C	1.075902	-0.429433	1.255651
C	1.008657	-1.574374	-0.861746
C	2.463241	-0.502417	1.256403
C	2.396363	-1.657459	-0.863225
C	3.120055	-1.127041	0.200736
F	0.328672	-2.065661	-1.879521
F	3.032102	-2.232597	-1.869854
F	4.438314	-1.180468	0.189110
F	3.160619	0.051992	2.232839
F	0.455580	0.183084	2.246539
H	-1.481119	1.497796	-2.384907

----

profile/TS-ii

Frequencies, energies and thermodynamic properties:

Lowest Vibrational Mode (1/cm) = -200.0694

2nd Lowest Vibrational Mode (1/cm) = 17.0377

E(RM062X) (a.u.) = -1807.39131612

Thermal correction to Enthalpy (a.u.) = 0.397836

Thermal correction to Gibbs Free Energy (a.u.) = 0.309570

Total Entropy (cal/Kmol) = 185.771

Esp(RM062X) (a.u.) = -1809.44946463

Optimised cartesian coordinates (Angstrom):

C	2.353942	-1.597676	1.556099
C	3.573002	-1.762196	2.199239
C	4.368239	-0.641603	2.452179
C	3.887138	0.600328	2.049319
C	1.969365	-0.303467	1.166102
H	1.697651	-2.437893	1.336510
H	3.902908	-2.757238	2.504621
H	5.330943	-0.725759	2.956775
H	4.470126	1.507087	2.241597
N	2.720544	0.766179	1.424833
C	0.703638	-0.131512	0.403222
O	-0.201093	-1.127341	0.594888
H	-0.438356	-1.364952	-0.397129
C	0.090935	1.200276	0.334752
C	1.929725	3.082472	-0.015553
C	-0.415251	3.294651	-0.121870
C	1.553136	4.366564	-0.775530
H	2.621265	2.430387	-0.555947
H	2.336937	3.274412	0.982682
C	0.093587	4.671357	-0.382126
H	2.236660	5.187680	-0.533118
H	0.035179	5.260388	0.545704
H	1.606306	4.178132	-1.856815
H	-0.480961	5.187360	-1.159157
N	0.617187	2.423752	0.124833
N	-1.245466	1.400842	0.189515
N	-1.560300	2.701624	-0.093292
C	-2.307566	0.464367	0.193667
C	-2.687536	-0.182800	1.365224
C	-2.991988	0.216435	-0.992416
C	-3.743569	-1.083440	1.353627
C	-4.057008	-0.677739	-1.007774
C	-4.428066	-1.326858	0.165239
F	-2.638407	0.827725	-2.104255
F	-4.715066	-0.915905	-2.130733

```
 F     -5.435945  -2.179310   0.153372
 F     -4.103470  -1.711965   2.461555
 F     -2.027134   0.037784   2.484035
 C      0.916956  -0.389042  -1.511726
 C      2.203730  -1.174332  -1.578848
 H      1.069029   0.654343  -1.864413
 C      3.433056  -0.505541  -1.660547
 C      3.247698  -3.211193  -1.498418
 C      4.607522  -1.248766  -1.646953
 H      3.454882   0.584540  -1.728524
 C      4.517363  -2.638639  -1.562680
 H      3.142378  -4.299414  -1.437209
 H      5.579582  -0.755155  -1.704993
 H      5.407718  -3.268158  -1.551906
 O     -0.192401  -0.976494  -1.779509
 N      2.117693  -2.503814  -1.500582
---------------------------------------
profile/TS-iii
Frequencies, energies and thermodynamic properties:
 Lowest Vibrational Mode (1/cm) =                       -120.9215
 2nd Lowest Vibrational Mode (1/cm) =                     16.2931
 E(RM062X) (a.u.) =                                   -1807.38964237
 Thermal correction to Enthalpy (a.u.) =                  0.399395
 Thermal correction to Gibbs Free Energy (a.u.) =         0.308469
 Total Entropy (cal/Kmol) =                             191.369
 Esp(RM062X) (a.u.) =                                 -1809.44951815
Optimised cartesian coordinates (Angstrom):
 C      0.119857  -2.254387   0.572316
 C     -0.139582  -3.117473   1.636987
 C      0.318385  -2.767353   2.902899
 C      1.022042  -1.566828   3.047729
 C      0.807828  -1.069289   0.830651
 H     -0.209927  -2.464281  -0.445842
 H     -0.689950  -4.046902   1.478721
 H      0.140294  -3.405487   3.769081
 H      1.401664  -1.270745   4.030637
 N      1.259839  -0.735702   2.040120
 C      1.053719  -0.118092  -0.322339
 O      0.493202  -0.301021  -1.410148
 C      0.027437   1.585766   0.448086
 C      1.526064   3.675438   0.864514
 C     -0.786736   3.656686   0.372405
 C      0.922371   5.058190   1.189620
 H      2.150925   3.669502  -0.038678
```

```
 H      2.090096   3.242337   1.698361
 C     -0.451864   5.108910   0.477416
 H      0.770783   5.139611   2.274522
 H     -1.207758   5.679706   1.029101
 H      1.584072   5.872946   0.875624
 H     -0.372764   5.533695  -0.534587
 N      0.325313   2.884898   0.596319
 N     -1.286937   1.650631   0.154913
 N     -1.813594   2.918698   0.096389
 C     -2.103334   0.533052  -0.116500
 C     -2.352663  -0.421342   0.865308
 C     -2.615155   0.344050  -1.399381
 C     -3.104926  -1.553936   0.572547
 C     -3.384581  -0.773287  -1.694079
 C     -3.624482  -1.725416  -0.705349
 F     -2.355754   1.224489  -2.346532
 F     -3.874043  -0.949860  -2.910398
 F     -4.343700  -2.795994  -0.986088
 F     -3.307166  -2.475449   1.500469
 F     -1.848227  -0.284741   2.077798
 C      2.432787   0.581410  -0.380472
 C      3.479625  -0.473085  -0.710400
 C      3.848024  -0.715787  -2.037756
 C      4.775930  -1.719704  -2.298979
 H      3.419131  -0.115684  -2.839650
 C      4.873236  -2.123062   0.056065
 C      5.302906  -2.443720  -1.231054
 H      5.085584  -1.933670  -3.323729
 H      5.270217  -2.666480   0.919309
 H      6.033950  -3.237858  -1.385989
 O      2.355785   1.571276  -1.365297
 H      1.652099   1.266425  -1.967846
 N      3.985398  -1.163522   0.313672
 H      2.678220   1.019367   0.595983
---------------------------------------
profile/TS-iv
Frequencies, energies and thermodynamic properties:
 Lowest Vibrational Mode (1/cm) =                        -427.5153
 2nd Lowest Vibrational Mode (1/cm) =                     15.1094
 E(RM062X) (a.u.) =                                    -2099.48919335
 Thermal correction to Enthalpy (a.u.) =                  0.532204
 Thermal correction to Gibbs Free Energy (a.u.) =         0.427803
 Total Entropy (cal/Kmol) =                              219.729
 Esp(RM062X) (a.u.) =                                  -2101.85613170
```

Optimised cartesian coordinates (Angstrom):

C	-1.894300	-0.762635	2.365412
C	-2.774928	-1.586839	3.056774
C	-2.496669	-2.949621	3.159228
C	-1.332361	-3.422808	2.554123
C	-0.741673	-1.336377	1.794190
H	-2.068433	0.308370	2.270823
H	-3.674116	-1.167181	3.512082
H	-3.160056	-3.631125	3.692079
H	-1.074662	-4.485927	2.611439
N	-0.483783	-2.643908	1.889344
C	0.134507	-0.479106	0.983785
O	0.002961	0.868206	1.189094
H	-0.781124	1.261124	0.670104
C	1.497312	-0.823763	0.669701
C	2.005191	-3.441199	0.779229
C	3.440796	-1.700798	0.091991
C	3.250379	-4.042353	0.095741
H	1.065770	-3.770570	0.329640
H	1.965884	-3.636642	1.856517
C	4.297163	-2.915644	0.001389
H	3.623016	-4.907686	0.655298
H	4.994818	-2.923225	0.852026
H	2.980235	-4.380115	-0.912565
H	4.886358	-2.935034	-0.922851
N	2.184194	-1.990380	0.562328
N	2.398290	0.109183	0.216196
N	3.600372	-0.442926	-0.136915
C	2.293450	1.516161	0.099069
C	2.352549	2.331256	1.227828
C	2.215914	2.098793	-1.160743
C	2.297568	3.711260	1.100126
C	2.166058	3.482464	-1.300418
C	2.211176	4.285684	-0.167199
F	2.161390	1.337793	-2.242153
F	2.082303	4.031748	-2.500739
F	2.166227	5.599188	-0.288992
F	2.335929	4.487775	2.170903
F	2.426955	1.788128	2.426128
C	-0.803735	-0.629864	-0.921185
C	-2.193623	-0.430128	-0.756601
C	-0.312381	-1.896314	-1.506603
H	-0.211743	0.255207	-1.185645
C	-2.732710	0.832350	-0.426068

```
 H     -2.875605  -1.262855  -0.917465
 C      0.892892  -1.910378  -2.227669
 C     -1.015171  -3.103547  -1.355431
 O     -2.041378   1.783561   0.030045
 C     -4.206061   1.076411  -0.590601
 C      1.374731  -3.088673  -2.794262
 H      1.452831  -0.978410  -2.344218
 C     -0.530660  -4.283181  -1.917351
 H     -1.944177  -3.124197  -0.783395
 C     -4.767620   2.185831   0.056297
 C     -5.033346   0.255993  -1.370942
 C      0.663484  -4.280717  -2.641464
 H      2.309551  -3.075798  -3.358448
 H     -1.089710  -5.211967  -1.789457
 C     -6.128141   2.462196  -0.059129
 H     -4.113221   2.824144   0.651086
 C     -6.394259   0.533363  -1.488026
 H     -4.611394  -0.594274  -1.908538
 H      1.038826  -5.204833  -3.084920
 C     -6.945868   1.634554  -0.830634
 H     -6.553814   3.326595   0.453904
 H     -7.026740  -0.109577  -2.102749
 H     -8.011874   1.850136  -0.924192
---------------------------------------
profile/TS-v
Frequencies, energies and thermodynamic properties:
 Lowest Vibrational Mode (1/cm) =                       -136.4279
 2nd Lowest Vibrational Mode (1/cm) =                     11.9959
 E(RM062X) (a.u.) =                                   -2099.49997630
 Thermal correction to Enthalpy (a.u.) =                 0.533463
 Thermal correction to Gibbs Free Energy (a.u.) =        0.426136
 Total Entropy (cal/Kmol) =                             225.887
 Esp(RM062X) (a.u.) =                                 -2101.86755658
Optimised cartesian coordinates (Angstrom):
 C      3.716295  -0.167087  -2.009606
 C      4.979213   0.287749  -1.636123
 C      5.112066   0.996561  -0.443987
 C      3.967056   1.219979   0.323063
 C      2.635353   0.104580  -1.169445
 H      3.537038  -0.720595  -2.931085
 H      5.848883   0.094762  -2.267436
 H      6.079138   1.373229  -0.109396
 H      4.033803   1.766263   1.269743
 N      2.759345   0.788485  -0.030476
```

C	1.241577	-0.373816	-1.582438
O	1.124148	-1.003171	-2.648719
C	1.029631	-1.768440	-0.117073
C	1.415904	-3.826578	-1.676415
C	0.705705	-3.851469	0.581833
C	0.815657	-5.206597	-1.338366
H	2.509689	-3.852231	-1.779744
H	0.985970	-3.344942	-2.559196
C	0.774664	-5.295378	0.206837
H	-0.209509	-5.257902	-1.730200
H	-0.075264	-5.871881	0.589544
H	1.393601	-6.021694	-1.787804
H	1.698131	-5.731752	0.616380
N	1.073648	-3.058052	-0.474967
N	0.623658	-1.856901	1.166884
N	0.411456	-3.135281	1.619859
C	0.368074	-0.743958	1.999626
C	-0.938176	-0.291785	2.172357
C	1.421215	-0.050208	2.590142
C	-1.195076	0.875165	2.880083
C	1.175321	1.125913	3.287758
C	-0.132262	1.581406	3.437186
F	2.657083	-0.494527	2.482491
F	2.176652	1.804849	3.824349
F	-0.364950	2.691151	4.111776
F	-2.430601	1.317749	3.023969
F	-1.937970	-0.947048	1.607543
C	0.121928	0.594149	-1.144531
C	-1.248281	-0.027854	-1.382716
C	0.290216	1.908202	-1.888557
H	0.240194	0.815107	-0.078668
C	-2.350482	0.678068	-0.622589
H	-1.487897	-0.051889	-2.456593
C	0.292382	1.946505	-3.289371
C	0.458136	3.106047	-1.184150
O	-2.109709	1.586243	0.145234
C	-3.760360	0.187139	-0.787779
C	0.451059	3.154889	-3.968338
H	0.186856	1.011485	-3.843862
C	0.616112	4.315815	-1.860585
H	0.457482	3.083371	-0.091914
C	-4.738395	0.704262	0.072342
C	-4.118673	-0.770916	-1.744096
C	0.611537	4.344226	-3.255846

```
 H      0.452315   3.167176  -5.060143
 H      0.742201   5.241152  -1.295016
 C     -6.057864   0.273826  -0.024497
 H     -4.437897   1.440113   0.819675
 C     -5.442902  -1.197527  -1.843522
 H     -3.371099  -1.188224  -2.419901
 H      0.734484   5.290403  -3.786246
 C     -6.411826  -0.676742  -0.985684
 H     -6.814802   0.677413   0.650106
 H     -5.718499  -1.940340  -2.593765
 H     -7.446968  -1.014193  -1.064089
 H     -1.260872  -1.083475  -1.055865
---------------------------------------
profile/chalcone_H_H
Frequencies, energies and thermodynamic properties:
 Lowest Vibrational Mode (1/cm) =                       24.7213
 2nd Lowest Vibrational Mode (1/cm) =                   34.1225
 E(RM062X) (a.u.) =                                   -653.298080528
 Thermal correction to Enthalpy (a.u.) =                0.241170
 Thermal correction to Gibbs Free Energy (a.u.) =        0.185514
 Total Entropy (cal/Kmol) =                            117.137
 Esp(RM062X) (a.u.) =                                 -654.017964564
Optimised cartesian coordinates (Angstrom):
 C      1.180364  -0.956546  -0.021644
 O      1.172685  -2.172840  -0.053522
 C     -0.084838  -0.176980   0.023084
 C     -1.261628  -0.822586  -0.055497
 C      2.489035  -0.217080  -0.011878
 C      3.655029  -0.969988   0.189189
 C      2.593176   1.165756  -0.211721
 C      4.901325  -0.352887   0.202252
 H      3.559407  -2.046515   0.336531
 C      3.844627   1.782261  -0.208237
 H      1.706561   1.774904  -0.388065
 C      4.997570   1.026803   0.002784
 H      5.802335  -0.946129   0.366943
 H      3.917609   2.858525  -0.372163
 H      5.974745   1.513087   0.010224
 C     -2.604819  -0.233396  -0.011827
 C     -2.831081   1.146141   0.141221
 C     -3.712483  -1.088338  -0.126951
 C     -4.127342   1.648538   0.175622
 H     -1.988276   1.832949   0.234808
 C     -5.011376  -0.584267  -0.092640
```

```
 H     -3.543506  -2.161028  -0.244546
 C     -5.221559   0.785904   0.058573
 H     -4.289011   2.721192   0.294992
 H     -5.860837  -1.263177  -0.183651
 H     -6.237053   1.184890   0.086137
 H     -1.221996  -1.911490  -0.164689
 H     -0.037470   0.905503   0.137669
---------------------------------------
```

profile/i

Frequencies, energies and thermodynamic properties:

```
 Lowest Vibrational Mode (1/cm) =                       123.3516
 2nd Lowest Vibrational Mode (1/cm) =                   224.2183
 E(RM062X) (a.u.) =                                   -361.199721699
 Thermal correction to Enthalpy (a.u.) =                0.106346
 Thermal correction to Gibbs Free Energy (a.u.) =        0.068820
 Total Entropy (cal/Kmol) =                            78.979
 Esp(RM062X) (a.u.) =                                 -361.607943816
```

Optimised cartesian coordinates (Angstrom):

```
 C      1.358384   1.301879   0.000064
 C     -0.019848   1.105959   0.000083
 C     -0.496497  -0.206380   0.000001
 C      1.607973  -1.088740  -0.000113
 C      2.188829   0.183113  -0.000035
 H      1.778969   2.308823   0.000126
 H     -0.728526   1.935176   0.000159
 H      2.240347  -1.981332  -0.000191
 H      3.274558   0.285782  -0.000053
 N      0.292584  -1.283659  -0.000097
 C     -1.965218  -0.477440   0.000014
 H     -2.222259  -1.561040  -0.000104
 O     -2.804114   0.385982   0.000083
---------------------------------------
```

profile/ii

Frequencies, energies and thermodynamic properties:

```
 Lowest Vibrational Mode (1/cm) =                       23.8088
 2nd Lowest Vibrational Mode (1/cm) =                   29.1913
 E(RM062X) (a.u.) =                                   -1446.16114662
 Thermal correction to Enthalpy (a.u.) =                0.290702
 Thermal correction to Gibbs Free Energy (a.u.) =        0.215162
 Total Entropy (cal/Kmol) =                            158.987
 Esp(RM062X) (a.u.) =                                 -1447.82595917
```

Optimised cartesian coordinates (Angstrom):

```
 C      2.018712   2.970640   1.033795
 C      1.792124   4.180516   0.381528
```

```
 C      0.721292   4.276440  -0.507075
 C     -0.077609   3.149282  -0.704104
 C      1.153640   1.908212   0.776990
 H      2.830611   2.798693   1.740464
 H      2.442224   5.040006   0.559117
 H      0.506754   5.201878  -1.042662
 H     -0.920179   3.181678  -1.403432
 N      0.130370   1.993575  -0.075023
 C      1.388667   0.571541   1.527995
 O      2.475432   0.509977   2.251454
 C      1.403245  -0.497805   0.404405
 C      3.961412  -0.940361   0.174390
 C      2.186864  -1.949221  -1.038440
 C      4.475525  -2.207951  -0.540346
 H      4.136802  -0.897324   1.251923
 H      4.321257  -0.009435  -0.283861
 C      3.417601  -2.571486  -1.612474
 H      5.468010  -2.050161  -0.976300
 H      3.637199  -2.101204  -2.582667
 H      4.547899  -3.026250   0.188802
 H      3.306630  -3.650056  -1.771447
 N      2.518451  -1.048209  -0.064058
 N      0.426097  -1.102548  -0.293876
 N      0.901923  -2.008099  -1.202889
 C     -0.965665  -0.879390  -0.179353
 C     -1.629987  -0.165105  -1.174463
 C     -1.672991  -1.357988   0.919085
 C     -2.991308   0.079802  -1.067694
 C     -3.037369  -1.111423   1.034837
 C     -3.693426  -0.398591   0.037034
 F     -1.050848  -2.027142   1.871307
 F     -3.711930  -1.560732   2.079180
 F     -4.987906  -0.169203   0.138382
 F     -3.622287   0.773530  -2.000401
 F     -0.962999   0.302166  -2.208713
 H      0.400810   0.379081   2.044892
--------------------------------------
profile/iii
Frequencies, energies and thermodynamic properties:
 Lowest Vibrational Mode (1/cm) =                          32.1330
 2nd Lowest Vibrational Mode (1/cm) =                      40.4330
 E(RM062X) (a.u.) =                                     -1446.18804670
 Thermal correction to Enthalpy (a.u.) =                   0.291103
 Thermal correction to Gibbs Free Energy (a.u.) =          0.216958
```

Total Entropy (cal/Kmol) = 156.051

Esp(RM062X) (a.u.) = -1447.84265461

Optimised cartesian coordinates (Angstrom):

C	2.873919	-2.501524	-1.125525
C	4.110288	-3.110560	-0.970404
C	5.053019	-2.540401	-0.111358
C	4.690754	-1.371000	0.556310
C	2.595519	-1.319052	-0.400764
H	2.125283	-2.910943	-1.803083
H	4.343344	-4.023149	-1.523102
H	6.035839	-2.986139	0.041466
H	5.400422	-0.891231	1.239431
N	3.505267	-0.782677	0.433770
C	1.285194	-0.703685	-0.507112
O	0.273995	-1.447315	-1.088349
H	0.129586	-2.248729	-0.559234
C	0.946965	0.552610	-0.056690
C	3.117836	2.057852	0.061911
C	0.893000	2.709057	0.500194
C	2.995467	3.594206	-0.018018
H	3.533603	1.613395	-0.850731
H	3.716841	1.714643	0.914013
C	1.690883	3.950293	0.726143
H	3.873999	4.092986	0.407017
H	1.862230	4.081890	1.805371
H	2.904512	3.896360	-1.070641
H	1.188838	4.845330	0.342424
N	1.724244	1.651758	0.236715
N	-0.347706	1.055628	0.107744
N	-0.353862	2.407644	0.433022
C	-1.567092	0.377080	0.098593
C	-1.752850	-0.812898	0.807227
C	-2.664216	0.911673	-0.585762
C	-2.973677	-1.474790	0.799810
C	-3.900152	0.275197	-0.565098
C	-4.057116	-0.921772	0.125318
F	-2.536686	2.024452	-1.288391
F	-4.925711	0.796555	-1.220484
F	-5.228410	-1.535231	0.134449
F	-3.119260	-2.610219	1.465830
F	-0.748654	-1.338455	1.494868

----

profile/iv

Frequencies, energies and thermodynamic properties:

Lowest Vibrational Mode (1/cm) = 8.1696

2nd Lowest Vibrational Mode (1/cm) = 29.9425

E(RM062X) (a.u.) = -1807.40614185

Thermal correction to Enthalpy (a.u.) = 0.400469

Thermal correction to Gibbs Free Energy (a.u.) = 0.310493

Total Entropy (cal/Kmol) = 189.369

Esp(RM062X) (a.u.) = -1809.46711265

Optimised cartesian coordinates (Angstrom):

C	1.752362	-1.710419	1.240054
C	2.580422	-2.011122	2.315004
C	3.173956	-0.963892	3.022660
C	2.896962	0.341069	2.623965
C	1.566912	-0.367417	0.900281
H	1.241913	-2.469852	0.648546
H	2.760367	-3.048514	2.604136
H	3.829620	-1.151672	3.873494
H	3.329077	1.187045	3.167324
N	2.111804	0.635420	1.585221
C	0.745960	-0.048259	-0.353257
O	-0.086615	-1.008711	-0.741565
C	-0.028202	1.260389	-0.096357
C	1.614167	3.317732	-0.079863
C	-0.730704	3.317092	0.192215
C	1.170999	4.626716	0.597779
H	1.866048	3.465442	-1.139112
H	2.419350	2.782485	0.431336
C	-0.342376	4.753487	0.308548
H	1.329342	4.541548	1.681687
H	-0.901492	5.275020	1.093417
H	1.743537	5.484100	0.228165
H	-0.534801	5.258438	-0.650141
N	0.372577	2.530101	-0.007093
N	-1.364003	1.329882	0.046888
N	-1.811517	2.604936	0.232394
C	-2.309579	0.274958	0.075818
C	-2.217844	-0.733940	1.030732
C	-3.371853	0.284176	-0.821334
C	-3.168168	-1.742755	1.074305
C	-4.334915	-0.719424	-0.775398
C	-4.232426	-1.728590	0.174749
F	-3.464543	1.230807	-1.737573
F	-5.344168	-0.714326	-1.630746
F	-5.144458	-2.681333	0.223621
F	-3.080349	-2.709747	1.973221

```
 F     -1.220856  -0.738385   1.892963
 C      1.721864   0.262678  -1.613149
 C      2.917300  -0.660309  -1.564413
 H      2.099980   1.295871  -1.583599
 C      2.842331  -1.942166  -2.118232
 C      5.034436  -1.042939  -0.773476
 C      3.927432  -2.799884  -1.962570
 H      1.940812  -2.240435  -2.652354
 C      5.049151  -2.346728  -1.269788
 H      5.904647  -0.650199  -0.237566
 H      3.899614  -3.810597  -2.374664
 H      5.922797  -2.982257  -1.121385
 N      3.998561  -0.217180  -0.914883
 O      0.901311   0.038725  -2.704561
 H      0.219915  -0.564523  -2.261007
---------------------------------------
profile/ix
Frequencies, energies and thermodynamic properties:
 Lowest Vibrational Mode (1/cm) =                        18.6606
 2nd Lowest Vibrational Mode (1/cm) =                    28.2605
 E(RM062X) (a.u.) =                                    -1014.54643251
 Thermal correction to Enthalpy (a.u.) =                 0.350822
 Thermal correction to Gibbs Free Energy (a.u.) =         0.278758
 Total Entropy (cal/Kmol) =                             151.673
 Esp(RM062X) (a.u.) =                                  -1015.66686665
Optimised cartesian coordinates (Angstrom):
 C     -2.554965  -2.406745   1.230101
 C     -3.633952  -3.204733   0.860232
 C     -4.168449  -3.053191  -0.416869
 C     -3.598504  -2.107661  -1.273372
 C     -2.057859  -1.491533   0.298920
 H     -2.091606  -2.474506   2.214276
 H     -4.051956  -3.932825   1.557345
 H     -5.013921  -3.654654  -0.752550
 H     -3.997719  -1.967541  -2.282104
 N     -2.566953  -1.344343  -0.925937
 C     -0.891479  -0.617171   0.685349
 O     -0.385447  -0.717687   1.779179
 C     -0.430966   0.428988  -0.320700
 C      1.035843   0.767240  -0.087410
 C     -1.337458   1.643187  -0.191813
 H     -0.560652   0.006974  -1.323222
 C      1.923416  -0.434214  -0.340121
 H      1.351036   1.590517  -0.747521
```

C	-2.081879	2.096738	-1.285975
C	-1.436877	2.327900	1.026300
O	1.447038	-1.505539	-0.649451
C	3.407744	-0.268512	-0.201432
C	-2.899735	3.221074	-1.170086
H	-2.021230	1.557854	-2.233984
C	-2.255171	3.451024	1.143877
H	-0.872793	1.972470	1.892681
C	4.224062	-1.380222	-0.448963
C	3.991739	0.950650	0.164755
C	-2.987573	3.901986	0.044605
H	-3.472718	3.565181	-2.033164
H	-2.323088	3.974670	2.099219
C	5.606553	-1.275482	-0.333540
H	3.751701	-2.322122	-0.731818
C	5.377492	1.053840	0.280777
H	3.370421	1.825007	0.363574
H	-3.628467	4.780759	0.135598
C	6.184395	-0.056737	0.031530
H	6.238120	-2.143923	-0.527854
H	5.828897	2.005120	0.566764
H	7.268901	0.027026	0.122716
H	1.202759	1.107643	0.946790

----

profile/v

Frequencies, energies and thermodynamic properties:

Lowest Vibrational Mode (1/cm) = 9.7346

2nd Lowest Vibrational Mode (1/cm) = 31.0747

E(RM062X) (a.u.) = -1807.40614233

Thermal correction to Enthalpy (a.u.) = 0.400510

Thermal correction to Gibbs Free Energy (a.u.) = 0.310907

Total Entropy (cal/Kmol) = 188.584

Esp(RM062X) (a.u.) = -1809.46710051

Optimised cartesian coordinates (Angstrom):

C	1.761768	-1.694667	1.254333
C	2.597963	-1.989244	2.324740
C	3.199505	-0.938010	3.019551
C	2.922068	0.364674	2.613498
C	1.576426	-0.353829	0.906354
H	1.244830	-2.456837	0.671955
H	2.778289	-3.024838	2.620014
H	3.861996	-1.120882	3.866162
H	3.360734	1.213618	3.146879
N	2.129028	0.653135	1.579119

C	0.749954	-0.042495	-0.345254
O	-0.080896	-1.006447	-0.728386
C	-0.028733	1.265142	-0.093610
C	1.603416	3.331453	-0.086343
C	-0.741801	3.320296	0.180755
C	1.151796	4.643212	0.580269
H	1.857079	3.472484	-1.146032
H	2.410051	2.804454	0.431030
C	-0.361584	4.759529	0.287244
H	1.308616	4.567588	1.665095
H	-0.925070	5.283755	1.067196
H	1.720402	5.500734	0.204956
H	-0.554993	5.256285	-0.675523
N	0.365879	2.537399	-0.010617
N	-1.365315	1.329357	0.045862
N	-1.819189	2.603255	0.222943
C	-2.306996	0.271029	0.078073
C	-2.212348	-0.734413	1.036402
C	-3.368963	0.273646	-0.819509
C	-3.159529	-1.746022	1.083097
C	-4.329072	-0.732707	-0.770278
C	-4.223674	-1.738366	0.183203
F	-3.464410	1.216501	-1.739361
F	-5.337987	-0.733499	-1.626044
F	-5.132679	-2.693833	0.235181
F	-3.068749	-2.709886	1.985109
F	-1.215663	-0.732803	1.898932
C	1.721858	0.265812	-1.606470
C	2.908631	-0.668792	-1.565699
H	2.109562	1.295328	-1.574849
C	2.815846	-1.951720	-2.114406
C	5.028669	-1.070164	-0.791928
C	3.893499	-2.819913	-1.964964
H	1.906674	-2.243018	-2.639306
C	5.025816	-2.375784	-1.283610
H	5.907437	-0.684300	-0.265033
H	3.851857	-3.831788	-2.373024
H	5.894213	-3.019707	-1.140662
N	3.999836	-0.234627	-0.927126
O	0.894499	0.052820	-2.695640
H	0.216126	-0.553829	-2.254967

----

profile/vii

Frequencies, energies and thermodynamic properties:

```
 Lowest Vibrational Mode (1/cm) =                        9.9472
 2nd Lowest Vibrational Mode (1/cm) =                    14.0529
 E(RM062X) (a.u.) =                                   -2099.51521621
 Thermal correction to Enthalpy (a.u.) =                 0.534278
 Thermal correction to Gibbs Free Energy (a.u.) =        0.428664
 Total Entropy (cal/Kmol) =                            222.284
 Esp(RM062X) (a.u.) =                                 -2101.87931375
Optimised cartesian coordinates (Angstrom):
 C     -1.691401   2.807071   1.894034
 C     -2.992609   3.284064   2.034850
 C     -4.040378   2.592169   1.430404
 C     -3.735045   1.442474   0.704449
 C     -1.492024   1.652031   1.134528
 H     -0.835748   3.308820   2.340240
 H     -3.186802   4.189548   2.612734
 H     -5.073019   2.930721   1.518107
 H     -4.527921   0.854601   0.229111
 N     -2.490085   0.994650   0.553823
 C     -0.066950   1.135376   0.884053
 O      0.813393   1.944185   1.550012
 H      1.797223   1.746249   1.152493
 C      0.083290  -0.294711   1.452651
 C      1.890567   0.046679   3.346860
 C      0.817754  -1.955354   2.681681
 C      2.644672  -1.167383   3.922689
 H      1.327807   0.613097   4.099220
 H      2.536327   0.712307   2.771584
 C      1.690901  -2.376051   3.813513
 H      3.535709  -1.352363   3.307502
 H      2.201138  -3.325035   3.614143
 H      2.969406  -0.982849   4.952362
 H      1.070639  -2.498402   4.714064
 N      0.921278  -0.610745   2.442328
 N     -0.488268  -1.473584   1.118082
 N     -0.035554  -2.508400   1.882048
 C     -1.442517  -1.788880   0.114643
 C     -2.800723  -1.765269   0.422879
 C     -1.028988  -2.234582  -1.137520
 C     -3.743521  -2.063456  -0.549656
 C     -1.968617  -2.544124  -2.116144
 C     -3.324311  -2.469102  -1.814629
 F      0.253763  -2.347285  -1.418929
 F     -1.577827  -2.944780  -3.313314
 F     -4.218205  -2.771148  -2.734112
```

F -5.035279 -1.980955 -0.276316
F -3.199714 -1.423045 1.629720
C 0.209762 1.069274 -0.662439
C 1.507438 0.370721 -1.018108
C 0.068875 2.468251 -1.245674
H -0.618033 0.477514 -1.080536
C 2.713142 0.515501 -0.390191
H 1.464717 -0.256575 -1.906901
C -1.120623 2.858971 -1.871976
C 1.114400 3.397146 -1.148304
O 2.953756 1.287583 0.635206
C 3.910554 -0.235650 -0.907285
C -1.269812 4.148697 -2.385971
H -1.940446 2.140890 -1.955457
C 0.965689 4.686139 -1.657660
H 2.047757 3.097621 -0.667040
C 3.811612 -1.413699 -1.663223
C 5.187282 0.250425 -0.593553
C -0.226981 5.067518 -2.277117
H -2.203239 4.433206 -2.875612
H 1.788553 5.398766 -1.573261
C 4.956512 -2.068299 -2.114282
H 2.830005 -1.836834 -1.882802
C 6.333419 -0.400466 -1.049180
H 5.258402 1.152047 0.015523
H -0.339533 6.076780 -2.677624
C 6.223086 -1.562090 -1.814181
H 4.859191 -2.986524 -2.696927
H 7.319124 -0.000108 -0.803114
H 7.118605 -2.076486 -2.167794

----

profile/viii

Frequencies, energies and thermodynamic properties:

Lowest Vibrational Mode (1/cm) = 14.1981

2nd Lowest Vibrational Mode (1/cm) = 21.3561

E(RM062X) (a.u.) = -2099.50948991

Thermal correction to Enthalpy (a.u.) = 0.534633

Thermal correction to Gibbs Free Energy (a.u.) = 0.429138

Total Entropy (cal/Kmol) = 222.033

Esp(RM062X) (a.u.) = -2101.87845249

Optimised cartesian coordinates (Angstrom):

C -3.292405 1.765350 0.006602
C -4.605080 2.060078 0.362914
C -5.299847 1.166318 1.177878

C	-4.640430	0.014461	1.603889
C	-2.729117	0.575863	0.471845
H	-2.678833	2.406541	-0.624928
H	-5.084466	2.975692	0.010361
H	-6.330023	1.354618	1.481922
H	-5.151259	-0.705594	2.251575
N	-3.386133	-0.277172	1.256925
C	-1.302149	0.212327	0.003927
O	-0.707665	1.155615	-0.687004
C	-0.481621	-0.119175	1.290093
C	-1.527728	-2.083347	2.706937
C	0.510048	-0.957622	3.057271
C	-0.602321	-2.942702	3.586210
H	-1.929503	-2.610745	1.838163
H	-2.355444	-1.629497	3.264115
C	0.461839	-1.974550	4.146400
H	-1.162465	-3.455954	4.375554
H	0.131895	-1.494278	5.080007
H	-0.110379	-3.692553	2.953242
H	1.438187	-2.440296	4.321135
N	-0.615032	-1.010530	2.278232
N	0.712581	0.454712	1.533132
N	1.339626	-0.061463	2.627848
C	1.355883	1.512499	0.840237
C	0.808446	2.793161	0.832803
C	2.587923	1.281814	0.243158
C	1.471840	3.826237	0.190143
C	3.262225	2.316885	-0.399263
C	2.705486	3.589218	-0.416292
F	3.111766	0.067371	0.246584
F	4.431022	2.093548	-0.979609
F	3.341263	4.580127	-1.016133
F	0.949900	5.043167	0.157820
F	-0.348495	3.023382	1.420727
C	-1.393777	-1.177821	-0.806626
C	-0.014417	-1.484540	-1.386904
C	-2.445784	-1.017484	-1.876763
H	-1.704192	-1.987608	-0.133595
C	0.880233	-2.334337	-0.513501
H	-0.091166	-2.007740	-2.355194
C	-2.232039	-0.168577	-2.972774
C	-3.679682	-1.670934	-1.766726
O	0.487944	-2.835026	0.522864
C	2.291813	-2.566510	-0.970863

```
 C     -3.229258   0.020005  -3.929675
 H     -1.285142   0.367251  -3.045012
 C     -4.678221  -1.483884  -2.723565
 H     -3.860067  -2.327443  -0.911784
 C      2.792830  -2.021208  -2.159648
 C      3.134917  -3.328373  -0.152000
 C     -4.455845  -0.636157  -3.809061
 H     -3.049168   0.687443  -4.774975
 H     -5.633354  -2.002609  -2.619800
 C      4.120266  -2.241009  -2.524043
 H      2.153388  -1.417693  -2.804812
 C      4.460479  -3.545230  -0.515424
 H      2.732000  -3.740926   0.774069
 H     -5.235072  -0.487568  -4.559021
 C      4.954564  -3.000680  -1.703184
 H      4.506171  -1.813824  -3.450917
 H      5.113372  -4.137697   0.127711
 H      5.994753  -3.167693  -1.988873
 H      0.487707  -0.524176  -1.597228
---------------------------------------
profile/x
Frequencies, energies and thermodynamic properties:
 Lowest Vibrational Mode (1/cm) =                        45.2274
 2nd Lowest Vibrational Mode (1/cm) =                    71.7271
 E(RM062X) (a.u.) =                                   -722.448528662
 Thermal correction to Enthalpy (a.u.) =                 0.216554
 Thermal correction to Gibbs Free Energy (a.u.) =        0.164559
 Total Entropy (cal/Kmol) =                             109.432
 Esp(RM062X) (a.u.) =                                 -723.253097044
Optimised cartesian coordinates (Angstrom):
 C     -4.186764   1.012807   0.000082
 C     -2.828656   1.304164  -0.000057
 C     -1.912055   0.240437   0.000006
 C     -3.626113  -1.312294   0.000354
 C     -4.602539  -0.319707   0.000288
 H     -4.920265   1.821161   0.000032
 H     -2.459698   2.328158  -0.000217
 H     -3.903100  -2.369744   0.000523
 H     -5.658829  -0.588106   0.000404
 C     -0.464567   0.504154  -0.000120
 C      0.464551  -0.504060  -0.000181
 O     -0.140172   1.812744  -0.000177
 O      0.140109  -1.812637  -0.000306
 C      1.912053  -0.240400  -0.000104
```

C	2.828600	-1.304174	-0.000280
C	4.186724	-1.012892	-0.000180
H	2.459586	-2.328147	-0.000497
C	3.626202	1.312239	0.000286
C	4.602573	0.319599	0.000103
H	4.920181	-1.821286	-0.000318
H	3.903246	2.369673	0.000522
H	5.658877	0.587941	0.000192
N	2.323322	1.040159	0.000188
N	-2.323249	-1.040144	0.000220
H	0.855562	1.857204	-0.000392
H	-0.855625	-1.857058	-0.000426
